# Specific *versus* Non-Specific Immune Responses in an Invertebrate Species Evidenced by a Comparative *de novo* Sequencing Study

**DOI:** 10.1371/journal.pone.0032512

**Published:** 2012-03-12

**Authors:** Emeline Deleury, Géraldine Dubreuil, Namasivayam Elangovan, Eric Wajnberg, Jean-Marc Reichhart, Benjamin Gourbal, David Duval, Olga Lucia Baron, Jérôme Gouzy, Christine Coustau

**Affiliations:** 1 INRA/CNRS/UNS, Institut Sophia Agrobiotech, Sophia Antipolis, France; 2 Department of Biotechnology, Periyar University, Salem, India; 3 UdS, UPR 9022 CNRS, IBMC, 15 rue Rene Descartes, Strasbourg, France; 4 CNRS, UMR 5244, Ecologie et Evolution des Interactions (2EI), Université de Perpignan Via Domitia, Perpignan, France; 5 INRA/CNRS, UMR441/2594, Laboratoire Interactions Plantes Micro-organismes, Chemin de Borde Rouge, Castanet Tolosan, France; French National Centre for Scientific Research - Université Aix-Marseille, France

## Abstract

Our present understanding of the functioning and evolutionary history of invertebrate innate immunity derives mostly from studies on a few model species belonging to ecdysozoa. In particular, the characterization of signaling pathways dedicated to specific responses towards fungi and Gram-positive or Gram-negative bacteria in *Drosophila melanogaster* challenged our original view of a non-specific immunity in invertebrates. However, much remains to be elucidated from lophotrochozoan species. To investigate the global specificity of the immune response in the fresh-water snail *Biomphalaria glabrata*, we used massive Illumina sequencing of 5′-end cDNAs to compare expression profiles after challenge by Gram-positive or Gram-negative bacteria or after a yeast challenge. 5′-end cDNA sequencing of the libraries yielded over 12 millions high quality reads. To link these short reads to expressed genes, we prepared a reference transcriptomic database through automatic assembly and annotation of the 758,510 redundant sequences (ESTs, mRNAs) of *B. glabrata* available in public databases. Computational analysis of Illumina reads followed by multivariate analyses allowed identification of 1685 candidate transcripts differentially expressed after an immune challenge, with a two fold ratio between transcripts showing a challenge-specific expression versus a lower or non-specific differential expression. Differential expression has been validated using quantitative PCR for a subset of randomly selected candidates. Predicted functions of annotated candidates (approx. 700 unisequences) belonged to a large extend to similar functional categories or protein types. This work significantly expands upon previous gene discovery and expression studies on *B. glabrata* and suggests that responses to various pathogens may involve similar immune processes or signaling pathways but different genes belonging to multigenic families. These results raise the question of the importance of gene duplication and acquisition of paralog functional diversity in the evolution of specific invertebrate immune responses.

## Introduction

Our perception of invertebrate immunity dramatically changed in the last decade. Initially thought to rely on non-specific recognition and killing processes, it is now known to be complex and diversified across invertebrate phyla [1], [2], [3]. One of the major breakthroughs challenging the original view of a simple system was the characterization of signaling pathways dedicated to specific responses towards fungi and Gram-positive or Gram-negative bacteria in *Drosophila melanogaster* [4], [5]. Despite these significant progresses, a comprehensive understanding of the evolutionary history and the functioning of invertebrate immunity is now hindered by the enormous diversity in invertebrate phyla correlated with a diversity of organismal ecologies and associated pathogens, parasites or symbionts. For example, recent studies showed that the immune system of some insect species is lacking at least elements of one of the three major signaling pathways characterized in *D. melanogaster*, suggesting that part of the immune response may rely on different and uncharacterized processes [6], [7]. In addition, most of our knowledge comes from a few model species belonging to deuterostoma or ecdysozoa (e.g. *Strongylocentrotus*, *Drosophila*, *Anopheles*, *Caenorhabditis*) and much remains to be elucidated from lophotrochozoan species.

*Biomphalaria glabrata*, one of the best-studied lophotrochozoan species to date, is a fresh water gastropod snail from tropical countries that transmits the human blood fluke *Schistosoma mansoni*. Because of its role in the transmission of this important human parasite causing schistosomiasis (or bilharziosis), *B. glabrata* immunity has long been investigated with a focus on the response to parasites and in particular to helminths [8], [9], [10], [11], [12], [13], [14], [15], [16], [17], [18], [19], [20], [21]. The existence of the somatically diversified FREPs (Fibrinogen Related proteins) involved in the binding of parasite glycoproteins (SmPoMuc) was a recent and remarkable discovery [22], [23], [24], [25]. A couple of studies also investigated for the first time the antimicrobial response of *B. glabrata* [20], [26]. In particular, a study based on custom-made oligo-array of a thousand sequences compared the responses of *B. glabrata* to wounding, exposure to Gram-negative or Gram-positive bacteria and to trematode parasites [26]. The results showed a clear difference between expression profiles of snails exposed to the two trematode species and further confirmed the specificity of the snail-trematode molecular interactions [26]. Expression profiles from snails challenged with *Escherichia coli* or *Micrococcus luteus* were different but overlapping and few candidates among the differentially expressed transcripts presented a function [26]. The question of the specificity of *B. glabrata* immune response to microbial infection therefore deserved further investigation.

The genome of *B. glabrata* has been the subject of sequencing efforts for several years now and the first assemblies are available for blast searches (see http://biology.unm.edu/biomphalaria-genome/index.html for details on the sequencing progress). However, inherent properties of *B. glabrata* genome interfere with the assembly efforts and the genome assembly is still very fragmented and not annotated. Despite this continuous sequencing effort, it cannot be anticipated when genomic data will be available for gene prediction (including immune-related genes) or for development of genome-wide micro-arrays. It is therefore crucial to keep gaining insights into the *B. glabrata* immune response while maintaining a gene discovery effort through transcriptomic studies. For this reason, we investigated the relative specificity of *B. glabrata* immune responses using a massive sequencing approach that does not require previous knowledge of immune transcripts. In this study we compared the transcriptomes of *B. glabrata* snails after challenges by Gram-negative and Gram-positive bacteria or by yeast. Since no natural pathogenic micro-organisms for *B. glabrata* are available to date for experimental infections, we mimicked infections by exposing the snails to three model organisms with sequenced genomes (*Echerichia coli*, *Bacillus cereus* and *Saccharomyces cerevisiae*). This study provides the first large-scale database of annotated transcripts in *B. glabrata* and shows that a surprisingly high proportion of transcripts are over-expressed in a challenge-specific manner.

## Results and Discussion

### Strategy

**Figure 1:**
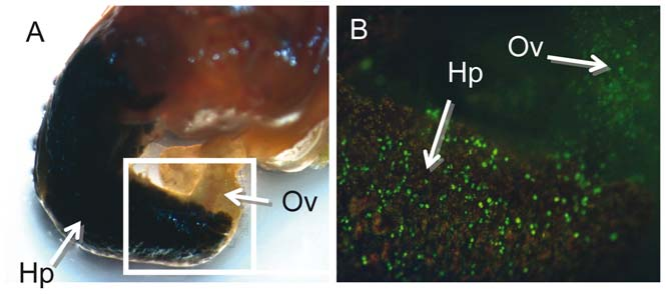
Presence of bacteria in *B. glabrata* tissues after balneation in a bacterial suspension. The efficiency of the balneation procedure used to challenge the immune system of *B. glabrata* has been tested using fluorescent *E. coli* (DH5 α/GFP). Snails have been removed from their shell to allow observation and rinsed several times to remove external bacteria. (A) light and (B) UV observation of fluorescent bacteria in the snail body, with a preferential location in the hepatopancreas (Hp) and ovotestis (Ov).

**Figure 2:**
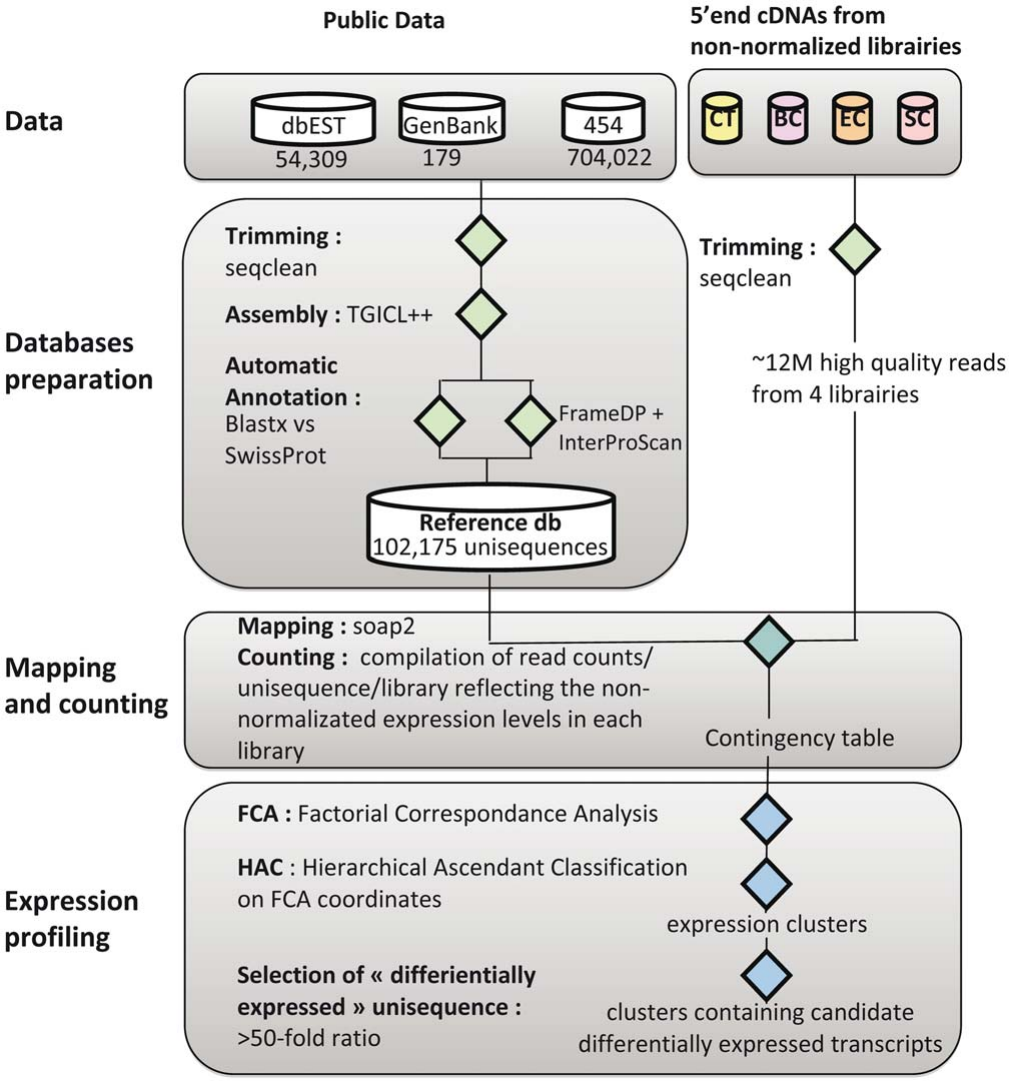
Schematic representation of the computational and data analysis pipeline.

The overall strategy we have developed to compare the transcriptomes of *B. glabrata* after immune challenges with Gram-positive or Gram-negative bacteria and fungi consisted in several key steps: 1) *Immune challenges* have been performed using organisms with known genomes in order to identify microbial sequences that could contaminate host cDNA libraries. Challenges consisted in exposure to the micro-organisms, mimicking natural infections (fig. 1) and minimizing non-specific stress responses induced by injection techniques. The time-point of 6 hours after exposure has been selected after a series of pilot experiments using previously identified candidate transcripts [11], [16] and time points from 2 hr to 72 hr post-exposure (PE) (results not shown); 2) *Transcriptome sampling* has been performed through massive sequencing of non-normalized oligo-capped 5′-end cDNA libraries [27], a method previously shown to allow quantitative comparison of transcriptomes [28]; 3) *The reference database* used for mapping the 5′-end cDNA reads has been processed and annotated from all ESTs available on public databases at the time of the study (see fig. 2 for the computational pipeline); 4) *The Data mining* strategy involved a factorial correspondence analysis (FCA) followed by a cluster analysis aimed at identifying clusters of transcripts showing similar expression profiles.

### Analysis of the reference *B. glabrata* transcriptomic database

**Figure 3:**
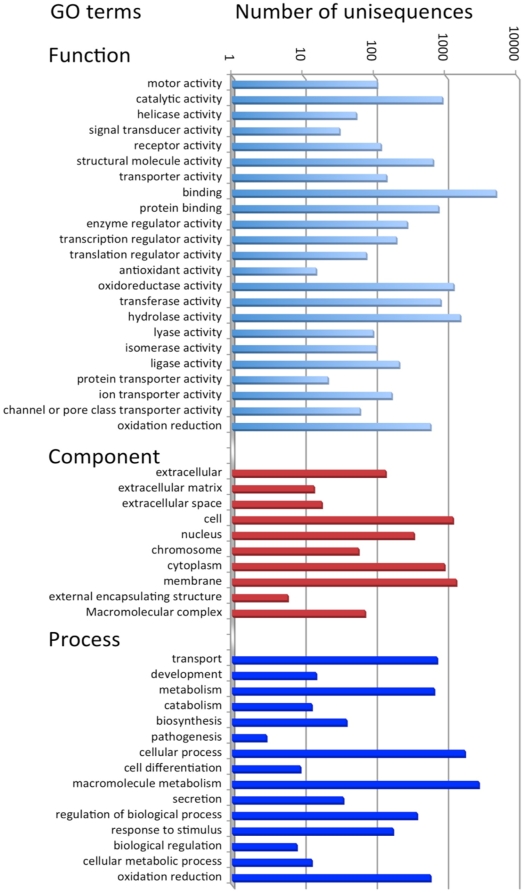
Classification of the unisequences (n = 9,074) according to their GO terms.

In order to map the 5′-end cDNA reads to a reference transcriptomic database, we processed a database using all *B. glabrata* transcript sequences available on public databases. These sequences originate from various laboratories using different approaches such as random sequencing of ESTs or ORESTES (see [29] for a review) as well as massive 454 sequencing from the Genome Sequencing Center (GSC) at Washington University (*Biomphalaria glabrata* genome project ID:12879). A total number of 758,510 sequences were trimmed and filtered to remove low quality and contaminant sequences (see table 1 for basic statistics on the reference database). Sequences were then automatically assembled and clustered in 43,238 contigs and 58,937 singletons. These 102,175 unisequences ranged in size from 100 bp (sequences shorter than 100 bp have been removed from the database) to 7,118 bp. Although large size contigs are well represented in this database, the average size is 353 bp (Table 1), due to the important number of unclustered 454 sequences around 260 bp (fig. S1). These sequences also explain the high number of singletons (58,937) present in this database. A first automatic annotation work consisted in the translation and peptide detection followed by a search for InterPro domains (IPR) [30]. It appeared that 12,5% of the unisequences presented an InterPro annotation and 8.9% also presented a GO term annotation (Table 1). The figure 3 shows the distribution of the unisequences according to their GO terms. A second annotation effort by searching the SwissProt database using the BLASTx program [31] resulted in the annotation of 18.6% of the sequences. The 102,175 unique sequences (consensus sequences from contigs or singleton sequences) have been deposited in the *Biomphalaria glabrata* database [32] as part of the joined effort of the collaborative consortium “*Biomphalaria glabrata* genome initiative” [33] and they can be used for BLAST searches. The sequences of the 43,238 contigs with automatic annotation are provided in the supporting file Text S1.

**Table 1:** Statistics on the reference transcriptomic database.

Total number of redundant sequences	758,510
Number of valid sequences	718,342
Number of contigs	43,238
Number of singletons	58,937
Number of unisequences	102,175
Average size of unisequences	353 bp
Maximum length	7,118 bp
Number of unisequences with a hit vs SwissProt	18,970 (18,6%)
Number of unisequences containing an InterPro Domain (IPR)	12,798 (12,5%)
Number of unisequences with a GO term annotation	9,074 (8,9%)

The *B. glabrata* transcriptomic database processed in this study displays a high number of unisequences and a modest proportion of annotated unisequences, both characteristic of the biological model. Firstly, *B. glabrata* is a highly polymorphic species known to present gene families such as FREPs that are somatically diversified [24], and the parameters used to assemble the sequences were highly stringent. The number of unisequences of this database therefore reflects the number of potential transcripts (including polymorphic and alternative spliced variants) rather than the number of genes. Using a lower stringency in the database would have decreased the number of unisequences but would have been detrimental to the identification of sequence polymorphisms and diversity. In addition, it is possible that a portion of the unisequences belong to xenobiotic organisms such as commensals, contributing to increase the number of unisequences. The future annotation of the *B. glabrata* genome should allow identification of foreign sequences. The database annotated here encompasses the high transcript diversity while remaining useful for future gene mining studies.

Secondly, the high proportion of transcripts showing no similarity hit is consistent with previous studies on mollusk species [11], [20], [34]. This repeated observation suggests that a substantial part of the genes are not conserved between these lophotrochozoan species and the deuterostome or ecdysozoan species commonly used in functional genomic studies [34]. Substantial efforts in functionally characterizing unknown proteins from these species are therefore needed and the results from large-scale expression studies will be useful for identification of functionally relevant candidates for further studies.

### Analysis of 5′-end cDNA libraries

**Figure 4:**
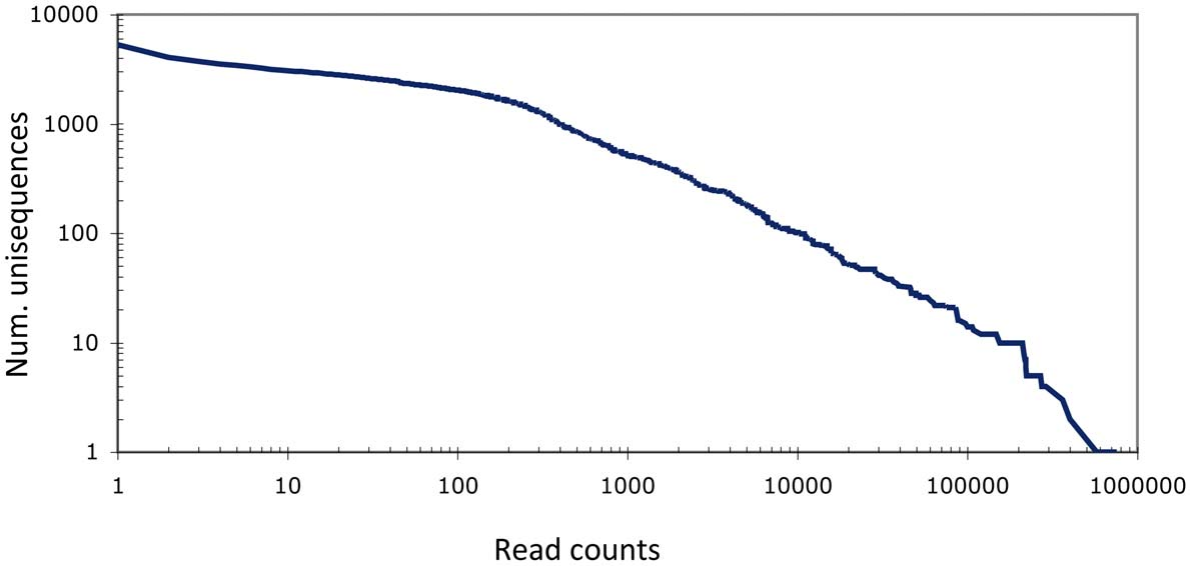
Reverse cumulative distribution for the number of different unisequences (n = 5,308) that have at least a given number of reads mapping to them. The two axes are shown on a logarithmic scale.

Illumina sequencing of 5′-end cDNAs from the four libraries provided 36 bp sequences referred to as “reads” in this work. The total number of reads obtained for the four libraries ranged from approximately 3 to 5 millions each. Erroneous sequences (low quality, low complexity and contaminant sequences) represented approximately 20% of the reads (Table 2). The remaining high quality reads from the four non-normalized libraries were mapped to the cDNA unisequences from the reference database using SAOPaligner/soap2 [35] and automatically scored. It appeared that approximately 20% of the reads did not map to any unisequence, probably due to the absence of the 5′-end cDNA sequence of the corresponding transcripts in the reference database. A consistent percentage of the raw Illumina reads from each library remained in the analysis (Table 2) and mapped a total of 5,308 unisequences. The distribution of the number of reads by unisequence (fig. 4) was consistent with distributions previously observed with 5′-end Illumina reads [36].

**Table 2:** Statistics on the 5′-end cDNA libraries.

	Ct	Bc	Ec	Sc
Number of reads	2,951,740	4 946,891	2 967,676	4 098,362
Erroneous reads	483,963	2,431,528	517,748	911,295
Total high quality reads (%)	2 467,777 (83.6%)	3 604,363 (72.8%)	2 449,928 (82.5%)	3 187,067 (77.7%)
% reads mapping a cDNA unisequence	55.3.%	61..07%	62.25%	53.57%

Libraries have been prepared from sham-challenged control snails (Ct), or snails challenged by *B. cereus* (Bc) *E. coli* (Ec) or *S. cerevisiae* (Sc).

### Comparison of gene expression profiles

At this stage of the pipeline, we obtained a contingency table where each of the 5308 unisequences mapped by at least one read (5,308 rows) was described by the number of mapped reads scored from each library (4 columns: control, *E. coli*-, *B. cereus*- or *S. cerevisiaes*-exposed snails). These counts are considered as the non-normalized expression levels in each library. When comparing the distributions of read counts in the four libraries, it appeared that each library was significantly different from the 3 others (chi-square test, P<10–12), showing that response to each immune challenge is associated with a particular expression profile. Then, in order to focus on the most differentially expressed transcripts we applied two filters. Firstly we removed unisequences mapped by less than 100 reads, considering that they were not represented enough for differential expression detection. Then, we selected unisequences showing at least a fifty-fold difference in their expression levels between at least two libraries. This arbitrary ratio is high because it takes into account both the high variation in gene expression level reported in this snail species after immune challenge [11], [12], [13], [16], [37], [38] and the over-estimated variations in expression levels resulting from Illumina/Solexa sequencing technologies as compared to quantitative PCR expression studies [39]. For this, the number of read counts of each unisequence in each library was normalized by the number of counts (mapped reads) of the library. After applying these two filters, 1685 unisequences remained in the analysis. This proportion of differentially expressed candidate transcripts is consistent with estimates from other studies. For example, in the fish *Pseudosciaena crocea*, 1996 genes were estimated to be up- or down-regulated after a bacterial challenge, for a total of 8216 unigenes found in the transcriptome, which represents 24.3% differentially expressed transcripts [39] as compared to 31% in the present study where three challenges have been compared.

**Figure 5:**
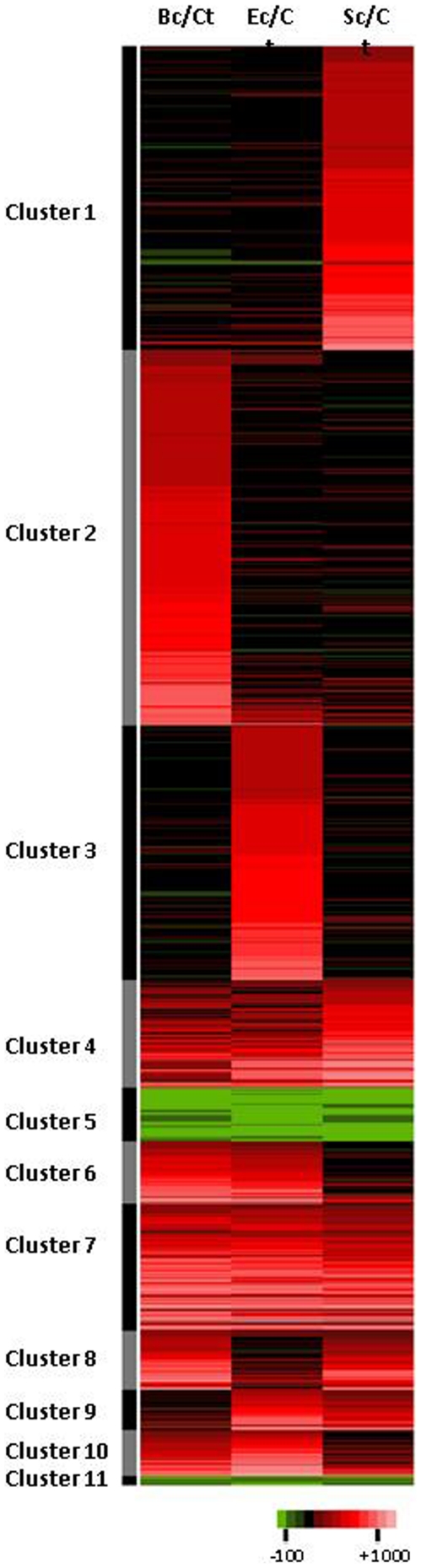
Relative expression of candidate transcripts in the three libraries of snails challenged by *B. cereus* (Bc), *E. coli* (Ec) or *S. cerevisiae* (Sc) as compared with their expression in the library of control snails (Ct). Note that transcript clusters resulting from the HAC analysis show clear differences in expression profiles. Color code for expression is shown in fold-change as compared with control.

To describe the expression profiles in the four libraries and to identify groups of unisequences sharing similar expression profiles, we used a combination of factorial correspondence analysis (FCA) [40], [41] and hierarchical ascending clustering analysis (HAC) [42], resulting in a dendrogram (not shown) computing all Euclidian distances between unisequences. The graphical representation of the expression levels of candidate unisequences, as clustered by the HAC, clearly shows 11 major clusters of transcripts sharing a common expression pattern (fig. 5). Interestingly, transcripts from cluster 1 are characterized by higher expression after challenge with *S. cerevisiae* while transcripts from clusters 2 and 3 are particularly expressed after a challenge by *B. cereus*, and *E. coli* respectively (fig. 5). Therefore, transcripts from clusters 1, 2 and 3 may be involved in a response that is specific for the immune challenge. In contrast, clusters 4 to 11 show more complex expression profiles with up- or down-expressions in two or all of the challenged groups as compared with control. For example, transcripts from the cluster 5 are under-expressed after the three immune challenges, and transcripts from the cluster 8 are over-expressed after challenge with *S. cerevisiae* and *B. cereus* (fig. 5). Clusters 4 to 11 may be interpreted as clusters of transcripts potentially involved in a less or non-specific response to immune challenges.

**Figure 6:**
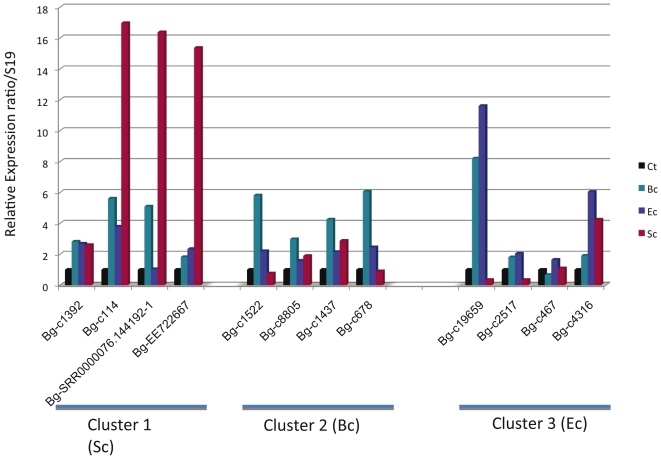
Expression of candidate transcripts from clusters 1, 2 and 3 using quantitative PCR. For each candidate, the expression is shown in control snails (Ct), or snails exposed to *B. cereus* (Bc), *E. coli* (Ec) or *S. cerevisiae* (Sc). These are 12 representative examples out of 36 analyzed candidates.

In order to further examine the reliability of this global approach, we performed qPCR expression analysis on 36 candidates randomly selected among annotated or un-annotated unisequences from clusters 1, 2, and 3. The expected expression patterns (higher expression in *S. cerevisiae*, *B. cereus* or *E. coli* challenged snails respectively) were observed for 28 transcripts (fig. 6) suggesting that approximately 80% of the candidates are correctly assigned to an expression cluster.

**Figure 7:**
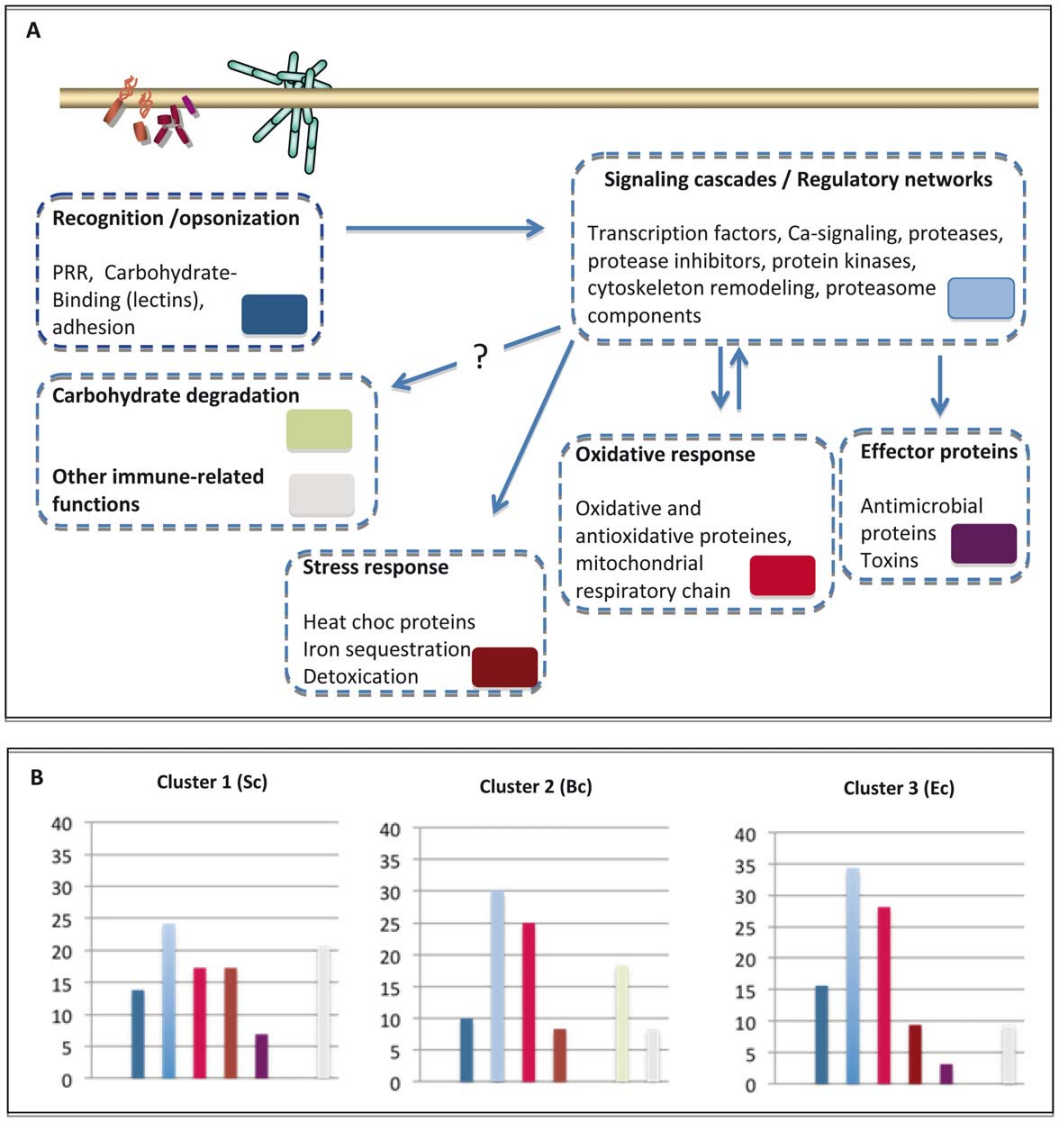
**A**) **Schematic representation of the relation between the major immune-relevant processes or protein category.** For each process, a list of typical proteins and a color code are shown. **B**) **Distribution of the number of unisequences per process/category in the clusters 1, 2 and 3.** Colour codes are as in (A). The complete list of candidate unisequences from each cluster (code numbers as deposited in http://www.snaildb.org/) is provided in the file text S1.

To gain insights into the functional relevance of these candidate transcripts, the selected 1685 sequences were analyzed further through a combination of manual searches for sequence similarities using all available databases (SwissProt, InterPro, NRdb, NCBI-dbEST). Almost a thousand transcripts remained un-annotated (Table 3), representing therefore a substantial dataset of unknown transcripts showing apparent differential expression after immune challenges. These transcripts are available for future expression or functional studies since the complete list of candidate unisequences from each cluster, with code numbers as deposited in the *Biomphalaria glabrata* database [32] is provided in the supporting Text S2. Regarding annotated transcripts, predicted functions could be assigned to several broad immune-relevant functional groups that are represented in the fig. 7A. Figure S2 lists the predicted function of possible immune-relevant unisequences found in the clusters of interest. Some of these candidates belong to families previously shown to be up-regulated after an immune challenge [26], therefore supporting previous observations. In particular, transcripts encoding PGRP, C1q, LPS binding proteins, serpins, FREPs and SODs were shown to be up-regulated 12 h after challenges using an oligo- array study [26]. Note that a vast majority of candidate transcripts do not align with *B. glabrata* sequences referenced in GenBank, showing that this transcriptomic study significantly improves our identification of immune-relevant candidates from *B. glabrata*.

**Table 3:** Number of candidate unisequences (total and annotated) in each expression cluster.

Cluster	Total number unisequences	Number Annotatedunisequences
1	356	133
2	440	191
3	298	103
4	126	49
5	63	19
6	73	32
7	149	76
8	69	34
9	47	21
10	54	12
11	10	5
total	1,685	694

It is not possible to compare quantitatively the composition of all clusters as they greatly differ in size (sequence number) and most of them only contain few transcripts with predicted functions. However, the first three clusters presenting expression profiles that are specific for the challenges contained enough annotated transcripts to be compared. As shown in fig. 7B, these three clusters do not greatly differ in the distribution of predicted functional categories, except for the carbohydrate degradation category that is only observed in cluster 2 (fig. 7B). Note that these distributions do not reflect percentage of reads scored in each functional group, but percentage of unisequences. The figure therefore provides an indication of the transcript diversity within each functional group. A common feature of these clusters is the low diversity of transcripts corresponding to immune-effector proteins, and the high diversity of transcripts possibly involved in signaling pathways or regulatory networks as well as oxidative or anti-oxidative processes (fig. 7A, B). This may be explained by the short time-point of 6 h post-challenge analyzed in this study, which corresponds to an early immune response. A small proportion of transcripts predicted to encode effector proteins has been reported in transcriptomic studies on other mollusk species [43] as well as in previous studies on *B. glabrata*, investigating longer time-points after challenge [18], [20], [38].

### Transcripts involved in pattern-recognition, carbohydrate binding or adhesion

Pattern recognition receptors (PRR) are involved in the first step of invertebrate immune response as they bind to highly conserved pathogen structures such as peptidoglycans or lypopolysaccaride (LPS) from bacteria or β-glucans from fungi [44]. In this study we identified differentially expressed transcripts corresponding to novel peptidoglycan recognition proteins (PGRP), Gram-negative bacteria binding protein (GNBP), putative thioester-containing protein (TEP), C1q domain-containing protein C-type lectins and galectins (figure S2). PGRPs and GNBPs are important PRRs known to play an essential role in the upstream activation of the Toll and Imd pathways [4]. Although PGRPs are expected to present a higher binding affinity to peptidoglycans (PGN), while GNBPs are expected to bind preferentially to LPS and fungal β-1-3-glucans, members of both PGRP and GNBP families show functional diversities [45], [46]. For example, members of the *Anopheles* GNBP family are involved in the defense against a broad range of pathogens, including Gram-positive bacteria and protozoa such as *Plasmodium* [46]. The presence of 3 PGRP unisequences in the cluster 3 (responsive to *E. coli* challenge) is not surprising as the cell wall of Gram-negative bacteria also contains PGN. Three transcripts from a long form (*BgPGRP-LA*) and one transcript from a short form PGRP gene (*BgPGRP-SA*) had previously been identified [25]. The three PGRP unisequences from cluster 3 significantly align with the short form Bg-PGRP-SA [25] as well as with the N-terminal part of the long form Bg-PGRP while showing substantial sequence differences (approx. 56–63% identity – alignment not shown). Similarly, GNBP unisequences from the clusters 1 and 2, both align with the previously described Bg-GNBP [25] but present substantial sequence differences (52% and 55% identity respectively). These results show that additional PGRPs and GNBPs are present in *B. glabrata* and deserve further investigation.

C1q domain containing (C1qDC) proteins consist of an optional leading signal peptide, a central collagen-like region of variable length, acting as oligomerization domain (sometimes missing), and a C-terminal C1q domain [47], [48]. Depending on the presence or the absence of the collagen-like region, C1qDC proteins are classified as C1q-like proteins or ghC1q (globular head C1q) proteins respectively [49]. Some C1qDC proteins with specific ligand recognition properties have been described and characterized in mollusks like the snail *Cepaea hortensis* [50], the scallop *Chlamys farreri* [51], *Mytilus edulis* [52], [53] and *Pinctada fucata* [54]. The role of C1qDC proteins in specific pathogen recognition has been investigated in mollusks. C1qDC transcripts increase rapidly and strongly in response to the injection of Gram-positive and Gram-negative bacteria in *Mytilus galoprovincialis* [55] and up-regulation of C1qDC proteins has been linked to infections with bacterial and metazoan parasites in mollusks such as *Ruditapes decussatus* [56], *Crassostrea gigas* [57], *Mercenaria mercenaria* [58], and, recently, *Biomphalaria glabrata* [26]. Thus it is not surprising to see C1qDC protein present in cluster 3 (responsive to *E. coli* challenge). Despite the role of molluskan C1qDC proteins in pathogen recognition, the implication of C1qDC proteins in the immune response of this phylum remains to be clarified.

Thioester-containing proteins (TEP) are a family of proteins characterized by a canonical intra chain thioester bond (GCGEQ) that is also shared by the complement factor C3, a major component of immunity in vertebrates [59]. TEP proteins play important roles in innate immune responses by acting as opsonins that promote phagocytosis of invading cells [60]. In *Drosophila*, dmTEP2 and dmTEP3 are required for efficient phagocytosis of *E. coli* and *S. aureus* respectively. The macroglobulin complement related protein also called dmTEP6, plays a crucial role in the recognition and elimination of pathogenic yeasts [61]. In anopheles, AgTEP1 binds to Gram-negative or Gram-positive bacterial surfaces through a thioester bond to promote their phagocytosis [62]. In *B. glabrata*, a recent study identified BgTEP1 as a protein interacting directly or indirectly via the FREPs proteins with mucins from the parasite *Schistosoma mansoni* [22]. According to the results from the present study, expression of BgTEP1 is highly increased after *E. coli* challenge. In addition to BgTEP1, the present study identified a novel TEP highly expressed after a *B. cereus* challenge. Its predicted amino acid sequence, although partial, shares about 37% identity and 56% similarity with BgTEP1. These results clearly indicate that both BgTEPs are expressed in a challenge-specific manner. Further analysis of lophotrochozoan TEPs will help to elucidate their functions as well as their specificity.

### Transcripts involved in regulatory networks and signaling pathways

Transcripts potentially involved in regulatory networks or signaling pathways include numerous serine proteases and proteases inhibitors possibly acting in the enzymatic cascades regulating the activation of signaling pathways such as the Toll pathway [63], calcium-binding proteins or proteins involved in calcium signaling, transcription factors, or kinases such as MAP kinases that are key elements of the immune-relevant MAPK pathways.

Zinc finger proteins are generally involved in regulatory networks [64] although their structural features are not sufficient to provide function predictions.

**Figure 8:**
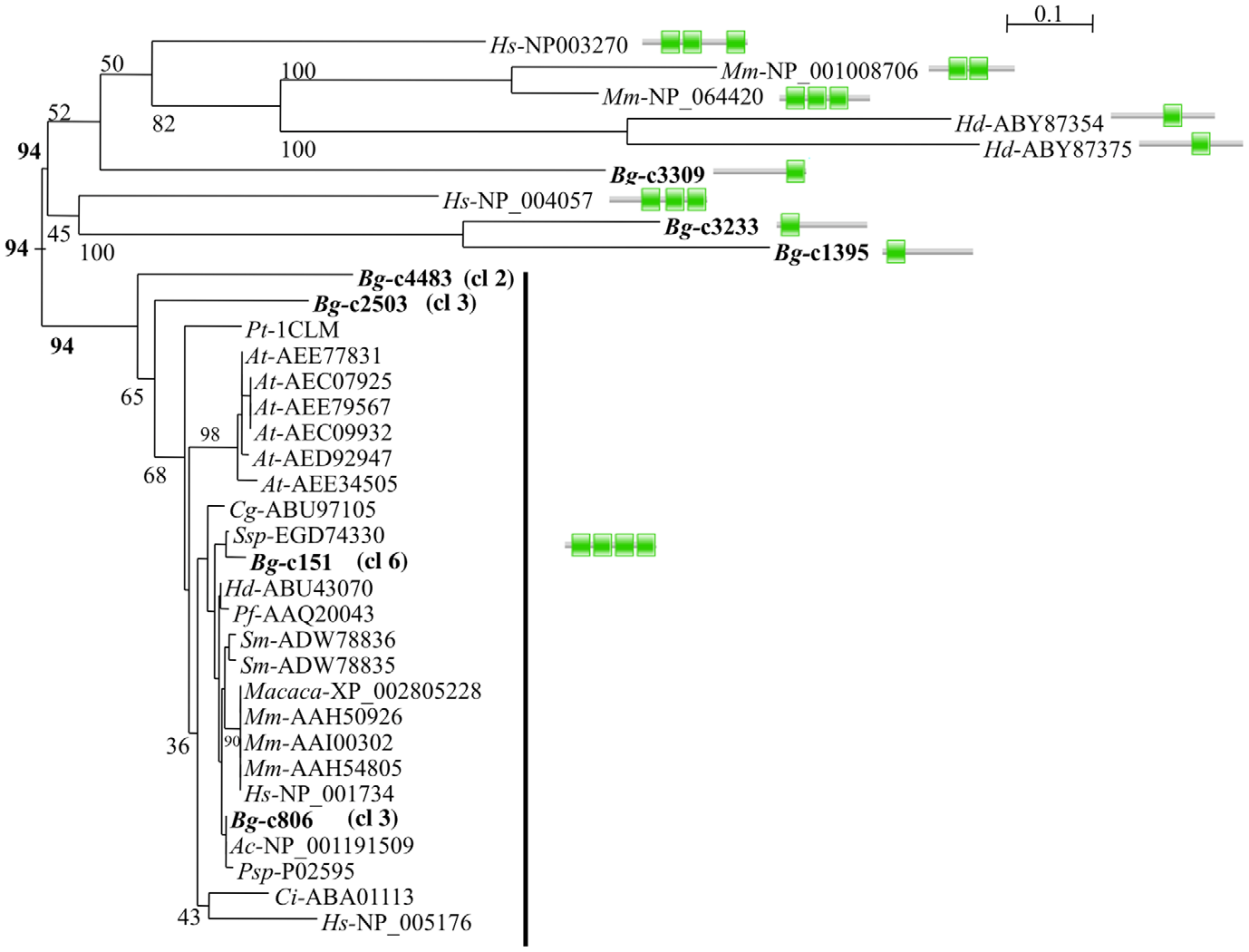
Phylogenetic relationships of calmodulins. Neighbour joining reconstruction of *B. glabrata* unisequences showing significant similarities with calmodulins (complete predicted proteins only, shown in bold) and sequences from *Homo sapiens* (*Hs*), *Mus musculus* (*Mm*), *Macaca mulatta* (*Macaca*), *Arabidopsis thaliana* (*At*), *Crassostrea gigas* (*Cg*), *Chlamydomonas incerta* (*Ci*), *Paramecium tetraurelia* (*Pt*), *Halichondria okadai* (*Ho*), *Schistosoma mansoni* (Sm), *Aplysia californica* (Ac), *Pinctada fucata* (*Pf*), *Salpingoeca sp. ATCC 50818* (*Ssp*), *Patinopecten sp*. (*Psp*). Genbank accession numbers of the sequences used for the reconstruction are shown next to the species identification. *B. glabrata* sequences belonging to candidate differentially expressed clusters are indicated by the cluster number (cl). Values at nodes are Bootstrap proportions. Bar: 0,1 substitution/site. Significant EF-hand domains (Pfam reference: accession no. PF00036; e-value<1.10-6) identified in each sequence are represented by green squares.

In addition to the expected transcripts such as transcription factors or kinases, this *de novo* sequencing study yielded a number of candidates deserving further investigations. For example, an intriguing observation is that calmodulins are present in all three challenge-specific clusters and in the less specific clusters (figure S2). Calmodulins (CaM) are ubiquitous calcium-sensing proteins, characterized by the presence of EF-hand calcium-binding domains. In vertebrates, the Ca2+//CaM complex is known to control the activation status of more than 50 target proteins, including major enzymes of the immune response such as Ca2+/CaM dependent kinases (CaMK and CaMKK) or the inducible nitric oxide synthase [65], [66], [67]. In order to further explore the relationships between *B. glabrata* predicted calmodulins, we performed a phylogenetic reconstruction, including all *B. glabrata* calmodulin-like sequences (predicted complete calmodulins from the 5308 mapped unisequences, including less- or not differentially expressed candidates), as well as calmodulin sequences from various animal phyla and plants. Calmodulins are known to be highly conserved proteins, differing by only few amino acids between vertebrates and invertebrates [68]. As shown in fig. 8, the four calmodulins identified in the differentially expressed candidates clearly cluster with highly conserved animal and plant calmodulins. Note that other calmodulin-like candidates, as well as calmodulins previously identified from another gastropod species, *Haliotis diversicolor* [69] belong to a poorly resolved group (fig. 8). When examining further the sequences, it appeared that the highly conserved calmodulins are characterized by the presence of four EF-hand domains (Pfam reference: accession no. PF00036, e-value<1.10-6) (alignment shown in the figure S3), whereas other calmodulin-like sequences present 1 to 3 significant EF-hand domains (fig. 8). Multiple calmodulin copies have been found in a variety of taxa including mollusks [68] and our results provide evidence that at least four calmodulins exist in *B. glabrata*. Their apparent differential expression after immune challenges suggest that they may be involved in different immune responses.

Another unexpected candidate is a thymosin β4 present in cluster 8 and characterized by transcripts with a higher expression after *E. coli* and *S. cerevisiae* challenges (see fig. 8). The predicted translation of the Bg-c1591 unisequence (not shown) corresponds to a 41 amino-acid peptide with a thymosin superfamily domain (Pfam reference: accession no. PF01290). In vertebrates, thymosins are involved in the regulation of numerous processes through their ability to bind actin and to promote or inhibit actin assembly [70]. For example they play a neurotrophic and antiapoptotic role during the development of the nervous system in vertebrates, and also promote wound healing and participate in the anti-inflammatory response [70]. To our knowledge, their function has not been studied in invertebrate species, but a recent study on another gastropod snail (the abalone *H. diversicolor supertexta*) showed that a thymosin β4 was well expressed in hemocytes and increased its expression after LPS challenge [71]. The likely role of thymosins in mollusk immunity clearly deserves further investigations.

Finally, among other candidates, one may notice a tyrosinase and a sialic acid acetylesterase. The role of tyrosinases in invertebrate immunity is well established in arthropod species since tyrosinases such as polyphenol oxidases (PO) are key enzymes of the melanogenic immune response [72]. However, melanisation does not occur during mollusc immune response, and although tyrosinases have been previously identified from mollusk species [73], their involvement in the immune response of gastropods is not clearly established. Similarly, the sialic acid acetylesterase pathway is known for controlling the inhibitory signaling of B cell receptors from mammals [74], but its possible involvement in invertebrate immunity has not been documented so far.

### Transcripts involved in oxidative response or anti-oxidative response

Production of Reactive Oxygen Species (ROS) is a common effector mechanism of the immune systems of vertebrate and invertebrate species [75], [76]. Previous studies on *B. glabrata* showed, for example, that production of hydrogen peroxide (H2O2) was important for killing trematodes such as *S. mansoni* [77], [78] and provided evidences for the existence of a correlation between production of ROS and efficient killing of the parasite [79]. However, because production of ROS also has negative effects on the host cells themselves through lipid peroxidation and DNA damage leading to loss of cellular function and ultimately apoptosis and necrosis [80], it is tightly controlled by ROS-detoxifying enzymes. Unsurprisingly, immune challenge of *B. glabrata* by microorganisms resulted in an increased expression of proteins involved in the production or in the detoxification of ROS, as shown from previous studies [11], [18], [20], [38]. Cytochrome c oxidases and NADH dehydrogenase (known to produce superoxide and hydrogen peroxide) are involved in ROS production, whereas glutathione peroxidases, thioredoxins, glutaredoxins, oxidoreductases, methyltransferases, mono-oxygenases or cytochrome C reductases participate in their detoxification. Two dozens novel candidates potentially participating to the oxidative (and anti-oxidative) response have been identified in this study (figure S2).

### Transcripts involved in stress response, detoxification or chaperone transcripts

Peptidylprolyl isomerases or cyclophilin, are chaperone enzymes catalyzing the cis-trans isomerization of prolines. Functions in intracellular signaling and intercellular communication [81] have been described for this diversified family of proteins. The three cyclophilin-like unisequences reported here appear differentially expressed in response to one of the microbial challenge.

**Figure 9:**
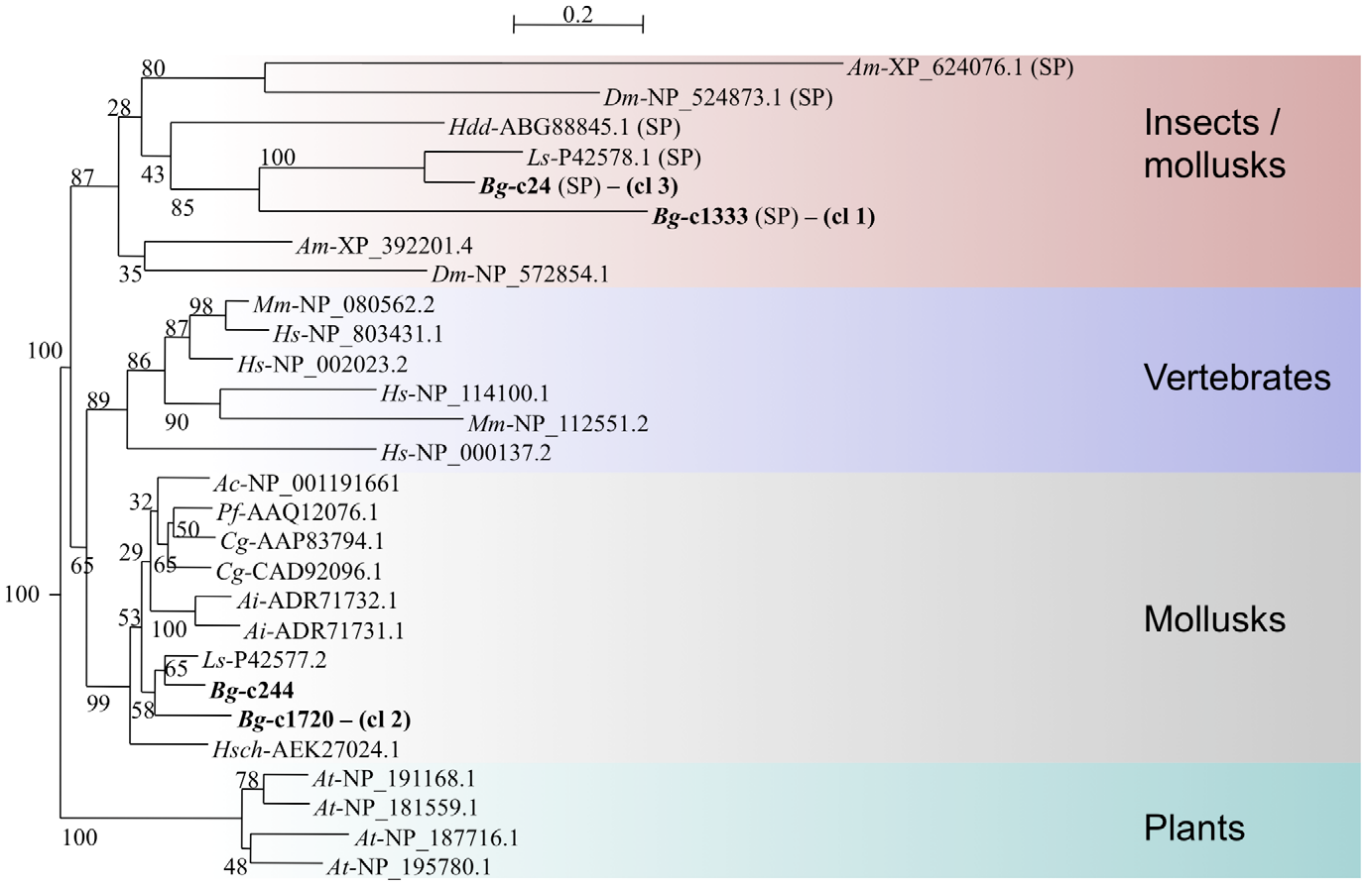
Phylogenetic relationships of ferritins. Neighbour joining reconstruction of *B. glabrata* unisequences showing significant similarities with ferritins (complete predicted proteins only, shown in bold) and sequences from *Homo sapiens* (*Hs*), *Mus musculus* (*Mm*), *Arabidopsis thaliana* (*At*), *Drosophila melanogaster* (*Dm*), Apis mellifera (*Am*), *Aplysia californica* (Ac), *Pinctada fucata* (*Pf*), *Crassostrea gigas* (Cg), *Lymnaea stagnalis* (*Ls*), *Haliotis discus discus* (*Hdd*), *Argopecten irradians* (*Ai*), *Hyriopsis schlegelii* (*Hsch*). Genbank accession numbers of the sequences used for the reconstruction are shown next to the species identification. *B. glabrata* sequences belonging to candidate differentially expressed clusters are indicated by the cluster number (cl). Presence of a signal peptide is indicated by (SP). Values at nodes are Bootstrap proportions. Bar: 0,2 substitution/site.

Ferritins are major iron-binding proteins involved in the regulation of iron distribution and in the detoxification of toxic free iron. Ferritins also participate in the iron sequestration strategy depriving infectious microorganisms from iron acquisition and inhibiting their multiplication [82]. An involvement of ferritins in the response to pathogens has been reported in many species and was functionally demonstrated in *Ceanorhabditis elegans* [83]. In *B. glabrata*, ferritin ESTs were previously reported among immune-relevant candidate [10], [18], [84] but no predicted ferritin proteins have been deposited in GenBank. To explore the diversity and relationships of *B. glabrata* ferritins, we searched for all predicted ferritin unisequences in the database of 5308 mapped unisequences and analyzed the predicted complete sequences. The phylogenetic reconstruction (fig. 9) shows that predicted ferritins from clusters 1 and 3 are substantially different but align with secreted ferritins from various mollusks and insect species. Confirmation of the presence of a signal peptide has been obtained for most proteins of this group (fig. 9). Two other ferritins present a higher sequence identity (see figure S4) and cluster together with mollusks proteins predicted to be soma-ferritins, some of which were reported to be differentially expressed after a bacterial challenge [85]. Our results further support the involvement of ferritins in mollusk immune responses and provide evidences for the existence of at least four ferritins in *B. glabrata*: 2 highly conserved soma-ferritins and two less conserved secreted ferritins. Further studies will investigate their potential role in challenge-specific immune responses.

### Effector proteins

As previously mentioned, very few effector protein transcripts, including putative antimicrobial proteins, were identified using this sequencing study and they encode an escaping/achacin/aplysianin precursor and two Lipopolysaccharide-binding protein (LBP)/bactericidal/permeability-increasing protein (BPI). Transcripts of both types of proteins have previously been reported in *B. glabrata* [11] but their diversity and biological activities remain to be characterized. Results from this large-scale study confirm previous observations from gene discovery studies where very few anti-microbial proteins and no antimicrobial peptides have been reported [11], [18], [26].

### Transcripts involved in carbohydrate degradation

Cluster 2 is characterized by the presence of enzymes involved in carbohydrate degradation.

Cellulases (including beta-1,4-glucanases) and chitinases are well-known enzymes involved in the degradation of the two most abundant polysaccharides in nature, which are cellulose (major structural component of plants) and chitin (major component of fungal cell wall and of arthropod exoskeleton). In eukaryotic animal species, these enzymes are generally involved in digestion and it is possible that *B. glabrata* cellulase and chitinase transcripts identified in our study also encode digestive enzymes. This possibility is however not supported by the fact that all transcripts are restricted to cluster 2 (more abundant after a challenge by *B. cereus*). Alternatively, a possible role in immune response has been reported for a chitinase-like transcript from the oyster *Crassostrea gigas*, showing an increased expression in hemocytes after a bacterial challenge [86], [87]. Further studies are needed to investigate whether *B. glabrata* chitinases and cellulases are restricted to the digestive function and how to explain their expression after a challenge by a Gram-positive bacterium.

### Transcripts of putative repetitive proteins

Several transcripts with repetitive sequences have been observed in cluster 1. Their predicted translations align with repetitive proteins such as ankirin repeat proteins, antifreeze proteins, or extensins (E-value of <1. 10-10). Exploration of the original EST database of redundant sequences confirmed that these repeats are found in individual ESTs and are not an artifact from the automatic assembly of ESTs. Additional work is required to characterize their full-length cDNAs and determine whether or not these transcripts represent novel highly regulated immune-relevant repetitive proteins.

### Conclusion

This work provides the first massive sequencing-based study of *Biomphalaria glabrata* transcriptomes. The transcriptomic database that we have developed, mainly from public 454 ESTs, represents a significant input in *B. glabrata* transcriptomics and allowed to compare the four 5′-end cDNA libraries described here.

In addition to providing a large number of novel immune-relevant candidate transcripts with expected differential expression profiles, this study yielded two original results. First, comparison of expression profiles indicated that a higher proportion of transcripts were up-regulated in a challenge-specific manner, as compared with transcripts regulated in a lower or non-specific manner after exposure to Gram-negative or Gram-positive bacteria and to yeast.

Second, when focusing on the predicted functions of annotated candidate transcripts, it appeared that transcripts belonging to different expression clusters did not greatly differ in the functional processes or even protein family they relate to. On the contrary, with the exception of a few transcripts (i.e. carbohydrate degradation related transcripts characterized among *B. cereus* responsive transcripts), most of them correspond to proteins predicted to be involved in similar processes. Intriguingly, results showed that transcripts of some protein families such as GNBP, TEPs, calmodulins, ferritins, or cyclophilins, are expressed after any immune challenges, but that a different family member is expressed after each challenge.

Altogether these results suggest that the response to various pathogens may involve, to a large extent, similar immune processes or signaling pathways but that different copies of genes belonging to multigenic families may participate to each particular response.

Efforts will now be made to characterize immune-relevant multigenic families and to analyze the functions of their members with a particular focus on a putative involvement in the pathogen-specific immune response.

## Materials and Methods

### Immune challenges of *Biomphalaria glabrata*

*Biomphalaria glabrata* snails were maintained in the laboratory according to standard procedures [88]. For immune challenges, groups of six adult snails from 9 to 11 mm in shell diameter were transferred to a 100 ml beaker and exposed to a suspension of *Escherichia coli* (ATCC 8739), *Bacillus cereus* (ATCC 10987) or *Saccharomyces cerevisiae* ajusted to 10^6^ cells/ml in pond water or exposed to sterile pond water (control snails). After one hour exposure, snails were rinsed in clean pond water, transferred to 500 ml water tanks and maintained under normal conditions until freezing in liquid nitrogen at 6 hr post-challenge. Experiments were independently repeated three times. This procedure has been selected after a series of 4 independent pilot experiments using candidate transcripts such as aplysianin, theromacyns, cystatins or dermatopontins previously identified as being differentially expressed after immune challenges, and belonging to different functional categories [11], [16]. Time points from 2 hr to 72 hr post-exposure (PE) were analyzed and the time-point of 6 hr post-exposure was selected as the time-point showing the highest number of over-expressed transcripts (results not shown).

### Preparation and sequencing of non-normalized 5′-end cDNA libraries

Total RNA was extracted from individual snails using Trizol Reagent (Invitrogen) according to the manufacturer's instructions. Pools of total RNA made of 2 µg RNA from each individual snail were used for preparation of cDNA libraries. Therefore, each of the four RNA pool was prepared from an equal amount of RNA from a total of 18 individuals from three experiments (6 individuals/experiment). This procedure aimed at obtaining samples representative of each treatment. For each sample (RNA pool), 20 µg total RNA was DNase treated (Turbo DNase kit) and sent to GATC for quality check, library preparation using an “oligo-capping” method [27] and deep sequencing according to Illumina/Solexa procedures. Briefly, after testing total RNA integrity, poly(A)-RNAs were purified and treated with calf intestine phosphatase (CIP) in order to hydrolyze the 5′Phosphate of truncated mRNAs. Tobacco acid pyrophosphatase (TAP) was then used to remove the cap structure of intact mRNAs, and an oligo-RNA adapter was ligated to the 5′ -phosphate of decapped mRNAs. First-strand cDNA synthesis was then performed using a N6 randomized adapter primer and M-MLV-RNase H- reverse transcriptase. The resulting cDNAs were amplified with 21 cycles of PCR. Amplicons in the size range of 350–650 bp were purified and processed for deep sequencing on Illumina Genome Analyser II (Illumina GAII) according to Illumina procedures. 36 bp long sequences were produced and referred to as “reads” in this manuscript.

### Cleaning of Illumina 5′-end cDNA reads

Reads were automatically trimmed and validated by screening for low quality (short sequences or presence of ambiguous nucleotides), low complexity or contaminant sequences (*S. cerevisiae*: genbank Acc. Number from NC_001133 to NC_001148 and NC_001224; *B. cereus* genome accession number NC_003909.8; *E. coli* genome accession number NC_010468.1) using the SeqClean tool from the Gene Index Project [89]. These erroneous reads (Table 2) were removed from the study and the remaining reads were mapped against the reference database.

### Preparation of a *Biomphalaria glabrata* transcriptomic reference database

To link the 5′-end cDNA reads to expressed genes, we prepared a reference gene dataset combining all *B. glabrata* transcript sequences available in public databases at the time of the study: the 54,309 ESTs present in NCBI dbEST, the 179 mRNA sequences as well as 704,022 sequences from 454 sequencing by the Genome Sequencing Center (GSC) at Washington University (*Biomphalaria glabrata* genome project ID: 12879). These 454 sequences were available at NCBI Sequence Read Archive (accession numbers SRX001380, SRX001379 and SRX000011).

All sequences were automatically screened for vector contaminant, low quality and low complexity sequences using the SeqClean tool. Known ribosomic RNA sequences were also used to screen the database and matching ESTs were removed from the analysis. Remaining trimmed sequences superior to 100 bp in length were then automatically assembled and clustered using a modified version of TGICL [90] named TGICL++. Briefly, the TGICL++ package was optimized to accommodate very large datasets. Using nrcl and tclust, tools available in the TGICLpackage, the TGICL++ pipeline performed successive clustering steps being very strict at first then increasingly permissive. The starting parameters were a match identity of 97% with an overlap of at least 100 bases. Parameters for final assembly were an overlap length cutoff superior to 40 bp and an overlap percent identity cutoff superior to 97%.

The unique sequences (unisequences) resulting from the assembly work were automatically annotated using two independent annotation tools. The automatic translation and peptide detection using the FrameDP tool [91] was followed by a search for InterPro domains in the detected peptides [30]. The second annotation consisted in a BLASTx search [31] against SwissProt protein database setting the maximum e-value at 10-3.

### Bioinformatic and statistics

Sequences were processed by a custom analysis workflow procedure mainly based on perl scripts developed in the BIOS project [92] and supplemented by custom perl scripts. Reads from the four non-normalized libraries were mapped to the EST unisequences from the reference database using SAOPaligner/soap2 [35]. A maximum of three mismatches was allowed and the selected match mode was the best hits. Reads mapping several unisequences with an equivalent score were conserved in the analysis. For each unisequence, reads originating from each of the four libraries were automatically scored.

The contingency table containing the number of reads for each unisequence in each library (control, *E. coli*-, *B. cereus*- or *S. cerevisiae*-exposed snails) was first analyzed by a factorial correspondence analysis (FCA) [40], [41] using the PROC CORRESP procedure in the SAS/STAT package [42]. This method is used to find a low-dimensional graphical representation of the association between rows (here read counts for each unisequences) and columns (here libraries) in a Euclidian space, the first axes being, by definition, those that explain most of the information available in the data. Two unisequences sharing close FCA coordinates have similar expression patterns. Then, unisequence's coordinates on the first three axes of the FCA were used to compute an Euclidian distance between all of them. These distances were graphically described by a dendrogram (hierarchical ascending clustering with an UPGMA method) by PROC CLUSTER (option: AVERAGE) of the SAS/STAT package [42].

### Quantitative PCR analysis

cDNAs were generated from 1 µg total RNA of control and challenged snails, according to standard procedures of the iScript cDNA synthesis kit (Biorad, California, USA).

Primers were generated using Primer3 (http://frodo.wi.mit.edu/primer3/). Real-time quantitative PCR was carried out on a DNA Engine 2 (MJ Research, Minnesota, USA) with qPCR MasterMix Plus for SYBR green I (Eurogentec, Seraing, Belgium) using one internal reference gene (ribosomal protein S19, GenBank accession number CK988928). The following protocol was used: denaturation (95°C for 10 min), amplification and quantification repeated 40 times (95°C for 30 s, 60°C for 30 s, 68°C for 30 s), melting curve program (65–95°C with a heating rate of 0.1°C/s and continuous fluorescence measurement). Signal intensity was measured at the end of each elongation phase and results were analyzed using the Opticon 3.1 software provided by MJ Research. Relative abundance was calculated by the comparative Ct method (Applied biosystems, Foster City, USA). Following each qRT-PCR reaction, dissociation curves were examined for validation of amplicon purity.

## Supporting Information

Figure S1 **Size distribution of unisequences from the reference transcriptomic database (n = 102,175).** Sequence number (y axis) is shown on a logarithmic scale.
(TIF)

Figure S2 **List of selected candidate unisequences from cluster 1, 2, 3 or from all other clusters.** Cluster 1, 2, and 3 include transcripts that are highly expressed after *S. cerevisiae* (Sc), *B. cereus* (Bc), or *E. coli* (Ec) challenge respectively, in a challenge-specific manner. Other clusters include transcripts that are up- or down-regulated after two or all of the challenges. The unisequence accession numbers are either as shown in the file text S1 and deposited in http://www.snaildb.org/ (starting by Bg-c) when novel or correspond to GenBank accession numbers when already deposited in GenBank.
(RTF)

Figure S3 **Alignment of the complete sequences of predicted calmodulins from** ***B.glabrata*** **.** *Biomphalaria glabrata* sequences (Bg) showing the typical combination of 4 EF-Hand domains (Pfam reference: accession no. PF00036) of calmodulins have been aligned (MUSCLE software; [93] to sequences from *Homo sapiens* (Hs) *Mus musculus* (*Mm*), *Macaca mulatta* (*Macaca*), *Arabidopsis thaliana* (*At*), *Crassostrea gigas* (*Cg*), *Chlamydomonas incerta* (*Ci*), *Schistosoma mansoni* (Sm), *Aplysia californica* (Ac), *Pinctada fucata* (*Pf*), *Salpingoeca sp. ATCC 50818* (*Ssp*), *Patinopecten sp*. (*Psp*). The four significant EF-hand domains (e-value<1.10-6) are positioned on the alignment.
(DOC)

Figure S4 **Alignment of the complete sequences of predicted ferritins from** ***B.glabrata*** **.** Alignment of two highly conserved ferritins predicted to be soma-ferritins (A) and two predicted secreted ferritins (B).
(TIF)

Text S1 **Consensus sequences of contigs (43,238) with automatic annotation.** Sequences are presented in a FASTA format. The sequence accession number is followed by the sequence length (len), the number of ESTs aligning in the contig (count), the presence of an InterPro domain (IPR) and the five best hits with the SwissProt database (blastx-SP).
(RTF)

Text S2 **List of the candidate unisequences belonging to the expression clusters (codes as deposited at http://www.snaildb.org/).**
(RTF)
