# Supplementary figures and images for: Specific versus Non-Specific Immune Responses in an Invertebrate Species Evidenced by a Comparative de novo Sequencing Study
Source: PLoS One. 2012 Mar 12;7(3):e32512. doi: 10.1371/journal.pone.0032512 (PMC3299671; doi:10.1371/journal.pone.0032512)

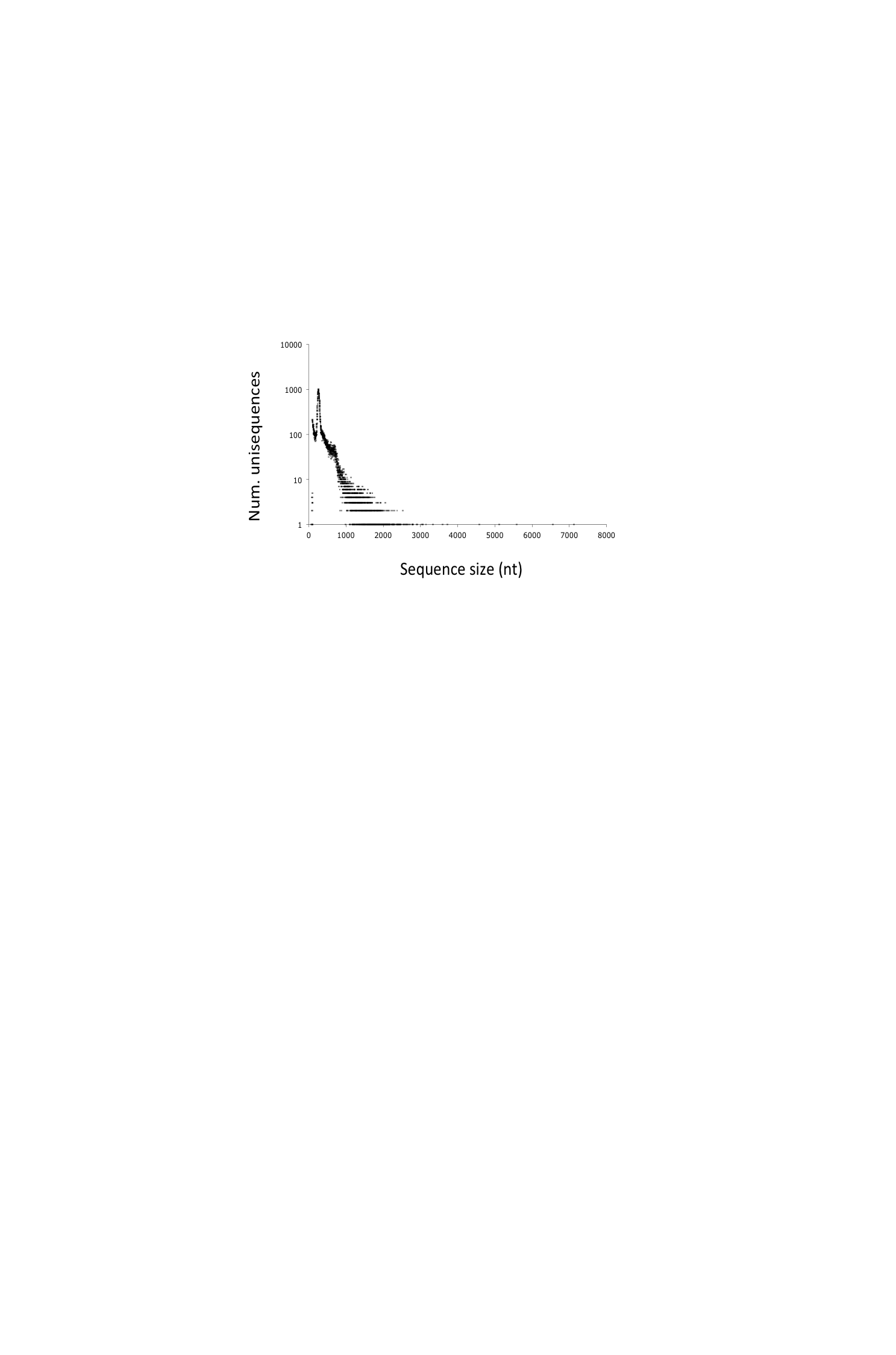

Supplement: Figure S1 — Size distribution of unisequences from the reference transcriptomic database (n = 102,175). Sequence number (y axis) is shown on a logarithmic scale. (TIF) [file pone.0032512.s001.tif]

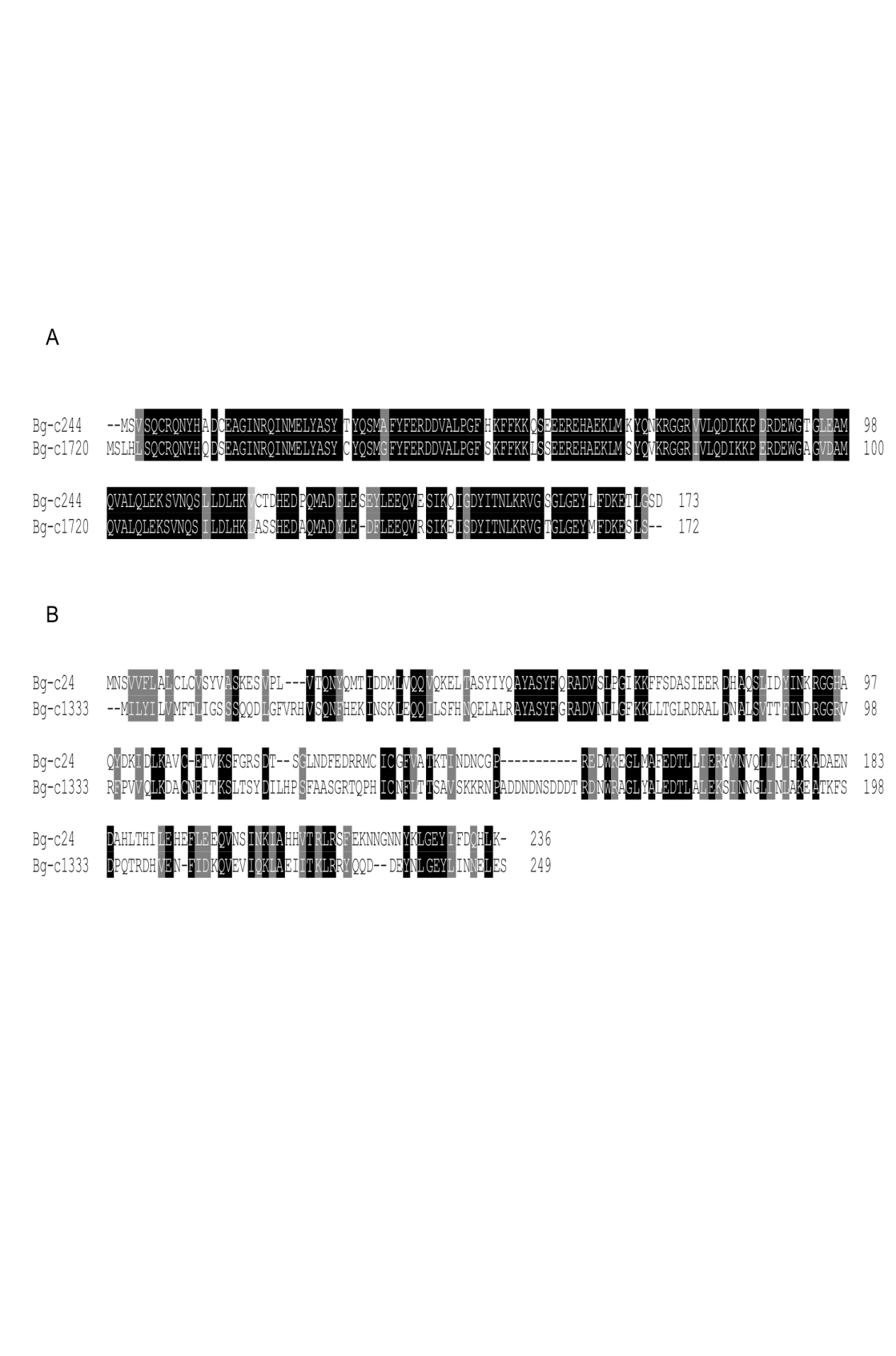

Supplement: Figure S4 — Alignment of the complete sequences of predicted ferritins from B.glabrata . Alignment of two highly conserved ferritins predicted to be soma-ferritins (A) and two predicted secreted ferritins (B). (TIF) [file pone.0032512.s004.tif]
