# Supplementary material for: Specific versus Non-Specific Immune Responses in an Invertebrate Species Evidenced by a Comparative de novo Sequencing Study
Source: PLoS One. 2012 Mar 12;7(3):e32512. doi: 10.1371/journal.pone.0032512 (PMC3299671; doi:10.1371/journal.pone.0032512)
Supplement: Figure S2 — List of selected candidate unisequences from cluster 1, 2, 3 or from all other clusters. Cluster 1, 2, and 3 include transcripts that are highly expressed after S. cerevisiae (Sc), B. cereus (Bc), or E. coli (Ec) challenge respectively, in a challenge-specific manner. Other clusters include transcripts that are up- or down-regulated after two or all of the challenges. The unisequence accession numbers are either as shown in the file text S1 and deposited in http://www.snaildb.org/ (starting by Bg-c) when novel or correspond to GenBank accession numbers when already deposited in GenBank. (RTF) [file pone.0032512.s002.rtf]

					
					
	Cluster 1 (Sc)	Cluster 2 (Bc)	Cluster 3 (Ec)	Other clusters	
Pattern-recognition, carbohydrate binding, adhesion	fibrinogen-related protein-FREP 14 - Bg-c16341; Gram-negative bacteria binding protein -  ABO40828.1; Selectin - Bg-c337, Bg-c361	C type lectin - Bg-c319; gram-negative bacteria binding protein - Bg-c185; Tandem repeat galectin - Bg-c5912, Bg-c23516, Bg-c2862; thioester-containing protein - Bg-c633	C1q domain containing protein - Bg-c95; Peptidoglycan recognition protein - Bg-c106, Bg-c140, Bg-c172; Tandem repeat galectin ABS28869.1| ; Thioester containing Protein (TEP1 B. glabrata) gb|ADE45332.1|	Beta-glucan recognition protein gb|ABL63380.1; C-type lectin - Bg-c97; C1q domain-containing protein - Bg-c13136, Bg-c7382; Fibrinogen-related protein - Bg-c2677; Gram-negative bacteria binding protein - Bg-c452; VWA domain-containing protein - Bg-c535, Bg-c22848	
Regulatory networks, signaling	Calcium binding protein - Bg-c4822; Calmodulin - Bg-c9303; Kunitz-like proteinase inhibitor - Bg-c516; Serine protease-AF302260_1; Serpin -Bg-c598, Bg-c29589; Serpine 1 mRNA binding protein - Bg-c2715	Calcium binding protein - Bg-c1743; Calmodulin - Bg-c4483; Cathepsin - Bg-c37884, Bg-c40030; Cathepsin Z - Bg-c267; Cathepsin-L - Bg-c138; Cathepsin-L-like cysteine peptidase - Bg-c1945; Chymotrypsin-like elastase - Bg-c664, Bg-c7544; Kazal-type proteinase inhibitor - Bg-c9789; Metalloproteinase -Bg-c1345; Protease inhibitor - Bg-c3896; Proteasome alpha type 2 - Bg-c488; Serine protease - AF302259_1; Serine/threonine-protein kinase - Bg-c193; Serine/threonine-protein kinase - Bg-c509; Tyrosinase - Bg-c1276; X-box binding protein - Bg-c785	Calmodulin - Bg-c806, Bg-c2503; Cathepsin L - Bg-c11016, Bg-c931; Cyclin –dependant kinase - Bg-c5295; Kazal-type serine proteinase inhibitor - Bg-c16; Proteasome subunit beta - Bg-c2531; Protein Phosphatase 2A Inhibitor I2PP2A  - Bg-c809; Serpin - Bg-c12928, Bg-c28097; biquitin-activating enzyme - Bg-c23671	Arginine kinase - ADH59421; Calcium binding protein - AAV91525; Calcium binding proteins - Bg-c161, Bg-c338, Bg-c87, Bg-c17911, Bg-c158, Bg-c4377, AAV91525.1|AAV91525.1|); Calmodulin, Bg-c151; Carboxypeptidase - Bg-c803; Cathepsin B preproprotein - ABS85545.1|; Cathepsin-L-like - Bg-c579; Cystatin-type2 - AAV91521.1; Cysteine protease inhibitor - Bg-c1700; Endonuclease G - Bg-c215, Bg-c208; Kazal-like serine protease inhibitor - Bg-c355; MAP kinase - Bg-c19712; Methyltransferase - Bg-c5309, Bg-c6900; Phospholipase - Bg-c1133; Profilin - Bg-c2756, Bg-c764; Proteinase inhibitors - Bg-c1937, Bg-c625,  Bg-c5294; Serine peptidase - Bg-c85; Serine protease - AAG40234.1; Serpins - Bg-c1343, Bg-c9973; Thymosin beta4 - Bg-c1591; Transcription factor IIB - Bg-c6583; Y-box factor-like protein - Bg-c476; Zinc finger protein - Bg-c3891,  Bg-c1737; ERM protein - Bg-c1132	
Oxidative/antioxidative response 	Selenium-dependent glutathione peroxidase - Bg-c4159; NADH dehydrogenase - Bg-c14944, Bg-c6315, Bg-c2730; Ubiquinol-cytochrome c reductase complex - Bg-c1392	ATP synthase-H+ transporting, mitochondrial F1 complex - Bg-c1648; DJ-1 protein - Bg-c794; Glutaredoxin - Bg-c678; Hydroxybutarate dehydrogenase, Bg-c607, Bg-c36; Methionine sulfoxide reductase - Bg-c8804; Oxidoreductase, zinc-binding dehydrogenase family protein - Bg-c1522; Phospholipid-hydroperoxide glutathione peroxidase - Bg-c1505; Sigma class glutathione-s-transferase - Bg-c636; Ubiquinol-cytochrome C reductase - Bg-c1378	ATP synthase alpha subunit - Bg-c370; Glutathione S-transferase hydroxyacyl-CoA dehydrogenase - Bg-c69; Microsomal glutathione S-transferase - Bg-c386; NADH dehydrogenase  - AAQ75770.1, AAQ74243; Omega class - Bg-c23157: Peroxiredoxin - Bg-c2101; Thioredoxin peroxidase / thiol peroxiredoxin- Bg-c19659, Bg-c16203	ADP,ATP carrier protein/antiporter - Bg-c245; AIG2-like / GGCT-like domain containing protein - Bg-c10042; ATP synthase - Bg-c1879; ATP synthase F0 subunit 6 - NP_976171; Cytochrome c oxidase - Bg-c768; Cytochrome c oxidase subunit III - AAQ75771.1|AAQ75771.1|; Mitochondrial ATP synthase - Bg-c1242; Thioredoxin - Bg-c31489, Bg-c1875	
Stress response, detoxication, chaperones	Ferritin - Bg-c1333,  Bg-c320; HSP70 - Bg-c915; Major vault protein - Bg-c13614; Peptidyl-prolyl cis-trans isomerase cyclophilin-type - Bg-c827	Heat shock protein (HSP22) - Bg-c1590, Bg-c1012; Peptidylprolyl isomerase-cyclophilin A - Bg-c3107; Soma ferritin - Bg-c1720; Universal stress protein G - Bg-c1547	Ferritin - Bg-c24; Histone 2A - Bg-c403; Peptidyl-prolyl cis-trans isomerase - Bg-c2760	Cadmium-metallothionein - Bg-c344	
Effector proteins	Escaping/achacin/aplysianin precursor - Bg-c114; LBP-BPI - Bg-c23257		LBP-BPI - Bg-c3171	Putative toxin - Bg-c1109, Bg-c207; G-type lysozyme - Bg-c73, Bg-c40073	
Carbohydrate degradation		Cellulase-endoglucanase - Bg-c305, Bg-c23287; Chitinase Bg-c2436, Bg-c8084; Endo-1,4-beta-mannanase - Bg-c260, Bg-c4791; Endo-beta-1,4-glucanase - Bg-c76, Bg-c2313, Bg-c852; Glycoside hydrolase - Bg-c4472, Bg-c4403			
Other	Apextrin - Bg-c1325; Lysosomal-associated transmembrane protein - Bg-c1994; Repetitive proteins - Bg-c29407, Bg-c1303, Bg-c10916; Sialic acid acetylesterase - Bg-c737	Like-Sm- -protein - Bg-c3927; Malate dehydrogenase - Bg-c563; Ribonuclease - Bg-c8169; RNA helicase - Bg-c6918; Schistosomin - ABW90145.1	Annexin - Bg-c3964; Heparanase –like - Bg-c5137; Hydroxysteroid dehydrogenase - Bg-c867	Death-associated protein - Bg-c464; Desmosomal cadherin - Bg-c4610; MMucin-like protein - Bg-c3969; Ornithine decarboxylase - Bg-c1810; Translationally-controlled tumor protein homolog - Bg-c1040; Transmembrane emp24-like trafficking protein - Bg-c13477	
