# Supplementary material for: Specific versus Non-Specific Immune Responses in an Invertebrate Species Evidenced by a Comparative de novo Sequencing Study
Source: PLoS One. 2012 Mar 12;7(3):e32512. doi: 10.1371/journal.pone.0032512 (PMC3299671; doi:10.1371/journal.pone.0032512)
Supplement: Figure S3 — Alignment of the complete sequences of predicted calmodulins from B.glabrata . Biomphalaria glabrata sequences (Bg) showing the typical combination of 4 EF-Hand domains (Pfam reference: accession no. PF00036) of calmodulins have been aligned (MUSCLE software; [93] to sequences from Homo sapiens (Hs) Mus musculus (Mm), Macaca mulatta (Macaca), Arabidopsis thaliana (At), Crassostrea gigas (Cg), Chlamydomonas incerta (Ci), Schistosoma mansoni (Sm), Aplysia californica (Ac), Pinctada fucata (Pf), Salpingoeca sp. ATCC 50818 (Ssp), Patinopecten sp. (Psp). The four significant EF-hand domains (e-value<1.10-6) are positioned on the alignment. (DOC) [file pone.0032512.s003.doc]

***Bg-*c4483** - ---MAAETPQDPPQDFPKELKEAFVLFDKDGDGKITYKELGTVMRTLGHNPTDSELQDMIQEVDADGNGTIDFNEFVAMMSRHNSETDRDKELREAFKVF-97

***Bg-*c2503** - ---MAEGLGEDQVKEL----KEAFNLFDKDGDGFITTSELGTVMRSLNQNPSESELEDMINEVDADGSGTIDFNEFHRMMARKLKETDTEEELKQAFRVF-93

*Hs-*NP_005176- ---MADQLTEEQVTEF----KEAFSLFDKDGDGCITTRELGTVMRSLGQNPTEAELRDMMSEIDRDGNGTVDFPEFLGMMARKMKDTDNEEEIREAFRVF-93

*Pt-*1CLM - ----AQELTEEQIAEF----KEAFALFDKDGDGTITTKELGTVMRSLGQNPTEAELQDMINEVDADGNGTIDFPEFLSLMARKMKEQDSEEELIEAFKVF-92

*Ci-*ABA01113 -MATNTEQLTEEQIAEF----KEAFALFDKDGDGTITTKELGTVMRSLGQNPTEAELQDMISEVDADGNGTIDFPEFLMLMARKMKETDHEDELREAFKVF-96

*At-*AEE34505 - ---MADQLTDEQISEF----KEAFSLFDKDGDGCITTKELGTVMRSLGQNPTEAELQDMINEVDADGNGTIDFPEFLNLMAKKMKDTDSEEELKEAFRVF-93

*At-*AED92947 - ---MADQLTDDQISEF----KEAFSLFDKDGDGCITTKELGTVMRSLGQNPTEAELQDMINEVDADGNGTIDFPEFLNLMARKMKDTDSEEELKEAFRVF-93

*At-*AEC09932 - ---MADQLTDDQISEF----KEAFSLFDKDGDGCITTKELGTVMRSLGQNPTEAELQDMINEVDADGNGTIDFPEFLNLMARKMKDTDSEEELKEAFRVF-93

*At-*AEE79567 - ---MADQLTDDQISEF----KEAFSLFDKDGDGCITTKELGTVMRSLGQNPTEAELQDMINEVDADGNGTIDFPEFLNLMARKMKDTDSEEELKEAFRVF-93

*At-*AEC07925 - ---MADQLTDDQISEF----KEAFSLFDKDGDGCITTKELGTVMRSLGQNPTEAELQDMINEVDADGNGTIDFPEFLNLMARKMKDTDSEEELKEAFRVF-93

*At-*AEE77831- ---MADQLTDDQISEF----KEAFSLFDKDGDGCITTKELGTVMRSLGQNPTEAELQDMINEVDADGNGTIDFPEFLNLMARKMKDTDSEEELKEAFRVF-93

***Bg-*c151** - ---MAEQLTEETIAEF----KEAFSLFDKDGDGTITTKELGTVMRSLGQNPTEAELQDMINEVDADGNGTIDFPEFLTMMARKMKDTDTEEELREAFRVF-93

*Cg-*ABU97105 - -------------AEF----KEAFSLFDKDGDGTITTKELGTVMRSLGQNPTEAELQDMINEVDADGNGTIDFPEFLTMMAKKMKDSDSEEELREAFRVF-83

*Hs-*NP_001734- ---MADQLTEEQIAEF----KEAFSLFDKDGDGTITTKELGTVMRSLGQNPTEAELQDMINEVDADGNGTIDFPEFLTMMARKMKDTDSEEEIREAFRVF-93

*Mm-*AAH54805- ---MADQLTEEQIAEF----KEAFSLFDKDGDGTITTKELGTVMRSLGQNPTEAELQDMINEVDADGNGTIDFPEFLTMMARKMKDTDSEEEIREAFRVF-93

*Mm-*AAI00302- ---MADQLTEEQIAEF----KEAFSLFDKDGDGTITTKELGTVMRSLGQNPTEAELQDMINEVDADGNGTIDFPEFLTMMARKMKDTDSEEEIREAFRVF-93

*Mm-*AAH50926.1- ---MADQLTEEQIAEF----KEAFSLFDKDGDGTITTKELGTVMRSLGQNPTEAELQDMINEVDADGNGTIDFPEFLTMMARKMKDTDSEEEIREAFRVF-93

*Macaca-*XP_002805228---MQADQLTEEQIAEF----KEAFSLFDKDGDGTITTKELGTVMRSLGQNPTEAELQDMINEVDADGNGTIDFPEFLTMMARKMKDTDSEEEIREAFRVF-94

*Ssp-*EGD74330- ---MADQLTEEQIAEF----KEAFTLFDKDGDGTITTKELGTVMRSLGQNPTEAELQDMINEVDADGNGTIDFPEFLTMMARKMKDTDTEEEIREAFRVF-93

*Sm-*ADW78835- ---MADQLTEEQIAEF----KEAFSLFDKDGDGTITTKELGTVMRSLGQNPTEAELQDMINEVDADGNGTIDFPEFLTMMARKMKDTDSEEEIREAFRVF-93

*Psp-*P02595- ---MADQLTEEQIAEF----KEAFSLFDKDGDGTITTKELGTVMRSLGQNPTEAELQDMINEVDADGDGTIDFPEFLTMMARKMKDTDSEEEIREAFRVF-93

*Sm-*ADW78836- ---MADQLTEEQIAEF----KEAFSLFDKDGDGTITTKELGTVMRSLGQNPTEAELQDMINEVDADGNGTIDFPEFLTMMARKMKDTDSEEEIREAFRVF-93

*Pf-*AAQ20043- ---MADQLTEEQIAEF----KEAFSLFDKDGDGTITTKELGTVMRSLGQNPTEAELQDMINEVDADGNGTIDFPEFLTMMARKMKDTDSEEEIREAFRVF-93

***Bg-*c806**- ---MADQLTEEQIAEF----KEAFSLFDKDGDGTITTKELGTVMRSLGQNPTEAELQDMINEVDADGNGTIDFPEFLTMMARKMKDTDSEEEIREAFRVF-93

*Ac-*NP_001191509 ----MADQLTEEQIAEF----KEAFSLFDKDGDGTITTKELGTVMRSLGQNPTEAELQDMINEVDADGNGTIDFPEFLTMMARKMKDTDSEEEIREAFRVF-93

*Hd-*ABU43070- ---MADQLTEEQIAEF----KEAFSLFDKDGDGTITTKELGTVMRSLGQNPTEAELQDMINEVDADGNGTIDFPEFLTMMARKMKDTDSEEEIREAFRVF-93

*< --- --- EFhand --- --- > < --- --- EFhand --- --- > < --- ---*

***Bg-*c4483** -DRDGNGLISAEELRHVMSTLGETLRDEDVRDMMREADKDGDGHINYEEFVAMMNAK---------------------------------153

***Bg-*c2503** -DRDQNGYISSSELRQVMTSLGENLTDDEVNEMIREADVDGDGQVNYEEFVRLMTTKQMGDSRN--------------------------156

*Hs-*NP_005176 -DKDGNGFVSAAELRHVMTRLGEKLSDEEVDEMIRAADTDGDGQVNYEEFVRVLVSK---------------------------------149

*Pt-*1CLM -DRDGNGLISAAELRHVMTNLGEKLTDDEVDEMIREADIDGDGHINYEEFVRMMVSK---------------------------------148

*Ci-*ABA01113 -DKDGNGFISAAELRHVMTNLGEKLSEEEVDEMIREADVDGDGQVNYEEFVRMMTSGATDDKDKKGHK----------------------163

*At-*AEE34505 -DKDQNGFISAAELRHVMTNLGEKLTDEEVEEMIREADVDGDGQINYEEFVKIMMAK---------------------------------149

*At-*AED92947 -DKDQNGFISAAELRHVMTNLGEKLSDEEVDEMIREADVDGDGQINYEEFVKVMMAK---------------------------------149

*At-*AEC09932 -DKDQNGFISAAELRHVMTNLGEKLTDEEVDEMIKEADVDGDGQINYEEFVKVMMAK---------------------------------149

*At-*AEE79567 -DKDQNGFISAAELRHVMTNLGEKLTDEEVDEMIKEADVDGDGQINYEEFVKVMMAK---------------------------------149

*At-*AEC07925 -DKDQNGFISAAELRHVMTNLGEKLTDEEVDEMIKEADVDGDGQINYEEFVKVMMAKRRGKRVMAAKRSSNSAEYKEKNGRRKSHCRIL-181

*At-*AEE77831 -DKDQNGFISAAELRHVMTNLGEKLTDEEVDEMIREADVDGDGQINYEEFVKVMMAK---------------------------------149

***Bg-*c151** -DKDGNGFISAAELRHVMTNLGEKLTDEEVDEMIREADTDGDGQVNYDEFVKMMTTK---------------------------------149

*Cg-*ABU97105 -DKDGNGFISAAELRHVMTNLGEKLTDEEVDEMIREADLDGDGQVNYEEFVRMMTSK---------------------------------139

*Hs-NP*_001734 -DKDGNGYISAAELRHVMTNLGEKLTDEEVDEMIREADIDGDGQVNYEEFVQMMTAK---------------------------------149

*Mm-*AAH54805 -DKDGNGYISAAELRHVMTNLGEKLTDEEVDEMIREADIDGDGQVNYEEFVQMMTAK---------------------------------149

*Mm-*AAI00302 -DKDGNGYISAAELRHVMTNLGEKLTDEEVDEMIREADIDGDGQVNYEEFVQMMTAK---------------------------------149

*Mm-*AAH50926.1 -DKDGNGYISAAELRHVMTNLGEKLTDEEVDEMIREADIDGDGQVNYEEFVQMMTAK---------------------------------149

*Macaca-*XP_002805228 DKDGNGYISAAELRHVMTNLGEKLTDEEVDEMIREADIDGDGQVNYEEFVQMMTAK---------------------------------150

*Ssp-*EGD74330 -DKDGNGFISAAELRHVMTNLGEKLTDEEVDEMIREADIDGDGQVNYDEFVKMMTSK---------------------------------149

*Sm-*ADW78835 -DKDGNGFISAAELRHVMTNLGEKLTDDEVDEMIREADIDGDGQVNYEEFVKMMTAK---------------------------------149

*Psp-*P02595 -DKDGDGFISAAELRHVMTNLGEKLTDEEVDEMIREADIDGDGQVNYEEFVTMMTSK---------------------------------149

*Sm-*ADW78836 -DKDGNGFISAAELRHVMTNLGEKLTDDEVDEMIREADIDGDGQVNYEEFVTMMTTK---------------------------------149

*Pf-*AAQ20043 -DKDGNGFISAAELRHVMTNLGEKLTDEEVDEMIREADIDGDGQVNYEEFVKMMMSK---------------------------------149

***Bg-c*806** -DKDGNGFISAAELRHVMTNLGEKLTDEEVDEMIREADIDGDGQVNYEEFVTMMTSK---------------------------------149

*Ac-*NP_001191509 -DKDGNGFISAAELRHVMTNLGEKLTDEEVDEMIREADIDGDGQVNYEEFVTMMTSK---------------------------------149

*Hd-*ABU43070 -DKDGNGFISAAELRHVMTNLGEKLTDEEVDEMIREADIDGDGQVNYEEFVKMMTSK---------------------------------149

*EFhand --- --- > < --- --- EFhand --- --- >*
