# Supplementary material for: Specific versus Non-Specific Immune Responses in an Invertebrate Species Evidenced by a Comparative de novo Sequencing Study
Source: PLoS One. 2012 Mar 12;7(3):e32512. doi: 10.1371/journal.pone.0032512 (PMC3299671; doi:10.1371/journal.pone.0032512)
Supplement: Text S1 — Consensus sequences of contigs (43,238) with automatic annotation. Sequences are presented in a FASTA format. The sequence accession number is followed by the sequence length (len), the number of ESTs aligning in the contig (count), the presence of an InterPro domain (IPR) and the five best hits with the SwissProt database (blastx-SP). (RTF) [file pone.0032512.s005.rtf]

>Bg-c38997 len=253 count=2 IPR:/ blastx_SP:/
ATGAATAATTCACTAATTCAATCATTTCAACACGCGAATATCTACAACTTTAATAGCCGCCCGGTAAATTTCTTTCTCTTTCACCTCTTTATCTGTGGTGCCCAACACAACATCTCCATTTCTCCTTTTCCTTGACTAAAATCTCGTTCAAAAACAGGCCCGTTCATTCTTATATATTGGGTTCGTTGTCTCTTCTTTTTTTCCTTGAAGGAAGATCATTGCGAGCTCTGAGGATCTTGTGATGTATGCATAC
>Bg-c6539 len=1633 count=17 IPR:/ blastx_SP:/
ATTTCTAAATTGTAAATGTGTTATCAATTTGCTAGCCTACTATAATTATTTAGCGTTAGCTAATGAGAGCTAGATTGATTCATGTGTTTATTTTTTATTTTGTTTGAGGAAAGCAAATACCGGTATATTTATATTTTTTTATATGTTACTCTAAGGCTCATTTAGTTTCTTATTCTATTTGGTTGTTATAATATACAAAATTCTGTGGGTTCTTTTTTTTTTTTTTTTCAGCTTTCTGCTTTGCCATTTTTTTAGTGTTAAATGCAAAATAAGCTATTCTAAAATTAAAAACAAAAAATTATTTACAAAATAATTTCTGAAATGTTAGTCATTTTCAAATTTTTCTGAATTTAAAATCATTTAGTAACATTTTTTTTAAAGTTTATAATATAAAAAATTACTGGTTTTTAAAACAATGTTAATATTTTTGTCTTGAGTATAAAAGTAATACTATGATTTTGTCTGCAACCCAGCTAATTGCCTAATTCATAAGCTGAATAGAATTTTAATGGTATACTCTTGTAATAATGCATTAAATGTACTTGAAAGAAAACCCTGTGACTATGGTATAAAAGTTTTCTTTACCTGATTCTATCCAAATCCCTAATAACTTGTGCAGATATCTTGCATTCTGGAAACAAAGGGAGAGGTCTCTACTCAGTCATCTCATGTTTAGATGATGAACTAAAGCAGCCAATGTTAACATTTGCGAACAAAAATAAAATTGTTCTTTTTTTTTCCCTTCTACTGTTTATTCCAAATAGCTTTATTTACTTTTTGAAACATTTTTTTAAAATGGTGGATGTTTTGCTAAACAGTCAGATCAGAAGATATTTATTTACCTAATGGATGGGCATAGTATTGTTTAGATATAGTAAAAAAAAATTGTACTTGTCCTAGTATTGTTCTCTTGTCATACTAACTACAACACCCACAAACAGTAGAAACAACAATTTTGTTTTAATCAGTACCATCAATTTGTTTTTTCTATTCTCTGATTTCTAAAAATTAAGTTTTTCTCAACTGTACATTCAGTAAAGTTGAAGCTCTGATGCACTGTCGTGCTCATGTTTATGACTTGCTCTATTGTTATATGAACTGTTTTTGTTAGTTAACATAGGAAACATTACATCTCTTGAAAGTAAGATTATTTGTGACAAAAACTTAATCCTATCACTAGAATGGTGACCATCCAAGTAATTCTCATTACGGGTACTTCAGTTTTAGGTAAAGCACTCGAATTTATGCTGTGTACAATGTTGCTTTGAATGATTTTGTGTTTTTATTTTAACCATTAAAAAAAATAATTGAAATTTTAGTTATATATTTGTTTACATATAACCTATTGAATAAAGGACCTAAATTCAATTTGATAGACAATAATTGTGTATTAATTAGGAAAAACATTCCTATGTTGTTATTCAATTAATTTTCTATCCATTCTGCTGGCTGAACCACCACAATCATCTCCTTTAAAATTTAGCAAATAGTCACATTAAGTAAAATGGCTTAAGTAGTTTGACAGTATAATAATTAAATCTATTGTATTTTAAATACCAGTATGAGATGTCTGTCAATTGTTTACCATACCATACCATCCCCTCCATGGGATCAAAATAAAAAAATATATTCT
>Bg-c33978 len=312 count=2 IPR:/ blastx_SP:/
ATCCAGATCTACCCAAAATGGACATCAATTTTTAGTCCCAGATCTAGATTGTAATATTTTAAATGTAGATCTAGATCTAGAATGTGCTGGACTGCTTGAACATATCTATACCAATCGATTTCTGCTTAGGGGAACTTAAAAAATTCTTTAATCATTGATTCTAGTTCTGATCTTGGGCTCAACCATCTTCACTAAAACAATGTAAACCCAGCTATTGTATGTAGATCAAGATCTAATCCATCCTAAATCTAGAAACTAGATGCTACATCTAGATCAATTATTTAATCTAGAATGTATTAGATCTAATAAAAA
>Bg-c22907 len=893 count=3 IPR:/ blastx_SP:/
ATTTTTAATTTTTTTTAAAAGTAAATGCTATTTGCCCTTTTGGGTGGCAGCATCTTTTAAAAATAGCAATGTCAGCTTTGGAACTGCTAAATGGTCTTTTGTTTCTGTCATTTCTAAAAAATATATGTATTCATATTGTTTCTAGGGTTGCCAAATCGTCGGTAAAAAAAAATAGACTTAAATTATTTTTTTTTTCAAAATTATGTCACTAAGAGAAAAAAAAAGTATGCCAATCAAAATGAGCAATTACAGACATTACTGTCCAAGTAATTCATTGACATATTGAGACTAACAAGTAACAACTGTCATAAACATGTTTAAAAAATAGTCTGCTATAAATGTGTAAAGACATGCAAGATTCAGAAAGGTGTATGTTACAAGTTTTTATATATGAAGTTGGCAACCATGACCTTTATGCTTTTTAAAAAAAAAAAAGTTTCTTCAACCTATTTTTTTTTTTTTGTAATATAAAAAAGAAACTGCTGAGGAGAACATAATTCTTTTTGCTGCTAATTTGCAGCAGTTAGTGTCTATCTAGTCCCTCAACACAAGTGCTGCTTTTTTAAAGTTGGCAACTGCTTGACTTTTGTCTTTGATGCTTTTCTTTGTTTGTTTTATTATTTTGTTTAAAGCTGATTTAATTCTTATTAATTTTGTTTATGCAAATGCTTAAAACAAAAAGGCATCAATCAAATTGTTTAAGTTTAATTTTTTGATGTTTTTTTTTTAGTTGAATAATTAATTGCACTGAATAATGTATGAGGCTGGAAAAAGTTGGTTATTTTTTTTTGGACAATAATGCCCATTTATTTTGTTTTTTTGTGTGCATTTTTTTTTATTGCAAATTCTTTCTTTATTGATTTATTTTAAAGAACGTACCAATATGAGAAATA
>Bg-c37109 len=269 count=2 IPR:/ blastx_SP:/
CCAAATCCTAGTCTCTGCACTAACCTTCAACTTTGTGTGTCTGACTGGCTATTATGTTTGCTGGTTTCATACTTTTACATTTAAGAATAACATTAAGTTTTTGATGATTTTTATTTTCATTTTATTTGGTATTATCATTAACATTGTTTTAAAAAAGAAGAAAAAAAAAACAATTTTGTTCTAACTAAAATGATTCTAGTGTGTGAAGAAATATGTGTTATCTTTGTGTGAAGTAGTACTTTTAATGTATGCCTAGAAGGTACAGCTAT
>Bg-c28756 len=230 count=3 IPR:/ blastx_SP:/
CCGTACGCATCGTTAGCAAACCAATACTGTGCCCCTCTTCACTACCAGATGACACTTGTATGACGACACGAGATTTCTAGCCCGAGCTAATTGAATCCCACATGATTACTAACTACCAGACAACTAATCCTGAACACTACTTGACCACCATGTCAGATACTATGTAATCTCACCATATTAACAATGGATACTAATTGATCACAAGGTATTTTACCATTTAGGATCTCAAC
>Bg-c32909 len=344 count=2 IPR:IPR006995:ATPase, F0 complex, subunit J blastx_SP:sp|O13931|ATP18_SCHPO/ATP synthase subunit J, mitochondrial OS=Schizosaccharomyces pombe GN=atp18 PE=2 SV=1/3e-08/284-132/1-51 // sp|P81450|ATP18_YEAST/ATP synthase subunit J, mitochondrial OS=Saccharomyces cerevisiae GN=ATP18 PE=1 SV=1/9e-04/269-132/2-47 // 
GAAGTATCTTCTTGCACCACTGCGCCGTGGGCTCCGTCGGACGCCGCCGCGACTTTGATTCTACCGTTCCTCCTCGAACTGCCGAGTCGACCGCATTGCAACGCTTAGTGGGCAGCGACCGGGACGCGGTGCGGATTACGACTGTCGTCACGGTACTCGGGTGCGGTGAGCATGCCGGCCTGGGCCTTGGCGACCAAGTAGAAGGTCATGGCGCCACCGACCATGAAGGGCCACATAGGGCGGAGGAAGGGAGTCGGCCAAGCGCGCATGCCAAACATGGTTAAGTAGCTGCAGGGGCGTACGCGGGTCAGTTGACGGCGCTCGGGTAATTGCACAAGGATCTA
>Bg-c17836 len=345 count=5 IPR:/ blastx_SP:/
GTTGTTGTTTTGTTTTTAAAAGAACAATAACAATAAAACAAAAATATTGCACTAAAAATGAGTTGGGATATTTATAGAAAAAAACAAAGTTATGACCGACCCATCGCTGATGAAAAAGAATACAGCCAAAGAACTAAATCCATTGTTTCATGAGAACCTCTATTGTTACATGAACTCATCAGCATTGAAAAAACTTTGCTGTCAAAGAAACAACCCATCACTGATAAGTAAATCAGTGTGGAAACAATCCATCAGTCCTTGCAGAAACAGCCCATCACTGATACGTAAATCAGTCCTTTGCAGAAACAACCCTATTTCTACTGATAAGTTAAATCAGTCCTTGCA
>Bg-c41443 len=226 count=2 IPR:/ blastx_SP:/
GAATACTGCAAGTAAATTCAATTTGACTTATGGGCTGTATTAGAATGATAGTGACGCAACAGTTTCTTTAATAAACAAAAATGACTTTGTACATTGTACTGCTTCAAAGTTTGTTTTATATGCTGTGAGCTGATTTCATTAAATTATTTTGAAAATTAAAAAAAAAAAAAAAAAAAAGAACAGATGAGTAGTATTAAACACACAAACACCACAGTTACATTAGCAA
>Bg-c30357 len=549 count=2 IPR:IPR002935:O-methyltransferase, family 3 blastx_SP:sp|P93711|CAMT_POPKI/Caffeoyl-CoA O-methyltransferase OS=Populus kitakamiensis PE=2 SV=1/5e-08/187-444/12-102 // sp|Q8H9B6|CAMT_SOLTU/Caffeoyl-CoA O-methyltransferase OS=Solanum tuberosum GN=CCOAOMT PE=2 SV=1/3e-07/187-438/37-106 // sp|O49499|CAMT4_ARATH/Probable caffeoyl-CoA O-methyltransferase At4g34050 OS=Arabidopsis thaliana GN=At4g34050 PE=2 SV=1/5e-07/187-438/54-123 // sp|P28034|CAMT_PETCR/Caffeoyl-CoA O-methyltransferase OS=Petroselinum crispum PE=1 SV=1/6e-07/187-438/36-105 // sp|O24151|CAMT4_TOBAC/Caffeoyl-CoA O-methyltransferase 4 OS=Nicotiana tabacum GN=CCOAOMT4 PE=2 SV=1/6e-07/187-438/37-106 // 
TATTTAACCAAGATGTCACAGAATCCCAGAAGAAGAAGATATCATGATCCAGCCATTGATCAGCTAATCAAAGCCATAGACCTGGCAGAGTCGACCAACTCAGCTCCAGAAGTCATAAAAGGTCTTAAGTATGCCCTTGAGTTGGTTCAACTAAGAGATGATTTTACCAAATCATCAACTTCAGCTGAAAGTGAAGCCTGCAAGAATATCTTGGAAGAGACTCTCAAGCATGACTGGGCAGCAGTGCATGCAGAGGGAAAGACAACTTGGAGACTTTCTCCAGGAATGATGTCTGGATCAGTGGAAGGTCAGTTTCTCAAATCATTTGTTAGTGCTCAGAAGGCTAAAAGAATCCTTGGTGTCGGAATGTTTACCGGATACAGTGCCCTTTCAATGGCAGAAGCTTTGCCTGCTGATGGAGAAGTTGTAACAATCGACCAAGATGACTATTTAAAAACTTTGGTTGAAGACAGTTTCCTGAAGAAATCTCCACACGGTTACAAGATCAAAATATATGGGAAGCACCAGAGTATCAACAGTGTCTGACAG
>Bg-c8759 len=694 count=12 IPR:IPR001841:Zinc finger, RING-type blastx_SP:sp|Q62210|BIRC2_MOUSE/Baculoviral IAP repeat-containing protein 2 OS=Mus musculus GN=Birc2 PE=1 SV=1/2e-08/487-344/565-612 // sp|A9ULZ2|BIR7B_XENLA/Baculoviral IAP repeat-containing protein 7-B OS=Xenopus laevis GN=birc7-b PE=2 SV=2/4e-08/487-344/298-345 // sp|Q8JHV9|BIR7A_XENLA/Baculoviral IAP repeat-containing protein 7-A OS=Xenopus laevis GN=birc7-a PE=1 SV=1/4e-08/487-344/354-401 // sp|Q13489|BIRC3_HUMAN/Baculoviral IAP repeat-containing protein 3 OS=Homo sapiens GN=BIRC3 PE=1 SV=2/5e-08/487-344/557-604 // sp|Q90660|BIR_CHICK/Inhibitor of apoptosis protein OS=Gallus gallus GN=ITA PE=2 SV=1/7e-08/487-344/564-611 // 
ATTTTTATTTATTTATTTTTTTCACGTTTTGGACGCCTCTTTAGGAATAAGATTTTTACGTCCTAGTTTAATCTTGTGCTGGACGGCTGGAGATGGCAGCTGGCTGGGTTCAAACCCGAGACCATCGTCACGATAGTATGAAGCACGTACCACATGACCGGGCAGCCAATTAGATCTAGTGAAGGACTGGCGCAGTATGTGACTTCCAAAAACTATTGGTATGAGGTATAAAAAAAAGGAACAAGCCACAACTTTTCGATGAACCATCAACTTCACCATTACTAGCTAGATACTCCTCAGTCGACTTCTTCTTCTTTTCATTTAAACTTTGGAATGACGTTTAGGCGAAAAATGTCGTGACGGTTCCTAAAATTCGACTTCTGCAATTCGGGCAATGAGATAACTGGTTAGCACACTCTTCACACGCGTACAAGTCACCACAAGGGAGGAACAGGGACTTGCTTGGCCGCTGTCTACATACCTTACAATTCATCAATTCTTTCAAGTTAGTATTCTCCTTCTTCAAAAGTTTAGATTCAGTACTCTGTGACCCTTCTGGTCTGGTTTCACTATTATTGATATGTTGCAATGTTCCAAGCTGTTCTTTATGGTCTTTTGTCTCACCTGGTTTATTGGACTCGTCATCTTGTCTTTTTTATGACTGGGATTGTTGTCGACGCTGAGACATTCATAA
>Bg-c38041 len=261 count=2 IPR:/ blastx_SP:/
CTGGTTCAAATGGTTGACGGCAGGGATGAAAGGTAAAAATACTGATAATGAAAAGAATCTTGGATATCATTCCTTTTCTCTCAAAGCAGAATCTAGCCCTCAATGGGCATCACGAACGAGTTGAAGAAGAATTATAAACTCTCTTAAGTAGGTGGAGTGTTAGTTGCTCTTCAGTCTTAATTTTCTCACCTATTTTAGATTCTTGGCCCCTGAATTAACTGAAATAAACGACGGGTTACCTTCAAAAACAATGATCGTCAA
>Bg-c5009 len=731 count=24 IPR:/ blastx_SP:/
TTTTCTGTACTACACTATATTTATTTAAATACTAAACTTATGACACGCGCCTATTAAACAGATGTTTAAAACATGAAAATTAATACATACAAATAATTTTCAAAAAAATAACAAAGTTAGAAAAGATTTTTATATATGGGATAGGTGACAGGCTTTTAAGTACCGTATTAAATATATTTCTTTTTATGATGTATCGAGATGAAATATTTGATTGTAAACAATTAGTTGATGATTATAAACACAGTTCAACAGCCTTACTACTGAATCCGTGCAGAAATAAAAATGTTCAATCTTAGCTAATGAGCTCCTGAAGAAATGAAGAAACTAATTGTGAATAAAATAAATGAGAAATGGATAAGCTCTCATCCCAATCACAAGAAAGATTATTCCTATTATAGGCCTGAGCTATCCCGACAAGATCAACGTCTAATCTTTAGACTCAGGACTGGACACAACAGAATGCGACATCTGTACCGGAAGCTCAAAATTGGAACCCGTGAAATCTGCCCATGTGGAGTGTCACCAGAGGATGCTGACCATGTCCTTCAAAACTGTTCTCTTTACCAAGAGGCCCGTACAAGACACTGGCTCCAAAACACCCCAATAGAAAGAAAACTATATGGAGAGATCCCTGATTTGGAAACCACTGCGCAATTCATCTCATATATTGAAAAGAAGGTCCATACACATAAGAAATTGACCAGTTATTGACTTAGTACACAAATGTATAA
>Bg-c34718 len=297 count=2 IPR:/ blastx_SP:/
TTACTAATTATTTAGCATAAAGATAAACAATAGTTAATATTCTCATTTGCATACAAAAGAAGGATTGAAAAGAATAAATAAAACCAAACTATTTTCAATTTAGAAATAGAGCTTCACTTTTTAGATTAAAAATAGACATTTTGAAATGACAAAATGATGTAACAATCTTGACCTTTATAAGTGCTAGTGAGATAGAGATGGCAACACTAAGATATACTGTTTTTAAACAGGATAATTCTTACAGAGTTTTATTCTACATATCAAACAACAAATGTACTGGAGTCTCAGCTTGAAAAA
>Bg-c12024 len=588 count=8 IPR:/ blastx_SP:/
GATCTCAAAAACTTTATTTCCCTTAAAATAAATGACTAATTTCTATACAATGAAACATGTGATGAAGTGAGCTTAGTCTGAATCGGAATCTGATGAACTGCTTTCAGAACTTGATGAAGTGCTTTCAGATATTGTTTTTGATGGAGCACTTCGAGCTAATCCTTCTAAGTACATTCTTTCTTCTTCTGGTAGTGCTAACAAGTCTAAGCATGTCTCAACCAAGTCTGGGATACCTTCATAATCCAATGCTAACATTGTTGTCATTACATTCTTAGCAGCATTGTAATCAGGCACAGAGATTAATTTTTTGGCCAGCTTGCTCAATGACAGTGCACTTGGACTTGTTTTCCTTAGGCTTCTTGTGTGTTTGTATAGCTCAAATAATTCACAAGCTTTGACATGATCATCATTGTTTAGGTGGATGATAATCATTTCTCCTATGACATCCCCATCAATGGCTGGAAGTTGATGCATCTTTTCTTGTTTTAGTTTCCAAACCACCAACAAAGACTGAACAATATCACACAGTTGTCCTTTGAAGCGTTGGTTCATGTTTGTGTTGGGCAAGTGTTTTGAAAAATGTCAGGG
>Bg-c4421 len=481 count=28 IPR:/ blastx_SP:/
TTAATTTTGAAAATCTGAACTATGCAGATACAATTTTGGATGTAATATTTTTTTCTTGGTTTGAAATGTTTTTATTTTACATGATGGCTTTTCTGCATATATGAAGATTTTCTGCTTAAAACATTAGTAAGAGATATACTGCTGGTTGGGGGTGCGCGGTGGTCAAATGGTTAAGCGCTTGGCTTCTGAACTTGGGGTCCTGGGTTTAAATCTCAGTGAAGACTGGGATTTGAATTTCAGATTTTTAGAGTGCCCCTGATTCCACCCAACTTTAATGCGTACCTGACTTTAGTTGGGGAAAGTAAAGGCGGTTGGTTTTGTGCTGGCCACATAACGCTGCTTGTTAACCATTAGCCAAAGAAACAGATGACCTGAACATCATCTGCTCCATAGATCCCAAGGTCTGAAAGGGGAAACTCTTATACTGTTGGTTACTATTTACTACCCAAAATGTTTTCTTTAAATAAAGGAATCATAAAAA
>Bg-c43182 len=102 count=2 IPR:/ blastx_SP:sp|B3QFS9|THIC_RHOPT/Phosphomethylpyrimidine synthase OS=Rhodopseudomonas palustris (strain TIE-1) GN=thiC PE=3 SV=1/6e-08/2-100/147-179 // sp|P61427|THIC_RHOPA/Phosphomethylpyrimidine synthase OS=Rhodopseudomonas palustris GN=thiC PE=3 SV=1/6e-08/2-100/172-204 // sp|Q2IYP8|THIC_RHOP2/Phosphomethylpyrimidine synthase OS=Rhodopseudomonas palustris (strain HaA2) GN=thiC PE=3 SV=1/6e-08/2-100/147-179 // sp|Q133T3|THIC_RHOPS/Phosphomethylpyrimidine synthase OS=Rhodopseudomonas palustris (strain BisB5) GN=thiC PE=3 SV=1/1e-07/2-100/147-179 // sp|Q07PX1|THIC_RHOP5/Phosphomethylpyrimidine synthase OS=Rhodopseudomonas palustris (strain BisA53) GN=thiC PE=3 SV=1/1e-07/2-100/147-179 // 
TGTCGCCCATCGCGAAAACCTCTGCCGCGAACAGATGCTGGAGCGCGCCGAGGCGGCCCTGGCGGACGGCGAGAATTTCGGCGCGGCGGTGCCGGCCTTCAT
>Bg-c2417 len=683 count=55 IPR:/ blastx_SP:/
GCTGGATTTTTAGAAGGACTGGCTTAGAACCATTGCAACAACTGTGTTGTATACATCCTAGCTGCCTGGTCATGTGTTATGCACACTAGACTGTCGTTTGTCCGTCACTGTTGGCCCAGGTTCATACTCTGCCCCCTGTCATGAAGGGCTAGCCTACAAACATGTAAAAACAAACATCCCTACTAGTTAAAGGAACAAAGGGAGGGAACAAATAGTATTTTCTTTCCTTTGTGTTGGTTGCTTTCTTCTTCCTTTTTTTTTTTTTAAATTCAAAAGCTCTTAGTTGCATTAATTTTAATTAGAATCTCTACAAAACAATCAAATGGTTTTGATTAGTAAAGAATTGAAACCTTGACATGTTTTTATTTTTTTTTATATGTTGTATTGTCCTATAACTCATATACAATAATAAATAATGAGATTGTAGATAGCAAACATTTCTCCTCCTAAAGCAGACCAATGTATATGTTATCAATGGTAGAAAGAAATTAAAGAATAAAGATTTATTTTTTTTTCTAATTGTATACATATTATTTCTAAGTAATTGACATAAATAAACTGTAAAAAAAAATCATTTCCTCTCTTGTATATATGTCATTCACTTTGAGCAGCAAATTTTTTAACTACATTAATAAAAAGTTGAAAATTAGAAAAAAAAAAAGTCGGATGCGTTGTTACCACTG
>Bg-c19117 len=802 count=4 IPR:IPR007185:DNA polymerase alpha/epsilon, subunit B blastx_SP:sp|P49005|DPOD2_HUMAN/DNA polymerase delta subunit 2 OS=Homo sapiens GN=POLD2 PE=1 SV=1/9e-55/209-604/90-219 // sp|P49005|DPOD2_HUMAN/DNA polymerase delta subunit 2 OS=Homo sapiens GN=POLD2 PE=1 SV=1/9e-55/592-786/216-280 // sp|P49005|DPOD2_HUMAN/DNA polymerase delta subunit 2 OS=Homo sapiens GN=POLD2 PE=1 SV=1/9e-55/69-209/43-89 // sp|O35654|DPOD2_MOUSE/DNA polymerase delta subunit 2 OS=Mus musculus GN=Pold2 PE=2 SV=1/2e-52/209-604/90-219 // sp|O35654|DPOD2_MOUSE/DNA polymerase delta subunit 2 OS=Mus musculus GN=Pold2 PE=2 SV=1/2e-52/592-786/216-280 // 
GTAATGAAATCTCCAAGCTCCTGAGATCTCAATGCTGTGTTGAAGTTGATAATAAGTATATTGTCAAAGAACGCAGTTTTAGCCGCCAGTATGCTCATCTGTATGCTGAAAGATTAATGACAATGAGGAAACATCTATCAAAAGCAGCAATTAACAAGTGGGGGACTGCTTATCCTCAAAAGAAACTTCACAATTTAGTTAGTGATGAAAATGTATTATAATTGGCACTCTGTTCAAACACATGGAGCTACAGCCTAGTATATTAAAGGAAATCAGTGAAGAGCATAATTTGCTTCCTCAACCAATCAAAAGCCGTTATACTGACTCCAATGACAAATTAATCATTGAGGATGAATTGCAAAGAATAACTTTGATTGGTAAACTCAATTGCCATGATTTTGTCACAGGTATAGTTATCGCTGTCCTAGGAATGGAGCCAGAAGATAAGAAAGGAAAGTTCTATGTTGACGATTATTGCTACCAAGATCTACCTGCACAAATCTCAAGACCTATCATGGATGTTGATAAGTTCATAGTATTTGTCAGTGGCTTCCAACTGGGGTGGATTGGATGAAAGAGTTTTTCTCATGCAAAATGTTTGCTGACCTGGTGAGTGGTCAGCTGGGAGAGTCTGAACAACAGCAGGCATCCTCACACATCTGTCATGTGGTCATCGCTGGGAATAGTCTTAGTAGGTCAACTCAAGACAAAGATGCAGTAACCAAAGCCAAGTATCTCAACCAAAAATCCTCTGCTGGGAGTGTCGATGCTATCAAGAACTTGGATCTATTTTCTAATGCAG
>Bg-c41743 len=218 count=2 IPR:/ blastx_SP:/
TTTTGTTCTCTGCTGAAAATTAACAAAAATGATGGAAGAGGCTAACGATGAACAGCCAGAAATGGTTTCTCTTGAAGAAGCCGTACAAGCTGAGAAGGAAGCAATACAAGTTATAGCTTCCTCCATCCCATCAGCAACTTCGCCACAAGTGTCCAAGTCTTTGGCTGCACCGGCTCCTTTACCAGCTTTCCTTAATCTAGGCAGTGGCGACTCTAGTA
>Bg-c21774 len=278 count=4 IPR:/ blastx_SP:/
CTTACAAATTGTATTTACATCTTCAATACAGAAATACAATGATATATCTATTCAATCAGAAAATTGCTTAATAATAGTAAAAACACATAATATATAATTAACGTCTATACTGACTATAAAAAAATAGACAATATATTTACATATTTTCTTAATAAATATTTTGATCAAGGAAATAAATGTATAGAATTTTTGGCCAGTTTAGCACTTCATTTCTTCAACTTCTGTAAATATGTACCATGAGGATTCTTTTTTATTTATCTTTTTGTTTTAATGTTTAC
>Bg-c8448 len=416 count=13 IPR:/ blastx_SP:/
ACAGGTTTTAATTGTATATTTATTGTTTTTTAAATGTTCTCTTTGCAATATTTTAATCAAACCTGACACAAGTTGGCTTGATTGTTGTTTTTTCAGTGTCCTTCCATGACTTGATCTAACAATGTGAAACTTTGTATAAATAAAACCTGTGTGACTGCTGAATGATCCCACCTCTCTCAATGTAATGGATTGTGAGTGTGTTTCTATGGTGACGGAGGTTAGCGAATGTTTGTGAATAATGCAAGATAGGTGGCATTGTCGTCACTTGTTCTTAGAGGAGACGACTCCTGATCGTGAGACTGAACGGTTGTGTTATTGTAGTGAGTAAGATCTTGTTAAAAGATATTATTACTTAAGTCTACTATCAGAAGTTGATTTTGTTTATATTATAAATAAAGTTCTATTTAGTTTGTTCA
>Bg-c29086 len=161 count=3 IPR:/ blastx_SP:/
TGGGGAAGGTAAAGGCGACTGGTTGTTGTGCTGGCAATCTCATTAACCATGGGCCACAGAAACANATGACCTTTACATTATATCCCCTATAGATCGCAAAGTCTAAAAGGGGAACTTTAAAGTTAAATGTGCTGTACAGGTAGAAGTATAATTACCATTTT
>Bg-c17605 len=378 count=5 IPR:/ blastx_SP:/
AGGAACATCCGTGGGGAGCTAGAAGAAAAGAAAACCTTAAGACAGTTATACACTGGATACGTCAGATCTGTAATGGATAACTGTCTCTCCATACAAGTAGCTGCCAACAAAATCTATCAGTCATCATTAGACAATCCAAAACTAAGCCTTGCGTCTAATTAGTGGAGGTATGAGAACTACTCCCACAGCAGCCTTTGAAATAGACTCCAATATTGAACCTCTTAATTAAGTTAAGGCGCAACAGAGCAGCATTAGAAGCTATTGAGAGATACAGAAGGTTGGAGGACGATCATCCTAATAAACGACTAACACTGAGAAATAGATATATGACAAAACTAGAAATAGTAACTATTTTATTTGACAGATAGAAATAGTATT
>Bg-c32262 len=371 count=2 IPR:/ blastx_SP:/
AAACGAACCCGAAACGGACATAGTAAACGTAAAATAAGAACAAAGAAGAAAGTATGAAGAAGTGANCACGGTAAATAACTCNGCGGGAAGTCAATATGACTTGACTACCTCCCCTAGCCATCAAACTGACACTCGGGTTTCTAATTCTTTTGTTCCAAAATTTACAGACTACTAATAGCTGGAAGAGGAATTGTTTTCAAAATAAACTAGAAACAGCCATCATAATAAGTCAGTACAGACAAATTTGAAGCATCGTCATTAACAAGAGTCTTTTAAGTTGGCATTTAAAAAACAAAACTTAATCACACAAAATAGCATTGTCTAGATCAAATAGTCAAATAGATGTGTAGTTTGTATTTCTAGTGTTAACC
>Bg-c23342 len=622 count=3 IPR:/ blastx_SP:/
CCTCTAACGTCAGCATCACTCTTATCCTTTTAAATTGTGTTTCTTCTTTTTCTCAAAGTCACTCCCTCAGATGTTGTGATAATTACATCACTGTCATCTTTGGCCTTTGACTCTACGTCGCATTGATCGTCTGCCAAAACTGGATCGTCCTTGTATGGGTACCAAGCGCCTGCTGAGCGTCTGTTTTCCTCAGTTTCAGTATCAGGGTCGAAATCTTCTTCATTACCCGTGAATGCATTAAACACGTCTTTGATTCGTTGAGACTCTCGCTTCAGGCTCTCTTTATTTGTTTTAATTTTCTTTTGAATATCTTCATATTTACAGACAAATCTAATGCAGGTATTTTTAATTTCCTCAACCAGTTTCAAAAAATGTATGACTGCTGGCAATTCGGATTCGTTGTTTGAAAACCGCTTAGATAGGGTGTCACATTTATCCAGTATTTCTAAAAAATTTTCATTCGGCTCATTGAGGTCATTATCAGCTATAAACCTATAGATCATTAAGATATCGCTGACTTCCTCTAATCCTACAGCAACCTTGTCCAAGTGATTCGCACGCAATAGCGCGTCGCGACTTTGTTTCGCTAGCTCGAAACGCTTGTCAGTGTAACGATGGCCGT
>Bg-c2790 len=685 count=47 IPR:IPR015425:Actin-binding FH2 blastx_SP:sp|Q0IHV1|INF2_XENTR/Inverted formin-2 OS=Xenopus tropicalis GN=inf2 PE=2 SV=1/3e-06/26-502/867-1023 // 
AAAAGCATCGCGGTTTTCCTTAGAAAATGTCAAGTCGGAATTCAACCAAATCAAAGCTAATGTCAAGAAATTACTTCAACAGATTCAGACTCAAGCTGATGAAGAAATTAAAGACCAATTTAAAGAGTTTCTTGAGGAAGCCGAAGGGGATTTATCGGATATTGATGAGAGTCTTGAGTTGCTCGTGAAACAAAGTCACAAATTAGCTCATCATTTCTGTGAGAATGAGAACTCTTTTAATCTTGAAGAGTTTCTGAGTGCTTTCCGAGAGTTTTGTGACAGAGTAAAAGTATGTCAGCAGGAGATCTCCTCGTGGAAACAGCAAGCTGAGAAAGCTGAACTGAGAAAGAGAGCACACGAAGAACTTCTGGAGAAAAGAAAAAGTGCCCAAAAAGAACTTCCTCCAGGAGCAAGATTGATGCCTGGCCTTGCAGGTACTGGGGATGCTAAAATTGTGGACAACTTAGTCAGTGAAATTCGCAGAGGCAATGTTCTCCGACGTCTATCAGTGAAAAGAAAATCCAAACCTGTGCCTGATGTCATTACAGAAAGTGTGCGCTTGTAGATGAAATGTGTTGTATCACTTGTAGAGCTTGTGAAGAGTAGAGTTTTAAATAGAATCTTTATGTAGAACAAACAGGAAGAAATAAAACTTAAGAATACTAATAACAATTACTAGTTTTAC
>Bg-c22554 len=239 count=4 IPR:/ blastx_SP:/
AAAATACCAAGAGGCATCGTGGAGGCTGGTGTCATACAGTGTGGGTGTGATTAGCTAATTATGTCGCCCTCCTCTCTCCACATATGAACACTCCGAGAAATAAACATTCTCATATGTCATTTGATGAAGCATTCCCATATTAAATGTCATGCGATGAAACAGTCCCATATTAAATGTCATTGGATGAAACAATTCCCACTATTAAATAGTCATTGGATGAAACAATTCCCACTATTAAA
>Bg-c41540 len=224 count=2 IPR:/ blastx_SP:/
GGGGGTGGTATAAGGCTGAGAGATCGGTGCCTACTCGTCGTTAAAAGATTCAGAACTATTCTGAAAAAATGTTTCGATAAAATAAATAATATTTGCGTATGGAACTAAAGAGCCGCAGCTTCACATCCAAAGAGCCGCATGTGGCTCGCGAGCCGCAGGTTGCCCAACCCCGGCCTAGAGTCACTAGTGACATTGATGATCACAATTCAAGTCAATGAAATGAA
>Bg-c28359 len=245 count=3 IPR:/ blastx_SP:/
CAACATAAAGTTATGTCCATTGTGGATATGTCATCATATAGAAAATTTCTGTGTAGTATGCATCAAATGTGTCATCTTAAAGAAAGTTCTGTTCATTATTGATCAGATATATCATCATAAAGAAAATTCTGTACAGTACTGGTATGTCATTAGGTAGAGAGTAATCTACTGAATTGGTAAATGCTAAAACAAAAGCTGGTAGATATCTACAGTATTAACTTAAAAATACATGTAATATAAATTAA
>Bg-c1459 len=1079 count=92 IPR:/ blastx_SP:/
TTTTTTTAAAACTAACTTTGATTTATTTATTGTCAGACATCTATTATAAAGCGGCAATGTAACTATTTTACAAATTACGCGCTTCTCTTGAATCATCATTGTCTAAGATTCCTTGATGATGACATCTATTTTTTTTTCAATTTTCTTGTTAATTGTAAATCAGCAGTTCCTCTGTTAGATATGTCTATTTCTATTGGAGGCGCGGTTGCGAATTCATCCTTAAAAGTCGTTTTTTTTTCTCAGCAACTCTGCATAGCCGTTTACTACGTAAATCCACCTCTCAAACTTTCCCTTTTGTTCTTCAAACCATACCAGATGCTTTTTCGTCACGTATCTATCAATGCTCTCGAGCACAAGCTTCAGGGCGACGACCGTCTTCTCCCACAACTCCAGAACCAAGCTCGTATGCTTGATATCGAAGAAAGCGCCATTAGGCGGTAGGATGAGAACGATGGGCTTGAGATTTTTCGCCTCAGCTGGGCCGCTTCTTTTCAGCCTCCTCAGAAGCTTTAGAATGAACTTTGTCATGTCCTTGTGCATCTCGGTGTAAGTGTGCTTGAAGTCTCTAAACATTCCTTCTCTTTTCCTTCGTTTTTGTTTCGCCAGTTTTTTCGCCAGTTCTTTCTCCATTATACTCCTGAATTGATCTATTTCGTCTTGCAGCTTACGATATTGTGGTTTGCTATATATTAATTTTAGAAATTCATCGACTGCTTCCGGAGGCATTTTCATATCCGTCATTTGTGTCAATTCTTCTTCTAGCCAATACTTCTCCAAAACTTTCCAATCTCCTCTTCCGCACCCCTCTGTCTTACAGACCAAATACATGGTGAGCGGAATCAGAAGTAGCAGACTTCGGGCTATCATGTTGTACGTGTGATTTCTACTGTGGTGAATTAATTAAACATGTGTGGTGTCAGACACGGGGCTCGTTTAAGTAGCATTCTCATGACAGGTCATTTATCGAAGACGTGGATTTGTAGTCAGCTCAAAGGCGTATGCACTTTCTTGGATATATACACATAATCTAGATATCTCTTTACTTCATCATTAGTCAAAGTGTTTCAAGGCAATACGTC
>Bg-c29787 len=670 count=2 IPR:/ blastx_SP:/
TCTTTATATTCTCCATGTGGGCAGTAATGGGTGCCCATCTGGGGCCCAGTTTTTTTTGAAGTATAAATAGATGCCCTTGAGTGAAAGTAAATTTGCTAGTGCATTTGCTTCGAGGTTATTCAGTTGGTGAAATCTGAATTGTATAATCAGTGAAATTCTGACCTCGTAAAAATAAGTGCTATTTTATGTCAGAGATAGATACATATCTAGTTAATATTATTCATATTTTGTTAATTCACGATTTTGTTGGTACTTGATATAAATCTACCTCTTCTGTGAACTTTGAACTGTTGAACTTGCAACTTATTCGTAAAAAAAAAACTGGAAGTGAACTGTTGACCTTCAAATTAGAAAAGATGTCAAGCGTAACTAGTTGTTTCCCACTTCCAATGAAGATATTGGTAATACTTCAAGCACTGGCGTTGTTTCGATGTGCCGCGATGACAGATGAATTCTATGACGAGGAACCCGAATCCGAATCCAAACGTCAGTGGTCACTAAGCTCAGACTGTTCCATGTGTATCACCTACAATGACCCTGAATACTGTACAAGGTGTCTGAATAGCAAGTCATCGATTCCTTTTCACATGGCTAAACGAGCCTTTATAATGTCAAGGTCAACGAGGAATTCTGGGTGCGGTTGCTGTTTGATGAGCAGATTCACCAATAA
>Bg-c39642 len=248 count=2 IPR:/ blastx_SP:/
GTGCTGTCCATCGTGCCATGTTGGAGCAGAAGTCACAGGCGGAGACGTGGGGATCGGTAGAGTGGGCCCATGGGGTGGAAGAGGAGGAGCTGACGACACGACTAGCTGCTGCTGCTTTGTTCGTTTACTTTAACAGTAACGCCGTCACGAAAAAGACATTGTAGAGATAGAGAGAGTAGGTACATACGACTACATAGTTACCGAATGGGGAGCTGTGTATTGTGATGCGTTCTCTCAGGTAATTTTAT
>Bg-c28788 len=228 count=3 IPR:/ blastx_SP:sp|B4JBC4|MOCS3_DROGR/Adenylyltransferase and sulfurtransferase MOCS3 OS=Drosophila grimshawi GN=GH10959 PE=3 SV=1/2e-10/12-164/401-449 // sp|B4LRB9|MOCS3_DROVI/Adenylyltransferase and sulfurtransferase MOCS3 OS=Drosophila virilis GN=GJ21670 PE=3 SV=1/1e-09/9-164/403-452 // sp|B4N7R4|MOCS3_DROWI/Adenylyltransferase and sulfurtransferase MOCS3 OS=Drosophila willistoni GN=GK18675 PE=3 SV=1/2e-09/9-164/408-457 // sp|B4HYP0|MOCS3_DROSE/Adenylyltransferase and sulfurtransferase MOCS3 OS=Drosophila sechellia GN=GM17034 PE=3 SV=1/3e-09/9-164/404-453 // sp|B4KI53|MOCS3_DROMO/Adenylyltransferase and sulfurtransferase MOCS3 OS=Drosophila mojavensis GN=GI10453 PE=3 SV=1/3e-09/12-164/404-452 // 
ATTACACAGTTCCTGTAGTTTGTGTTTGTCGCAGGGGTAATGACTCCCAGCTAGCTGTACAGCAGTTGATGAAAGTTTTGGCAGATGATAAAGTTAAAGTCATTGATATCAAAGGTGGTCTTCATAGCTGGAGCAAACATGTTGATTCCAATTTTCCCATTTATTGATTCTACTATACATGCCTAGTTCATATGTAAAATAAAATAGCTTAATAATAAAAATCTTAAT
>Bg-c35492 len=286 count=2 IPR:/ blastx_SP:/
CATGTTCGGTGTTCGTTCAGGCTAGAAACCAATCAAAGCAGCATCCAAAACGGGGCAAAGCTTTAATATAAAATGTTGTGTTGTCAGTCTGTCATGTTTACATTTTTAAATGAAGAAAACGTTTTAAAATTCTATTTCCCCAGGTTTGTACATTGCCAAATTTAGTCATCTAGAATTTAAACCTTTTTTTTTAAATATTATGCAAGCCATATTTAAAACAATGCTTATAAAGTAAATGTATTATGACAATGGAGGACGCATGGTGCATTGCTAATAAAAATAAGAA
>Bg-c35653 len=284 count=2 IPR:/ blastx_SP:/
GTCGGCTGCGTTGCCGGCGTCTGCGAGATCTGGTCGTGCGAGGACGGCTTCTCGTTCGACGCCGACAAGGCTGCTTGCGTCGCTCTCTAAGCGCGCAGTGCTTCCTGTGTCTCTCTCCCCTGTCGTTCGCGCATTCTTTCCTTTCCTCGGTGTTCTCGACGGCCGGCGTGCCGCTTTGATTTTGCCGCCGGCACTTTTATCGCTCTCTAGCTCATATTCCCTGTAGACAAGAATCGTACCTCCATACCTTGCCTCCTGCGCCGAAAGCTCTACTTCCCCTCTCA
>Bg-c35654 len=284 count=2 IPR:/ blastx_SP:sp|Q9UKV3|ACINU_HUMAN/Apoptotic chromatin condensation inducer in the nucleus OS=Homo sapiens GN=ACIN1 PE=1 SV=1/2e-14/282-1/1030-1139 // sp|Q9JIX8|ACINU_MOUSE/Apoptotic chromatin condensation inducer in the nucleus OS=Mus musculus GN=Acin1 PE=1 SV=2/7e-14/282-43/1029-1109 // 
TTCTTCCTCTTTTTGTTCCTGCAGTTTCTTAGTGTCAATGGGTTTTTTGGCTGGTGCAGGCTGAGGCACATCACCTTCAGACTTCCTGTGCTTTACATCTTTTATGGTGGCAAAATCTACAAGAAGAATTTTTGGATTGGACTGTGGCCATCGTGTCTTGTGCAGAGCTGCTCTTGCTTTCACTGCATCTTCCTGACATTTGTAAACTACTAGACAGTGTGACTTGATATCATTGATCCAAAAGTTATCCTCATCCAGTTCACCAGACCTCTTCAACAGCTCTT
>Bg-c32286 len=370 count=2 IPR:IPR000504:RNA recognition motif, RNP-1; IPR012677:Nucleotide-binding, alpha-beta plait blastx_SP:sp|Q9JLI8|SART3_MOUSE/Squamous cell carcinoma antigen recognized by T-cells 3 OS=Mus musculus GN=Sart3 PE=2 SV=1/4e-12/293-129/794-848 // sp|Q5REG1|SART3_PONAB/Squamous cell carcinoma antigen recognized by T-cells 3 OS=Pongo abelii GN=SART3 PE=2 SV=1/9e-12/278-129/817-866 // sp|Q15020|SART3_HUMAN/Squamous cell carcinoma antigen recognized by T-cells 3 OS=Homo sapiens GN=SART3 PE=1 SV=1/9e-12/278-129/799-848 // sp|Q9W4D2|RNP4F_DROME/RNA-binding protein 4F OS=Drosophila melanogaster GN=Rnp4F PE=1 SV=1/3e-05/269-129/725-771 // sp|Q9W4D2|RNP4F_DROME/RNA-binding protein 4F OS=Drosophila melanogaster GN=Rnp4F PE=1 SV=1/3e-05/81-28/785-802 // 
ACCTTGTCAGTAGNGAAGTTTTACTTCTGGTGGATGGCTTAGAGCAACTGAGATTGTATTGTCTCCTATCACTGTCCCATCTAAGTTTTTTAAAAACAGCTTTGGGAGGCTACTTTTGTTCATCAAGTAAATTCAACATAAGCAATTCCCTTTGGTTTTCCATTTCTATAGGTCACCATCCTAACATCTTTCAGTTTTCCATGAACTTGAAACATTGTTTCTAAATCCTCTTTAGTCATTGTGAAAGGAAGACCTTTAATAAAGAGTTTTATTTTTTTCTAAGTTTGTTGAAAACTTCAGTGGTTGAGATTTAGCTACAGATCTGTCTTCACATCTTGACACAAATACTGGTCTACCATTAATGAATTCT
>Bg-c2686 len=719 count=49 IPR:/ blastx_SP:/
AGCAGTGGTAACAACGCATCCGACGCGGGGGGAAGTAACAACACAGTAGCAGTCAGCAGCCATCTGAGAACACCATGAACGCTAGACAAACTCTGAACTTGATGGTATCTGCAGCCGTTTTGGCTCTTTCTTTTTATGTGGTTGGAGTGAACGCGATATGTGAGGCCAGCAAAGGTTGTTTTACGTGCTCTAATAAAACCATTTATGCTTGTTAACTTCTGAGACAGACCAACCACGTTCAACATGAAAACGACAATGAAAATTGTTTAATCAGTAATTTATCATGTTTTCAATTATTATCAAATACTATGGGCTAACTCTATGAAGAGCATGTCAACAATGATCAATGACAACAAGAATGTTAAGAACTACATATTTATATTTATTTCAAAAAGTACCCACTCTTCTCCCTGTGCACCAATCCCATCATTTCATTGTCTTCATCATGCCATATATGTCACTAGTTTGTCTAACGTAAATCTTTAATACAACAATCAAAACGGAGTCCCATCTTAATATAAAATGGTTTGTATTTGCTTGTCATTATCTTGTAGATATTTTCAGATATTACTGAGAAAGATGCATATTAGGCTACTATCATGACTTGTGTGTACATCTGAAATACATAACACACTAGGATTGTTATACATATGTATAGATTTATTTCACCGGATTTATTCATATAATGTACATACATACAAGAAATACAGATTTGTTGT
>Bg-c19009 len=124 count=5 IPR:/ blastx_SP:/
TTCATCATTTACAAAAAAGAAGAAAAAAAGAAACCTTTAGTATTACACCCTGTAAGTTCAAATTTTGTAAAGACTGTTACACAAAATTACTTGATTAATGCTTTTTTTTNTTTCAGAGTAGCAT
>Bg-c21820 len=276 count=4 IPR:/ blastx_SP:/
GAAACTTGTCGAACTACTCGTTGGACACGAGAAGTCGAAGGAACTGGTGGAGCCAATGTTGGTGAAGCTGTGAGTTTGCGGAAGTACTGCCCGTATATTCACTGTATAAGTACCTTCAATATGTCTAGTAGGTGTTTTTTGTTCTCGGCTTTAGTATTAGATGTTACTGGTGATTAACCTAGTTTACCGGTGTTGGTGTTATTTACTTGTAGTTAGTTGTTGTTTTTCAAATTGTTTTTATTCAACGAAGTACCCTAAATAAATGGTTCGAATTTT
>Bg-c13983 len=320 count=7 IPR:/ blastx_SP:/
GTTTAGAATTTTAATGTTTCTAAAATTAAATAAAACATGATGAAAAGAAATTTTCCGTTTCTAATACATTTTAAAATCATATAATTTATTTTATTACTGGCACAAATATGGAAGGCAAAATTACAACAAATATTTACAACTTTACTTTCCAGTTCATATTTACGATATCAATTATTTCAATTTAATTTTCTAGAATCATAAACACCTATTGTTCAATGACAATAATAATCTACCAGCTTCTAAGTGATAAACAGATGGAAGCATAAAAAAACAACAACATTGAAATTAAACTTTAAAACAAACAAACAGAAACACGAGGC
>Bg-c23313 len=637 count=3 IPR:/ blastx_SP:sp|Q28GF8|TM205_XENTR/Transmembrane protein 205 OS=Xenopus tropicalis GN=tmem205 PE=2 SV=1/2e-15/276-620/7-121 // sp|Q6UW68|TM205_HUMAN/Transmembrane protein 205 OS=Homo sapiens GN=TMEM205 PE=1 SV=1/2e-14/285-635/10-126 // sp|Q5REM8|TM205_PONAB/Transmembrane protein 205 OS=Pongo abelii GN=TMEM205 PE=2 SV=1/3e-14/285-635/10-126 // sp|Q6GPW4|TM205_XENLA/Transmembrane protein 205 OS=Xenopus laevis GN=tmem205 PE=2 SV=1/5e-14/276-620/7-121 // sp|Q91XE8|TM205_MOUSE/Transmembrane protein 205 OS=Mus musculus GN=Tmem205 PE=1 SV=1/6e-13/270-635/5-126 // 
AAAATCTCTAATCGCAAAAACCTACAGTTTAAAAAAAAAAAGTTTTTAAGCGCTCAGATTTGTTTTAAGACAACTGAATCGAACGAAATTACTAAATATCAATCAACTAGATTTTTTTTGTTGACATCTTTAATTTGAAAATCATGGGAAATATAAGTCGCGTTTCAAGCAACTTTGACGCATTAAGGATGCATCATTTTCAATTCTTTGGAATGGTGGTCGTTATAAGTTTTCTCAGTTTCCTGCTCTACCCATCAAGGCGGAGAATGGACTCGCCCAGCGTGCTGTATGGCATGTTGCACCTCGGCAGCTTCGGTGCCAACTTTGGTGCGCAACTCTGGGTCACATTAGTAGCTGGGCTCACCATGTTTTACAGCCTGCCACGACACATGTTTGGTAAGGTTCAATCCAGACTGTTCCCCATGTTCTTCTTGTGGAGTCTCGTCTGTTCAGCCATCACTCTGTCCACATTCCTTGTCCAGCACCCGTGGGACACCCTGGAGACAGCTCAGCTCGTACAGGTTGCATCCCTTTCCATTTGCTTCGTTACTGCTGCCTTGAATTCCTTAGTAGTCTCCCCTCTTATCGTCAGCGCAATGTTGAAGACATTCAAGATGGAGGTTGACGCCGGGGTGGG
>Bg-c28833 len=224 count=3 IPR:/ blastx_SP:/
AGCTAACTAAAACTGATCTTTATTGATGCAGATCTTCAATCTGATGACATTGATGTCAAGCTGGATGTTTTATACATGTCTGTACCATAACAATTTCTTGAATCTGAACAAACCAGTTTCTAAACAAGTGAATTGGTAAATTGGACTTGATCCCTGTATCTTTGTTCTATCTGATAAATCAGTCACATTTGATATTTGTTTAATAAAGATGGTGACATAATGCA
>Bg-c17125 len=464 count=5 IPR:/ blastx_SP:/
CGGTATGGCACATGTTGAGATCAGGAAGTTCTTGGGCTGGTTGGAAAAGAAAACATTGATTTTGCTACGTGGTTGATCATGGTTGGAAAAACAATATAATTCAAACTACATTTATTAATTCATCCACAGTCGGTGAAGGCTGTAATGAATCTCAGGGTTTCATCGTGTCACTTCTGCCATTTTAATGTACAGACATAACAACCTTTAACTTCCAAAAAAAATATATTTTAATTTTTAGCATTACTTGCTGACATTTTCCCGGTCTTTCATTCAAAGCAAAAAAAAAAAAACAACAAAAAAACTTTGAGGAATTCACTGTGCTATTAGCAAACAAATGGTGTACAGGAATATGAATGAACCATTGCATTTCAAGATGTTTTTTGTTGGTTTGTAAGTGACTTGCTTTTAAATATGCCTGTTTATAATGTACATAAAAATTAAAAGAGAATTATAATAAACAAAAA
>Bg-c41541 len=224 count=2 IPR:/ blastx_SP:/
GAATGTTAAAAATGTTTCAATAGGGGGGCGGTTTCTTTAAACATAGATTTACGCTTTCCCTTTTGGGCTGACTGTCAATGGCCGTGAAAGAGTCTTCTGTCTATTTACCGACAGGAAAAAACGGAATTCTTCTCAACAAATTGGCAAAATTGAAGAGGTTGAATCGGAGATTTCCAGCGATAGTTCCGTTAGCAGCAGACGTCAGGTTGGCGGCTCCTTGCTGA
>Bg-c35896 len=281 count=2 IPR:/ blastx_SP:/
GCTTTTATCCAGGTCGTCTTCAAACAATGAATTGTTAAAATTCAGGTTGAAATTTACATCTTCTTCTACATCACAAAATGAAGGGTTAGATTTATATAAGTCATTTTTATGCTTGACTATTTTTTTAAACCTACTCTTGTTAATATCTAATGATTTACCAGAAAAATCAACAACACCCTCATCATCACCTGAAATAACCTCATTTATATCTGCATCACCTGACCGCCTTTTTCTTTGTAGAGCTTAGAAAACAAATCATTATATTTTTCAACTGGTTTTGT
>Bg-c28360 len=245 count=3 IPR:/ blastx_SP:/
TTCAGCAAACCAAAGAGGGAGCTGTGCATGTACCATGTCATTACGATGAAGGCCCGCCGCCATGGCGTCGTACGAACTGTCAGATGGTTCATTCACGACAACATCGGTGGGGCTTCGTTTGCCCCTCACTAGCTGACCGTTCTCAAAATCTTCTTCAACGTCATCATCGGGGCGTTTAATGAAACTGCTACCGATGGAGTCCAGCGCCCTCTTGTCATCCTCCATTTCTTCATCGTAGTCTCTTT
>Bg-c3716 len=502 count=34 IPR:/ blastx_SP:/
TTAAAGAAGCTTAGTCAGTTTCTTTGTTCTTTTAAGAAGGAACTCATTTGGTCTCTCAGGACGGATCAAAACAAATTAGTTTAGTTGCAGAATGTGTTACTTACATAGCAATACAAGTGTATTATAGGCAACATGTTACTAATTCATAATTGGCAAGTCTCGGAATCAAGTAAATTACAATTTGTCTTTGACCGTGTGATTTCCATCAGATCTTGTGTAGGGAGGTTTTCAAGTAGGAGGCCCGGTATTCCAACACTTGGGCCATGAACTCAATTGAGAGGGTGAAACAAAAACGACTTTCCATGTGTTAGGTTCTTGCCGACATTGTTTTAAATGTTTGCCACTCTATACTGATCATGTTTCTCTAACCCAATTGGGCGGAGGGCTGGGTAGGTCTCTGACTAGAACACAGGCTTTCTTACTATGTTTTGTCAATTTTGTGTACATAGCAAATGTCTTTATCACTGAATGTCCTGATTAAACCAATGAATATATTTGTGCA
>Bg-c3166 len=253 count=41 IPR:/ blastx_SP:/
AAGTAGCAATGAAACAGCTGACATAGCCAAAGCTCAACTTATGACAACAGAAACGATACATTTGTTAGTATTATGGGCCAGGACTTAGTATGAACCTTTCTTGACCACTAACTCTTAGCAAACAGTATCCTGGCCACTAATACTTACAATCAGTACGTCATGCATAATTACCAAGTACTGGTGTAGTTGCTGTGTACTATGTACAATTTTCAAGAACCAGATTAATTTTTACAAATAAAATAACATTTTAATT
>Bg-c21922 len=272 count=4 IPR:/ blastx_SP:/
AAAAATGTGTTGCTTTATTCATTTAAAAAAAAAGTAATACATTTTCCCATCACAGACAAATATGACAATTTTATTACCGATTAAAAAAAAAAGAACTTCAATAAATATTGCTTATTTTGCACATATGGATTTTATGGTATTATACATCAGCTAAAACAAGACATGAACCTGCTGCCTGGCCACAAGAGAAAAGAATCAAAAAATAAAAGGTTGTGTTTCAATTAGAATTGACTTATCTTTCACCAAATAATATCATCTAATGATTGATCAAA
>Bg-c38265 len=259 count=2 IPR:/ blastx_SP:/
ATACTAGAAATGACTACATCTATATTCAATAATTTAGCATTTAGATCTAGATCTAGATTCTAGAATTCTAGATCTAGTCTAGTAATAGAAACTACATAAAAATTAGGATAGTAGACAGAACACTTCATTATGGACAACATCTTGCAAGACAGCACAAGAGATGTTGAAGAGGACTCAGAACCTATTTGTAAAAAAGCCAAGTTGAGCAATGGCAAAACAAATGTAGATGCTGTGATTCAGTTGGAAAGTAATTCAGAAG
>Bg-c20250 len=422 count=4 IPR:/ blastx_SP:/
GAGCTCCATCTGCCCGAAGCTCCCCATTTTGAGGCGCCAGGTATCCGCATTCACCCCTTCACCTGTTAGTAAGAGCAGTTTCGCCAGGTTTAATATCTAAGCCACACGAGCAGGCCAGGTGCTGGACTTAGTTGCCAGAGGCTATTTGAGACGCATGCCATTAGGAGTATTTTATAGACAATGGGAGCTTAACCCCATTGACACCCCTGGCCATGACAACCTTTGAAACCAGTCCAGCTAGTAATATAGTAAAATCAGATATAAATCACTATAATCATGGGGAATTGCTTCATATTCCTATTCTGTTACCTCCAGAAACTTTGAAAATAGTGTGGTGGACTTGTTGAACTGGCCTCTCTCTCATCCTCACCTTCATTGAAAAATAAAAATTAGTAGCTGAAGAAATAATGTTGCATTTAAAA
>Bg-c30644 len=482 count=2 IPR:IPR001395:Aldo/keto reductase blastx_SP:sp|Q6IMN8|ALRA_DICDI/Aldose reductase A OS=Dictyostelium discoideum GN=alrA PE=2 SV=1/5e-17/345-1/1-114 // sp|Q8SSK6|ALDR_ENCCU/Aldose reductase OS=Encephalitozoon cuniculi GN=ECU01_0970 PE=1 SV=1/1e-16/339-1/4-113 // sp|Q6GMC7|AK1A1_XENLA/Alcohol dehydrogenase [NADP+] OS=Xenopus laevis GN=akr1a1 PE=2 SV=1/3e-15/327-1/10-117 // sp|P23901|ALDR_HORVU/Aldose reductase OS=Hordeum vulgare PE=1 SV=1/3e-15/327-1/18-123 // sp|O60218|AK1BA_HUMAN/Aldo-keto reductase family 1 member B10 OS=Homo sapiens GN=AKR1B10 PE=1 SV=1/3e-15/345-1/1-113 // 
AGGCCAATGTATAAGATAAAGATCTAAATACTTCACAGTCAAGTCTTTGAATGGAGCCCTAACAATGCAGCTTTAGCATTTCCTTTACCCTTGCTCTGAAGGTCCAAGTTTGCTGGTGATAAATATGTCTTTTCTTTCAAGGTTATATTTTGGCAAAAGTATTTTTAATGCTTTTCCTATGTCCGATTCATTTCTATACACAGATGCTGTGTCTATTGACCTGTACCCTGCAGCCAATGCAGCTTCTAGCACTGTATAAATAAGATCATATCCTTGTATTTTGTAGGTTCCGAATCCAATAATTGGCATTTGATGCCCGCTTATCAGATGAACCATTTTACTCATTTTGGCAAGACCACAAAATCTTCACTGTTCTTTAGTAAGCGGCGTCCTGTGTTACGATCAAGAGAACCGAGATATTACAGTTAAAAAAACTACATCCCCCGGCCGTAAGGCCGAATCCGGGCATAGGCCGGAAAGTT
>Bg-c3771 len=959 count=33 IPR:/ blastx_SP:/
CTTAAAATCCAAAAACCGGTTGGAACCAGCGGAGAAAGTGTTGCTTAAAAACGAAAAACACAATGTGCTCAAACCAATTGAGACCAAGTGTGCCGTTTCAGATACTAATGCTCAGGCAGCAGGACAAGAAACTCCAAAGAGCTGTTTCATCAGACCATCTAAAAACAAGCCTCAAGTCAGACGGGAAGCTAAATCTACGGACAGACCAAGCCCCAAAACAACAATGCCATTGAAACAAAATGAGAAAGCAGATATCAAAAGAATAGCAGCGGTACTGAAAAAATCTACAGCTGAGTCACCCCAGGTCATGTTCAAAGAGAGATCCATAGCCAAGAAAGAAAAACCCCTTGGTATCAAGAAGAAACCCGTGCCTTTGTTGCCACTTGAAATGGCGCAGTCCTTCCCTGAAGCCCAATTTAAGCATGAGACTTTGATCTCTACAGAACGAAACGCTTCGTCCAATGGCGTTGCTAGAAGATCAGTCCCGAGTCAGGCGTGCTCTAGTCAAGTACTACCTCGACCTGTCAAGGTTACTGTCCACTCTGACGAGAGCGTCATGACCATCAGAAGCTATGACCTGGATTCCGATCACGAGAATCGATCACTTCGATCCCGACCTTTGAATGCAGACAAACAGCGCAGGGTTGTTTTGAAAAAGTCTGCTTTGGACGAGATTGTGAAACAAATGCAATCACCGTAATAGACAACAAACTTTCATTAACAGATTTATATGGTACACTATCACAAATATTCTTACACTAAAAAACGGAGTCTTTTAAAAAGCAGTCTGAAGCCATTTCAATTACTTACTCAAATTAAACAAAACTAACATTAAGCACTTGTAATTCAGTGGCATGGGATTGTCAATTTTAGAAATACATATGGACGCTTGTTATATTGCTAACAATGAACTATTAATGAACGAACCATCTTCTACAAATAAATATATGTGAAATGTG
>Bg-c37110 len=269 count=2 IPR:/ blastx_SP:/
TGCCACTTGAGCAGGTGCGACCACTCTATGACAACTACCCGTCCCTGAGACGTGCTTGCAGAGACGGCAGAGTTATCATGACCACACCAGACGTCGGTAACGCTGTTCCCTTTGAGACTGAAGACTTGACCATCTTGGTGGTGGACGGAACTGTGGCCATCAGAATTCCCAAGCGTATCCCACCGTCCCCCCCCCCCCCCACTCGGAGGCTTCACCAACCCAACGACCAAAACTAAGATTCGGACCTCGTGGGCGAAGGACGCAGACCG
>Bg-c32064 len=379 count=2 IPR:/ blastx_SP:/
AAAACCCACCTTGGCTACGCTCATAGAATCTGGTGACTGATGAATGGATAATCTTATATTGAAGAAGGGGTTTTCTTGTTAATTTTGGAGGGGGTTTTAAAATCAAAATCTTCCTTAGCTATGCTCTTGGAATTCGGAGATTGTCGTTTGCATTTTTTTTTTGTTTAATTTTATAGAATTTTTATCCCCTTCGTTGGGGAATTTTTTAACTAAAAAAAACCCTTTATTATGGGGTTTTAACCTCAAAACTCCCTTGGCTGCGCTGGGGCAAGTGATGGTTTAGTATTAAAATCTCACCTAAAATAAACAAAGCAAAGCAAAAATNTAAGTCACTAAATTCGACCCCCCCCGGGCGCAGTTGGGGGGGGNGATCCCCCGC
>Bg-c23168 len=688 count=3 IPR:/ blastx_SP:/
TGTACATTAAATGTTGAGACATTATTGAATTGGACCCTTCTGGGAAAAATTTGTGACAACAAAAATGAAAAGAGTTCAGTTATAACAATGCTAAATTCTTATCCAGTGTGTGAGGCTTTTTTTCTTTTTAAGCTTTAGCTACATTTTGTATTTGGCATGCTGAAACTTTTTTTTTAATAGTTATCTTTAGTGCTTCACTTACCTTCAACCTGAAATGAGTAAGTTCTGTCAGCAAAATGTTGAAACACAACTCCTTATAACTCTAGTCCTTGATAACATTATTTGTGTTATAATAAAGGAGAAAGATAAACTGTTTCACTTACATCAAGGATCTCTTCATGCTGATATTTATAACAGTTTACAATCAGTTAATTAGTTGTTAGTCACAGATATTAATTGTTCTATAATTTCAATAATGTTTTATTAATGTTGTCCCTATAAACAGAATCGTGTCATAAAGATAGTTATTTTAAATATTGCAAGGAATAATACCGTCACAAGTTGACAATGGAAATATGTTGTGCTGTTACTTTTGGCTTGTTTTGTTTTTTAAGTGATAGGGTTTATGCTTAATAATCAGACTTTCATGAATCTATCTATCTTATAGAAAGTGTAATGACACTGATGTCTAGCAAGATCATTCTGTTTAACTCATCTAAACATTTATGTACATTGCTACAGTTAAATA
>Bg-c42753 len=141 count=2 IPR:/ blastx_SP:/
AACTACAAACAAACAAAAACCAAACAACTAAAAGAAAACGCGTTTCGAAATGCGGCGCCCTTGTCTTTTACCACTCAATCGTTGTTCAATATATGAAAGAAGGTTTACCTTGTTCATTTAATGGGACAAAAAACAACAAAT
>Bg-c30753 len=469 count=2 IPR:/ blastx_SP:/
GGAGCCTGACCCGATGGACGCCCTAGATTAACCTACAGAGATGTCTGCAATCAAGAACTTAAGGCTAGAGGCATGAAAGAAAATATGTGGGAGCTAATCTCACAAAATCGGACAGCATGGAGACAGACTGAACGTGCTGGTATGAAAGAAGGGCTGAAGACAAAAGAATGAGAAAGAGAGATGCCCTGTCAGCTAACCCTAACAACAGATGCTTTCAAATGCACTAACTGTGGCAAACTTTGCTGCTCCAGAGAGCTGCCCTGTCAGCTAACCCTAACAACAGTTGCTTTCAAATGCACTAACTGTGGCAAACTTTGCTGCTCCAGAGACATGCCCTGTCAGCTAACCCTAACAACAGATGCTTTCAAATGCACTAACTGTGGCAAACTTTGCTGCTCCAGAGAGATGCCCTGTCAGCTAACCCTATAACAGATGCTTTCAAATGCACTAACTGTGGCAAACTTTGCTG
>Bg-c11157 len=487 count=9 IPR:/ blastx_SP:/
GAACAGTTGCCAACTCAGGACCATCATGATAAGCCACAAAGGATCAGGCAGTACAATAGTTTACTTGTTCAAAATGTGTGAGAGTCTTAAAGTAGTGACATGATTTCACTACCTATATATTTGTTTGTTATTTTGGTTTAAATAATTAATCATGTATTGAAATAGGATGAAGTCATGGGAAGGGAAGGATTCAAACTATGGAAGGATGCAGCCTTTTCTTTTGACATTTATTCTTAGTATAAATTCTCAAATGTGGAATGTACAAATATAGGAATGTATAAACTTTGTATTTTGTCTTCCTACAGCCATTCTTGTTCCATCTAATAATGATTAATTATTTAAACCAAAATAACAAACAAATATATAGGTAGTGAAATCATGTCACTACTTTAAGACTCTCACACATTTTGAACAAGTAAACTATTGTACTGCCTGATCCTTTGTGGCTAATCATGATGGTCCTGAGTTGGCAACTGTTCCATCCCCA
>Bg-c7262 len=1020 count=15 IPR:/ blastx_SP:/
TTTATGTACTTGCACAAGTGTGATAATGGCCTATGAAAATATACATCAGGAAAAAAAACTGAGTAATTTTGTTTTGATAACCCATTCTATTATCTTTATATTAGAGAAAAAGTATATATGTGACCAAATCTTTGTCTTAAAAAGGTTTTTGAGAAAAATTAGTACCTGGTTAGGATTTCATTTTTAAAAAAATGCATAAAAAAACACATTGGCAAGTAGATTATTAACAAACAATTTTGATTATTTAATAAATGCTAGAAAATGAATACTTTTAGTCAACATCACACTTATAGACAGATGTGTACAACCAGAGTTCACCTCTCCCTAGAAGCTAGATCAAACAACTGCCTAATCACCAGTGACATAGCTGGGGAGGGGGGGGGGATTTGAAAATTCCCCACTTGAGGGGGGCCCCAAATGTGTTTGAAATTGTTTTTTACATTAAATTTTACACAAAATGCAGGGGCCCTAAAGAGGCAAGCCCCCCCAACCCCCAAATGATGGCCAATTCCTAGCTATGCCACTGCTGATCACTTTTCTTCTGGCGGCACTGTCTACATATTTTCTTCCTGCTACCCCAAACCAAGCTGAACCCGACCCATACCAGTAGTGTCTCATAAGCATTTTGATCCCCCACCAAACACACCAAAGTACTGATGATCTATTTTCAAAAGCTGTAAAACCGCATTAGAAACATCGTTTTCATATTAGAAACATCATTTTCATAGTACTTTAGCTTAAATCTAAAGAGCAGCCTTACTGAATTTGGACCATTCCATAAGCCATCATCACATTGTGTTTGACAGAAAAGTATTGAACATTTTGGACAGATATATTTGCTGTTTAAAAAAAGGTTGGACAAAAAAACAAAAAGATAACTGTAAAATGTGCTGAAAAGAAATAACCTGGACAGAAATAGAAAAAAAAAGGGATGGACCAAAAAATGTAGGACACTATTTCATTGTTTCACTTGTTAGAAATCACGAAGAAATAAAAATAAAATGCAATTCTATTTACAGA
>Bg-c12603 len=342 count=8 IPR:IPR003918:NADH:ubiquinone oxidoreductase, chain 4; IPR001750:NADH:ubiquinone/plastoquinone oxidoreductase blastx_SP:sp|O21406|NU4M_STRCA/NADH-ubiquinone oxidoreductase chain 4 OS=Struthio camelus GN=MT-ND4 PE=3 SV=1/1e-15/44-340/159-257 // sp|P18939|NU4M_CHICK/NADH-ubiquinone oxidoreductase chain 4 OS=Gallus gallus GN=MT-ND4 PE=3 SV=1/2e-14/44-340/159-257 // sp|P03905|NU4M_HUMAN/NADH-ubiquinone oxidoreductase chain 4 OS=Homo sapiens GN=MT-ND4 PE=1 SV=1/2e-11/44-340/159-257 // sp|P03905|NU4M_HUMAN/NADH-ubiquinone oxidoreductase chain 4 OS=Homo sapiens GN=MT-ND4 PE=1 SV=1/2e-11/1-42/145-158 // sp|P48916|NU4M_FELCA/NADH-ubiquinone oxidoreductase chain 4 OS=Felis catus GN=MT-ND4 PE=3 SV=1/2e-11/44-340/159-257 // 
GCTGGCATTTACCTCCTGTTCTACACCCTAGCCAGCTCACTCCCCCCTGCTCATCGCCATCCTCCTCCTACACAACCAAATCGGCACCCTATACTTCCCCATACTCAAACTCTCACACCCCACAGCCTCAAACTCCTGAACAGGCCTAATATCAAGCCTAGCCCTACTAATAGCCTTCATAGTCAAGGCCCCTCTATATGGCCTACACCTATGACTCCCCAAAGCCCACGTAGAAGCCCCAATTGCCGGCTCCATACTACTAGCCGCATTACTTCTAAAACTAGGCGGGTACGGAATCATACGAATCACCCTCCTAGTAAACCCATCAACAAACAACCTCCA
>Bg-c19351 len=665 count=4 IPR:/ blastx_SP:/
TGCAAATTAAATATATGAAATAACATCTTTAGTATTGTGTAGACCAGGGATGAAAGGATAGTAGCTATTCAGTTAAGTGTCATGCTGATTTGTTACTCTTGACTATTGGCTTATATTTGTGTGGGTTCTGTCAGGATCCCAGCTGTCAACATAATGTCTTGCCAGTCTTTTGTTTTAATAGATTTTTCTGGTAGAAGGTTTCTTTGTTTGTTTTGTTTTCTTTTTAGTATACTGGTCATATGACACTCATGTAGACTTATAGACAGAGCTACAGGTCACTTTGATCTCACGCCTCATTGTTTTTGGTGTCATTGAGAACAAAGTTGACAGAGCTTGAACTATTGTGTTGAATAATTCTGATGTTAATAAGCAAACATGTGTATACCTTTTATTTAACAAAATAATTTATTATTTATTAAAAGTTTTTTTAATGAGAATCATGTTGCACTGAATTAATAAGGATGCCATTTGATAACAAGAAAAAGGGACATAACAATGTTCTTTTTTTGTTACAAACTGTTATGTCATTGTTTCTTGAGCTTGGGAGACTTCAGCTTTAATTCATTGAATAAGTTTTTTGCATTGTATTTTTGTATGTAATGAAAATTTCATATTTTTGTAATGCTTGAAACAAATATCTCCACTAAATTCATGCTATTATAATT
>Bg-c13637 len=420 count=7 IPR:/ blastx_SP:/
TACTTTTCAAATAGATGTATTTTTATAGATACATTTGAATTTGTCAACACTGATGGAGAGTGGAGATAGCGTCTAGCGGACCTATGCGTCAAGTTTCGAATTGACATTGTTACAGTTTCAGCCAAAAAGGTCTAGATCATTTCAAACAAAAGTTGTAACCCCCTCCCCCTTCCCCATCGCTCTAGTGTTGCGTCTTTTTAATGCAATAATAATTCTGATTATATAATCCTGTTGTAGGCAACAGGCAAGAGCTCATTTTTCTTAGTTGGATCGCTGTGACTACTTTTGATATCATTGAAAATAAGATTCATATCAGGATCAACCTTAGGTATAGGCAAAATAGCTAGGGCCTCAGATTGGTGGGCACCTCGAAAAGGGCTCATAACAATTTTATTTAGAGAAGAAACTGAGACACGCAAA
>Bg-c16213 len=249 count=6 IPR:/ blastx_SP:/
GTTGGGAAGAGGTTTTCATCGTTCAATCACCAGATTCCTTGACAGATTCCTTGACAGATTTCTTGACAGATTCCTTGACAGATTCCTTGACAGATTCCTTGACAGATTCCTTGACATATTTCTTGACAGATTCCTTGACAGATTCCTTGACAGATTCCTTGACAGATTCCTTGACAACGATCCACTGATCTATTTTCTTCACGATTAATTTTTTTAGATCAGATAAAACCATTTACGCTTGACACTGGT
>Bg-c40284 len=242 count=2 IPR:/ blastx_SP:/
AAGACGGCCAAGGTCACGGTCAACCACATGGCCATCATGTGACAGGTGATTATAAACAAGGTGTGGAACATCAGAAAGTAGACCCATTGTTTACTGTGACAGTGTGGTTTGGTGAAGATCGGCCTAGAAAGAACAAAGAAACTCAGATTAATTTAAGTTTATTCATATTTAATAAACAATCAAGTTGTTTGGTATTCAATACATACGAGTAATCGACGTCTCATTGAAATATAACTTTATTC
>Bg-c20950 len=343 count=4 IPR:/ blastx_SP:sp|Q3TVW5|TCHP_MOUSE/Trichoplein keratin filament-binding protein OS=Mus musculus GN=Tchp PE=1 SV=2/3e-06/126-236/219-255 // sp|A0AUT1|TCHP_XENLA/Trichoplein keratin filament-binding protein OS=Xenopus laevis GN=tchp PE=2 SV=1/6e-05/123-236/220-257 // sp|Q5RE49|TCHP_PONAB/Trichoplein keratin filament-binding protein OS=Pongo abelii GN=TCHP PE=2 SV=1/8e-05/126-236/220-256 // sp|Q9BT92|TCHP_HUMAN/Trichoplein keratin filament-binding protein OS=Homo sapiens GN=TCHP PE=1 SV=1/8e-05/126-236/220-256 // sp|Q1RM03|TCHP_DANRE/Trichoplein keratin filament-binding protein OS=Danio rerio GN=tchp PE=2 SV=1/2e-04/126-236/221-257 // 
AGTCAGCACGGCAAGAGAAAATAGCATTTGAACACCAGATGGAAGAAGAGAGATTGGCAGCCTTGGAGTTGGAGAGAAGGAAAGAAAGGGAGAGGCTGAAAGAAGAGCAAGCCTTAAAAGAAATTCTCAGGGAACAGATGATGGAGTTCAAGAGAAGAGAAGCAGAGGCCAAAGCGTGGAAGCAGCAGCAAGAAGAACTGATGAGACAAAAGTGGGAGCTGGAGAGAATAGAAGAGTATCAAAGAAAGAGAAGAAAAAGAAAAAAAAAAAAAAAAAAAATACGGAATAGACGTTAGTACCAACCTACGTCGTCTCGTAGAACGAACCGACCGAACAAACGGGG
>Bg-c20158 len=434 count=4 IPR:/ blastx_SP:/
TAAATGTGGTAAAATTGTTATGTTTATTTTTTTTTTAAACGTCAAATGGTAAAGCATATGTCAATAAAATTGGTTTACCTGTGGTGATTTTTTTAAAATTGATTTTGACTAGCGTGACTTGTGTGCTATAGCATGCTCGGTATGGTCAGCTCTAATCTCTTTAGTGGACCTGTGGGGGAAGGGAGAGGTTTACTTGTAGCACAACACAGCTGGAGTCAAATTATTTTTTTTTTTATTCTTGTCTGCATCAGGATTCAAACCGAAGCTCACTGGGTAGCCAGTCGGTAGGGTCATGCCACTAAGCCACATAACCCACATTGTAAGAGTTAAACTGAAAACTCTGGGGTTGAAATTAGTCCCCTTTAAGAGAAAAATTAATACGAAAACTGTTTTTTCTATTACTTTTTGCTAGAAAATCAACTCCTATAACTTTA
>Bg-c17688 len=365 count=5 IPR:/ blastx_SP:/
TAGGCTAATAAATACATACTAAGTAGAAAAATGTAAAGTACAAAAATAATACCAATTTTGTTTGCACTTGAATAATAGATTTAATTGAAGATAAAAAGCATGACAATAGGTGCAATTTTACAGGTCTCCTATAGCATAATTTCTAAATATCTAAACAACAGTACTGACACAATGGCTACAGTTTAAAAAAAAACAAAAACATTGTGATCACAACACTTAAAACAAGCCAAGTGCCACTTTCAGTACTATGTTTTTAACTACTGAAACCAAAAAATAGTTTTTTTTCCGTTTATCTTTAAAACTTACTTTTTCTATCAGAAGAAAAACGGAAACATTATGCTAAATTGATATAGAAAAAAATTTAG
>Bg-c12066 len=558 count=8 IPR:/ blastx_SP:sp|Q7Z0T3|TEMPT_APLCA/Temptin OS=Aplysia californica PE=1 SV=1/4e-23/3-257/33-117 // 
ATGGAGACTCTGTGCCACATCCCTGCAAGCCCAACACTTTTTGGGCAGGAGTGGGGCATCAGAACGACCAGGGCTCAGGAGAAAGAAATGCTTTTGGACGAGATTTTGAGAGAGAAGGCAAAATTTGGACGCAATCACTATGTCATTTAGATTCTGACGGAGATGGACTAACCAACGGTCAAGAGCTAGGTGACCCAGAGTGCGTGTGGACTCCAAACACCTTGCCCAGTAGACAAGTTGGTCTGAGCAATCCAGGCATCTGCGACCCTTGGGACTCTCCTGTCTGTTTCTCAAACAACATTACAAGCGTTAAATACCAGACTCAAGAGGAATGGATGCGAGACATGTGTAGGGCTGGTCCATTGCTTTGCCCAGGCCTGAACGAGTCGGACGTACATCAGGTGGCGCTGACTATTGACAACGGAACTCAGGTTCCTTTCATGGACAAAACGTATTACTGCCAGATATTTGACTTGGAAAGTCATATGACCATTCCTGGAAACTATCAGATAATTGCCGTGGAACCGGTCATTGACAATGCCCAAGTCCTTCATCATA
>Bg-c30558 len=500 count=2 IPR:/ blastx_SP:/
GCACGAGGCCTCGTGCCGAATTCGGCACGAGGGGATGCCATTGCAACTAGAATAGCAGTTGAAATAATTATTTTAGAAACTTTATTTTTATTCAAAATTTTAATTACTATTTTTTTTAAATGCTCCACCACATAAATAATTGGTTTGAAAGTATAAATGAGATACATATACTGCAATCTTTAGTTTTTATATTATTGACATTTGTTTGCTATTGAAAAAAATTATCAATGTTTACCATTCCACCTTTTTGAAGGTCTAAATAGTTTTTGTTTTAATGCATTATGGACCATCTGTTTGTTTACCTCTGTATGCTTCAGTTTTGTTTTTCTCTATGCTATGAATATGTATATTTCTGTTTGTAATCATTCCTTCAAACATATGCATTTACTCTCTGTTGTATTTTAATAAAGATTGCTAAAGCAGAAATTGTGTTATACCGATAGTTACTTTTATTTATACATAAATTGGTTAATTTACAGTGGCGTCATTATGGTGGGTGT
>Bg-c34549 len=300 count=2 IPR:IPR000994:Peptidase M24, structural domain blastx_SP:sp|Q6P1B1|XPP1_MOUSE/Xaa-Pro aminopeptidase 1 OS=Mus musculus GN=Xpnpep1 PE=2 SV=1/2e-28/2-229/485-559 // sp|Q6P1B1|XPP1_MOUSE/Xaa-Pro aminopeptidase 1 OS=Mus musculus GN=Xpnpep1 PE=2 SV=1/2e-28/225-293/558-580 // sp|O54975|XPP1_RAT/Xaa-Pro aminopeptidase 1 OS=Rattus norvegicus GN=Xpnpep1 PE=1 SV=1/3e-28/2-229/485-559 // sp|O54975|XPP1_RAT/Xaa-Pro aminopeptidase 1 OS=Rattus norvegicus GN=Xpnpep1 PE=1 SV=1/3e-28/225-293/558-580 // sp|Q9NQW7|XPP1_HUMAN/Xaa-Pro aminopeptidase 1 OS=Homo sapiens GN=XPNPEP1 PE=1 SV=3/1e-27/2-229/485-559 // 
ACATGGAACTGGACATGGCGTTGGAGCTTTTCTCAATGTTCATGAAGGTCCTTGTGGAATCTCATTCAGAATATCTCTCACTGAAGCAAATCTTGAAGAAAACATGGTCTTATCTGATGAGCCAGGTTATTATGAAGATGGAAAGTTTGGTATTAGAATTGAAAACTGTGTGAAAGTGGTGAAGGCAGAAACTAAACACAATTTTGGTGGTAAAGGTTATTTGTAGTTTTGAACCAATTACCCTTGTACCCATTCAAACTAAAATGNTTGATGCATCTCTACTAACAGAAAGGAGATAGA
>Bg-c10598 len=290 count=10 IPR:/ blastx_SP:/
CATCTGTTTACACAATTTATTTTGAATTCCTGAAACATTTTATTGTATAATGACAACATATGTGTTAGGGTGTAACAATAATCAAAGTAGAACCAAGTCTAAGCGACACAAATACAAATTATAAATGATATGAAGCCTATCATTTGATATCAAGTCTATGATAATAATAGCAACAGGAAGGTTTAATGGCAACTAAGAGAAGTCATGCAAATTTGTTTAAAACTAGCAAAAGATTTTGTAACAAATTAACTGGATTCTAATCAATGTTAAGAAGCAAAGTAACATTCTGA
>Bg-c32331 len=368 count=2 IPR:/ blastx_SP:sp|A7MBH5|CC151_BOVIN/Coiled-coil domain-containing protein 151 OS=Bos taurus GN=CCDC151 PE=2 SV=1/2e-09/261-1/314-402 // sp|A7MBH5|CC151_BOVIN/Coiled-coil domain-containing protein 151 OS=Bos taurus GN=CCDC151 PE=2 SV=1/2e-09/343-278/287-308 // sp|A5D8V7|CC151_HUMAN/Coiled-coil domain-containing protein 151 OS=Homo sapiens GN=CCDC151 PE=2 SV=1/1e-07/261-1/314-402 // sp|A5D8V7|CC151_HUMAN/Coiled-coil domain-containing protein 151 OS=Homo sapiens GN=CCDC151 PE=2 SV=1/1e-07/343-278/287-308 // sp|Q8BSN3|CC151_MOUSE/Coiled-coil domain-containing protein 151 OS=Mus musculus GN=Ccdc151 PE=2 SV=1/2e-06/261-1/310-398 // 
CAACCGAGTAATTTGCTTTTCATTTTCCTTCTTAAGATCTTCAAGGTGACGAGTTGTTTCCCCTTGGTTTTCAAATCTGTGAACTACTTCCATAGTATCAGCAACTCCAGTGCATTCTTTAATACGTTTGAAAGCTTCTTCATATGTTGTGATTTTTTGTTGATGATCCTCCCCATTGAGAGCTGCCTTCTCCTGTGGACTGAGGTCATCTTGTTGAACTGATCCACGCTGAGCCAGCCTTTTCTCAATTCTTTCATGCTGCATTTTTTTCTCTTCTGCTTCTTTCTTCATTTTTTTGCAGTTCAATCTCTCTCTTTTTCCTATCAGCATAGACAGTCTCTTCATGTTTCCTCAGATCTTTCAAAGTT
>Bg-c36276 len=277 count=2 IPR:/ blastx_SP:/
CACAAGATTTCAGTTTTTGATTTTATTATCATTTTACCAAAAAATTTCACAAAGCACCAATGCATATTTTTGTTAAATGTCAAAAAAAAAAAAAACTAAACACTTTTACTTTGGTACACCCTGGGCATCCCTATGGTTCTTCATGGCCCAAGCCATCCAGTCCAACTGTTTTAACAAGGTATCTGCTCCCTTATCTAAGTGATCATTCAGAGGTTTGCCATTCTCATCTAGGGCCTTGTGTACTTCAGGAATGCCAAAGNTGTTGGAGACGCTCAGT
>Bg-c3167 len=232 count=41 IPR:/ blastx_SP:/
GTAGCTGTGCGTGGATAGATCTGTGTGAGTAGCTGTGCGAGGATTGATCTGTGTGAGTAGCTGTGCGTGGATAGATCTGTGTGATTAGGTGTGCGCGGATAGATGTGTGTGAGTTGTTGAGGGTGGGTATGTGAAAACAAAAGAGTGAGTGTTTGTGTGTGTCATCAGGTAGCCAAGAAGGAATTCGTAGATGTATCGCAAATCGAACACAGGTAATAAAAAAAATTTAAAA
>Bg-c22034 len=266 count=4 IPR:/ blastx_SP:/
GTGTTCGGCGACATACAAAGTGGGGGGTGGGGTGGAAAGAGGGAGGTTGAACTTTCGCTGGAGTCCTAGATGTTAGATGCAGGATGATCAGAAAACTCTTGAGAAAGATTTATGATTTTTTATTTCCATATGTCCCGTCCTCTAGGGTGGGACAGTTTTATGATAGCATTGAAATGTCCCAAACCCCCAACCAAGGCTTTAGTGAACCTGCAGCTTTATTCTTGCTTGGTTACAGCGATAGTCACATGAAAGACTGTGGACAGCGG
>Bg-c16619 len=639 count=5 IPR:/ blastx_SP:/
CTATTATAAATATGTTTTAATGTGAAAAGGTTATTTAATATTATTACAATGTCTAATTATACCATACACAGCAAAGCTTCATATATAGTTTTATGGTATACAATTGAATCATTATGAGACAAAAAGTAGTCAGAAAATTTGTTACTGTTGTAAAATTATTGGTAAAATAGGAAACACCTTTTATTTCAAAATTCCTTTTCGCTTGCAATTTGCTCTAATATCATTTCAACAGTAAACAATTTATTAAGACAGATCATTTAATGTCAAGAGACAATTAGGTTCACAAACATTAAATAGCATCACTAAAATATGAGTGTATAAGTTACATATTTCAAATGTACAGTGTACAAAAAAAGAATAAACTACAGGCAGAAATAGCACCAGTGTAAATGTGTTTAAACAATAACAACAAGAACGTACTGACAGATCTTCTGAAAAAACTTTTAGACAATTATTGTGCATAGCAATTCATTTCTTAAAAAAAAAAAATAGATACTTATTTTAAACTGCTGAATACGTTTTCTTAAAATGTCTAAAGTTGAGATTAAAAAGACTTGTATATAGAGAAGAAAACATTGCATATATTCAAATAATATCAATGAAATATAATTTAGCCAATGATATAAAAGTTGCACATGC
>Bg-c14518 len=730 count=6 IPR:IPR006052:Tumor Necrosis Factor; IPR008983:Tumour necrosis factor-like blastx_SP:/
TTACTGCTGTAACAATTTACTAACAAAAAGGATCTCTTTTGGAATTACAGCCTGTGCTGTTCTAACAAGTCTTCTGGCCTTCGGCATTAATGTGGGTTTGTGGACGCACAATAAAGAATTCAAAAGGTTAGAGACAGAGGGTGTCCTAGTAATCAATGAGTCTCTAATTTCTCTGTTAATTTATCAGAAAAAACTAATGCAGCTAGCAAGATTAGAAAGACCTAAGAGGATGAAACACACTTCGATCAGTGCTCACAAATCTTTGGCTCTACCTCCAAGAATTCCTGGTTCTGAAGATAAAAAAGACCCGTACTTTAAAAAAGATTACAATGTGTGTGTTCGAATTAACATGAGCCATAATCCCACTGACCATGCTAGAGGTGTTACACTGGACATTGACGGACTGATCATCCAATATTCAGGTCTGTACTTTCTCTACAGCAGTGTAACCTTTAAACCAAACTCTACCGACTTTTCAGCGTCATTCGCATATCAGACCTGGTTCCAGTACATCTATAAAATGCGGCCCAACAGCCCAGCTCATAGTACAGTACTGACTCGAGTTGTCCACACTTGCTGTCTGAATTGTACCAACAGCCAGAACACGGCTTACTCAGGAGGTGCTTTTTACCTGGAGGCTGGAGACATATAAGATGTGTGTCTAACTGGACAAGGTCTTATTACGCCTCCACGGGCACAACGTATCTGGGACTGTTTAACTGACCAGTGG
>Bg-c39266 len=251 count=2 IPR:/ blastx_SP:/
ATGGTGATACAGTTACAGTATTCTTATGAAATTGTTCTGTTTGCAACTGATGAAGTCATGGACTGAATGTTTATTGTAACATAAAGTTTATCCTAAAACTTCTAAGGTAAAAATGAGTGAATGTATGTGTTGGTTATGATTCTCTTTACTTGCAACATTTTTGCTGGAATAATTTATTTACCATATAACCTAAATACAATTTAAATGAAACTAATAAAAAAGAAATAAAAACTTAGAAATACAAAGAAAAA
>Bg-c13529 len=464 count=7 IPR:/ blastx_SP:/
AGCAGTGGTAACAACGCATCCGACGCGGGGTGCTGACTGTGTCATGTTATCTGGAGAGACAGCCAAGGGCAATTTTGTTTGTTATTAGTTTGTAACGCAAGTCTAAAGAAACAATGGGGGAAACTTGGTCAGTCACTGTTGAAGTATTTGTCATGAGCTGACCTAGGACCACCATGTCCTTGCTAACATCTGTTTGACGTTAACTAATCCTTGTTATTTCTTGTTCTCCCAATTCAAATGATGTACATGTTCATGTTTTCTGGCCTGCTAGAGAATGGATATAAAGAGAGCTTGGTGACTTTAGGGACAAAAGATCATTGACTGAATTTCGGAGGTCCTGATTGTTGGTGTTTGTGAAATGGATGATTTCAATTATTATTTTTTATCCTCATATTATTTTGTATCTCTTTGCTTTTATTGAATAACTCTTATTATCTAAAATAAATAAATTATGTGATATTTTT
>Bg-c35726 len=283 count=2 IPR:IPR001998:Xylose isomerase; IPR013022:Xylose isomerase-like, TIM barrel domain blastx_SP:sp|Q2NXR2|XYLA_XANOM/Xylose isomerase OS=Xanthomonas oryzae pv. oryzae (strain MAFF 311018) GN=xylA1 PE=3 SV=1/1e-23/23-280/254-339 // sp|Q3BMF2|XYLA_XANC5/Xylose isomerase OS=Xanthomonas campestris pv. vesicatoria (strain 85-10) GN=xylA1 PE=3 SV=1/1e-23/23-280/254-339 // sp|Q5GUF2|XYLA2_XANOR/Xylose isomerase 2 OS=Xanthomonas oryzae pv. oryzae GN=xylA2 PE=3 SV=2/1e-23/23-280/254-339 // sp|Q8P3H1|XYLA2_XANCP/Xylose isomerase 2 OS=Xanthomonas campestris pv. campestris GN=xylA2 PE=3 SV=1/1e-23/23-280/254-339 // sp|Q4UNZ4|XYLA2_XANC8/Xylose isomerase 2 OS=Xanthomonas campestris pv. campestris (strain 8004) GN=xylA2 PE=3 SV=1/1e-23/23-280/254-339 // 
GTAACAACGCATCCGACGCGGGGATGCAATGACGGTCATTGGTTTCTTGAGAACCTATGGATTAGCTAATGAAATCAAGCTTAATATTGAACCTAATCATACAACATTGGCTGGCCATGCTTATGAACATGACATAGTTATGGCTTCAGCCTTTAATATGCTTGGTTCAGTTGATGCTAATACAGGCTCACCAGATTTAGGCTGGGACACAGATCAGTTTCCAATGGATATAAAAATGCACCCTTCCTTTATGCAGGTCATATTAAAACAAGGTGGTCTACAA
>Bg-c32488 len=361 count=2 IPR:/ blastx_SP:/
TTCCCCAAACAGACTATGCAGTATTTTTTTTTAAAAGTCTTTTTTTTAAAAATTTGTGTGAGGTCCTTACTCTTAAAACAAATTTCTCTTCAATTTGAAAATAGACTCTTGATATTGTCTAGTAAAATAGATACTGAAATTATTAGCCATTTATATCACCTCAATGTATCTACATCTGTGTTCTACTGGTTAATGATATGAATTCAAAATGTTTCTAAATAACTGATAAAAATACTTAATAAACATCTTCAGTGGTTATCTCCATATTTTCTTTAACTTTTAATTTTCGTGTGTACCACTTCTAGACTTTTGTAAAATAAGTAATAAATTTAACAGGACATCTTAGGTTTAATTATAATTA
>Bg-c29478 len=735 count=2 IPR:/ blastx_SP:/
CTGGGACATGTTGTCAATGCTTTAGAATACACAATTCTTTCCTTTTTGGGGGCAATTTTTCTTCATAGAAATTCATTTATTATGATGGTGTAAACATCTGACTGACTGCATTGAGAGACAACAGTAGATTTTTTAAAATGTATAAATGTGTTGTATTGAAGTGTAAGAAAAATTACAATAAACATTTTGGATGTAATTTTTGTGGTCAAGACAATAATGTATTTTGTGGTCAAGACAGTAATGTATTCAAAGTGTAAAATGGATAATGATAATTAAAGCATCTTGGCACTTATTTAGTCCTCTGTTGTAGCAACTAACATCTCAAGTTTTATTTGTGCTTTGTTCACTTATGACCTTCTCAGCGTGTAGATCATCAAACTAATATTTGTTCAGCTCTAATTTGCCTTACATTTTCTTTCAGTTTCATCCACAATCTATCACTTTCTAATCTCATGCAATCCACAGCATTGATTCTAGAGTGTATATTTATTAAATCAGATTTACTTTGAGTGCTACATAACAGAAGTAGGGCGAATTGCTAAGTATCATTGCTCTTTTTATTTCTTATAAATCTTGTGTAGGAGAGGGGCGTAGGGCAATCTCTAGGACATGTTGTTATCCTGGCCATAATTATTTTAATTATTTTAAATGTAAAAATCAGGATGGTTTGGTTAAAAATTGTCAAAAGAGATTAAAAACTAAATCAATAGGTGTTAATTATCTTCTCTTTTGAAA
>Bg-c14655 len=637 count=6 IPR:/ blastx_SP:/
CGTCCGGATCTTGTTATAAAAAGATAATGTTAATATTACAGATATACACTTTGAGTTTAATATGTTGTCAATCACCAATTTTTGGTAACTCTGAATGAGAAATTTAAGAATATTGTTTTCTCCAGATTTAGTCTAGATAAAATAAAAGTTTCTACTAGATCTTGATGTCTATATCTATGTAGCGACTTTACTCTATACTATTGAAAAGTATTACTAACGTAAATATAAGTTACTCATGAAGTGTTTTAGAGAATGAGAGTGCATTTTTCTATAAGAAAGGATGTTGGGATGTCAGCATTAGTTTCCCAGAAGTCATAAACAAAAATTAACAAAAACATTTAGAATGAAAACATTGTATGAATTTCTGTGGTTAATAGTTGTTGTTGTTTTTGTACCATGAGCATTGGATATAAATAAATTCAAATTTTGAAAGCAGGCGTTATGATAAATAAATGAATCTTTTAAATTCCTCTCAAGGTATTCTTTTGTTGAGTAAGTCCTTAGTATCAAATGTGTATTGTGGCAGTATCCTTCTAATAATGAGTATGACTTAAAGGTGGATTATTTTTGCTTGGGTTGAAGGTTTAGTAATCACTTCAGATCCTCTTTCACACAATGCTGTCTATGGGAGCAGATT
>Bg-c27213 len=271 count=3 IPR:/ blastx_SP:/
GTTAACAATTTTATTACTATTAGATTGATCATTATTAATGTAATAATGAACAGAGAATATCATTCTTATGAAAAATAATACAATGGTGAACTATGTAATTCAATCATCAAAATATAGTTAAGTGTTTTCATTTACAATCTTTGAAAATTAATAATAGAGACAGACTGCTCATTAATATAATGAAATTTTTATGTTGGTACAGGCTTATCATAAAAAAAAATAATGGAGAGAAATGGTGGCAAGTAAATGTTGGGTTTTAATACTAATAAGG
>Bg-c41580 len=223 count=2 IPR:/ blastx_SP:/
TTTTTATGAGTAATTATAAGCCTATATATTTCATATTCCCGTGACAGGCATCAGAAAAACATTCTCGTGCTTTTGACAAACTTCATCTCACGCGCCAACTGAGCAAGTGCGCATGTGTAGCAGCAGAGACTCAGACATCCGGAAGTAATGGAAGCATCGTAGCTCAGACTTCCCTGTATGTTCTCTCTTCCCTCAAAGAAAACTCGGAGACCAAAGTGCGAGG
>Bg-c7476 len=484 count=15 IPR:IPR002172:Low density lipoprotein-receptor, class A (cysteine-rich) repeat blastx_SP:sp|P98092|HMCT_BOMMO/Hemocytin OS=Bombyx mori PE=2 SV=1/6e-09/85-324/1335-1418 // sp|P98166|VLDLR_RAT/Very low-density lipoprotein receptor OS=Rattus norvegicus GN=Vldlr PE=2 SV=1/1e-07/106-330/245-314 // sp|P35953|VLDLR_RABIT/Very low-density lipoprotein receptor OS=Oryctolagus cuniculus GN=VLDLR PE=2 SV=1/1e-07/106-330/245-314 // sp|P98156|VLDLR_MOUSE/Very low-density lipoprotein receptor OS=Mus musculus GN=Vldlr PE=1 SV=1/1e-07/106-330/245-314 // sp|P98156|VLDLR_MOUSE/Very low-density lipoprotein receptor OS=Mus musculus GN=Vldlr PE=1 SV=1/8e-04/97-384/29-129 // 
AAGTGGCTACTATTGCAATGGTGTTGCCTGTGTACTCAAGAAAGAACTGCCCTTGTGTGATTGATGGACAAAATAGTCAAGTCTGGTTCTGTCATTCGAAATTCACAATGTGACACATGTCAGTGCATTGGAGGAGAAACTGTCTGTGTGCCAAAGTTCTGTCCAGCATGTCATATTGGAGCATCTTATGTGGACAAGAAAACTTGCAACTGCCAGTGTGAGACTTGTAGTCCTGATGAACACCAATGTGGCACAGGCTTATGTATTCAAGCCAACAGAAAGTGTGATGGCATCATTGATTGTGTTGATGATGAAGACACGTGTGTGAATCATCTTCAAGTCAAAACATCTTTGAAAGGTGTAAAGAACACGTAATGTGACCCACAAGGATCATACCTGGACTTTCTACTGTAGATAATTTTGTTTGTTCATGATTTTGTTTTGTTATCTCAAATAATAAAATTAGTCTTTGTTTGTTTAATTA
>Bg-c38998 len=253 count=2 IPR:/ blastx_SP:/
TTATATTAATTTATTCTGCATGCATTTTATAATAACAACTAAACTAAACTCGGTCACTACTCATAAGTTGACTGAATTAAGTTCTCAACAAAGAGCAGCATTACATACAAAGAGAGATTCGATGTTATGTTCAATCTTCTAGAGTTTTCTTGAAAAAATGATTAGAAAAAATAAATAATCATGAAAAAGACAAAAATTTTGTCTAAGAAAAATATTTGAAATGTCACACATCATTGCTGTGAGATTTCACAAA
>Bg-c31895 len=387 count=2 IPR:/ blastx_SP:/
TGTGCACAAGGTAAATCAATTAGATATTTAACAAGATATTGTAATTAAGTAATTTTTTAATTGGTTTTTTTTTTTTTATTACATCTACTAGGCTTAAAATAGAAGTCCTTTTTTTTTTTATTTATTACATAATCAACTTTCATTTTTAGGACATTTTCATACGTAAGATTGCTATATGCCCTGAGATTTTTATTTGCGCCATTTTTCTATTTAAAAAAACACGTTTGTGTGCATGTTTCAATTGACCTCTCGTATAAGATCCAGTCCCCTTTTTGCTTCTGCTGTTGATGCATTGTTTACACCAGTCATTATCATATTTGTTGTATGTGATAGAAACTAATTGTTATTTTAATGTTTATACTAAACGTAAAAAGGGAAATCCAATTA
>Bg-c15401 len=385 count=6 IPR:/ blastx_SP:/
CAATGTTAGGACCAGGCGGGGCACCAATGTTTAACATGTTGCAAAGCTTCACAAAACAAAGTGTATACAATGAGAAGGGAGCATATAAGGGGAGATGATCAAGGACATCCAATGAACTTACAAACCTGTCCCCTTGCAATTTTGTTCTAATGTCAGGATCTCTAGACAAGTGTAGTATGTCATATATAGTTAAGAAATTGTTTATCAGTAAGTAGCGAGCGATATCATCATGCCCAGAAAGCAAAGCCACACTCAGAGGCGAAATGACTGTGCCATGGTGGTTGAATTTAAAACAGGTTTCACTACACTTTCTGTCACGAGGGGGACATCCATTTAAAAATCATGTATCTCACCACCTTACGCTCACCTCTTGCAATTATCTCAT
>Bg-c9078 len=367 count=12 IPR:/ blastx_SP:/
GTCACGAGAGCAACCGGTTAGGTCTGGCGTGGCAATCAGGGGCTTGATGTCCATCATATTGAAGTCAAAAGACAGAAGAGTTGAGCCTCCCTGCTTGAAGTCATTGTGATGTTTAAATTGTAACCATCAATCAAGGAATATACGGTGTCCACAAAGTATCACAGGGGAAATACACTGAAACGAAGATAATGAATTCACCTCCACAGTTTGTAGGCCTTCCAAGAAGTTGCTGTATTTTTATATTTCCTATCACAAGTAAATTTTTTTTTTGTTGCGAAATTAAACTTTCTGATGTGACAGTCCGAATGTCAGTTTGAAATTGACACTATTTATATATAGATCTATGTATATATAAATATACTGTCTG
>Bg-c17403 len=410 count=5 IPR:IPR003841:Na/Pi-cotransporter blastx_SP:sp|Q5REV9|NPT2B_PONAB/Sodium-dependent phosphate transport protein 2B OS=Pongo abelii GN=SLC34A2 PE=2 SV=1/1e-28/9-386/134-259 // sp|O95436|NPT2B_HUMAN/Sodium-dependent phosphate transport protein 2B OS=Homo sapiens GN=SLC34A2 PE=1 SV=2/1e-28/9-386/135-260 // sp|O97704|NPT2A_SHEEP/Sodium-dependent phosphate transport protein 2A OS=Ovis aries GN=SLC34A1 PE=2 SV=1/5e-28/3-356/136-253 // sp|Q06495|NPT2A_HUMAN/Sodium-dependent phosphate transport protein 2A OS=Homo sapiens GN=SLC34A1 PE=1 SV=1/5e-28/3-356/136-253 // sp|Q9JJ09|NPT2B_RAT/Sodium-dependent phosphate transport protein 2B OS=Rattus norvegicus GN=Slc34a2 PE=2 SV=1/7e-28/9-386/135-260 // 
GGCTAATGAGCAGCTCTGTGCTGCTGAGTAACCCTGTCGCTGGATTGATGGTCGGGGTTCTGGCGACGGTGCTGGTTCAAAGCTCCTCTACGTCCACCTCCATCATTGTCTCCATGGTGTCAGCTGATATTCTACAGGTCAAACTGGCCATCCCGATAGCCATGGGCTGTAACATCGGAACGACGGTCACCAACACGCTTGTCTCCCTTGGACAGCTCAACGTGAAGAGTGACTTCAGGAGGGCGTTTGCAGCCGCCACCGTTCACGACATGTTCAACTGGCTGTGTGTCCTAGTGTTGCTGCCCCTGGAGGTGGCCTCGGACTACCTCTTCCTGCTTACTGAGGCCATCACTAGTGAACTTGCTCACAATCGCTCAGACACGGCCTCCAACACCAACCCTAAGATGTTG
>Bg-c32106 len=377 count=2 IPR:/ blastx_SP:/
ACATTGTATGAATTCATGTAGTCAGGGATAAGGTAGATACATTGAATCTTAATGTTTGTTTATTTTATTATTTTTTAATTAAGCATAGACTTTTATAATGTACTCACAAATCACCAAGTCATGGTTATGATTGGGTTTATTTCAAAGTCTTAAGGGGTCTAAGCAGATAGACATTCTGTATAGGATGTATACTTGAAGATTATCATGATATGGTTGGGGTTGAATGCTGTGTGTCCTAAATCTGACCTACATGAATCACCACTTTGAAGGAGGACAACCTGTACCAAGGGAAATAAAGAATTCACTTCTACTACTTTCACATTGATAACGACAATTGGTGTCAAAGTTGAATCCGACCATTAGATGTCACTGTTGAA
>Bg-c7684 len=1046 count=14 IPR:/ blastx_SP:/
GTCCAGTGCCATGACTGTTGACTATGAATGTCTCATTGCATTATTTGAACTGGGGGGGGTGACATTGGCAAGTGTTAGAACAGAATGTGTCAGTTAAAGTTGTATATTTGATAGAGAGGAAGCAGATGTCATATTTATTGTCTTTCTTAGTTTATATTGTTTTATATTTATAGAATTTAATCTGAAACTGAAACTAACAGTTGAAAGTTCACCTTCTCATTACATTGTACTTGTCTTTGTGATCTTAAATGTAATACATTGTGGCCCCACATATTTAATGAAAGCATACTTGAGCAGGTCTGGATTTAAGAATGCTGAGGCCTTGTGCATATTAGCTAGCAGTCTCCAATATTTCAAATTAGGGTTTTGACCTATAGCTACTGGTTTTTGTAGTGATGTGACCTTACTCTGCAAGATATAGAGTGTATCCATGGCTGCGTGTTAATCTGAGATGCTGAAATATGTTGAGGTAAATAAACAGATCATTGCTAAGCATCAGTGTACGTATGACCATTAAAACAATGGTAATAAGTTAATATTTAGCTATAATCAAACTGCTGTCAAATATTTTTGGTCTTTGCTAACATTTTTGTTCTTCAAATATGCAAAATATTTCTTGTCCCACTACAAATATTTTTTACAATACCTCTATTCTGCTCAAACCTTAAACCTAAAACTTTGGTGGACACAGATCTTGAAAAGGGTTCTAACAATTTTCCTAGAAATTGACAGTTGGTTTATATCGTTGGGAAAAGAATTACTAGCACTTTGGCTTCACTGGGAAAATTAGTTAGACCGTTATAATTTTTTAAAGTATATAAAGAAATTAATTTAAATTTGGCTAGGGAACTAGAAAATGTTTATCTTCATCATAGTTATACGTTTTTAGGTATTTCCATATGTTGGCCCTATTCGTTCAAGAGTGGAATACATTAATTTTCAGAAACATTTTGTAAGTAAACAAAAATGAAATTTTTTTCTGACTTTCTCCCTAAAGGGCTGATATAACTTGTAACATCAAAGTATGATGATGACCCAAAAAGACG
>Bg-c3426 len=767 count=37 IPR:IPR002110:Ankyrin repeat; IPR020683:Ankyrin repeat-containing domain blastx_SP:sp|Q8IWZ3|ANKH1_HUMAN/Ankyrin repeat and KH domain-containing protein 1 OS=Homo sapiens GN=ANKHD1 PE=1 SV=1/1e-103/29-766/1213-1458 // sp|Q8IWZ3|ANKH1_HUMAN/Ankyrin repeat and KH domain-containing protein 1 OS=Homo sapiens GN=ANKHD1 PE=1 SV=1/1e-30/35-577/1046-1227 // sp|Q8IWZ3|ANKH1_HUMAN/Ankyrin repeat and KH domain-containing protein 1 OS=Homo sapiens GN=ANKHD1 PE=1 SV=1/3e-24/29-532/1111-1279 // sp|Q8IWZ3|ANKH1_HUMAN/Ankyrin repeat and KH domain-containing protein 1 OS=Homo sapiens GN=ANKHD1 PE=1 SV=1/6e-24/26-559/294-469 // sp|Q8IWZ3|ANKH1_HUMAN/Ankyrin repeat and KH domain-containing protein 1 OS=Homo sapiens GN=ANKHD1 PE=1 SV=1/7e-20/29-532/428-623 // 
CGGCAGCAGTCAAACTGTTACTAGATATGGAAGTGATATCAATGCTCAGATTGAGACCAACAGAAATACTGCCCTAACTTTGGCCTGTTTTCAAGGACGCCATGAAGTGGTTAGCTTACTGGTTGACCGCAAGGCTAACATTGAGCATAGAGCCAAGACTGGCCTTACACCATTGATGGAAGCTGCCTCAGGTGGCTATGTTGAAGTTGGTCGAGTTCTCCTAGATAAAGGAGCAGATGTAAATGCTCCACCAGTACCATCTTCACGAGACACAGCACTTACAATAGCTGCAGACAAGGGACATTATCGTTTTGTAGAACTACTATTACACAGGGGTGCTTCTGTTGATGTCAAGAACAAAAAAGGCAACTCCCCACTTTGGCTGGCTTGTAATGGTGGTCATAGTGATGTGGTGTCTTTACTGGTGCAAGCAGAGGCTGACATTGACAGTCAGGACAACAGGAAAGTGTCTTGTCTAATGGCTGCATTTAGAAGAGGTCACATCAAGGTGGTCAAGTGGATGGTAAAGAAAGTAACACAATTCCCTTCTGAAAATGAAATCAAGAGATATATTGCCACTATAACAGATAAGGAACTTCAAAAGAAATGTAATCAGTGTGCAGAAATCATAGTGACTGCCAAGGAGAGACAAGCTGCTGAGGCTAATAAGAATGCTGCGAGTATGTTGGAAGAAATCAAGCAAGAGAAAGAACAAGCCCCAAAGGGAGCAGAATTAGCCAGTAAACGAAGAGAGAGAAGAAGAGAAA
>Bg-c40494 len=240 count=2 IPR:/ blastx_SP:/
CAAAGTGATAATATTATGGGAGATATTTCTGACTCTCTGCCATCACATATGTCATTGTTATCTTTAAATGGGTCATCACATACTCTGCAAGGTTCAAGGACACCCGACTGTAGTTCTGATACTTTGTATATGATTGATGTACAAAACATAGGCTGCAAGACACGTTTGGAGCTCTCCAGGCTATTAGACCCTCCTCAGTTTGAAAAAGATGTCGTTGCCCTTGCCAATCACCTAGGTTTT
>Bg-c28694 len=233 count=3 IPR:/ blastx_SP:/
GGGACTCGATGGTATAGTGTGCGGGTAGCCTAGAAATGTGTGGGACTCGATGGTGTTGTGTGTGGGATGCCTAGAAATGCGTTGGGCTCGATGCTGTTGTGTGCGGGGTGCCTAGAAATGTGTGGGACTCGATGGTGTCGTGTGATATTCGCTGATATCGTGTGATGGTTGCCCGAAATTGTGTGGACTTATCTGGTGCTGAGTGGGACTGCCAGAAGATGAGGTCACAGAAA
>Bg-c35727 len=283 count=2 IPR:/ blastx_SP:/
TTACATGTGGGTGCTGCTTATATTCGATAGTTTGGTACATCCGATTACTGATTACTATATGTATTACATGTTCATACATTCTATTGATGTATTTAAGAAAAATTGGTTTGTATTCAAGTGCCTTTATATTTACCCAGTAAAACTATCCTTGTGTTATATACATTTAAGCATACTTATTTTTTGTTTAATTTTTTATTTGACTCTATTCTTTGTGATTCAGTTTTTTAAATATGCTGAAATTTCATTATTTTTCAAAGGCCAACGGATAGTGTGTAATTTTTTT
>Bg-c19138 len=773 count=4 IPR:IPR001092:Helix-loop-helix DNA-binding domain blastx_SP:sp|P13903|TWIST_XENLA/Twist-related protein OS=Xenopus laevis GN=twist1 PE=2 SV=1/3e-11/548-772/38-113 // sp|P97831|TWST2_RAT/Twist-related protein 2 OS=Rattus norvegicus GN=Twist2 PE=2 SV=2/2e-10/491-772/10-107 // sp|Q9D030|TWST2_MOUSE/Twist-related protein 2 OS=Mus musculus GN=Twist2 PE=1 SV=1/2e-10/491-772/10-107 // sp|Q8WVJ9|TWST2_HUMAN/Twist-related protein 2 OS=Homo sapiens GN=TWIST2 PE=1 SV=1/2e-10/491-772/10-107 // sp|O96642|TWIST_BRABE/Twist-related protein OS=Branchiostoma belcheri GN=TWIST PE=2 SV=1/2e-09/485-772/33-136 // 
CACCCAGGATTATTATCTACAACACTTTCAACAGACCCTTAGAGCTTTGGTGATGTGTGTTTTTTATTAAAGAAGGATTATCAGTGCGATATTGGTTGTTATCGACAGTGTATTGTGCTTATTTATTGTCAATCAAAGAATACTTAATCGGATAATAGTGTCAGTTCTGTCAAGCAGATTAATAAGTGGTTGATTCTACAACATCTTTGCCCATGCTATGGAAGGGTACCGGGGTGGTTTGAGCAGAATACCTCTGCTGCTCTCGAAGGATACTCCGAGTCACGTGGTAAAATCCGAGGCCGAGTTTCAGGCAGCCATGGACAGCCTTGGCCGGCCAGGCGAACACATGAGCGCCTACCATCACAGCAACAATCTCCCCATGGGCGCTGCTCACGGAAGTGGAGTCATGTCCAGCACTAACCAGACAGCCCTCAGCAGACTAAGTCCTTGTAGTGATAGCAGCTCTCTTGCAGAGTTCCCCCGATCCTGTTCCTCTGAAGACAGTTGCGGTGACACAAGCAGAGCCGAACACTTCATGGAACACAGATCTCGCAAGCGGCCTTACAAACAAGACTCATCCCCCAGCAGCGAGGCAGCTCGTAAAGCCAGACGGAAAAGCCCGCAATCTCCCGAGGAGCTTCAGGCACAGCGCTGCTTGGCCAACGTCCGAGAACGCCAAAGGACTGAGTCTCTGAACGAGGCGTTCTCTCAGCTGAGGAAGATCACTCCCAACCCTGCCCTCAGACAAGCTGAGACAAAACTCCAAACGCTAG
>Bg-c8858 len=571 count=12 IPR:/ blastx_SP:/
ATGGACCAAAGGCTTCTTGATTGATGTTCCTTGGGCTATTGCTAGGCCATTGAAGATTTCAAAATCCTTCCCTGTGTACCTTATCATCAATTGCAAATAGTTGATGCTAACACTCATAACTTTATGACACAGCTAGTATTCTTAAGTTTAATGAGTATGCAAGTCTACACTTTATCCTTGACATGAATTTGTAGTCCCTGGAAACAACTTCCTCTGCAATCTTTTAGCTTAGCTATTTCATTCCAGTGAAGTTCTGGTGTCTAAGTTCTATGTCACTTCTCTAAATACTTACACAAAGCAAGGTTTTTATAGTTCAATTATACTCAATTTGATAGTTTGTTTGTTTTTTAAACTGAATTTTAAGAGTTTACTTTTGTAAGTGATGTATTATGTACTTTTCACCTTGTATTCTAGGTTGTAAAATTTATTTTGCATATTAGCAAACCAACAAGTTAAAACATTCATCTTAATCAGTTCATTGGACTTGTGCTGATTGTGAGAGTGCAACTAGTGATGGAAGCTGTTTAATGATTGACCTTTCAACTCTCAACACTAATAAAAGATGCTTAAT
>Bg-c1146 len=1568 count=113 IPR:IPR002557:Chitin binding protein, peritrophin-A; IPR011583:Chitinase II; IPR001223:Glycoside hydrolase, family 18, catalytic domain; IPR013781:Glycoside hydrolase, subgroup, catalytic core blastx_SP:sp|Q91XA9|CHIA_MOUSE/Acidic mammalian chitinase OS=Mus musculus GN=Chia PE=1 SV=2/5e-89/15-1247/84-473 // sp|Q95M17|CHIA_BOVIN/Acidic mammalian chitinase OS=Bos taurus GN=CHIA PE=1 SV=1/2e-86/15-1244/84-471 // sp|Q6RY07|CHIA_RAT/Acidic mammalian chitinase OS=Rattus norvegicus GN=Chia PE=2 SV=1/7e-86/15-1247/84-473 // sp|Q9BZP6|CHIA_HUMAN/Acidic mammalian chitinase OS=Homo sapiens GN=CHIA PE=1 SV=1/3e-83/15-1244/84-475 // sp|Q13231|CHIT1_HUMAN/Chitotriosidase-1 OS=Homo sapiens GN=CHIT1 PE=1 SV=1/6e-80/15-929/84-386 // 
AGAGCCATAAATCTAAAACAAAGGAATCCCATTTAAAAGTCCTGCTGGCTATCGGAGGATACAACATGGCGTCCGACCCGTTTACGTCATTCGTGTCTGACAGTGGCAAGCGAGCAGAGTTTGTGAAAAATGCAATCGAGTTTCTGCGGAAACGTAACTTTGATGGGCTTGACATGGACTGGGAATATCCAGGTCAGAGAGGAAGCCCACCTGAAGACAGGGAACACCTGGTCTTGCTTATGCAGGAACTCTGGAACGGTTTTCACCACGAAGCTTACGTCTCCAACAAACCACGTCTGCTGCTGACTGCCGCATTTCCTGCGGGGAAAGAGAGCATTGATATTGGCTATGACATTCAAGGAGTGATCAATTTTATTGACTTCATCAACATTATGACCTACGACTTCCACGGGAGCTGGGAATCTTACACTGGACTTAACGGACCTTTGTTCGCCCACCCCAGTGACCACGGCAACGACAGCTACTTAAACTTGGACTGGGCTGTCCAGTACTACATTCAACTAAATGCGCCTAAAGATAAACTCAACGTTGGCATATCTACGTATGGACGCTCTTTCCTGCTGGACAGTGCTTCAAACAATGGCATCAGGGCTCCAGCGGCACAGCCAGGCGATGCTGGGAAATACACAGCAGAAAAAGGTTTTGTCTCTTATTATGAAGTGTGTGAGCTGATCAAAGCAGGGGCTCAGGTCGTCGACGTGCCTACACAACGTGGACGTTACCTGGTCAAGGACAGACAATGGATAGGTTACGATGACATACAGACAGTCACGGAGAAGGCTTGCTATACCAGACAGCACGGGTTTGGGGGAGTAATGTTTTGGGCGGTGGACCTTGATGACTTCTTGGGACAGACATGTGGACAAGGGAAGTATCCACTGATGACCGCTGTCCTGAGAGAGATGACTAGTACCTCATTCGCCAACTGTCCTGTGAAATGGTCTGATCAGACCACTTTGACCACAAGACAATCCACAGCTTCCACAACGCAGACAACGTCGTCGCCCACCACTCAGAGCACAACTGTCAGCACACAAAGAACCACAACAACAGAAATGACCACAAATGCAGTCCAGTTCGATTGCAGCGACAAACCAAATGGTGTGATGTTCCCTAATCCGGACTCTCCCTCCACATTTTATGAGTGTTATAACCAGGTTGCTCATTTAGCCTACTGTGTTGTAGGTCTCCAGTTTGATCCAGCTCTAAATAGATGTGATTTCCCACCTACCACGACAGTTAAACCTAATGAAAGACCAGATTCCACTGAGCAGCCAACTACAACTGAACCAGTACCAACAAGTGCTGTGTTTGACTGCACAAATAAACAGAACGACTTCTACCCAGATCCTGCATCGTGCAATAGTTTCTACGTTTGTCTAAATCAGGCTGGTATGAAAGTTGACTGTAGTTTTGGAATGAATTACAACCCTCAGATAAAGACTTGTGACTACACCAGTGATTGCCATCACGTAGTTTAAGTGTTCTTACAGTTTTCTTAATATTATATTTATCTAATAAACTATTTTATTATTTAAAGAAGTAAA
>Bg-c29884 len=652 count=2 IPR:/ blastx_SP:/
TTGTTTCCCCACCATGATTGCTGTCTACTTCTTTCTTTCTCTCTGGTGGACAGAACTACTCAGTGTCTGAGACATTCTACATGTTAACAGCAATGCTGTTGAATATTTTTTTTTTTTAAATTGTTAGTTCATTTATGTTTGGGGTCAAGCATGGCATTTTGCAGTGAGCACATTGGACATTATTTGTTGGAAGTAGCTATACATTTTATAAACTTCTTCTTGTACATCTTTATGTATTTTTTTTTATGGCTTGACTTGTCCATCTTTAGTCTGTGCACACATAGTGACAAAGTTTGTCTCATTTGTTTTAGATTTTTTTTTTTTTTTTTTTGAAATAACTTGTGTTAATGGCATAAATCTGGAAACTTAATACACCAACAACAAAAACTAAAGACAATTACATTCCTTATTCAAGTGATGACAATGTTTCAGTAGTATATCATTCATTTAATTACTAATAGTTAAAAATACTTATGTTGGCTTTTTCAAAATCTAGATTTAATATATGGTTTTAACTTTATTGTTGTTAAATTTCAATTCTGTTAAAGAAACAAAAAATCTGGGTTTACTTTAAGCATCATATTTTGGGGAACTTAAAAATAATCTGTTTTTCAAAAGACTGTTTGACATATAGGTATAGACAGTAGCTTGA
>Bg-c17239 len=438 count=5 IPR:/ blastx_SP:/
TGCTGACATATTTTAATTAATTGGATTAAAATAAAGTTAAGAAAACAAATTTAATTTAATAAATAGCACAATAATAATAATAACATCTGTTTTTATTTAACAATCTAAAAAAGAATGTGTTGAAACTGACACACCAATAGCTCCAGCCTTGAGTGGTTAAGCACTTGGCTTCTGAATCTGGGCTCCTGGATTGGAATCTCGGTAAGGAATAAGATTTTTCAGGTGCACCCAAGTCCACCCAACTCTACTGAGTACCTGACTTTAGTTCGGGAAAGTAAAGGAGGTTGGTCATTTTTCATTATGCTGGCCACATGACACCCTACTCATTAACCATAAGCCATAGAAACAGATGACCTTAACATCATTTATGGAAAGTAAAAAAAGACAAAAGAAAAAACAACATCTAGACTCTATATATGCAAATTATGCCAATATAAT
>Bg-c40891 len=235 count=2 IPR:/ blastx_SP:/
TTTTTTTCTATTTTTGAGAAATATAAATAGTTCAAGGCGTCTAAACGCATGTTCAGAGTGTAATCTCTTAGTTTTTCTGGTTTGGTGTGAATCACCAAAATGAAATTATCAGGTTTTTGTTTTCTCAGGTTATTAATCAAAGAGGCCTCTCCATCAAACATCGACATCCCACTTTTACAGAAAGACTTGGTGACATACAAGAAAACAAAAAGTAGCCTTTTGGAGAGGAAGCTCT
>Bg-c5434 len=489 count=22 IPR:/ blastx_SP:/
CTATGTTGAGCAAAGGTGATGTCCCCAAGGATGTCAGCGTTTCTGACTGGTGTCCTACAGGAGACTTGGCTCAAGTTCAACGTACCGTCTGCTCTGCTCCTGGTGAGGCCTGGGCTACATGCTTGCTTTTGTCATGCAGCCTTGTGAGCAGTTTGACACTCACCAAGACTTGGCTGCAGACTTTAAAAGGAAATCGTTGGGGACCCCGGGGCCCAGGTTGGAGTCTTGAGTTGAACTCTTGAAAGGACGCTAAGAGTTTACGAGGCTTACGAATACATGTACTAACTATTTAATAGCAGACTCTGAGGAAGTTCAAAAGGATGAATTAATAAATTTATATTAATTAAAAAAGGAAACACTCAGAGGAAGAAGATGAAGTCATTGCAGAGAAAAAGTCAAAAAAGAAGAAAGTAGAATCAAAGGAAGTTCAACCTGAAGAGAATGGTGAAAATGGTGAAGTAGAATCTGAACAAGAAACAACTCAAGTGG
>Bg-c2335 len=666 count=57 IPR:IPR020090:Midkine heparin-binding growth factor, C-terminal blastx_SP:/
TGGTGATTTATTTCCCCTTCTCTTCCCTTTCTCTCTCTCTTTCGCTCCAAACTTCTCTTTTGCCTGTGTATAAGTTCTAACTGCCAGAGATAGTTTCATTAATTACCTTTCTTGGGTTTCATTTTTTTATTTTTGTCACAGTTCTTCATTTTGGTCTGAGTTGGTTCACAGTCTGCACTAGAGCCTTCTTTTAAGGGCATTGTCTTCTTCTTCTTGCCAGTAGCCTCGTCACATTCACTCCAACTGGCATTCTGCTTGTCATACTTGCAATCTTTATTGCCCTTGGACTTCTTGGTGCCGTTGCAGGGTTTTTGTTTGACCATTGTGGGAGCGCAGGTGTTGGCATCACCTTTCTTCAAGGTGAAAGTGATGGCCTTGGTGTTTGTTGTTGTATCACATTCTGCCTCTTTGCCTTTCTTGTTGTACTTGCAAACAGATTTATTAGCTCCCCTCTTCCTTGCCTCTGTGCAGACCAATACAGCCAGCACCACAGACACCAACAACAGCATACTCCAGTTCTGGGACAACTTCATCTTTGTCTTTTATCCCAGTAATATCCCACACAAAGAAAAGCCTATCCCTATTGCAACTTATTCCTCAACTAATAACCTGAAGATATCGCGTTCTATTATTTATGAAGTCTTTTTTTTCTACCTACGAGTAAGG
>Bg-c4463 len=1354 count=27 IPR:IPR006634:TRAM/LAG1/CLN8 homology domain blastx_SP:sp|Q6AYM9|CLN8_RAT/Protein CLN8 OS=Rattus norvegicus GN=Cln8 PE=2 SV=1/3e-36/1058-417/53-267 // sp|Q6AYM9|CLN8_RAT/Protein CLN8 OS=Rattus norvegicus GN=Cln8 PE=2 SV=1/3e-36/1179-1111/12-34 // sp|Q9QUK3|CLN8_MOUSE/Protein CLN8 OS=Mus musculus GN=Cln8 PE=2 SV=1/2e-35/1058-417/53-267 // sp|Q9QUK3|CLN8_MOUSE/Protein CLN8 OS=Mus musculus GN=Cln8 PE=2 SV=1/2e-35/1188-1111/9-34 // sp|Q5JZQ7|CLN8_CANFA/Protein CLN8 OS=Canis familiaris GN=CLN8 PE=1 SV=1/3e-35/1058-417/53-267 // 
ACTCCTAAAATAAATACACAATCTAGAACAAATATAATAACTTCACAATCGGATAATTTCTCCAAATTAACTTCCTTTGTAAATGTAGACTGTCTGTACGGAAACGGGATACAAACAAATTTTTAGACAAACTTCCTGGTTTTAAAGCAAAGTTAGTCCCCTTTACTATTTGTAATTAATTCCAATGGTTCCAACATAGCAGATCTAAAAGGCGTAACAAAAACACGGGATCACTCAAATGTTACACTTTTTAGTTTGGCCCATGATATTTTGAACTAGCTTTTTTAAACTTCTAAAGCAGTCCTGAAGAGAACGCAGAAGAAAGTAAATTAAAAATAATCTACGTCTCATTTCCGGGTCACGGAGACTGATTTGTTTCATTCCTTTTTGGATGCTAACTTGTCCGTGTTTTTTGTTTCCTGGAAATTCCAATCCACTGGGTTGAAGAGTTGCTGTGTTTTTTTGTAGCCCCAGTATGGCGTCATCAAGAAGGTGACCAGAACGAGGTTGGCGTAGAGCAGAGTCAAGAGGGGCACTGGCATGTGGTGCCAAATGTTACCCCAGTTCTGATAGGTGACGTACCACAGGTAACATTCCACCACGCTCCTCAGGTGGAAAGTGTGAACCAAAACCATTTGGTTGACGAACCAGGATCTGGAGTTCTCCATGCCGGCTTTCAGGAGGACATAACACAGGCAGGAGAAAGGAGTGCTCATCTCGAGTATCATCCCCTTGCTCCCGTAAGAGTAATTGGCCTCGTAGTAGATGGCCAGGCTGTAGGCCGTCAGGGCCAGGCTGTGATGCATGATTAACATCCTGGAGAATGTCCCGAAAGCGATGTCGGAGATGACAACGGCTAGGATCTCGAAAAGAAAGAAGCCGACCGAAACACACATGGCGAAGGCGCTGGTGGCGGTGGTCCCGAAGACAACATCCCTGTCCAAGTTGGTGGTCTTGAATATGGCGTAGACGCCCACGATTAGACCGAAGAATCCGTACACGCCCCTGACCACAGCCAAACACATGAACACCTTGTGTTTAAACACCAGGGCCCTGAACGCCGGGAAGGCATTGCAAATCGCAAGTGTGATAAGAAATAATGAAGGAGACGAAGGTGAAGGCTGCCAACACGACTTCCGCTTTCACTTTCCTTGAGGTGTAGTCCAGCTTCAGAAGCGATGGACATAAGTATTGTAATATTTCCTCTTCAGGTATGACCCTGGTCACTTCTGCTGAAGACGTATCTATTAAACTACTAGTATTCATAGTGGGCTTCAAGTCACTTGGCGTGAACTAAAAAAAGTAACCAAGATCAATCAGTGCAAGGAAGACACGCTATTATACGTTGTCAACG
>Bg-c40892 len=235 count=2 IPR:/ blastx_SP:/
TTGATTTTAGAATTTTTATTTCATCTGAAAGAAAGGCACAAAAACGAACCCGCTGCGAATAGGCGTGTGGACAATGAATACATGGTCATCGGGATGTGGCGACATGTGGTATGAGTGTGTCGGCAGTTAATATACATCATCATCATCATTATCGTCGTAGAGCTTGCACCGATTGCAGCAGGCTTTGGAGGATCGACGACAGTTTTCTGGCTAGCGAGATCGAGCTGGATGTCGG
>Bg-c13490 len=476 count=7 IPR:/ blastx_SP:/
ATCAAATTTGACACGGTCAATGAGGGTGCGGAAAACAAAGGAAGCTCTCTGGACGGAAACGCCAAGAAAATATTTTTGGAAGAAGACCCAAAAGAGAGCGGCAAACAGGAAGACGTTGAAGACAATGAAGATGAGCGAGGTAATGTCGCCGACGATGATTTTGAGCAGTTAAAAGCCATTAGCTCAAGGCTGCAGAACAACGAAGAAGAGAATTCGCACGCAAGTAAGAAGCTGAACCTTACCGGCAACGAAAGATTCCATCAGTTGGTCAACGATTACAAGATGGAGAAACTCAAGAAGAACGAGGATAGACTAGCGTCACTGGACGATGAAAATAACGATCCGCTAGAGACGGGCAAAACTGGGAGCTCCGGTCGTAGCGGGTCCATCGTTTCTGTTAAAGACAACCTCTCTCACAGAACCACTGCGCGTGGGAAGAGACCCAAGCTGAACAAAGGTTCGACAACAAAATCCTT
>Bg-c10754 len=910 count=9 IPR:/ blastx_SP:/
AATTATCAATTAAAAAAAATTTTTTTTAGCCNNAATATTTCTTTTGATAAAAAAAAGAACACAATACTTTTTGAACATGTTCGTCAAACAACTGCATTTTTTTATAATAAAGAGAACTGGGTAAAACGAACAACTAAAAATAACTGGTCCCCTTTCCCGTTAGATGTTTCCTTGATTCTTACAAAAATCATCATAAACATTATAAAGTCTGCTGTTGTAAAAAAGGTGTCAGTGTGTGTGTGTGTGTATAGTTAAAACAAAGTGTTTGAAGTCTATCTCCTAAGACTTTGTATAGTTCTCATCAACAATACTTAATTGAAAGATTCCAGGGGTGTGGATTAACAATGGACACACATGAGATAACATTGTCATGAACAACACACAAACACACACATCGTCCATCTTACATGTGTGTGTGTGTGTGTGTTAAAACTGAAGTTCCATGGGATTGCTTCATATATATCTCCTGTCATGAATAGAAGAAACTCAAAAGAGATGATGTTGTGGGGTGATTAAATACTTTTTTGAGTCTACTTCATTTGAATAAAATAAACATTCTAGCTCTTGAGATCAATGATAATATGAATAGTTATTAATATGCTTTTTTTTTTTTTTTTTAAACTTTTTGGGAAGGTATGATTAAACAAAAATTGTTGAAATATAACAAATGAAAACCTTACAAAAAATAATTGACTAATGGATCTGTCCATTAAGTCTCAGCACCAGTTTGAAAAAGATTTATTAGATTGTTTACAAAAATAAACAAAAAAAAACTTAGCCTGATTATGACAAAATTTGTTTCAACTGCAGATATAGAAATAACTAGGAATAATAAAAGCATTTGGAAACTAGTCAAGCAGACATAGTGAGAAAGCAAAGCTGGATTACATGAGAGCACTAAAAAGGGAGG
>Bg-c35191 len=290 count=2 IPR:IPR002884:Proprotein convertase, P blastx_SP:sp|Q5REC2|NEC2_PONAB/Neuroendocrine convertase 2 OS=Pongo abelii GN=PCSK2 PE=2 SV=1/2e-25/2-286/513-602 // sp|P16519|NEC2_HUMAN/Neuroendocrine convertase 2 OS=Homo sapiens GN=PCSK2 PE=2 SV=2/2e-25/2-286/513-602 // sp|P28841|NEC2_RAT/Neuroendocrine convertase 2 OS=Rattus norvegicus GN=Pcsk2 PE=1 SV=1/4e-25/2-286/512-601 // sp|P21661|NEC2_MOUSE/Neuroendocrine convertase 2 OS=Mus musculus GN=Pcsk2 PE=2 SV=1/4e-25/2-286/512-601 // sp|Q03333|NEC2_PIG/Neuroendocrine convertase 2 OS=Sus scrofa GN=PCSK2 PE=2 SV=1/2e-24/2-286/513-602 // 
CCTCAGGTCAACATACAGAGGCTGTGTCACCATGTACCTTACTTCTCCCATGGGAACAACATCAATGATCCTTAGCCAAAGGCCTAATGATGATGATGACAAAAATGGTTTCACCCGCTGGCCCTTCATGACCACCCACACATGGGCTGAACTCTCCCATGGAACATGGACGCTGGACATTGTCATGGAGCCAATTATTGGTGTCCAGAGAAGGCCAGAGACTGGTGTCTTTAAAGAATGGACACTTGTATTGCATGGAACAAAGACTGCCCCCTATGCCAACCAGCCAG
>Bg-c36083 len=279 count=2 IPR:/ blastx_SP:/
AAACGTGTTTTGTTTTTCTCGCCATTAAGAAAATTGATCATAATCACCTGTGATAGTTAAATTTAGTCCATCAGAAGAGTTTTCGTCTTTTTCAGATAATGACATCTTTTTACCTAACAAAAATAACACAGTCAGTGGAATAATTGGTATTAATAAAAACATGGGGAGAAACTGTGAGAGTCCAGATGGGCAAATACCAGTGACCTTTTCTTTACAGTCAGTTTTACATTTGAGGTAACAGTCCTCTCGACAGTTGAAGCCGTAACGCATCGGTAGAAA
>Bg-c35897 len=281 count=2 IPR:/ blastx_SP:/
AAACATTCTATTTGTATTTTAGCCACCACAATCTTAAGTCGGTTGGCAACATTGTCGTGATTAACAAGTGCCCGCTTGTTGCATGCTCATGAGTCTTTAATAATAATAATAATATGTAAATGTAGCTCCGGATTTAAGTACCGCTATTGTTATTCATAATAATCATATAAGTCTTTTTAAAGCTTTTCGAAAAAGTGTATGGGACAAAATCCTGCCCCGGGGCCTGGACGCATTTTAATCCGGCCCTGTCTGTTTAAAAAGCCAACGCTTAACGATAGTGT
>Bg-c7997 len=397 count=14 IPR:/ blastx_SP:/
TTGCTCTGTAAACGTGTTCTATTGATAAAAAGGTCACGGCCACATTCAAACCAATAGATCTTTGTGTGTGTGTTATTATTTCATGCAGTACTTCATTATTCTCTCACTATAAGCTGCTCGTTCTCTTTCCACGGATTTACTGTGTACCATTCACTGGTATGTACTGTGTGTCAGTCATTGTGTGCTCCAAGTCGTGTATGTGTCAGATTTGATTGACAGATTTCCATTCAATCACGCCTACCTTTGTGAAACTAACCACTTCCATTCTTTCACTATTGATGTAATTTTTAATACTCAGTGTTTTAATACGGCCCCATCTCCCCACACACACGCCACATATTATTCTATTTGGGTTCTTTTTTTTTCTCTTTTCTTTTGTATGTCTACAATGAATACT
>Bg-c38758 len=255 count=2 IPR:/ blastx_SP:/
ACGGCAGACATAGTGTGTCATGTCGAAGATGCGAAACGCTGTGAGGACGCTTACATGTAAACTAGCACTTTGCCTGGTTTGCTTACATCTGATGGCAGGTGTAAGTGTAGCCGTTCCAGGGCTAGATTTTACTGTACTGCCTCTCACTTCTGATCAAAGAGACAAAGTCTCCATCAGATGCTCAAGTAAAAATCAACAGCCCGAATTATCAAGACTACAGACCCTACAGCCTGACGATATACAAAACCAAGCAGA
>Bg-c24431 len=409 count=3 IPR:/ blastx_SP:sp|Q5ZM57|PTAD1_CHICK/Protein tyrosine phosphatase-like protein PTPLAD1 OS=Gallus gallus GN=PTPLAD1 PE=2 SV=1/2e-05/7-153/307-355 // sp|A7YY55|PTAD1_BOVIN/Protein tyrosine phosphatase-like protein PTPLAD1 OS=Bos taurus GN=PTPLAD1 PE=2 SV=1/5e-04/7-156/307-356 // 
GTGTAGACAGGATTCTTCAGTATTGCACTTCCCAACTCTGTCAACTTTGCATTTTACTTTCCATATTTCCTAATAGTGTATTTAGTGGCTATGTGCTTTGGCTCCAGCAATAATTTAAAGTATTTATACATTCAACGACAAAAGCAGCTTGGCAAGATGAGTCAAGTGAATGGAAAGTCTCCAAAGCAAAAGACTTCATGAATGGAGAACACTCAGCAATTTTTAAGTTATTCCATTTTTTTATTGGTTATAATTTAGCAATAAATCAATTGTATTTTTTTTTCTAAACAAAAGACTGTTTAGTGGTGCTTGCAACAAAGTTCACCACTTTATTACTGTCATTATTTTTTTATTATGCTCATTACAAAAAAGTAATTTTTCTAATTTAAATTAATTGATAAGACTAAAA
>Bg-c6102 len=560 count=19 IPR:/ blastx_SP:/
GCAAAGTGGAGACATTTCGCTGACAGTGACAAGACTCCAAAGGCAAAGCGGAGACATTTCCCTGACAGTGACAAGACTCCAAAGGCAAAGCGGAGACCGTTCCCTGACAGTGACAAGACTCCAAAGGCAAAGTTGTCGACTGGCAGTTGCAAGACTGCAAAGCGATCATCACACGAAAATGACATGACGGCAAGGTCAACTGTTAGGTAGTTATTGGTAATTAGTAAACATGACAATGACAAAATGACAACCACATTAGAACAGGAATTTGACAGAGAGACAGGTCACCCTGACACTGACATTTCACGGAAAGATATTGTGTACCACTGACCAGGACCGGATTTAGATTTTATATGGCCGTAAACTTTTTGAAATATGTTACCTCTTGTAATCAGCTTAAGGTAGTTTATTCCCTTGCTTTTCTTCAAAACAATATTCTGTGTTCCCTAAGCTAAAGCTTAAGCTGCCAATGGAACAACACTAGCCCATGAAAAATCTACCAATTCATTAAGACCAGGAGATGAAATAGAGTGTGGTCCAACACCAGGTGAACAGCTGCT
>Bg-c9009 len=423 count=12 IPR:/ blastx_SP:/
GAACATTTTATTAATTTTCAATCAACAAAACAAGATATTCAATCAGTTCAATCGGCATACATCAAAATAGTGACTTGATTTGTGCTCAATTAACATAATACTAAACATGAACAATTCTAAACATGAACAATTCTATCTTTTCACACTAATTGGCGTCCATGTGAAATATAACTTCTAGTCTAGTAACCACTAATATTTATTTCATGTCTTATACAATGTGTTACCCAAAAACGTCCTGTATATCTCCTTAATGTGGCAGGGTTTCCTGTATATATGTTATAAGATATTATAGTGACATTGATACTAGACCCTTTAACCTTTTTTTAAATCAAAATGTTACACATTTTATAGCACAATGAAAATAAAAATAAATAGGGCAGAACCGGGCATCGAACGTTGCCCGCGTCGGATGCGTTGTTACCA
>Bg-c9384 len=668 count=11 IPR:/ blastx_SP:/
ATTCGTTTCATGAGATAGATCTACATACTTTATTTTAAAATTCCATTTCACCCTTAGGGGGAGTTCCAACTGCATCACACCAAGTCAGGGGCATTAATATGAGGAAAAGTTCAGACTCATCACAATTAGATTTTGAATTATGATTAATCCACATATTTTGTTGATGAAATGTGAAGTAATTACTGCTAAACCAAGGAGTTAGTTAAATGAAGATGCCCCTTCATAATTTGTATATACACCAAGTTTAAGTATAATGTATGTTCACATTTAGTCAGCATTAGACCAAAACGATTATTTATTCAAACATTGCCAAGAGTACTTTAAAATGTTAATGTTACCAGACAACCACCAACACTGCACCAGAATAAACCTTCAAGAGGAAAATATTTTGCTGCACCTGATGATGTAAAATCTAAATTATGTAGTCCTCTGATAACACAGTTGTTGGGTATATTCATTTATTAATGTAGAAAGGTATAACTGACTTCCTGTACAGTTCCCTAGGCCATCTTGACATTATAAATAGAATGCAACTTTATCAGTCTTAAGTATAATGGTAACAACCAATAATATACTTCTGTTTGTTTGATTATAAAGCATACTTTTTTCAATTTACAGCAAAATGCATTTTATTTTTAATAAAACAACAAATAAATGTTTAAACAATT
>Bg-c33385 len=328 count=2 IPR:/ blastx_SP:/
ATTTGATTGTATTAGTTGGTAGACCCTGTCAGAGTCCACCAGTTTAAAAATGGATACCTAACACTAAATATCCTTGTAAAAAAATTTAGGTAATGAATATTTTATTTATAAAAACAGATCTGTAAAGGGATAAGTTATAGTCCAGATACATTGAAAGTAGTATTATTTAAGTCATTTTCTAACATACAGAGTTGAACTGGTATGACTAATTTTTTTTTACTTTTTTATTTTTGTTTAATTTAAAATTAAATTTAAGTCGACNCCAACCTAATTGTTGGTAAGTAATTTAATTAGTTACTAATTATAGTGTACCGGTACGTAATATAAA
>Bg-c647 len=1235 count=170 IPR:/ blastx_SP:/
GTCGGATGATGACGAAGATGACTTTTTAAACAATGGTCAAGAAATAGGCCAAGGTGAACGATCATTTGCACTTCCCCCAGGTTAACATCGTGTTACCGAATCACTTTTGTATAGATCTAACCTGAATGTTTGTAACAAGTACAGACTAAAATAACAGGAACGAAAATGATTGATAACATCTTCTTCTCTTTTCTCTTAAATTTGGTAGCTGCAATCTTTTTCTGTCTTGTACTAGCTCTGGCTTATGTTTGGCTAAATGGACATCTTACTGATGATCCAGATAACACCACAGAGAACTGGTTGAACCATCTACCGGAAACAACAGAAACCAGGAGAGGAACAGAATCATCTTTACCATCAACTTGTGAACATTTGAAGAAAGTACAACTTTGGAATGATACCGCCACGGAGATTTCTGACATGGGTGCTGGACAATATAAGTCAATGAGCTATCATTTTAAACCAATCAATAGTGCCAAAAAAACAAAATACCAATACCAGAATGGAAACTGTAAATTAGTGAATGCCGTCTCTCAAGATGGACAGTTAAGGACAATGAATACAGTCCACCAGGATGAACATCCGGTCACATCTCAAGAAAATGCCAGTTTATGTTTGGGTAAGACATGTGGGGAAATTGGAGAACATTTTATACAAGTCAAAGTTCGAGATGGACGCTGTATAAGTCGTCGTGAGACCGTCACTCATACGTGAGTCTTGAGACACCGCAAGGCCGCTACTTTGATGTCAAGTTCTGTTAGTGTGGTCCTATTAAAATGTCATTATGTAAATGTCAGCCAAATAATTTTGACTGTTAAAAGATTCATTTATAGATGCTACACAATTCCCGAAGGATTAGTTTATAGTTGATATAAAATTCATGAAAGTATCGTTTAGCAATGAAAAAAATTTAGGTGCTCGATTGTTCTATTACATTCAAGTAATGGTTAATTGGACGTTGATTTGATGACAGTACATTTTGTAATAAACATATCGATATAATATATCTATAAATGATATATGTGATAAAGAGCTCAGTTTACTGTGTGATTAACTTGAAAGTGAAAATCTGTATAAAGCTTTTTCTTATAAAAGATCTTTTTTACTAAATTTATCTATATTAGTACCTGTTTATGATTATTTCATCTAAGATTACTTATCATTCAGCTATCGTAGCTTGACTAAATATCAAGTGTTGGCATGTTCCGGTTGGCATCATTAAACAAAGAAACAAA
>Bg-c40572 len=239 count=2 IPR:/ blastx_SP:/
CAAGCTACAGCTAATTGGAGTCTGCATCAAGTAACAAAACCTATATGGAAATGTTCTGGTCCTGGTGCAATGCAAATATTTTGACTCTGGTCAAGTGATAGTCACGTTCGTCTGTCAACTAGTCTGTTGTAGGTTTATTTGTTCATTGCACTACTTAATATTCATCAGCAAGATCTGACTAGCTTCAAGTACTCAGGTCTTGTTTCCACTGAAACAATTTATATATCTACTAGAAATGC
>Bg-c34164 len=308 count=2 IPR:/ blastx_SP:/
CCTGTGGAGGAAGCTCCTTGAAAGATTTCTGTAACGCGGTTATGTCCTCTTGGGCTTTGGAGATGTTCAGAACAATGTTCGGAATCATTTTGTTCTCAATGTCGTCCAAATTGTCGTAGAGCTCTTTCCAATTGACTTTTTCTAATTCTTTGATTCTTGGTTCAAAACTAGCGTTGACGATGAATTCTGTCTTGACGGCCAAATCTTCCACGTTTTTCTCTCGCTCTCTCAGAGATTGCATCTACTCTTCCAAAATATGAATCCTCGTGCTCTCAAGCTCTTCCAGTTTCTCTTTGACCTTCTGGAGT
>Bg-c25412 len=343 count=3 IPR:/ blastx_SP:/
CTTCAACTAACCCACCACCTTACTACACATCACCTAAGTTAGAGGAAGAGCCCAAGCGAAGATTTACTGAGGAAAAGGAGGAAAAATCCCAGAAAATTTGTTGGGCTATCAGCATGGACCTCCACATCTGGTGAACCTGGTTCAGTCTAGTCCACCGCCTCACAGTAATTACCCTCCTATTTCTCAAGGCATGGTCCCACCTCCAGGCACTCAAATCATACAGTCCCAGCATGGTCTGATTCATGTGAGTCATCTGGCTCCAGGCCAAGGTCAGTTTGCTCAACTTGGGGCCCAGACTATCATTCAGTCTGCCCCTCAGATGTATCAGACTACCCCAGATGGA
>Bg-c4427 len=433 count=28 IPR:/ blastx_SP:/
AGTCTAGACTCTATAATAATAAACACTGGCTATGGTTGATCTAGATCTAATCTTAATCTAGACTATTTATTTTAGACTAGAGTCGCTAGATCTAGATTATTATAGATCGGTAATCTATAGATCTAGATTCTAGATCTACTAGAGATTTTTTTTTATAGATTTACTATAGACTTAATAGACTATAGACATAGTCGTCATAGTTATGATAGTAGTGATACTGATAGAGTCATAGATCTAGACCAATCTAGTCAATCAGTAGATCTAGAATAGATGCGTAATTTACTAATTATAAGGTTCCATCACTGATGAATTAAAAACGTATTCCTCTGTGACAAATCAAAGAAAAAAAACATGTGGCTAAAACTGTGTTTGCTCCTCCTGTACATTTTCCTGCTGTTTATTATATCCCGGTCGGATGCGTTGTTACCACTGC
>Bg-c39136 len=252 count=2 IPR:/ blastx_SP:/
AGGAGAGATGTCAAGGGTAGGTCAGGCTTAGTGTAGCTCTGGGTCCGGGGTTGATAGGATTACAGGAGGAATTTCCCCCACTTGGGTTCTGTTAGAGAAAGTTATTCAAGTCATGTGTGCACTACAAATGTTGAAGTTGTGTTGTAGAGTGATCTGTTCAAAATAAAGATTAGATCTGTTGTCAAGCTCATGTTATGAATTTAATGTATGCTTGTCAAGTATCTTAATTCTTATTACTAATGAGAAGGCTCA
>Bg-c2019 len=2796 count=66 IPR:/ blastx_SP:sp|Q54YM7|TM2D2_DICDI/TM2 domain-containing protein DDB_G0278163 OS=Dictyostelium discoideum GN=DDB_G0278163 PE=3 SV=1/2e-08/2117-1941/53-111 // sp|Q9GPR3|TM2D1_DICDI/TM2 domain-containing protein DDB_G0277895 OS=Dictyostelium discoideum GN=DDB_G0277895 PE=3 SV=1/8e-06/2120-1938/3-63 // sp|Q9GPR3|TM2D1_DICDI/TM2 domain-containing protein DDB_G0277895 OS=Dictyostelium discoideum GN=DDB_G0277895 PE=3 SV=1/7e-05/2342-2142/1-66 // sp|Q9GPR3|TM2D1_DICDI/TM2 domain-containing protein DDB_G0277895 OS=Dictyostelium discoideum GN=DDB_G0277895 PE=3 SV=1/1e-04/1913-1731/2-61 // sp|Q9GPR3|TM2D1_DICDI/TM2 domain-containing protein DDB_G0277895 OS=Dictyostelium discoideum GN=DDB_G0277895 PE=3 SV=1/1e-04/1679-1545/10-54 // 
CGTTTCTTGTATTGAATGTTTTATTTATTTATGCACATACTCTTTTTTCTATTAGGGGGTCATGAATAATGTCACCAAAATATGAACTACCTTTAAAATACCAACATGGTATACAACACATTCATGGCATAAATCTAAACATATGTCACTAAGACTACTTTTTTTTTTAAATAACATAATTTATAATAACAATTTGTGAGACACTTTAAAATAATAAAAAAAACAATGACAATGACTAACCTCACAAAAGTGTTAAAAAGTCAACAATAAATTTGGATACTTGTAATCATTGCAATACAGTCATCATAAGATTTTATTCTTTTCTAAAGAGATGAATGATGATGCTAAATGTCAAGAGTAAAAACTATTTACAATACAAACACTAAGTAATCATGTGACTGGAAATAATGACATCATGTTTCCTATTATTTTCACATTTTGTCCGCTCAGGCTGATTAATATATCGACTTTATGTTGTACGAATTGATCTAGATAGTTATTGATAATGGTCACTCAACCTGAATAGGCTACTGAGAAAATTTCTTTTTGTCCCTTATTTGGTTATTCTGCTCAAATGGTTAAAAATAACAATAAAAAAAAAAATAAAGAAAAAAAAACAAACAACATTAACTCTATCACTATAGTTGTTATATTACTAATTGTAAAATTTCTTTTAAAATTTGCATGAATGGAGAATGTAAATTGAAATACAATTTAAAAAAAAATGGGAAGATTTAATTTTTATAATTTAGCTTGATAAGGCTCTCATAGAAATCCAGATTTAGTTCACAGACCTATATATAATCTAGATATACATCTTTATTAAATTTACCTCCCTGCTATCAAATCTCCATGGTCTTTCGTTTGTAGCACATAGAAATTAAGCTTACTTAGATGGTAACAGGGATCTCTTAATACAGTGTCCTTGACATGTTTTGTTTTTAATCGCAACATTTAATCATAAAAATCCCCAATTTGGCTTTTGTTTTCTTGTTTTTTTCTAGCCAAAGATTTTCCCTCGAAAACATCTACGTGAAACAGGTGTGGCAGAAACTTCGAAATTAACATCTTAATGAATCAAGATATTGATTTTTAATGTTCTTTTTCCCGTTAATTTGATGAATACGCTCATTGGACTGATACATTTGAACTGACATGTTTGTCAGAAAGTAGTGAGGCCTACTAAACCATGTGCTTCTCTGGTAACGGACCTGCTTCCTGTTCTCTGGGGGAGTATGGTGGCGGCTGGGGCTCGGCCATGATGACTGTGCTTGGCGGCTGCCCTGGGGCGAAGTAGTCGTAGCCCGCTGTTTGGTAGGACTGGGCGGCAGCGCCCTGACCATAATTGTAAGCCACTGGATAGTTAGGATATAGTCCTGTCTGTGGTCCCGAAGGTGGGTACTCTCCTGGGTTGACCATACCGTAATTTTGATAACCTCGTGTAACTGTAACGTGGGCCACTGTCTGCCTGTTTGCTTCCTCGATCTGACTTGCAATAATGCGGCGCCTTTCTTCATTCTCCTTGTTCACTTCCTTGACCAAAAAGGTATCCTACACATGTCTACGATCCAGCCTATGCCAAACAGACCCAGAGTGAAGAAATACAGCAGCCCCCAGAGAGGTCGCCCCAGATAGAAATGGTGGAAGCCGAGGAGACCAAAAACGAACCACAGGATATACGCGTCGTCTGCAGTCTTTACGTTTGGCGGCCTCTTACCTCGTACTTCTTCGTTGACTCTGGTCACGATGTAGGGCATTCTGATCCAGTCAAATACCCAGCCAACACCCACCATACCTAAGGTCAGTGTGTAGAGCACGCCCCACAGAGGGCGGTTGAGATAGTAGTGATGCCCTCCCAGGATTCCGATAGGAGTCAAGCCCAAAGCGTAAGCTGGACAGAGGTTCTTCTCGTTTCTCTCTATGGGACTGGACATTATTCTCTCGTTGGCAGCTTTGACTAGCCAGGGCATGCGAATGAAGTCGACCAGCCAGCCTATGCCGAAGATTCCTCCGCTAAATGTGTACACGGCTCCCCAAGCGTAGTTTCCCAGGTAGTAATGGTGGAACCCGAGAAGGCCGAAAGGAAACCACAAAGTGTAAGCGTCGGAAAGATTCTTTTTCTCCTCCACTTTGGGGTCCGTGCTCTGAAGCTTTTCGTTGGCGTCTTTGACAAGGAAAGGAACCCTGAACCAGTCGACGAGATAGCCGCATCCCGCTAATCCTAGGGTGAAAAAGTAGACGATGCCCCAGTAGTATCTCCTGAGGTAAAAGTGATGAGCTCCTAGGATTCCAAAAGGCGAAACTGCCAGCAAGTAGGCTTCCAGGACCGACTTGCGAGGTAAAAGAGCCAACCTGGTTGCTGCGGTATGGTAGCTTCTGTCCAACGAGGCTGTGTAGGGCGGGGTGTTGGCTGTGTTGATGAAATATGGGCTGCTGTTCGTCGGTGGGCCAAAGTTTGAAGGTTGAGGAGGGGGGATATAGCCTTGGACTGGAGCTGCTGGGCCACATTGGGCCTGAGATAAAGGTACGCCATTTTGAGGTACAAAGGAGACACCTGGCGAAACATTTTGACTAGGACCTGAATAACTTGGAGGTTCTTTTTTGGAAAGAGACGTTTTCATTTTCTCTAATATTTCCTTCTAAAAAAGTCTTTTTTTTCTTCTTTCTTTTTTAAATTTTCTAGTTTCTTTTAATCAATAACAAATTTCACTTTATATAAAGTACTGCACTAGGTCCAAAGGTACATTGTAAATAGATGCTTGAATCTAAAAACAACTTCCTCGTCGTTTCGTTCCT
>Bg-c41992 len=207 count=2 IPR:/ blastx_SP:/
AGGGACCTTAGATGATGCCGAAGCATAGATTAAATTGTAATCTGGTCTATTATTGAGATCTATGTTTAAATCTACAGTTATATAGATCTAAGAGCATTATGATCATAGTACATCAGAGTGCATTATAGTGCATCATAGTGCATTATAGTGCATTATAGTACATCAGAGTGCATTATAGCACATCACAGTACATCAGAGTGCATTATA
>Bg-c14778 len=563 count=6 IPR:/ blastx_SP:sp|Q17FR9|SRRT_AEDAE/Serrate RNA effector molecule homolog OS=Aedes aegypti GN=Ars2 PE=3 SV=1/1e-57/516-10/509-675 // sp|Q5TUF1|SRRT_ANOGA/Serrate RNA effector molecule homolog OS=Anopheles gambiae GN=Ars2 PE=3 SV=3/8e-55/516-10/518-697 // sp|Q66I22|SRRT_DANRE/Serrate RNA effector molecule homolog OS=Danio rerio GN=srrt PE=2 SV=1/2e-53/516-10/446-609 // sp|B4LIK8|SRRT_DROVI/Serrate RNA effector molecule homolog OS=Drosophila virilis GN=Ars2 PE=3 SV=1/8e-53/516-10/523-706 // sp|B4KLY7|SRRT_DROMO/Serrate RNA effector molecule homolog OS=Drosophila mojavensis GN=Ars2 PE=3 SV=1/8e-53/516-10/541-724 // 
ATCTTGTTCACCAATAAGCTCATCTTCCTCAAAGCTTCCTTCTTCCACAAGATAATCTGTGATATTTTTTAACACTGGATTTTTGGACAAAAAACCAAACTGCTGCTCTTTCTCTTCATCTTTTTCTCTTCACCAACATCCTCCCAGAGTTTGCATTTTTTTGTCCAGGATCTGTATAATCTTAGCAGCCATCTTGATATCATTTCTCATGACTTGTTTATGAACAGTTATTCCATTCGCAGCACGAATTCTCTGCTTGAGTTCTCTATTTAAAGTAGCCCCAAGCTCACAATCCCTTAGCCTGATATTGTTCAGACTCCAGCAGACATCTTTGATATTGCAGTCCTTAGAGAAAGTTACCCAACCTCTTCGGAAAAAACGTCTTTCAGGTTGAGGATCTTGTAGGGCAACTCTTTTGAATCCAGGGTAACGCCTACACATAGCTTCCACTTCTTGTTTGGTTATTGTAGGGGCTAAATTACGAAGGAAGATAGAGTTAGTTTTGTGCAGAGGTCTGGGCTTGGGTTCAGCTATTTCTTCCTTTNTTTTCTCAGCAGTCTTTT
>Bg-c13729 len=390 count=7 IPR:/ blastx_SP:/
ACAAACTGTAATAAGAAATGTTGGAAATTCTGCAGTGAAGAGTTGCAGTTCATAGTCATTGGCAATAATTGACAGATTGATTGGTTGAATTCTACCAGAGTGGGGTGCACCACTGAGCTACTGTCCTAGGCAATGAGTCTTTAATGTGGCGATTGTTAACTGTTAACAGGGTGTGATAGAAGTATCTTGGGTTAAGATGCTCTACTGCATTCATGTATTGTGGTGATCAATTGTGATATTTTAGCTCTATTGTAATTTTTTTCTATTCTATTTTAATAAATGTTTTATATTTTTTAAAATAAAAAAGCCTTGGACAATTGTATAAAATTTGGTAATTTTTCCTTTGTTTTGCTAGTTGATACATTATACATGTAGATAATCACATACATA
>Bg-c9157 len=286 count=12 IPR:/ blastx_SP:/
TGTGCTATTGTCCGAATAATCTACGAGTCTGAAACCCGCGTGTACAATGGAAACCCTTGCCAAATCACTTTAAGCGAGTTATCGAATCTACTAGCCAAATTCTTCCCCTATAATTCGCTTCATAGACCAATAATAATTATTTCAAGGTGGAGATTACATATTATTCCTGGTTAGGAACTATATCACCTACTCATTAGTATGCCGTGTGCCCAAAATTAGAAACCGTAATAACTATTTTTATTTTGTTTTCCTACCCTACTCGCCTACCACCCTACTTCGCTCACTA
>Bg-c41111 len=232 count=2 IPR:/ blastx_SP:/
AGATACAGAAGAGCCTCCCTCCTTTTGCTGATTTTAAGTTCCCAGAAGCTACTAAAGGGATGGATTTATCAGGCTCTGACAGGAAACAAATGACAGAAGAGAGTTTGCCATCTTGTGACAGAGAGGCCATCATCTTCAGAACAGATGAACCGCTCTCCCATGAAAGCTCACAAGTGGACTCTATTCCTGAAGTACTTTTTTGAAATCACTCAGTCAGATAGTTAAAATTCTT
>Bg-c6010 len=896 count=19 IPR:IPR001304:C-type lectin; IPR016186:C-type lectin-like blastx_SP:sp|A7X3Z7|LEC2_LIOPO/Lectoxin-Lio2 OS=Liophis poecilogyrus PE=2 SV=1/3e-06/877-413/4-162 // sp|A7X3Z4|LEC1_LIOPO/Lectoxin-Lio1 OS=Liophis poecilogyrus PE=2 SV=1/9e-06/877-413/4-162 // sp|P07439|LEC3_MEGRO/Lectin BRA-3 OS=Megabalanus rosa PE=1 SV=2/1e-05/874-440/6-153 // sp|O54707|KLRD1_MOUSE/Natural killer cells antigen CD94 OS=Mus musculus GN=Klrd1 PE=2 SV=1/2e-05/778-449/72-174 // sp|P02707|LECH_CHICK/Hepatic lectin OS=Gallus gallus PE=1 SV=1/3e-05/784-440/90-204 // 
CTTCTCTCCTTCTTTCTCTTCTTTTCTTTCTTCTCTCCTTCTCTCTCTCTCTCTCTTTCTATCTCTCCCATTCGCTCTCTTATTTTCTCTCTCTACATATATTATATGTATTGTTTCACTATCATGCCAACATCACAATAAAAATAAATAATGACTCTGTTGCTCTAAAGGTTAGATTTCATATCTGAGATGTCCAAGAAGCCAACCATGTTCTGACCTCAGCATTAGAAGAAAAATAAAGTCTTGGGTTTCTCTTCTGCCTTTTGAAAGGAAGGGGTTTCAGATAGTATCTATGTCTTCCTAAGCCACTTCAGTGTAGATGTTTCTTACTTTGATCCAATCTAACGTTAGTAGTTTCATCTTGTTTTGTTGATTTGATTTAAGGTGTTGACAGTATTGAGTTACTTGCTAGGGTCGAACTATCACTCGCATTCTGAGCTGGCCACATGCAGATGTAATGAAGTTGGTTAGTACAGGTACTTCCTCCTACGCTGACTGTTGAGTTCATGTACATCACCACCGCGCAGTCGTGAGGTGTACTTGGTGCTTGACCTAAGACCCACAAGTCCTTCAGTAAGCTGGTGGCCGATTCGTTATTACTTGCCCAAAGCCAAACGCCATCTTTGTCTACGTCATTAGCGCCAATCCAAAAGGGCATTGCTATATCCAGGTCCTGAAGTTGTGTCTTTGAGTCAAAGGTTGCAAGAGATCCATTCAGTAGTTGACACGCCTCCTTGGCTGTTTCCCAGTTCATGACCTCCGTATACTTCTTATAACATCTTCCTGTGGACACTATGGAGCCGTCCATTGAACATGTGACTTTCTGTCCAACCAGAGCATGTTCTTGTAGAATTAACTGTAAAGCAACGATCACAAAAATACTGGAATCCATTGAA
>Bg-c35728 len=283 count=2 IPR:/ blastx_SP:/
CTGTCAAGGAGTAGAGAAGTAATACAAGGAATTCATCCCTAGTTCTTAAATCTTGTTTAAAAAATGTGCGTTATTGATTGTGTACAATGAGCATTATTGGTTGCGTACAATGTGCACTATTTGTATATATAATATATATATTTAACTTCATTTTGGCAGGTATCAAAACTGTACAAATACACTTCGATGTAAAAAGTTAAATATATGTTTTCGTTTTATTCAAAAAAGAAAAAAGCTACATAAAAAAAGAAAAGTAATGACACTAACAAGGTTACTTTTTAGT
>Bg-c12573 len=351 count=8 IPR:/ blastx_SP:/
ATGATGGCTCAGTTGATGATGAGTGGTTGGGACCAGGGTTTGAGCCATTCTCCTGATTGCCAGATCTTGCTGCAAATCATAAAAAGTGCATTTGTCAACTCCGGCCGATTTTCCCTTAAGGACGCCACTGAGTCCACTTAGCTCTCTCTAATGTACACCTGACATCTGTTGTGCTGGCAACATGACATATTTTAACCTTGGGCTACAGAAACAAATGACCATTTTATCATCAGCCTCATTATGCGCAATATCTAAGAGTGGTTACTTTTTTTTTGTTTGTAATACTAAATAAGTTTAGTTTAATTTAAACTTAAACTTTAAAATTTAAACGTACCCCCGTAAAAACCCTTT
>Bg-c36375 len=276 count=2 IPR:/ blastx_SP:/
TTATAATTTCTCGATATTATTGCAACCTGCGCATAGAACGCTCACATAATATTTTGGCCGAACATCCCCCCCCCGGCCATCTTAAAGCTCCGCTAACATGATTTTGTCTCATGATACGTATGTTACGTTGAATAAGAACGGCCTCCACACTTCTGGCCTGAGAATGTCAGTTTCACTTTTCAAACTTTCCTTTGTCACCACCACTAAAAAACATAATTTTACATGAAATCACTGAATTGTACTTTTCTTTTCTTTGGCTTCTTAATTCAAAGTATT
>Bg-c2265 len=666 count=59 IPR:/ blastx_SP:/
GCAGTGGTAACAACGCATCCGACGCGGGATTCATAACTAGAAAAACAACTTGTTGACATAATTTGTAATATTCTTGATATCCTAAAATTGAAGTGTCCAACTGGTCATGTAGGACGTGAGGTTGAGTAAGATATGCAACCAAGACTGGTCCGAAACGGAACTTTGACTTCCACCGTGGATCAGTCTCCTCCTGTACACTCGCAGGTCAGAGCGGAATGGCCAAACGTTAGTGATCCGACAGGGGGCATCAGCAGCGGCGAAGAATTACCTGACCAACTCGGCGTGCCTAACGAAAGTGGCTCCTCCACGCACAGGGGATCGCTGAGATCTCTGCGGCCCACCAAGTTCTCGCCGCCCCCAAAGCCGCCGACCTTCATCTCCAACAATGTACTCCTGACGATTCTGTTTATTTTCGTCGTGATTCTGGTCATAGCTCTCATAGTGACATTCACCGTGTTTGAATGACAGATACAACAGAAACAGTGACAACAAGACGAACTCTGTAGACATGCTAGTTCGAAAGATCTAAATTACGAAACATAAAAAAAAAATGATGAGAGTGTCACCTAATGTAATCGTTCGTTATGTCTAAATGACCGCACACATAAAGGATTTAAAAAATAAAGTCTATTTGGTAAATAAAATAATGAGATAAACATTGTTTTA
>Bg-c38999 len=253 count=2 IPR:/ blastx_SP:/
GATGTCATCTGTTTATTGATTAACAATAACAATCACCATCTCCAAAAGTATCTTCTAAATCAATGATATACTCTTCAAAATAAATAACAAGAACAGTTTACATATCAGAGAATCTCCATGACTATAAATGTACACAAATTCTTTCAAATCATAGTACCAGTACTAAAATTAAGATAATTTAATTTCACAGCTTGAATAAAAAAGAAAATAACTTTAAAAAAAAAAAAAGATATAACAAATGGCATGTGCAAAT
>Bg-c43006 len=118 count=2 IPR:/ blastx_SP:/
GTGGTAACAACGCATCCGACGCGGGGACATGAAAAATGTTGACGGAAGTAACGCAATATGCAATTACATTTGAAGCTTTTGGTATTGAATTTATGTATGTGTCAATTATTTGTTTAAG
>Bg-c5418 len=549 count=22 IPR:/ blastx_SP:/
AGTTTCGTTTTGAATTTTTTATTTTTATCGATTGAATTTTTGAAAGTTTTTAGTTGAGAAAACAAGAAATTCGCGAAAATCAATTAAAATCATTTACAAATGTGAAAATCAAGATGAAAAAGAAAAACTGTGGAGCATTTTATCGAACGCTTCGAAACGGCTGTTGATCAATCGAATTGACATCACCTTTTAGATTCCTAACCGATGTTCGTCCCGAAAAAATTCATTTGATATCATCGATTGACTCGACAGCGAAGTCGTTTAACGACGACCCCAAAGTCCACCTAAACCCCAACCCCAACCTAAGCCGAAACCGAAGCCCCAAGGCATGATTGGAAGAATTGGAGCGATGATGGGCGGAATAATTGGTGCAACGAGTGGTAAGAAAGCTTCCGAACCCAAAGATAAAAGTCCGAAAACTAAAATTGTTGCGATTAAGAATTTAGCGAACATTGTCGAATTGAAAATTGTCGAAGAAAATCAAATTGAAATATGAATTGAAGATGAATCGAAGCGATGTCCCCGCGTCGGATGCGTTGTTACCACTGC
>Bg-c1538 len=649 count=88 IPR:/ blastx_SP:/
TTTAGTTAGAAAGTAGATAAATGTATATCTTGGATAATCTAGATCAAACGGTTTTCAGTTTGAGATCCTACTCTACTACAAGTTGCGTATACATGTAGATCTAGATCTTTTTTGAATACTAAAGGACCTTGATAAAAGAAACAACACAATGATGTCTTTGCTTTGTAAGAATATCAGCCAAACGTCACAGTCTGAAAATCGTTGTCCTACGCTATACAGTAAATAGGAGAGGTATAAACTAAGCGGCTGTATTGTTCTTTCTGCGCTTTTGTTTTCCTTCATTTTCTTTGGGACAGATCTCTACCTCTATGCAAACAGAAGGAGAAACTCAAGAGACACTAGTCGAAGACGTCAATATTTGTCTATGCAAAAAGTTAGTACCTTCATTGTAAGCCATGATGGCAGAGAGCATCCGATGTATCACAAACCTTTCTCCCGCTTGACCACAGGTTACCCAAGTACCAAATCGAAAATTAAGTCTCTTGCTGAGTATCAGTCCACTACATCTCTACCTCTGAGTATGCAAGGCTCTTCTTACATTGAAACGAACCCCAGGGATTTCTGGTACATCAAATCGATCCTTAAAATGTCTCCGAATTTGGATGTCACCAACAACCAGCCAAGTTTTATGAAAAGTCTATTTCCATTT
>Bg-c19218 len=716 count=4 IPR:/ blastx_SP:sp|Q0IHI3|CB062_XENLA/Uncharacterized protein C2orf62 homolog OS=Xenopus laevis PE=2 SV=1/2e-38/10-456/202-349 // sp|Q7Z7H3|CB062_HUMAN/Uncharacterized protein C2orf62 OS=Homo sapiens GN=C2orf62 PE=2 SV=1/4e-31/10-519/203-372 // sp|B9EKE5|CB062_MOUSE/Uncharacterized protein C2orf62 homolog OS=Mus musculus GN=Gm216 PE=2 SV=1/2e-28/10-474/190-344 // sp|Q08CH6|CB062_DANRE/Uncharacterized protein C2orf62 homolog OS=Danio rerio GN=zgc:153063 PE=2 SV=1/1e-27/16-480/187-349 // 
TGCCCTTAGGGGGATCTTTGCCCAGCCATCTATAAACGATTGCCTGAGCGAACACAAACAGTTGAAACGGTTGAGCTGAGGGTGTTTGGCATTGAGAGAACTATCAGTTCCTCTGTTGACTTGCCCACTTCCTGGCAATCTTATTTCATGAAAGATGGACACCTGACTAACAGAGTACAAGTTGGGTCTCCTGTCACTATGAAGCTGGCCAAAGTTCCTTTAAAAATTGAAAGAGATGAAGAAACCCCCAAACCTGTTTTTGAAAAGAAAGATCTCAAGTGGGAGGAAGACTTGCAGCTTCATTCAAGATTCTTGGACAGAAAGGAGGAACTGAAAGGCGACCATGCCACCTATATGTGTCATCATCCAGAACTCCGAGCTTTGTTGGCAGACTTTTTACAGTTCCTTCTTCTTCGAAAACCAGATGATGTCATCGCCTTTGCTGCTAATTATTTTTGCTGCTTTCTCCACCTTGATCCCTGACCCCTCACCTTATATGGCATCCAATGCCCCAACCCCATTCCCTGCCAGTAGAACAAATGCTAAAATTGATGAACTCAGAAATCTGTAAGGAGACATTGTACAGTATGATGCTATTGAAATCTGTTGTACTTTGTGTTTCATAATAATACACCAGAGTTGTGATATTAAAGTCTGCTGCCCATGTATTATAAAATGAACTAATGCAATTAACATCGTAAAAAGCTTGTGTTTTC
>Bg-c38388 len=258 count=2 IPR:/ blastx_SP:/
TCAGATGTAATGTTTATTCATAAGTAGATCATATATCTTAGCTGACGTCAAACAATAGAATGCTACAATTGAAGGGATCCTTAAGAGTCAGTTATCATAACGCAACTGAATGGAATGAATTGATTTCTAAAGTTGAGAAACATTACTCTCTAGTGTGACCCACAAGTTTGTATCAAATTCAAACACTTGATACGTACAGTAATTGCTAGTTTTTTGTTTTCTACAGCTATTATTTTTAAGTCTATGTTTATAAAACCA
>Bg-c23989 len=454 count=3 IPR:/ blastx_SP:/
GGCAAGTTTCACTCCTCACTTTGAAAGACCCCCAAGAAGTGGTTTTCCATTGGGTCCAACTGACTTTGGAGCATCTAGATCGGGAGAATCTAGCAGTAAAAGATCTTATGAAAGAGATGTTGATGAATTTCTTCGGCGTACAACTCATGGTCTCAAATTGAAAAGTCGTGATGACAGAAGAAGTGATAGGGACCATGACAGAGGGCGGGACAGAGATCGTGGAAGGGACCATGACAGAGATAGGGATCATCGCAGTGATAGAGATTATGACAGACGAGAACAGACTAGAGACAGAGATCGTGATCGTTCAAGCAGGCATCACAGAGACAGGCGATGAATGAGGATTTGGATAAATAGTATTAGTCTGTTGTATTGAGGTTGTAAAAGTGAGATCAATAAAGATGAAGGTATTTTAGTTTTAAATTGTGAGAACCAGTAGAAAATGTGTAGGTTT
>Bg-c29026 len=186 count=3 IPR:IPR002498:Phosphatidylinositol-4-phosphate 5-kinase, core blastx_SP:sp|Q5I6B8|PI51C_RAT/Phosphatidylinositol-4-phosphate 5-kinase type-1 gamma OS=Rattus norvegicus GN=Pip5k1c PE=1 SV=1/3e-19/186-1/261-322 // sp|O70161|PI51C_MOUSE/Phosphatidylinositol-4-phosphate 5-kinase type-1 gamma OS=Mus musculus GN=Pip5k1c PE=1 SV=2/3e-19/186-1/261-322 // sp|O60331|PI51C_HUMAN/Phosphatidylinositol-4-phosphate 5-kinase type-1 gamma OS=Homo sapiens GN=PIP5K1C PE=1 SV=2/3e-19/186-1/261-322 // sp|O14986|PI51B_HUMAN/Phosphatidylinositol-4-phosphate 5-kinase type-1 beta OS=Homo sapiens GN=PIP5K1B PE=1 SV=2/1e-18/186-1/211-272 // sp|Q5CZZ9|PI51B_RAT/Phosphatidylinositol-4-phosphate 5-kinase type-1 beta OS=Rattus norvegicus GN=Pip5k1b PE=2 SV=1/5e-18/186-1/211-272 // 
TCCTACAAGTAAACTATAGTCCATGATCTTAAAACTTTCTAAAACCCTACAGTCCCTCTGTAATGTCTTGATCAATGCGTCATATGTGTCTTTCTCCAGCTGTAGACCTTCCGGGTGGTTGTCCATGAAGTCCAGGTCCTTGAGTGTTGGGGAACTTTTAGACCGTTCATGCTTGGAGGCTTTCCT
>Bg-c38641 len=256 count=2 IPR:/ blastx_SP:/
AAAATGACGCAATGAGTCCCAAGGCCACACACTGGAGTACAGGGCAGGGTACAGCATTGAACATAACTGCTAAAATGGAAGTCTGGAGAAATTGTCTTCTTATAGTGGGAATGGAAATGAGCAGGGTGGATTTGCTTATAGGAATTAAATATGTTGATAAACTAATACATATTTTATTAATAGCATTAATTAATCTATTATCAACACTCCTTCTTTTATTATTGTTAATTAGGGTAACCCCGTTTAAACCTAAAGA
>Bg-c8894 len=532 count=12 IPR:/ blastx_SP:/
TCTAGCTTTAAATCTGACGTTTTGTGATTTATAAAAGAGAAATATAGGACAGTAAATTCTAAAAATACAAAATCTTTTTAGTATGCCATTAGATCGCTTTTAGGCAAGGCACATACTTAAATACTCAATACTTAATTACAGAATCACTAATATGTCAAAATAACCAAAGCTAAAGAAAACAGTATGCTCTGATCTAATGCAAAGTTGTTGTTTTTTTCTTTCTAAAAAATGCCAAATTCTGATTGCACTTCAGCGTATGCCAATAGAAATTTGATATCTAAGACAAATATTGCAGAAACATAATCAATAGACAATAATTTAAAAAACATGAAGAAACAGTTACAAATATTGAATGTGTCTTCATTTAAAATTCCCTAACTCTCGACCTGACAGAGTTGAGCTGAGCTTCGCTACAACTAAAGCAAAAAACATTATATTATCCTACAGCCTGACCTTAAATATAGTCTTCAAACCAAAATAAATTTAAAACACATTTTTTCATTAAATTGAACATTGGAGTTTAACTTACTTG
>Bg-c10199 len=549 count=10 IPR:/ blastx_SP:/
GTATTCCCCTTGTGGGGGTAAGATAAATAATAAAAAACAAATGGACCCTTTATTAAGAAATAAACTAACGGGTGGGTGTTTTAAATTGGCAACAGATTTACAAAAACAACATGAAGAAAGATGATATTTACGTTAAGCTATTTTGTCAAAGTATGGGACGTGCAACTTAAAAGACCTGTTTGCCCATCTAAGATCTTGCAATAAATATACGAAAGAAGAGGTGACATTGTTAATAAGATTTAACACAGAATTGGTTCTTAAGAAAATACTTCATTAGCGTAATTCTTTATCATCTTAGGGCAGCATGGATACTAGATGAAGGTACTCTTTCTCCAGGAATGTCTACAAATGTGATAGGACCAGAAATGAGCATACAGCTGTTGTAGCTAAACACAACAATCAAGTAATAATAAAGGCACAAAGTAGACCATGACATAAGTTAAAGTACAGTATTCATTTAGGACCTTGTGATCTTTGGGGGCAGTTCATGTGAAGCTTATCTGTTTCTATGGTGCCAAAGATTAACAAAGGTGTCATGTGACCAAACAA
>Bg-c4612 len=1373 count=26 IPR:/ blastx_SP:/
AATATAGAGATCTAGATTATAGGCCTAATATTAATTTAATTATAGTCACAACTTTGGATAAATGCTAAATGGAACTAAGAATAAAATCTACAGTAGGCTACTAGACTATATATATCTAGATCAACATATATACTTTGAATTACACCGAGGAACAAAGAAACACACATTTAAATAACAACGAGAATGGCCAGCCCCGAAAGCTTCCTAGCCAGATCGGGTATTCTTCCCGGGCGCAAAAGCCGTGTTCCGAGCTTGCTTCAAGAAATTCAGATGGACATTGTCAAGATAAAAAGAGTTTCAAGCGACACTTTGGCGTCCCCGATGCTTCGGTATGAAAAACGTAGTAACATGAAACCTCAGTTAGTTCTGACCTCAAAACTAGCAAACAAGTCAGCACAGAATGACGTTCTAGGGAAGACATCACTTGGGAAGACATCACTTGGGAAGACGTCATCACTGAGCCTCACTGAAAGTAAACGTGTGCTAAGCAAACAACATTTAGACAATCAAACAACTCATGGATTTCGATATCATTCAAATGGCGATGAACGTTTACCAACTAGACGCTCAGAAACTATAGATTTGTCTCGTGCAAACAGATATTTCCCCAAGTTTCCATCTGATGGGAAAAGAGATATTTATTTGGATAGCCGGATCATTTCTGAATACATGCAGAACAGCTATAGCGTTCCAAAGTTCAACAGAACATCTAAAAATCCGTCGAATTCTGTGATTGATGACTTTGAACAATGTTACAGGGATGGTTTTAGGCGGAAGAGTGAGCCTATTCTTAGACACCTGTCTCGGCTTTCAGAAGAACGATTTCTTAACGAAAAGGGCAGACTGAACCAGCCGTTAATGCAGTTGACCAGGAACATTGGTTACAAAGATAACTGGATTTACAACGTTAAGGCACAGCACGAGTTCCACTGCGATAGCATTACTCGTAGGAAAGGCAACGCAAAGGGAGCACGCAACGGCCGAGGTACAGCTCTGCAACCAGCACAAAGTTCTTTTAATATGCGATACGGTGTTTCTTCAAAGCTGCTAGCTTCTAGGAAGCCAACTTTGGAGGATTGCAGAATGCATGAGCTGGAAGAGGTGTCAAAGTTAAAAGGTCAGATTGACACATACACCAAAAGTGTTGAAGACCTGAAACGCAAGCCAAACAGCCAGGACATCATGAAGATCTTTTAAAAGTGAGTCGCAGAATTGTAGTTACAAAACTGGAACACGTTCCACAACTTTGTACCACTCATAGGTTGAAGAGTTCTGCAATACATTTAGAGTTAAACAATAATATAGCTTCCCATTGACTTATTATGTGTTTGAATTCCTTCTGGTATGTCATTAATAAAGAGCACACAGTTCAG
>Bg-c38876 len=254 count=2 IPR:/ blastx_SP:/
ACAGTCGGATTAATTCATACATTTGAATGCAACATTATTTTGGATAAAAAAAATTTTGAAATGAAAAAAAAAGGATTTTCTTCAGATGATTCTTATAAATTACTTGTTATTATTAAGTTAGTTGTCAATTGCTTAGCTACAGCTCCCATTTCTGAAGACAGTTCCCACGAGGGCAGTAGGCAACGCGGCCCGGANAAGTGTAATAGACAGCAGGGTTCTCGTCTGTTCGGTACTTCCCAGCAGTCCTTTCATCA
>Bg-c5753 len=1052 count=20 IPR:IPR001313:Pumilio RNA-binding repeat blastx_SP:sp|Q86U38|CN021_HUMAN/Pumilio domain-containing protein C14orf21 OS=Homo sapiens GN=C14orf21 PE=1 SV=1/2e-27/414-839/412-559 // sp|Q86U38|CN021_HUMAN/Pumilio domain-containing protein C14orf21 OS=Homo sapiens GN=C14orf21 PE=1 SV=1/2e-27/233-397/352-406 // sp|Q86U38|CN021_HUMAN/Pumilio domain-containing protein C14orf21 OS=Homo sapiens GN=C14orf21 PE=1 SV=1/2e-27/884-1051/573-627 // sp|Q8BMC4|CN021_MOUSE/Pumilio domain-containing protein C14orf21 homolog OS=Mus musculus PE=2 SV=1/1e-23/402-839/408-559 // sp|Q8BMC4|CN021_MOUSE/Pumilio domain-containing protein C14orf21 homolog OS=Mus musculus PE=2 SV=1/1e-23/884-1015/573-616 // 
CTGCAGACTGTCTTATTGATCCTACACAAAACATCCCCCAGTGAATACTACCAGCTGTCTAAGAGTATCATTGAGAAGACTCGCATCATGCCAGACAGTGAGGATGTGAATGCTAATGATGTCAGTAAGAAACTTCCACACATTGTCCAGAATGAAATAGGATCTTTCTTGATTGAGTTGTTGATATCCCTGGCCAGTGAGCCGCAGTTCAAGGAGATTTACAATCTCGTTTTTAAAGACAAAACTCATCTACTTTGCAGTTCATCCAGTGGCTGGCTTTGTCCTGAGGACTTTATTGTCGTCTGTGTCTGACAAATCATTGATGGAAGAAATAATGAGGACTCTACTTCAGTACACAGAAGACATCCTAGCTGTCAATCACATGGGTATAATAATAAAAAATGGCTGAATGTTGTTGCCGAACAGGACATCTACAGAATGAGTTTTTAAAGGCCTTGATGATGGCTTTCCATTGTTATGAGCCTGAGTTGAGGCAGATCAAAATTGTTCCACTGCTAGCATCCCTCCAGACTTACGACATATTTTTTAGCAGTGATTCGGGCAACACAGGATCTAGTGAGCCATCAGGCCACTTGTTGAAAAGTGTCAATATCCATGGGTCTCTGTTCCTTCAACAACTGCTCCAGTTTAAGAACACCAAGCTGGTGGTCTCGAGTCTAATGGGCCTGTCACCTGCTGAGGTGGCTGCCCTCTGCGCTGACAAATATGGCAGCCACCTCATTGACACGTTTTTTCAAAGCAAAGCTGTACCCGATAAGCAAAAGAATGCTTTTATCAAGTATTTACAGGGTAACTATGTCAATATAGCTTGCAACAGAAAATGGCTCAAAGGTGTTTTAGAAAAACATTTGGAAAAAGTTGTTCCTTGAAACAGAGAGAAACAATAGCAGATGAACTGTGTCAGAAACAAGAGAGGGTTGAGAGTGACAATTTTGGTCGTTTTCTGTACAGGAATTTCGCTCTGTTCAAATTTTCTCACCGCAAACAGGAGTGGTTAAGAAAATTCAAGGGGGCCAGCTTGAAGAAGCAAA
>Bg-c25845 len=318 count=3 IPR:IPR004045:Glutathione S-transferase, N-terminal; IPR012335:Thioredoxin fold blastx_SP:sp|O18598|GST1_BLAGE/Glutathione S-transferase OS=Blattella germanica PE=1 SV=3/4e-10/41-256/45-116 // sp|P46428|GST_ANOGA/Glutathione S-transferase OS=Anopheles gambiae GN=GstS1 PE=2 SV=4/6e-10/41-268/44-120 // sp|Q09596|GST5_CAEEL/Probable glutathione S-transferase 5 OS=Caenorhabditis elegans GN=gst-5 PE=1 SV=1/8e-10/50-202/49-99 // sp|P46429|GST2_MANSE/Glutathione S-transferase 2 OS=Manduca sexta GN=GST2 PE=2 SV=1/2e-09/41-253/43-113 // sp|P41043|GST1_DROME/Glutathione S-transferase S1 OS=Drosophila melanogaster GN=GstS1 PE=1 SV=2/2e-08/41-268/90-166 // 
GTCAGGCTGGCCNGGGAAAAGTGGCAAGAGGTTAAAAGCCCCACAACCCCCCTTTGGCCAGGTCCCTGTGCTGGAGATTGACGGTAAGAAAAAGGCGCAGTCCATCGCCCTAGCCGCCTTCCTCGCCCGGGAGTTCAAGCTGTACGGCAAAGACAACTGGGACGCCCTGACCATCGACACTGTGGCTCAGCTTGCTGAAGATCTGATCCAGTCCTATGCCAGATCCTTCCGCGAGAGCGATCCGGTACAAAAAAGAGCGATCGTGACAGAGGTACAAAACAGAGGTACGGACCCCAATTCCTTGGCTTCTTCGAGTCG
>Bg-c7044 len=591 count=16 IPR:IPR001304:C-type lectin; IPR016186:C-type lectin-like blastx_SP:sp|P82596|PLC_HALLA/Perlucin OS=Haliotis laevigata PE=1 SV=3/7e-04/411-91/24-122 // 
TATTTTAATTGTAAGCAACTTGCGACATGAAATAATTTTAATGTTATTGTTAATTTTATATGCAATATACTTCTCTTCTTAACACCTATCTGGTACTTCGCAGACATAGCCTAAATCATTCCTATTAGAGTTACACAAACACGCCATAAGCTTCCAATCATTTCGTGGGTAAGAACACTGGCAATTACCACTTTGGTAATTAGCTGGTTCTCCTGGCGCCCAGTCAAACAACGGCCTGGCTGCTGGACTGTGCTCATTTACCCATGAGCCGTTCTGCTTTGTTGCCCCTGACCAGAAGTAATATGAGATATTCATTTTGATCAAGTACTCTCTAAAGAAATCCATCTCTCCCTGAGTGTCTAGTTCTGCAAGGTATCCGTCGATTAACTTGCAAGAATTCTGAGCTTCTGCAATAAAAAAAACGTGATATGTTTTGAGTCATGTAGTAGCTGTGGCCATTGAAGAAAACAGGTGAGTAGAAGTTTGACTTGGTTTTTTAATTTATTAATGTATTCATCTTTCCTGCTCATGCAGCTCTGCATGGCATTAGAAAGATGTTGCAATAGACTTAGATTTGCGTTAGCCTNAGTA
>Bg-c34165 len=308 count=2 IPR:/ blastx_SP:/
TGGTTCAAGCATGTTTGTGCTTACTTGTCAGATATAATATGTTTTTACTGGTTAATTAGTTATTTGCTTCTTATTTTATGTTAATTTTTACCAATGTTTCCTTGATGTTATCATTTTAAATCACTGACAAAAACAAAAGCATTTATTAACTTAAGAATCTAACAATCATTTCAATAATAGGACAATTTATTTTCAAAACAATCAAACCAGAATTTGTTTCTCATTTTAAAAAATTGTTTTTTTTAATCAATTTTGTTTTTATTCACTAGAAAAACTCTTTGGTACTTCAAGAAAAAAGACTGTTAAAA
>Bg-c8033 len=342 count=14 IPR:/ blastx_SP:/
AAACTGCGGAGGTTACGGAGTCGGATGCGGCACAGGGGGCGGTAACAAAGGGAACGGTAACCAAGGGAACGGTAACCAAGGAAACGGTAACCAAAGAAGTGGCAACCAAGGGGGGAGTGGTTCAGGAGGGTGTAGCCAGGCCAACAATCCTTTTCTAAACAGAAATCAAAGCTCTGGGGGCTGTGGAGGAAGCGGGGGCGGCTCCTGTGGGACCAGTAGGTGTGGTGGCTCTAACACTATGCAGGGTTAAAGTTTAATGTATTCATCTTTTTTTTTTGTCCTGATCATCCAAATAGTCCATAATTTTGTATGCATTTTGTTAATAAAAATACTTGTTACAAC
>Bg-c42927 len=125 count=2 IPR:/ blastx_SP:/
CCTGATCTTTTCTCAAATGTGGGCTCAAACCTACACAACATGCCAAGGGGAAAAAAAAAATAGCATTACTGCTCTAGTTTAAAGAGCTCTTTACATTCGTTCTCCCAAGGCACACAGGGGATAGG
>Bg-c27993 len=254 count=3 IPR:IPR002937:Amine oxidase blastx_SP:sp|Q6QHF9|PAOX_HUMAN/Peroxisomal N(1)-acetyl-spermine/spermidine oxidase OS=Homo sapiens GN=PAOX PE=1 SV=3/5e-09/224-33/406-469 // sp|Q99K82|SMOX_MOUSE/Spermine oxidase OS=Mus musculus GN=Smox PE=1 SV=1/1e-08/224-27/313-378 // sp|Q9NWM0|SMOX_HUMAN/Spermine oxidase OS=Homo sapiens GN=SMOX PE=1 SV=1/1e-08/224-27/313-378 // sp|Q865R1|PAOX_BOVIN/Peroxisomal N(1)-acetyl-spermine/spermidine oxidase OS=Bos taurus GN=PAOX PE=1 SV=3/5e-08/254-33/258-332 // sp|Q8C0L6|PAOX_MOUSE/Peroxisomal N(1)-acetyl-spermine/spermidine oxidase OS=Mus musculus GN=Paox PE=1 SV=3/8e-08/224-33/261-324 // 
ACCTCACCGTATGGAGTCTGAGTGTAGCCTACAAATGGCTTGTCCCATTTAATAAACAATTTTCCGATTCCTCCAAAACCCAGATTGTTGATGGCAGTCTGTGCTGAAAAGGGAAGCTGAGGTGTGAAGAGCTGCTGATGTTTGGCCTGGAGGTATCCAATACTAGTGGTCACTATCACATGGTCAGCTAAAAAAGTTTCTCCATTGTCACAAGTGACAGAAACTTTACCAGAATCTGGTGACTTTGCATTACG
>Bg-c9336 len=729 count=11 IPR:/ blastx_SP:/
CACTATTGATATTTTTATACTAATTTTTTATTTATATTATTAATTATTTATTATTATCTAAGCATCTGTTTGTATTTGAGCTGGGTAATGAATATAGATTCTTAAATTTAATTGTTGTTTGTTGCTTTACTCACTTTTAAACTGTGACCATGTAAATTTTGTCTAGATGGAGAATGGTAATGTCTTAGTGTTTATGATTAGATTGAGAATGTTACAATGTCTTAGTGTCAATGATAACAGTGAGAATGGTAATGTCTTAGTGTCAATGATAACAGTGAGAATGGTAATGTCTTAGTGTTATTGATTAGATTGATAATGGTAATGTCTTAGTGTCAATGATTACATTGAGAATAGTAATGTCTTAGTGCCAATGATAAAACTGAGACAATCATTAGATTGAGGATGGTAATGTCTTAGTGTCAATGGTGTAACACTATGATTAGAGTGAGATGCACGAGATGATTTCAGTTCACGTGAAAATGGGTCTTACACATTGTCAAGCAAAGAGGTACCATTTTTGATCACATGACCACGATTTTTTTAGTAAGTTAGCACCATGATGTACCAAGAGACGTAGGTGTCTCTGTTTTATGAAATTGGATTAAATAATATGAGTACCTAATTGGAGATATTTCTATGGATGTAGTTTGTATTTGATATGAAATAATTCGTGATGATATTTTATGGCTGAGATTGCCTACTTTATGAACTATTATTTGATGTAATAAA
>Bg-c43207 len=100 count=2 IPR:/ blastx_SP:/
AAAACAACAACAACCATATAATGATATAAAATAAATACACTCATCTCACCACCCCTTGCCTCTAATATACAGGATTAAAAAAAGGAACACGGAACCAAAT
>Bg-c36782 len=272 count=2 IPR:/ blastx_SP:/
CTTAAGAGATCTTCATTGTCACTAATGAGCTGCCTTTAATCACATAGGAATAGATCGTAGTAGCACAATTCGTCCTTCTTCACGATGTCGTAACTTTTTGCACACCTTCGCTTCACAGTTGATATCGGTTCTAGATTCTTTTTCTTTTGAACACACAATCTAAGAATTCCTTGCAAACTTTCTTGGTAAAAGCAAAAGCTCCTTCGCAACCCTGTCTTTCGTATCTTCTTGCAAAACCATTATGCTCCGCTGAGCAGGGATGGAGAAGGGAT
>Bg-c23030 len=744 count=3 IPR:IPR013320:Concanavalin A-like lectin/glucanase, subgroup; IPR001079:Galectin, carbohydrate recognition domain blastx_SP:/
TCTTGATTTTTGCTTTTGGCATAGGGTATTAGAGATAACATCAAAAAAATGGATAAAGAACCGGATACAAAAGAGAAAGCGATAAAGCCAAAGGAGAGAGTTCCTTACTTACAACTAATAAAAGGGACCATTGCTCCTGGTTCAAGCTCCATTCTTATAGAAGGCAAGATTTCTAGTACTTGTAAAAGCTTTTTCGTAAGACTTCAGTGTGATCAAAACATCGACAAATCTGATTTCCCAGTTATGTTCGAGTTCATCTTTGACAAAGATGAGAAACAAGAATTGACATCAGTCGTAAGCAAGTCTAAGGGACAAAAATCAGACGCCATGGACTTCAATCAAAAAGAATTTCCATTCTCACACAATAACGAATTTAAGCTTGAAATTAAGATTACTGAATCCAACAGTTGTACAGTAATCGTGGATGATAATGTTTACATGTCTTGTGATACCACAAATGGACTGACTAAAGACTTGGGGCTTGAAAAGTCTAAATGGCTTTACATCGATGGTCGCATTGAGTTAAACAAAGTTTACACCCACTGAAGAAGTTTTTCATTTATAAAAAGTTTGAGGAATGCATTGTAAATTGTAACGCAATGAATGTAATTACTATCATTTATTAATTGTATGATTACGAACTTCTTTAGAATATCTTTTTTTCTGTATTTCGGAAAACAATTACTATTGTCCAGTGTCTTAAAATAGTATGTCACAAATTAAACCATTAATATTCAAAAAAAA
>Bg-c41542 len=224 count=2 IPR:/ blastx_SP:/
AAGTCGTTGCTATGATTGATGTAAAGAAGAAAACAATGGAAGGATATATTGCAAGGACTGTTTCTATTAAAGCAGAAAATTTGGCATTTTGTGACCATATTAACAACGAGTCTTTATTGAATAAAGAATTTCCATGAGATAGAAAAAAAAAAAAAAACATGTACAATATCCCCAACAATATAAAAATAATGCACAGTAGAAATAAACAGGATTACAACAAAGGA
>Bg-c4964 len=1063 count=24 IPR:/ blastx_SP:/
GTTTGATTAGGACTACCTTTATGCCAGTAATGGAGTTTATTTATCTGTACAAGTCATGGTAAAAACAATGCAGTCACAAGATGTGAACATTCTTTGACTGTAGAATAAAAATATTACAAAAGCAGTAGGCCTAATAGATATTACACAACTTTTATATCCTCATACAAAGTTTGATTCAGATTGAAGTGACTTGTGTTGAAGACTATAGATAGGTAAGTTGTTTCTCTTTGAGTCGCTGAATGAAATGCGCACAAACAATAAAAAGGGAGACAGATCATTTTAATGAGCAGCCTAGCTCCTTGGCAAAAGGGCCAACAGTTTTTTTAAACACTGATCTTGTAAAGTTGGCAGCTTTCTTGGAACATGTCCTCTCCACGTTGATGTGAATACAGTTGAGTAAATTTTGAAAGACTCCGCATAGTCCAGTAACGGTTTTGCTTGGTTTGTCTTGTTTTGCTAGATTGACAGTTGTCTGCAATTGCAGAGTGGCGATGTCCGACTCCAGCCTCATTGCCCTGGAACACTGGTAGGCGCCCCATTCAAAGTCATCCCTCGAGAAGCACCCTGCTTCTGACAGCAACACATCTCTACCTTCACCACAGATAAACTCCAGAGTGTCTCTCACCGCATGGCTGACATAATATTTGTTAAAAATGTCGCACGGAGACTTAGAGATGCATTCATTTAGTGACATTTGCTTACTGCAAACACTGTCTAATAGGACTCCATAAGTGAGTGTTGCTAGAGCTCCGGCCTTGTCTTTCTGTATCAGATCTACCAACATGGGGAACGGGTCGAGGCACGTTCTCAGAGATGCACATCCTTTTCTGCTTTTTGACGTTTGTAATCTTGCCAGCGAAGTATAAAGAAAAACTAAATTACACAAGAACACTGTACGTAACCACATCTTGGAAGTTATGAAGTTATATGCAAATAATTGTACTATAACGAAACAAGAAATCCAGACAATGGGCTAGGTGCAGGTAACTCCTCGTTCGTTAGCACAATTTGACACTTGAGCTTTACCTTTTGTCCCCGCGTCGGATGCGTTGTTACCACTGCT
>Bg-c32463 len=362 count=2 IPR:/ blastx_SP:/
CAGTGCTTAATGTTAGCAGATAAATTTGAGTTGTTTCTTTTAGGGTTTGTTCCCTGACCTAATTCCATTTGTTATCTACGTCCCGGCTGTTATGTGACAGCTTTTTTCACTGGAAAATGTTCATTGCATTTAATAGAATATCGGTTTGTGAAAGGGCAGACTTAACATGTAGGTTACCTAAGTACTATTTTTATCTCCACGCAATGCCATTGCAAAATAAAGAACAATTACTGAGAATGTCTTCATACATTGAAATTGTTTTTTTTTACTTTATAGAAATTTTAATTGCAATTTTCTAACAAAGTAATGCGTTGTGATATTTTGTTTCTTTGTAATTTATTTATTAAACTTGTTTATTATTG
>Bg-c31675 len=399 count=2 IPR:/ blastx_SP:/
CATTTCAATTATATTGTTTTATTTAAGTTATTGTCCCCTTTGTGGTATGTGAGTACAACACACTGTCACTGTGATGCTAGTACTAAACAAATTTAAACCGTTTTGGAGTTGTTTTTTTTTTTTTTTTTTTGTGTAACATAATTAAGGTTACAACTGAAAACAGTTTTGCTGTAAAGTCAAAAACCAAGACATGGTGATATTCATTGATGACCAGTGGAACTAAAGTCAGTTGAAGGAACTCTTGATAAATGTTAACTATGTTAATAAATAAATATCAATGTTTCTTAATCTACAGAAAAAGCTGGGCCTGTTTACCTTATGCCCTTTTAATATTTTTTTTTCATTTTAAAGAATTTTGGTCTGGTTATGTCTCAAGATAATTATTCTTCCTATCTTCTT
>Bg-c22142 len=261 count=4 IPR:/ blastx_SP:/
TTCACCAATTTGAATAGAGTGCTCTCCCCTTTACGACAACCCACCAATAGCGTGAGAGTTTGCAATAGCCTTCACGTAGACATGCGCATACACAAGCAGACTCTTGAATCGAAGCGCTTAGAACGAACAATACAAAAAATCTAATAATAAAACATTTATGCTTCAAAATTTCAAACAGTTTTGTTTTTTTTTTAGACATGGTCCTACAGGCTAGGAGGACTTTTTTTCTAACAGTTTAGAGGTTGAAATGCTTACAATAAA
>Bg-c39137 len=252 count=2 IPR:/ blastx_SP:sp|Q9P2K1|C2D2A_HUMAN/Coiled-coil and C2 domain-containing protein 2A OS=Homo sapiens GN=CC2D2A PE=1 SV=3/2e-17/247-2/475-556 // sp|Q8CFW7|C2D2A_MOUSE/Coiled-coil and C2 domain-containing protein 2A OS=Mus musculus GN=Cc2d2a PE=2 SV=1/7e-17/247-2/475-556 // 
GAAGTTCTTCCTCAATTTCAAATTTCCATCTTTCTAAATCTTCAGCTTTGTTTGTTTCCTCTTTTTGTATCTGCAGTTTGACAGGTGTGGTAATAAACTTCTGTTGGTCTCTCAATGCTTTGATTTCTTTCCAAGTATGTATGATATTTTTTAATAATGTTCTATCAGTTTGTTCTTCTCTGTCCCTCAAGAAGCGAGTTTGCCGTATTTCACTTTTGTAGTCTCGTAATCTCCTCTCATAATTATCTCTGT
>Bg-c1897 len=587 count=71 IPR:/ blastx_SP:/
CTGTAGAAGAGACTCAATGGTTTTTAGGTCCAATTTCTTTTGCAACATCTAGAGGGAGGGGTAACTGGTAGAAGGCTTTTGTGATTCCATCTTTTGATCAGCTAACACATCTTTTGATCAGCTAACACATCTTTTAATCAGCTAACAAATCTTTTAATCAGCTAACACATCTTTTGTTCAGCTAACACATCTTTTGTTCAGCTAACACATCTTTTATTCAGCTAACACATCTTTTGTTCAGCTAACACATCTTTTGATCAGCTAACAAGCCAGAGTTAAGCTGACAAGGGCACCAAGACAAGAGACCTTTTCTCTTTCCAACCTGAATAGGGCCACCGTTGATGGAGTAAAATGAATATAGGCTCAGTATGTTTTCACTCCAAATGTTTCTTTATCTGTGTACAGAAGGTCCTGGCAAACAAAGTGGTCTCTCCAAAACAATTATATGGAATGAAGCTTTAACGTAACAACGAAATAAGCGACTTATTTGTTCTACTCATTGTTATATTCAGTGTTCCATTTAACAGACTAGTCGGTCTTGTATCTCTTCAAACATCAAACAATACAGAATACACTATCAGCAGGTA
>Bg-c8318 len=564 count=13 IPR:/ blastx_SP:/
TGCTTTATTTCTTTTATTTGTTCCTCATTGTATTATGATATGACAGCAAAGCATTTAATTCCTTGTTGAAAAAAAGAGTTCAGTGAATAAATAAAAAAGAAGCTGAACAAACACAAAAAAGTACATGAAAGAAACAAAGCATAAAAGCTAAACATTTCGATGATGTTTCTGTTTAAGCAAATTTATAAAACAATAAATAAAGTAAAAAAATGATAATCATACTAGCAAAATATGAAAAATAAAAAGCTTTAAAAAAACATCAACAAAGCTTATCTAAGGAAAGAGAAAGATTTTTTTTAATCGAATAAGGGAAACAACATCTAAGTTCTTGAAGATATAGTTGTAAACGCAGAGCTTTTTTTTTTTTTACTAATGTTTTGTTAGGTACAATAAATAATTTTTTTTAAAGTAATGGTGCGAGAGAAATAACGTGTTCAATCATCTAAGAGGAAGAAACCCTAAATATTTAAAAGTATCTGTGAATACTGAAGTATTAATTTCCCATGTCGGTATCTAATAATTAATTGACTAATTGTTGTTTTTTTAAGTCTTGTCTATGACAAT
>Bg-c5435 len=488 count=22 IPR:/ blastx_SP:/
ACACCTATAAGGCTGGTGAAAGCTACTTCAGCTTCAATCCATATAGTTTCTATGATTTTGAAAAAGATATGCACAAGCACAGAGCGCCACAGCCAAGTTCTCTTCCCAAGGCATAATTTTCTATTTATTTATTATTAGTTTCAAAGTTTTGTTGGTTAATAAGTTCTTAAAGTTTTTAAAGTTGAAATTGACATTTTAATTGACTTTTAATTGTTATGATATTGTCATCTGAAGATACAAAATTTTTAACATTTATGTCTTCATTTTACTGAAAGCCTAAATATTTGATTGTGTAACAGCACTAAATTTAAGCAAAATAGTTTCAAGAAAATACTTAGTTTTATATTTTTCACATTATTTCTAGCTACTTTAAAAGCAAAAGCAATGCTTGGACTCCTCATGTCAGTGTAAATAGTATACATGTATAATTTGTAATATAGAAACTGGTTTATGAATGATTAAAATATTATATTTGACTTGTGAATTGC
>Bg-c27896 len=256 count=3 IPR:IPR001060:Fps/Fes/Fer/CIP4 homology blastx_SP:sp|Q5I0D6|NOSTN_RAT/Nostrin OS=Rattus norvegicus GN=Nostrin PE=2 SV=1/7e-07/216-1/14-86 // sp|Q6WKZ7|NOSTN_MOUSE/Nostrin OS=Mus musculus GN=Nostrin PE=1 SV=2/1e-06/216-1/14-86 // sp|Q8IVI9|NOSTN_HUMAN/Nostrin OS=Homo sapiens GN=NOSTRIN PE=1 SV=1/1e-06/216-1/14-86 // sp|Q2KJB5|NOSTN_BOVIN/Nostrin OS=Bos taurus GN=NOSTRIN PE=1 SV=1/2e-06/216-1/14-86 // sp|O55148|GAS7_RAT/Growth arrest-specific protein 7 OS=Rattus norvegicus GN=Gas7 PE=2 SV=2/8e-05/219-1/164-234 // 
GTGTAATTCTGCTTCTTGTTCCATGGCTACTGCTACTGCCTTCCATCCATCTGTTAGAGAACCTACTCCAGTGCCACATGCCTTCAACAGTTTAGTGGCGATCTTTGATAAACTTTTGGCATACAGAGTTTCAGCCTCTGACCTTTCTTGTAATAATATAGAAACTTCTTTACTGAATTCGTTGCCTTGTTTAATAAATTTTCTCAATTCTTCAAATCCATCGAAACCCTTTGCGATGTTACTGGCAGGCAGTGGG
>Bg-c11056 len=554 count=9 IPR:/ blastx_SP:sp|A5D9D4|MSTO1_BOVIN/Protein misato homolog 1 OS=Bos taurus GN=MSTO1 PE=2 SV=2/4e-11/14-346/459-568 // sp|Q9BUK6|MSTO1_HUMAN/Protein misato homolog 1 OS=Homo sapiens GN=MSTO1 PE=1 SV=1/1e-09/14-334/459-563 // sp|Q5RF82|MSTO1_PONPY/Protein misato homolog 1 OS=Pongo pygmaeus GN=MSTO1 PE=2 SV=1/1e-09/14-349/459-568 // sp|Q2YDW2|MSTO1_MOUSE/Protein misato homolog 1 OS=Mus musculus GN=Msto1 PE=2 SV=1/2e-09/14-349/445-554 // sp|Q4R681|MSTO1_MACFA/Protein misato homolog 1 OS=Macaca fascicularis GN=MSTO1 PE=2 SV=2/1e-08/14-331/459-561 // 
CTCAGAAGTGTTCCCGAGTTCACAGAGTGCTAGTTTTGTTCTACGAGATAGTGTTAAAGTGGGATTACCCTTTCCACACATATTTGATCCTCATATCAACCATTGTGGGTTTATTACAGATACTCTTAGGCCAGCATTATCAGGTGTTGAAACTGTTCCCATCATGACCTCCTTGCAGTCTAATCCAGATGTTTTTAGTTACATTGACAGCCTGGAGAGTTGTGTCTCTAAATTTAACATAGCCAAGCATCAACACTTTATTGAAGCTGGCTTGGAGCCAGATGATTTCACTGAGATGTTATATAATCTCAAATCTTTAGCACAATGTTATAAACACTTGGAAGATCTATAGCAGATTTCAGTTACAAAAATCTTGATAACCCAATGTAACTTGGACTTAACTTTTTATATCAGCTCAAAGCATTGCCACAATTATATTGATAATCTTTTCTCTGACACATTGTTGCAATTTAGTGAAAGCCAGAGTAGTGATATTTAGCAGTGTTATTTTTGTGTGATATTTATGAAACAGTATAAAAATTGTTGTCTAAAAA
>Bg-c9604 len=450 count=11 IPR:/ blastx_SP:/
ATGAATTATCAGTAGATGCTTATGTGTACATGTAGAGATGTATTTAGGTGCAAGAGGTTGAAGTCCATTATAACTCACTAACTCAACTGCAATGAAGACACACACAAGATTTAAAGTAAAAAAAACAACTAAGTCCTCTAAAGATGAAGACCTTAATTTTTAATACAAAATGGGAACACTCGAGTCTCTGTTCTTTTTAAAGTAAAATGACCAAGTCCACTAAAGACACACAAGATTTAAAGCAAAATGACTTAAGTCCACTAAAGACACACAAGATTTAAAGCAAAATGACTTAAGTCCACTAAATACATATACAAGATATAAAGTAAAATGACTTAAGTCCACTAAATACATATACAAGATTTAAAGTAAAATGATGTAAATGAAGTAAAAGTCATCTAAAGGTGAAGACCTTAACTTTTACTGCAAAACAGGAACAGTCTAGTCTCT
>Bg-c33133 len=336 count=2 IPR:/ blastx_SP:/
TTTTTAAATCAATTTTTACTAACGTTTGTTTAGTGTCATCTCCTAGAGCTCATGTGGGCCCCAATTAGTCATTGTCATGGGCCCAAACGCAATGATTCCCTTTGCACTTAGGTTGCTACGCCACAGGCTATGGGCTCCTAATGGTCGTGGGCCCGGGTTCACTGAACCTCTTTGCGCCATGGATGCTACGCCACTGGATAGATATCCTCATTTTGACAGTTTACAAAACTAAAAGATCTATTATTGATCTAGACTAGCTCTACACTAGATCAAATAGATTTGTCAAAAATAGTTTAAACTTTTGTTGGAAACCTCCGTCACGTGAACGTTGCTAAA
>Bg-c20334 len=411 count=4 IPR:/ blastx_SP:/
TTATTACAAAGAATAACACTTTGACATTTCAATGAAACAATATTAGTACACATTTTATCACATCAATCAGTCTTCCTATAGTAACAATTTTCTCTACATGGAAGTTCATCTTTGATAGTTTCCTTGCAGAGCTTGAATCATTTGATCAAGTTTATAAACATCTTTGTCTAGTGCAGCAATCTTGTTTTTTAAGTGGATTGCCTTTTGGGAGTCTCCTTTGTTTTTCCTGAGAAATTTGTCTAAGTTTTTCATTCAACTCTTTCTTGTCTTGTTCTTGGTTTTCCTTTTTCTTTTGAAGTCTATCTAGTTCTTGAGTCAAATCTACTGAAGTTCCCTTCAGCTCAACCTTGACCTTGAGCTCAGGATTAACATAAACTTCAGTACACATTTCTGTCACTTGGTCTGAGGGTT
>Bg-c30635 len=483 count=2 IPR:IPR001813:Ribosomal protein 60S blastx_SP:/
TTCGAAGATTCTCTCCAGTGTCGGAATCGAATGTGATGCAAAAAAAGCTCAAAATGTCATCGATGCTTGTCACGGCAAATCTGTTGATGATATCATTGCTGAAGGTACGAAAAATTAGCCAGTTTACCAGCTGGTGGTGCGGCTCCAAGTGGTGGAGCTGGTGCACCTGCAGCCGCTCCTGCTGAAACGAAAAAAGAAGCAGCGAAAGAAGAACCGAAGAAAGAAGAGAAAAAGAAAGAAGAATCCGATGATGAAGGAGATATGGGCTTCGGTCTTTTCGATTAAGTTGCACATCATCAAGGGACCAACCAGCAAAACTCATCATTTGACGTTCATTTTCAACAAATTCACAATGGATTTGAAATTTTCTTTTGAGTTTTTGTTGATGTGCGTTTGCGTGTGTTTGTTTTCGAATTGTGTCCTCGTCTTATCCGTAAAAAAAAAGAACTTGACAAAATAATAAAATAATCGCCCGAAAGATCA
>Bg-c40816 len=236 count=2 IPR:/ blastx_SP:/
AAATTATTAACAAAACGACCATTGAAGCTCTGGGGGTGGAACACCGGCTGCTGATGGTCACAGATGGGGACTCGTTAGCAAGTCTAGTTAGACATCCGTAGCGGTAAATAAATATATCGGCGGGAGTTGGTCACATCGCGTCGACCTTAGCTAGCAATTCATTGTTAATAATTATACAATAGCAGGCACTACACGATGTGTGTTGAGATAATAGTAGATGTGTATTGAGATAATAA
>Bg-c24554 len=399 count=3 IPR:/ blastx_SP:/
TACTAGCGGCAATAGAATACAAGATATGAATGAGAGAATACTCCGCAGCAGTAAATCTTCCTAACCAAGGCATCAAGACTGGCCAACATCAGCTAGCAGCTATTTAGTGATGACAGGATAATCCCAAAGGACCATCAAGATATTTGTAAATCAGTAGTGTTAACTAGTTGAACCTCGGTCCCCTAGAGGGCAAGTAGTCACATAACTAGCCAAATCTCTAACCATGACATCACATTCACTTCACCAACTATCTGATAAATCTAGAGCATAAGAATAGTGATGTTTTAGCACTTTCAGACTGACATAAACATTGTTTTGTCAAAATGGTGGCTGCATTCATTGCATCAACACAGTGAAACCTTCTTGAACCTACTTAGATGTAGTCAAACTGAGAAGCTA
>Bg-c42513 len=163 count=2 IPR:/ blastx_SP:/
ACTGGGGTCAATAAAGCAAAGCGTATGTCGGAAAGTTTCTGCTGCAACAATTCAATGACATTATCAAAATTTAAGATTAAATTCCCATAAACTCATAGCAATCGAACTGTATTTGCACAACGATTGTTCATGCTTTTATTAAAATATATAATGAAACTACTGA
>Bg-c8915 len=513 count=12 IPR:/ blastx_SP:/
GAATTGAAACATTTTATTGATATCTAGCAACAGAATGGCACAATTGTGACAATATCAAAGTAGCTGAGCTGAGAGAAATATACCAATACTTGTGCTTCACATTGAAAGATGCATTTAAGGGAGTGGCCAGTCTTCAGTGGAAATGTCAACATGGAATGTCAGCAAAGGACAAAATATTAAGTCTCTAAAGTATTGCCAGAGTGGCTTAATGAAATAGGTATAAAAATATATAATTTGTGTAATTAAAATACAAAAACAAATATTTTTTTGGGGGGAAGGGGGGGGGGGGAGGTATCAAATTATAGAGGTACTTGAAAAAAAGTCGTGGAATTAAACTAAACATTAATAACTAGACAGTTATCTATATCTATACTACTAAATAGCCCAACAACTAAGCAATGTTTTAATTAACCTACAATTAACCAGTGTAAAATACCCAATAAATAGATGCTAACAAAAAAAAAAATACAGGACAATGATCACAACTATTGGTTGGTCAAATTATACCAATAG
>Bg-c38642 len=256 count=2 IPR:/ blastx_SP:/
TCATTTCTTCTCTTCAGCACCAACTACATGAATCCAAGGCTAGAACTAAATACAAATGTTGCCTACGATAAAGATCAAGTTAGAGAAAACATTTTAAACGACATGTGTTCAGAAATTAAAAAGTTTGTTTAAAATAAAAAGGACAAAATGAATACTTTGTGCTGTAATAGTAAGCCAGAAGTGACCTTGAGATTAGGTCAAAAAAAAAAAAATAGTACTCTAGAAATCTAATGAAAAACATACAGGGCTTTAGTTT
>Bg-c8883 len=547 count=12 IPR:/ blastx_SP:/
TTTTTTTAACTTAAAAAAAAGTTTAATTTCCGAAATGGCCGAAGACAAAAACTGAATAGCATTTGATCACAAAACCAAAAAATAAAATTATGATACTAAAATGAAACTAAAAAATGCAATATGCCAACTATGCTACTGATAAAAAAAGGTATCTAAACTACATCTCATTGTATACAAATAAAGAGATTTTAATGTTGAAATGAAGAGCGAGATGTGAATGGCATCCATCAACAGACAAATCTCTCTTTTTTAATAGACGTGGTGAATGTAATTGCTTTACAGAAAGACTTTGATTGTCGACATTCATGTGCAGAGTAACGGGATAAAATAAAATGATCTCGATCTTGAGTCAAGTCGTCTGCCACATTGCCTAGCAACCATACATTTCCCACGCCGAATATGACATCCGTCTTTTTGAATGGGCTATTTAAAAACATAGAATCAGGAATAGAAAAGAATGTCTACTTCAAGCAATGCTTCGTCATTAGGTTCCTTTGTCTCAGTACAGCGGAACAAAGGGGGAAAAAAAAGGAATTCAAGAGAAAGA
>Bg-c27042 len=275 count=3 IPR:/ blastx_SP:/
TTCTTACTGTGTACAACTCAAATAAATCTATTGACACGAATGTACATTCGATAAATCTCATGACAAGTTTCACCTATTAGGTGAGGAGAAAGTCTGGTGGGCTATTTCTATCAATCAAACTGACAAAGATTCAATTACAGAATCGTCACGTACACTAGGTCAATACAAAAGGAATCCAAAAGCATTACAAATATTTGTATTAAAAATAAATTCTAGATCGTTTAAAAAAAAAGAAATGTTTGTTTGTTTGTCTACTTCAAACGTAAAACTAGACT
>Bg-c11426 len=352 count=9 IPR:/ blastx_SP:/
TCATTTAATAGTAGATTTATTTAATTAAATAGCTGAATTTTCTTTTATTAGTACTTTGCAACTAATATTTGTTGTATATATATGTCTAATAGGTTACACTGCATATAGATATGAGCAAAATTCATTAGAATTCCAGCTTTTGTGAAAGTGTTAAAATTCTTTTTAAGAATAAAAAATATTGCAAGGGCAGGAGACCCTATCTCATTACATGTCACTTCACTTCAATAACAAACATAACATAGTTAATACTTCAACAACAAACATAACAGATAAACTATTTGATACTTTCAAATGAATCATATCATGGAATCAACAAAACTGGTACAAGAGATGTCAAGTAAGCTTATGGTAT
>Bg-c30338 len=554 count=2 IPR:IPR015898:G-protein, gamma-like subunit; IPR001770:G-protein, gamma subunit blastx_SP:sp|P38040|GBG1_DROME/Guanine nucleotide-binding protein subunit gamma-1 OS=Drosophila melanogaster GN=Ggamma1 PE=2 SV=1/1e-05/163-309/22-70 // sp|Q28024|GBG12_BOVIN/Guanine nucleotide-binding protein G(I)/G(S)/G(O) subunit gamma-12 OS=Bos taurus GN=GNG12 PE=1 SV=2/7e-05/163-309/25-72 // sp|Q9DAS9|GBG12_MOUSE/Guanine nucleotide-binding protein G(I)/G(S)/G(O) subunit gamma-12 OS=Mus musculus GN=Gng12 PE=1 SV=3/1e-04/163-309/25-72 // sp|Q5RBQ0|GBG12_PONAB/Guanine nucleotide-binding protein G(I)/G(S)/G(O) subunit gamma-12 OS=Pongo abelii GN=GNG12 PE=3 SV=3/1e-04/163-309/25-72 // sp|Q9UBI6|GBG12_HUMAN/Guanine nucleotide-binding protein G(I)/G(S)/G(O) subunit gamma-12 OS=Homo sapiens GN=GNG12 PE=1 SV=3/1e-04/163-309/25-72 // 
ACAGAAAGGCGCATTGGATCTTGATTATTTAATCTCTTCAAAAAGGACTTACCAGAGTAGGTGAAGCTTAACTGATAAAGATAACCTCAAAGCCCCAAAATGAATAAATTCCAAGAAGGTCTTCAGCAACAAAGAAGACTGATTGAGCAGCTTCGGTGTGAAGCCCAAATAACACGTTTTCGAGTTTCTCAGTGCATTGATGACTTGCAGAAGTATTGTGATTCCCACATAGAGGAGGATTATCTCATCAGTGGCTTCAGTAAACCAAGTGAAAATCCATTCAAGGAAAAAGGCAGCTGTGCAATATTATAAACTGTTCATAACATTCGTAGATTTTAATGTGGCCTCAGTTTAAATGTGTTAAAGATCAGATTTTTTTTTGTGAATAAAATCTAAAAAAAAAATATTGACATTGGTAATGTCCTTTTGAATTAACAAGATATAGGCAAATTTTTTTTTTTTTTTTNTTTTTTACATTTTATATTAAATCTTTGATTAATTATTTGTATTTACCGTTTAACAAGTACTATTCTTTTCTCTTGTTTGTTTCTTTA
>Bg-c43126 len=107 count=2 IPR:/ blastx_SP:/
CGAAGAATATAATATTAGAGACAAGCTAACGGAGACTATAAGATGTTTGGAAAGAGATTCTCTAGAAAGGCGAACTATTGGTCTTAAGAAAATTGCTATAACTAACC
>Bg-c19557 len=576 count=4 IPR:/ blastx_SP:/
TTGATATAAATCACAGAGTATTTATTGATTACAATTTATAACTTGAACACCCAGGTAGAAGAGAAACAAAGGTAATACAACGTTTCAGCAAGGTCTCTATAAAATACTGTTTTTTGTTTCTTTAGCATTACCTCTAGGCAGGAGGAAATAAATACACCAGAAAATAAGATAGAAATTCAATGGCTAGCCTGGACAACTAATATTGATCTTACTATTGATACATATTCAATAATTGGTGTAAAACTTTGTGCCACTAAGCTATTTTAAAGTGTAATTCAAATATGAAATCCTACAGTTAAAGACATATGCCAGGACACATACATTGCAGTATTGCTCTTTATTGGAAACCTACAATGACAAATGAAGATAGATAGTCCTTGATGTCATCTGAAAGAGCAGCAGCATTTGAGTTATGTCAAGACAATACTGTTGCATCATGTAGGACAAAGCTGGCCACCCAATGCATTGTCTCTTAGTTCAAAGATTCTATATGATTTCAACAAACTGTAGACCACTAGAAAGATCCAGCCACAGATGTGCACAGTTTGTTAAGTGCACAGTTTGTCACATCTAAGT
>Bg-c25043 len=364 count=3 IPR:/ blastx_SP:/
TGCTGAAGTTGGCATGGCTCCCACAATTGAGGAGAAAGGTGGGGGAGAAATACCACAGCTGCACAAAGAAAAAAGATATCCAATACGATTGGAATGGTCATACAAAGAACATACACATCCATAAAGCTGATGAACAAACGCCAGGAAGGACAGGGATAGAAATGATAGAAGATCAAGACCATATTGAGTGTCCTCTACCTAGCACTTACAGATTAGATTTCAAGCTTCATTTCATGAGGGGGGGGGAAGGGGACCATGTTTTTTATTTTCTGTGTACACAAACATTCGTCATTTTTTGTTACATTTTCTTGTCATTTGGTTTTAATTATGTTGTAAATAAAAATACTTGTTACAAAATAAGTTT
>Bg-c23482 len=566 count=3 IPR:/ blastx_SP:/
ATTCATAAAATTAATTTTATTGATTATAAGAAAGACGGTATAAGACGGTGCAGAAACAGTGAAGTTTAGTACAAACAATTTTCGAACGAAAAAGTCTGTTGGTAGGCCTACTTAAAATGTATTAGTTTGAGCTTCTAAGTCATTTTCTAGATGAATTTTAATGAAATATACCTCCTGGTCTTTCATTTTTTAAAATACTTGTAAGGGTATAAATTGACAATATTATGTTAACGGTACAAGCATTGATTTACAACTGAAAGTAATATATTTAGTGGTAACCTTGTCAACATTTTTGTATAAAACAAAACACTTCACAACCACAAGGGTACGGAACCATGGCAGGCACATAGATTCAGATGTGGAAGCGCTGTATGCTACACAGTTTGGCCATTATAGACTCACATCAGGGCCGGCCTTAGGAAGTTGGAGGCCCTATGCGAAGTGTATAGGGTAGCCACAAATGAAATAGAAAACAAAAAACTAAAACCCAAAAATATGCAATGATTTAAAAACCTTCCTGTATTTATATCTAAATCAACAAATAAGTTAAATTCATATATTGCTGA
>Bg-c25714 len=326 count=3 IPR:/ blastx_SP:/
AACCTGGTGCGAAGGTTTAAAATGTTGGCAAAAAAAAAGCAACATCATATATTCTTAATTCCCTTTGCAAAACTTACTGAGAAGAACATGCATGTTGTTCCCTCATAAGTTTCTTGACAGAGGAAATCTTGTTTATATTAGATTTGAATTAAGCAAATTAAGAAAAAAATTATAATCATTATTTCAAATGGTTGTATCTAAACCTTATGAATTATTTTTCAGTTATAAGTATATTGAAATGAATGGATAATCATGAAATTGTTAAATAATTGCTGCTATCATTCCATTGAACTAAAGAACCTATTTAATTTTTTTTTTATAATTTA
>Bg-c23769 len=489 count=3 IPR:IPR020474:Toll-like receptor, leucine rich repeat-containing blastx_SP:sp|O15455|TLR3_HUMAN/Toll-like receptor 3 OS=Homo sapiens GN=TLR3 PE=1 SV=1/5e-11/289-17/24-114 // sp|Q9WVC1|SLIT2_RAT/Slit homolog 2 protein (Fragment) OS=Rattus norvegicus GN=Slit2 PE=1 SV=3/2e-10/376-5/246-376 // sp|Q9WVC1|SLIT2_RAT/Slit homolog 2 protein (Fragment) OS=Rattus norvegicus GN=Slit2 PE=1 SV=3/2e-04/280-20/31-117 // sp|Q9R1B9|SLIT2_MOUSE/Slit homolog 2 protein OS=Mus musculus GN=Slit2 PE=2 SV=1/3e-10/298-5/271-372 // sp|Q9R1B9|SLIT2_MOUSE/Slit homolog 2 protein OS=Mus musculus GN=Slit2 PE=2 SV=1/5e-04/280-20/31-117 // 
CTGGGAATGTTCCTTCAACCATGGAGAGGTGATTGTTGCGCAAATCTAAATGTGTCAAACTACCCAGGCCACAGAACGAAGTGTTGTATAAATGCGATATTTTGTTCCTGGCTAAACTCAAGCTCTTTAACTCTGTATAACGACAGAAGACATACCCAGGCACTTCTCTGATACCGTTATTGCCGAGTTTCAGGCTAGTGATATTAGTATGCAGATTAGTTGGGATACAGGTCAAGTTCAGATGCTGACAGTCACATTTGTCGTTATTGTAAATGCATTTCGTAGCATTGCAATTAACACTCGGAACCACTTCCGAGCAATTGCTTTCGGCGCGAAGCGAGGATTGAAAGGAAAGGTGCTTGCTGTCTATAACACTTCCGGCTGACGACATGCCCACACGCATTGCATGCACGAAGCTTCTCCTCTGGCGTGCATATTTCTGGGCTACTGTTTTTCTTGATGTGGGTTTAGAGTCTGGTAATAGTTTTT
>Bg-c16935 len=510 count=5 IPR:/ blastx_SP:sp|Q0P3X7|CV039_DANRE/UPF0545 protein C22orf39 homolog OS=Danio rerio PE=3 SV=2/1e-14/144-419/12-100 // sp|Q3U595|CV039_MOUSE/UPF0545 protein C22orf39 homolog OS=Mus musculus PE=2 SV=1/2e-14/144-419/11-99 // sp|A2BD89|CV039_XENLA/UPF0545 protein C22orf39 homolog OS=Xenopus laevis PE=3 SV=1/3e-12/144-422/11-100 // sp|Q5RE30|CV039_PONAB/UPF0545 protein C22orf39 homolog OS=Pongo abelii PE=3 SV=1/1e-11/144-419/11-99 // sp|Q6P5X5|CV039_HUMAN/UPF0545 protein C22orf39 OS=Homo sapiens GN=C22orf39 PE=2 SV=1/1e-11/144-419/11-99 // 
AGTACCAGTAACAGTTTTAATTTTACCCCCGTCAATCACTTGACTGTATATTAGAAAAGGCTGAAATAAACAACAAAAAGTCCTCTAGATACAAAATGACAGACCAACAAACCACAAATTTACCTGAGGATATTTGGCTAATTCGGCCTTGTGAAGTTTACAAAGAAGAGTACAAAGATTGTAAAAGTTTATTAAGTCGAGTTTATCAGCATTATGTTTATGGCACACAGCAAGACTGTAGTCAGTGGATGACAGATTTCAATAATTGTATGAAATTTCGGCTAACCAGAGATACAGAAGCTGCAGTATCTTTAGTTGCCATAGAAACAGAAAGAAGACAGAAAAGATTGCAACTAGCCAAGGACAATGATGTGTGGGAATACAGAAAAAGACCACCCCCTGAGTGGTTAGCTCCTCTAGAAACTAAATAAATACATATTTCTCATACTTTATATAAGTTTCCTAAATTATATAACAAATAACAAATAAAAGTAACAAAATAAAAAATAA
>Bg-c33603 len=322 count=2 IPR:/ blastx_SP:/
CTTAATAGAAGAAGCTAAGTCTCTCTGAGCTTGAACTGCCATTTCTGTCAGGTTTTGACTGTTGGTCATAAGCTTGTGAATCACAACCTCAAGTACAGCATTATCAGCCTTCTCGTCATTTTCTTTTTTTCTTGATCTGTGCGTCAACTACTCTTGGATCTATTTTGCTTTTCTTTTCTTCTGGTAATGTTACTGGTGTTTCTTTGATTGATGGAGCTTCTACAGTTTCACTTTTTTTTGTTTCACTCTGTGAGACTTGAACCTGATTTTCCTGAACATTCAGCTTTGTAGTCTTTTTCCAAAACCATTGGAAAATCTGGTT
>Bg-c5562 len=802 count=21 IPR:/ blastx_SP:/
TTGTTCTGTAATATTTATTTCCCCTTGGTACACAAAGATATAACAGAACTAAATGCTGACTAATAAATCATAACTAGTGTAATTAAATCAAAACTTGTGTAATTGGTAAATAAAAAGGAAAAACAACTGCCGTTAATTTACAAACCAGTGGATTTTTCACTCTGGTTTGTAACCATATTAAAAAAGAATCTACTTTTGACTTACCAAAACTTTATAGCTGATTGAATTATTCATGCACTTTTTTAATTAGCATAGTGTAACTGATTGATCTGGTAAAATTATCTGATCTCACTAAGTTAAATTTTTTTTTTTTTATTCTTGCATTGATTTCAGAATGTGAAACATCGTTTAAGGAACATACCAGTCTCCAGTGTGACCTGTATTAGAGTCTTTTACTGTTCTCTTTCTCTTGCTTTTTTATTTATTTTATACAATATGTGAAATCATTTATGATTGTAGGTGAGATTTGCTGAATTAGAAAAAATCCTGTGTACGGTATTTAAAGTTTAGTAATGATGAACATTGCCGCAAGTGTGCAAGTAGTCATCTTTTGGAGCTGCTTCAAAATGAAAAGATGTGCACTTAATGTTCATGATTATGGTATTTTAAATAGTCTTAATTTTTTTTTTTTTTTTATGATATAAATTATACTATTTACAATTTTTACCTTATCAATGCTGTAATTTAAATTTTCTTTTTTAAATTTGATCTGGATGCTACTGGTAGTGTTCTGTGCTCATTAAATATTGTTTCACATGAATTAAAAATCTAGGGGTCTTCTATTTTTTTTTNTTTTAGTTAA
>Bg-c38389 len=258 count=2 IPR:/ blastx_SP:/
TATAAGCTACATTCTGTCATCCTCTGTTTGATAGTCATCATTTGGTGTTCACATCTCTGTACATAGCAGAGTGATAATATAATATATCCCATGTAGATATGTAAGATTATATACAAAATGATACTAATAATCATGTTTCTGTGATTGTTCTCATAATATTGATGTATTGGTAGATGAAGAAAATGTGAATTTTTGTTTGTTTGTTGGGTTCTCTTTATTTTGTTTTTTTTTTTTAAAGCCCTTCTATTTTTAAAAGGT
>Bg-c27939 len=255 count=3 IPR:/ blastx_SP:/
GTGAATGTTCTTGTTAAAGATTGATTTGTTTAAGAAACAGATATTTAGGATTGCTTGCAGAGTGCATCTCTGTTTCTTGATGGAGATATTAATTTATCAGTGAGGACAAGGAGATATTAATTTATCAGTGAGGACAAGGAGATATTAATTTATCAGTGAGGACAAGCACTGACGTTTTGTAGAAAAAAAAAACAAATGCTATAATAACAAGACTTTCACGACATTTTAACTACTCATTAAGAGAGTAATCTTTGT
>Bg-c36677 len=273 count=2 IPR:/ blastx_SP:/
TAATTATTTTATATTTCTAAGTTGCCACTGACTCACAGATTCATTGGTCGTTGGTCAATAGCTTTTTCAAACTGTCTTACTGATCAACATGACATTTCTAAGAGATTGCTGAGAACCATTTTGACACAGACCTGACTATTATCATTTTCGAAAAATGTTATTTTCGGTTATTTCCCAAAAAGGCTAAATATATATCACTAATTCTTTTTCTAAGGCCTTCTTTTTCTTATTTTCTATTTTTAGCCGCAAAATGTCAGTCTGTTACACTGAGAT
>Bg-c11210 len=461 count=9 IPR:/ blastx_SP:/
CTTTCATCAAACACAAATGTTGGATCAAATTTTAAAAATGTGTTCATTTCTGTGCACAGCAATAAAAAATGTCAAGCTACAAAAAGAATTATTTTCACATTTTTCAAGAGGCTGGGATGGTCCAGTACACAAGTACTTAGGTAATACTAATGCCTAGCTGTCAAGATTCACCAAAATAAAAAAGGCTAATGTAGTATATGCTATATTCATAATGTAAATATTAATTTATAACAACCAGGCAGCAAGTGGCTTCCAAAGGTCTGGAGATTACTTACAAAGGCTGAAGGATACACATTTGAGACCAAAAGGGAAAGCTATTGCTGAAGACAGGCGTATACAAAAAATGTACTTAACAACATGATGACAAGGACTTTGTGTTAGATGTGGCAAAATATATAGATCACAGCTAGGAATGCCATGGGAAATACTGCACTCTTCCTTAAATTTCAGACTTGAGACAA
>Bg-c826 len=870 count=145 IPR:/ blastx_SP:/
ACTTTCCTGGATAAATATTGTAGCATGCTTTACATCAACATTCACATCGCAAACATCTTTTGTAAACAAGATATTGTCATAAATTTAAAACTCTTGTTGGTCAACTGAACAAAATGGCGAGTTTCACGTCCAATCCTGGCCGATTAGAAGGTAACAAGAATGTGAGTTACATGGGGATGTTGAGGCAATGTTTACCTCGCAGAGATCTTGCTTCAGAGGAAGCTAAGAAGATCGCGAGTCGTTACAGCATAGACAAGCCTGATTTCTACTTCGCTAATCATCACCAGGTAGGTAACTGGGCAGCTTCAGTTGGACTAGGCGAGTTCTCTCCCCTGTTTGTGCACTATTTAATCAACGGCAGACGACTTCTAACGCTCGACGCACTGGCGTTAGAGAAACTAGGCTCTAGAAATGTGGAGAAGAATCTAAGAATGGAAAATGCTATAAGAGATCTGCGGCACCAGCTTCTACGTGAGAGCGAAACGTTTGAGACTCTGCCTTACTTCACCACACAGGCTCTCAACCTGGGGCGATGGCCACCGCAGCGTTACCATCGTCAGCTGTTTCAGTTCACCGACCCTTGGCTAACGGAGAACTACAATTTCTTGGACATAAGTGCCAATGACCTTTGCCTGAGACTCAAGAACCATTACCCGCCGCGACCATACATGAACACCAGGGCGATATGACGTTAACCTCGCAGTGCGTCGCAATTTTGGAAATGTCATCACAAATGTCCAAATAGTAAATAGGAATTTATTACATTTATTTCTAGGATAAAACTTGCAATAATGAGAGAAATGGCAGCAATTGAAAGATGGTCTCGCTTCACTCAATATACGCTTTGTTTTTTGTATTTTTTAAAATAAA
>Bg-c10080 len=654 count=10 IPR:/ blastx_SP:/
CGTACACAACACTCACTGGTCTAAACGAAGCAGAACTTAGCAACGTGTCAGGCTCACAGGCTGCCGCTTTGAGTAAGTTCATCTCCGCAGACGTGCAAACGTTCACTGCCTTGCACGCTGCGACTCTGGGTCCAGTGGTCGCATTCAGAGCAGCACAGTAGTTCTCAAAGACAACTAGCTGCGTAGCGTTCTCGTTCGAGTCCACACAGCTGGAGACCTGAGCCTGGCATTTCATCGACGTCTTGAGCAGAGCGTCAGAGAATGTTTGATCTACGTCAGTCGCGGTGACTTCACACGCGGCGGATTTCAGCTGGTCGAATTCCGACTTTGTGCAGGACTGCGTTCCATGAGTGATGCACATCTCGGAAGTCGTCCCTTTCATGTCCAGGTTTAATAAAGAACAGAACTGTTCCTGCTGCTTCAGCACCACAGATGCAGAATTGGACTTCAGGCATGAATGTAGCTTTGCCTGACACTGATAGCTCTGGCTATTCAGAGCAGCATAAAAGTTGCGTCTCGCTCGGCTTATGGTGAGCTGTGTGTACGGGCGGCTCCTGTTACAAACAGTTTCTGTAAGAGTGTTGTGTTCGTTCTCGGTACAAAAACCCATCCTGACCAAGTACTGTGCCAACACCTTTAACCCTTCTTGACTGC
>Bg-c8268 len=643 count=13 IPR:/ blastx_SP:sp|Q95SS8|TMM70_DROME/Transmembrane protein 70 homolog, mitochondrial OS=Drosophila melanogaster GN=CG7506 PE=2 SV=1/1e-24/33-503/60-217 // sp|Q9BUB7|TMM70_HUMAN/Transmembrane protein 70, mitochondrial OS=Homo sapiens GN=TMEM70 PE=1 SV=2/4e-20/18-527/79-256 // sp|Q921N7|TMM70_MOUSE/Transmembrane protein 70, mitochondrial OS=Mus musculus GN=Tmem70 PE=2 SV=2/7e-19/36-515/80-241 // sp|A6H773|TMM70_BOVIN/Transmembrane protein 70, mitochondrial OS=Bos taurus GN=TMEM70 PE=2 SV=1/4e-18/30-515/79-242 // sp|Q5ZLJ4|TMM70_CHICK/Transmembrane protein 70, mitochondrial OS=Gallus gallus GN=TMEM70 PE=2 SV=1/2e-16/30-491/74-229 // 
ATCTTGGAGCCAGACAAATTCAATATTTCAGTAGCTCCTCTGTAGAGAAAGAAGATCTTGGAGAATTGGTTTATCAAGGAAGAATAGGGAGTATGTTTAGAGCCTTGAAAATATTTTCCTTGTCAACCAGTGCTATTGGACTCTGCCTTCAGCCTTATTTACTAATGACATATCAAGATACACCTTTAAAACTGGCAATTCCCATGTTTGCAGTGCTTAATGTTTTTGTTTTTGTCAACCCTATTTTAATTCATTTCATTGCCAAGAAATATGTCTTAGAAATGTACTTTAATACCCAAACCAAAGTCTTCACTGCCATTTTGTTGACTTTTTTTGCTAGAAAAGAAAAGATATCTTTCACAGCTGATGATGTTGATTGTCCAGATGTGCCAAATATGTTTGCGATGTTTACAGCCAAAGGTAGACCACTCTTCGCTCATGAGGCAGATTTCACAAGTTTAGAAGTTTACAAACATCTCATGGGCTTTGATGTTCCACTGGATTTGACCAAAGAAACCAAGAATCATTAAGTTATTGCTAATGTGTTTATAATTCAAAATTGGATTTATTTATGTATGGCATAATTCTAATGTTTGTTTTTCTANGTAGTTAACATTGTTTAGTTCAACATTTATTAAGTAAG
>Bg-c41841 len=214 count=2 IPR:/ blastx_SP:/
TATAGACACTATTGAATAGGACACGCATTGAAGCATTGAACTTAATAATGCTTATATTTTGTTAAGTGATATTAATACACATCCTTATTGTGCATGCTAAGAGCTGGAGTTTTGCAACATTAGATTAAGTCAACAACACTTATATATGTTGTCTTGTGAGAATAAAGTCCTATAGTGTTCCTAGTCTCTGTTTTGGAAGCCATTCAGAATGTAG
>Bg-c26347 len=296 count=3 IPR:/ blastx_SP:/
TTTTTTACCTTTACCTATCCCTTAGTCTGTTGGGGCACCAGGCAAGACTTGTCGACCGTCTTTCTCCGTTCCTCCCTTTTTTTGGTCCTTGTTTAGAACCTCTCTCAATGGCAGGTCCGTCCATTCTTTAATGTTGTCCTCCCATCGCTTTTTCTGTCTGTGTTCTCTCCCCTGGCACTGTTCCCTGAAGGAAGGTCTTTGCGAGCACCCGTAGATCCTGTAATGTGGTCATAGGTTTTAAGCTTTCGTCTTGCCAGGATTAGAGTCTCTTTTAATTTTAGGCTTGTCTTTGCTTT
>Bg-c16119 len=261 count=6 IPR:/ blastx_SP:/
TGGTAAGCAGAGCTGCGCTGGAGATCAAGATGATGGCCGTGCTGGGGTTCAAAACAAAGTCTTCAAGCATGTCTTTAACGATGTCCAGCCCAAGCTTGTAAGCTTTGAAAGTTGAAAAATGAGAAGAGCTCCTCACAAGAACGGTAACGCTGATGAATTGATTGAAGTTTCTGGACAGCCTATGTTCGTATCACTCGTATAGTCTTGTCACTATCTACACTAGCTACAGTTCCAAATCTTACAGCGAGTTACTTCACACTA
>Bg-c34775 len=296 count=2 IPR:/ blastx_SP:/
GGACCTCTTTGTGACCAGGTTTGCAGTAACAACTGTGCCGGGGAGGGCAAGTGTGACCAACACAACAGGACCTGTGTCGCTGGCTGCCTAGAGGGGTACGACGACAGTCGATGTGAGATCCCCCGGAAGCCCTTGATAGTTTACGCCATACTGATAGTCGGGATGCTGTTGACCGTGGTCATCTTGATCGTTTGCGTGGACAATAGATGAGTTCGCTGACGAGGAATCGTTTATGCTACCCAAAGGACGCAAGCTCCTCAGGAATGATGGGAACTCGAATAGAAGAACTTATGGCA
>Bg-c3287 len=525 count=39 IPR:IPR001841:Zinc finger, RING-type; IPR018957:Zinc finger, C3HC4 RING-type blastx_SP:sp|Q7L0R7|RNF44_HUMAN/RING finger protein 44 OS=Homo sapiens GN=RNF44 PE=2 SV=1/8e-14/388-161/351-421 // sp|Q4V7B8|RNF44_RAT/RING finger protein 44 OS=Rattus norvegicus GN=Rnf44 PE=2 SV=1/3e-13/388-161/269-339 // sp|Q3UHJ8|RNF44_MOUSE/RING finger protein 44 OS=Mus musculus GN=Rnf44 PE=2 SV=1/3e-13/388-161/326-396 // sp|Q9H0F5|RNF38_HUMAN/RING finger protein 38 OS=Homo sapiens GN=RNF38 PE=1 SV=4/4e-13/388-161/434-504 // sp|Q08CG8|RNF44_DANRE/RING finger protein 44 OS=Danio rerio GN=rnf44 PE=2 SV=1/7e-13/388-161/367-437 // 
TATTGATAGCCGTTCAGTTTAATGAGTTTACTGTTGATGGATGTCGTTATATAAGATAAGCATCTTTTTACAAAAAGAACATTCTTAACTCCCAGCGCCATCTTATAAATGAACATTAGATGTGCAAGTACTGGCCAGGTGTTTACTACTTGACTAGATGTCGGCAAACAGGACAGGTGGCGTTCATCCTGAGCCATTTGTCACCACACTCTGTATGGAAATTGTGAACACAGGGCAATACACGCTTCTGGTCACCCGTCTCAAAATCCTTCAGACAGATTGAGCATCGAACATCTGAAGAGGCCTCCTCTCCGTCGGAGATCCTGTCCCTGTTGTGCACGACCGTTGGGAGGACATCAATTTCTGACTTTGTCAAACGCGGAGGTTCCGGTGTTTCAGGCCTTGAATCAATGCTATGAATTGAGTAGCCCAATATCCCAATGCGATATCCAAGTGCATCGTCTCTGAGTGTATACCATTCCTCTTCTTCATCATTGTCAGATTCTAATGCAAACAGGACCTGAA
>Bg-c17987 len=325 count=5 IPR:/ blastx_SP:/
ATAACATTATTATATAAATGAACAAAATCTGTTAGTTAGAAACAGACTAAATGAGAAAGACTAGAGCTCATACACAGAGACCAGTGATTTACGAGAGTCAAGTTTAATGGATGGACGCGTGGCTGTAAGTTTACGCATCGGACAGCCCCGAGCGTAAACCTCGCCGGCTGCCACCCACTGCCGTCCTGCAGTAGGCTTGGGCTAAGGCGTATAAAAATCAGTTCTGATAGAATTTCAGAAACATAACTTACAAAGAAACATAAAAGTAGAAGTTTTGTGTCTACTGATTAATAGAACCCTAAAAAAATGGACATTTACTTGCACA
>Bg-c11316 len=399 count=9 IPR:/ blastx_SP:/
AATTTGATGTAATTTGTCACTGCGTCGACCCATTCTTCCAGATTTGAAGTGGTCTCTTTTAAAATAAATTCCAGTCAGTGCAGTCTAGACATCCCTGTAGTTTGAGAATATTGTCTTGAGTCCATTCTTGTACGTTTTTTTTGGAATGATTTTTCCTTCTTTAAGTTTCGTACGGTAGGTAGGCAGAAGTTGTATTGTTTTGTGGTCCGATGATCCTAGTGGTGGCCTTTCACGAGATGTGAATGCTCCTTTGATGTTACAGTAACATAAGTCCAGAGTTCTGTTCTCTCTTGTCGGACATGAGATGTGTTGATAGAAGGTGGGTAGGTCAACCTTCAAAGTGCATTTATTAAAATCACCCAGTATAACTACTGGTGCGTCCGGTGACCTTGTCTGAAA
>Bg-c19723 len=517 count=4 IPR:/ blastx_SP:/
TCCCTGTCCAAGACAACCACATTTTAAGTGGAGACATCCTTCTTCCCTTTCGCCAACAAAAATTGTTCTTAGAAAAGAAACCATAGGCAGTAAAACCACCGTAGAGTCAATGGATCAAAAGCACAAAAATGATGAAGGCCTTACACACATTTCAAAAGATGACACAGATTTAGAACAAAAATAATTGTAGAATAGAAAAATCCTTCTACCTCTAATTGTCCATTTATTTCTTTAAAATGTAACTGTCAACAAAAAAGGCCCAAGACCAGCATGAAAGTTAAAGAATATACCAATACTAGAGTATGACTGCTGCCTTCATCAACATCCAATCAAGTGAGAGGCATGCAGTCATGCAGAACTTTTGAAACTATCCAAAGAATGAACGAATAGGTGACCTTATAGGACTGGAAAGGAATTGATGCTGACAACAAGCAAGGACTATCTAGAGACGGAGACTTGCAATATTCAAAACTTCCTATTATAAAAGCAGAGAATACTAAAATGATAGATAAGCCTC
>Bg-c2658 len=1574 count=49 IPR:IPR020472:G-protein beta WD-40 repeat, region; IPR001680:WD40 repeat; IPR019782:WD40 repeat 2; IPR017986:WD40-repeat-containing domain; IPR019781:WD40 repeat, subgroup; IPR015943:WD40/YVTN repeat-like-containing domain blastx_SP:sp|Q0III3|DC1I2_BOVIN/Cytoplasmic dynein 1 intermediate chain 2 OS=Bos taurus GN=DYNC1I2 PE=1 SV=1/2e-159/1-1146/226-607 // sp|Q5NVM2|DC1I2_PONAB/Cytoplasmic dynein 1 intermediate chain 2 OS=Pongo abelii GN=DYNC1I2 PE=2 SV=1/6e-159/1-1146/252-633 // sp|Q13409|DC1I2_HUMAN/Cytoplasmic dynein 1 intermediate chain 2 OS=Homo sapiens GN=DYNC1I2 PE=1 SV=3/6e-159/1-1146/252-633 // sp|O88487|DC1I2_MOUSE/Cytoplasmic dynein 1 intermediate chain 2 OS=Mus musculus GN=Dync1i2 PE=2 SV=1/8e-159/1-1146/226-607 // sp|Q62871|DC1I2_RAT/Cytoplasmic dynein 1 intermediate chain 2 OS=Rattus norvegicus GN=Dync1i2 PE=1 SV=1/3e-157/1-1146/252-633 // 
ATGGATGGCAAAGATGATGATAGTCTAGCTGGGGAGAGATTAAAATTGCAGTTGGAGTTTTTTGATGAGAAGTGGTCTAAGCGCAGAACTATCACAGGAATGGATTGGTCACCTCAGTATCCTGAGCTCTTGTTGGCTTCCTACAATGCCAATGAAGATGCACCTAATGACCCTGATGGAGTTGCTCTCATCTGGAATCTGAAGTTCAAAAACATGGATCCTGAATATATATTTCACTGTCAGTCTTCCGTCATGTCAACATGCTTTGCACAGTTTCACCCCAACTTAGTTATCGGGGGAACATATTCTGGTCGCATTGTCCTCTGGGACAACAGAGTCAACAAAAGGACACCTGTGCAACGTACACCTTTATCTGCCACATCACATACGCATCCAGTCTACTGTGTGAATGTCGTCGGTACCCAAAATGCCCACAACTTGATCAGCGTGTCAACTGATGGTAAACTCTGCTCCTGGAGTTTGGATATGTTATCACAGCCCCAAGACAGTATGGAGCTCCAGAGCAAGCAGAACAAATCTGTGGCTGCCACCTGCTTCAGTTTCCTGTCTGGAGATGCCAACAATTTCATTGTGGGTTCAGAAGAATGCATAACATACTCTGCTTGCAGGCATGGAAGTAAAGCCGGTATAAATGATGCCTTTGAAGGTCACCAAGGTCCCATCACCAGCATAGATACACATAAAGTTCCAGGACAGATTGATTTCTCACCATATTTCCTAACATCATCTTTTGATTGGACTATCAAACTGTGGAGCATTAAGCAACCTTATTACATCCACTCCTTTGAAGACAACAATGACTATGTGTATGATGTCAGGTGGTCTCCCATTCACCCAGCGTTGTTTGCCAGTGTGGATGGAGAGGGACGTTTGGATTTATGGAATCTTAACAGTGAAACTGAGGTCCCCACTGCCAGCATAACAGTTGATGGCCGTCCAGCCCTGAACCAGCTGAGATGGCACCAGACTGGACACCATATTGGTGTTGGAGATAATAATGGACGTATTCATGTGTATGATGTCGGAGAGCACATTGCTAACCCAAGGTCAGATGAATGGTCCAACTTTGTACACACCCAACAAGAGTTAAAACAGCATGCTGCAGAAAGAGAAGAGGATTCATCACTGGCTGGTTCTGGAGCGCCTCTAAGATAGATCTTTATTTTTAAAAATGTAGCTTTATTTATCCAATTCGAATGTTCCTGGACATGGGCTGTGTAGAGATTTTCACCCTTTAAGTAGACTTTTCCCCCCTTTTTTTAAATTAAGAAAACATTTTCTTGTCATGTTGCGTGATTAAAGCTGCTATGTTTGGTGGTTTTCTTGTTAAGTTAAGCTTTTATTGACCTTGGATAGTTGTAAGTGCAATATCGTATATTGATAGATGAAGTATGAAATCTGTTAACATATATGTCTGTTTGTATTGTGCTATTGTGAACAAAGTATAGTGATAAGTCACACACTATGTAATCGCTTGACCTATTGTTTCAGGTATTTTCATAACACACAAAGAAAACTTAGTACAAATAATTTTAGTTTACCTCTACTTTGTT
>Bg-c24785 len=382 count=3 IPR:/ blastx_SP:/
CAAAAAGAGATCTAATTGTTTCACGATAAACATGAGATCCATCCCACTGAGAATGAACAGCTGTTTCTTATTGACAGTGTACTCGTGGATCGCGTTAAGCACATGTCACGTGGTTGACAGATTCAGCGTCGGTCAAAACGAGTCTTTTACTGTGAGATGCACCATACAGAAGTTTCGAACAGAGGACTATCCCGAATCCCCCTATGTCGTGACGTTGGCCGCTGAGAGGAGACCCACATGGGAAGAGAACTTTCAACTCATGGCCAAGTATTCTCCGTTCGCTCCCTTGGAAGAGTACAGGAAAGTCACGAACTTACCACATAAACGCAATTGGAACTTTCAATTCCTAAATGGCGACTCATTCGATAACAGAGACAAAATC
>Bg-c39387 len=250 count=2 IPR:/ blastx_SP:/
TATTGAAACAATTTTTATTTTTAAGGAACACAACAATTCCAAAGAATGTATACACCTCCATGATAGAAATATTCATTTGAAATATATATCACAATGTCAAATAGTATAAGTATATATGAAGACCCTAAAAATATATATATTATCACAACATAATTAACAAAAACTAGGGGTGTGTGGATGTCAATGTTCACATTTCCTAAACTGGTTCAAACGTGAGAGAATAAAATGAAGGGGGATACAAAACAAAAAA
>Bg-c39873 len=246 count=2 IPR:IPR003108:Growth-arrest-specific protein 2 blastx_SP:sp|Q8NHY3|GA2L2_HUMAN/GAS2-like protein 2 OS=Homo sapiens GN=GAS2L2 PE=2 SV=1/1e-19/244-2/160-247 // sp|Q5SSG4|GA2L2_MOUSE/GAS2-like protein 2 OS=Mus musculus GN=Gas2l2 PE=2 SV=1/1e-18/244-2/160-247 // sp|Q99501|GA2L1_HUMAN/GAS2-like protein 1 OS=Homo sapiens GN=GAS2L1 PE=1 SV=2/2e-18/244-2/149-249 // sp|Q8JZP9|GA2L1_MOUSE/GAS2-like protein 1 OS=Mus musculus GN=Gas2l1 PE=2 SV=1/6e-18/244-2/149-249 // sp|P11862|GAS2_MOUSE/Growth arrest-specific protein 2 OS=Mus musculus GN=Gas2 PE=1 SV=1/3e-05/244-2/159-244 // 
GTAAGATCCTGACAAAGATAAGACTGCGGGAATCTCCGATTTTGTACTTGCCCTCACCGACTTTCACCATTGGGAACTGCACGGGACACGTACACCGCCGTATGAGATCACGAACCCTTTCATCCAAAGACCGCATGTCACATGTCTGAATCTGTGGCCTCGGCAGTGGCGGCTCATCTCCGGCAAGCTCTGCGTCTATCTCCTCCTCCATCTGGACCAGCGTGGGAGCCAACATCCCGAGTTTGG
>Bg-c39505 len=249 count=2 IPR:/ blastx_SP:/
TAACGCCCATCCTATACATTTATTGAATCTCTTCGTAAGCGAATCCTCCCTTGCTCGATCTCATTTGGATTCGGTCACGTCTTCTCCCTGACCTGTCACTTCTATATGTATCTGCACGCTTACAGTTGACCACCACGAACGTTCGTTGCTGATCCAGAACTTATAAATGCCTTCCTCTTCAATCTCTATGCTTTCTTCCAGCGGGGTCACAATCGGGCCTGGCATCCATGGAAATGTTGGTACTATTTG
>Bg-c3906 len=609 count=32 IPR:/ blastx_SP:/
GACTTGGATCTACTGCCTGGATCACATCGTGTCTCAGTGCTTGTCTACTTTCTACAATGATTGTGTGTTGTCTAAACAAGTCTAAAGACTGGACAAGTTCTCAGTCATGCTGACCTTGTGGTGTGACTGATCACCAACACCATCAAAACAAAATGGCTGTCATTTGGACATTCAGTCTCATTGTTGCTTCTTTACTTTCTATTCAGATGACTGGACAGACTGTTTCAGCTGCCTATCTAGTCATCAGCGCCTCTCCATCATCAATTCAGCCAATATTGACCACTACCCTGAAACTCAGGTGTTCCATTAAGTTGGATAATACACTCAACGCTCGTCAGATACATGGGAATCTAGGCGTGGAACAGACCAACACTGAGCCACCAGCCTTGGCAGACGTGGATCACATCGTGTCAATTATCTTGAAGAAATACAACTCTGTCACCTTGACGAACGAAGCTGTGGCCAATATCATCCCTTACGACAAGCCAGTGGCCGAAGGCTCTTACGCCAATCTGGTCCAAGTAGATGGCAACACAGACAGTTCTCCTGTGCCTGGGAAAAGGGGACCTCGAAGTCACGTGTTAAGCCATGGAGGAGGACGTTAGTGTG
>Bg-c22408 len=248 count=4 IPR:/ blastx_SP:/
TTGTGTCTGTTAATAAAGATTGGCTAGTGTCATTCATTTTGAATGTAAAGCTTGACACACAGTCTATGCTACTGCTAAAGTTAAAACTAGTATCATATCTTAAGAGATCATACTTTATAGGGCTACTCTGGTAAGTGGTGTGCATAGATTGATATGACCCTGGCTAGTAAGCTGAATTGTTTTTACTTTGCCTGGTAAATGCATTTCAGAATCGTGTACAATGCAGATTTGTACTTTCAGAAAATTAA
>Bg-c19446 len=615 count=4 IPR:/ blastx_SP:sp|A2AVQ5|CA177_MOUSE/Uncharacterized protein C1orf177 homolog OS=Mus musculus PE=2 SV=1/3e-12/608-108/228-401 // sp|B1H283|CA177_RAT/Uncharacterized protein C1orf177 homolog OS=Rattus norvegicus PE=2 SV=1/3e-10/608-108/216-389 // sp|Q3ZCV2|CA177_HUMAN/Uncharacterized protein C1orf177 OS=Homo sapiens GN=C1orf177 PE=2 SV=3/4e-07/608-108/232-405 // 
AGTACTAAAAACCTGCCCGTATAGTGGCCATCAAATTTTTCAGCTATGAAAAAGCCCCTTTTTCAGATTATTGTGATAGAACGTGTCATTCTGTAAGGATCCATGTATTTGGCTTTGTCTGCTGGACTTATATCTTTAGCTCGTAGTCTTTCTTGAAGAAATCGGGACAGTTTTTGATTTGGTCTATTTGTTCTAGATTTGAAAGAACTTGTGCATCCATTAACATCTTGAGCCTCCTCATATTTTTGTACATCATACCTTCCAGCGCCAACAGGATTATAGTTCCCAGTAAAAAATTTTTGAGATAATTTATCATCACGTTTTGCTGATGAAAGAAATCCTGGAATGTTGACGCCATGAGAGCATTTACTGTTGTGTTGTTTTACATCATAATTTCCAGGACCTGGAAAATCCTGTAGAAATGTATCTGGATACTGATCAACTTTAGCAAATCGTCCATGACTTCTTTTGTGCTCATTATTTAATTCATCCACAAAAGATTTGACATCATATTGTCCAGGACCTAGGTTTGCTGGTCTCACAATTGCATGGTGGCCTATGACTGTTTTTGCATCTCTTTCTAAAGAAAACAAATTTGTAAGGCCCCTTTATACC
>Bg-c7542 len=389 count=15 IPR:/ blastx_SP:/
CCTCACTTTGTCCTGTGCTGTTACTTGTGTAGATTTCATCAAAAACAAAAAAACTTAGCACATTACCCAAGTTGTTGGTTCTATTTTAATTAGCACAGAATTTTGTTTTATAACACTTCATTACATCATACTTGGCTTGGATGAGCAATTGCCTTTAGTATTTGAACTTTGCTGCAATGATTCAAGTTATTTCAGAAGCCAATACAAATAGAGGTATTGTTATGTCATTGTCTCATTTCACACTGTTCATATGCTTTTGATATGGTATAGAATTTTACTGAAACTGTAAATTTAGTTTTTTTTTTTTTTTTTTAACTTAACTAACTTACCCTTTTTCCGTTTCGTTCGTTAGTTAAATTTTAAATTTTAATTTAAGTTAGGTTGTTTAG
>Bg-c931 len=835 count=133 IPR:IPR000668:Peptidase C1A, papain C-terminal blastx_SP:sp|Q26636|CATL_SARPE/Cathepsin L OS=Sarcophaga peregrina PE=1 SV=1/6e-89/157-783/130-339 // sp|Q26636|CATL_SARPE/Cathepsin L OS=Sarcophaga peregrina PE=1 SV=1/6e-89/3-74/72-95 // sp|Q95029|CATL_DROME/Cathepsin L OS=Drosophila melanogaster GN=Cp1 PE=1 SV=2/1e-84/160-783/163-371 // sp|Q95029|CATL_DROME/Cathepsin L OS=Drosophila melanogaster GN=Cp1 PE=1 SV=2/1e-84/3-161/103-163 // sp|P25784|CYSP3_HOMAM/Digestive cysteine proteinase 3 OS=Homarus americanus GN=LCP3 PE=2 SV=1/3e-83/160-783/115-321 // 
ACAGTTTCTACCTCGGAGAGAACGCCTATGCTGATATGTCAAATGAAGAGTTTGTCAAGACCATGAATGGCTACCGCGGACACAGTGGCCAGGGAAGTCACTTGATTCACGTTGGTGGAGATGTGAAGGACCTCCCAGCGGAAGTGGATTGGAGAACAAAAAGGCTACGTCACAGACGTTAAAGATCAGGGTCAATGCGGGTCATGCTGGGCTTTCTCAACCACCGGGTCACTGGAAGGTCAACACTTTAAGAAAACTGGAAAACTTGTTTCTCTGTCTGAGCAAAATCTGGTCGACTGCTCACAGAAGCAGGGTAACCAAGGCTGTAATGGCGGTCTAATGGACCAGGCTTTTACCTACATCAAGGTCAACAATGGTATCGATACCGAGGCTTCCTATCCATACGAGGGTGTAGATGACACCTGCAGGTTCAAGGCAGCCAATGTGGGCGCCAATGACACTGGTTATGTTGACGTCAAGTCCAAGGACGAGAGCGCCCTTCAGGATGCTGTTGCCAACGTGGGCCCCATCTCTGTTGCCATAGACGCCAGTCATGCCTCCTTCCAGTTGTACTCTGGTGGAGTTTACCACAGCATTTTGTGCAGTCAGACCAGACTTGACCACGGTGTGCTTGCAGTGGGTTACGGCAATGACAATGGCAAGGACTATTGGCTGGTCAAGAACAGCTGGGGATCTTCCTGGGGTCTCCAAGGCTACATCATGATGTCAAGAAACAAGGACAATAACTGCGGCATTGCTACGGCTGCCAGTTATCCTACAGTTTAAATTACACTTTGTATATGGTCAATAAACAAATGATTTCAAACGGCAAAAA
>Bg-c21989 len=269 count=4 IPR:IPR002627:tRNA isopentenyltransferase blastx_SP:/
CGGCTCTTCAGACAAAGTTTTGGACGAAATGCTGAACGAACTTCTCGCCAAGAGTTCTGCAGAGCTTTACGCAGAGCTAGAGCGTGTGGATCCCGAGGTTGCAAAACTACATCACCCAAACAACAAAAGAAAGATCGTCAGGTCAGTTGCTGGAGTTACTTTATGAACGTTTTAGTTACCACCAAACTACGGGATATGAATATTTAAACAAATTTGTTTCTTTATGCTTTAAAATGTGTTTCATTAGAATTAAATGTCCATTCCTGGAA
>Bg-c41813 len=215 count=2 IPR:/ blastx_SP:/
CCTATCTAAATGTCTTAATGGTTTGAGTTCCTGAAACTATCATTACTATTGCCAAAGCAGTGGCATAGCTAGGGTTTTTTGATGCCCAGAGGGGCAGCTCCTCATGATGTCCTACAAAATAAAAAGTAATGAAAAGACTTTGTTCACAAAATGATATGTATTGTAATTCGTTGTTAAAGTATATATTGCCTATTCATAAAAGCTAATTTTCCATT
>Bg-c40388 len=241 count=2 IPR:IPR020683:Ankyrin repeat-containing domain blastx_SP:/
ACCAGGACTTCACTAGTCGATGCACCTTAAAGGGCGTGGCGTCATGAACAGCTGCCCAGTAGGAAGGGGTTAGTCTACGCACAAGTTCCATCATCTCAGCCTTGGATACGCCTTTGACCTGCTCCAGCCAGTACTGGTCATCACCACTGAATTCAGCACCGCACTTTAACAGTGCAGCCAGAATCTGTGGAAGTCTTCTGGTGACGGCGTATCTAACAGCTGTTGTTCCAGAATTGTCTAG
>Bg-c41050 len=233 count=2 IPR:/ blastx_SP:/
TGTGGGACCATAAGGCTAAATTGGAAAAGCTGCGAGAAAGATGCAAAAGAAAATCCATAATATTGAACGAGAGCAAAACATTGGAACAAAAAGAGGCGGTCGAATTCATGGGACACGTCATCAGTGCACAAGGCATCAAACCAGACCCCAAGAAGGTGCAGTCCATAGTGGACATGGATCGCCCTAAAACGCGCAGTATGGTGGTATGGTGCAGTACCTCAAGTCCGACGATT
>Bg-c11141 len=495 count=9 IPR:IPR016044:Filament; IPR001322:Intermediate filament, C-terminal blastx_SP:sp|Q01241|NF70_LOLPE/70 kDa neurofilament protein OS=Loligo pealeii PE=2 SV=1/3e-37/490-5/428-580 // sp|P31732|OV71_ONCVO/Muscle cell intermediate filament protein OV71 (Fragment) OS=Onchocerca volvulus GN=OV71 PE=2 SV=1/2e-24/490-2/256-396 // sp|Q17107|AV71_ACAVI/Muscle cell intermediate filament protein AV71 (Fragment) OS=Acanthocheilonema viteae GN=AV71 PE=2 SV=1/2e-22/490-2/218-358 // sp|P23730|IFEA_ASCSU/Intermediate filament protein A (Fragment) OS=Ascaris suum PE=1 SV=1/4e-22/490-2/321-462 // sp|Q19289|IFB1_CAEEL/Intermediate filament protein ifb-1 OS=Caenorhabditis elegans GN=ifb-1 PE=1 SV=1/1e-21/490-2/412-553 // 
GGAAAACCAGATCATTAGGTCCAGCCTGATTGGTTTGAGATCTGGCAAAGATCTTCACATTTTTAGCTGGTTTAAGAATAAAGTTTCTGAATGTATACTCGTAAGGTCTGCTCATGTTTTTGTCCACAATACGTCGTATCTTCCAGCCATCCATGTTAATTTCCTTTCTAACACCTCCTGAGGGGTTGTTCTCCAGAAGAATAAATTTGCCTTCTGGGTTGGCCTCAGCGATGCTAACAGGTCCATTGGCTGTCCTCTGGTAAGTGGTCTTGGCAGACACTTCCCCTCTCATCATCTTCATGGAAAGCTGAGAGTCAGCACCAGATTCATAGGATTGGCCCTCAGTGATGTTAATTATGTCCGCCAATCGAGCGCTTCCGCTTTGCCTGACCCCAAGGGTTTGTTCCACAACTTGTCTCAGACCGACCCTTGTCTCTTCCCCCTCCAACAGTTTTCTGTAGGCTGCAATCTCCAACTCTAGGGACAGCTTGGCAT
>Bg-c16550 len=678 count=5 IPR:/ blastx_SP:/
GCTAGTTGTGTATCTGACAGACTCAACAGGCAACAGGAAACAGTATTTGTTAGCAGCTGACAATGCTAAAAGAATTCATGTGATAAGTAATGGAACCATAGTCAATTCTTTAGTTGCACCAGTTAATATTACTGCAATGTGTCAAGGCAGATTTATTCCATCCAATAAACTCAGTTTAGCCTCAAGCCCAACACTCTCTACAGATGGTGAGCAAGTTGCCTTAGGATCAAGCAGTGGTTCCGTTTACATTTTACATAACTTTAATATAACTCTGGATGACTATGTTAACACCAAAAGTCCCATAACTAGCTTGTGCACATTACCTCAGTCTGACCACAGCACTGATCTTCTTTTATGTGCTGGACATTTCAGTTCACTACATTTATATCGTGATGGCCAGCTTTTTTATGAATACCTAACAAGTGATTGGGTCAACAATATAGCTACAGCTGACATAGACGATGATGGTGTACTTGAAGTTATTATTGGATGTATGGACAAGACAGTTGTGGCTTTGAAAGTTTAAATGTCTTGTGTATTTTGTGTACAATGCCTGATTTTATTGTACACAGAATATATACATATCTGTAAGAATTTCACTATTGTTTTGTTTTAAAATGTGTTCACCCTAAAAGGGTGTATCAAGAAACATTAAAAGTCAAGTATGTAACTGTTAAA
>Bg-c35060 len=292 count=2 IPR:/ blastx_SP:sp|Q4KLN7|ARFG3_RAT/ADP-ribosylation factor GTPase-activating protein 3 OS=Rattus norvegicus GN=Arfgap3 PE=2 SV=1/1e-04/11-133/485-524 // sp|Q9D8S3|ARFG3_MOUSE/ADP-ribosylation factor GTPase-activating protein 3 OS=Mus musculus GN=Arfgap3 PE=2 SV=2/1e-04/11-133/483-522 // sp|Q5R787|ARFG3_PONAB/ADP-ribosylation factor GTPase-activating protein 3 OS=Pongo abelii GN=ARFGAP3 PE=2 SV=1/1e-04/11-133/476-515 // sp|Q9NP61|ARFG3_HUMAN/ADP-ribosylation factor GTPase-activating protein 3 OS=Homo sapiens GN=ARFGAP3 PE=1 SV=1/1e-04/11-133/476-515 // sp|Q3MID3|ARFG2_RAT/ADP-ribosylation factor GTPase-activating protein 2 OS=Rattus norvegicus GN=Arfgap2 PE=2 SV=1/1e-04/53-133/492-518 // 
TCGACAGTACAGCAGCGCCACACCTGATCTGCAGGACATTAAGGATGGGGTGAAGCAAGGAGTCACCAAAGTAGCTGGTAAAATCTCCAGTCTGGCTAATGGAGTTATGTCCAGTTTACAGGATCGCTATGGATGACCACAAGGTGTTAAGGACGCGGACAATGTATATGACCATAATTTCCACCAAGTTCTTTATGACCCCCATTCTGACGGTCCATGCTTTCAGCATGTGGACGGACAGCTGGGCCAATGGCGGGAGAATATGATAATTTATATTTGATACACATTGTTG
>Bg-c27440 len=266 count=3 IPR:/ blastx_SP:/
CGAAGTCTCTGACGATCGCCGCTGGAGTATTGTCTGCGACTCGGAGGTGTTTGTCTTCTGCGGCGGAGGCTGCGCGTAAGCCGGCGGCGACGGGTTTTCCGGCGATGAGGAGTGAATATTCCGAGGCGACGGAAAAGACGTACTGGATGAGGGATCCGAAGACGGGGTGCTGGATCCCGGAGAATCGGTTCGGGGAGGTCGACCCTGCCGATTTGAGAGCGAAGGTTATTTTCAACAAAAGAGATGTGACGGCCGCGGAATGTTGA
>Bg-c38759 len=255 count=2 IPR:/ blastx_SP:/
TGGTGAATCATCTTTAGTTTCATCATTCTCTTTTGGTGAATCATCTTTAGTTTCATCAGCCCCGCCATTTTCTGTGTTCGGGTCGTCGCCGAACATTAAATCGCTCAAATTCTTGAGGGGTCCTCCAGATACATCTGTCTTCTTCGCTCTCGGCTGACATCCTGAGAAATACTGTAACTTCTCAGCCGGAAGTTCTGAGCAAGGGGAACAAACGGTATCACTTTTAGTTGAGCAGTCTTGGTGTTTGTAATAACG
>Bg-c36376 len=276 count=2 IPR:/ blastx_SP:/
AGCCTAACAATGGCAGTTCTTACAAGTTTGACCAGTGACTCAGTGTCAACTACAGTTTTTATAAATCCATTAAGAAGGATGGACATTGGTTGTCCTTGTGCCACTTGACCAAATAATTTATTTATAAAAGAAACATGATTAAAAAAAAACAACTGACTGGCATAACTTCAATACTGGAATGTCCAATAGAGTTTAGGCTTCACATTAGAAGCACATTATTTACTGAAATTAAAAAGTTATTTGCAATTGATCTGTTTCCCATAATGTGGATAGGGG
>Bg-c6230 len=271 count=19 IPR:/ blastx_SP:/
TTACTTCTGTTTGTTCAGTTTATTTCTTTATCAGTGCAGTGACATAGACTTTGATGATGAGCATTGATGACAGAATAAACTTAACATGTTATGAGCATTGATGACAGAATAAACTTAACATGTTATGAGCATTGATGACAGAATAAACTTAACATGTTATGAGCATTGATGACAGAATAAACATAACATGTTATGAGCATCGATGACAGAATAAACTTAACATGTTATGAGCATTGATGATAGAATAAACTTAACATGTTATGAGCATTGA
>Bg-c41340 len=228 count=2 IPR:/ blastx_SP:/
TGGAGTATCAGATGAATTGGTACTACAGTTTCTCAGTGGCTTACATCTTGTGAATCATGAAACAGCAGCAGAAGATGAGTAACAAAGTGTCCACATCACAAAAACAGCAGGACGCCACGTCTACCAATTCATAGACATCACGTGTGTTTCCGTAGTTTTTCCGTTTGCCTGGCTGAATGAGGTGTGTCTCTTCTTTCTGAGCTGGAAGATGTGGGATCAGAGGTCGTT
>Bg-c23102 len=715 count=3 IPR:IPR002138:Peptidase C14, caspase non-catalytic subunit p10; IPR011600:Peptidase C14, caspase catalytic blastx_SP:sp|Q5IS99|CASP3_SAIBB/Caspase-3 OS=Saimiri boliviensis boliviensis GN=CASP3 PE=2 SV=1/4e-07/1-168/222-277 // sp|Q8MJC3|CASP3_RABIT/Caspase-3 OS=Oryctolagus cuniculus GN=CASP3 PE=2 SV=1/8e-07/1-165/222-276 // sp|Q2PFV2|CASP3_MACFA/Caspase-3 OS=Macaca fascicularis GN=CASP3 PE=2 SV=1/1e-06/1-165/222-276 // sp|Q95ND5|CASP3_PIG/Caspase-3 OS=Sus scrofa GN=CASP3 PE=1 SV=1/1e-06/4-165/223-276 // sp|Q5IS54|CASP3_PANTR/Caspase-3 OS=Pan troglodytes GN=CASP3 PE=2 SV=1/1e-06/1-165/222-276 // 
CTTCTAAGTAAACTTGGTAACAAGTTGGATTTGATGACAATGATGACAAGAGTCAACAAAATAGTAGCAGATGAATTTGAAACAAAAAATAAGGCTGAGTTTGACAAAAGAAAAAAGCAAATTCCATGCATAACATCAATGTTGGTGAAAGATTTCTATTTCTTCAAAAGTTGAATTGATTTATTATCAATATATGCGTAAGTGGCCCTACAGAATATTTTACTGTAAACAATATAGTTTTGATAGTGAATCTCACTAAGGTTTATAAAAAAATTTACAATCAGATCTTTATATACTCATCTGTAGTTTTTTTTTTTAAAGACTTCTGTAAATTTTAAAGCATTTTTTAAGCAAGTTAATTATATCAAAAGAAACTTTAGAAAAAAAAACGATGCTATCATGATTAAGAGGTATTTATTTTTACTGATTATTATGAAAATCTTTTTTTTAACATAATTCAGCATTTCCTGAGTAGATGTACATTCACAACAGTAAATTGTTGAAACATTTTGATCTGACTGTCTAGAAGTATATATGTTTTTTCGTCAAGTCTAATGTAACTGTAAAATGTATAAAATATTTTTAAAAAAATACTTCTTTTATTTTACTAATGCAACTTTTCGCCTGACTGCCGAAAAAAAATCCACAATATGTATTAAATTACTTAGCACAAAGATGGTCAAAGTGTTTGGACCTCATGGCACATTGAAGTGAT
>Bg-c20082 len=445 count=4 IPR:IPR000998:MAM blastx_SP:sp|Q0WYX8|MDGA1_CHICK/MAM domain-containing glycosylphosphatidylinositol anchor protein 1 OS=Gallus gallus GN=MDGA1 PE=1 SV=1/3e-11/444-97/800-905 // sp|P60756|MDGA2_RAT/MAM domain-containing glycosylphosphatidylinositol anchor protein 2 OS=Rattus norvegicus GN=Mdga2 PE=2 SV=1/7e-11/444-43/794-927 // sp|P60755|MDGA2_MOUSE/MAM domain-containing glycosylphosphatidylinositol anchor protein 2 OS=Mus musculus GN=Mdga2 PE=2 SV=1/7e-11/444-43/794-927 // sp|Q9GMT4|MDGA2_MACFA/MAM domain-containing glycosylphosphatidylinositol anchor protein 2 (Fragment) OS=Macaca fascicularis GN=MDGA2 PE=2 SV=2/7e-11/444-97/296-409 // sp|Q7Z553|MDGA2_HUMAN/MAM domain-containing glycosylphosphatidylinositol anchor protein 2 OS=Homo sapiens GN=MDGA2 PE=2 SV=2/7e-11/444-97/801-914 // 
CTCAGGGTGAGCGGTCTTGTTGGACGGTGGTTTGGGCTTAGTTACTGCTGTTTCCACATGATCTGTTATAGGAGCTGCAAAATTTGAACAGAAGTCTATACGTATATCATCAATAGCGGTGTCCTGAAACCAACTTGTTGACATTGTGAATTGAAAGACAATCTGAAATGGTTTACTTTGTATAGGAAGGACTCTATACGCCCTAATCCACTGGTCCCCCTGATTGCCGTACTTATAAAATATTCGGTTCCTGACGCCCAGAGAAGAAGTTTTTACATATTGTGGTCTTAGAAACACTTCAAGGCTTCCGAAACCAGTAGTAACATTCTCTCCATACATATGGTAGTAAAACTCAAAACATTTGTCCGTCTTTAACGACTTGGGGTAGAAAGGAGAGAGAAATCTGGCCTTTTTCCATTGTCTCTTTGGGTCACTCGACTCTACG
>Bg-c31007 len=441 count=2 IPR:IPR001289:CCAAT-binding transcription factor, subunit B blastx_SP:sp|P18576|NFYA_RAT/Nuclear transcription factor Y subunit alpha OS=Rattus norvegicus GN=Nfya PE=1 SV=1/1e-07/89-241/270-320 // sp|P18576|NFYA_RAT/Nuclear transcription factor Y subunit alpha OS=Rattus norvegicus GN=Nfya PE=1 SV=1/3e-07/51-152/257-290 // sp|P23708|NFYA_MOUSE/Nuclear transcription factor Y subunit alpha OS=Mus musculus GN=Nfya PE=1 SV=2/1e-07/89-241/275-325 // sp|P23708|NFYA_MOUSE/Nuclear transcription factor Y subunit alpha OS=Mus musculus GN=Nfya PE=1 SV=2/3e-07/51-152/262-295 // sp|P23511|NFYA_HUMAN/Nuclear transcription factor Y subunit alpha OS=Homo sapiens GN=NFYA PE=1 SV=2/1e-07/89-241/276-326 // 
GTCAGCCTAGTTTTCTCACCAACCCCTATTTTATTTTTTGACCTTGACCACAAACTTTGTATAAAAATGCAAAACAATACCACAGAATATTAAAAAGGAGACAGGCCAGAGCCAAATTAGAGTCAGAAGGAAAATTCCTAAAGAAAGACAGAAATATCTTTACGAATCACGGCATCAACATGCGTTGAAAAGAACAAGAGGTAGTGGGGGCATATTTATCAAAGGCGCTGATGGGGACAACAAAAGTAATATTCGTTTATAGTTTCACAGACTTGCATGGACCATATCTTCTTTTATAGTAAAATGTTCATCTGTTTGTCAAGACTGTCATTATCATTCATGGATGATAAACTGATTGAGATGAAGGAATGCATTTGATCAACAATGTTTTTTTTCTTTGTGTATTATTGGAGTAGTATTGTACAATTTAAAAACTTAAAC
>Bg-c10235 len=524 count=10 IPR:/ blastx_SP:/
CTCTACAAAGCTAGGCTACAGTAACTGGCTCTGCCTGGTAACTCGATCCACTATTATCTTGACAGTTATTGGCTGTTTAAATGTTAATTTACGACACGATATGGTTTCACTTGGTCCTACTGTATGACATATTACCCTGAGCTTACACATTGATTGTAGACATGTATCACGCCGTTGGCTACATAACTATTTTAGTTTAGAGTGTTGTGTGAAACACAGGGATGTTTAGCTAGATTTAATTACCTGACTATTGTGTTTTTTTTTAAATTGTTTTTATGATCAGCAAGCACTTTATTAATCACTGGTCAACGCTTTTCAACTAAAGTTCGGAATATAACGCCAGTCATCATTGGGCATGATCAGAGGGTGTCTCTAAATGGGCGCCAATCAAGGAAAGGATGATACATCTACCGGAAGTCTACCGGACGCACTGGACAGTGCAGAAGTCGACAGTAACACGTTGAAACAGAAACGAAATGTTCAGGGCAAATTAGAGAATACATTTGCTCTTGATGCAGTGACTC
>Bg-c40737 len=237 count=2 IPR:/ blastx_SP:/
CTCCAGCTGGTTATAACTGTTGGAACTCAGCACGACTTTGTAATTCAAATGTTGTGTCTGTAACAAGTGCCTTATATTTGATTGTTTTTTGACAGTTTTGATATCACTTTGATTGTTTTTGTTGAGTTGTTTAAGGCGGGATGAAGAAATGAAAGCATGTTATTATAAGTGTGTTAATGTTAATTACATGAGATATTTTTGTTTTATCGGTAACTATGAAAAAGTAAAAATGTTAAA
>Bg-c39138 len=252 count=2 IPR:/ blastx_SP:/
ATGTCTATAAATGTTATTTTAATATGGACAAGTCTAGTACATGGACCAAGCAATGAGCACACACAAAAGAAACATAAACTGTTGCTGTAAAGATAATACTAAATGTAATGGATAAATGAGAATGTAGAAGTAAATGCAAACTTATCTAGTCATGGATGGAACTGGCTCAAAGAAAAGAGTAGACAAACTGAAGGACACTAGCCAAGCAAACAGAATGGACAACCCCAACTGTTACTTAACTCAGTTTTAGTT
>Bg-c462 len=1005 count=208 IPR:/ blastx_SP:sp|Q5ZIP4|XRN2_CHICK/5'-3' exoribonuclease 2 OS=Gallus gallus GN=XRN2 PE=2 SV=1/3e-12/5-295/750-837 // sp|Q5R4L5|XRN2_PONAB/5'-3' exoribonuclease 2 OS=Pongo abelii GN=XRN2 PE=2 SV=1/2e-09/5-157/751-801 // sp|Q9H0D6|XRN2_HUMAN/5'-3' exoribonuclease 2 OS=Homo sapiens GN=XRN2 PE=1 SV=1/2e-09/5-157/751-801 // sp|Q9U299|XRN2_CAEEL/5'-3' exoribonuclease 2 homolog OS=Caenorhabditis elegans GN=xrn-2 PE=1 SV=2/2e-09/5-247/779-859 // sp|Q9DBR1|XRN2_MOUSE/5'-3' exoribonuclease 2 OS=Mus musculus GN=Xrn2 PE=1 SV=1/8e-09/5-157/751-801 // 
ATGTATCACTGTATGCTATAAAGATTTACAATTTGATCCAGATTACATTTTCAAGGCAGTTGTGTTGCCAGGAGCAGTCATGCCACCTCCAACATTGAAACCTGATGACTGGAACAGCAAAAATCCTGGCCAGCGATACAGACCCCAGCTGGGTTTCCAGCCTCACAGTGGCTATCGTGACAACAAGGATATGTCAGCAGCCAACAGGTTGATAAGGAACACTGCTGGCATGGGAGGTCAGGGCTATGTGATGTCTTCCCAGAACATGATGCCATCAATTTCAGCTGCTATTCCATATAACTACGGGTCCAACAACAGGGGCTATGACAACAGTGGCTACAACAACAATAACAACAGTGGCTACAACAACAACAGAGGCAGAGGTGGATCAAACAACAGTTACCAGAGGGGCGGTGGCTACAGCTCTCAGTCTGGCAACAGCTATGGCAGCAGAGGAGGTAGCTACAACAATAACAACAACAACAACAGGAGTGGCTACAACAGTAACCAGCAGTCTTCACACAGAGGTGGAACCAGCCACAGAGGTTACGGAGGTCAAGACTACGGAACTCAAGGTGGCGGTTACCGAGGCGGCTCCAACAACAACCGACAGGCAGCTGGTGGCTACAACAGTTCGCGGAACTACAACGACGACGACAGCGGCTACCAGGCATACCCTCCCGGGCAGGGGAGACATCATCCAGGCTCACGGGTTTTTGATGCTAACAGAGGTAGACATCAGCAAGATCATGGACAGAGAGATAGATACAGGCCTTTTTAGGAGAGCCAGTCAGCACAGATGTCTCTCAGTATTCACCAGTGTAGCCGTGTTGATGTTGTCCAAAGTTGTAGACTCTAAATGAAACTAGAATGAAAGTTTTAGTTTAAAAAAAAACACATGAAATAAAACAAAAAGCAAAACAAAGCTATTGCAAAACTAAAGGTTTAGATCTGGAACTACAGATTTTGTCAATTTAATAAAAGATAAGCGATGTTTCTTACTAC
>Bg-c5391 len=631 count=22 IPR:/ blastx_SP:/
GGGACCTATTACGTATTTAACTAGTATTCTTTTCTTATTTGATTTTCCACATGTGGCTACTAAGAGTCTGGGACAATATTGCCTGTTTTACAAAATATGCTGTTCTACCCCTTGGTAAAAGAGAAAACTTGTTAGATATCTCTTGCATTCGTTAAAGAAATCTATTGGCACTGCATCCAAGAACAGATTAGCATCGTGTACAGCATCACCAACAAACTTAACAATGTGTATCTAGTCTTACTATCTAAGTGATCATGATCTTTGTGGAACTCATATTACTGGCTTCAGCATTGCATTTGATTTCAATATTTCTAATCATGAAACATCCAGTAATATATACATTCTGCCAGATATACCCAGAAAGATTGTGTAAATTATTTGACAATCAATGCATATCTTGGTTATAAAGGAAATCATTGGACAGAAAAGAGAATGTATTCATTATATGTAACTATTTATTGTTTTTTTTTAGTTTAATGTATATAAAAATAATAACCCATTGTATATTTCTCTTGGCTGTACCATACCAAAAGTGTCTTTCAACTGAGAGTTGTGAAAGGTTTTAGTATGAATAAGTGCAATTCATGTCATTTTAATGTTCAATTCTAGATTTGTTAAATAAATCTTTTGT
>Bg-c38390 len=258 count=2 IPR:/ blastx_SP:/
CCCCACACTGTACTTTATGGCTGCAATATTAGCAAACATTGGTCCCACACTGTGCTTTATGGCTGCAATATTAGCAAACGTTGGTCCCACACTGTACTTTATGGCTGCAATATTAGCAAACGTTAGCCCCACACTGTACTTTATGGCTGCAATATTAGCAAACGTTAGCCCCACACTGGAATTTTTAGGTAATTATATCAAATGTAGCTTTCCACCCCCCCCCACCCCTTTTTTTAGTTCNCGGTCCGTAAACTTTAA
>Bg-c14501 len=748 count=6 IPR:/ blastx_SP:sp|O75443|TECTA_HUMAN/Alpha-tectorin OS=Homo sapiens GN=TECTA PE=1 SV=2/7e-19/26-679/1277-1480 // sp|O75443|TECTA_HUMAN/Alpha-tectorin OS=Homo sapiens GN=TECTA PE=1 SV=2/2e-14/26-679/499-706 // sp|O75443|TECTA_HUMAN/Alpha-tectorin OS=Homo sapiens GN=TECTA PE=1 SV=2/3e-11/20-676/883-1092 // sp|O08523|TECTA_MOUSE/Alpha-tectorin OS=Mus musculus GN=Tecta PE=1 SV=1/8e-18/26-679/1277-1480 // sp|O08523|TECTA_MOUSE/Alpha-tectorin OS=Mus musculus GN=Tecta PE=1 SV=1/3e-15/26-679/499-706 // 
TCTTCGTGAAGAGCAATCAGACGTGTGTAATGTGGATTGCGGAGATACCAAACCACCTGTGGATAAATGTAACTTGCCAATGGCACTAATACAGAAAGAATGTGACAAGCTTATGAATCTCAACACCTCACCATTTAAGAGTTGCCTGATGGTTAAAAAAGAGATTGATGTTGAACAGCTGAGGAAGTCTTGTGAGATTGATTTATGCTATGTTGAGGATAACTTGGACGATGCTATCTGCAGATTTGCTGAAACAATGAGCTATGACTGCACAGAAAATGAAAAGATTGAAGTCAAGAATTGGAAAGTTGATGTTACAGCTTGTAAAAAGCCAACATGCCCCAACAACATGGTGTATCAAACCTGTGGTCCAGCCCAACAAGAGACTTGCATTAGCAAACCTGTTCAGAGTAACCTGACTNTGGTCAATGACACCATACCTTGCAATGAAGGATGTTTCTCTGCTGCTGGGCTGGTCATGGAAGGAGACAAGTGCATCAAGAAGGAGCAGTGTGGATGTTTTTACAACAACGGCTATATGGCTACCAATGATAAACTCATACTTTCCGACTGTTCCTATGAGATCGTATGTTATGGTAAGAACTCGACTGGGGAGTTCCCTGTCACGTGCCAAGAGAACGAAGCCTGTAGCACTAAAGATGGAGTCACAGGGTGTTACTGTGCTGAAGGTTACACAATGAAACCAGTACAGACCACATGCGAACCTGATGTGTGCAGAGATGTTGTG
>Bg-c40193 len=243 count=2 IPR:/ blastx_SP:/
TCCTCTGCTGGCAGCTTAAATACTTCAGGCTCAAAGAAGGGCAAACCTGAGCCTCAGTTCACTTTATTGAGACCCCATGCAGAGATTCACAACAGTCAGATCTGCCTGAGGATGGCACCTCGAACACCACCCAACAAAATTAAGATTTCAAGAGACTTCTCTCAGATTCAGACTCTGCCGTCCAAAGAGCGGCTGCGCATTGTGCGCAACAGGTTCCGCGAGACCAATTAAAACGAGGGGCTT
>Bg-c19299 len=682 count=4 IPR:/ blastx_SP:/
GAGAAGACTTGGAGAGATGAACAAGACAGACGGACCAAGAGATTATTTGTAAGAGGAAACTCAATACACGTTAGTAAGATAGGTGGATAGAATGAAATGGATGTGAAATAGAGAGAGAGAGAGAGAGAGAGAGAGAGAGAGAGAGAGAGACAAGGTGGGTGAGGTTGTATGAAAATGAACGGTTGATAAAATGAGTGAGAAAGAACATAAGAAAATAAAAAAAAAAAAACATGACGAAATTTTGAGAGGAGAAAGAATGTGAGAAGTAGAGAGAGAAACAAAGAAAAAGATAGAATCAAATGATGAAAGAGAAGAGATGAACAGAAAGAGAGAGAGAGAGCTTGGGACCAGAAGAAAAAAAAAGATAAAGAAATGTTTGTATATTTGTGAGTAAATAGGGAACAAAGAAAATAAAAACGGAGACCCAAAATAAAAAAAATTAATGAAAAAGAAAGTTATTGAGAGAGAAAGGGAAGAAATAGAAAGAGAGTGAGAAAGAGAAGAAATAAAAAGAGAGTGAGAAAGAGAAGAAATAAAAAGAGAGTGAGAAAGAGAAGAAATAAAAAGAGAGTGAGAAAGAGAAGAAATAAAAAGAGAGTGAGAAAGAGAAGAAATGGAAAGAGAGTGAGAAAGAGAAGAAATAAAAAGAGAGTGAGAAAGAGAAGAAATGGAAAGAGAGTGA
>Bg-c29140 len=134 count=3 IPR:/ blastx_SP:/
GAGGCTGCCTCACTGCCTCTGCCATCAGCATCCTCCAGAATATCAGACGCTCTATCTCTTTCTCCTCCATAGTCCACTCATCTTCTTCATCACTGGCAGTTTGTTTTGGTTTCCTCTTCACTCCTGATCCATAG
>Bg-c14896 len=515 count=6 IPR:/ blastx_SP:/
AGATCTATATCTTTAGACTTGATTACATGGCATCACTTTAAAATAACAAAAAATCCAGGAGGGCAGGGTTTGTGATATCTCTAAAATCACTGCTTCAAATAAACACACAAAGTCATGGCTATGAACAATCACATGGAAGCTCGTCGTCGTCAAATGTTTATGGAGAGAGTTTGCGCAGCAAGAAAGCGACTGGAACTTGCTGAAAATCAAAAAGAGTCTCAAGAGGCTGGAACATCAAATGCATATTCTATACAGTCTGATTCTAAGCTTAATACTGCACAAGGTCCCTCTAAAACAGCCAGAAGTCAGGCTGATGAACATCAAAATGCGAAAACCTATTCTATTTATGCTCCGAGCAAATAAAAAAAAATTAGTGACTAACCTGTTGCCTCCCAATACTAAACCCTGCCCTCCATCTCTGGAAGGTGTTAAACGAGACCATGTGTATAAGATACTACACCCATATTCTGAATACTAGAGCACTTTGCCATACATAGCCCTTCTGTGATAGTTAA
>Bg-c4229 len=865 count=29 IPR:IPR013126:Heat shock protein 70; IPR001023:Heat shock protein Hsp70 blastx_SP:sp|P27541|HSP70_BRUMA/Heat shock 70 kDa protein OS=Brugia malayi GN=HSP70 PE=3 SV=1/9e-66/322-822/418-586 // sp|P27541|HSP70_BRUMA/Heat shock 70 kDa protein OS=Brugia malayi GN=HSP70 PE=3 SV=1/9e-66/8-331/313-420 // sp|O73885|HSP7C_CHICK/Heat shock cognate 71 kDa protein OS=Gallus gallus GN=HSPA8 PE=1 SV=1/3e-64/322-822/419-587 // sp|O73885|HSP7C_CHICK/Heat shock cognate 71 kDa protein OS=Gallus gallus GN=HSPA8 PE=1 SV=1/3e-64/8-331/314-421 // sp|Q91291|HSP70_PLEWA/Heat shock 70 kDa protein OS=Pleurodeles waltlii GN=HSP70 PE=2 SV=1/5e-64/322-822/421-589 // 
GGCCATCATGGACCCAGTCAAGGTCGTTCTTCAAGACGCTTTCCTGACCAAAGAAGAAATTCACGAGATCCTTCTCGTGGGAGGCTCCACGAGGATTCCCAAAGTGCAAGCGATGCTCACGCAATTTTTCGACGGGAAGGAGCTGAATAAGTCCATCAACCCAGACGAGGCTGTGGCTGTTGGTGCTGCTGTGCAGGCCGCTGTCATCTCAGGAGTTACCAGCAAGTGTATTAAGGACATTCATCTCGTTGACTTGTCTCCATTGTCTCTTGGGATCGAAACAGCGGATGGCGCTATGACAGTGCTTGTCAAACGTGGGACACCCTGCCCTATACTACATCAAGCGTGTTTACAACCCACTATGACTACCAGACAGCAGTGACCGTACAGATCTTTGAAGGTGAGAGGTCCATGGCCACAGACAACGAACTTCTTGGAGTGTTTGAGCTTCGAGACATCCCTCCTGCGCCACGCGGGGTGCCAAAAATAACAGTCACATTAGAGCTGGACGACAACGGCATCCTGAAAGTGGGCGCTGAAGACAAAGTTTCAGGGAAATCCAGCAACATATCCATCACAGCAAATAAAAATCGCTTGTGTAGATCTGCTGTCAAAAAGTTGATCGCTGAGTCCAAACGACACAGGTTGGACGATGAAACGATGAGGGCGCTGGCGTCGTCACGAAACGAATTAGAATCATTGGTCTACAAAACTCAGCGCGCAATGCAGTCGGCGGGGATCCTGCTAAGCCTAGAGAACAGATTGCTCGTCGATAAAGTCTGCAATGAGACTGTCCTATGGCTGGAAGCCAACAGAGATGCTGTTATGGGAGGAGTTGGAAGCTCAGCGAGACAGGCTGCAGTCG
>Bg-c31384 len=415 count=2 IPR:/ blastx_SP:/
CAAAAACAAACAACTAAAACTTTTTTCTTTAGGTCAGTGCTTTCGTTACAAATAACTATGAAGTTTAACCAACTAACCTAACCCCCATTGTTTTTGTTTAGCATGTAAATAGTGGTGGGGTGTTTGATGGAGAAGATTGAAAATAAAATTATGAAACAAAAAAACCACAGAAGTGTTATAATCACACACTCAAATTGTTTTGAACGACATGTTGACACCAGTGTAAAGATTTTCTTTGTTGTCATTGTGTTGTTTCCAGTCTTCAATAAATACTATATAGGGGGGGACTTGATCTCAAAAGGTGAAAGTGAATCCTTGTGATTGATTTATTTATTCTGGGCCTTTCAATACTTTCTGGATAAAATCTTCGTATTTAACATTTCCGTCCCGCGTCGGATGCGTTGTTACCACTGCT
>Bg-c27665 len=261 count=3 IPR:/ blastx_SP:/
AAACAAACAAAGACATCTGGACATTAAAAATAATTAACTTCATACAATGTTTAAATGCTGCTAGTAAGGTCATTGGCAAAAACAAACAAAGACATCTGGACATTAAAAATAATTAACTTCATACAATGTTTAAATGCTGCTAGTAAGGTCATTGGCAAAAAACAAACAAAGACATCTGGACATTAAAAATAATTAACTTCATACAATGTTTAAATGCTGCTAGTAAGGTCATTGGCAAAAAACAAACAAAGACATCTGGAC
>Bg-c15904 len=289 count=6 IPR:/ blastx_SP:/
ACGCATCCGACGCGGGGGAAACGAATAGCTCACTATTTTAAGCACCAATATTAATTTCTCCCAGAAGCACGGATTGCACAGGGGGGTGGCACTGCATTCTTCTGATTTTGACTGGAAGGAACAGTAGCTGTACACACGCAGCCTTCTGACTCACCGTTTGAAACCAAGCAAATACAGTAATGCAGGGGCCATTGTTTATACATTTTAGTGCAGCCAGCTCATAAATTCAAAGCAGAAAAAAATCTAGGCAGGGATGTTAGCGGAATATAACTAAAAAGTCACGATCTTA
>Bg-c17951 len=330 count=5 IPR:/ blastx_SP:/
CCAGTCTGACGTGATAGTTTCCAAAATCAGTTTCATGGGGTCAGAGTGTTAGCCAATAGCTTAACAAAATATCCAGTTAAAAAAAAAAGAAAAAAAAAAAACAAACAAACAATCAGGGTCTATCGCACGGTCGCACATCGCAACATCGAGTGGCGGAGGCTGGCAACACCGGAAGTAACAAGGGACATGAACGGTTTCACCATATGAAGTACATAAGAAGCGGGTTCGAAGCACAAGTGGGTGTTGCATAATGCAATGGCCACATCTTGCTCTAAGGAAAAGCACGTGTTTTCAATTATCAAATATGTTTCTCAGCATCGCCTTGTTCTT
>Bg-c37207 len=268 count=2 IPR:/ blastx_SP:/
CTTGATTATTACTTATAGTAGCATGGGGTGAATTTTCAATGAAGCTTTTGCATTCATCCACTCTGACCTGAGTAGTGTGTAATTCATTTTTAGCTGTTTTCGGTGAAGGGCTATTTAATGTTGGCGACTCGCTACTATCACATAGTTTAATGGAAACATCAAGTTCATTGTTTAAAGTTTTCAGCTGAAACTTGCCGCTATTTTTTCTTCTTTTTCTTTAATTTCTTTGTGACCTTTGTATCGACCACTTCACAGTGCGCCGCTTGAT
>Bg-c29762 len=675 count=2 IPR:/ blastx_SP:/
GATTCGTCTTCACAAATCGGGTAAGCCCGTTTACACAGTTTACTTTTATTTTCTCTTTAAGAAGCTAAAAAATGTGAAGGGAACATTCCCAAGGCTAGACATAGATAAAATCCAATCCACTAGATCAGTAATACCTAAACATAGACACGCAGGCCGCGGGTCATATATGGCTAGTATAACTTAAAAACCAAGTAAAGGTTACACACCAATGTAGCACTAATGTAGCACGGCAACACAAAACACTTATACAGAACATTAATAAATGAAGCAGACAAAGTAAACAAACGTATGAACAGGGTGCCCAGACATAAACAATACTCTACGCGCTTTAACCGAGCTCCAATCAAAGGGAAATAACCAAAATAAAATAAAACCAATTTACTATGGTTCTAGTTCAGACCCCCTTGGCACACAAAGCAATGAGACCTTACTGGCTTGAATGTAAAAGATATCCATTGAAACCTCCAGAATAAATATTCATTACTTAATTTGTCTTTTTCAAGTTAAAAAGTTATATGACTTATAAACTAGAGTTTCTTTGGACTTTTTAGGACCTCTTCTTTGTTTCGAAACAAAAATTAATATAAATCTGATACACTATGTAATTAATTGGCACCTAAATCTGATTCTGAAACTTTTTTCCTTAGCGGAACACTTCACACATTGTTAGTGATA
>Bg-c31886 len=388 count=2 IPR:/ blastx_SP:/
AACATGGCTGTATCGTTGTGGAGGACAACTACTTGCATTTTGAAACGATCAGTTTCGTTGGCATCTGTTAGATATGGCCATGGTCCACCATCAAAACAATTTGACATTCAATAAAGGGGAAAAGTACTTAATACTAGAATAGAAGTTGGTTTACCTGACAGTTTAGGACACTCTGTTGGTATTGAACGCTATGTAACTTCTGGCAAATCAAGCTGGTGATGATGACCCTTTTGAGATAGAAAGTTGTTAAAAGAGCCAAAGGTTACATTTGAAGAGCCTACCATTATTAAATCAGTCAATAATAAAAGGNTGATTGGCTGCATATGTAGAAAGAGGATGCCTTGACCATTAACTGGATGTAATGTACAACGAAAGGAGAGCCTAAACG
>Bg-c13420 len=505 count=7 IPR:/ blastx_SP:/
ATCACTCTTGGTGTTTCCGTTGGATTGGGAGGTTCTTCCCTTGCTAGAAGAGAAACCATCACCCCCTTTAGCGTTGCCGTTGGACTGATAGTTATTTCCCTTGCCGGCTCTGTTATTCCCGTTGCCTGAATTTCCGTTGGCTTTAAAGTTATTGCCCTTGCTAGAGGAGAAGTCGCTGTTGTCTTCGTCGCTACTCTGCTGGTCGTCGTCACCGAAGTCGCCAAAGTCTTGGCCTACGCGGCCTTGACCTTTACCTCCGGATTTCTGACCGTTGTCGTTACCAGTCCCGCCACCCTTAGAGAAAGAAGTCCCTTTGCCATAACCACCGTCACCTTTGAAACCGCCGCCACTTTTGGATTTTGAATCACCGTAGGAAAGTGCGTCGCGCTTGGACCTCTGTATAAGCTGACCGCTGGTAATGACCAGACAGGTCAGTAAAAGAATAAACACTTTCATGGCTTCTTGGCCACGAGGAAAGCCCTGACACTTTCACGGCTTGACCTCT
>Bg-c30571 len=497 count=2 IPR:/ blastx_SP:/
GCAGATTCTCCTTTAGGGGGTTGCTTCTACATTTAAAATTAGAACAGACACTTTAAAAAAAGATAATGAGTCATCTGCTGAAGAACTTTTAAGATCTATGATGTGACACAGACTTAGCTATGTAAACTGCTAACTATTGTTTTTTTTTTTACCTAGGTTGACAAATTTATCTTAATAAAAGCTGGATACTTTTATTCCATTTTTTTTTTAAAATAAGAATTTAGGTATCAGTAGATTCTTATGTTAACGAATAATTGCGTGTGTATTATAAATACTACAATTTCATACTGCGCTGATTAGTCTTTTACATACTTGTGACACGCTATTACAGTGAAATAAAATGGAATAGATGCAAATGAAATGTATTAACGGCTAGAAATCAGTTGTGATTTTCTATCTCAGAATAACATCAATACATAAAATTGAAAAAGTATGTGTGGGTTTTTTTTGTTTTTTTTATCGTTGGTAGTTTTTCGTATTGTTTAAAAAACNTTTTG
>Bg-c41173 len=231 count=2 IPR:IPR000203:GPS domain; IPR000832:GPCR, family 2, secretin-like blastx_SP:sp|Q9Z173|LPHN3_RAT/Latrophilin-3 OS=Rattus norvegicus GN=Lphn3 PE=2 SV=1/9e-12/225-4/909-983 // sp|Q80TS3|LPHN3_MOUSE/Latrophilin-3 OS=Mus musculus GN=Lphn3 PE=1 SV=3/9e-12/225-4/910-984 // sp|Q9HAR2|LPHN3_HUMAN/Latrophilin-3 OS=Homo sapiens GN=LPHN3 PE=1 SV=2/9e-12/225-4/829-903 // sp|O97827|LPHN3_BOVIN/Latrophilin-3 OS=Bos taurus GN=LPHN3 PE=2 SV=1/9e-12/225-4/910-984 // sp|O88917|LPHN1_RAT/Latrophilin-1 OS=Rattus norvegicus GN=Lphn1 PE=1 SV=1/4e-11/225-4/825-898 // 
GACAAGGTTCTTGTGGATAGAGTTTCTCTCCCCTTGTAGTGAGCTGAAACACTGGAAAGTGACCCAACTAGCCAGTAACGCTGTGCAAGAAATCACACATCCGATGATGGTCACTAGATTCAACAAAGATTCATGGACTTCATTGACCTTCAGGCCGTGCACGTCCAGCAGGATGGCGAAGCTGGTCATGTGATCACACTGACACGTGGTGTGACTCATGTTGCTTAGTGA
>Bg-c12044 len=570 count=8 IPR:/ blastx_SP:/
ATTCACAGCTCTGAATTAGGTTATCAAAAAAATAATAAGCTTGATACCAAAGAGACTTCTTGATTCTCCACTAGGTGTTCAATATTGGCTGAAATACAAGTCACACCACTCTCACTCAGATAAATAAAAGTAAGGACTGCTTATCAAGCAATTTGAAGCTTACATTCTTACATTTTTGTCATGTAATTTTAGCATGTGAAGATCTTTTGTGTTACTGTAGTAATAGCACAAACATAATATGACTGGATTTCTCAGACAAAGTGAACATGTGAAAGCAAGAAGCAAAACAAACAACATGACATTTCTTAATTTTGTTACGAAGTGTTTTTTATTCACTTGTGGTTAGGAAAACAGTAATCAATGTGCTTCTATTTTGTGCCCTTTTTAATGTGCTAATGTTTTCATGATGTGTGGTGCTCGTGGTAAGTGCTGACATTCAGAGTGCTATCATTAATCTATAATAGATGTGTAAGGCATCCTCTTTTTTTTTTATATTCAAGGAATTTCAGTGAATTGATTTCATAGTGGAAGAATTATTTTTTCACAAAAGGGCAAAATGAATTTTAATCT
>Bg-c13598 len=435 count=7 IPR:/ blastx_SP:/
ATTTATTTTGGTTTTCTACGATAAACTGTAGAGCTTTTCACATGGTGGATAATATACAATAAACAACGTTGGCAAAGAAGCTTTGCCATGAGTTCTTTAGCTTTTGTTACTCACCCAAGTGTTCGTATAAAAGGAACAGAAAATAGTTACACTCCTTCCCAGTGCCTTGATGATAGGTGTCCAGTTAAAGCACCTCACTTGCATTGTCCTTTCTGTGTCAAGACTGATGCCTACCAAGATCCTGTTATCCTTAAGGCTCACTACAGAGTGAAACATGTTGACAAAGGCATTGAATTTGCTGGTTTGAAGATTCTGAGGTGTTGTGATCAGTGTGATATCATAGGTATCATTAAAGGAGAAAAACGCTTCAAAGGAGCTCACTGGCATTGTTATCGTTGTCGCAACGGTTTCAATAGAAGGGATGAGGCCATAAAG
>Bg-c18395 len=280 count=5 IPR:/ blastx_SP:/
GAAGCTCCAACAACGCTTCCCAAGACGGAGGCGCTGAATGCGCCAAGTCTGGTGTTGGACTGACTCTTCTGGACCTTGACTGCCTAAAAGCGGAAATTCTAGCAGGGATAAAAAATGAGCTGAAAGAAACATTGGCGCAGCTGCACTCTCCTGCGGGGTGCAAGCCCATTGCGAAGACGGCAGTGGCGCAAGGTCAGCCTCCTCCTTCGATTTCGCCAAGCCAGTCGGGAACAGCTCGCCCGTACGGGCAGACCTATACCACACACATCTCTACACCCAA
>Bg-c25784 len=322 count=3 IPR:/ blastx_SP:/
TCTTTTGAGAATGAATAGTTAGGTCTACTGTTCCAGCCTTCTCGCAGATTGATGGTCAAACTTTCTGTATAGGAGAAAATGCTCAATACCAGGCAGAAGCGGACCTTAGTTAAATTTTAACCATTATAAAAGGAAGTGGGTAGCAGCCCGGACAAGTGGCTAACACTTGGAGCACATTCTGACTCGTTAAAAAAAGTTTGAGAGGACGAAATTAAGCAATTTAGAATTTTTTTAATGCGGTGTCTTTAAGTATTTGTTTTAATGTAGTTTTTATTTTCAGTTGTATCCCTTGGTTCGTCGACAGAACAATGCAGTTGTACGT
>Bg-c42501 len=164 count=2 IPR:/ blastx_SP:/
CTACAAACACTAACAGACTAACCAAAACAACTATCTAACTAAATCACTACAATTACTACCCTAGACCTAAGCTTAAAATTAAATAAAATATAACAAAAAAAAGGTAACAAGGAAGCGATGGCAGACTAATGGCAGTAGACTAACAACAGCCTACAGGCAGTAGA
>Bg-c7270 len=966 count=15 IPR:IPR020864:Membrane attack complex component/perforin (MACPF) domain; IPR020865:Membrane attack complex component/perforin (MACPF) domain, metazoa blastx_SP:sp|Q2KJC3|MPEG1_BOVIN/Macrophage-expressed gene 1 protein OS=Bos taurus GN=MPEG1 PE=2 SV=2/2e-60/1-966/95-412 // sp|Q2M385|MPEG1_HUMAN/Macrophage-expressed gene 1 protein OS=Homo sapiens GN=MPEG1 PE=2 SV=1/2e-57/1-966/95-412 // sp|Q5RBP9|MPEG1_PONAB/Macrophage-expressed gene 1 protein OS=Pongo abelii GN=MPEG1 PE=2 SV=1/2e-57/1-966/95-412 // sp|A1L314|MPEG1_MOUSE/Macrophage-expressed gene 1 protein OS=Mus musculus GN=Mpeg1 PE=2 SV=1/1e-53/1-966/95-409 // sp|Q9WV57|MPEG1_RAT/Macrophage-expressed gene 1 protein OS=Rattus norvegicus GN=Mpeg1 PE=2 SV=2/2e-52/1-966/95-409 // 
GAACGTTTTGCCGAGTTTATCGACAACTGGAACAGCGCTACTTCCCTTACCTCCAAAAGTGTGAACGTAGCTGCGGGAATGTCTCTAGGTATATTTTCTATCAGTGGACAGTACTCAAGTGAACACGAAGAGTTAAAGTCTAAGCAAATAGAAGACAATGCTGCGACTATCAGAGTTCAACTGAGATATCCAAGATATGAAGCAAAGCTTCAACCAGATGCAGAGTTAAGTCCTCAATTTAAAAGTCGACTGCTAAGCATTGCTGTTATGATAGAACTTAACCAGACACGTCAAGCTGAGTATGAGGCGCAGCTGCTAGTGCGAGATTTCGGTACCCATGTACTGTCCAGCGTTACGGCCGGCGCCGCACTGGTGAAAGACGATTACGTTAAGACGGATAAAATGTCCAGCTTCGCAGATTCGAAAACGGCATACTTGGCTGCTGCCAGCGCATCTTTCTTGACTCTATTCCAAATAAGTTCATCTTATAGCTCTTCATACACCGATCAGCAAAAAGAATCCTACAGCAAGGCAACGACTCACTCAGTTGTAAAAAGTTACGGTGGTCCTTTGTTCGATCTTGAATCAATGAATCTGAGTGCCTGGACTCAAGGCGTAGACAAAAATCTAGTTCCCATGGACAGAACTGGTGATCCATTAAACTATCTGGTCAAGCCACAATTACTTCCAGACCTACCTTACTCAACAGTCGATGCACTGGAGAAAGTCATACGTAGATCAATAGAAATGTACTACGAGATGAATACCTACAGAGGTTGTACTAAAATAGGGGATCCAAATTTTAGTTATGTAGCCAATGTTGATGATGGATCTTGTGGTGCTAAAGCTACAGACTTACCATTCGGTGGTGTTTATCAGACGTGTTCGGTGTATGGGCCAACTATACGTAAAAACCCTTGTGATGATCTCCAGCACGTGAATCCAAAGACCGGAACTTATTCATGT
>Bg-c27169 len=272 count=3 IPR:/ blastx_SP:/
AAACTCCAACCTGTGAAGTTCTGTAATTCCAAAACAGAACTGTCCTGTGGAGATCAGGGGAATGCAGTGGAGATTCCTGAGTTAAATAGTCTGATAGCAGACTTGAAGCCTTAACCCTTAATTTATTCAAAGTGTTTTCTGTTCAATTCTGCCACTTGTGGTCCCCACATCCAGCTGTTTGGGGATGTATTTATGATCTAAACAATGCACAGTCTGATAGTAGAAACATTGTCATAGCGCTATTACATGCATGGCTGCATAACTATTTATTT
>Bg-c28118 len=251 count=3 IPR:/ blastx_SP:/
TTCGATGAGAAGCAGATCAGGGCGATTAAAATCTACCGTTTTGTCAGTCAGTAGACTCGAGAACCTTTTGTAGTGAGTATTTGTAATAAGGTGATGTATTCTTCCTGATCAAATTGTGTATCAATGCCAAGTGCTGGTGTATTTTACTTTGTAACTTGGTCATGGCGACCTAGTTAGGCAGATTCTGATAGGGTTGAACATCCTGCCATTATGTGTTCAATTATAATAATTATTATTATTATATATAAAAG
>Bg-c26398 len=294 count=3 IPR:/ blastx_SP:/
ACAACGCATCCGACGCGGGGACAAATTGAAATCTGAAGTCTCCTTAAAATGGTAGAACCCCAGGAATATTGTTCAATGTTCTTGTTGACACAAGTATTTATCAGCTTGTTTCATCATTGAAGTTATTTATGGCAACTGATCAATTTTCATGTTGGTCTAATCCACAAACAATATTTAGATTAAATATTGNTTTCTTAGAAAAAACAATTTTCTTATATGCAGTTTTATTTTTTCTTGTTTGGAAAAAATAACTGGATTCTTGAATTATTCTTTCCTTCCTATTGACTTCTTCTT
>Bg-c34214 len=307 count=2 IPR:/ blastx_SP:/
ATTTAATGTTTATTTTTTTTGCAAGAAATCGACAAATCAAAAAATTGGTCTTCAAAAAATCTTAAGCATTTTGTATTCATTTGAGACATTTGTGTATCTACTTTTTTTGTGTTTACAGGTCTAAGACTGCAAAAGGAAAATAAGCAAAATTCTTAAGAGAATAATTTATGGTTTTAACATTAATTATTTTTTAAACCATTTTTCTACAATCAAAGTCCGAGTAAATTATCTGTAATAAAGAATAAAGGCTTTCAGTGTACTTGGAATGGGAAGAGAGTTAACTTTATCTTTCAAATAAGCTGAGAAA
>Bg-c1818 len=1113 count=74 IPR:/ blastx_SP:/
TTAAACCTTTCTTATAAACCTCATACACACAAAAAAATGTTACACTCTTGAATACTCAGTAAAAGTCTCCTTTAAAATAACTCAGGTATATATGTGATTAGAAAATATTAGAATTCAATATATTGGGACATATGAGAGTAGATCAGACTGAGAGCTAATATTTACCTTGTGGTTATTCTCTTGAGTCTAACTGACCTACACGTAGTCATGTGAAACACTGCACCCATCCTTTATGTCCGGAATCGAAGACATTGACCATATTACCATCACAAAATCTTCTAAGCTAAAAACATTTTTAGAATAAGGACAACAGTTTGCTACTTTTGTGTTTTTAAATTATTCTACACATGAGTGGAACGAGGCCATTTTGGATCAGCATTTTTTCCTGTCAATATGGCATGAGGACGATGAATCGGAACTGCTGATACACTTCCGTTTTTTCTTATTAGACAAGCAGCAAGGCATTTTGGGACAGCTAGGTAAAGGAATACATCCGCAAGAATGTGTGTAGAAGTATATGGCATGCAGGAGCATACCCACACTGATCAAGAGAAGAATAATACTGGAGTAGAGCCTGAGCATGGTCTTATTGTGACAGTGAAAGTAGCGCTCTAACCAAGTTGGTAAAGCGTTATAGATTGGTAGCCAAATATAGTTCATGAGCAGGCCGGGTATCACCAGGAACAGCAGGAGCAGCATCAAGTTTCTCAAGAACGTGCACGTGTAGATCTTAAACCACGCCCATCGCTTCATGATACGCTCCAGCCAGATAATGATGAAGATAGTGTTCAGCCAGGATAGGATTGGACTCTGCACAATGAGCCTTAACAGAAAAGGATTCAGCCATAGAAGAAGTATTAATCCTAACTCGTGCGTGCGCACACCAAAGTGGTAGATGTTGGCCAGAAGAGACAGGGGAACCAGAATGTTCCAGAAAATGTAGAAGTCTCCGGGAACATTGGGCAGCACTGGGCCTGTCTCAGGGTGCGTCGGTTCCATATTTCTTCTTTCATGTAAAATGAACCGACCACGTGATCATTGTGCCTGGCCCTAATTTGTTGGCAGGCACTCAGCATTGCATAGCCACAACAAGGAAAGACTACAACAATGGTT
>Bg-c23495 len=561 count=3 IPR:/ blastx_SP:/
GGGTAATAAAACTGAACTCCATTTTGTAGTATTGTAGCTCTAACTTGAGGGACACAGCCATGACCAATCAATTTCTTTCAGCCTAAAAATCGTGTGTAATCCATAACAAGAGTCCAGGGGAGGCAAATACGTATTTTGTTTGGTTGTATCGTGTGGTGAATGTGTAACAAATTGAGCAAAGAAACTTTCAGTTTTAAAGGTCTATAAGTAGTTTCCTCTAGATCCAGGTTGTTTTTTTTTTTGTGTGCGCTTGACATTAGAATTTATTAAAATAAAGGTCAAAGAATCTAGGTTGCAGAGTTCAAGGTTCAGGGCATCAAGTTAATAATGTGTAAGTTTACCTCTGAGAGTGTGTCTAAATATTTCATGATTACACATGAAATAATGATGTAACAAGAGTAACTATTTCACTATTTAAAATTGTACAAGAGATATTAGTGGTAGTTTCTGCTCGAATGCATACGTTTAACCTCAAAGTACTAGACCTACGTTAATGTGTGTGTGTTAGCCAAAAAAAGGGCAGGAAAGAGGAAATAAAATGAACATATTTTAGAACTGAGA
>Bg-c36878 len=271 count=2 IPR:/ blastx_SP:/
CTTTTTGCCTTTTTTGCCTTTCTTTCCTTTTTTGCCCTTTTTGCCTTTCTTTCCTTTACCACCTCCACCTTCTTCCTTTTCAGCCGTTTCTTTTTGTAGCTCTATTGGATCGGTCTGGCGGAAGTCACGTGACCATCTTGTTCTACCCCACGATAGAAGGGGTTGACGTCACGTTCGGGCTCACGTCGCATTTCCCGTACACAGACGGGAAAGTGCCTACTATGTTCGCCTTGCAGTACAGTCCTGCTCCGCAAGGGCATGACGACTCCGT
>Bg-c3834 len=424 count=33 IPR:/ blastx_SP:/
GATGGCCGCCAAAGTATTGGTCATAATCATGATGTCCAGCTCCGATGATTTCTCCACACTTAACAGTTAATATCCACCTGGACGTAGACCAAACTGATCCCCACTGGTCAGTCAGTGGACAGATCAAAGAAACAGCTTGATGTAGTATGATAGTCAACTGACACGGTCCACTAGAGTTAGTCCAAGAAAAACAAAACTAGTATTTTTATTCTGGTTCACATCAAGTTGTAGCAATGTTTTCTTTTAGATTCTAGTGTTGATATCAAGTGTCCTAATAAGAGTCACATCCACTATACCCATGGGCTAAGAATTGGAAAGTGTCACCTAAGTACAACAAATACAATTTCCATTACGTTTTAGACAAAACATTTAGTAGTAGACACACAAACTTTCAATTCCACGACGTCTTACTTTAAAACATAGC
>Bg-c32782 len=349 count=2 IPR:/ blastx_SP:/
GTCTCCTAGCGTAATGACCTACTTATTCTAACAGCTGTATGCTCCTTGACGGACAGGTTTGTACATGACATCGGATTTGGAGCGGTCAACTCAGGTAGTATCCTGTTTTTACTTACCACACGACTGGGTATGGAGTTCTTAGAAATCTAAATATAGGTAGCTTTTAAATGGCTCGTATTGCATTGTGACAAGCTATTATGTTTCCGATTAGATAAGTTAAAGATAAAAAAAAATATTTTTAACAAGATCAAAATAAAAATACCGTATATTTTAAAAAAATTTATACATCCTTTATACAACTTAAAGAGCGACTTTTTTCCCTTGACAACTAATGAATGTTCGATATAAA
>Bg-c16794 len=554 count=5 IPR:/ blastx_SP:/
CAAAGTTTTTAAGAAAAAAAATGTATAGCCTATTAATCCATTGTGCATATTTCAGTTCACCTTTTTAACATGTATTGTCTATTGTTAGAGGTGTAAGTGTTAATGTTAATGCTCAATCCTGAAGCCCTCCATAATGATGAACAAGAGCTCATTTGTTGAGGTGAGTGTTCTGCCTGGGTTTGTTACTTTGAGATAATATAATAAATATGCCAGTAATACATGATGTTTTTCTCAAAGTACCTTGTTTATTCTCTAAAGAGGCTTGAAACCATACCTGTGGGTTTTTCCTTTACACAGACGTGTTGACATTCATATTATTTAATATGGCTCAAGAAGCTATATTACTTAGCGTATACATAATCAGAACTGTTTATCCAGAGATTTACTGAAGTTGTATTTTTAAACTTACACACACATAATTTGTATTATATACACTATATGTATAGATTTAAATATGGCATAGTGTCTGTTTTGCGGTATCATTCATAGTGTAAATTACACAGTTTTATTATTTTTGTAATTTCTTGTTAGTGTATTTGAATAAGAAAAGAACT
>Bg-c15208 len=428 count=6 IPR:IPR011042:Six-bladed beta-propeller, TolB-like; IPR013017:NHL repeat, subgroup blastx_SP:sp|Q9U489|LIN41_CAEEL/Protein lin-41 OS=Caenorhabditis elegans GN=lin-41 PE=2 SV=1/4e-10/193-426/872-948 // sp|Q9U489|LIN41_CAEEL/Protein lin-41 OS=Caenorhabditis elegans GN=lin-41 PE=2 SV=1/2e-07/214-426/832-901 // sp|Q9U489|LIN41_CAEEL/Protein lin-41 OS=Caenorhabditis elegans GN=lin-41 PE=2 SV=1/2e-05/193-372/919-977 // sp|Q03601|NHL1_CAEEL/RING finger protein nhl-1 OS=Caenorhabditis elegans GN=nhl-1 PE=1 SV=2/7e-10/175-420/686-766 // sp|Q03601|NHL1_CAEEL/RING finger protein nhl-1 OS=Caenorhabditis elegans GN=nhl-1 PE=1 SV=2/9e-10/166-423/867-955 // 
GAAGGAAGAGACGACCACGTCGGAGGTTTACCACACGCGGCATCGTTTCCAGGAGCGCGTTCTTTTAACTTTCTCCCTCCTGTGCTAAGAATTTCCGCTGCAGTGCGGGAAGATGACGAGTCCCCCCGTGAACCGACCCCGGAGTTCAACAGCAACAGCCCAAGTCTCTACGTCAGCACCCCTAGAAACAAATACAACCAAAAGGGCACCGCGCTGGTGCGTTTCGGGCAGCGGGGAAGTGACATAGCACAATTTACCTGGCCCAGGGGTGTGTCCGTCTCCCCGATGGACGACAACATCTACGTAGCCGACAGCAGCAACCACAGGGTCCAGGTGTTCGACAGCACCGGGAAATTTCTGAAGACCTTTGGTCAGCATGGGCAAGGTGAGGGTGACTTTGATTGTCTGGCGGGCGTGGCTATCAACGG
>Bg-c16543 len=685 count=5 IPR:IPR005710:Ribosomal protein S4/S9, eukaryotic/archaeal; IPR001912:Ribosomal protein S4; IPR002942:RNA-binding S4 blastx_SP:sp|P55935|RS9_DROME/40S ribosomal protein S9 OS=Drosophila melanogaster GN=RpS9 PE=1 SV=2/8e-62/25-426/4-137 // sp|P55935|RS9_DROME/40S ribosomal protein S9 OS=Drosophila melanogaster GN=RpS9 PE=1 SV=2/6e-06/610-684/137-161 // sp|P29314|RS9_RAT/40S ribosomal protein S9 OS=Rattus norvegicus GN=Rps9 PE=1 SV=4/5e-61/49-426/11-136 // sp|P29314|RS9_RAT/40S ribosomal protein S9 OS=Rattus norvegicus GN=Rps9 PE=1 SV=4/8e-06/610-684/136-160 // sp|A9L913|RS9_PAPAN/40S ribosomal protein S9 OS=Papio anubis GN=RPS9 PE=3 SV=1/5e-61/49-426/11-136 // 
CGGTTGCCTAATTTCAAAATGCCGGGAGGTGTTCCCACTCTTCACAGTAAGACTTATACTGCCCCACGTCGTCCTTTTGAGAAGGAACGTCTTGACCAAGAGTTAAAACTTATTGGTGAATATGGTCTTCGTAATAAACGTGAAGTTTGGAGAGTCAAGTATACATTAGGAAAAATCAGAAAAGCAGCCAGAGAGTTGTTGACACTTGATGAAAAAGATCAAAAGCGTCTATTCGAAGGTAATGCTCTCCTCCGCCGACTTGTTAGGATTGGTGTGCTTGATGAGGGCAAAATGAAACTTGATTACGTTTTGGGTCTTAGATTGGAAGATTTTTTGGAGCGTCGACTTCAAACTCAAGTTTTCAAATTAGGTCTTGCCAAAAGTATCCACCATGCACGTGTGCTTATTAGACAGAGACACATCAGGTATGTTTTCCTATGAAAAGCAAGCCTCACTCTTAAAATCAGTGCAAACTGGGTCTGTGGTGACTGCCTGACCGCTAGGTTTTGATAATTATAAACATTAAGACCACTGGGGCTTGAGCCTTAATGCATTCGGGCTTGAGCCTTTATGTCTATGGATAAAATACATTAATTTTAATTAATGTTCAGAGTTCGTAAACAGGTTGTCAACATCCCAAGTTATGTTGTTCGTTTGGACTCTCAGAAGCACATTGATTTTTCAT
>Bg-c4838 len=765 count=25 IPR:/ blastx_SP:sp|Q9V9Z1|RM32_DROME/39S ribosomal protein L32, mitochondrial OS=Drosophila melanogaster GN=mRpL32 PE=2 SV=1/2e-24/135-587/27-179 // sp|Q2TBI6|RM32_BOVIN/39S ribosomal protein L32, mitochondrial OS=Bos taurus GN=MRPL32 PE=2 SV=1/5e-17/126-572/39-188 // sp|Q9BYC8|RM32_HUMAN/39S ribosomal protein L32, mitochondrial OS=Homo sapiens GN=MRPL32 PE=1 SV=1/9e-17/90-572/22-188 // sp|Q9DCI9|RM32_MOUSE/39S ribosomal protein L32, mitochondrial OS=Mus musculus GN=Mrpl32 PE=2 SV=1/3e-16/102-572/29-187 // sp|Q04907|RM32_CAEEL/Probable 39S ribosomal protein L32, mitochondrial OS=Caenorhabditis elegans GN=C30C11.1 PE=2 SV=1/6e-10/105-584/4-168 // 
GGTAACAACGCATCCGGGGGGTTTATAAAATGGCTTCGATGTTGAGGACGTACATTGCGTCCTTAGTTAGCAAATTTCATGCCACTTATAACCATTATTATCAAATTGTGCAAATATTGTCAAAGAATAATCCACCACCTTCTCTAGCAGTTTTAGGAATTCCACAAAATTCACAGGTGGCCGCAACTGAAAATGATACGTCTTCAAAGTCAATATTAGATTCTATTTTTGATGGTATTTTGTTAGCTGTACCCAAACATAGAAGATCAATAGAAAAAAGACTCTTTAGAAAACACAGGTATACCAGCTTTATGGAATATGGTACACCCAAAAACACTATAATTCCATGCCTGGAATGTGGTCAGTTCAAGGAGAAAGGGCATCTGTGTAAGCATTGTTATGAAAAGGTTCGATTAGAAACCAAAGAAATGCAGGCCAAAATGGGAGAAGATTTGCAATTTAACGCTCCTAGACAGGAAGTTGAATTTGTGTATGAAGGTGAAAAAGCTGAAAACAAAAACACTTTTGTTGTAAATATGGACAAATCAAGACCTAGTTGGTTCTCCAAACATTTGCTAAATAAAACAGGATATTGATTTTACACCTTAAAAAGTTAATACAAAGTTTGTGTTTTTTTGTTTTGTTTTTGAGTTACAATTTTAATTTTTGCATCATTTGACCTTCTTGAACAGAACTATCAGTGAGTGTATCATAAATGCTCATGTTAGCCTAGGTGTTCTAATTACCTACAGGTACCTGATGACT
>Bg-c25355 len=346 count=3 IPR:/ blastx_SP:sp|Q99PE7|ABCG5_RAT/ATP-binding cassette sub-family G member 5 OS=Rattus norvegicus GN=Abcg5 PE=2 SV=3/2e-13/340-14/514-627 // sp|Q99PE8|ABCG5_MOUSE/ATP-binding cassette sub-family G member 5 OS=Mus musculus GN=Abcg5 PE=1 SV=1/3e-13/340-14/514-627 // sp|Q9H222|ABCG5_HUMAN/ATP-binding cassette sub-family G member 5 OS=Homo sapiens GN=ABCG5 PE=1 SV=1/3e-13/340-14/513-626 // 
GATAGGCGCCGGTCAGGATAAAGTTCCGGGTCATGTGTTCCAGAGCCTGTGGGTACTGAGCGTGAATAAACTCTGCACCAGTCTTGATACACTGCTGACCTCGTCCAGAAGTATCACAAGTAAACTGAAGTCCGCTGAACTCATTTCCCACCACGATCTCTGTCACATACTTGTGGATGGCCACGTAGCCTGCCCACTGCAGGATTGTTGGCATGTTCTCCATCGTCCTCAGAAGCCCAGAAGCGACCAGGCCAGAGGCTGAAAAAATCAACGCTGTTGTGTTGTTGGCCAGTTGGGCGTTCCTGAAGAAGCCACAGATGCTGATGGTCATCACCTCGCCAAACTG
>Bg-c7897 len=553 count=14 IPR:/ blastx_SP:/
TGCAAAAAAAGTTTATTTCAGATGTACTAAATCTACGATAATCAAATCCTCACCAAACAAAAATAACATATTTAAATACAAATATTTATAAAAGCATTGCAGACAATAATAGATATTATTATAGCAAGTTTCTAAGAGATAACAGTACACACAATTGTTTAGATGTTTAAAGTAGAAATGATAATAGTCTCTATGATTAAATTACAGTACAGCCATTTTTAAATTATCTGAACTATATTTCAAAAGTATTAAGCTTCTTTTCATATTTCAAGAACAATTAGATCTATATGTAATTTGAAAGACAAATAATCAAGGATTTTTAACATGTAGAAAATCACCAAAATGTTAAGCCTTTTATAAAGTAAACAAAACAATACAATTACAGAAAATATCAGATCTGAATGAAAACATGACATGTTGCTTTTGTATTTACCTTTTTTTTCTCTGTATAGATTTTTGTGTGTGTAGAAGTTACCATTGAAACAAATAATAGTTAGTGATCTTTTATTATTATTGTAGCCAATATAATATGATTTTTTTTAACTTATCTTAT
>Bg-c15694 len=325 count=6 IPR:IPR000504:RNA recognition motif, RNP-1; IPR012677:Nucleotide-binding, alpha-beta plait; IPR003954:RNA recognition, domain 1 blastx_SP:sp|Q8JZX4|SPF45_MOUSE/Splicing factor 45 OS=Mus musculus GN=Rbm17 PE=1 SV=1/1e-28/319-95/326-400 // sp|Q96I25|SPF45_HUMAN/Splicing factor 45 OS=Homo sapiens GN=RBM17 PE=1 SV=1/1e-28/319-95/322-396 // sp|P42698|DR111_ARATH/DNA-damage-repair/toleration protein DRT111, chloroplastic OS=Arabidopsis thaliana GN=DRT111 PE=2 SV=2/6e-14/310-101/303-374 // sp|Q9WV25|PUF60_RAT/Poly(U)-binding-splicing factor PUF60 OS=Rattus norvegicus GN=Puf60 PE=2 SV=2/4e-10/319-95/484-561 // sp|Q3UEB3|PUF60_MOUSE/Poly(U)-binding-splicing factor PUF60 OS=Mus musculus GN=Puf60 PE=2 SV=2/4e-10/319-95/484-561 // 
TGGAAATAATAAGGACATTTAATTACTATTTACAAAATAGAGTAGCAATCATAATCAATCAATGATCGAAATTTTAATCAACAAAACCGTCGTAGTCCATCCTACGAAACTTATCCAAGTTATAGAAACAAGCTTTGACAACTCGTCCTCCAAAAAATCTTCCATTCAGATCAACCAGTGCTTTGATTGCAGACTCCATTCTTTCAAATTCAACAAATATTCGAACACATTCTTCATCAGAAGCATCAGGAACCTCATATATAACACATTTATTCACTTTGCCATACTTGCTGCATTCTTCAGCAGTTTCTGCTGAGACACGCAA
>Bg-c38266 len=259 count=2 IPR:/ blastx_SP:/
ACAATTATAAACATGACAATAACAATTAAAACAATAAAGAATCAACATGAAAAAGAATACTTTAACCTAGAATTATCTTTATAAGGCAATGGCTAAGTTAAAAGGATATAATAACAAGCTCATTTTATTAGTGAATTTACAAAATTCATAAAGAGTAACCTCCTAGAAGGCAATGCAGAATATCAGTAATTTTACACAGAAACTCTAACATTTAGTACTGATATAAATTGAAACAAATAAATCTATACCTTTTCTTTGT
>Bg-c19716 len=518 count=4 IPR:/ blastx_SP:/
CTTTTTCTCCTTTAGTGACAGGTCTATGATAAGATTTTTGGCATCAGACTTTAAAAAAAACACAAAAAATTGCTAAAACTACCCATTAAGCTTGTATTTATGGCTGTATACTCATGTTTATAATATGCTGCTATTACTAAATAATATAAATCTGAAATTTAACAAAAGCCTGTTTTAAAAATGAAATATGCAACTATTTTTTAAAACTATTGATGTTACAGTAAAAAACAAACACTATTTTGTTCTCTACATTTCTCCTACATTCTAGCGTCGTCAACAAGATGGTACTGTCAAGGCATTTAGTTACAATGATGTTAACTTGAAATTCACGTTAGCAAAAAAAAAACACAAAAAAATTTAGTCCTGGTAATGGTAACATATTACCACCAATTAATATTTCAATTAAAGGACAGCATAAATATTTAACTTAAAAGTAATCATTAAAAAAAATATTATAACTTCTTAATACCAGTACTAGAGAACACACACTGTTGACAAATAATCAGGTTAAAAAAATA
>Bg-c6426 len=462 count=18 IPR:IPR000953:Chromo domain blastx_SP:sp|Q9DBY5|CBX6_MOUSE/Chromobox protein homolog 6 OS=Mus musculus GN=Cbx6 PE=2 SV=2/6e-11/106-216/24-60 // sp|O95503|CBX6_HUMAN/Chromobox protein homolog 6 OS=Homo sapiens GN=CBX6 PE=1 SV=1/6e-11/106-216/24-60 // sp|O55187|CBX4_MOUSE/E3 SUMO-protein ligase CBX4 OS=Mus musculus GN=Cbx4 PE=1 SV=2/4e-10/82-210/16-58 // sp|O00257|CBX4_HUMAN/E3 SUMO-protein ligase CBX4 OS=Homo sapiens GN=CBX4 PE=1 SV=3/4e-10/82-210/16-58 // sp|Q9QXV1|CBX8_MOUSE/Chromobox protein homolog 8 OS=Mus musculus GN=Cbx8 PE=1 SV=1/1e-09/106-210/24-58 // 
GAAGTGTAAACATTCGGAATGCACCGCGAATCGGCTTGATTTAGAGTCTAGATCTATTTCTATGATCAATCTAGATCTGATCTAGACTGTAAAGTCTTGTATTTAGGCAAAGTGGAATATTTTGTTAAGTGGAAAGGCTGGTCTATTAAGTATAACACATGGGAGCCTGAGGAAAACATTTTAGATCAACGTTTGATCGAGCAATTCAAAAGAGAAAGCTCTGGTGGTGGCAAAAAAGGACAGAGGTTCAAAAAGAAATTTCAGGAACTTGCAGAAAACACTCTGAATACTACAGATGGAGACGATGATGATGATGATGATGAAAATGATGATGATGCAGAAGCAAGCAGCAGTACGGATGATGAATCTAGTGAATCTAGTGTTGAAGCATCTAAACCTAAAAAGGACTCTCATCGTTCTATCAGCCCAGCACGTTGTAGAAAAAAGACCAAAGAATAAAAT
>Bg-c22920 len=856 count=3 IPR:/ blastx_SP:/
TTTTTTAATATGTAACCAATAAATACTTTATTATAGTTTACCCATTGAGGGAACAATGATTTCCCTTTAATAGAAGTCTTTAATAAAAGAAATGAATGCAAATACAACCAAACAGTGGATACGTTTTGGAATACACTGATTCCGAAAAAAAATTGTAATAGATAGATATAAAACAATAGTTTGGTTCCTTCATACAAGCACACAAACACACACAAATGGCGAAACAATTGTTAGTTTCAATACCTGCTAGGTCATATCGGCATTTTGCTACCCCTATTTTAATGAAAAAAAAAAGTAGCTAAATAATTTTTGCTAAAAGTGAAAGTACCATTCAGTTTCACTTTGGTTAGAGACACTGCACATCATATTGTACCATAACCTCAAGCCAATCACGTTTGGTAGATATTTAAAAACACTTCTAGATCTATACAGACCTGCTCCATGACATTATCAGTAAGAAATAACATGAAATCTTCCAATTAACGATACAACTATGACATTCTTATAAAACTGACCAGGATATTTGGTGTTAGACAATGATTTAAAATTAGCATTTCTCAATCTGTTATTTTAAAGAAAGAATCAACATCCTATTTTAAACCCCAAGAATTGCTAAAAAATAAAGCACACCCTATCTTAATCTTAAGCCAAAGAATTGCTAAGAAAGAATCAACATCTTATCTAAAACCCAAGAATTGCTAAGAAATATCTTATCTAAGAATTGCTAAGATGACACAAAGGGTTAATTATTTAATAGGAAAAAAAAAAATAAACTTCAGCAAAATTTTTTAAAATTAATTTTTTTTATTTTAAAAATTTAACTAAACTTAAATTAGTCACTAATTATGTGCATAGG
>Bg-c7400 len=608 count=15 IPR:/ blastx_SP:/
TGAAATAAGGAGCTGGGTATCGCTATGGCATGTTTACCATTTAGTGGGACATGTTTTCAAGCTGGTCTAGACAACTGGTACAATCAAACCAGCTGATCTCTCTTTTGTGTGTTTGACTTCCTGGTATACATGGTTGAATGATTTTCTAGCTGATAGAACTTGATGTTTTTATCTTGGTCCTTGTGAATGAAATAAGAACTGAGTTGGCGACACTGAAATAAGAAGCACTTAGTTGGCGACACATTATACATGTTGCCACTGAATAATAAAATTTAACTACAAATGAACAAATGGTGTTGATTAAACAGTTTTTATAAATGTCATTGACTCAGTATATGTCAATGTTAAAATTATTTAGAATAAAATCAATGTAGCCACAGATTGGTTGACATTATGCTGAAATTTTTGTTTACTAGTTGTATACATTGCATTGCATGTGTAAAAATATGTCTTATAGGGTATTTAAAGATTTGTGTAAATTATGTCTTATAGGGTATTTAAAGATTTATGTGTAAAATATGTCTAGTAGGCTATTTAAAGATCTCTGTGTATGTTTCTTAAAGTAATTTCTTTACAAACCTTGCAATAAAGATTGTAATAATTTTTGT
>Bg-c16517 len=704 count=5 IPR:/ blastx_SP:/
GCACGAGAATTCCTTCCCTCCCTCCACCTGCCCTAGCTACGCCTTTGATGTACCCCATTGCATCGATTCATGATTTTCATTAGGAATTTTAATTTGAAATTGACATTTGCAAGGGCAGCATGAAAATCAGAAAGAGCTACCCCCTCTTTCAGAAATTAAACCATACGTGGCCCTGTTATGGTAGATGCCCAGGGCACCGCGCACTGCTTAAGCCGGCTTTGTCACAAGTGCCATCTTCGAGGCCCAAACTGTTACTTTCTATCAGTGACCTGCCATATTATGATGGATACTTTGACCCCACAATTTTCCAGATTCTCGATTTTGACAGTGGCAGAGAATGACAAAGCGTATTTCAACTCACTCTTTCTGTCTGGTAAAAAGTTTGTACACGTCATTTTTCCCACACCCAATGTCGGATCAAGCTGAAACTTTGCACAATTATTCTCGTGCCGAATTCGGCACGAGAAATAAAGACAGGATGTGACGTTTCCTGACATGTTATTCCAGAGGATACTAGGAGTGTTTGTGCAACTTTGGAGAAGCGATTAGGGACTCTATATAAGCCAGAAAGTTAATGTGAAAGTCAGTCGTATGGAGTCGTGAGTGAGCCGTTACAGTCGATACAGACGATGTAAGACGTGTGCGGCCCTAGTGGAAGAGAAATGTGTACGACTCGATGCGAGTTAACTAGGGTAGAGTTAACG
>Bg-c17443 len=404 count=5 IPR:IPR019448:Oestrogen-responsive protein Fam102A/B, N-terminal blastx_SP:sp|Q5T8I3|F102B_HUMAN/Protein FAM102B OS=Homo sapiens GN=FAM102B PE=1 SV=2/6e-45/3-401/5-138 // sp|Q6GNM6|F102A_XENLA/Protein FAM102A OS=Xenopus laevis GN=fam102a PE=2 SV=1/2e-44/3-401/8-141 // sp|Q78T81|F102A_MOUSE/Protein FAM102A OS=Mus musculus GN=Fam102a PE=1 SV=1/3e-43/3-401/8-141 // sp|Q5T9C2|F102A_HUMAN/Protein FAM102A OS=Homo sapiens GN=FAM102A PE=2 SV=2/4e-43/3-401/8-141 // sp|Q8BQS4|F102B_MOUSE/Protein FAM102B OS=Mus musculus GN=Fam102b PE=1 SV=2/2e-33/84-401/1-107 // 
AGAAGAAATTCAAGTTCCAGGTACAGCTTGGACTAGAAGAGCTCGCGTCTGTTCCATTTGTTACTGGTGTCTTGTTTGCTAAAGTCAGGCTTCAAGAGGGCGGCAGCTTCACAGATGTATCTTCAAGGGAAGAAGTACACAACAATTGTGTCCAATGGAAAAGTAAATTTGAATTTCCCTGCAAGATGACAGCAAGCTTAACAAATGGTGTCCTTGACCCTTGTATAGTTAGAATCTCAGTTAGAAAGGAGCTCAAAGGAGGCAGGTCCCATCAAAAACTGGGCTACGTCGACCTTAACCTTGCCCACTTTGCTGGAGGAGGCAAGCTATCAAAGCGCTACCTACTAGAAGGCTATGACACCAAGCACAGGCAAGACAACTCCAACATTAAGATCAGCGTAGAG
>Bg-c26043 len=309 count=3 IPR:/ blastx_SP:/
TTTTTTTTATTGAAGTAAAACAAAATGGTCAACAACAGTAGTTTACAAAAGTTTACTTTTAATTTCAGAAAATGTTGGATCCTTTTTATATGATGGTCTAAAAATATACCATTCCCATTTTTTTATAACATTTTTCCATACCACATAAAGATATTGAGGCTGATGCATAAATAACACATATATTAATCCACAGTGGACATTAATCTTAAGCAAATATAAACATTTATGGAAGAAAAGTAAACAAACAAGTTTCAAAGCTTTCACAATTGGAGGATGGATCTGATGGACAAGATCATTTAAAAATTTACA
>Bg-c3043 len=1691 count=42 IPR:IPR013781:Glycoside hydrolase, subgroup, catalytic core blastx_SP:sp|Q8WPJ2|MANA_MYTED/Mannan endo-1,4-beta-mannosidase OS=Mytilus edulis PE=1 SV=1/2e-88/1691-705/35-363 // 
TAAATGTTTATTAAAAAACAACTTTATTTGCATCTAAAGTTTTGGAAGGTTTCACATAGAATAAGGGAGAAAAAACAACAACAGAAGGCCTGTCAAGGGAGGCAACTTGAGCTCTTAGGATTGTCACAAACTAGCTCCATTTTTTGTGAACCAAAAGATCAATGAATGAACCCCAAGATAATTACGTGAGCCTAAAAAATCAAAATTGTTAACCAAAAAATCAAAATTGTTAACCAAAAAATCAACGTGAGAACCCAAAGATCAACATGATCTCTCAAGATCCAATGACTTCTGTCTCATTACAAAATGTAAACTAGTACAGACATTGACATGATAGCAGCCCAATTGTAAGTCATAATAGAACATTTAAAATTTCTACCTATGATAAATTTTCGGTTCTACAAGACAGACTAGAGCATAGTTTTAGGAACAAATACAAATATTTTGTGGCGTTGGCCTTTTATTCCACCTAGAGACTACACCACCACAGTGTAGGCCTACACATTTAGTTTTAACAGGAAACTTGAATAAAAAATTAAAAGAAACTAAACTTAGACAAAACTGAAAATGTCTAAAAAAAAATAAAACGATGGCACCTATTATTGTAAAGGTCATCTATTTCGTGACATTGTCCTTATTGAGCAGCCATACAGGCTAGTAGCTATTAGGACAGTGGGCTAGGTAGGTCACTGAACGCTAATAGGTATGACCCCATTGGTGGTCAAGTCCTTTATGTGACGAATGCCATTCCCCTGTTCTGGGTGACCAACGAAATCCCAGCTCCATGCACCAGCGTAGCCGTGATTGTAAGTCCAGTCAAACATCTGGACAATGGTCATGCCGCCACCATCTTGTTCGTTGAATTCCCCCACAAGGATTGGTTTGGAGACAACATACTCTGTGAAATTGTTCAAAAAGGGTGATTCTTTGACAAACTTGCCTTCCCATGAGTAAGAGTGGAATTGATAGAAGTCCATGACACCCGTTGGTTTTCCACCTGCTTTGATCAGACAACTGTCCGAGTAATGGTCTACATCCCCAAACTTGTCTGTGTTAAATCTGGGATTTGAGACACCAACACTGACCAGGTATTTGGGGTCAACCTGTTTGATTGCAGCCGCCTGCCAGTTGAGAAATCTTAGAAATTGTTCATAGTTGTATTTTTTACCAGCCCAACCAGCGCCACTATATTTCAAAGCTGTGGTGTCGAAACAAGGATTCGCGTGAACGATTTCTGGGTTCAACATGCCTTCAGGTTCATTCATAATGTCCCATGATCCCAAAGCTGGATGACCTTTGACCGCGGTGGCCCATGGTATTAACGCATTGTCAATGTAGGACTGGAGTTTCACTGGGTCTCTAAGAAGTCCATCCAGTCTGTTGTGTGCGTCTTGGTTGACAGCAGCGTTCCACAGACATGGCGTGATGAGTATATCGTATTTTTGTCCAAGATTAAGTAGCTCAATAAATTCACTCAAGAACGTTCCTTTGGTGTCCAGTCCAATGACCATTCCACTGCTGTTGAAGGCTGGGGTTGTCTCCGCTTGTATGTGAATCCAAAGTCTCATGGAGTTGCCTCCAGCGTCGTGTAACATTCTCATCTGATGCTCCAGCTGGACTTTACGTTGCTGAAAGAGCCCATCCCCAAAGTCATAGGCGTAACTGATCCAGGGGAGGTTTACCCCAGAGAG
>Bg-c18471 len=274 count=5 IPR:/ blastx_SP:/
GGAAATAAAAAAGATTTTCCGTTTGGATTTGTAGGAAAGATGAATAAAGCATGGATAGAGGGTACTTCTACAGAATGGAAATTACATCACATTTAGCTGTTGCCATTAACTTGATTTTAATCCTACCATTAAGTCTGGCTCAACCTACTTTAAAGTTAGTGAGTGTGGTGAGTATACAAGTGTCACTTCTAAATAAATTATACTTGAGTGACTTGATCTGGACATTTAATGACTGAACCAAAAGGGTTGTATCGCCATGGTGACCGTAGCCCTG
>Bg-c453 len=1445 count=209 IPR:IPR013320:Concanavalin A-like lectin/glucanase, subgroup; IPR000757:Glycoside hydrolase, family 16 blastx_SP:sp|Q76DI2|BGBP_TENMO/Beta-1,3-glucan-binding protein OS=Tenebrio molitor GN=GRP PE=1 SV=1/4e-36/407-1192/169-430 // sp|Q76DI2|BGBP_TENMO/Beta-1,3-glucan-binding protein OS=Tenebrio molitor GN=GRP PE=1 SV=1/4e-36/1230-1364/438-481 // sp|O96363|BGBP_HYPCU/Beta-1,3-glucan-binding protein (Fragment) OS=Hyphantria cunea GN=gnbp1 PE=2 SV=1/3e-32/29-1189/2-425 // sp|O96363|BGBP_HYPCU/Beta-1,3-glucan-binding protein (Fragment) OS=Hyphantria cunea GN=gnbp1 PE=2 SV=1/3e-32/1188-1364/424-481 // sp|Q9NL89|BGBP_BOMMO/Beta-1,3-glucan-binding protein OS=Bombyx mori PE=1 SV=1/2e-31/404-1192/185-444 // 
CACCTTGTAACATACAAATCGAAATACCTGCTTCAGGATGAAGTTAAAGATTTTATTCTGGACACTTGTCCACTTGGGCGTTGTACTGTCTGGACAAGTCAACATTGAATACAACAAACCTGTCTTAAAGTTAACATTACCTTCCTATTCGACACCTGAAGTAGGTCGAATTATCTTCCGTTACACAGTCAAAGATGTTCAGAAACATGGTGTTGCTGTTCTTACCACTGAAGGCTGGCAATATCAGACCAGTGATGTCTCACTAGATGGCGCTGATGAAGTCTCTGTTTATGCCGTCATCTACGACGTACATGGAAATCTGAAAGAAGTCACTGATACGTCTGCATTGAAACTAGCTCAACAGGGCGCCGTTGAAATCCCCTCCCCAAGACGTATCAGGGCGGTGATATTCAGAGATGATTTTAATACATTAAACAAAGCCAGCTGGCGTTCTGAAGTCTCCATGTATGGAGGAGGTAACGGAGAGTTCCAAGTGTACACCAATGATCCAAAGAATGTGTACACTCATGATGGTCACCTGTACCTTAAGCCGATCGCTACAGTGAGTGACCCCAGATTTAATGAGAACTTTCTTCATACTGGACACATGGACATGTTGACTTTGTTTGGTGAGTGTACACATGATGGAAACAACGGCTGCACAAGAACTGGCGGGGCTGAGATTCTACCACCTGTCATGTCCGGTAAAGTCTTGAGTATTCCTTCCCTTAGGTATGGCACAGTGGAGGTCCGAGCAAGACTTCCCAAGGGGGACTGGCTGTGGCCAGCCATATGGATGGTCCCTAAAGATAGCAAGTACGGAGGCTGGCCAAGATCTGGCGAGATAGACATCATGGAGTCTAAAGGTAACCCTGGGCCTGATTTCGTTGAGAAGGTAACAAGTCACTTGATCTGGGGCACTTCCTGGGAACACCAGTACTGGGCAATCACCTATCACGGAGAACGACACGCCACTAACTGGCATTCTCAGTTCCACACATGGCGCATGGAATGGACTCATGACCACATTCTGACATTTGTAGACAACCAACTGATAATGACGGCAGCGCCACCAGCCGGTGGATTTTACGAGCAAGGGAAATTTACCGGAACTAACATTTGGGCTTCTGGGACAAAAATGGCTCCCTTTGATCAGGACTTTTACCTCATCCTGAACGTGGCTGTGGGCGGGCACCAACGGCTACTTCGCTGACGGCAATCATTACGGTAACGTCACCAAACCTTGGCACAACAGTTGGCCTCACCCCAAGCTTGACTTCTGGAACGCACATAACACCTGGCTGCCCACCTGGCATGGAGAGAACGCTGCTCTGGTCGTCGACTATGTGGAATTTAAGAGTTTATAAGACGTTCATTGTTTAAATTCTGTTTCGCAAGAAATAATTATTCTGTAAAGACCACCAAAACATAGAAACTATTGCCCA
>Bg-c31659 len=400 count=2 IPR:/ blastx_SP:/
CCCTCAAGTGGGGGCCCGGGGGCATTTCAAATTCTCCCCCTCCTGCCCTCACCTTAGCTACGCCTCTGACTACACCACACAATGGCACTGCGCAGCTTGTACAGAACAGAACCTACAGCATACTAGAGCCGGCCCTGCTTAGTTGAAGGAAATTTTAAAAAAGCATTTTGAATGTAAGTTAAATATGATGAAGTTGTCATGATCTACACGAGTGAGAGTGTGAAGTTCAAGTTTTTACGTACAATCTCATGAACAGGCCTCTTGACACAGCATTAGGGTAGAATGAGCATTTGTATGAATATGTCCTAGCATATGGAATAAGAAGTGTGCCTTTGTCTTTGAGTACTTCTGAGTATTTTATTAGGTTGCGTTTTTGTAGTTATAAGTTTTTGGTTTAGTG
>Bg-c18742 len=254 count=5 IPR:/ blastx_SP:/
TTGTTTCTGCACACCAAGCACATTTAAAATGGAGATCTACATAGGCCTATAAGCTAGATTCAGAAGAACATTAATGTCACTGTTAAATATGCAAAATCAGATCCTTCCTTTAAGTTTCAAATATACTTTTGATATTTTTGTGGAAACTGTCTTACACTTCACTAAATATAGACTACAAGTATGCATCTTGCCTATTTTGTACGCAAATGGACACAAAAATAAGCGAATACAGTTTATCATCTGAAAATATGCTA
>Bg-c22875 len=981 count=3 IPR:/ blastx_SP:/
CTAGCTCAATCAACTTCATCTGAAATAGCTGCCATGAGTGGATCTGCTCACCAATCTGATGCTGTCAGTGAAGATGTGGCAGGTGTCAAAGATGAAACCACGATTGGTTCATCAGTGGAAATCCATGAAGATGACGAGGATCCATCTCTTGTGATAGACGAATTGTCAGAGAGTGTTTCAGATGAAAAGAAGAAACTTACTAAGAAAAGAAAAGAAACTTTTAAAACAGCAGCAAGAAGAAAAGGAAAGAAATGACCATATGGATGAAGTCATTGCCTTTGTTGCCAATGCTGGCTCTGGTGATGAAGATGATACTGGGGATAAGGAAAGTCCTTCCAGTTTGCAGATAGATTCAGCTAGTAATGACAGAGAGCTGGATCTTTTAGATGTCATAAACCTGAAATATGAAACTAAATTTTCCAATGAGAAAGACCACAAGTCAATGATAGTTTCAGACATGTTTTTTCAAGATGATAAGAAAAAGTCTCCTGAAACTGCTCCATTCAGTGCTTTCCGCAAAGATAAAAAGGAGACGTCAGTCAAGAAAGATGGGGACCAAGAAGTCACTCAGAAACCTACAACTCTGGCCAAGAATGATGACATAAGTTTAGAAGAGGGTGCAACTGTAGCATCCAATGAATCACCAGAGAAAGAAGAAAGAGAGAAGCAAAAAGAACAAGAAAGATTAGAAAAAGAACGGCTTGAAAAAGAAAGAGAAGAAAAAGAAAAACAGAAAGAAAGAGAAGAGCGAGAGAAGCCGCGAGAGAAGGAAGAAAGAGAAAGAAGAGAGAGAGAACGTGAAGAAAGAGAAAGAAAGGAAAAAGAAGAGAGAGAAAGAAAAGAACGTGAAAAAGAGGAAAAGGAACGTAAAGAAAGAGAAAAGGAGGAGAGGGAAAGGGAACACGAAGACTAAGAAAAGGAAGACAAAGAACGAACCCTTAAAGGGGAAAAGGAGAAAAAAGAAAAAGAGGAGAGGGAAAG
>Bg-c25829 len=319 count=3 IPR:/ blastx_SP:/
CAAAACTGTGTGATCTCACCGACAAATACATATCATTTCTATAACATTAAAAAAAAGTAGGCCTTTAAGATAATTTCCTAATTAATAGTAATCAAATACACATTGCGCAGCGATTTGGAAGCAGATGGGCAAAATGTGTGGACAGTTGGAGGCTCACCCAGAACCGAGATGCCTAGAGGAAGCTGGTTGGTGGCCTATGCCCCAGAAGGGACCACAGGCAGAGATGAGATGACACATTTGTTTATAAAATGGAAAAAACTCTTCATAAAAGGTATTTGTACTTGTCTTTTAGCAAAACTTGATTAAATAAATAAGAAAA
>Bg-c24056 len=446 count=3 IPR:/ blastx_SP:/
ATGAAATCAATGAACTTTTAATAATTTAACAATTAAAAGAATTGTAGATCGACATGGTATTATTATGTTATTATTCCTCTATAAAGCTAATTTAGATAGTCCAAGATAGATCTGGTCTTCTTTCCTCTTCTGTTACGAGCGAAGCTCTAGATTAGATCTAGAATTCTAGATCACATTTGTGTAGCAATATAAGCAAAGCTAATAACTAATCTTAAAGAAACATGGAAATAAATCTAATTTTAATAATTTAGAGCTAGATCTAGATATTGGCCAAGTAGCAGAATGATGTATTCTGATTTTATTGATAGGAATAAATTATTAAATTTTATTTCCAATTTCCACCTTTTCCCCGTTTCCATCCATGTAAAAAGATTATAATAGGCATTGTATATAATAAAAACATATTAGAAAGTATAAAATTAGTATACAATTATACAAAAAAACTC
>Bg-c29372 len=790 count=2 IPR:/ blastx_SP:/
TCTGTAATTCTGTATTAAGGGAGGTCACTGTAAGACCCTTTAAGGTGAGTTGTTGATAGAGCATTGTGTTTGTAAATCTATAGCACAGTTGTCTTTCTTTAAGGTAATTTTTTTTAAGACTATGGATACAGTGAACTGTATTATGGCTTGTAAAGACATCGAAATCACACACCTTATGAGGGTTTAACTCCCACATTGTGAGGGCTAAACTCCCACATTGTGAGAACTGAACTCTCGCATTGTGAGGACTTAACTCCCACATTGTGAGGACTGAGCTCCCACATTGTGAGGGCTAAACTCTCGCATTGTGAGGACTGAACTCCCGCATTGTGAGGACTGAACTCTCACATTGTGAGGACTGAACTCTCGCATTGTGAGGACTTAACTCCCACATTGTGAGGACTGAACTCTCACATTGTGAGGACTGAATTCTCACATTGTGAGGACTGAACTCTCACATTGTGTGTGCTGCAAGAGTAAAATCATAATTTTAATGCCTCCGAAGGTTCCTAATATAAGGAAATGATAAGTCAAAATCCAGAAAGTCATGTTCTGTACAATGTCCTCTCCCGAGTCATACCTCATTATTTGTACTGTTCTCTTACATTGCCAAAACAACGCAAATAAAAAAATCGCCATTCTCTTATTGTGAAATTATAAACCATATTATTATATGATAAGATAATATCTCTTATTTCAAAATAGATGTATCATCTATTTTTTTTTGGTATATATTAATTTGGGGTCATTTTTGCATCATTCTTCATCCAACTTGACATTCATATTGTGG
>Bg-c18017 len=320 count=5 IPR:/ blastx_SP:/
ACAAATGATTGCTCTTCAGTTGGAGAAGGAAAAAAGGGCATGGAGGAAAGATCTGCTGTTAACAAAGGAGAGAGACAGCTACAAAAGCCTGTTAGATTCTTATGAGAGTGAGGTCACTGTCAACTTCGACAGTCAGAGAAAGAGCCAGGTCTTAAAGCTTGAAGAGGTTATTGCTGGATACAAAGAACAGAACTCATCTCTGGAATCAGAGATAAGATCATTGTCTGAGAAACTGATGACAGCACAGATCAAATATGAACAGGTGAAACAAAATTTTGATGGTGTACAACAATCTGAGCAACAAAAGTCTTCTAGGGAAA
>Bg-c7578 len=348 count=15 IPR:/ blastx_SP:/
AACCGTATAACTATTTTTATTTGTTTCCACCTATCGCCTACACCTACTTCGCTTCACTATATAATATATAGATTACTAAAACGGATAGTATGGGTTCACTTACAGTGATCCTTATTGCAAACTAAACATATCATTGTATACGTTATATCGGAACTCTTATATAATAAAACAACAGTTAAAAACTGCTTTTCTAATCCGATATTACGGTTAATGTGCTAGAGCACTACTCTCTCATGCAGAATGATTAAGAACTTTATCTGTCTACCGGTACAAAGACATAAGTCTGTTAAAAGAATACAAATTCGTTAAGTCACCACTTGTCCGAAAATAAATATTCTGCTATTAAAA
>Bg-c3988 len=977 count=31 IPR:/ blastx_SP:/
GGTAACATATAAACAATTCTATTAATATTTAGACAAGATAATGAATACAACATCAATACAATATCACAAAAGTAAACTCTGTTCCAGCAAAGAAAAAAATGGGCCCATTGCCCAATCTTTGAATAGTGCAGTAGATTGTTAAATGGAACATTGAATGAATGCTACTTGTTTAAAACAAAACTTGATACAGAATCCCTAGACAAAGCAGATGAAGAAGCTTCATATCTATTTCTAACCATTCATAGAAATATCTATAATGAAGTAAAATATTCAAACAGTCAGAAATATTGCTATTATCACATAGACAAGTAGGTAATGGTGTAGGGTGAATAGAAAATAGTTACTGGATAGTCCATGTTATCAAACTCAATATCAAAACATGAACTCTGTTCAATAGTTGGAACACTAGACCAACTCTTTTTGACAAAATGCTCATTAATACATTGTATGTATTCATATTAAAACATTTTCACTGTTAGCATTCATATTTAAGTTTAAGAACAAACAAAGAAATGATGTACTGATGCTGAAAACTTTAAAGTTCAAGGACTCAAGAATAAATAAGTTCAGAAGAATTAATGTGTGTCGATTCACTGTTTGGCAAATATTATAGTCATCTATCTACAACTGCACAGTTATAAAAGCATAAATGTAAGCTAGGACATTTATTGAACTTTGTACACCTTCTTGCTGTTCAGTCATCAAATGAAATGAGCTGTAACTCCCTCTGACCAGCTGATGCCTGTCCTGCAGTAGGTCCACCTTGCTGTTGGTAGGGCACAGCATAAGGCTGTTGTGTGGGCTGTTGAAATTGTTGTGGTTGGCCAGGAGTGACATACTGTTGTTGAATGGGCTGATTGGGGTTGTTAAACATAGGAGCATTGGGATGTGATGGTAATGCTGCTGCTAAACCTGGCATGTTAAAAGACTGAAACTCTTGAGGTGTAGGTGCCCCAGCATAACTTGCTGCACCTTCT
>Bg-c39874 len=246 count=2 IPR:IPR002229:Blood group Rhesus C/E/D polypeptide; IPR001905:Ammonium transporter blastx_SP:sp|Q9QUT0|RHAG_MOUSE/Ammonium transporter Rh type A OS=Mus musculus GN=Rhag PE=2 SV=1/1e-24/1-243/217-297 // sp|Q7TNK7|RHAG_RAT/Ammonium transporter Rh type A OS=Rattus norvegicus GN=Rhag PE=2 SV=1/5e-24/1-243/228-308 // sp|Q02094|RHAG_HUMAN/Ammonium transporter Rh type A OS=Homo sapiens GN=RHAG PE=1 SV=2/1e-23/1-243/207-287 // sp|Q6XL41|RHCG_CHICK/Ammonium transporter Rh type C OS=Gallus gallus GN=RHCG PE=2 SV=1/2e-23/1-246/226-307 // sp|Q7T3R4|RHCG2_TAKRU/Ammonium transporter Rh type C 2 OS=Takifugu rubripes GN=rhcg2 PE=1 SV=1/2e-23/1-246/226-307 // 
TCGGATATCTTCGCAATGATTGGTACCGTTTTCTTATGGATCTATTGGCCATCTTTCAATGGCGGCTTCGCAGACGATCAACAGCAAAGGGATAGAGCTTATTTGAATACGTTTTTATCTCTCTGCGCATGCACGGCGGTTACCTTTGCTATATCTAGTCTAGTGGACAAGGAAGGAAGGTTGAACATGGTCCATGTACAGAACTCTACACTGGCAGGAGGTGTTGCTGTCGGGGCCTCAGCACAG
>Bg-c20088 len=444 count=4 IPR:/ blastx_SP:/
CTGTACCTAGAAAATTATATTGGCTCAACATTGTTTTCTGCAGCTTGTTTTCTTTTTTTACTTCTTGGTTCAAATCTTAACTTTTTTTTTTAAAAAGAAAAACAAAAAGAAACATTTCAAATGTATTTTCAAATATAAATATACAGAAAAATTTCAATCGTTTAAGATTTTTTGAATGATTACAAAACACTTTTTACTCTAGATTTGCTGTCATCTTCTTATACTGGTAACTAAATAAACTGCCTTTTACAAGGCAGTTTCGCAGTTAATATGTACCATTCTTGCATACTCCATAACAAACATTTTTTTTTTCTTACACAAAACAGATATTCTTCACACACTGTACTGCGTTCTCTGTGGAGGGGGTTGGGGCACTATAGGAGGTAGAGGAGGGGTTTCTCCAAGTGGCCGGAGAATAGGTGTAACTCTGTCTAGTAAATCATT
>Bg-c911 len=1908 count=134 IPR:/ blastx_SP:sp|Q6DEU9|CTR9_XENTR/RNA polymerase-associated protein CTR9 homolog OS=Xenopus tropicalis GN=ctr9 PE=2 SV=1/3e-18/1-222/757-828 // sp|Q62018|CTR9_MOUSE/RNA polymerase-associated protein CTR9 homolog OS=Mus musculus GN=Ctr9 PE=1 SV=2/3e-18/1-222/757-828 // sp|Q6PD62|CTR9_HUMAN/RNA polymerase-associated protein CTR9 homolog OS=Homo sapiens GN=CTR9 PE=1 SV=1/5e-18/1-222/757-828 // sp|Q4QR29|CTR9_XENLA/RNA polymerase-associated protein CTR9 homolog OS=Xenopus laevis GN=ctr9 PE=2 SV=1/1e-17/1-222/757-828 // 
ATAGCTTTGGTGCAACAGAAACTGGCCACAATGATCCTACAAAAGAATAGAAAAGAAGTAACTTAGAAGAAAGTCTTGATAGCTGTGAGAGATCTGGAGCTGGCTCACAGATACTTTACCTATCTAGGTCAACATGGTGACAGAATGAAGTTTGACTTGGCTCAAGCTGCTGCTGAAGCTCAACAATGTTCTGACTTGTTGAGTCAGGCTCAGTACCATGTAGCCAGAGCTAGAAAGATTGATGAGGAGGAGAAAGAGATCAGGAAGAGACAGGAAGAAGAGATGGAGGCAATCAAACAGAAACAACTCAGTGAACAGCTTGAGAAACTGAAGCAGAAAGAAGAACTGGAGAAGAAGATGTTGGAACAGAGAGCTGTGTTTGTGGAGAAAGCCAAGAAAATTAACTTGGAGCCAGAGCCAGAGGACAGACCACGCAAGTCTGGTGGCAAGAGGTCCAAAAGAGCAGAGGATGGTGAAATCTTGTCTGAAGGAAGTGAAGATGACAGGCCTAGGAAGAAAAAGAAGAAGAGGTCTTCCAGTATGTCTGGGGAAGAGGAGGAGGAAGGAGGAGAGAAGAGGAAAAAGAAGAAGACTAAGAGGAGATCAAAGAAAGATGATTTTGTCAATGACAGCGATGATGAGAATGATGACAGAGGCGACCGTAAGAGGCGCAAGAAAGGTAGAGGAGAAAAGAAGAAAAAGCAGAAAGATGAGGATGATGGGCTGACGGCCAAACAGAGACGTAAAATTGTCTCCAAAGCTGTCATCTCCTCTAGTGAAGGCTCTGACTCAGACACCAGGAAGAAGAGATTGGAAAGCAGTGGAAGTGAACGGGAAGAGGTCGGTGTTCGAAGGCGAAGAATTCTGGGAAGTGATAGTGAAGATGGTGGAGGTAGTAGAAAATCTGGCTCTGAATCAAACAGAAGTCGTTCAAGATCTAGGTCAGGCTCAGAGAGAAAATCCAAGTCAAGATCTCGATCCAAGTCAGGTTCTCGGTCCAAGTCTAGATCTGTGTCTAGGTCAAGGTCACGTTCCAAATCAAGGTCTAGATCTAAGTCCAGATCACGTTCCAAATCTGCTGGAAGCAACAGATCTAGATCAAGAAGTGGCTCACGGTCTGGGGGAGAAGGAAATAAATCTGGGTCAGAGGCTGGCAGGTCACCTCCCAGGTCTAATGCTGGGTCAGATGCTGAGGGCAGTGACAGGGCAGCAAAGTCAGGTAGTGAACGTGGAGGTGGCAGTGAAGCTGGCTCAGGCGATGAAAGAGATTAGAGAGTCAATGAGAACGTTTTTTTTGTATTGCTTGTAACTGTTGTTTTAACTTTGTCTTTAAGTTGTTTTTTTTTTTTGTTCAAAAGTATACAGTTTTAGAGTAAATAAATTTTACCCGTATAAATAATAAACCAGGTGGATTTGTGGTCCCTATTTGTTGTTTGTTCCATATACGACTGGAAATACTAGTATTGGCTAGAAGATTGTACACTGTTTTTGTTGATGATTGTAAATTGACTGACCAAGTTTAATAAACAGTTGATATCTACATGCCTGGTTGACTTGTTCAAGATTCACTTTATCAGGTGGAGAGGGACTGGAAATGACGTTCAAGATTCACTTTATCAGGTGGAGAGGGACTGGAAAGTAGCTAATGTTACACCATATTTTAAAAGAGAGAAATCTGATCCTTAAACTAGACCAGTATGACTCACCAGTATCAGGTGTAAAATACTAGAACACATAATATGTAGAAACATCATAAACCACTTAGATAAAAACATATGTCCTTACTGCATACCATCATGGCTTTAGCAAATTTAGGTAAAGTGAAATTCAATTAATAGGTCTAACTGATGACTTTTTGAAGTGCTTGAAATACATGCCATTCTCCAACCTATTTGACAAACTTACAGA
>Bg-c35729 len=283 count=2 IPR:/ blastx_SP:/
TTAATTTTGGCAGCAAGGCAAGGCCAGCATGATGCTCTTAACACCTTAATCAATGCAAGAGCTGATATTGACTTTACTACCAATGAGGGATACACACCTTTATGGGAAGGTAGCTGTACAGATTTACATATGTTGTTGTTAGTTTTAGAGATGTTAGTTGGAATAAGTTAAAATTTCTGGCTAATCTAGAAAACCTTTCCTCTTTCTTTTTGTGTCTATTTAGGTCTGTGTCCTAAAGTTTATAGAATTGTTAGAGCTAAATATTTACATTTGCATTAAAAAA
>Bg-c31292 len=421 count=2 IPR:/ blastx_SP:/
AATTCCTAGTTTCTGTTATTTTGTAAACTCTAAACACTTATGATTAGAGACTTGTAGACTTCATGAAGTTGAGACTTGTGCAAAGAAACATCTGTGTCCTTAGAGGAAATAGAATTGCTTTGGGAATGATTTTCAGGATGAATTGTTTGTTTCTATTTCCCAATCAGATCATCTGTTGTAGACCTAGACAATAATGTCAGTCAATGACTCACCTGATCTTGTATATATTAAATTACTTTGTTCTTATTTTCTTGGATCAAGTGTGTGTGTTTGTCAGAATGGAAGGGCATGTAGTGGATGTATTGACTGCATTGCATTCACTGTCACTTTTTTTGAAATTCAAAGTCTTTTATACTAATATAAATTTCTGTGGGACAGAACCTATGTGTACAATTATGTTCAATGAATTCTGTCCAATGTG
>Bg-c23916 len=464 count=3 IPR:/ blastx_SP:/
TAGTATTAATTTTTCTTTTAATGACATGACTTCCTTTTGTATTTACACTCAAAAGTATACGTTCATTAGATACATAGATATAGTTATGTATAATAAAGTAAATAATACAGTATTAGTACATTTAAGATATTTAAGTGGGCTTTGAATACTTGGAAACAATGTTGTTTGACTTCTTTTTACTGTAAAACTTAAGTTTAAAAATTATCTGTAATTTTTTTTTTCAATGAGGCCGTTAGCATAAGAGAAAGGAGGAAGAAAAGCTATAAACATAGGGAGGTCAATGCTTAGAATATAAGATCAATATCACAGATACATTTGAAAACATTAACCATTTACTGATAGCTGATAACTCACCAATGGACACAATAATAAATAGTAACAGTGGCGTAGCAAGGGATTTTGGGTCCGGGGGGGTGCTGCTTGGCCTCGTTTAGGGGCCCCTGAATTTTTGACATGCGACATCA
>Bg-c5801 len=749 count=20 IPR:/ blastx_SP:/
ATCTTTTTTTCATTTAATTCCAATGACTATTTGTATTATTTTGCATTAGATGACAGCAGAATTTTAGATATCTGCTGAGGCAGTAAACATTTTAGTGAACACTATAATACATATAACAGAGAAAAAGTATACATGAGATTGAACTGATTTTTTAAAATACATTTTGGCCATTTTATTGTTTCTGAACATGTTTCTTTTGATCATTGAAACTACTTACCATAACTGGATAAGATTATCAGAAAATATTTAAATGTCAAAATTTATTACATTTTATTAGGTATTTAAATCCAATTTCTCAAAAGTTACCAAATTTGTGTTGTTTTTTAATTGCACAGTAGAAATTTGGTATTTTTTTTTTATCTCGGGTAATGGCAAGTTGGAACAGCCCTTTTATGTTTTATTTACCTTTTAAAAGTAAATGTTTACCCTGATCAAGGTGATATTAATATGCCTAAATATTATTTTGTATGTAGCATTAAAAAATCATGCTACTTTTTTTTTTTTGTTTTGATCTTAGACTATAACATAACAAAGAACATTATTATAGATTCATAAAAGACAACATATTCTATATTCTGATCTTTCTTGGCCATAGTGTCACAAGAAAAAGAAAGAGACTTGAAAGATGCATCAAATAACATTTTGACAAATACCAATTTAAATCCACATGAAAATGCTCAGCACAAACAGTAAACTATTTTCAAACAATGTAATTATTGAAATCCAAGACAAAATGAGCTTGTATGATT
>Bg-c27852 len=257 count=3 IPR:/ blastx_SP:/
TGTTGGGAATACAAGTTTTATTCTGTCATTCATGCACTGACATAGATAGATTCAAATGTAATCAGCAATCAACATTTTAAAGAGAATGTTCATATATGCGAGTATATCATTATAATATGCATCTCAGTGACTTAGTATAACATTTTTGTCTAATTAATTTGAGATTTATTAGACTTGATGTAAACAGTAAATGGTCTCAATAAATGAAGTTAAAAAGACAAAGCTCAACATTTCTTGCAAAACCCCTTAAACGTAAG
>Bg-c34922 len=294 count=2 IPR:/ blastx_SP:/
TCTTACAATTTTGTATTTCAATTGCTTCCCATGTTGACCTGGCAAAAATCACTAGCAGTTGAATAAAATGTCTTGAAATAGTTTGCTGGTCATTTATTCTTCACTCTTGTCTTGAACCCATTACCCAGCACTCGTGGCTAAACATATTGCGGTTTTGATGATTATTTTGTGAACTTCCCCGATGACTTCATTTCTTTAAAAATTGGTTTTTTTTAGTTCGAAACTATAAATTATCTCCTTAGAAATTACTAAAAAAAATTCTTTTTTTTCTAGTTTTCATCCTCATCTTCCATA
>Bg-c26191 len=302 count=3 IPR:/ blastx_SP:/
GTGACTACTTCGTCAGGAAGTTGGATTAACATTGAGTTGTCAATGAAATAGCGGGAACAAGGATAAAGTTGTTCCTAGTTGAGTCCGTTCGAAGTTTAACAAGTGTTAGGGGAAAAGCGTGCTTGACGACAATAGAAAAGAGTAGCGTATTCAAGTTGAAAGTTCAAGGACTTGTGTTATCCTAACTTGCCGTGGTTTACACTATACAGACCTTACACCATATCTTAGGGTAGTATTCTAAAAGGTTACAATCTACATAGGTCTTTAAAAATAAAAAAAATAGCTAATACAATAGTTGTTCG
>Bg-c24953 len=370 count=3 IPR:/ blastx_SP:sp|Q8CIF6|SIDT2_MOUSE/SID1 transmembrane family member 2 OS=Mus musculus GN=Sidt2 PE=1 SV=1/3e-25/370-17/649-765 // sp|Q8NBJ9|SIDT2_HUMAN/SID1 transmembrane family member 2 OS=Homo sapiens GN=SIDT2 PE=1 SV=2/5e-25/370-17/649-765 // sp|Q9NXL6|SIDT1_HUMAN/SID1 transmembrane family member 1 OS=Homo sapiens GN=SIDT1 PE=2 SV=2/1e-22/370-92/644-737 // sp|Q9NXL6|SIDT1_HUMAN/SID1 transmembrane family member 1 OS=Homo sapiens GN=SIDT1 PE=2 SV=2/1e-22/63-16/747-762 // sp|Q6AXF6|SIDT1_MOUSE/SID1 transmembrane family member 1 OS=Mus musculus GN=Sidt1 PE=2 SV=1/1e-22/370-92/644-737 // 
GATTAGTTTAATTTAGAAAGAAAAGTACAAAGATCCAGCCCATGTAATTACTGATGTACAGATGACTAATTTTAACTAACCAGTTCAAATGCTCCTTGTACAGCAACTTCATTATGATGTAGAAGATACAATAAAGCAACAAATTTCCTATAAATATTGCTAGAAGGTAAGTGGCAAAGTCTGAAGGCTGCTTTATGGCTCCATATAATGCAAAAACCCAATTAATAATATTACCAATTAGTAAAAGAGTGAAGCGACTAGGATAAACTGGGTTAGTACATTTTCTTCCTTCTGTTATCACAAGACGCCATATTCTTCTAAATATATTACAATCAATATTCCATCTCCCCATATAATAGACTTGAGCTGT
>Bg-c41112 len=232 count=2 IPR:/ blastx_SP:/
AAAATGCTTCAGAGACACCCTCAAAGCTTCTCTGAAGGCTTTCAGCGTAGACCCAGCCACCTGGGAGACAGAGGCACATGACAGAGCACCATGGCGTCGCGCTGTGAAAACTGGCGCACAGGTTGCTGAGGAAAAAAGAACAACGCTGGCAGAAGAAAAACGCCAGAGAAGAAAAGCAAGATCAATGACACTAGCTCCAGCCGGAATAACCAGCCAGTGTGCAGACCAAACA
>Bg-c37786 len=263 count=2 IPR:/ blastx_SP:/
AATGCAAGGAAATTAAAAATGCCGTCTAACTTAAAGTTCAACATGTAGGGATGCTGGTATTTTCATATCTCAATGCTTACGAGCACAACCTTAATTATTCTGTGAGTAACACAATAAGTCTCAAACAATGAATTATGCAATAGGATGTTTGTATAGCATATATCAAACTCAAGACCCACAATGGAATTCATGGACTTTTAATTAACTCATTAAATTTTTTTTTAACTCACGTCTTTCTCATTCTTATGTGATTTGTATGTTTA
>Bg-c4424 len=463 count=28 IPR:/ blastx_SP:/
CCATACATTACGCCATAAGTTGGACTTATAACCCGTAACTGTTGTATCCCGTGTTGTTTTAGCCGCTTTGCAGCAGCACCAGAGTTTCCTCTCCGGGGCGCATTCCTGGGCCTTGGATAAAGGGGTCATGGGTGGCCCATGCGTCATGATCCCTCTCTCGACCTTGCTGACGTGATCCAAAGGAACGCATCGCATTACATTTGGCACCAACTCAGTTGCAGAAGCTGCCGGAGGGAATTTCATAGCTGATACTCCCAACGCCTAAGGGGCTTCACTCCTGATTTCTCCTCGAGGTTGTCTCCTGAAGCCTCAGTACCGCAAGGCAGCGGGGGTTTGAAGTCAGAGTACCCCTCTCCTAGATGAACTGCCTTACTAGGCTGACGAGCTCCATCTGCCCGAAGCTCCTAGGTTTTGNGGCGCCAGGTATCCGCCTTCACCCATTCACATTACAAGATCGTACGGG
>Bg-c7047 len=587 count=16 IPR:/ blastx_SP:/
ATGTTGTCGATTGCATTTTCAAATCGATTATTGCAACTCTTTTGAAAGCTGACAACCAGTATTGTCAGCTTATGACCTTTAGCACAAACAACAAAAACGCCAACACCTGCCTCCAAGACTGTTCCAGCACCGAACTAGACAACTTTCGTCGAGCGTCCTGTAACACGTCACTGGCGCAGCTACAACCGTCAGCGTTTACAAAAGCCGTCATGTCGACCTCACAGCCGTGTAAAAATCAAATAGAAAACTGCGCTTTCGGCAGCAATTTGGCTTTTGCCTCGATTCAGAGTGGACAGTATTGCAAGATCATGAACAAATATCAGGCAGGGGCGGAGACCTACGCTTGTTTAGTTGGTAGCGGTGGGTGCACAGACAACGAATATCAGATTATGGAAGACGCAGCTTGCGACAATGAATAAATACATAGAACGAGTCTGGGAAACTGAATGTTCATTCTCTGGTAATATGATGCTCTTAATAAAAATCTTTAAAAATTTTTACTACCGTGTTAAATTAAATTCGCTGATTAAAACGAACTGTTAATGTGTTGTTTGTTTTTTTTTAAACTTCTATTATTTACACTATTA
>Bg-c29325 len=830 count=2 IPR:/ blastx_SP:/
TAACTGGGGCTTTCACTTTCATAATAGTTAGCAGATCCCTTTTGGGTGCTAATAGAGTTACCTAACTTGATCATCAGAGGAAGAGCTAAGCTTTCTGTTTCTCTACTTGTAAATAGTAGCATAAATTGTAGTCCCACTAATTTTGATCAAATATCATAGTTTTTGTTACTGTTATTGAATTAATGTGATATAAGAATGCAAACTAAAGATATGTTTGTATTGGAGCAGGTGCTGAGTGTGTTTGTGGTATTTGTCTGTGATGTAGCAGTATTAAATTCTTATCTAATTCTAATTTGTATTTATGAAATAATTTTTTTTTATTTATTGTAATAAATGTAATTTTGTTTTGTGCAATTCATTTGGTATTTCTTTGCTATCTAGGCTAGCTAGATATGCAACTTCCTCTGTGACAATGTGGCATGAATTTACATTGTTTGACTGCCATTGTTAGCATAAAACTGACTGTCTCTTGAATCACATAGTGCTGAAGTGCCATAAACATAGCTTCCATTTGTATTGTTATAAATGGTGCTGGTTTGTTTCTGTAAAGGAGTCACAGTTAGTTCTGTAGCAATCTGCTTGTGGTATTTGAAATTATGCTATGCTGAGTTAGACTAGACTGTTACAGTAATCTTATTTTTGAATTCCATTGCTAAGCTTTGAATTCCATTGCTAAGCTAATTTGGTTTTGTTTCTCAAACTTTTGTATGGAGATATCTACAGTTGTCTACTATCTGATCTAATAAAACACAACTAGCATCAATAAACTGAAGAAAAAAACAACAACTAATTCTTTATAATTGCAAAAAAAGGGAAAAAAAGAACACTTC
>Bg-c23518 len=554 count=3 IPR:IPR005108:HELP blastx_SP:sp|Q26613|EMAP_STRPU/77 kDa echinoderm microtubule-associated protein OS=Strongylocentrotus purpuratus GN=EMAP PE=2 SV=1/4e-48/116-553/49-180 // sp|Q9Y1C1|EMAP_LYTVA/77 kDa echinoderm microtubule-associated protein (Fragment) OS=Lytechinus variegatus GN=EMAP PE=2 SV=1/9e-46/110-550/25-157 // sp|Q4V8C3|EMAL1_RAT/Echinoderm microtubule-associated protein-like 1 OS=Rattus norvegicus GN=Eml1 PE=2 SV=2/8e-44/98-553/168-308 // sp|Q05BC3|EMAL1_MOUSE/Echinoderm microtubule-associated protein-like 1 OS=Mus musculus GN=Eml1 PE=2 SV=1/8e-44/98-553/168-308 // sp|O00423|EMAL1_HUMAN/Echinoderm microtubule-associated protein-like 1 OS=Homo sapiens GN=EML1 PE=1 SV=3/3e-43/98-553/169-309 // 
GATCACTTCGTGAGCAGTGCTTTCTTTCTTGGACACTCATTTTCTTGATATGGAAGACCGGAGCACATCTCATGGGAACCTTTTGTTGCATCCAGGTCGGAGCATGGCCAACAGATCAAAAGAGCCCCAATGGAATCAAGAGGAGGGTTCACTCAGGTTATTCTTGAGGGGACGTGCTCTAAATTTTTACGGACCAAGCGACCTTACAGATTACAATATCAGCAAACAGGCCGATGCGCCATCAGAAACACTTCAGCTAGAGTGGGTATATGGCTATAGGGGCAGAGACTGCAGGTCTAACCTTTATTATCTCCCGACTGGGGAGATCATTTACTTTACTGCAGCTGTAGTTGTTCTTCACAATGTAGAGGAGCAGACACAGCGGCACTATCTGGGTCACACAGATGACATTAAATGTCTAGCTATTCATCCTGATAAAATCAAGATAGCCACTGGTCAAGTAGCTGGGCATGAGCTGAGAGAGACTAAGCCTCAAGCAAAGAAAAAAAGTGCAGCTCCAGATGAGAGCCTACCCCATGTCAGAGTCTGGGATT
>Bg-c3717 len=497 count=34 IPR:IPR003102:Coactivator CBP, pKID blastx_SP:sp|Q03061|CREM_RAT/cAMP-responsive element modulator OS=Rattus norvegicus GN=Crem PE=2 SV=2/6e-10/16-378/30-145 // sp|P27699|CREM_MOUSE/cAMP-responsive element modulator OS=Mus musculus GN=Crem PE=1 SV=2/8e-10/28-378/34-145 // sp|P79145|CREM_CANFA/cAMP-responsive element modulator OS=Canis familiaris GN=CREM PE=2 SV=2/6e-08/79-378/54-148 // sp|P27925|CREB1_BOVIN/Cyclic AMP-responsive element-binding protein 1 OS=Bos taurus GN=CREB1 PE=1 SV=2/6e-08/61-372/29-127 // sp|Q1LZH5|CREM_BOVIN/cAMP-responsive element modulator OS=Bos taurus GN=CREM PE=2 SV=3/1e-07/118-378/60-148 // 
CCTGTGACGCTGATGGTGTATTTTGACGTGGCGACTACGTATTCTACAAATTTATCCACTCCGATTGCAGCAAAGTTGGTGACGAAAAACATGTCAGCAGGAAATGGTCCTGGAACAGCTGACATTGAAAATGGCACCCAGGGCATTTCAGTGGTCCATGTGAGCATTCCTAACCAAGCCATACAGGTTCAGCCTGTCATTCAGGCAAACCCAACTGTCATTCACAGCGCAGGCAATTATCAGACAATCCAAGTTGTTAGGGTTGCAACTGTTGATGACGATTTGTCATCTGATGACTCTGATGCCAAAAAGAGAAGGGAAATCCTAGCAAGAAGACCATCTTATAGGAAAATTCTGAATGATCTTTCCTCTCCTGTTAATAAAATGGAAGATGATTCAAACAGCAGTCAAAGTCAAGACGGTCAAGAATGTGGAACAACTATTGTACAGTATGCACAAGGACCAGATGGCCAATTTATTATACCAGTTGGAGGTAC
>Bg-c1339 len=653 count=99 IPR:/ blastx_SP:/
GGACGTGGGTGACATGATGAACCTCCCCCAAACCATCCGACGTCTGCTGAAACCTTTCTTTTATTTTACTAACACTGGAACTAAAGAGCTGATTAGGTGCAGGTTTGGAAACCTGCCCAGCATCCTTTTCCTGAAACCTCTTCTGAATGGAGAGAAACTGAGAGGAGTTCTTCCCATCTGGAGGCGCCACATTCTCCACATCCTCCATCCTTGTAAGGCCGCTGCGGGCACGGCTAGCAGCCAGAACTTCTAACCTTGTATCTACTGTGCTAGACTGAGCCATGATACTTTCAATGAATCTGATGCAGGACACAGGGCAGTAGGACGTCCTTTAGGACAGCAGACCTCCATGTAATCCCTAGGCAATGTCCTGCAGGACTTTTCTGGCCAACTCTGACAATCAACAAACGCTTGAATACAACTCTGCAACAATTTATCTGATCTAAAACACAGGGATGAATGAAGCTCCAAGACAATGTGTTTATGACATCCTTACATCTCTGACACCAATCTTTTGATCATTAACCAGTCCAGATTTCCAAAAGGACAACCAGCTTTGCAAACCCTAAAAAACTTAAGAAAAAATTAACAAATACACAAGTTACTAAAAATAAGCCATTTTAAAAATTTACCACTTGTATATCNGATGCAAC
>Bg-c40495 len=240 count=2 IPR:/ blastx_SP:/
GTATGAGTTTGATACCAGTCAATACTTGACTTTTGGTGACTCCACTGAAACACTATTTGGACTTAGTGTTTTCTCTTTATGATGACCTGATTCACAAACAGCATCTGTTTTTGTTTTCTCTGATTTGTGCAGTTCCTCTTGACATTTTTTTCCCTTCATTTTTTTTGCTACTGCCAGATTTTACTGTTTTTTTGATGATCTTTGCTATTTCCTTCAGATGCAGAGCTAGAAGACTTGTCA
>Bg-c10129 len=611 count=10 IPR:/ blastx_SP:/
TTTTTTTTGGTAACAAATTTTATTCTTTTATTTTTTTCTCAACATTTTAGTAACAATGTTTTGAAAGGTCTCCTTTGACTATAATGATTATGTGGACATCCATTATTTGAGAGTCTTATATTATTGTAGAACAAGGTTCAATAAGTTATAGGTAAAAAATATACAAGACATATAAAATCATTCACATGTCTTTCTGTATCACAGTCTGAGAGTGGCTTAGAAACAAATATCTTCATACATTTCTTAATGTCAATATTATACCAACATTTTACCAAAACTTATACTGATAATACATCAATGTTACCAGTATGCCAAAATTATATAAACTCTTAGTACACCAGTACTATAAAAGCATTATAGTATTAGTGTATAACAAAACAGTGGTTTAAAGAACAATGATAATCTTTTAATCTTCATTTCTATTCATTCAAATATTTTTTTTTAATGTTATAGACTTATAATACTGTAAGACTATTATTAAAAATGTGTCTACTGTCTCTTAGTCAAGTGTTACAAAGATATAGTATGACCTGTGTTAGGATTAAACACTTGTGCCATATTCAATTAATAAGAAGGACCTATCAGAAAAAGGCTTTTCCCTAAGTTTCAAA
>Bg-c15163 len=439 count=6 IPR:/ blastx_SP:/
GAGTTTTCGACAATGTATTAAATCTATAAATCTTTAGCCAAATCGCCAAATATATTTAAGCATATATATTTATAGAAAATAGTCCAAAAATCCTTTAGTTAAACTTAATTTATCAATAAAGCCATTCTATTTAAAGCACCGCTCAGCGATAAACTATTTATAAATTAATTATGATCTTTACATATATAAATTCTTATTAACTATAATGCGTGAAAGAGATTCGTCAATTGAAGAGCCTGGCAGGCTAACCACTTGACCATTAGGCTAGATCTCGGACCATTTCTGTTCGACTCATTAAAACTTGCCGTGAACAGCTTATCTTTCGACCTGTCAGATCTGTCTTCGAAATGGCAGAAGAAATGAAATTCGAGGTAAAATCCAAAGATCCTAACTTCATAAACCAACGAAGGAGGAAGACATCGTTCAATGTTTGGTTAGG
>Bg-c11142 len=495 count=9 IPR:/ blastx_SP:/
TTTTTTTTAATTGATTCATGTTCTGCTAGGTACAGTAAATAAATGTTTTTATGGATCAACTTGATATGAAAACGCTGGAGGGAGAAATAGCGTTAACAATGATTTATGGGAACTAAAAACAACAAATTAAGCCACATCTGTGAATATAGAATAATTAGCTTCCCTTGTTGGTATCAAACACAAATAATTAATTACCAGTAATTAACTGACTGAATGGTTACTTTCTTTTATTGATTCATGTCTTGTCTACGCCAATGAATAATTGTGCGAAGTTTCAACTTGATCCGAAAATGGGTGTGGGAGAAATAACATGTACACACTTTTTACCAGACAGATAGACAGACAGACAGACAGACAGACAGAGTGAGTTGACATAAGCTTTGTAAAAAAAAATATGTGTACGTTTGTCAAACAAGAAGGGTCGGTGAATTTTTTAGTCAAACAGTTGTATATTTTAATTCCTTATTCCAGTGGTATGTGTGTTGGTTTTAGGGA
>Bg-c9393 len=657 count=11 IPR:/ blastx_SP:/
TATTCTGGCATATTTGATTGGTTGAAAAAAAACAAGGACATTGGCTGCACCCTAAACCATGATTTATTCTAGTATTATTGAGTCTGTCTATTTAATGACAAAACTCGTACATAGACCAGGTAAATACTGTGAACATTGCTACTAGTGATTATTCTTTATTCCTCTTGGATTTGAAACAAGTCTCAGGTCAAATGTGAGCTTGGAATGAATGCTTTTTTATAATTTGGTTTGGTGTCAAGATGTACACACGTTTAGAAACCATATTGCTAAATATAGAAATCTGTACTACCAGTATTGGGATAAAAATAATTTTGTTTCAATACAATGACTGCCTGAGCATAACAGTTTGTTTATTGTGTCGGCCAATACAATACATGTATATTTTGATTGTTATAAATTTGTGTATGGTTCTAACTTGATTTTTATATATATATATATTTGTAGCTTTTTAATATTTTTTTCTTCTGTGGTTTTAAAATAGTTGTGACCTTTGACACAAAATGTTTGACTAATGAATACAATGTTTGACTAAAGAAAAAAAAAAAAAAAANAAAAAAAAAAAAAGTAAAACGGTCGGTGTCGTTCGGTTTACCGTTAACCCTACGTCGTTTTTAAACTTTTTTAAAGTTAAGGTAGGTTCCGTACCTAATTTTTACG
>Bg-c23780 len=487 count=3 IPR:/ blastx_SP:/
CCATGGGAGCAAACTAATGCGGATTATAGAAACAAAGGAAATTAAAAAAAATACACTAGAGTTATTGTTCTTACTGCAATAATTTCTTGTTCTGAATATAAAACTTTTAAAACGTTAGAAAAATTGCTAATAAATTATAATAATCCTTTCCCTAAAAATAATCATGTACATAATTTGAAATACTGACATTATTCCTGAGTTAATGTTGTTGTACAATATACAATAAATAAATATCCCTAAAATGGAAGCTTTGAACCTGACTATCTCTTCATTACCGCCGAATGTTTCAGTAAGTCCTGTATCTGTTGGACCATCTCGTCTTCATAGGCCAAGTAAACAACATGGCCGCCTCGAGCAAACACCAAAACTCTGCATAGCCATCCAGGTGGAACGAAAATTGGGTCCCTATTTGGTTTTGAGTCTTTGGAGAACCTAACTGTATTGTCTTTGGGAATACGCAGTTGCTGATAGGCCATGTTTCTTGTTG
>Bg-c5817 len=680 count=20 IPR:IPR000781:Enhancer of rudimentary blastx_SP:sp|Q93104|ERH_AEDAE/Enhancer of rudimentary homolog OS=Aedes aegypti PE=3 SV=1/6e-41/5-304/1-100 // sp|Q94554|ER_DROVI/Protein enhancer of rudimentary OS=Drosophila virilis GN=e(r) PE=3 SV=1/1e-39/5-301/1-99 // sp|Q24337|ER_DROME/Protein enhancer of rudimentary OS=Drosophila melanogaster GN=e(r) PE=1 SV=1/3e-39/5-301/1-99 // sp|P84089|ERH_MOUSE/Enhancer of rudimentary homolog OS=Mus musculus GN=Erh PE=1 SV=1/4e-39/5-301/1-99 // sp|P84090|ERH_HUMAN/Enhancer of rudimentary homolog OS=Homo sapiens GN=ERH PE=1 SV=1/4e-39/5-301/1-99 // 
AAAAATGTCTCATACAATTTTATTAGTACAGCCTAATCAGCGTCCAGAAACAAGGACATATTCAGATTACGAATCTTTGAATGAATGTCTTGAAGGTATTTGCAAAATTTTTGAAGAGCATCTGAAGCGTTTGCATCCACATGATCCATCCATTACCTATGACATCTCTCAGCTGTTTGAATTCATTGATCAGCTAACTGACTTAAGCTGTTTGGTTTTTCAGAAATCTTATGGAACATACACTCCTCATAATAAAGATTGGCTGAAAGAAAAGATCTACCATATGTTGAGAAAACAAGCTGGAAAATGAAAGAACTAGTTCTACATTTTGTCATGCGTCATTTCATTCAGTGTCCATTCTTTGTAAATATGTTTTTAACATTTTATTGAGCATTTAAACAATGGTTATTCATTTGTAAAATGTAATCCTATTCTTATATAACATATTTTGAAAAAGCTTTAGTAATATTAACCTGCAATGGAGTAAAAAAGTATAATCTTGTCTGGAATTAAAAATTAAGAGGTGATTAAAAAGTTTTGGCCATTTCTTAAAAATTAGTATAATTTTGTTTAAACTAAATTTTACAAGGATTAAATGAAAATTAATTGCATTTCTTTCTTTTTAGTTACATGTTTTAAATTGGACTATGTAAATGTATAATAAATTAATTAAAAAAACT
>Bg-c26467 len=291 count=3 IPR:/ blastx_SP:/
AAAGATGAATCATCTAGTTCATCCGATGATGATGATGATAAGAGAAAGAAGACTGAAAGTAAAAAACCAGTAAGTAAAAGTCCAGAGAAAGCTGTTAAAAAAGAAAGTGATCAGAAGCAGAAAGCTGGTGATGGGGCCAAAAAAGATGTGAAAGAAGCAAAAACTTCCAAAGTAAAAGACAAAAAGAGAGATGATGACTCATCGTCATCCTCATCAGATGATGAAAATCATACATAACAATAAAATAATCAGGCGCGACTCAAAATATCTAAAAACTAAAAAAAATTTAAA
>Bg-c21969 len=270 count=4 IPR:/ blastx_SP:/
TTACAATAATAGTTTATTGAATCAAAGACACTGAAAACATAAAAAATAGAAAGGTGAGAGGGGGGTGGGAAGTGTGGGTGACACATATTGCAGTTTCACAACTTGAAAGAATAAAACAAACAAAACAAAAAAATAAATACATTCTGAGCTCCAGCGGCTGAAAATAAAATAAATAGCCGCCCACACCCCCATCCCCCTCACTGGTCCATATCACAGTTCCAAGACACATATACACACACACACTAGTATTCACTGCTCCAATATTATCAA
>Bg-c25607 len=331 count=3 IPR:/ blastx_SP:sp|Q9VJN5|WEK_DROME/Zinc finger protein weckle OS=Drosophila melanogaster GN=wek PE=1 SV=1/5e-05/59-286/228-309 // 
GCCCAGTGCCGCTCTTCCTTCCGTTGTCAAAACCGAAATTTCAATGGACAGCGATATGGAAGAGATCTGCGATTCTCCACCTACGACCATTCGATCCCCTGGACAAATGCACAGTTCTGAAGATGACAGCATTGAGTTGTCCGCCCACGGAATGATGGAAGAAGAGAAGCGAAAGCCACACTCTTGTGATCTCTGCAAGGAACTCTTCAGGTCTTTCAATGAGCTAGAGGCACACAGTGTCGACATCCACAAGCGCTATCTGTGTGAGCACTGCATGAAGTACCTTCACCACACGGCCCAACGACGGGACCGCCACGCGCGCGTGCAACGG
>Bg-c39000 len=253 count=2 IPR:/ blastx_SP:/
TGAATAAAATAATTATATTTGTTTGTTTAAAACAATAACTACATTGTCGTTTGTTGAGATTTGAGAATTAAGATCATTACATATCAATTTCTGTCTCACATTTTTCTCATGGAACATGCTGCAAACATTGATACAAAGTAACAAAAATAAATCAATGGACAATACTTGGGACTAGGACTTCACATACCATCTCTCACTTACTATAACTTAGCATACCATAAGCTACAGATTCTTTGACTTAAGTCACAAAATA
>Bg-c41051 len=233 count=2 IPR:IPR007110:Immunoglobulin-like blastx_SP:/
AGCTAAACCAGTGCCACAACTCCCACAGATCTTCTCTTCAGGTATTGAGATTGGCGGAGAGAATCTGTTGAGGTCAGGTGAGACACTTCAACTTACCTGCAACGCAACTTTGGTAGACGTGAGTAGTGAACGGATAACTTGGCTCAAAGATGGCAAGCCATTACTGGCTGAGAAGGATGAGCGATTCAGTATTTACACCACCATGACAGTGAATAGCCAGGGGATGGGGTCAA
>Bg-c13563 len=448 count=7 IPR:/ blastx_SP:/
TGAAAATAAAAAAGAGGCGGCCGCTGCGGCTCAACCCAAAGTTGTCCAGGAGGAGAAAAAGGAAGCTGAGAAGAAGGAAGTTGAAGAGAGCAAAGAACAAAAAAACACAATCACCAGAGAAGAAAGAAACAAAGCCAGAACCCAAGCCAGAGACTCCAAAAACAGACATAAAAAAAGATGTGGTCAGGAATCGACGCAGAAACTATTGGTTTAACAGAAGGAGAGGTTTTTACCGCGAGGAAAAAAAGGCTGAAGGCCCTGCTGCAAAACCAGTGTACCCAAAGCCAGAAACAACCCCACGTGGTCAGAGACCAGAAACATCTTCACGTGGTCAGCAAAGCAGAACAGATCCATCGCCACGTGGTCAGATAAGCAGACCAGAAGCATCTCCACGTTGGTCAGCTAAACAGACCAGTAAGAAGACGGTCCCCTTGGGTACCGGGGAACA
>Bg-c28327 len=246 count=3 IPR:/ blastx_SP:/
ATAAGTCAGACAACGAGAAGTCGGAAATGTCCCAGAACTCAAAGTCCGGACCTAGTGTCACTGTTGAGCTGGGTGACCTAGAAGTCCAATCAGATGTCCACGACTTCTATCCGCAAAGCAAACCACAGGCAGCGCAGCCCAAATCATGGCCAGAGCAAATTCCTCCTGTTCATGGGCCCGTACAGAGTCAGCCATGGATCGTACCGCCCCAGCCATGGTATGCGCAACCAACAAGGGTTTTCAGTA
>Bg-c19212 len=719 count=4 IPR:/ blastx_SP:sp|Q5SS90|CG057_MOUSE/Uncharacterized protein C7orf57 homolog OS=Mus musculus PE=2 SV=1/4e-08/110-664/30-200 // sp|Q8NEG2|CG057_HUMAN/Uncharacterized protein C7orf57 OS=Homo sapiens GN=C7orf57 PE=1 SV=3/6e-08/134-460/33-134 // sp|Q8NEG2|CG057_HUMAN/Uncharacterized protein C7orf57 OS=Homo sapiens GN=C7orf57 PE=1 SV=3/6e-08/82-114/18-28 // sp|A0JNL1|CG057_BOVIN/Uncharacterized protein C7orf57 homolog OS=Bos taurus PE=2 SV=1/8e-08/134-643/33-201 // sp|A0JNL1|CG057_BOVIN/Uncharacterized protein C7orf57 homolog OS=Bos taurus PE=2 SV=1/8e-08/82-114/18-28 // 
GATTATTCATATTGATATTGCCATTACATCCTCTTTTGTCGCCCCCTAAGGGGAATGCCTTTAAGTCAAAATGCAAAAGAATGTCGGTGGTTCTACCATGCCCCAACTAAAAAAAAATGTTGGTGCTCCAAAATTGGACTATCCAGCGCCTTCCAATATCCCAGGATTAGGCTTTGATGATCCTGTAGATGTAGAAAACCAACACTGCAAGGAAATGGTGTTTAAAGAAACGGATACACATTACATTAGATTAGCTAAAATGGGAGGTAGAAAAGATTTGCTGTCCTTTAAATCTGACAATGAACGTCAGAAATCAGCAGGTCCAAGGGGGTATCCAAGAAATGATTGGTTTTATTTAGAAGACAACAGGATGCAGGATGAATACGAGCGAGGAAATGAAGAGAAAAAATCGTGGCAGTTCTTATTGCCAGACTACATGGTTCATCAGAGTTACAAGCCTTCCTTTGAGGAGCCCGATAGCAGACCAACACGTAGTGCTGCCCCTTTTTATACTGAAGTGAACTGTGGTATTGAAGAGGGAAGACAGGCAACAGATAAAACTGTCAAGATTCAAGAGCCAAGAAAACCAGGATTTGGAGTCAGATTGGAGAAGCCTGCAGCTACAGCACAGAAACCACCACCCAGAGATAAAATAACTAAACCAAAAGACTCAGAAACTTTGCAGGGAGAGAGAAAGAAACAAAACTTGATTGTTATGC
>Bg-c27718 len=260 count=3 IPR:/ blastx_SP:/
TCCCAAAATTCATTCCATGTGTTCCTCGGTTATTTGGATAAGGTTATACATTGATGCCTTAAGAGAATCTGAAAAAAAAAGATAATTTCTTCACCTTGGACAAAACTCTACTAGAAATGTTTAAAATATCTAGGTTTAGCTACTATTAGCTACTTCATAGTTCCAATCTGGAAATACTCTTAAGGTACTAAAAGAACTGGATGTCATGAAAGGAGATTTTCACCACATAAGATTCCCACACTGTGAAGCCAATGCACCTA
>Bg-c17200 len=447 count=5 IPR:/ blastx_SP:/
TCTTCCTTCTGGATTTCAACGCACTGATCTTCTAATCCGATGTTACTTTGTGCTGTGAGGGCATTTCCATCAGAATCCTTTGTTTCGATGTTTTTATCTTCTAAAGATTCTGTCTGTATTTCTCTTATCTCATCCTTTGCTTTCGAAACATCTTTTTCTCCAATCTCATCCTTTGCTTTCGAAACATCTTTTTCTCCAATCTCGTCCTTTGCTTTCGAAACGTCTTTCTTAGTTTTATCTTTTTTTCGATTTACTTTCTTCCTTTTTCTTTTTCTTTTCTTCTTTCTTTCTTCTCTTCTCTTCATTCTCACGTTCTTTCTTTAGTTTCTTCAAATGTTTTTCATTCGCTTTTTTATCCGTTAGAGCCTTTTGTGCATTGTCTGTTACTATCTCAGATTTTGACGACTCAACNTGCTGCCCCTNTTCTTTGGCATTTCGACGTTTTGG
>Bg-c1725 len=606 count=78 IPR:/ blastx_SP:/
GACATGGCATCAAAATGTTTTAAGTAGGCCAACATTACAAAATTGCAGCCAATTTTTGCATACCGGTACTGTTTTGTTTCTAAAAGGATAGTGCCAAATGTAAACTTGAGTAAGTGTCAAACACAGAAGTTTTTTTAAAAACTTACTGGAATTCTTTTTTGAAGACAACTTGTTATAGTTGTATAGTTCAATATTGTATTGTTATTCTGGGGGCTCTTATCACAAATGATTGAGGCCATAATTTATTATTTCATTTATCAACCTATAATTTATATAAAAATGTCATTCCCATTCACCTGAAACTGTGTTGGTCTGTGAATTCCAACTCTCTGTCTGTGAGAAACTTCTTGACAATCTTCTCCCTGCTTGAGATTGTCTTGCCTAGAATCGGCAGCTCATTGCCTGAAGAGATTCCTAAAGACCATTGGAGCGTTTCCATACTGTCAGTGTAATCTTCATCTCTAAGGGAGATAAAAGGCATATTACAGTTCAGAAGTTTAACCCAATAGCTCCTACTTCAGGTTAGGAAAAACCCAGGCCCAGGCCCTAAGCGAAGAGGGATAACCCTGAATTTAAAAAATACCTAAAAAATAAATACCGGTATTT
>Bg-c41113 len=232 count=2 IPR:/ blastx_SP:/
CCTTCGGCGTGGAACGCATCGGCAATGCGCTGTTGCTCCGACAGACCAGGTCCAGTGACTGCGCCCACCTGATAGGAGCGGTGGCTGTTGGTCTCTTCAACGGTGGCTGCGTCGATGCCCCAGAGTCCGGCGAGCAATGTGGCAGCTTCCTTCGGCTGCGCCTTCACCCATTTTCCGGTCGCCGAGAGTTTTGCGAAGATCACGTTGAGGACCTCGGCGTGCTTGTCCGCAT
>Bg-c29566 len=716 count=2 IPR:/ blastx_SP:/
CGTCTACATAAGCTAGCCTACTTCCATGACTACTATGTTGTCCATTCCCTTCGCGTTCACACCAACAGCACAACGTAGACAACGAGAACCTGGATGTTTTAGTAAACCATAAGTATTTTGATTGACATTCATAGTAGCCGTAGACAATGTTTGGCATCATCTTTAGCCGCTGGAGAATGGAGAGCTTTATAATCAGCATTTGGAAATAGCTCCAACATTAGCTGCTGGAGGATGTATATTAGGACAGTAGGCTAGCTTATGTAAAAAAAAAAAAAAAAAAAAAAAACTCTAATGAATTCTATATGATAATGATCAAAGCAAAATAATTATTTGTGACCTACACACTGGGGATACCACATCTTGGCTTGTATACATCTGTAATATATATAAGCATCTTGTCATTTATTTCTAATCATAGTTGCTTAATTGGCACAAAAATGCTGTCGTGAAATTTGATAAAACATACTTTAGGCTCTTTCGCCAAGTCTGTGATTTCTAAATGATTTATTCTTTTCTATGCTTTACATATTGAAATAGCCAAATTTTATGTTGCCACTTTTTTTTTAATGAATTTTCTTGTGTAGACTTTATTGTATGATTGTAAGAATGCTCAAGTTAGTGTAATTTCATCATTGTTCAACTGATTTATGCTCATGTTTGAAGGCAGTTGTTGTTGTTTTATCTTGATTGTTAACCATTAGAATTCTATTCCCTTT
>Bg-c39643 len=248 count=2 IPR:/ blastx_SP:sp|Q8BIQ5|CSTF2_MOUSE/Cleavage stimulation factor subunit 2 OS=Mus musculus GN=Cstf2 PE=1 SV=2/4e-06/2-127/166-208 // sp|Q5RDA3|CSTF2_PONAB/Cleavage stimulation factor subunit 2 OS=Pongo abelii GN=CSTF2 PE=2 SV=1/6e-06/2-124/166-206 // sp|P33240|CSTF2_HUMAN/Cleavage stimulation factor subunit 2 OS=Homo sapiens GN=CSTF2 PE=1 SV=1/6e-06/2-124/166-206 // sp|Q8HXM1|CSTF2_BOVIN/Cleavage stimulation factor subunit 2 OS=Bos taurus GN=CSTF2 PE=2 SV=1/6e-06/2-124/166-206 // sp|Q8C7E9|CSTFT_MOUSE/Cleavage stimulation factor subunit 2 tau variant OS=Mus musculus GN=Cstf2t PE=1 SV=2/6e-05/2-85/166-193 // 
TCCACAACTAGCATATGCGCTCTTACAAGCCCAAATAGTTATGAAAATAGTTGATCCACAAGTAGCAATGTCAATGCTGTATCGTGATGAAACTCAACCCCCGATAAATATGAATCCACCAAAGGTTTGATCTGATACATTTTTTATTATTTACATTTGTTGTTACATTATTTTGAAATAATATAGCTTTTACGTGGAATAATTATGTTTAAAAGACTAAAACTATCTCACTTTCACTAGATGTTGAA
>Bg-c9038 len=394 count=12 IPR:/ blastx_SP:/
CTCGACAACGTAGTGATGGTGTGTAGTTCTGAGGTTCGGGACACCCTCTGGACTTCACACATTCACTAGCGTAACCCTTGCTATTCATCAAAATCTCTCGGCAAAAGTTGGTGTTGGCCGTAATCATTGACACAATCCGGTGCTTACTATGTATTTCATGGTTTAGTTATTGTATGTTGTGCTAGATGTATTTGTAGATGATATTCTAAGCTTTGTACTCCGTGTTTTTCTGTTACCAAAACGGGGAATTCACGTATTTCCTCAGATGTATTCGCATGCACTTCGAAGTCGTCGAGTGTGAGCCAGCTTCGACGCCTTTTCTTGGCCTTAGTACCAGATGAACTTGAACATAAGAACACCATTTGTCAAATGTGTTTGTAAATGTTTATTATGA
>Bg-c5436 len=484 count=22 IPR:/ blastx_SP:/
CCTCTCCAGTTCTTGCTGTTGTCTCTTAGCTTCTTCTTCTGCTTTTTTCCTAGATTCTTTAATTTGCTCATACTGAGCAACAATTTTCTCTGCAGCCAAAGCAGATCTCTTTCTCTCTTCTTCTAATCTTTTCTCTTCTTCTTTCCTTCTTTTCTTTTCCTGTTTCTCAAGTTCCTTCTCCCTTTTTCGTTCCGCCTCCAGTTGTCTCTTGGCCATTTCATGGAGACGTCTTTCTTCTTCTTCCTTTTCTCTGTTTTGTCTCTCTATTTCTAATTTAATCTTTTCTTCTTCCTGTCGACGAACTTCTTCAATTTTCTGTATCTCTATTTGTTTTTGTCTTTCTAGTTCTTCTTTTTCTCTTCTCATCTGTTCTCTCTGTCTCTCTAATTCTTCCTGTTGTCTTAATAATTCTTCTTTTTTCTCTGTAGTTCCTCTTCTCTTGCTTTTTCTTCTTCTTTTAACCTTTCTTCCTCTCTATCCCTTC
>Bg-c42148 len=197 count=2 IPR:/ blastx_SP:/
GCTGCTATCAAAGTTATTGCCCCTGGCTTAATGGACAAAAATGCAGGCTTGGCATGCTCAATGAAAACAAGTCCAATGGTGGAAGGTTTGCGATGCTTCCGTAAAGTCATTAAGTCATGGTCATCAAAGAAATCTGGTCACCAACCATTTCTTCAATCCACTGAACAGTTGTAAATTAATGTCAAAATAAAGTGTCA
>Bg-c31355 len=417 count=2 IPR:/ blastx_SP:/
AAGGGAACGGGAGAGCTTCTTAAAGACGTCTCTGTACTCATGAGTTTATCTAGACTCTGCTTTGGACATTTTTAATTAAACGGGGAAAGTAACTCTTCATGATTGTAAAGTGTGTGCCTATATCAAGTTAGCGAGAGACAAAATACTAGCACCATGCCCGACAATAAGTACTGTGATTTTATTTGATATTTGTGCTCATTATTGAAACGCTGGAATGAATTAGAGACAAGTGTTCATACTTTGATTACAAGACAGGATCTGTTTTGATTGGACACAGCTTTCAACATGAAAGAATCAACTTATGGATGGTATCCCTCTGTTCAAGATATTTATCGCCCCAAAGGAAGACCATCGTCTTACCATCTGGAATGTCTGTACAAATATAACAATTCTATGTATTATCAAGACAAAGCTCTG
>Bg-c25980 len=312 count=3 IPR:/ blastx_SP:/
TTAAGATGTTCAATACTGAATTTTATTTCGAAATGCATTTGTAGTTTAAAATATGCTTATAACTTCCCAGAGCCAAGGCACTGGAACAAGAGGTTTTGTACACAGAGGATAAGAAAAGATGAGAGTAATAACAAACATGGAGTTTAAAAAATTGAAAGTTTAACAATACAAGCCTGTCACAAGGGGAAACTATCTACAAGCTTTAAGAAAAAAAGAACAATTAGTAGGTAATAAGGAACTTGAGGTAAAACTAAGAGTTCCATAGAAAAATTTTAAAAAAGTTGGATGAAAATAAAAACAATTACTAGGTTT
>Bg-c10236 len=524 count=10 IPR:/ blastx_SP:/
CACGTCAAAGCTGCCGTAGTTGGATTCTTGGCATTTATAACCAAATCGCATTAACATGCATTGATTTGCAAGAGTGCACATTCCATCATCATCACAAGATGGCGGGTCGCAGTAGCACCGATAACGGCAATGCATGCCGTACATGCCTCTAACGCAACCCTCTGGAACAGAAATGTTGGCCGGAGTTTGCATTGTACTTGGTGTGGCAAACGATTGTTGGACAAGGGAAACGGTGTGAGGTTTGACGGTCCACCTATAGGTCTTCCTTCATTCGGTCGCAGCACTGGGGTTGTAATATTGCTATCTGCATTTGTGTCGTTATTGGTGCTGTTCTGTGCTTGGCATGGTGTCGCACTAAGACACACGAGAAATAAGACCAGGGCCAGGGTCCTCATTATCTCTTGTGGCTGGTCCTCAGCTCGCCCCATTGATACACTTTGGTCAGCCCTTCATAACATGGTGTGACAATTTCAAAATGCCCTTATTTACTTTATTTTTGATGAAGCGCAAGGGTTGACTACCGG
>Bg-c4807 len=890 count=25 IPR:/ blastx_SP:/
GGCTAATACAGTTCTAATGGTTGACCTTACTCCAACTGGCTATGCAGAGTGATTGACATAAAAATAAAATACCATTCTTGTACCTATTTTTACCATACCTCTATTCAAATAACGCTTAATAGAGGCTGCAACCTGGACATTGATCTCAAAAAAAGCTTGAACTATTTTCCTATAAGTTTAACAGTTGATGTACATTGCGTTAAGTCTGTCCTTCTATACGGTTCAGAAACTTAGAGAATCACTAAAACAAATATGGAAAAGCTCCAGACCTTTGTTAACAGATGCTTACACCGGATATTAGGAATTAGGTGGCCAGAAAATATAACTATTATCGATTAGTGGGAGAGAAAAGCCCATAGTCCAAGACATCACTAAACGAAAATGGAGCTGGATAGGAAACACCCTGCGAAACCAGCTACCTATGTTGCAAGGCAGGCACTTGACTGGAACCCACAAGGAAAGAGGAAAGTGAGCAGACCCAAGCAAACCTGGAAGAGGTCAGTCATCAGTGAAGCTGAGGGTACTGGAATGACATGGGAGCAGATGAAGAAAGCTGCTCAGAAACGAGTACGGTGGAGAGGTGTGGTTGCGGCTCTATGCTCCCCTGGGAGTGCACAGGATTAAGTAAGTAAGTAAGTTAAGTACATTGTGGAGAAAAGACTCGTTTGCCACACTGGGAATACTCTAGTTTGACCGTTATAATTTTTCAAAGTAAAAAAAAAGAAACAAATTTAAATTTGGCTAGAGAACTAGATCTAGATGAAACATATTGATTCAAGAGTTTAATAAATTAATGTTCAGGAAAAGTTTGCGTATCGTATTTTAAGGCTAGATCTAAATGCCTATCATTGTAATATTATAAAGTGTAGTAGATCTATACTTTCACTTAG
>Bg-c42892 len=128 count=2 IPR:/ blastx_SP:/
ATTACTTGACTGTACAGTTCTATCATTCATTCAGTCTCTCTTTACAATTTGTTTGCCCATTTTTACATTAGTTGATTTCTCTTGACGGTTGTGGTGGTGTTTTTCCTTCACTATTTTTTTAGACCTTG
>Bg-c8897 len=529 count=12 IPR:/ blastx_SP:/
TACAGTGTAATGGATAATATTTCTGTGTGATTAGATTTAAAACATTCTAGATATTTACAAGTTTGACAGAGATTTATTTAACTTATTTGGTATTATAATGGTTAAATAATCATTAATATTATTATTTTCTTTTTGTTGTTTTATTGATTTGATCTATTTAATTATTTTTTTATTACTTATGCATCTGTTTAAAAATGTTTTCATATATTTAAAAAATAATGCCTTTAATTTTTTGTATTACATTTTATACACTGGGAAAATGAGTAATGAAAGGTAACCTACATTTTCAATCAGTGTTTTTTTTTGTGATCAAAAGGAAAGTTATTGCTGAAGACAAGCCTAGATGGCAAAAAGAGAACATGAATGACCAGTGTAGCCACATGAAATACTGAACTCCTAAATCTTCAGAATATTCTGAAGATAAGCCTTAGTTATTAATTTTATATGTGAGCTTATTTATGTAAAAAGTGTTATGTAATCAGAAATTAAAATGTTGACTTTTTTGTAAATAAAAGAACAATTAACAGCT
>Bg-c7725 len=862 count=14 IPR:/ blastx_SP:/
AGTCTGCATAGACTTGTCTGTCAGTATTACATCTACAAGGGAGATTACTCTTTTAACTACCTAGATCTAAGAGTTGTAATTTTGGAAGCTGCGAATTATTCTGATCTGATATGGCAGAAAAAAGCACGTCAGAGCCCATGGATAGGTGAAGCAGACGTACAGTTTAGTCAAAACGAGGCTGATCTTCACAAGCATAGGAACGATTGTAAGAAGAATAAAGGCCATGCTAATTTTATCCCCATAAATGCCTTCACTCTCCAGCATTTGCCAGAGGATTACAGGGATAAGGATATTTTTGATTTTATTAAAATGACTGCTGAACTGACTGTGAGGATAAGCACCAATTACGTCAGCCACAAAAGACCGGAAGTCATGCCACATACAACTACTCCCTATCTCTTTTGGAATAAACGTGGCGAATATTCAATGGCCTCTGGAACAGGTCGAGTGTGTGACGTAACAATGAAGTCAGACCCACGTGCCTCAGATGACGAGATGTACGAGGATCTAAAAGCAGTCGAGGAGCTGACTGATTTGAAGCGAATCTACGAGACCTGTGACGAATCAGATAAGCCCCACGAGGAGTCTGGTTGGGTCAAGGTCGTCCCAACAAGACACTGCTTGTGCGAAAACTGCAAGCAGTCAAAAGAGCCAAGCCGAGAATTCGGATGGGTCAAAATCATAACGGCAAGACACGTCATCTTCAAAAACAAGGAAGCCGAAAACGCAACTTGCAGAATATTCTTTGATGAAAACGAATGTCCCAAAACACTAATATACGGAGTCAAAATGGCCACGCTGAATAAGCCCAAACTTTTGGAATTGGTGCTGGTTGAAATGCATCACGTGTGACCTAGATTTG
>Bg-c22841 len=1201 count=3 IPR:/ blastx_SP:/
ACGCGGGGCAAGGGATCATAAATGCTTTCTGTTAGCAAGGGGACATAATGTGCTTTCTGTTAGCAAGGGGACTTAATGTGCTTTCTGTTAGCAAGGGGACATAATGTGCTTTCTGTTAGCAAGGGGACATAATGTGCTTTCTGTTAGCAAGGGGACATAATGTGCTATCTGTTAACTGGAGAGACATAATGTGCTTTCTGTTAGCAAGGGATCATAAATGCTTTCTGTTAGCAAGGGGACATAATGTGCTTTCTGTTAGCAAGGGGACATAATGTGCTTTCTGTTAGCAAGGGGACATAATGTGCTTTCTGTTAGCAAAGGGACATAATGTGCTATCTGTTAACTGAAGAGACATAATGTGCTTTCTGTTAGCAGGGGGAAAATACTGTTCCTTTACTTCTTTCGATGCTCAGCTTTTACTGCTCATTAAGTTACTGATATTTTATAAACACATTCAAAAGACTTTATTGTTATACTGTTTATATTTATACATGTTTTTTGTTGTTATTTTATTACTATGAAATGGGGGACCAACTGGGGTTTTACAGTCTCTTTCAGGTTGTTACAAATTGTTGACAAATGGAGGCCATGAAACAAAACATTCAAAACTCTTTGGGTCATTCTTTAGACCTACTTTGTATTTAAAAAAAAATTGTAAATAACAATGTACAGGCGTGAGCCCATTACCCAAAAGTTTTCTCCCTTTGAAATTTTGCACGATCTTTCGGACCGTTCCTATTTCGGACTCAAACTCCAGCCTTTAAAAACAATCCTCCAATCTCTTACCCTGTTTTACAACTTGACCCTAACAACCTCCATTCTCTCCACCCTTCGATCCTCAATTTGACCCAGATTAGATTTTTCCAATAAGAAACACAACAAATGAGCAAACCCCATGTTCCCCATTTTTAACCAATCAAATTTTTTTTTTTATTTTCCTGCCTTGAACTTGTTTGCAGCCTTGACCTTTCTCTCTCACCTTTTGTTTCTCCCATTCTATTTTTGCGAACTTCTTGCGTTGAAATAGTCACAATCGAGTTTGGCAGTTTTGGCAGAAGTCACGAAAAAATATTTAAAAATCTGAAGAAAGAAACGAGACAAAAAGTGAGAAGTGTTTTAATTTGTTAAAAGAACAAGTGTAAGCCATAAGATAAGAGGAACGTATAAGATAGTTAACTTATCAATGTATTTATTCTCTTCC
>Bg-c5118 len=245 count=24 IPR:/ blastx_SP:/
GTAACAACGCATCCGACGCGGGGGGGGGGTAGTTTTTCTTATGTACCAGTTGTTGGGTTGTTGTTGTTTTGGTACGGTATAATTTGTGTTTGATGTTCTATGGATGTCTGATCATGAATACAAATGTCTGCTGTGTTTGGACTGATGTGACAATCTTTGTGGGAATATGATTTGTGTCAACTGTTTGCATGTACATAATTTTTCATTTGGTAAATTAAAACAAGTCAATAATGGAAACTTAAAAA
>Bg-c39267 len=251 count=2 IPR:IPR008012:Proteasome maturation factor UMP1 blastx_SP:sp|Q5R9L9|POMP_PONAB/Proteasome maturation protein OS=Pongo abelii GN=POMP PE=2 SV=1/6e-08/3-119/103-141 // sp|Q9Y244|POMP_HUMAN/Proteasome maturation protein OS=Homo sapiens GN=POMP PE=1 SV=1/6e-08/3-119/103-141 // sp|Q9CQT5|POMP_MOUSE/Proteasome maturation protein OS=Mus musculus GN=Pomp PE=2 SV=1/8e-08/3-119/103-141 // sp|Q3SZV5|POMP_BOVIN/Proteasome maturation protein OS=Bos taurus GN=POMP PE=2 SV=1/2e-07/3-119/103-141 // 
TGGACACCTTGACTGGAGCCGATGACCTCATTAAATTTGAAGATATATTAAACAATCCTGCCGACAGTGAAGTGATGGGCCAGCCTCATGCCATGATGGAAAGAAAGTTAGGCCTGTTGTAGTTTGCTTGTTACAGCTTGTCTTCATTTTGTTTCTCTTACATGGCAGTGTTTGACAATAAAACAATTTAGTAAACTATTAATTCACAGCAACTGCATCAATATTTATTGATCAACGTGTTAAATAAAAAT
>Bg-c21485 len=296 count=4 IPR:/ blastx_SP:/
CTCATTCCAAAACTGTTATATCCAACCTGATTTACTTAGGCACCATTCACCCAATGATGAACCCTAGAGGCAGCACTAACTGTAATATCTTGAGTGTTGAGCCAGCTGTAGTCTGGAATTTGTTTTTTTATTATCAATGGCCTCGTTTGGGAGTCTGTTAAATTTATGAGGAAGGGATGGAGGTCACTTTCTAAGTTTAATTGAATCAAATGTTAACAACATTAGCATTTTATTGTCTGCTCAATTTGCATGTACCATTTACATCAAGAGTACACAATGTATGAAGTGATGAATGT
>Bg-c1946 len=996 count=69 IPR:IPR000073:Alpha/beta hydrolase fold-1 blastx_SP:sp|Q8BM14|LIPK_MOUSE/Lipase member K OS=Mus musculus GN=Lipk PE=2 SV=1/1e-78/2-892/101-398 // sp|Q5VXJ0|LIPK_HUMAN/Lipase member K OS=Homo sapiens GN=LIPK PE=2 SV=2/3e-78/2-865/102-390 // sp|P07098|LIPG_HUMAN/Gastric triacylglycerol lipase OS=Homo sapiens GN=LIPF PE=1 SV=1/6e-76/2-874/102-393 // sp|P04634|LIPG_RAT/Gastric triacylglycerol lipase OS=Rattus norvegicus GN=Lipf PE=2 SV=1/1e-74/2-874/101-392 // sp|P80035|LIPG_CANFA/Gastric triacylglycerol lipase OS=Canis familiaris GN=LIPF PE=1 SV=2/3e-74/2-874/102-393 // 
CCTGGCCTACATCCTGGCTGACGCCGGGGCTGATGTCTGGCTAGGTAACAGTCGGGGCACTATCTACTCAACTAACCATACGCACTTGAATCCGAAGAAAAAAGAGTTTTGGCAATTCAGTTGGGATGAGATGGCCAAGTACGACCTCCCGGCCATGATCAACTTTGTTCTTAAGACTTCTGGAGTGGACCAGATTTTTTACGTGGGACACTCTCAAGGAACTACTATTGCCTTTGCGGAGTTTGGAGAAAATCCAGATCTAGCTTCCCATATTAAACATTTCATCGCTATGGCGCCCGTGGCACAAGTCTGGAACACCAAGTCACCTATCAAGGTTTTAGCACCCTTTGCAAAAGATATAGGGGTGTTTCTCCAACTGTTTGGTAGTGGTGACTTTAACGTGAGTCCAGAGATCATGCAGCTGCTGGCTGGGACATTGTGCAACTCTAACAAACTTCTTTGTGAGAATATTTTGTTTCTCCTTGGAGGCTTTGACTACACCTCTATGAATCAGTCGAGGGTTCCTGTCTATGTGGCCCACAATCCAGCAGGGACGTCAGTGAGAGACATGTTACATTGGGGTCAGGCCATAAATTCCAAAAACTTCCAGCATTACGATTATGGCTCAGCTTCAGAAAATATGAAACATTATGGACAGCCAACTCCACCATTATATGATCCAAGAAAGGTCAAAGTTCCTGTAGCTATTTTTAGAGGCGATCAGGATTGGCTGGCGGACCCAGCAGATGTAAAATGGCTGCTTCCTCAGCTGCACGTGACTCACGACGTCAATATTCCACATTATGAGCATTTGGATTTTATCTGGGCATTTGATGCACCGACCTATATTTATAGCACTATTCTTAAAATTGTTTTTTCAAGCTAGTCAAACCATCAAACACTAAATATACTAGAGACAGACATTTAAGATTGTCCTAGACCAAAATGTTCTACTGTTATTGTTACGAAAATAAAAATCTTGTTTTAAAATTAGTT
>Bg-c42025 len=205 count=2 IPR:/ blastx_SP:/
TATCTAAATATCAAAACACGTCGGAATCTGGTTATCTCTGCTAGAGTTCTCGCAGACCCTTGCATAAGTACGGGCCAGGAGGGGTCACCGTAGTCCACTTAGGCCAGCCTATACTTTTGGTAGGAAGCCTTTTGTTCCACTCGTAACTCTTTAAAAGAGTGGTAGGTGTAGCCCCTCGACGTCCCCGCCGTCGGATGCGTTGTTA
>Bg-c30355 len=550 count=2 IPR:/ blastx_SP:/
GCTTTTTTATGACAGTGAGATCCATTACATTTTATGCCAATCTTTCTGTGTGTCACACTTTAATACAAAATATTGGTATGGATCAAATAACATTTTTCGCTGCTTTGAAGCATCCTATTTTTTTTCCCTCAATGGAAATTCAAAATGTGAAGCAATTTGTATGTGACATTTCTGTATCAATGCTTTACTTCATCCTTTGATCTGTTTCCTTCATCTCATCACTATTTCATGTAAATATACATTTGTGTCAAGTTAGTTCAAAGGTTTCATTGTATGCAGTTAGGGAAATAAAATAAAGTTCATCTGAGACCTTCATATAGTTTCAAAACATTTTTTAAATTCTGTTTAGCAACCAAAAGAATAGACTTCATAGCAGTTTAAAAAAAATGAATTATAACTTGATGTATGTGCCTCACTAAGCTACAAAAGAAGTTTTTGATTAAAAAAAAGACAATAATGTCTTTAAAACTGGTGAAAGGGATTTTGTATTCAAATGTTTTTATTCATGCCCCCCCCACTGTTTTCCAGTCATCCGACAGCAGATTATTTT
>Bg-c43007 len=118 count=2 IPR:/ blastx_SP:/
AATAAGTACCTTACTTTAGTCGGATAAGGTAACGGGGGTTGGGTCATTGTGCTGGCCACATGAAACCCTCGTTAACCATCAGCCATTTCAAGTTTTTTTGTTGGTTTTTATTTTTATT
>Bg-c33782 len=317 count=2 IPR:/ blastx_SP:/
CTAATCCTCGAAAAGAGCCAGGCATTGCCGAAGTTGTGAAACATTGTCACAAAAACAACAAATTTAACACACACTTTTAGCTATAATTTCAATCTATGTTAATTGAGGCCAATAATTAACAGTTGGAGCTCACTGTTTGGCCATGATTTCTTGGAGTGCACTTTTAACATACAAAAAATGATTTTGGGGTTAGTAATGCCCAGGTCTGATGTACAACTGACCAAACAGAAGACAAACTACGCTAAAGGTTTTGGAATTTTTTTAAATTTTTTTTTTTTTAATTTTTTAAAAACTTTTTAAAAAAATTTTAAATCGTA
>Bg-c6880 len=273 count=17 IPR:/ blastx_SP:/
AGCAATACAAAGAGCATCGTCCTCCAGTTGGAGGCCGCTTGGTGCTGCCTCCTATTGAGAACGTTACGCCGCACTATGTTGTTGAATCAGAATTCAATCCACAAGCTTCTCCCGCCCATCGTCCTGTGATACAAACTCTCCCGAAACCTACAGGAAGTGTTGTCCCGCCGCTATTCAGCAACAGTGACTACGGCAGAGTTCTCACAGTCACTCAGGACTTTAATGCAACATCTGGAGAGCAAATTACTGTAAATCGAGGCAATAAGGTAGTTT
>Bg-c27085 len=274 count=3 IPR:/ blastx_SP:/
CTAGTCTCAAATTGATGAGAACCGCCGGATTTGGGCCCCCCACAGATCTATAATATGAGAGAGTACCGCATGAATGATTTGCAGCGGGGAAGTTTAAACCACGTGGCCCTGAGGAAGAGAAAGACAACATAAACCATCGGGGTATACGAAAAATGAGGGCAGAGTGCTAGACCATTTAGCTGCCGAGCCACTGCTTGAATTCAAAGTAAAGAAACCTTATGTACACACTGCACTGTGTGACTACTGCGTGTACAAAGACATTTTTCTTTAAAAA
>Bg-c12265 len=455 count=8 IPR:/ blastx_SP:/
ATTATCTTGTAGAAGTGAATTACAGAAGTACACACAGAAATAGAACTGAACAAAACACAAATAAAACTTAACGAAAGCACACAGAAATAGAACTGAACAAAACACAACAGAATTGAACACAACTACACTTGAACTGTTAGGTAAAGAGCTGGTGTACAGAGAGCTGGATAGAAATGATCATCTCTTAGATTCCAATAAACACGATGCTGGTAATATTTGATAAAGAAGAACAGATTTATAATGCAAATAGGGTTGCTCTGTCACTCGAATCTGCATCTTGCATGGGTCTTAGGAACCCTTCAGGTCAATATTAGAGTTCATGGTAGACAACTCTGATATCAAAGAATATATCAAATAAATCAATCTTCAAATCTTGAAATACATATTTCCAAAACCAATATATATCTGATGTCATTTCATCATTACTATATAAGATCAGCTATGAATAAAAAGGG
>Bg-c39139 len=252 count=2 IPR:/ blastx_SP:/
CCTAGACAAATTTGTTTTATTTGAAATAGAGTTCTACAAGTGAGGCCACAGAAGACTAAAGGTCAGCAATGTGGTAAGTGTTAACTAGTGTTTGTAGTAAGCACAATGGTCACACAGTTTACTCTTTTATTGCCATCTGTAATTCTATATTATAAAATAATACTTGACTTAAGCATTGAACACAAGTCCTCTTTGAAATGTACAAAAAGACATTCAACAATTTGCTTCAAAAATAATCTAAAAGAAATTATT
>Bg-c13145 len=666 count=7 IPR:/ blastx_SP:/
AATATTTCATAATGAACCTTTGGGGGGGTGGCATATTTTATTGCAAAACTGAGTGCCTTTAGTTGAAGACATGTGAGCCCAATAGACCACGATTAAGAATGAGTACAACAGTGACAATGATTGAATGATTGATTACTTTACAAAGTGTGACATCAGTGACAAATGAGCTTACAGACAGATACAGAGAGGCTCCATACAATGACATTGAAATTGCATTCAACTTCTCAATAGTTGTACCACAAAAAACTGAGCAAATTTTGAACATTGTTTTTTTTCAGTTGGCCTGGAAGAAAGAGGAGAAAAAAACAACAACTTCTGAAAGAACACACAAAGGTATGTCCACTTTTGTAGGCAAAATAAGAAAAACAAATGTGTTACTTGACTTGGTACAAGATTATTGAACAAAAAAAAAAAGATACAATGATCTTTTGGTAGAAACTTAACTTTGAACTGGTAAATAAACAACTACTTAAATGATAGAAACAAACTATCAACAGAATTTCCTAAGTTCAACAATGGCATGAGTCTGAATAAAGAAACTAACAAAAAAGCAAAACAAGATAACAAATTGAATAATTGTAAGGGAATGATGGGTACACAATAGAAACAAATAATGTACATGTTTAAATTAATGTCTTAATTAATTGTAAGGTCTTCAATTTTCAA
>Bg-c11122 len=507 count=9 IPR:/ blastx_SP:/
TGTTGTGCCAAAGGGTTTTCTAAAAGGAACACTCACTAATGAAGGTAAAACATTAAAGACAATTTATATTGTTATGGTAAAATTTAATTTTATGTTTTATATATTTATAAAATAAGTATAAAAACACTAACTTGGTAATTAAAGTATAAATGATTGAATAGGCCGAAATTCAAGAAACCTTTGAATGAATTTACATTTGTTACAATTACATTCAAATAGACGTTATTATTGTTAGGTTTATTTACAGCAGAATTGAACTAAAAAAATATTGAATTCCCAATCAGATTAATTATAAAAATAAAACTATGACAAACCACTAATAAAAATTGGACATATTTTGTACAACAACTGTTCATTGGTTTGTTGACATACATTTTTGAAAAGGTACAATTAAATGTTTAAAAAAATAGACAAACATAAAAAAAAAGTCTAACTCATAGCTAAAAAAAATAATTAAAAAACTAAGAATTAAAAAAACAAACTCAGTAGTTTAGATAAGTGTGAAAT
>Bg-c41543 len=224 count=2 IPR:/ blastx_SP:/
GGTGGAAGTTGTGTAACCAGTGCTGTTTTGAATGTGAGCTTACCTTTCTACAACACATGAGTGTATGCGTGTTTTCAAAGACTACAACACCATGCGTGTGTATTGGGGCTTGTCCAACTATTCTTCAAAACTGGCAGCTTTATGCAGTGTAGTGGCCTCCCTCTTTCACAAAGTAGTGAGTGTTGTTAGCTGCCTGTCCTCACAGGACTGTACTACTGCTAGAC
>Bg-c26926 len=278 count=3 IPR:/ blastx_SP:/
AGATGAAACAGGAAACACTTTTTCGTAAAGACGAGAAGCAAAAAATCACTTCCAATAGTTCTAAGAGATACGTGCCACAGCCCTGGTGGGATACCACTATTGACCGGGCAACACCTACCTATAAATATTCTCGGGATACAGACAAGTGGACTTTAGTGAGATCAACACCTTTACTCTTTGAGTCCACAACTTATAGCACAATGCATACAACTAGCCTTGGTATTGACACTCACAGGAGGAAGAAGACAAAAAAACGTTGTATGATAATGTAAAAATAG
>Bg-c29003 len=192 count=3 IPR:/ blastx_SP:/
ACTTACGACTATCCCTCCAAAGAAAATTGGGAGACTGCTACTCAGCATTATTACAAAAATCCAGAAAATATCCCAGATTACGTCACTCCTGAAAACCACACTTCCCACAATTGAACCAGTCTATAACAGTGATGCCCAAAATACGGCCAGCGGGCCGGACGTGATTCCATCCGACCCACCGAAACATCGGCA
>Bg-c38760 len=255 count=2 IPR:/ blastx_SP:/
CATTCAGTCACTTGACATTTTTACTACGCCATCTTTAGGCACCATGTTCACCAAGCTGGTCATCATAGCTCTTGCTGGGCTTACAGCAGTTCTGGGATTCTCGACATTCAGATCCCGTATTCCTAATGGTGATCGCGTGCCCAGCCCATCGCCCTGCAGGAGGTAGTTTGGGGGTGGCGGTTGGGCATTTCAATTCTTACTGGAGGTGGACCCTTAAAACCCAATTTGGTGAAGTATTTCGAAGCAGTCTTTGTT
>Bg-c1178 len=537 count=111 IPR:IPR000793:ATPase, F1/V1/A1 complex, alpha/beta subunit, C-terminal blastx_SP:sp|Q27331|VATA2_DROME/V-type proton ATPase catalytic subunit A isoform 2 OS=Drosophila melanogaster GN=Vha68-2 PE=1 SV=2/8e-54/3-383/487-613 // sp|P50516|VATA_MOUSE/V-type proton ATPase catalytic subunit A OS=Mus musculus GN=Atp6v1a PE=1 SV=2/2e-53/3-383/490-616 // sp|P38606|VATA_HUMAN/V-type proton ATPase catalytic subunit A OS=Homo sapiens GN=ATP6V1A PE=1 SV=2/3e-53/3-383/490-616 // sp|P31404|VATA_BOVIN/V-type proton ATPase catalytic subunit A OS=Bos taurus GN=ATP6V1A PE=2 SV=2/5e-53/3-383/490-616 // sp|Q5R5H2|VATA_PONAB/V-type proton ATPase catalytic subunit A OS=Pongo abelii GN=ATP6V1A PE=2 SV=1/7e-53/3-383/490-616 // 
TGTCTGAAATTGTACAGCTTGTCGGCAAGGGTTCACTTGCAGAGTCTGATAAAATCACACTAGAGGTAGCTAAGCTGATCAAGGATGATTACCTACAACAAAATGGTTACACACCTTACGACAGGTTCTGTCCATTCTACAAGACTGTGGGCATGATGAAAAACATTATCTCTTTCTATGACATGGCCCGCCATGCAGTCGAGACCACTGCCCAGAGTGACAACAAGGTGACATTTGCCATCATTAAGGAACAGATGGGAGGTATTATGTACAAGCTAAGCAACATGAAATTCAAGGATCCTTACAAAGATGGTGAAGCCAAGATTAAACAAGAGTTTGAGGAACTACATGAAGAGATGCAGCAAGCATTTAGCAACCTAGAGCTGTAGACACATGTAGGCAGACTTAGACCAGTGTGTAGGCAATGTTGTGACTCATTTTAGTATGTTAGTAGTCCACTTCTTTCTTCCCGTGCACTCAGACTAGGTACAACAAATGTTTTTTATTCTTTCTATGAAGGCTGGTGATGTTCAAATT
>Bg-c8824 len=609 count=12 IPR:/ blastx_SP:/
AATATCTTAGCCAAAAGAACTGTGAAATACTATTAAAAATGTAATGTAGAGGAATATACAGCAGGTACTTAAGGGTAAAAATTATATGATACGGAAAAGTTATTTTAAATCATTTAATGATTTTAGAGCCACACCAAGAGCGCCATGCCATTTGTAACCAGCTTATTCATTGCACCATTCAGTCTTGTCACAATGCCTTTCATCAGTACCTCAGGCTGTATGTATTCTTCTAGTGGATGTTTTAGAACTTGATATAAATATATATACTTATATAATATATTCTATTTTGTACAGTAGATGGGCTTTACCTCACATATACAAGTCTTTGTTGATCATCATTTATGCAAAACAATAATAAATGTCAAAGAAAAAAACTCAGAAAATGTTTGTGATAAATTGTGTTTCCCTCCCAGAATGTTTCTTGTTCCAAGACTGAATAGTTGTTTGGTTCTATTTCTTTGATAAACTTTAGATTCTGTGAAAGCTTTAAAACAAACATTTTTTTTTAGTCTTGAATTATGACATTTTATGCAAAAAAAAATTTTTTACTACAAGTTAATTGTTATTGTGAGGTTTGATCACTCTCACTAGAGAGTGTACTTTAATTAA
>Bg-c2899 len=521 count=45 IPR:/ blastx_SP:/
CAAGCATGATGCTCTTTTTTTTATTGATCTGAATATAAAGTCAAAGAAATTATAGATGCAAATACTGGAAGACATTATGCAAAGCCAGATGCATTACAATTATTTAGCAACTAAAATTCATATTAATAAAAACAAAATATTCTTTAAAGTGTTGTAAAAGTCTTAAGTGATAAAAAACAGGATATTAAGGTCATTCTTAATGGTAATGGAAATCAATATAATCATGTATATAAAATAACCGAGATGATGACAAATAAAACCAATCACTTCCTAAAGCAAATCCAATTTGAGTTTTTGTTAAGTTCTAAGAAAATCTTATAATTCTTGTCCTTTTTCAGTCCTTGTTTAACTTTAATTGAATGTTTCTAAACCAATAGAGGACCTTTTTATTTGGGTCTAAACTATTTTTAAAAGCACTCAAAGCTTATTTTGAAATATTTGTGATTTTCAAATTCAACATTTTCACCAAGTGATTGATTGTCAAAGAGTATCACACATATTTAAACAAGAACTTTTATAAA
>Bg-c19773 len=503 count=4 IPR:/ blastx_SP:/
TTTTTAAAAGGTTTCTAGCAAAAATGATTTTAAAATATTTCAAGACTTAGTGTGGAAATCTTTTTAAAAATAATGTATATAATTTGTCCTCAAAACTATATCTAGTGTTTGACATTGATTTGAAAATTTTGATCCACATACATCACTTTTTTTTTTTTTTTTCAAATTTTTATTTCAATTTGTCACTCTGAAATATTATTGACTTGAGAAAAATTTGTTAGCGTAACATTCTTGTGTCTCATTTTTTTTATTTTTAAATTAGAGATACAATGTCAGTGAACTTTGTGATGTCTTTTTTCTTGATGTTTAGTGACAGGTTTAGTTGATCACTCTTTAGTTTTCTTGGGAGGGGCATTTTCAAGTGGTTTGGGTTTTTTTTTTTTTTGTTTAACAAGTTGGGGATGTACTTGCAGTGGGGAACGCCTGCAAATCCTTCTGTTGGCCGTCTATGTATTTGTACAAACTGCATGTCATTGTTATGTGTTTTGTCAATAAAATTATTT
>Bg-c32664 len=354 count=2 IPR:/ blastx_SP:/
TAATTTATTCCCATTTACCCTTTATGAACAATATACGACCGTGCAGCCCATCCCCAATCAACGTTACGCCTCTGTAATCTTGTAATTAGATTTAGTCCTAGTCCTCAGCTTGCATCGAAGATTCACAAAATATTGAATTAGAGGATTTCACTTATTTTCAATATCAATTTTGGTTGATTAAATATTTAAATTTCTTTTGTAAAAAAAAAAAAGTAAACATTTTTACTTGATTTGTTTTTTTTTATTTTTAATTAACTACTAAGTTTCGGTTTTACCGTTTTTTTTAACTTTTTTAATTTAGTTAAGTAAATAATTAGTTTTAATTTTAGTTCGTACTAAAATAAAGTAAGTAAG
>Bg-c2360 len=943 count=56 IPR:/ blastx_SP:/
AGTTTGATGCCATGGTTACCTCACTTTCAATTATTTCTAGATCCAAATTTTCTGTTTGGGCGCCACTTCCTGTCCAACAGGGACAAGGTATTCATAAGGTACATTCTATTGCTTGATCTGAATTATAGATGAAAAGTTATTATGTGAAAGAACAAACACTATTTTACGGAATTCAAACCTTAAGCATAAGAAATTAGAGTTTTTATTATTTTAACGGTCAGTGTCTATAACATTTGTGATGTGAGCAAGATGTTTCTGTTGTTATACATCTTCTTTCTGAGGAACTCCATATGGACGACGGCGCTTTGCCCAGTACACTATTTCATGACGGAAGTGGTCAAGGGGACTTGCGAATCGTACCAGAAGGGCATCTGGCAAATTCCATGCGACAGTGGAAAGCAAAAAGTGAACATAAATCACAGTGACTATTGCTGCGTCTCTTGCTCGCCATGTGGCGTCTGCGGTCTTGGAGTTTTCTTGTACCATGACTTTGAGGGCCGCGCCTGCGGCTGGAACTCTAACGTCATCTGCTGCGAGGAGGAGAACATGGACGTCTCGAATGACCACTGCGTCCACAGGACCACTCCGTCACCCGACATCATATTGACCTCAACGCTTTCGTTGGCGCTGGAGGCAAAGACGACAAAGAAGCTGATGTTGCGAAATCGTCAAGAAGCTGAACAATTCAACTTGAATACATCTAACAAGTATAGAGAATCGGTTTTCTGTTTCAGTGTCGTTATTGTTATCAATCATTTTGTTTGCACGTGATTAAAAGACACTTTACCCACTTTGCCATCATTCGATTTAAACTGTTATATCGTCTTGTGAACGGCATGTCATTGACTGCAATCCCTTATCATTAGCTTGCACTCTCTACACGGATGGGTGAAGGTAGGAATGAAACAGTTTATTTTTAATTCGAACATGGCTCTAACAGTTT
>Bg-c952 len=678 count=131 IPR:/ blastx_SP:sp|P83553|DERM_BIOGL/Dermatopontin OS=Biomphalaria glabrata PE=1 SV=1/1e-39/583-143/1-147 // sp|P83553|DERM_BIOGL/Dermatopontin OS=Biomphalaria glabrata PE=1 SV=1/3e-10/424-137/2-95 // sp|P83553|DERM_BIOGL/Dermatopontin OS=Biomphalaria glabrata PE=1 SV=1/3e-10/589-305/51-147 // sp|Q01528|HAAF_LIMPO/Hemagglutinin/amebocyte aggregation factor OS=Limulus polyphemus PE=1 SV=1/4e-32/589-143/17-167 // sp|A8QZJ5|MCTX1_MILDI/Millepora cytotoxin-1 OS=Millepora dichotoma PE=1 SV=1/1e-31/592-143/73-222 // 
CTTTATTTACCCGAATGATTTTCAGATACATATATTTATTTTCCCATAATTATAATTTGACTTCGTTTACAATTCAATCTTCAATAACTTAAAGCCATAGATCGACATCTATCATTGTAATTTGTGGACGAAATCAAACAGTGCACAGAGTGAATCTCCAGCGTCTGTCCTCGTAATAGTTGTTGTGCTCACTCATGGCCCCTTTGATTCCTTGACCTTCTGGCACGAAGAGGGTCAGCTTGCCGTCCCAGTCGTTGACGTAATCGGTGGTGTAGCAGTCGGATGGCACCCTCTTGGAGACTGTGCAGCACTGAAACCTGTAGCGTCTGTCCTCGTAGTGGTTGTCATGGTAGCTCTCAATTCCGGTGATGACGCTGTCCCCTGGACAGGTGAAGGTCACGGGGTTGTCAAACGTGTTGACGTAATCACTTTTGACACAATCTTTGGTGTATCCCACTGTGCGACAGAAGAGTTCCCAGCGCCTGTCTTCATAATAGTTGTCGTTGATACTTGACACAAAAGATAAAATCTGTCCGGTCGGACAGTTGAAATTGAAAGGCTTGTCCCAGTCGTTAACGTAGGCAGCGGACTTAACTTGAGTCACCACCAGCAAGAGTCCCAGGCTGACTAAGATGACCATTCTGAGCAGTTGGAAATCTTTGAAATGTTCAAAGTCTG
>Bg-c16471 len=731 count=5 IPR:/ blastx_SP:/
GTAATACATGTTCCTTAAGTAAGTAACTTGCATAGACATTGGTTGTGTTGTTTGCTGTCAGTACCTGGTATGTATTGTGTGTGTTTAAATACTGTCACCTGAAATTCGGCAGACAGATTATACATTTAAAAAACAAACAAACAACTCACTTGTGATGTGTTGGTCAAAGTGTAAAGAAGGGATGTCAGTGCTCAAAGTGTATAGATGTAAATACACATTTTGATTTTTATTCAATTAATGTATAGCAACTCATTATTGGACATTTGCTATTCTAGACATTTGTAATGTGACATCCATTGTATGCATTGGTTTGTAGAAAGTTTTGATTTGATGCTCGACTATTTCATGAGCATATTGTTTCTCCTAGAGAAACTAGTCAACAGACTTTCATATTGAAGGAAATACAATAATGAATTAGCATTAAGTACTGAGTTAAAGGATGTGATGTTTACAGCTGTTGTTATCAATTCAGTCTTTCAATTTGTTCTTTATTGCTTCAGTATTTGCTAGTATTAGTTGTGTTCAAACATCATTTAATGGCCACCTCTATTTACTGTATAAAGTATTATAAGAAGATCTGCTTGCTATTTACATTAGTTAGTTCTTGTACTTCCTGGCTGAAGGTTATAGTGATGTTCAAGTCTGTCACAATGTAGACATTATTACATATTTACATTTTTACTATCTCCCCATAAGGTCTGGAAATTTTACTTGAAATAAATCTCTATATT
>Bg-c17773 len=353 count=5 IPR:/ blastx_SP:/
CTCTGATTCTAATGTCTCTAGTTGCTGTGATGTTTTAGATTTCAATTTGTCTAGTGTTGCATTTAATTCTACTATTTGCTCCTGCTGTTGGGCAAGTTTGCCCTGATAGTTTTGAGCTGCCTCCTGAAGTTGCCTCTCCACCTCCCTCTGCAGCGCTGCATAGCTCTCTTCTACTTTAGCTAGACTTTGAGAAGAGCTCAACAAGTCGTTTTGTAGATCCCCGATTTTTCTAGAGGAAACAAAGAAACAACAACATTCATTTAAGCTCGGCAGTATTATTTCTGTTGAGAATAGGACCACCTTCAGACATTTGTTTTTCTTGCAGCCAAGCCTTTAAATACTAATTACCTTTT
>Bg-c37002 len=270 count=2 IPR:/ blastx_SP:/
ATTGTGTAATCANCGGGCTGCCCCCAACTAACTTTCAGTGCCTGTAGCAGTGCTCCCTGTATAGCTGGCTTGCTGCTGCACCCCATAGGCTTGAGCCCATGCATTCTGGTCATAACTAGGTTGCTGGGCACTATATATGGCTGGCTGTGATGGATAAGTTACTGGCTGCGTCGCTGGGTTAGTGTAACTAGTCTGTGCTTGTTGGCTGACTAGCAATAACTCCGTCGCGTTGGGGTTAGTCACTAGTACTCTTGTTGGTACCATAGCTGT
>Bg-c10511 len=345 count=10 IPR:/ blastx_SP:/
TTACTTCACTGGCATTATTTAATTCTAACAGAAGTAATAGGATACAACATTTCAGGCACATTTAAAAATGACTTAAAAAGACAATGTTAACAAGGTTTAGTTTTCGTCTATGTCAATGCTTATGTGAATATCATCGCCAAATACCTGCTGATCAATCAAGACGTCAAGAGTACAGTCAATCATCTCAGAAAAGTTCAGTCAATCATCTCAGAAAAGTTCAGTCAATCATCTCAGAAAAGTTCAGTCAATGAGTCTACTTCAACGACTGGCTCCTTTGGTTTGACACGTTTGATTTTAGTCAATCGTCTGACCACCCACCTGTATTTGATGATGATCTCATCTTTC
>Bg-c8263 len=651 count=13 IPR:/ blastx_SP:/
AATTGGGAGCTTGTGTCAGTCTGCTTGTGTTGGACAGGAACAGAGTCCTCAGTTCATACAGTTCAGCTACATATGTGTCCAGTACGGCTCTCAGGGCCAGCCGTTGGTTGATCTCCTCTTTTAAGTCCCTTTTAATCGCAGCCATTTCTCGAATACAGGAGCAGTTTGAGATCGTGGTCGACCAAATGCTTCGGATGTTATCTTCTTCCTTCTTGGGCACGTTGTCCTCCGAGACTCTCATCTTCTCCGATGTCTCCTGGAGCATACCGCGCGGTGTCAAGTTTTGACCTTGAGCACCCCAACTCAAGGTGAGTAACAAAAGGTATACTGCGTGGCCTAAATTAGACATTTTTCTTATTCCGATTTTTAAAAAAATTGAAATAAAACAGCGAACTCCTGATTTCTTTAAAGGAATGACACTATCTCGTCAAACACTTATTAACGAACATTCAATGTTTTTGTTATGTTTTATATATAAAAGTTTTGATCTATATACAAAGTTTTTCAGATAACAAGGATGTATTTTTTTCCCAGAGCTCTCCAAAAAAGAAATCTAAGCAAGAAACTTTGTCTCACGCTTCAGAGTGTCACAGCATTGTGACTGCGGAGTCCGTGTCATCTTACGGCTCCAACGTGGAGGGGTTTCATTTT
>Bg-c3785 len=825 count=33 IPR:IPR000253:Forkhead-associated blastx_SP:sp|Q8TAD8|SNIP1_HUMAN/Smad nuclear-interacting protein 1 OS=Homo sapiens GN=SNIP1 PE=1 SV=1/4e-70/633-115/215-387 // sp|Q5M9G6|SNIP1_RAT/Smad nuclear interacting protein 1 OS=Rattus norvegicus GN=Snip1 PE=2 SV=1/3e-69/615-115/212-378 // sp|Q8BIZ6|SNIP1_MOUSE/Smad nuclear-interacting protein 1 OS=Mus musculus GN=Snip1 PE=1 SV=1/7e-69/615-115/208-374 // sp|Q8W4D8|DDL_ARATH/FHA domain-containing protein DDL OS=Arabidopsis thaliana GN=DDL PE=1 SV=1/1e-51/645-157/150-311 // sp|Q54VU4|Y8013_DICDI/Probable serine/threonine-protein kinase DDB_G0280133 OS=Dictyostelium discoideum GN=DDB_G0280133 PE=3 SV=1/3e-39/678-115/1298-1501 // 
TCGATTCAGTTATTTTATTTTATTAATTATTAACATACTATCAATATACAAATATCAAAACATATAAATTTGTAATGCATTTAATGATCAACCATTATCTCACCCTAGTTATACTTCTTCCATGTCATCACCTGCCGTGACCTCTGAAAGATCAACACTGTCGTGAAGTACCACATACTCTCTAGTGCTAAAACCAAATTTAACAACGTCTTTTTCTCTGAGTTCGTAGTAGCGCTTGGGCTCTATCTTTTCATTGTTCAAGTATGTTCCGTTGGCAGACTCGAGATCTATGATGTAAGGCCTGACTCGTCTGCCTGGTGTCCCATCAGGACGTGTGTAGGGCATGGCTCGAAACTGAAGAACTGCCTGCTGTTTCGAGCAGGAAGGGTGGTCGATAGGAATGTCTGCTATTTTTCTATCACGGCCCAACAGATAGGCACTCTGTCTGTGAATATGTAAAACAGGTAAGGCTGTATCACCTTTGAAAGGATACAATCGCCACCGGCGTTTTGGTTTCATTGCTTCAGGTGGCTCATTATATTTTATAACGACTCCTCGAAATGTGTTTGTATCTTCCGTCAACTTACCAGAAAGTTGAAAGTTTGGTTTTTGTTTATCGTCTTTCTTTTCCTCGGGTGCGTCTTCCGATTTGATCTGAATTGTATTCAAACCAAAGTCGCCGCCAGCCACATCTCTTTCTCTCCTTTGCTCTCGCCTCCGTTCGTTGTTTCGGGACCTTTCCCTGTGCTGGTGCCTGAAATTTTCATCATCAATCTTTTCTTGTTTTATCCGTATGTTAGGGTCATACATTTCACTTCTCTTTTT
>Bg-c6942 len=844 count=16 IPR:/ blastx_SP:sp|Q6A037|N4BP1_MOUSE/NEDD4-binding protein 1 OS=Mus musculus GN=N4bp1 PE=1 SV=2/1e-10/13-354/744-893 // sp|Q80U38|KHNYN_MOUSE/Protein KHNYN OS=Mus musculus GN=Khnyn PE=2 SV=2/1e-06/13-318/559-658 // sp|O15037|KHNYN_HUMAN/Protein KHNYN OS=Homo sapiens GN=KHNYN PE=1 SV=2/2e-05/13-318/566-665 // 
CGTCCTTCTTCATATGTCTTTGTTGGAGATGCATTTATGCCTCCTGCGGTTCCACTGGGTCGCAATGGTCCAAAACTTGATGTGTTTCTGAAAAACCCTTCTTACAACAAACCTAATGTATCATTCATGCCCTTCAAAGAAGTAAACAGAGTTGACAAGCCTTGGCAGAAACCTATCATCACAGCTAGTGCTAATCCTAATGACACCAGAAGAAGTCGGGAAGAAACACAACGGTTGTATGAACAGTTGATTCAGGTATTTCCCAACAAAGACCAGTCCCTGCGTGTCTACCAAGTTCTAGAGAACCACTGTACGGAAACAGACCTGGTCAAACTGACCAATTATGTCATGAATGCTCTTTTCTCTAAAGACCATTAACAGGCAGACGGCGCTGTCCATTACCCACCCAACACAAAAAGATGAATATATTGTTCCACTAACTGTCTGAGCTATGTAGCTCATTATGTTTGAATTGGCCAATAATAACATAAAAATCACTGTATGCCTTGCTCAAATGTGATTGTATTTTTATTTGTATCATTGGCTGAAGGATTTAGATTTCTCTGAATATCTGGAGTAACCTGTACAGGAGTATGTTTATTTGGTAATAAATTGAGACAACTTTTTTTGTATATGAGTGCAATAATAAAATACTTTCTTGTGTTGATGTGTTTCTGCGTCTAGTTACAGGGCTCTGCTTATGTATTGGAAGACTTTGTGAACTATGGAGTTTCAATGAAAAACTCATGCAATGAAATTGAAGTATATAGACTGAAGCTAACTATTCCTCCCACTCAACATTCCTTATGCTTTAATATAAACTTTTTTCAAACTAAAATGTGAT
>Bg-c37918 len=262 count=2 IPR:/ blastx_SP:/
GCAATAAATTACTCAGAGAAGCAGCTGACATAAATATTTTGTCAGTTTAATGTATGTCATGCATTTCACAAACAAAATTGGCTTAATATTAAAATTTGTTGAAAACAAATAAAAAGAAGCTAGATCTCCCTCTAACTATTTGTTGATGTTGCTGCTATACAAGCAGCCCATAGGACTCTCAAACATAATGTCTTTCTTCTGTGCTAATTGTTAGATAGGAGAAAGCAAAAAGGAAAGTAAAAAAAAATTAAACATCCCATTT
>Bg-c2902 len=461 count=45 IPR:IPR007512:Protein of unknown function DUF543 blastx_SP:sp|Q9N4K0|U327_CAEEL/UPF0327 protein F54A3.5 OS=Caenorhabditis elegans GN=F54A3.5 PE=2 SV=2/3e-09/43-237/9-73 // sp|Q7TNS2|CA151_MOUSE/UPF0327 protein C1orf151 homolog OS=Mus musculus PE=2 SV=1/1e-07/55-240/2-63 // sp|Q5TGZ0|CA151_HUMAN/UPF0327 protein C1orf151 OS=Homo sapiens GN=C1orf151 PE=1 SV=1/1e-07/55-240/2-63 // sp|Q7RYI0|U327_NEUCR/UPF0327 protein NCU06495 OS=Neurospora crassa GN=NCU06495 PE=3 SV=1/3e-06/34-240/15-83 // 
TGGTAACAACGCATCCGACGCGGGGGCATACAAACGAAAATGGCGAGTAAAAGGTCAGAGGATGTTTATGGACAAAAGGTCGATCGTTGTATGTATAATGCTCTAATTAAACTTGTTAGCGGTGTTGGTATCGGCATTGTTGTCTCTGCATTTCTTTTCAAACGCAAGCCTTGGCCAGTCATTCTTGGAGCTGGCTTAGGAACAGGAATGGGAATCTCTGACTGTAACCATGAGTTCAAAGGTCATGTCCCAGTGAAGCCAGTGCCAGTAGAAGTAACATCTGAGACATCATGATTGACAACTGAGTGAATTTATTAACATTGATTGTGTCATGCTGTACTATTTTGAACTGTCTTCAATGTGTATTGATTGAGAACATTGCAAGATGTAATGTCTGTCTGTTGTCTTGGAATAAGACAAAAGTTTAGTAGAATTTCTTTTATTTATTGTCTTTGATAGAA
>Bg-c1894 len=645 count=71 IPR:IPR002557:Chitin binding protein, peritrophin-A blastx_SP:sp|Q11174|CHIT_CAEEL/Probable endochitinase OS=Caenorhabditis elegans GN=cht-1 PE=1 SV=1/7e-06/555-193/485-616 // sp|A8XWX5|CPG2_CAEBR/Chondroitin proteoglycan 2 OS=Caenorhabditis briggsae GN=cpg-2 PE=3 SV=2/4e-04/555-199/224-330 // sp|O76217|PE1_ANOGA/Peritrophin-1 OS=Anopheles gambiae GN=Aper1 PE=2 SV=2/7e-04/537-190/36-153 // 
CACAATTCATTTTACAAATTTACACAAATGGATAACAAAAAAACTTTCAGCAAGAGAAAACAGACGTTTATTTTGTCATATTAAGAATTTTTAAAATTATTATTTTAGCTTTTAACCTGGAAACTCTGTCCACTTCAGATCTTGTGAAACACTTTAACCAGTGTGGGCTGTGAAAGAGAAGTGGTAGTTGTCCGCGCAGTTGATTCTGTTGGCTTCCCCACACAACTTGGCCTTGAAATCGAATTCTAGGCCATCCGGACACGCCAAGTGGTATGTGTTACCGAACACGCACTCCACATAGTGAGAACAGCTACCGGGGTCTGGATAAATGCCGTTTGGAAGTTGGTAGTCGTAGCAAACGCTTCTCATATCCAATGTAACAGGTGCAACAGTGGAAACTTGATCGTTGCATGCAGGGACTTGGGCAGAATTCCGGCATATTCTTATTAACGGGTCAAAGGACTCTCCAGCTGAGCACGTATGGATGGTCGTGACGAAATTGTTACACGTCACGTACTTGTTGCAGTCTGTAGGGTGTGGGTGTGTCCCTTCAGCCCAGTGGTTTTGTCCACATACGTCCTGGCCACACACCACAAGCATGGACTGTGCCAGGACAGCTGTTAAAAGCAAACCCAATTCTAATGA
>Bg-c303 len=666 count=265 IPR:/ blastx_SP:/
GCAGTGGTAACAACGCATCCGACCGAGGGCACCATTTAGTCACTGTCTCTGGACAAAGGAAAAAAAAAGTAGCAGCAGTCTCAATTCAAGGTCGAGCGTTCGATCGTCCCTGGTCCCAAGATGGCGTTCCACCAAGAGTGGGCCTGGTACAACCTGTACTTCCCCAACCCTGACGGCGCCCTGTGTCGACAACACATGACTGAGCAGGAGTGGCAGCGGATGACCAGGTGGAGGGACTTTGACTGGTTCAACCAGGGCGGCCAGGAGTCCAACAAGATACTGACCTACACGCAACAAAAGGCGACGCCGACGCGAAACTCAGACGGCAAGCTGCAGCCTCACCTCCTTCCACCAGCTGGCGTCATCTCCAGCACACACGCACGTAGACATCCGGGCTTTTACATGGAGATGTACCATCGCCTGCTTCACGAACGGGAGTACTTAGTCCGGCAAGGACGTCTGCCACACGACCTTCCTTGTCCATCCTTTCACAATCCGTGCATCTGCAACCACCATGGAATGATGCCCAGTGGTCAGCAGTAGACTACTGAAGATAACCCGAGGTCAATGTTAGCCGCCTGCTACTTCAAACTATAACCCTAGTTCAATATCAACATCTTGATTGAATGCTAATAAAATGTGTATGATACGCTGAAAAAAAGAAAA
>Bg-c644 len=384 count=171 IPR:/ blastx_SP:/
GTAACAACGCATCCGACGCGGGTTGATGGCAGTCATAAACTTAAAACAACAAGCTGAAAAGTCTATGCATTGACATTGCAGCTCTCTAAAAAAATCCAACTGTCCAAAGACGGCATGCTCCGTGAGGATGACAACACTTTCTTCAGAAAGGAAACGCACAGGATAAGACGCGAAAACATGGTGTGGGCTTTGCTAACAAGAACAGTCTACTTCCGATGATAGTCTCTCAAGTTGGTGGCTCGGAAAGGCTATCACGCATACGTATGATGAATAAATCTGGAAAAGTCACTCTACTTAGTACCTTTGCCCCCAAACTATGTTCACTTCAAAAGGACAAAATCAAGGTCTATGAAAACTTCAAGGAAGCTATTGAGATAATTCCTT
>Bg-c655 len=1709 count=168 IPR:IPR013027:FAD-dependent pyridine nucleotide-disulphide oxidoreductase blastx_SP:sp|A7YVH9|PYRD1_BOVIN/Pyridine nucleotide-disulfide oxidoreductase domain-containing protein 1 OS=Bos taurus GN=PYROXD1 PE=2 SV=1/1e-148/131-1582/12-502 // sp|Q6PBT5|PYRD1_DANRE/Pyridine nucleotide-disulfide oxidoreductase domain-containing protein 1 OS=Danio rerio GN=pyroxd1 PE=2 SV=1/3e-147/128-1582/12-490 // sp|Q68FS6|PYRD1_RAT/Pyridine nucleotide-disulfide oxidoreductase domain-containing protein 1 OS=Rattus norvegicus GN=Pyroxd1 PE=2 SV=1/3e-146/134-1582/10-498 // sp|Q5REJ2|PYRD1_PONAB/Pyridine nucleotide-disulfide oxidoreductase domain-containing protein 1 OS=Pongo abelii GN=PYROXD1 PE=2 SV=1/9e-146/131-1582/12-500 // sp|Q8WU10|PYRD1_HUMAN/Pyridine nucleotide-disulfide oxidoreductase domain-containing protein 1 OS=Homo sapiens GN=PYROXD1 PE=1 SV=1/3e-145/131-1582/12-500 // 
GAAGAATCAAAACAAATCTGCGACTGTAATGATTTTCTTAGATCTACAACTTAGAGTAGTTAAAGTAACGAGACATTAACATTAATAACAAAAATAAACTTATAACAGACTGTTGGTGATGCCTGATCTTAAATATGTTGTTGTTGGTGGTGGTATAGCGGGAGTAACCTGTGCAGAAACGCTTTCTCTCCTTGCAGAGAATGCCACTATTACATTACTGTCAGCGTCACCACTCATCAAAACTGTGACTAATCTGATACAGATAACTCCAAACATTGAAAGTTTTAATGTAGAAGAACAGCCGTACAAACATGTGAAGGAAGGTCACTGTAAAGTTCAAGTCATCCATACGACAGTCAACTCTCTCTGCGCTCAAGAAAAGAGACTACATCTATCAGACGGTAAAACTTTGGACTATGATCAATTATGTATCTGTTCTGGGGGCAAGCCAAAGCTCATTGCAGTCGATAATCCGTATGTACTTGGGATACGTGACACGGAAAGTGTCAAGCAATTTCAAAAAAAATTAAAATCAGCCCAAAGGATTGTGATAGTCGGCAATGGTGGAATCGCAACTGAACTCGTGTACGAGCTTGAAGGCTGTGAAGTTATTTGGGCCATCAAAGACAAGTCCATCTCCCACACGTTTGTTGACGCAGGCGCTGCTGAGTTCTTCATGGCTCACCTGAACAAGGCCAAGGACAATTCTCCCGAGGGGCTTTCTAAACGCTTGAAATATACAACCAATGAAGAATGTGAGGTCATGGCGCCTGACAAGAAACCGGTTATGGGTAGTGCCCTAGGTCCAGACTGGGCAGCTCAGTTGGACATCCGAGGTGCTCAGCAGATGTCTCACAGAGTCCATGTTGAACATGAAGTGGAAGTGGAGAGAGTTCTGTCCCCTGATGACCTCACACTGTTGGGGTTAAAAGCTTGCAGTGTGGATCAAGGTCAAGATTTCACCAACTGGCCAGTGTATGTACAACTGACCAATTCCAAAATCTATGGCTGTGACATCATAGTCAGTGCTACAGGTGTGGAGCCATTCACAGAAGTCTTTCTGCCGGGTAACAATATTGACCTTGCTCCAGATGGTGGGATGAAGGTTAATTCCAAGATGGAGACAAGTGAGCCACATGTTTATGCCGCAGGAGATGTGTGCACTGCATGCTGGGACCCCTCACCATATTGGCAGCAAATGAGGCTGTGGTCGCAGGCCAGGCAGATGGGTTGCTATGCAGCTAAATGCATGGTTGCAGCTACCAAGGGAGAGAACATCACTTTAGACTTTTGTTTTGAGTTGTTTGCCCATGTCACAACATTCTTTAATTTTAAAGTTATTCTCCTTGGAAAATTTAATGCCCAGAATCTGTCCAACGACTATGAAGTTTTGTTGAGGGTCACAAAAGGTCAAGAGTATGTCAAGGCCATTTTACACAACAACCGACTTGTGGGAGCCATTCTCATAGGGGAAACTGACCTTGAAGAGACTTTTGAAAATCTAATTCTGAATGGCCTTGACCTGACTGCATTCAAAGACAACTTGCTGGAACCTGGTATAGATGTAGATGACTTTTTTGATTGATGGACTATTTTGACTTGACACCACGGTGATATAATCTTTGCGTATATAAAATAAAGCTTACTGCCAGAAAAGCAATGAAGTGTAACATACAGATAAATAAAGTTAAGAATATATCTTGAATAAA
>Bg-c26956 len=277 count=3 IPR:/ blastx_SP:/
TGTGAGCTGAAATAATGGTCAACGTAACAGAGCGGCCGCCAACTCTTCGCTAGGAAAGACATTTTAATTTACCGGTACTGATGCATACAAGATGACTATATTTTTTTTATACTCAAAAGTTGAGGAGGGGGCCCCACCATCTTAACGCCACTGCTGTAGCAGTATCCTGAATAGAGACTATCTCAAGACCACTTCATGAATGACATTGCAACTCTTTTATCTTAAACACAAGTTTAGAACTACAGGCATCCCAGTGCTAATTTTTTTTAAAAAAAGG
>Bg-c5605 len=638 count=21 IPR:/ blastx_SP:/
AAGTGATCCAAACTGTGACCAATAACTTATTCTCTATCCAAATTCTAGTTATTAATTCTAAAATTAGTTCAAAATGTGTCTAGCTTAGTCATATCAGTTGTAATAACTATGTTTACCCCAAGCCTATTGTATCAAGCAAAAGAGATGTCCAGGAAGTCTTGTTGGCTCTGTTGTACATTTCATTGGACTTGGATTAACAAGATGTCTTTCATTTGATGTATACTTAAACAGTATACTCTCAATGTTCTTGATTGTTCATTTGTTTCTTAATAAAAGGACTAAATTAGGATAATAATGTTTTTGGTCAGTCGTCGGTTGGTAGCACCAAATCGCTAGTACAACTAAACGGATGTAATACAGAACTTTGAAATAGCGTTAACTTCTTTTTCAAAACAAAACTAAACCTTTTATAAACAATATAGACAACTTCAACTTGAGATGAGATAAAGTTCTAATAGTAATGATGGCATTTAAACAAAATCTTACTCACTACAAAACATATGCCCTAATTGTTTCAAACCTCTGTGGTGTCTCATTCTTAACATAGATTGTCACAGAGCATCATTCTACAAGACTAACAATTAATGCTCCCTTTCTTCATCATCACTTAGAAACAAACACACACACACTCCATAGCA
>Bg-c20388 len=405 count=4 IPR:/ blastx_SP:/
CTCTAACTTACTGATTTAATGAAAAAGTATGTTAAATTAATATAAAGATTGATGTAGATAAGTCGTCATGATTTGAATTCTATTGAAAAAGTAGTAAAAAAAAAAAATACTGTTTTACAAGATAAAGGTCATGTGTGTTGTCTGTGAAGGGACATTGTTAGCAACAAAACAATTCTTAGACCTTTATAGAGTTTACTTGATTTCTATTCATTTACTTTCAAGTGTAATTAGAAAAATGTTATATATTTTCACACTGGTGACCAGTATTATATTGCATTTTTTTAAATATTGCATTTTTGGTATCATGTTCTTTATTTATATCAAACTATGAAGATTGTTTTTTCCATTCATGTATAAAGGTAAAACCATATCTGCATATGAGGGTAAATACATAATTTATTAATG
>Bg-c32489 len=361 count=2 IPR:/ blastx_SP:/
GTTTTGTGCTGATCAGAAAATTGAGTGTTTCTGTACTTGTATTACTACTCTGAGCTCTTAGTAACAGACTTACAGATCAAAACATATACAATACAGGTAATCAACTGGCATAGACCAAGTATGACTGTTTAACAATCTAATCTCAGTCTCTATTACATGGTTGTTCGCTAGTCTCAACTGTCTGTTTATCTATATCTAAGCTACAGTCTCGTTTTGAATTAATGTCGTCACATTTGCGCATTCACTAGCAGTCACCTAAGGTGCGTCTCTACATAGGTATAAAACCAGTATTGACCACTCACCCACAACGACTCATAGATAAACCTTGAATCCCTTTCATGGACTAAACTATTTTACAACG
>Bg-c16654 len=617 count=5 IPR:/ blastx_SP:/
AAAATAACATTTATTACAACACAATGTGAGTTAATATAGTGAGAAAGTTAACAAGACGCTTTGATAAAGTTCAACCAGAGTAACATTAACATCACACTAACAAGGTCCTACATCAAGGCTTGTGGGTGATAGAACAGCTTCAACAGTGAAACAAAATCATAGTTTTTTTACTCAGATTTGAAACCTAACTTGTCAATACAAAGAATGATGGTGTTGAAATACAGACTTTCAATACATTATTTACATCTTGTTATTTGACAATCTTGTAATAACATATATGGAATGAAATGTAATAAAACAATCCGAGTTACAACTTTTCAGGGTGAAATGTTATCAAAATACTAACATAAACTTCTTTTATATAATATATGAATCCATAAAATGAATTTATACTTGCATTATAATGACAAAGTCTTTTAAAAAAAAAAAAATTAAATATCTTGCAAACACATACGATTCAACTTTTTATATGTCACCAAATGAAATGGACACAAAATGGCTTTAAAATCAAACACATCTGTCCATCACAATTTATAATGACAAAGTCTTTAAAAAAAAAAAAAATGTTTGTCTTGCAAACACATATGATTCAACTTTTTATATATCACCAAATGAAA
>Bg-c2998 len=1099 count=43 IPR:/ blastx_SP:/
CAAATAAAATATCTTCAGTTTCAAATGTTGAAACGTCATAACAGCTAGCCAACGCTGGGAACAGGAAATAAAGCAAGCTACTTCCACTAATCAGTACAGAACACAGAACTACAGCTTGGTGTTCAGTACTTCATGATTGGGTTGAGTGATAAATAATAATTCTTACAAGCAAACAAATGGATGACTATGGATTTGTGTCTTTTCAGAGTGAAAGAATAAATATACTCATTCGGATGAATCATAATGTCATGCAAAAAAATATTTGTCATAAAATAAAACACACACAAAACTGAATGCTGATAACAAATATCTAAAAATGATTATAAAAACAAAAACATTTTTCTGATGATTATTAGTAACAAACTACTAGATAGTTATATTCATTCTTTTATCCTCTAGTTAAATAAATAAACAAAAAATAAATAATACTAAATCAAAGCAATTTTTGATACAGTACTTGACTGCAATATATTGTTCTGAGTAATTGTAGTTTTGTTTTGTTCACAGAAAAAGCTACAGAACCTTCATTAAAATGGTGTCATCATCAGAGATTTATAAAGAATTTTTCATTCACCAGCATGCATATCCTGTCTCATATCCATAAATACAACTACAACCTGCACTAGCGCATACACATTAATTATGGCTTACACTATTACACACATATATGTGCAACAATAAAATGGTGCTTCAATTAGTCCCCCTTTAAGTATCCAAAGTTGGACGCAAATAAATAAATAATCAAATAAAAATTTTAAAAAGCTGATTAGTGTAAGTAATCACCTTAATCATATGAAAATAGAAAATGTTTTAAATATTTATTTTAATTTATTTTACTAAAAAAATAATTGTATAAATTTAGAGATATTTTAAAATATGTTATATTAATTAGGCTACATTAACAATAATGTTCCTAATTTAATTTCTTGACCTTACATAATGTTAAATTCTAATAATTTAGACTATAAGACTAGATATAAATGTAAAAAAAAAAACAACATATTTTTTATAGAACATGTATAAGTATTATTACTAGATTTTATATTTGCCTCAAATGAGATTTTTAAAGTTTTATTCTAATTCAAATTCCATTTTTTAT
>Bg-c24786 len=382 count=3 IPR:IPR015421:Pyridoxal phosphate-dependent transferase, major region, subdomain 1; IPR002129:Pyridoxal phosphate-dependent decarboxylase blastx_SP:sp|Q9Y600|CSAD_HUMAN/Cysteine sulfinic acid decarboxylase OS=Homo sapiens GN=CSAD PE=1 SV=2/2e-14/114-278/338-396 // sp|Q9Y600|CSAD_HUMAN/Cysteine sulfinic acid decarboxylase OS=Homo sapiens GN=CSAD PE=1 SV=2/2e-14/277-372/400-430 // sp|A6QM00|GADL1_BOVIN/Glutamate decarboxylase-like protein 1 OS=Bos taurus GN=GADL1 PE=2 SV=2/1e-13/126-272/374-422 // sp|A6QM00|GADL1_BOVIN/Glutamate decarboxylase-like protein 1 OS=Bos taurus GN=GADL1 PE=2 SV=2/1e-13/277-375/428-459 // sp|Q80WP8|GADL1_MOUSE/Glutamate decarboxylase-like protein 1 OS=Mus musculus GN=Gadl1 PE=2 SV=3/2e-13/126-257/403-446 // 
ATTTTTATAGGGTTTGCCATGGATCCAGAAGAAGTGGAAGGCGATGATTTTGAAGATGGCCCAGATGTAGAAGATGATGAGCCTTTAGATGAACCAATAGACAATGACCAGGGAGACAGAATAGAAATTATGATATAGGAGATAAATCTATACAGTGTGGTAGGAAAGTAGATGTCTTGAAGCTTTGGACCCTGTGGAAGGCTATAGGAGATCTTGGGATGGAGAATAGAATCAACAAAGCTTTTGAAAAAGCAAGATCTGTTAAAGAAAAGTCTAGGGTTTAGACCAGTTTTACCAGAATTTCAGTGTGTCAGTGTCTCCTTCTGGTACATCCCAGAAAGATATAGAGGGCAAGAAGAGACACCAGAGTGGTGGCTGGCTA
>Bg-c12787 len=275 count=8 IPR:/ blastx_SP:/
CTGGTTGTCTAATGGAGCTGCGTGTCAGCAACAAAAATTGCTTTTTAAGATGTCCCTTTGGTAATTAACTTGAGGAGCCTAAATAACTGGCACCTTTTTTTTTTCTTTCTGACACTTCTCTTGGTCACATGCACCAGATTTAATTTTGATGCTTGAAAACCCAAAACAACAGCACATTCTTGAAAGTCTAGCCTCTAGTTAAGCCTGGACCTCCAAAATAAAATGTATAAAATCTCATCTAACGACCTTTCTGAATGGTCACCTCTCAGTGTGGT
>Bg-c19843 len=485 count=4 IPR:/ blastx_SP:/
GACAACTTTGGTATTGTCTACATTGAAATAAATCGCAAATCCACCAAGGTACTCTGGTTTCTCCACTATTATTCTGATGTATGTGAAAAAACTTTTGTCTTTCAACTTGATGCTCACTGGTACTGTAATGTTGTCACACGAGCTGTCGTTGTTGTCGGCTAGGAGGCCCAGCGGCCCATTCTCGTCCCCAGACAGACTTCTAATCTGACATCCGGGATTGAACCAGCCCTTCGCGCATTCGGCAGAATCCCCCTGCACAGCTGCCGTCACTTGAACAGTTGAAGTCATGACAATGGCAGACGAAGGAGACGTTGTTATCAGAAAATACGTTAACAGGACCAGGGATGCGATATATGCAAAGTGAGTTCCGTCGAGCTGTGCACCAAGTGTCATACATCATGAGTCCCGGATTAATAAAAGTACAATCTTCCTCCCGTGACAATATTTGGTTGTTCGGTTCGCCCTTCTTCCAGTATTTTTTCCAA
>Bg-c20488 len=394 count=4 IPR:/ blastx_SP:/
TAAAGTTTCACCACTGACCACTGTAGCTTTTAGATAGCACTGACCTATCCATTAATCAAGTATTGCTCAGTTATTGCACTTAACTATATATCTTTAAAAAAAATTAACATAACCTAAAAATAGACACTATCAGTACATTGGCACTAACTGAAGTAAGTAGGTCAGTTGTGTTGTCAAAATAGTTGATTTTTTATTTAAATGCTACATAAACACAAACTTTCAATGTTTAGAGCAAAAAAAAATGTAACATGGCTACATCTAATTTCTACAATTTTAATTACTGGTGTAGACAATTGGGATATTCTAATATTTCCCTATCAATAAAGTCATAATAGTCTACTTATATTTTTAAAAAAATTGTTAATAATCCATTCTGTCAATGTAATCGAACATC
>Bg-c8611 len=128 count=13 IPR:/ blastx_SP:/
TAACAAACACTAAAATTGTTAATTCACTAGAGCAAATCTTTAGTGACAATTAAACATTTGCTCAGAATTGTTTTACTAAATGTGAATAGACTGTATGCACCAAATAAACTTTACATTCTCAACTACTT
>Bg-c23344 len=621 count=3 IPR:/ blastx_SP:sp|Q6P829|NOSIP_XENTR/Nitric oxide synthase-interacting protein OS=Xenopus tropicalis GN=nosip PE=2 SV=1/8e-47/505-2/1-177 // sp|Q5U3S7|NOSIP_DANRE/Nitric oxide synthase-interacting protein OS=Danio rerio GN=nosip PE=2 SV=1/1e-46/505-5/1-182 // sp|Q3SWY5|NOSIP_BOVIN/Nitric oxide synthase-interacting protein OS=Bos taurus GN=NOSIP PE=2 SV=1/1e-46/505-5/1-180 // sp|Q9D6T0|NOSIP_MOUSE/Nitric oxide synthase-interacting protein OS=Mus musculus GN=Nosip PE=2 SV=1/2e-46/505-5/1-179 // sp|Q6NUH3|NOSIP_XENLA/Nitric oxide synthase-interacting protein OS=Xenopus laevis GN=nosip PE=2 SV=1/2e-46/505-2/1-177 // 
CATCTGGTTTTTTAATTTGAGTTGCTTCAGCCTGTGGCGTGAGAGCTGGAATCCAGAAACTGGGAAGCTTCTTGTCATCAGACAAACCCACAGACTTAGTTTTCTCTTCTGATAACTTGTTGTCAGCATATTTCTGACCAATAGGATTTGATTCTTGCTGGGCAAACTTGAGAGCTTTAGTTTCTTGTTCAGCTTTGGCTAATTCTTCTAGTTCTTTCTGTGATCTTAATTTTTGTTTTTCATATTCCTTTAGTTTTCTGGCAATTTCCTTTTTCTGATGCAATATGTTTTTGAGAATAGCCTCCTTGTCATACAGGTGACCATCTTCCGTTACCACTGGATCTTTACATGGCTGTAAAGTCAGACAGCAACAGTCAAATTCCTTGACTGCATCTTTCCCAAACCGTACTTTTTGCGAACCAAAACCAGAAGCGTGTGTATCCTTTTTCCTTTCATGATATGTGTATACAGTACCTGCTGTACAATTTCTGCCGTGCCTTGTCATTATATATCTCAATATTTAGATATATTTACTAGACTTAAGCTGTACTTAATTTCTTAGATTTTATATAGATCTATAAACTTCAGTAACTTTGATTCAGTCTAACGACAACACACAAT
>Bg-c11763 len=921 count=8 IPR:IPR002861:Reeler domain blastx_SP:/
AACTACAACACTCAAATCTGAGACCCTTTAGATTTAAGTGACATTTAGTAGTCTTAATAACCAACAATGTATTTCCACTTAAAGATAGTGTTTTTGATGCTTGTAGCTTTGACATTTGTTGTTACCGTTCGTGCTGATGAAACGAAATGTTTGGAATCTCTTGAACCAAATTCAAGCGTTCGAGGTATCAATGAACTCAAACCAAACCAGCCTCTGTATCACCTCCACTTTGATGAACACTTTTTTAAAGACAGTCACGTGGGACATAGTCAATACAGAGTTCATGTGTCCAGCAAGGATCCCCTAAGACCTTTCCAGTCCGTCAAAATCACAGCTGATACCATTGACGCGTGCGGTGCTGGGGAGTTTGTGTTTAGTGATAAAGACTACACAGACAACAAGGACGATCAGTGCACATACATTCTGGAAACCGCGACAGACAAGGCAACTCTTGACACACCATCTCTTACTTGGAGACCTCCATTTTGTGGCTGTGTCCATTTCAGGGTGCAGGTTGTTGATCAAAATAATGTTTACTACATGGACGCGAAATCCACGAAGAATGGACAACTGTCACAGACAGTGTGTGTTAAAGAGAGGCTGACCAGAAGTCACTACATGGAGGCAATGTGCACGGCCATCAATCTCAAAAATGCCCAAGCTGTCATTGATAGTCCAGCTTTCCTCAGCCGACATGGACTAGACGCTAAGACCATGGACAAATTTAACTTACTGATGGACATGGAGTTTAGAAGAACCCACAACATTGAGTGCTGCCAAAAAGAAAAATCTGGCAGACAGGTTGGAGTGTTTGGGTGACAACAGACGTAGACGTATTGATAAGTTCTGTGCATATGGCATGTGATATTCCTTTGCTACATTTACGAAATGCACATATGACGTAATAGAGAGTCACACTGT
>Bg-c42661 len=149 count=2 IPR:/ blastx_SP:/
GCTTGCTCGAAATATGAGTTGACAAAACATGACTTAATTAGCACTCTGCTAACAGCCTAAACAGCAAATGAATTAATTCCTTGCAGAAAGTTTGTAAAAAAAAGAAAAATACTACTTGTGCCCGCGTCGGATGCGTTGTTACCACTGCT
>Bg-c31174 len=428 count=2 IPR:/ blastx_SP:/
CCCCGAAGTCTGCTTTGAATCGGCTTTGAATGCTATAGTCCAAGGACATTGATGGACCAGTCTATAAACTAGATGTGAGTATCAAACGATTTCATTAGAATACGGCTAAAGATTCATTTCGTAATCCTAATGTCCTGCAATGAAAATTTCTTGAGTATTTTTTGCGGTAAATGTGTTTTGGCAAAATACTAAAAAACAAGAAAGAAGAAAATTGCTGTCAGCTTTACTTTGCAAACGAAATCTTTGATTTAAAGATACATTTGTAGGCCTATTGATTGGCTATCTACATTGTTATTTTTAGATATATGTATGAAAGATCTAATGCTAATCTTAGCTGGTTTTCATCGAACATTGTGATAGCAAAAGTAGGTGTATACAATTTTGAATAGTACTGTGTCTTTATTTTTAAGTCTTAAGGCCATTATTAA
>Bg-c33041 len=339 count=2 IPR:/ blastx_SP:sp|P55828|RS20_DROME/40S ribosomal protein S20 OS=Drosophila melanogaster GN=RpS20 PE=1 SV=1/4e-06/255-338/65-95 // sp|P23403|RS20_XENLA/40S ribosomal protein S20 OS=Xenopus laevis GN=rps20 PE=3 SV=1/7e-06/255-338/63-93 // sp|P60868|RS20_RAT/40S ribosomal protein S20 OS=Rattus norvegicus GN=Rps20 PE=3 SV=1/7e-06/255-338/63-93 // sp|P60867|RS20_MOUSE/40S ribosomal protein S20 OS=Mus musculus GN=Rps20 PE=1 SV=1/7e-06/255-338/63-93 // sp|Q4R5D0|RS20_MACFA/40S ribosomal protein S20 OS=Macaca fascicularis GN=RPS20 PE=3 SV=1/7e-06/255-338/63-93 // 
TAGTACTACAATAACTACTATTAACATTTAGAAATATTTATAGTGCAGAATATTACCACTAATATTTGCTTCAAGTTTTACCACTTCTAATTATGGGTCAGTAGAAAATGAGGTTATTTTATTCAATTTATTCAAATTTTCTGAGCTTTATTTAATCCTGCAGTATTGATACTGATCTGTCTATTTTTTTTTATTTTTTTTTTTTTTTCACAATACAACTTTTATTTAGAACAATTTATGCAGCATCTGCAATGGTGACTTCAACTTCCACACCAGGTTCAAAGACCTGGGACAGATACCAAATGAGAATTCACAAACGTATCATTGATTTGCACAGTC
>Bg-c16902 len=519 count=5 IPR:/ blastx_SP:sp|Q99MR8|MCCA_MOUSE/Methylcrotonoyl-CoA carboxylase subunit alpha, mitochondrial OS=Mus musculus GN=Mccc1 PE=2 SV=2/8e-16/2-472/465-619 // sp|Q5I0C3|MCCA_RAT/Methylcrotonoyl-CoA carboxylase subunit alpha, mitochondrial OS=Rattus norvegicus GN=Mccc1 PE=1 SV=1/4e-15/2-472/465-619 // sp|Q96RQ3|MCCA_HUMAN/Methylcrotonoyl-CoA carboxylase subunit alpha, mitochondrial OS=Homo sapiens GN=MCCC1 PE=1 SV=3/2e-13/2-469/469-622 // sp|Q54KE6|MCCA_DICDI/Methylcrotonoyl-CoA carboxylase subunit alpha, mitochondrial OS=Dictyostelium discoideum GN=mccA PE=3 SV=1/2e-04/2-379/450-580 // 
TTTCCTAATGGCTCTAGCATCCCACCAGTCTTTTAAAGCAGGGGATGTAAACACTGATTTTATCCCACAACATCAGACAGAGTTATTTCCTCCACGCTCACTCAGTAAGACCTCCATGGCTCAAGCAGCACTTGTGATGGTGGCTAGTCACAGAATGAGTGTTGCCAAGAAGGTTTTGTCAACCAAAGACAGCTATTCTCCGTTTGCATTAAATTATAACCTGAGACTGAACACTTCAGCAAGCTATACACTGAGTCTTGCTGATGGAGACAGTGAGCATAAAATCACATTAACTGAAGTGGACCCCAACAAATGGCATGTGGCTGTGGATGGTGGGGATCAGCTGAAAGTCAGTGATGTTGTGCTGACGGAGGAGAGCTCCGACAAGTTTACGGCGAAGTGTTGTGTGGACAACACAGTTTCTACCATGACAGTATTACTGCAGGGCAACTCTGTCCACTTGTTTACTGTGTATGTAGAATAACAAAAAGAAAAGAACAAGACCAAGAAGAGTGGGGG
>Bg-c33078 len=338 count=2 IPR:/ blastx_SP:/
AATATTTTGCATTATGAATATAGATTGTTGAATACACTAGTTCTGGTTAGCAATGGGTGTTAACATGTTTCTACTATGATTTTAGAAGGAAGTAATTCTTTGTCATTCTTATATTTAGGTGTATGTGTGGACTTGGTGCTGATCCATTCCCATAGTTCACTATCACTTTGACCTTTTTCATTTTACGAGGTATATCCCCTCCCATCCCCCCTTTTTAAAAACAATTTTATAATTTTCTAACCAGTGTCCACACATGCATACACCCTTGTAAAAGAATTACTTTTAGAGTAGAAAAATAAACTATGAAACAACTGAGACACGCAAGGGGATAGGCAAGG
>Bg-c19330 len=672 count=4 IPR:/ blastx_SP:/
CTGCAGACCTCGGACCCCTTTTGGTCGTTTTGCGGACCCCATTCTTGTGAATGAAACGCTGCTGTTTTTTTTTGTTTTTTTTTAGTTAGAATTTATTTACACATTGTGATAGTCTAGTCTTCTAATTTTAATAGTAATTTTATCTTGTTATCTTAAGTCAATCATTTTTAGTTGTTTAACTATTCAGTGTTTCTGTATACTATTTAGCTCTAAATGAGAGATGTATGTTTCTTAATGTGAAATCGTTTGTCACACGTACATACACACACATCTAATATCTATACATTGTAAATCTAATATATATTTACAAGAATTTTATTTTTAGTTTACCCCATTTCTAATTTAATAACTATAATTTAATAACACTGAATCATGAATGAAGAAAAAAATGGCCTTCTTACATATATATTGGATAGACCCGTAAGATGTGTACTAGGCTACATGCCAAAAACGTCCAGAGAAGTCTTGGTTTATTTTTAGGTATTATTATTTATAAAACTAGGATTTATTTATTTATATATTTAATAGACTTGATGTATTTCTTGTGTGTATTACTGATGTGTGTATTACTGATGTGTGTATTACTGATGTGTGTATTACTTATGTGTGTATTACTTATGTGTGTATTACTGATGTGTATTACTTATCACATTAAAATTACGTCAAAGTTCC
>Bg-c15905 len=289 count=6 IPR:/ blastx_SP:/
AGTGTTCAAAAGTTAGATCTAGCTTAGTCATCTGTTGAGAGTAGTCCAAATTTGTTGCCAACTGGTTTCAGTCCCCTATTTGTTTTACTCCCAATGTTATGGTTATAACAAATATGTTATATCTCTTGATCTAAATCTTATTTAATGTATTGTTCATATTTATATATGACATTTAATTAGGTCAGCAAGAAATGTTATTTTTGTTTTAAATGTATTTATTCTGTAAGAAAGTATGTATGTGTGCTGTTTTGTGTAAATCTGTCAATTGTAAATAAATGCATTTGAACAT
>Bg-c1624 len=1802 count=83 IPR:IPR000998:MAM; IPR001304:C-type lectin; IPR016186:C-type lectin-like blastx_SP:sp|A7X3X8|LEC5_ENHPO/Lectoxin-Enh5 OS=Enhydris polylepis PE=2 SV=1/1e-10/1036-1389/46-155 // sp|A7X3X0|LEC3_ENHPO/Lectoxin-Enh3 OS=Enhydris polylepis PE=2 SV=1/1e-10/1036-1389/46-155 // sp|A1XXJ9|CLEC2_BUNMU/C-type lectin BML-2 OS=Bungarus multicinctus PE=1 SV=1/3e-10/1009-1389/37-155 // sp|A7X3Y6|LEC7_ENHPO/Lectoxin-Enh7 OS=Enhydris polylepis PE=2 SV=1/4e-10/1036-1389/46-155 // sp|A7X3X3|LEC4_ENHPO/Lectoxin-Enh4 OS=Enhydris polylepis PE=2 SV=1/6e-10/1036-1389/46-155 // 
CTCTAGGGTTGGCTCTGCTGATCATCGTGGGTACCCCTTTAGGGTGGACTTTACGCCTGGCTGTAGTTTTATTGCCCTTTAGTGCATGTACACTGTCCAGCTTGGCCACCAGGGTCAATGTGACGCACAACCGGTCGCTGTGTCCTCAGGAGCTGACTCCCAATGTTGTACGAGTCGTTGCAAGGAGAGAGAGACTCTCTTCTGGAGATGTTACACAGGCTTGAGACGGAGATGAACAAAACAGAGTCACAAAACTCTCACATCTTAGCTCTTGCAAGGGACGAGATCGCTTCTCTTAGAAGAGACCTGACGAACATGACCTTAGCTCTACGTACATGTCAAGCTGCCCTAGGTGTCACAACTGGCCAGAATGTTCTGCCTGATTCTTTAAGTGGAGATGTTCACCTGTCTTACTGTAATTTCAACTCAACGGACATCTGTGGCTACACTAAACTGAGTGGCACGACATCATTTTACTTGCTTCATACAGGAAGTTCCACTTTAACAGGACCTCGGGTTGAACACAGTGCAGGCATTAACACTGGTACCTATTTGGGCCTGGACATAAAAAATGAGCTCAGTACAGAACGGCAACCAGGGCTTCACACGGCCATTATAGAAAGCAGCCTGTATCAGCCGGCCAACGGCTATTGTATTTATTTTTGGTACTCCATGAGAGGAAGTGACGTCAGGCAACTCGACGTCAATATCCGAATTGGAGGAGGTACTGGCTACCCCGTGTTTTCCAGATCTGGAGATCAAAAAATGGACTGGTTGCTTGGTCAAGTTGACCTAGACAGTGAATACACCTCACATCCATTTAAGCTTGACTTTGTTGCTACTACAAATGCTTATAAGTCCTACAATTACAACAACCGGAATTATGACTTTAATTACATCAACTCAGACATTGGGATTGATGACGTTTATGTGTACAACACGTCATGTGCAAATATACCAAGATGTCCACCAATGGCTGTCAAACACACACTGAACAACGTCACTTCCTGTTACACGTTCCACGCCACCCCAATGACATGGGCGGAGGCCTATGACTTCTGCAGACGAGAGGGACCCTATTCTGCCCTTGTCAGTGTTGAGACAGAAGCGGAACATATTTTCCTGGTCAACCACATCAAGCAAGATACAGCTTTATCTGTGGTGGGACAAAATGGTTTCTACACAAGCGGAAGTGACGTCGGGAATGAGCACAGCTTTAAGTGGACAGACACTGGCATCCCTCGGCCAGTCACTTGGTCAGCCGGTTGGCACACTGGTCAGCCTAATAACGTAGGTGGCAACCAGAACTGCTTGTTGATGCAGTACCCAGCTGATGACTACAAATGGGGCGATATAGAGTGTGACAGCAAGCACCCTTTTATTTGTGAAGTGTACTACCAAGTTTAAGTTTTGTATAGAGTTTAATAACAAGCATCATTTTATTTGTGAAATAGATAGTAGTCACACTATATTGTATAGGGTTTGATCACAAGCACCCTTTTATTTGTGAAGTCTACTACGAAATATAACTTATGTATAGAGTTTGATAACAAAGCATAAGAAACATAGGAAATACTTTACAAATGACACAAATAAGCTTATACGAAGTGTAATTGTATCACTTTGTTTAGAAAAGTCATGTCATTAAATTTGTAATAGATCTAGACCAAGAACAATAAATGATAACTAAAATTTTTAAAAAAAAAAAAAAAAAAAAAAAAAAACAAAACNGAAGAACGAACGAACAAACGGAAGGGAAGTACGGAACGAACGGACCAACGAACGGGAAGGGGTAAGTAGGA
>Bg-c99 len=934 count=409 IPR:/ blastx_SP:/
TAAGTGTCTTAACGCGTTGTAGGAGCTTTTGTTGTAAATTTAAAGAGCTCATTGATAGTTCTTCTTTTGTAGGAGTTACAGTTTTCCGACGTTATTATGAGTAGGTCAACAATGGTTCTCATTTTCACTTCACTTTTGGTTTTCATAAGTCTAACCTCTGCTGAGATGACCGTCCCACGACGTCCGCTTGGGTTCGTTTACAAAGATGGCCAACCCACCGCTTCAGTTAGACTAGCTTCCTTTCTTGACCTGACATGCCCGGACTCTCTGGAGGCATTTAAAATCTTACTCCAGGTGGCCGACTCCTTCAGTGACCGCAGTGTCCAGCTGCGATTGTATCTCTTCCCTCTACCTTATCACACCAACAGCCATCTCCTGTCTAAGGCTGCGCGCTTCCTGGACGACTTCACGAAGGTGTCTCCTTCCAATGCTACCGTTTTTGACTGGATCCAGCTTGTGTTCAGCAACATGAGATATCTGAGCACCAGGGCTACATCCAACAGCACCGAGGTTGAGGTTGTGGACTATGTGACGTCTCTTGCCCAGTCACTTTTCCACGTATCTGCAGAGCAGTTTAAACATGGAATTTACAACGCCAGTATAGACAGAATGACCAGGCTGGAATGGAAGTACGGCGCTACGCGTGGCGTCTACGCCACCCCAATGTTCACCATCAATGACGTATTTGTCAATGGGGACGCGTGGAACGTGTCCGAGTGGACTGCACGTGTGGACAACCTGTTGCAGGACAGTGTACACAGCGGCTGTGGAGCGAGGCTAGGGGCTGCTACTGTTGTGAACCTGTTAAACATTTTCGGAGATCCCTTCGGAGCGCTCAGTGGCTTAAGTCATATACATCTAGCAGTCTTTATCACTGCGTTACAATGTCTTGTTTTCATACATGAGTAGGAATTAAATGACCATTCTAATAGAA
>Bg-c39140 len=252 count=2 IPR:/ blastx_SP:/
TTGAAATTTTCATTGTGCGTTGGAAAGAATGACTTCATTGTTTATGACGTGAACACTTTGTCCTGATAGGAATGTACACAACAGTACTTATTGCGGATTGTTTCCATTGACAACCTTTGTGATCAGATTCTTTTCATTTCATTTTTAATATGTATGTCATCTGAACATATTTACTTAGAATGTTTTCTTGTACCTTCTATCAATGATTTGTCTTACACATTGCATTCATAACTAATTCACCAATTCATAATA
>Bg-c12523 len=370 count=8 IPR:/ blastx_SP:/
ATGTGTTTAGGAGCACTAGTGTAAGTGTGAGCTATTCAAGCTGTGTATGTGTTTAGGAGCACTAGTGTAAGTGTGAGCTATTCAAGCTGTGTATGTGTTTAGGAGCACTAGTGTAAGTGTGAGCTATTCAAGCTGTGTATGTGTTTAGGAGCACTAGTGTAAGTGTGAGCTATTCAAGCTGTGTATGTGTTTAGGAGCACTAGTGTAAGTGTGAGCTATTCAAGCTGTGTATGTGTTTAGGAGGAAAAAAGGGAGAACAACTGTGTATATGATTTTTATGTGTTAAAACAAAAAATGCAATATTTTATTACCTTTCATCTATGGTTATTATTGTTGATTGCCCATTTTAAATAAAATTTTACATATTCCA
>Bg-c40573 len=239 count=2 IPR:/ blastx_SP:sp|O08837|CDC5L_RAT/Cell division cycle 5-related protein OS=Rattus norvegicus GN=Cdc5l PE=1 SV=2/1e-26/231-1/110-186 // sp|A7SD85|CDC5L_NEMVE/Cell division cycle 5-related protein OS=Nematostella vectensis GN=cdc5l PE=3 SV=1/1e-26/237-1/106-186 // sp|Q6A068|CDC5L_MOUSE/Cell division cycle 5-related protein OS=Mus musculus GN=Cdc5l PE=1 SV=2/1e-26/231-1/110-186 // sp|Q99459|CDC5L_HUMAN/Cell division cycle 5-like protein OS=Homo sapiens GN=CDC5L PE=1 SV=2/1e-26/231-1/110-186 // sp|Q2KJC1|CDC5L_BOVIN/Cell division cycle 5-like protein OS=Bos taurus GN=CDC5L PE=2 SV=1/1e-26/231-1/110-186 // 
CTGTAATGCAGCTAAACGTCTTGCTTCCTCCAGCTGCTTTTCACGAGCCTTTCTTTTTGCCTTTTTACCTTGAGTATTGGCAAGCCTGGCCCTGGCTTCTGACAGCATTTCCAGCTCATCTTCATCCATGTCAACTGGATCAGGTCGAGCTGGCTTGGTCTCAGGGTTTGGGTCAATCTCTCCAGGTTTGAGTTTCCTGGGATCATCTTCATTGTCAGCTTCTCTGTTCTGTGCCTTGT
>Bg-c1876 len=1400 count=71 IPR:/ blastx_SP:/
AATCTCCACAAGGATTGGACTCTAGCATTTCGCGGAACATCCCCTTTTAGGGGATCTGTGTATGACGCATACAAGCTAGGGCTAGGCATTCCCGAAGATGTAGAAAGCGGATGCATGCAGGTTGGTCAAAGTTTGCCCTGCGCCAACCACTACAGAAACAACGCCATCTTGGACAACTGGAAAGACATCTCTCAAGTTGCACTGGTGCTCTACAAAGATAATGTCAAAGTAAAACAAGTCATCTTTGACGGTGCCGGCTCTAACTATATGAACTGGCTGACCAAGGCAAGAGTCCTTGATTCGTCATGGAGCGACATGAAAACCCAAGTGTCCAATATTTTCAGCATCGATGGAGATATCAGACCAGAACTTAAAAGAGTCTTCTTGCTCAACAGTGTCTACGGTGGTTGTCCAAACGATGTCGGCTGGTTCATGGCTGTTGACATGGAAACTGACGGCTGTAATTGGGCGAAAAACCCAGATTTTCCAATGTTCTTATACTCCATGTCCAGCGAAAGGGAAAATTACAATAGCGTTAATATCTCAACCGCTGATTACTTTGCGATCTTTGTACGAAATTTTAATCTCCCTTAAGGCTATGTGGGACACTAAATTTAGGTAAAAAGGTCACAAATTTCGATTTTCAGTTTTAACCTAATTTAAAAAGCCCAACCATTTCTGGACATAAAAATATCATTTTCATGATTGGCAAATAATTACTTATTTTTTTATAATTTTTTGGTAAGGTATCTACAGCTGTCTCTTTTCCAATTTTGTTTCGTTTTTATCGATTTTCTCAAAATACTATTTTTATTCATATTTTTTACTACTCCTTCACTAATTCTGCATAGAATTAAATCAAACTTGTCCTGCTTATGACTTACACCACGTACAACAACTTCTTGCTTACATTTTCTAAGTCTGAATTAATATTTTGTCTTAATTGCAATAAAATACTTGAAAAATGAAAATAAAATTTATGTTTTTCTATAGCAAGGTTATAAAAAAAAATTTATAAAAAAAAATTTATAAAAAAAAAGGTTTTTACTATATTTAGAAACTAAATGCTAGAAAATGTTCTGCTATGATTGATTTTCACATTGTATATTTTTGCTTGGCAATAGCTTAAATAGTTTTAGAGCCTTAGTGATTTAAAAACGATAAACAAAAATCCAATATGGCGGCCGTTTCCATGACAACCATTATAAGCAAACAATTTTAACCACATTTTTTTTAAATGGTTATTCATAGGGATTATACATATAAAGTTTAGAGTAATTTGATCAATAAATTTAAAAAAAAGTTGGCAAGGGTCCCACATAGACCTAACCGAAGTCATTTGTAGGCCCTATATGAGTTTTGGAAATAAAAATGATTGTATCCAAAACAATAAATATTTT
>Bg-c39979 len=245 count=2 IPR:/ blastx_SP:/
CCTTACTGACTTAACACATTCTCTCGCACATTCAACGAAGACTTAATCACTCAGTTCACAACACGTGCAACACTTCGCATACATAACAAGGGGATATAACAATAATATCAAACTAACATTCATTCTCGCCATACCTCTCCCTGACATTCCTCAAGCGATCGTTTCCACCCGAGTGCCACGATGAGGCAGAATAGCGTACACAGGCTATCCTTCTTGCCTCACTCCATCTTAGAAACTTGGTTACA
>Bg-c32413 len=364 count=2 IPR:/ blastx_SP:/
TAATTATTAATTGTGGCATGTTTTCAATAAGTTTATGGAAAGTTTCATTTTATTTCTGGATTTGAAGTAGTCAACAGAACTAAGTCCTAGGTTACAATCACAGACCTAACTAGATGACTTTGATAGGACTGAAGGTTTATAGAATGGTATTTTATAAATGTGTCAGACAAACTACTGACGCTTCTCTAATGATGCCTGATGTTCAATCAAAATACTTGGATTAAATCTAAACCTCTTGGCATTGTCTGTTAGCATCTTGTACATTGAACAACTGTAATGAATATTATAATCAAGGATTTTTTTTTTGCTGTGAAAATTTGTTTCAACAACACAATTTTAGAAATGTTAAAAGTCTGTACAAATG
>Bg-c236 len=1891 count=303 IPR:IPR000504:RNA recognition motif, RNP-1; IPR012677:Nucleotide-binding, alpha-beta plait blastx_SP:sp|Q6AYL5|SF3B4_RAT/Splicing factor 3B subunit 4 OS=Rattus norvegicus GN=Sf3b4 PE=2 SV=1/2e-104/1528-887/1-214 // sp|Q8QZY9|SF3B4_MOUSE/Splicing factor 3B subunit 4 OS=Mus musculus GN=Sf3b4 PE=2 SV=1/2e-104/1528-887/1-214 // sp|Q15427|SF3B4_HUMAN/Splicing factor 3B subunit 4 OS=Homo sapiens GN=SF3B4 PE=1 SV=1/2e-104/1528-887/1-214 // sp|Q09442|SF3B4_CAEEL/Splicing factor 3B subunit 4 OS=Caenorhabditis elegans GN=sap-49 PE=1 SV=2/2e-97/1528-890/1-213 // sp|O14102|SAP49_SCHPO/Spliceosome-associated protein 49 OS=Schizosaccharomyces pombe GN=sap49 PE=2 SV=2/2e-63/1507-938/6-195 // 
TCTCTATTATGTTTATTATTATTATGTTGTTTCTCCGTGAGGGGAAGACAGTAATTGTCAAATTTTAACTAAGCCTTTGTAACAATAACAGTACAGTAAGAAGCAATATAGAAAACAAATGTTTTGGATTTTTTAAAAATGTGTTTAATAATAAAAACATCAACAAATCAGGTTTACACTGTTCCATTTAAGCCATCTGTGCACCTGATAGATCAAACTAATATTCAATGTTGCCTGGGTTACCCATGTCCCCACTGCCTCCAGCTCCTGTGAACCGTGGTGGGGGTGGCCTCATTCCTGGTGGTGGTCCTCTCATGGGACCTCTTGGTGGCATTGGGCCCCGGGGGCCATTCATCATATTGTTGTTCATTGGAGGTCCTCTCATGTTGGGTGGTGGTGGGCCAAAACCCATTGGAGGTCCTCTGGGCGGCATCGGGCCTCTCCATCCTGCCGGAGGTGGCCTCATGCCAGGGGGTGGTGGACCACCATAATTCATTTGGCTTTGTGGTCCCATGTTCTGCATTCCTTGCATCTGTGGATTCATCATGTTGTTTGAATTGTTCATCATTTGACCTCCAGTGTAATCCATACCAGGAGGCCCAGTCATAGGTGGTGGTGGAGGACGGGGCATCATCTGGTTGCCCTGGAAATTTTGTCCAGGGGGCATTGGTGGAGGTGGCATTCCCTGCATGCCCATTGGATAGTTCTGTGGCATACCAGGTGGAGGTAAAGGTGAAGGGGCTGGCGGTGGTGGTGCCATTGGAGGCATGGTACCCATTCCAGGCATGACAGGGGGCATGCCAGGTGTGAGCCCCATGGGTGGAGGTGGAGGAGGTACAAGCCCAGGTACACCAGGAGCCAGTGGTGGCTGTGACGGAGGTGGAGGAGCATCAGCAAACAGTTGGTGAGGTCTGCCCGTTAAAGACAAAGGATTTTGTGCAGCCAACAAACGCTCTGCTGCTGAACCACATCGTTCTCCCTTGGCATCCTTTTTGAAAGCGTAAGAAATGGTGATGGCCCTGTTACAGAGATATTGGTCATTCATAGCTTCAATGGCAGCATCGGATGCTTCAAAGCTTGCAAAATTAATGAAGGCATAACCTTTAGAGTTGCCAGAGTCTCTGTCTCGCATTATCTTGGGTGTCTGTAAAATAACTCCAAAAGCACTGAATGTGTCATACAGTAATTTTTCATCTACTTCAGGATCTAAGTTTCCAATAAAAATATTAGCACCAATGTCTTGTCTTTTTGTATGGGCTGATGCCTTGTTGACTCTGATGGGTTTGCCATATAGTTTAATCATGTTCATGATCTTTATGGCATAATCAGCATCTTCCTCACTCATAAACTCAATGAAGCCATAGCCCTGGTGTGACTGAGTGACTCTGTCTTTTGGCATGTGAACATTCACTACAGGGCCTGCTTGCAAGAATAGTTCCCATAAGATATTTTCTTGCACTTTTTCATCAAGGCCACCAACATAAACTGTAGCATCTTGATTTCTTTCTGCAATAGGTGCAGAAGACATGTTAACAATTATTTTTATTTCACAATATCATGGGATGGATCTTGTAGTTTAGGTCTCTAAAACTATAAACATGAATTTGGAAAACTGGACTTGGCCTGGATGCTACGATTCAGCTGTGGCATTCGCTTATTGTCGCCATGATTGCAATTTTCATTATTTCAATTTTAGTATCTTGTGCTATGATCAAATCCCAGAATGCTATCTTGTACATGGTTGGTATGTAGAATCTGGGTGTGATAGATATTCTGACAACATAAGCTGGAATCCATCATATTGTGATGATAGTTATTTTAATACATTCGACAGCTACTGTGTTCATGGGGCTTAGTCTTAAAAATATAAACAAATAAATGATCCTTTCCA
>Bg-c32372 len=366 count=2 IPR:/ blastx_SP:/
ATTTCAAAAAAGGGAGATAAAAAATACCAACTCACTAAAGTTAAATTGAGTTTTCAATCAAACACAGAAGCCCTGAACTAAAATATTAACATGATTGTTACACATTTGTACAAAGAAATGCACCTAAATAAAGTTGATTTTTGTTTGTTCCAGATTTAAACAGTTCTATTCATACTTGTACATTACAATTTCATTCTCTCTTGAAAGAGATAGACAAAGTAAAGTGCTGCTTTGAGACCTTGCTATCTATGGCCAGATAATGTAAAGTTCATCATTCCTATGGCCAACAGTCAGCACAACCATGAACTTCCTTTACTTTCCCAACGTTGTCTGGTTCCCTTCAGAGTTGGGTGGACTCAGGGGTGT
>Bg-c16482 len=725 count=5 IPR:/ blastx_SP:/
ACCAGTCCTGCTTCTTGTTAATACTTTAATATGTTAGGCATCAAAAATGAAAACATGGGTAGCTGCCATTATTCTTCTATTTACATTTTTAAAATGTATATTGAAAATTAAATTATGAATGAAAACTGAATGCCACAGTTCAGATTTCTGGACTTGACACTAGCACTGGAAGTTTAAAACAAACTGAAATACTTTCTTTTTAAATCAACAAAACAACTTTTGAACAGTGTGGTAAATCTAAGAAATTCAATTATTATAATGACCAAATATGGATGCACCTCACTACACTTGAACACTTTTGTCTTGGTTTTACAAAAAATGGAAAAAATTACAAATACACAAGTTCAAAAATGCCATTTTAAAAATTCCACTTGCATATCAGATGCAACAGGGCAAAACAATATATTTCATTTATTGACTAAAAAAAATATATCACAGATTCCTCACGTCTAGAAAGTGAAATCTAAGCATTAACTATTTTCTGGTACAATTGTTTTTTGAAATTAAATTTACATCAAATACATAATGAGGACTGCTTAAACATTAGTTTTTTATAATTAAGTTAATTTTAAAAAAATATTTCTAGCATGGTCTAGTTTAAAAAAATTCTTTTGATAAAAGCTCATTATAAAAAATGATCTTTACACCTTTAATTAAATCCCTGCAGCTTTTATAGCCATTTAACAGTCATTTAGCATGTATGACTCCTCTGGCCTTAAAACGTT
>Bg-c34215 len=307 count=2 IPR:/ blastx_SP:/
GATCTCGTGGCTTAATTTAATAATGGGTTGGCTAGTTACAACAATGTCTGATGTTGATTTATGAGGAGTCAAAAACAAATAGAGAATAAAACTTGGTGATGGTACTAGCTATACAAAGCTCAAGGTTCTAAGTCTTGTTCATGTTTTAGGAAGGGAAGTGCAACAAAAACAAAAAGTGAAAATACCCCCCCCCAAAACTATAATTGAACACAAATTTCATACTCTTAATAATTAAAAAATTACACAAAAAAATTAGAATTTTCCTTTTCCCTTGGAAATAGGAGTTAATTTGTGATTGGTAATATAA
>Bg-c42674 len=148 count=2 IPR:/ blastx_SP:/
ATCCCACCATCCAACAAAACACGAGAACAGAAAGTTCACAATACCCACCATCCAACAAAACACGAACACCAAAAGTTCACATCCCACCATCCAACAAAACACGAACACCAAAGTCACATCCCAGCATCCAACAAACACGAGCACATAA
>Bg-c36783 len=272 count=2 IPR:IPR003565:Bis(5'-nucleosyl)-tetraphosphatase; IPR020476:NUDIX hydrolase; IPR000086:NUDIX hydrolase domain blastx_SP:sp|Q9U2M7|AP4A_CAEEL/Bis(5'-nucleosyl)-tetraphosphatase [asymmetrical] OS=Caenorhabditis elegans GN=ndx-4 PE=1 SV=1/2e-13/233-3/5-81 // sp|P50583|AP4A_HUMAN/Bis(5'-nucleosyl)-tetraphosphatase [asymmetrical] OS=Homo sapiens GN=NUDT2 PE=1 SV=3/3e-13/239-3/3-87 // sp|P56380|AP4A_MOUSE/Bis(5'-nucleosyl)-tetraphosphatase [asymmetrical] OS=Mus musculus GN=Nudt2 PE=1 SV=3/2e-12/239-3/3-87 // sp|Q6PEC0|AP4A_RAT/Bis(5'-nucleosyl)-tetraphosphatase [asymmetrical] OS=Rattus norvegicus GN=Nudt2 PE=2 SV=3/5e-12/239-3/3-87 // sp|P50584|AP4A_PIG/Bis(5'-nucleosyl)-tetraphosphatase [asymmetrical] OS=Sus scrofa GN=NUDT2 PE=2 SV=3/8e-11/239-3/3-87 // 
GGCTTGTTTTTAACAACATAGTTGAGAGAATGTTCAAAGTTTTTCAAAATATTAAGCTGTTCTTGTTTGTATCCAGATTCTTCTTCTGTTTCTCTAAGAGCTGTTTCCCATTCAGACTCACCAGGATCTACATGCCCTTTGGGGGGTGTCCAGTGATGCTCGCCGTACGACGTCTGTAACAACAAATATTCAAACTTATTGTGCAGTCTCCTATAAATCAGAAGGCCAGCAGCAACTAAATAATTGAGTGAAAATAGCCTTGTAAAGAGGAG
>Bg-c39506 len=249 count=2 IPR:/ blastx_SP:sp|Q6TFL3|CI093_HUMAN/Uncharacterized protein C9orf93 OS=Homo sapiens GN=C9orf93 PE=2 SV=1/5e-06/39-197/232-284 // sp|Q6TFL3|CI093_HUMAN/Uncharacterized protein C9orf93 OS=Homo sapiens GN=C9orf93 PE=2 SV=1/5e-06/187-249/281-301 // 
AATGAGTGAACTATGTCAGCAACATTCCAGTCTGATATCAGAACTAAATGAAAAATCTTCATGAGTTTGAAACTGATAAAAGAAATGTTTTGGAAAGTCTCAGGCGGGCCACTGCTGAGATTGAATATGCTAAAGACAATGATGCCAGACTTAGAATAGAATTAGAGAATGCTTTTACTTGAATTAAAACCCTTGAAAGAAAATATTGAAATGGAGAGAAGTTCTCATCTGGAGACGAAGTTTAACTCT
>Bg-c8878 len=550 count=12 IPR:/ blastx_SP:/
CTCCTTCACACGGTCATGCTTGTCTGGTTCATAGCTCTTACTATTTCTTTCGTTATTGCTCAAGAGGATTCGGCAGCACAGCCAGTGTGCTCCGTCAATCAGCTATTCAATGGGCAAATTTGTACCTGCGCACCGGGATATTTTTCGTCGACTAATGATGAGTCTAAAAACAGATGCGAGGATGAGTGCGAAGAGGTTTACTTCTCATTCTTCACGTATGGCAAATGTGTTGGAGATATCTTTGGAAAGTTACCCAAGGATCAGCAACCAGCTTGCAATCTGAGATGTGGAGTTCGTCCCCGTCTATGGACTACTATTGGCCTCATATGTGTGTTTGCTGCGGCTTGTGCAACCATTGTATTCACCATTCCCATGTGCATTGCTACGTGCGCATCTTGTCTCCACGCTAAAAAGGCAAACAAGAATGCTAAACGAGTAGTGATGGAACAGCAAGCCGCTCCCAGTAAGGAGCAACAGGTGGCCACGATGGGATACAACCCCTATGCATATTGTCCTTATTATGGAAGGGCGTAGACAACTAATCTTACAT
>Bg-c29857 len=656 count=2 IPR:/ blastx_SP:/
CTTTTGTTGTGACATGTTAGTTGTTGAGAAGGTGTCTCTTATCTTTGTGGGGGAATTGATCAACAACTTTGATTTTTTTTTTCCTAATCCTGGAAACAAATGTTACCATTACTTTTCTTGAAATTAATAATTTATGTGTGTGTGTGTGCTTCTGTAGACATAGTTGAATCTGTTCCCACACTCACAAAACTTTTGAGCATTTCCTTTTTCAATGTTCCAAACATACCCAGTCCTTCATAGTAGTAAGAAGCTACTATGGAGGATATTATGCTAATTTGCTGTGGCACAAAACTTGCAGCATTTATCTTGAATATTAGAAGTGTTTCAATGTGTCTCTTGTGAACTAGTAGCAGAAGAAATTAGTTCTGTCAGTTTTTTTAGTTATCAAAAACTCAAATTCTGTTTTAGCTGGTTGGCAGGGGGTGTACTTTCTAACACTTGAAAAAAATGGCATTGGAGCTTTGAGATATTCTTGGCTTTGTTGTTTGATCTGACATTTACTTTCTGCTTGACCACAAAACGAGCTGCACTAGTCTTTGAGCATTTTACCCATCTGCTGGCTTCTCCTTTTTTTTTTATACAGGCAAATGTCATTCAACCCAGGCTTTGATCATCCATTTTTTCATTTTCTCTAGCCAGTTAACCTTTCTACTTGC
>Bg-c41942 len=210 count=2 IPR:/ blastx_SP:/
GAACATGTCACTATTCTTTCATCATCCTCCGTGTAACAAATGGAAACGATACATATTTAAAAATACTTATATCAACTCTGTCAGTATGTCTGTCTGTCTTTTTCTGTCTCTGTGTATCTATTTGTCTGTCCAACATTACTAAACGACCACGTTGATCACAGTGTCATATTGTCTTGTTATAAATAATAATACTTGATGATATTTTGTATT
>Bg-c2336 len=649 count=57 IPR:/ blastx_SP:/
TTTCTATTTTCAGATGCCGGACATGGAGCACATGTAAGTATTGAACAGAAAAACAAATTATCTATGGGGAATAACTCCTCCCTTACAATTATATCTGTCAATAATATATAAGCTATTTAGCTATTTCTTTTATTTGATTTCAAACAAAATAATTAATTACCAATAAATAAATGTCTAATTGTTTTAGTTGTACTTGATTCGTGTTTTATTAGGTACCATCAATGATTGTTTAAAAGAAATAGCCTGTATCAGTGATGCCCAAAATACGGCCCGCGGGCCAGATTCGGCCCGCGACGTGCTTCCATACGGCCCATTGAAACGTAAGCACAAAATATAACAAATCCTCCCCCACCTAAAAATGTTGAAAATGTATCTTTACATAAGATAGGGCCCTCAATATTGTTTTAATGTATTGCTACCTAGGCAGAATATAGAATCTACCAGGAAAAGTGAACGGATCTTTACTTTTTTATGGAACATAATAACATGCTAGCCAAAATGTTTGTTTTATAAAAGGTGTGGCTGTTTAAAAAAAAACATTACCAGAACTTACGCCATTTTGTGACGCGTAAAAAGAAAATTCAAATGCCCCCATAATTAATGTAAGTACAAGATACAAAATAGTCCCAGGTCAATTTCATTAAGTTTT
>Bg-c3277 len=617 count=39 IPR:/ blastx_SP:/
TTTGTAATGTTATTTTTTTTTTACCGTATTTTGTATCAAAAACGCCTGCTTTTTAGATTTGTTTTGGAATGTATTTATGAGAACTTTGTCTTTGACAAATATTTTAGTGACCACGCCAATCTACCTTTATTTCTGACTACTCTAATAAAAAAGTAACATGTACTGCTACAAGATTTAATCTTTGTTTGGGCTACACATCCTACCCCATATCTTGAAATGAAATTATATACAACTTTGAAAGCAACAGAGCAACAGTACTAAACAGTTTAACCAACAGTATGCTGCAAAAGGTTGTAAATAATCATGATATTCAATTTAAAAATGTTAATAGTTGGCAAGATACAATTTATATTTATTGTTCACAATTTGTTTTTATACTTATGTGTTTTATTTCCTTGGTGGCTATCTGTGTCAAGATAACTGTTGCATACTTATTTTCAGTCTGTTATCACATCATTTCAAACTATGTACAGTGTTATTAATGTTGTTGTTGTTTTTTTAATCCAGTGTACATAAACATTTGGAACATGAATAAAATCTTCTTTTTTTTTATAGTTACATTCGTTTTACAAAGAATGGTTTTATTCTTATTAAGGATACATAATTGAAGCAGTAGG
>Bg-c41993 len=207 count=2 IPR:/ blastx_SP:sp|Q27650|LYS4_ENTHI/Lysozyme OS=Entamoeba histolytica GN=LYS4 PE=1 SV=2/3e-04/9-152/153-193 // 
ACGCGATGCTCTGGTACTGGAGTGTCTACGGTGCCGGGCCCACCAACGAGTCTCCAGCCAACTTCAACGACTTCCGCTCTTTTGGTGGATGGACAGCACCATCGGTCAAGCAGTTCGGGCAGGTTGAGTCCGTCTGCGGTGTCACCGTCAACAGGGACATCTACACTGTCTCCGGAGCTGCGAAACTCGCCGGAATGGCCAAGTACG
>Bg-c5420 len=543 count=22 IPR:IPR011759:Cytochrome C oxidase subunit II, transmembrane domain; IPR008972:Cupredoxin; IPR002429:Cytochrome c oxidase subunit II C-terminal blastx_SP:sp|P24894|COX2_CAEEL/Cytochrome c oxidase subunit 2 OS=Caenorhabditis elegans GN=cox-2 PE=3 SV=2/4e-55/3-539/7-184 // sp|P24882|COX2_ASCSU/Cytochrome c oxidase subunit 2 OS=Ascaris suum GN=COII PE=3 SV=2/4e-55/6-542/8-185 // sp|Q8SEM9|COX2_CAERE/Cytochrome c oxidase subunit 2 (Fragment) OS=Caenorhabditis remanei GN=cox-2 PE=3 SV=1/7e-55/3-539/6-183 // sp|Q8HEC3|COX2_CAEBR/Cytochrome c oxidase subunit 2 OS=Caenorhabditis briggsae GN=cox-2 PE=3 SV=1/7e-55/3-539/7-184 // sp|O79417|COX2_BRALA/Cytochrome c oxidase subunit 2 OS=Branchiostoma lanceolatum GN=COII PE=3 SV=1/3e-16/276-533/95-179 // 
AAGGTTATAATTTGATGTTTTCAAATAGTCTTTTTTCTAGTTATGTAGATTGATTTCATAATTTTAATTGTAGTTTGTTATTAGGTGTTTTAACGTTTGTAGTTTTTTTATTATTTTCTTTGGTTTTGAGTGATTATTATTTTAAGAGTAAGAAAATTGAGTATCAATTTGGTGAATTATTATGTAGTGTTTTCCCAACTTTGATTTTAATAATACAGATAATCCCTTCTTTAAGTTTACTTTATTATTATGGTTTAATAAATCTTGATAGTAATTTAACGGTTAAAGTTACTGGTCATCAGTGATATTGAAGTTATGAATTTAGTGATATTCCAGGTTTAGAATTTGATTCCTATATGAAGTCTCTTGATAGTTTAGATTTAGGTGAACCCCGTTTGTTAGAAGTTGATAATCGTTGTGTAGTACCTTGTGATACTAATATTCGTTTTTGTATTACTTCTGCCGATGTAATTCATGCTTGAGCTCTTCCTACTTTTTCTTATTAAGTTTAAGATGCTATAAGAGGAATTTTAATTACTTTGT
>Bg-c1094 len=1078 count=118 IPR:/ blastx_SP:/
TCCCTGTCTCAGTTTTACGTTTACACATTTATTTTTATTAATAATATTTCTAAATCAGTTTCATTATCCAAAGAAGGCAGCTATGTCTCCGTTAGTTAAATCGTTTGCTAGAATTGGATCACCGGAAGTACACGGTGTGGATGTCAAGGCTGCAACCAAAATGAGTTCATCTGTGTCAAACGATCGGCAGTAAATCGGAGTGCCAAAGTCTCCCCCACACGAAGAATCGTCGGATCCCAGACACAAAACATCCGAGTCGGAGTTGACCATGGTGTTAGGGTAGGTGCCGTCAAGTTGGACGTGGCGTGTAACGGCGTCCATCAGAACGTCACAGCATGGGGCAGTAGTCATGTTCTGCACTACCAACTTGTATGGCGTCCCAGTGTAGGAATAGTCACCCTTCGTCCTGCTGCCGTAGCCGACTCCAAAACAATCATTGAAATTGACGCGTCCGGCCATCTTGGCAGAGTCATATGTGCACGCGTACTCTGGGCAGTCTGTCACGCTCACTTCCTGTGGCAGTGCTATCTCCGCCATACCATTGGTCAAGTTTTTATATGTGAATAAATCTTGGGTCAAAATTATATCTTGCTGCTTCCCAATTGTGACGATTTGAGTAATAGGCTGTGATTTGAGAGTTTCCTCGTAGAAAATGGCACAATCTTGTGGCACAATGAATGTCGACTTTGACGTGAGGACTCCGGTGCAAAGTATTGTGCTTGTTTCATTATCCCGAATTGCTACGACGCCACCAGCATCGCACGCACCAATGGCTACACCGTTGAGTGTTCGTCGTGTCATTGTTGACACTGCTTGCGTATTGGACGGGCACATTGGAGATGGCGTGGTAGTTTCTTCAGGTGTTGTTGTCTCTTCGGGGGTTGTTGTTTCTTCGGGTAGAGTTGTCTCTTCGGGTGTTGTTGTCTCTTCGGGTGTTGTTGTTTCTTCAGGTGTTGTTGTTTCTTCAAGTGTTGTTGTCTCTTCGGGGGTTGTTGTTTCTTCAGTTGTTGTTGTCTCTTCGGGTGTTGTTGTTTCTTCAGTTGTTGTTGTCTCTTCAAGTGTTGTTGTCTCTTCGGGT
>Bg-c13365 len=534 count=7 IPR:/ blastx_SP:/
ATAAGCTGACAATAACTGGTTGTCAGCTTTCAAAAGAGTTGCAATAATCGATTTGAAAATGCAATCGACAACATTATTCTGGCATGGTTTACTCGCAGACCTTAATAAAACATACAAGAGGGCGTTATCGAGTATAAACTGAGAGGCAGGATCATCACACAATGCCAGACGTAAAGTATAATATTCTGACTCTGTACATAACCCTATATCGACCAAACACTGATGGGCACTAACACCATTAAAATTTATTTTTAACAGGTAACAGTAGTCTTTTATTTTGTACAAATATTGAGCCAATGGCGATATTGCGACACAAGTTTCTACAGAATGTATACACATCGATGACACTACCGAAAAAGCATCGTTATCCTCGCAAGCTTCTTTATATAATAACCGAAACTCAAAGTCATTGCACTTGTTAGAACCAACCAAACACGACATAGCAGTGTTCGAATTTATAGGTTCACAGTAATCTTGGTTACGTATTGAGGAAAAAGCTGTCTGATTCAAAACCGTACAGTTCTCAATAGCCGA
>Bg-c26376 len=295 count=3 IPR:/ blastx_SP:/
CACAAAGGGATGTTACCAGAGGAGTTAAAAAGTTACCTACTGCTGCTGCCTGTGTTATTTGAATGTATTACTTATTACAATAGCTAAAAGACCCAGTATTTATCTACAACTTGTAAACAATGTCTTTAAAATACTGTTGCAATGTGATATGGCTGATAGTCACTCTTGCTATTTTAAAAATTTACATGTGATATAATAGTATGTACATTTGATTGTGATTCAAAAGTATTTGAATTATTGTCACCTGTTATATAGGTGTGAGTTGTTCTAATTAAAAGGCGTTTTTAATAAAAGC
>Bg-c32332 len=368 count=2 IPR:/ blastx_SP:/
ACCAGATACAAAGTCCAAAACCTGGTACAAAGCCCAAAACCTAGTCAAAGCCCAAAACTTGGTATAAAGTCCTTAAACTTAACACGGTTTAACACTGAAACCAGAACGTAGCCCCTAGTAGAACAAACGTCAGAGATCTAGCGATGTCGCTAATCTAAAAAGTCTAACAATGTCGCGCCTCTTACCCTATCGAGTCTAGTTAGGACCATATGTGAACTTACCCTTTTAGCAAGGGAGAAAACTGCGTCAGAACGTGAACAACAACATTTTGAAAAAACAAAACCACGCAACCTTCCTTCAAAACCAGTCCTCACATCGGGAACCAGCCTCACCACTTGTTCTCCGAAAATGTTTTGAAACCCATTCGG
>Bg-c19194 len=728 count=4 IPR:/ blastx_SP:/
TTCAAGTCAGGAATCGTTACACTGACAGCTCAACTTAAGTATTACATTTAGGGAGTGCAGAATGGTTTGTCAAATCAGTACTAACACACTTGGTGACCAGTATAATCTGATGCCATTACCATCTCATGTTTATTTATCGAAAGGCTAAGAAGATTTCATGTGTTGGTTCATTGACAGGTTGTACTACTTCTTCAGTTGACTCACGTACCTCCTCAAAGTGTTTTTTTAAATAATTCTTATGAAAATACTTCACTTCTTATCTTGCATTAAAATGGGCTCCACCAAATAGTTTGGCATGATAGGTTCAGATGATGGTCATCCTACTTGCCTTGAATTGTAATTGACATGATGTGTGTATAGTGCACTTGAGATAACTACTAGTCAGTATTTAAGGAACTATTTCAACATCTTTTGACCATTCACAATAACTTTTGTTCAGAAACAGTTTTAAAAAGTTGACAATACAAATCTTGAAATGTAACTGTTGGCAGTTTGAAGTATCCCCTATGATGTTTAGTGAAGCATCTTTACTTAATTTTTGTTTTTATTCCAAAACTGTATTGAGATATTGAACTCAATGTTTGTATACAACAAACTATCGAACTGGATCTTTCTGACTATTAGGATGATACTTTTAGATGAAGTTTTTTCACTTGGTTATCAATCAAAAAATTTAATCATTTGGGGTAAAGGGTAGAGTTACCCTTCTCAACCCTGCAATCTGTAGG
>Bg-c4127 len=658 count=30 IPR:/ blastx_SP:/
TTCAATATCTTTGCAAACAAAATATTTATTTAATTTAACCAAAATTCTATGACCTTTGACTTCTGTCAGAAAAGTTTTGAGGTGATGAATGTAGTAATTGGTTTTGTACTTCTCACATTTAATTAAATTCAGTGATATTTACATTTTTTTTTTATTAATCATGAAACATTTATATGTACACAATTACTGTTTAATCAAAAGCATCTGAATAACAACATTGATACACTTTTCTAACATCAATATGTTGGCCCCTCATCATCATCTTCATCATCATCACCATATTCTTCACCATTGTCAAAATAGTCCAGGTTATAATCTGTTTCCTCTTCAACATCTTCATCATCTACTTCTTCCTCCTCCTCTTCTTCTCCCTCTTCTTTTTCTTTCTTCTTATCTTCTTCATCCCCATCATGTCCTTCAACCTCTTCCATGTTTACTGACTTTGCATCTGTATCTTCTAATTTCTCCAATAATTGAGAAACATCCTCAACTTGAGCTACTTTAGCTTTTTTAATGTTAGGTTTTACAAAATGTACTGCTCTCTTCCGCTTGACTTTCTCTTTCAGTTCACTAGGAAATCTTGTCCAATCTGGCATCCAATTAGTAGAAGAATCCTCCAGTCTGTCTCCCCCGCGTCGGATGCGTTGTTACCACTGCT
>Bg-c40496 len=240 count=2 IPR:/ blastx_SP:/
CTGAATGAGTACGGACGTTTCGAGAGAAACAACATTGAGATCTGATGTAAGTGTCTAACACACTGACCCAGACAAAAACAAGAAGTGACCTACAGCTTAGCAAGTATAGGGTCAGATTTATGGTTCGCAATGATGACCCCATGGATGTCAGTTCTGCTACAAGTGACCTGGCTAAAGTTCAACATATTGTCTGCACTGCTATCGCTGAGGCCTGGGCTACACTCTACCTAGTTTTTATCT
>Bg-c20159 len=434 count=4 IPR:/ blastx_SP:/
GACTGTGTGTAGCTAACTGCAGTATGAGTTTGATTACTATTCAGCCTGATGGCCTAGTCGTTTTGGAAATGATGGGGGACCATGGACATATTGAGCCTGAACATATCACATTTCACTAGCAGGAATTTTTTTAATGTATATGCTATAGTATATTCTAACTTTTTATTTTATAGAACATTTGTGATATTAGTTAATAGCAGAATACATAGAATAACCACTACCTATAGTAAATATGCTGCCTGATACTCAGGTTTGACTGCGCTAAAAGACTTGCATTATTTATTCAATAGTAATTTATAATGTGTGTGTGTATTTTAAAAATGTTATCCATTGAGGAGTCACATGAAATTCTGTCATATTCATCATCAAGTATTTTTTTTAACTAACCATGTATTAAAACGTTTAAAATAGTAAAAACTTTTAATTAAAGTTTA
>Bg-c37555 len=265 count=2 IPR:/ blastx_SP:/
GTTTTAGTTTATTGCATCATTAACGCGCGATTCGTACACAATAAAATTTTGTTATAAAGAATGAGAAACGACGTACATAAAATATAAGAGGGAACTGCTGTGACGACGTGGTGTGCTGTGAGAAGACAAAAGAGAAAGTAAGAGAGTGTATGCAATGAAAAACGTGTGACGACGATCTTTCCACAAAAAAGAGCAATAAAAACAATCCTCGCTAGAATAACACTAGAATGCACGAGTAGGTGGACCGATAAGATGGGTGGAAAGA
>Bg-c32441 len=363 count=2 IPR:/ blastx_SP:/
GACCTCGGCAAAACATTTTCAGCGCTTAGCAAGAATAGTTCAGGAGGGAGTGAAACAAGGAAAACTTTCCAGAAGCTTGAGCTCAAAGATGAGACGTTTGATCAGCCTGGTCTAAATGCTGACTTAGCAGCACAGCTTTCATCTTCAGAAAAGAAAAACTTTCAGCTGTCAGTGGACACGAAGAAGGATAAATCTGTCGCCAAAACAGTTTTCACAAAGGTAACTAAGACAAAATCAGGCAACCAAAGAAGGCCTTTAAAAGATGATCTTAGGCTACCTGTTGACACCAATAAATTAGCATATCTTAAGAAAACTCCAGAAAATGAGAAATCTCAAAAGGTGGACAAATTAACATCACAAGCT
>Bg-c39753 len=247 count=2 IPR:/ blastx_SP:/
CCCGGGACCATAGTAACCATAGGGGACAGACCATAGCGCATCTCGCACGACCAGGCTTTCACTTAACTCATATAAATCTTTTGGCTATCACCATTTACTATGCATACGTCTCTTGCATCAGCCGTCACTTTCTTTTTAAACTGTGCGTGTGTAGTCTCTTGCATCAGCCCTCACTTTCTTTGAAACTGTGGTGTGTAGTCTCTTGCTATCAGTCTTTCACTTTCTTTTGAAACTGAGCGTGTGTAGT
>Bg-c2562 len=1676 count=51 IPR:IPR000504:RNA recognition motif, RNP-1; IPR012677:Nucleotide-binding, alpha-beta plait blastx_SP:sp|Q14151|SAFB2_HUMAN/Scaffold attachment factor B2 OS=Homo sapiens GN=SAFB2 PE=1 SV=1/1e-36/16-411/367-491 // sp|Q80YR5|SAFB2_MOUSE/Scaffold attachment factor B2 OS=Mus musculus GN=Safb2 PE=1 SV=2/2e-36/1-414/404-536 // sp|Q5R452|SAFB1_PONAB/Scaffold attachment factor B1 OS=Pongo abelii GN=SAFB PE=2 SV=1/1e-34/55-411/372-490 // sp|Q15424|SAFB1_HUMAN/Scaffold attachment factor B1 OS=Homo sapiens GN=SAFB PE=1 SV=4/1e-34/55-411/372-490 // sp|O88453|SAFB1_RAT/Scaffold attachment factor B1 OS=Rattus norvegicus GN=Safb PE=1 SV=2/2e-34/1-411/383-512 // 
GCAGCCAGTGATGCTGCAGCCAAATTGTCTGAAAATGCAGAGTCTAAGGATGCTTCAAAGACAACAGCCAGTAAAGATGAAAAAGACACAAAGACTGCAGCCAAGAGTTCAACTGCCAAGGTTGTTAAAAAAGAGGACAAACCTTCTCAGAGTAGTCGCAATCTGTGGGTAAGTGGATTGTCATCTAGCACCAGAGCAACTGATCTAAAAGCACTTTTTAGCAAGCACGGAAAGGTGATAGGTGCCAAGGTAGTTACGAATGCAAGATGTCCTGGCTCTCGATGTTATGGTTTTGTTACCATGGGATCAGCAGATGAGGCAACCAAATGTATTCAACATCTTCACCGCACAGAACTGCATGGTAGAATGATATCTGTAGAAAGGGCAAAAACTGAACCACAAGGTACAAAAGCTAAAGTATTACCCCCTAGCAGCTTAAAATCACCTGTAAAATCATCCCGTCCGCCTCGACGATCAGATTCTAAATTAAAAAGTTCACACAAAGAAGAAGATAAAGATGCCTCTAAAGAACCCGGTAAAGAAACAGATAAAGATAAAAAAGATGAGAAACCTGATGGAACAAGAAGCACTTCTCGTCAAAGGAGCAGAGACAGGAGACGCTCTCCAAGAAGTACTAGTGGCAGACCTGGAGATCATTATCATCACAGATCAAGGGAAAGAAGATCTTCTTTGGAAAGAGCTGGGCTTTCAGATTTGGAAAAAAAAGAGTTAGATGTATTATCATTTGAAAAAATAAAGGCTGAACGTGAACGAGAACGGCTGCGTCGTAAAGAGATGTACTTGCGCCATGAAGAGCGAAGGCGTCAGCTTGATATAGAAAGAGAACAATACAAGCAACAGTTAATAGAAAAGCGCCAGAGAGAAGAAGCTCTGAAGATTGAAAGGGAAAAAAGGCGACTAAGAGAAATGAGAGAAGAAATAGAAAGAGAAAAATTAGAAGCTGAACGGCTTCGACTTGAAACAGAAAGACTTCAAATTGAGCGTGAGCAGGAATCCTACAGAAGAGAACAACAGAGAGTGTTGCAAGATCGTAGAGCTATGAAACGCCCTGGGGAAAGACCAGGTTTAAGAGAGGATGAGTGGGCCACCAAACGACCAATGACTGACAGATATGGTTCTGGAGAAAGAGGGGGTCGGTTTGAACGCAAACCAGAAAGGTTTGAAAGAAGGGAAACAGATGGAGCTAGATTTGATAGGCGTGAAGAGCGACCCAGACCTGAAAGATTCGATGAACGGGAGCGACCTCCGCGTGCTGAGCCAAGAGAGAGAGATTATAGAAGAGGTGAGGAGCACAGACAATTTAGAGAGGATAGGCCTAGGGAGCCAAGATCAAGAGATGATAGGCAAGGCAGAGACTCAGCTAGGGGCCCTCAAAGACATGATTCTCGAGATTGGAAATCTGAAAGAGAGTCTAGCAGAGGATCTGGAGGAGGATCAGGTGCTTGGAATGGTCCAGTTGAGCGTTCAAAGTTGGATTGGGGACAAGGAGTGGGCTCTGGTGGTATGGGTCAAGGTGATGGCTGGGAAGGTGGTGTTGTTATGTCTCAGCCGGGGGGGAAGCTTCAGTATTCAACAAGGTCCAATGATGACTGGTGGTATGGGAGGAGGTCCTAGTTTTTATGCACAGCCAGTCTTTGCAGGTCCTCCTGTCATGA
>Bg-c7627 len=274 count=15 IPR:/ blastx_SP:/
TATTTTTTGTTTTTTTCTTTTTTAGTTTTTTCGGTTTTCGTTTCCGTTCGTTCGGTATTACTGAGTATGTCGGATATAAAATGTCGGACTATCAAGAGTCAACTGTACACATTCATAATCTTAGTGTTATCTAAATATATTAAATACAAACTTTTTACCAGACAGACAGATAGAGTTGATAGAAGCTTTGTAATAACGCAAACCTCGGTATGGCATAAGATTCTCAGGACTTCTGAAGACATTCCTTCAAATAAAATTACAAGAAAATAAGCTA
>Bg-c38267 len=259 count=2 IPR:/ blastx_SP:/
GTATTGTCAGGGTATATTTGGCACGTCTTGTGTGGAAATAATCAGGAATATAGATTGCAGCATCTAGTTATGTTTAGAAAGAAAAATGATAACGACAGAATTAGATTAGAGATGAGTTAAGTACACATAGACTAGGGAGGAAATAGACAAGGAAAAGCATTTCCCTTTGGAGAAAAATAAACATTGATTATTGAGGGGGAAAAAGTTGTGGGACTTTGATGTTTCGTGATCAGCTAGATGAGTGAACTTGTTCACATAG
>Bg-c1073 len=1064 count=120 IPR:IPR013740:Redoxin; IPR012335:Thioredoxin fold blastx_SP:sp|P99029|PRDX5_MOUSE/Peroxiredoxin-5, mitochondrial OS=Mus musculus GN=Prdx5 PE=1 SV=2/3e-26/915-616/109-210 // sp|P99029|PRDX5_MOUSE/Peroxiredoxin-5, mitochondrial OS=Mus musculus GN=Prdx5 PE=1 SV=2/3e-26/1060-929/61-103 // sp|Q9R063|PRDX5_RAT/Peroxiredoxin-5, mitochondrial OS=Rattus norvegicus GN=Prdx5 PE=1 SV=1/6e-26/915-616/112-213 // sp|Q9R063|PRDX5_RAT/Peroxiredoxin-5, mitochondrial OS=Rattus norvegicus GN=Prdx5 PE=1 SV=1/6e-26/1060-929/64-106 // sp|P30044|PRDX5_HUMAN/Peroxiredoxin-5, mitochondrial OS=Homo sapiens GN=PRDX5 PE=1 SV=3/2e-24/885-616/123-214 // 
TGTTTATCAAACTATTTGGAAACTTTCATGGCTGTAGTACCATTCTAAGACATACTTTGGTTTTAAGGTGAAACCTCCCCTTAAGCTTCTAAATTTTACTAATCTTATAAATAGAGCAGAACTGCGCCTACTTTTTTTTTTGTAATTTTAATTGTAAAAAACAGAACTAAATATTTTTTGTTCCAGCTGTTTCATTAAAGATTCTTTTAAAAAAATGTCAAAAGTAATATGTCACTTTTTTAAAATGTCAAACGCATTCAGATGGTCTTTACCCTCATACATTTTATAGACGGTAATCACATTTTTATAGCATTCATATCCCACTTTGCATGATACATTTCATAAATAATACATTAATATTTCACTGAAATAATGATACTTTAATGGAGTATTCTAATTTGTTAGGGCCTGCACAAAATAAAAGCCTGATTCTGGTCTAGATGCACTTTAAACTAGCAATAGAGGACACATGTAAAAAAAATAAAACAGGATGTCGGCAGTGCTATGTGCTTGATAGAGAAGTTGAAACAAAACTGTGTGGACGCCTGTTACAGTTAGTAATTATACATTGAGCTAATAAAACATAAAGACAGAAGATCCTCGATGCCTGTCTCACAGTGAGTTCAGGATGTCACTGGCTCGGCTGCATGTCAGACCAGTGCCATCTGGTTCCAGATTGACTTTCTTGACAACACCTTGATCAACTAAAAGGGCGTATCTCTTGTTGCGAACATTGCCAAGTACTGCAGTTAAGTCCAAAGTCACATCTAAAGCCTTTGCAAACTCAGCACATGTGTCGGCCAAAAAGCGAATCTTGCCGTTAGGATCCAGTGATCGACCCCATGCAGCCATGACAAAGGGATCATTCACCGACACACACACAACCTCTTCACCCCTTTAGCTTTCAAGGAATCCACAGCAGACACGAACCAGGCGCATGGTCCTTGGTGCAAGTAGGAGTGAATGCCCCTGGGACACCAAAAATGAACAACTTTGCCTTGATTGAAACTTGAAAGTTGTTTTAACTTTTTTGTCAGGAGTCTCCCTCGTAGAGGTCTACTACG
>Bg-c26092 len=307 count=3 IPR:/ blastx_SP:/
CAATGTCTACACTTTAAAGCAAAGCTTTCAATATTCGGCACAGACTTCGGGACGTTACAAACAAAATCAAGACGTGACTCGTTACATCTAGACATTTCATATATAGAGCATGAATCAGTGGCGTAGCTAGGGTGGGGGAGGGGGTCCAAATTTGAAATTCCAGCCTTTCCTTCACTTGAGGGGGGCCCCGAAATGAGTGTCCAAATATTTTTTTTTTAACATCAAATATTAAGCAAATGCCATGTTATCTTGCCATTCATGTTGTTATGTAAATTAGTGACCATGTCGGATGCGTTGTTACCACTGC
>Bg-c30725 len=472 count=2 IPR:/ blastx_SP:/
ATTTGAATTTTTGTCTGTTCTCGTATTCCCTATCTTCGTTAGGGGGACCAACACGCGAATCTTCAACTTTTGACTGTTTAAATTCTTTTTTAAAGTATCACCATATTACTAAAGGAAATTAAAAAATGATACATAATCCTTGCGTTGTTTACCTCAGAATCAGAAGAGCAAGTTTAGTTGTAAGTTGTAATAGGGTATCAAACATTGAAATAATGGAGAGTTGTATTTCTGCTAATCAAAGGTAGAACTAGCAATCTGGGACCAAACTATGTTTTTCATGGTAATTTCACATACCATTTTGATAGTGTATTGACTTTGAATTTCCCCAGATGCATTTATTGTAAAGCTCCACAGTGCAATTTAGTAAAAATTATTTTCTGATTTATTTAGATGTCCCTTCCACCACAAACAAAATAACAAATTGAATGCAAGGACAGAACATGGAATGGCAACAATGAAGAGTGTCTACCCA
>Bg-c16125 len=260 count=6 IPR:/ blastx_SP:/
TAGGAAAAACAACACTATAGTTAACATGCACACATTTCAGGATTGAAGCAACATAAAAAAGGGGCAATAAGAATGTTCAGCATGGTTATCGTTCCATTTTTTAATTCAATCAATGAGGGCAAAAAGAAAAAGGTTATTTTTTGAAACAGAAGGCAATATTTTTCTCAAAATCAATTTAAACAGGATTAAAAAACACATATTGTGCTATTACAAATAATCAAGTCATGCATTCATTCCTTTCCAAGCCTTGTTTACAAATA
>Bg-c8641 len=972 count=12 IPR:IPR010041:2,3-diketo-5-methylthio-1-phosphopentane phosphatase; IPR005834:Haloacid dehalogenase-like hydrolase blastx_SP:sp|Q28C69|ENOPH_XENTR/Enolase-phosphatase E1 OS=Xenopus tropicalis GN=enoph1 PE=2 SV=1/8e-46/925-374/69-254 // sp|B5X2D1|ENOPH_SALSA/Enolase-phosphatase E1 OS=Salmo salar GN=enoph1 PE=2 SV=1/4e-44/901-377/81-255 // sp|Q6GMI7|ENOPH_DANRE/Enolase-phosphatase E1 OS=Danio rerio GN=enoph1 PE=2 SV=1/4e-44/895-377/83-255 // sp|Q0VD27|ENOPH_BOVIN/Enolase-phosphatase E1 OS=Bos taurus GN=ENOPH1 PE=2 SV=1/1e-43/925-374/69-256 // sp|Q7Q9C0|ENOPH_ANOGA/Enolase-phosphatase E1 OS=Anopheles gambiae GN=AGAP003331 PE=3 SV=5/5e-42/925-377/75-255 // 
GCCCTTGCCTTTTTTTTATTTGATGTCAAAAAAAAAAAAAAGTTTCATCAATATATTAATTCATGTATCAAGTCTTGCTGTAAAAACAATAGTGGTTCAATGTTGCAAGTTACTTTTATTTTTTGTTTCTTCCCATCAAACATCAAAATGTAAACATTAACAAACAGTTGATGCTCTTTCATTCGTTACATTTACTGAACTTCAGTTGAGACTTCTTATAGGCATCTTCACTAAATACAACTTCTCAAGCATCATCACCTTCTTCCTCTTCTTCCTCATCGTCATCACCCTCTTCCTCCTCCTCATCATCGTCGTCAGCTTCTCCATTGCCACCAGCAAACCTCTTAATATCATCTTCTTCATCTTCATCCCCGTAGAGCTCATCGAATCTTTCTATACAAGCAAAGTTTTGCAGATTTTCATCAGTTAATTCCTCATTGCCAGGTCGAATAACAAGTGCACTGCGTAATCCTGCACCCACGGCTGCTTCTGCCTCTTCAGGAATATCCGTAAGGAATAAAATATCTTTAGGCTCATGTCCTATTTCTGTTGCAATTTTCTTGTAACTGGCCTTATCTCTTTTACTTCCTGTTGTTGTATCAAAGTAACCAGAAAACACATCTGAAAGATCTCCTTGATTGGAATATGAGAATAAAAGCTTCTGTGATTGGATACTAGCTGAAGAAAACACATACAGTTTAAATCCTTCTTCTGCTAGCATTTTCAACATGGGACCAACATCTTCAAACAACTCTCCTTTTATCTCTGAGGATTCAAATGCCTCTTTCCAGATCAATCCCTGTAAAGCTTTCAGTTCAGTGGTTTTTCTGTTTTCACCCATCTGCCATTTCACATTGTCAATGACTGCTTTAATAACATCTTCTTTACTAGCATGTCCACTTGGAATTTCAACGACTCCTTCAACCCCCTAAAGGGATCTTTTGCAGCCTGGTCTCTGAATGCTGCAATAGA
>Bg-c25503 len=338 count=3 IPR:/ blastx_SP:/
TTGTGGAATCGTAACTCACCATTTTTTTGGTTAGTTATAGAGTAACTTTTTTTTTGTTGACATGTGCTAACAATCTTTGCCTAAAAGAAAGTCAGCTTTGCTCAGCCGCTGGGACAGTATACAAAAAAGTTTGCCTGCTTGTATTCCTGCACATAATTAAATAATTTCAGGCTCAGGGTGTTGGTGTCTATTAATCTTGTGACGTAACAAATATGATTGTGGAAAATTAATAACAATAAATTGAGATCAGGTACTAATAAATGGTTGTGAAGGCAAATATATTTTGTTCTTTTTAGTAGTCTAATAAATGAATTGTACTTTTACTACATTAGTTATGT
>Bg-c27297 len=269 count=3 IPR:/ blastx_SP:/
AAGTGAACATAATCGTCTAATCGGCTATTGGATGATTTTTCTCCTAAGCTACTTTAATATCAACTGGCTTGCTGGCATTTACAAACTCAAATTTAATTCTTTCTACACATAATTCTTTGTCTTTGCATTTTAAATAATTAATAAGGCTCAGCTAATATAACATTGAGACACTGTAGGAAGGGACACCCAAACTATATAATCAGAACATTTATTTTTATTTGCACAAATGCACAAATTGTAAGACAAATTTCCTTACGGACAATAAAGAT
>Bg-c14529 len=723 count=6 IPR:IPR003439:ABC transporter-like blastx_SP:sp|Q767L0|ABCF1_PIG/ATP-binding cassette sub-family F member 1 OS=Sus scrofa GN=ABCF1 PE=3 SV=1/2e-84/565-8/349-534 // sp|Q767L0|ABCF1_PIG/ATP-binding cassette sub-family F member 1 OS=Sus scrofa GN=ABCF1 PE=3 SV=1/2e-84/692-567/307-348 // sp|Q767L0|ABCF1_PIG/ATP-binding cassette sub-family F member 1 OS=Sus scrofa GN=ABCF1 PE=3 SV=1/2e-08/433-110/684-791 // sp|Q8NE71|ABCF1_HUMAN/ATP-binding cassette sub-family F member 1 OS=Homo sapiens GN=ABCF1 PE=1 SV=2/5e-84/565-8/387-572 // sp|Q8NE71|ABCF1_HUMAN/ATP-binding cassette sub-family F member 1 OS=Homo sapiens GN=ABCF1 PE=1 SV=2/5e-84/692-567/345-386 // 
CCTTGGCTTCTGCTGTCTTGGTGGACTTGCCAGATGCTTTTAAATCTTTAATTTTTTTCTCTTGCTTTTCAAAGTCACGCAACTGTTCTTTTCTTTTCTGTACAAACATCTTTTTGAAAGTAGCATAGTTTCCCCTGTAGTAGAAAAGCTTCTGCTGGTCAAGGTGAATAATATCTGTGCAGACATTGTCTAAAAAGCTTTGATCGTGAGATACAACTAGTAATGTCTTCTTCCACTGCTGGAGATAATTATCTAACCAAATAACAGCATTGAGATCGAGATGGTTGGTAGGCTCATCAAGCATAAGTAATGTGGGCTCCATGAACAAAGCTCTGGCCAGAGAGACTCTCATTCTCCAACCACCAGACAAGTCTTTAGTGGCTCTGTTCATCATCTCTTTAGTAAAACCCAGACCAGCCAAGATTCTTCTTGCCTTTGGTTCAGCAGCATCAGCATTGATTGCTCTCAATTCTTCATAAACATCTTTCAATCGATCAGTAATCTCTCGCCCCCCTTCTGTTATTTTAGACATCAACTCTTTCTCCTCCTGTAGTAGTGCTGTTCTCTTTTTGTTCTGCTTTAAGGACAGCATCAATAGCTTTAGTATCATCAGCGGAAACTTCTTGCTCACAGTACAGAACATCAATGTTGGCAGAAATACCTAAAGCTCGGCTAGCCATATGCTTCAACAAACAAAGGGGCCATGGCCATTGGGTCCAACCA
>Bg-c37556 len=265 count=2 IPR:/ blastx_SP:/
GCCAAATAATTGACCTGAAAAACTAGGGTCCTGCGATGTAAAGTTTTACCCTATGTCATATATGTCAATTGTCATATGATTAACTTAAGACATTTATATGATTTAAGACAGAGAATTCAAATTCCATTGACAAAGGCTTCTAAAGAACTAACTCACTCAATATTATGATGGAAGACAAGGTACAAGACTTAACTAATGATCTCAGAAAGGCCATGAAAATTGAGAATATAGAATGGTCAGAGACACCAGAGAACAGCTTCACTCG
>Bg-c30648 len=481 count=2 IPR:/ blastx_SP:/
TTTTTAATAAATTGTTATTAAGTTAACATAGTAAGANGACTATTTTTATTATTTCATATTTCAGTTTCTAGTCCTACAGGAATGATTGAATCATACTCTGGTGTATTTGATCACATCATTAGAGTAGTGGAGCCAGTGTTCATTGGACGACTACACAGTGCAGTGGTTATGTGGAGGAACAGGAATATATCACTGCCTACTGATATGAGGTACCATTTACTTGTCTTAGACATTGTGGAATTTTGAATACAGAGCAAATACACTAAACATTCTTTCTTCATTGTTGTCATTTCATATCATTTCAAAACTATGTGACTAAAAAAATTAACATACTTTAGTTGTTTTAGTTTTCTAAAAAAGACTTACTATTTTGTATAAAAAAAAGAAAGCNCACAGAAGCCTTTCGTAAGTAATTGGTAAGTAAAGTTACTATCAATTTAAGTACCTAACGTTAAAAAATTAAACAATTATTTTGGGTCGG
>Bg-c8556 len=300 count=13 IPR:/ blastx_SP:/
TTCTTTAACTTCTTTTCTTCTTCCGTTAATTTATTTTATTCACATTCTGGTGGAATCAACGTTGGTATCATCAGTTAGGAGAGAAAGAGTTGAAGAATGCTTAGATTTTTTTCATATTAAAAAATATACTACAAGTAAGACCTACAAAGTACAAGTAAGACCTGCAAAGTACAAGTAAGACCTGCAAAGTACAAGTAAGACCTGCAAAGTACAAGTAAGACCTACAAAGTACAAGTAAGACCTACAAAGTACAGGTTTGATGCAAAGTAGAAAGAGAAATAAAACAATTATGGTAACTAA
>Bg-c9893 len=228 count=11 IPR:/ blastx_SP:/
TTTAAAGATTAACATTCTTTTAATCAGACCATTGTTAAAACTATATAGAGACAACATTTGTCACTGGGTGTATCTAACTGGGTTACACAAGGCAACAACAGGAGAACATGACAAGTAAAAACATTACTACAATGTTAAAATAATGGACTGATTGATGTACATTGTGTGCTGTCCACATAATGCTATATTACATTAACTCACATAATCAATAAAACACTTGGCCATTTG
>Bg-c33358 len=329 count=2 IPR:/ blastx_SP:/
ATCAGTTCCTCTTCTTCGTTCTTATTTTACATGTTGAATGGTTCAAATCAGTAATCCTATATCTGAGATGAACCGCGTAGTGGTTTCCAGATCAGGCAGTTCTCCGAATAGTCTTTGGAGAGTCTTGTTCGGGTCTCTTGATTTAGTATGCACCATTGAAGGACATGGTCAGCATTCTCTGGTGATTCTCCACACGGGCAGGCTTCGCTCGTCCCGACATTTAACTTCCGGAACATAAATTGTCTCATTCAGTGAGCAAGCATTTTTTTGTGGAAACTAAACAAGTATAATCACTGGACAATGTTGACTGACTGTAATTTGACAGCAGT
>Bg-c5245 len=441 count=23 IPR:/ blastx_SP:/
TGTAATTCCAATTTATTTAATGCTTTCATATATGCATGTGTGTTCATATATAGGTACGGTACCGGTATTACAAATTTAGAAACTAGGAATAGTGCTGGCTTATTGAAGTAAAGGATATCTGTAAGAAATCTTTTATTCAACGAGTCCGAGACTTCATACCTACTCAGGATATTAGGATGTCTTGGAATAGAATAGTCATAATGTCTGCCTATTGTGCTGTTTACACAGTACTGGTTACTTTTTTTTCCCCCTTTTTGTTGGTCACATAAAGGTAATACACCTGCTTATTGTATTGAAAATATGAATTTTACTCAGAAACTTCCTGATCAACCTTATTTCTTCACTAGCTTTAAAACACTTTAAATAATATTACTTTTGTCTAAATAAGGGTTTAGGATACAGCAAAGAATATTTTTGCAAAATGGTGTTTATGAATAATTT
>Bg-c22835 len=1358 count=3 IPR:IPR014021:Helicase, superfamily 1/2, ATP-binding domain; IPR014014:RNA helicase, DEAD-box type, Q motif; IPR011545:DNA/RNA helicase, DEAD/DEAH box type, N-terminal blastx_SP:sp|Q9BUQ8|DDX23_HUMAN/Probable ATP-dependent RNA helicase DDX23 OS=Homo sapiens GN=DDX23 PE=1 SV=3/3e-118/1241-3/122-524 // sp|Q5RC67|DDX23_PONAB/Probable ATP-dependent RNA helicase DDX23 OS=Pongo abelii GN=DDX23 PE=2 SV=1/7e-118/1241-3/122-524 // sp|A1CX72|PRP28_NEOFI/Pre-mRNA-splicing ATP-dependent RNA helicase prp28 OS=Neosartorya fischeri (strain ATCC 1020 / DSM 3700 / FGSC A1164 / NRRL 181) GN=prp28 PE=3 SV=1/1e-73/770-3/241-498 // sp|Q4WPE9|PRP28_ASPFU/Pre-mRNA-splicing ATP-dependent RNA helicase prp28 OS=Aspergillus fumigatus GN=prp28 PE=3 SV=1/1e-73/770-3/241-498 // sp|A1CHL3|PRP28_ASPCL/Pre-mRNA-splicing ATP-dependent RNA helicase prp28 OS=Aspergillus clavatus GN=prp28 PE=3 SV=1/4e-73/827-3/219-500 // 
GTGGCAATGACTATCTCGCAGCCTTGTCTCAACTTGCCATTAAGGGGTTCACGAGATATACCACCAATGATTGATACTGTTTGGATGCCCAGATTTTTACCAAACTTAATGGACTCTTCTTCAATCTGCTGTGCCAACTCACGAGTGGGTGCAAGAATAATGGCATATGGACCTTGGTCAGCACCTTCCATTCTTTCTATTTTGGGAAGTCCCATAATCCATTTCAACAAGGGTATAAGAAAAGCTGCAGTTTTACCACTGCCAGTTTCAGCTACACCAATAATATCTCGATTCTGAAGACCTATAGGAATAGCTTGTCTTTGAATAGGTGTGGGTTCCTTATATCCAATTTCATCAATTATATCCAACAATTCTTTAGGAAGATCTGCTTCTTTCCAGAACCTTATTGGATTAGGAATTCTACCACCTTTGCAACTAATGTTGAAATCTTCTTTGAAAATACGCCAATCTCTCTCAACCATGTCCTCAAGAGGTTTCTCTGACCAGTGACGATCATCCCAGCGTTGTTTGGCTTCCCTAGAAGCCACACCTTTTAATCGTTTCTTTTCCTGATCTTTCTCTGCCTCTGTTCTTCGTTTTTCCATGAGATCTCCATAAAACTTAAACTGATCTTTCTTTTGAGCCTTTATGTCAATCCCAGCAATATTGCCTCGACCGAAAAACTGGACCTGATGTTTCTCTTTGTAAAGCTGGTTATAGTCATTTGAAGTGTCATCACCAGCATCCCAGTCAAACACAAACTTCCTATCATTCAGACGTCTTATCCTCTTGCGCTTTTTGATAATTCCCAGGTATCTTTCTCTGATTGCTTCAGATTCTTTGTCTTTGTCTTTCTCCTTCACTTCTTTCACTTTACTCTTATCTTCTCCAGGAACATCTTCTCTTTCTTTTTCTCTTCGCATTTGTCTGTCTCTTCGTTCTCTTTCTCGATACATATCTCTCTCACCTCTGTCACGATCATCAGAAAACTCTTTCGCTGTTTTTAAAAACTCAAGCTGCTTGTTTCTTTCTTCCTCCATTTTTCTTCTGGCTTCATCAGCAGCTTCTTGGCGCTTCCTTAAAGCCTCGGCAATACGCTCTTCTTTGGTTAAAAATTTGGGCCTACTAAGGGCTGCTTCTTCTGCCTTTTTCTTGGTTAGTAGCTCTTCTAAAGATAAAGGTACCTTTTTCTCTGTTCTTTTGTCTTTGTTAAATTCTTCAGCTTCTTCAGCAGGATCTTTTTTGCCATCTTTTAATTTTGGAGAACGAGAGCGTTTTCTCTTGCTTCTCTCCCTTTCCTTACTTGTACTCCTTAAAAAGATCCGCTCTTTACTACGACTGCGTTTCTTTCCCTGCTT
>Bg-c23532 len=547 count=3 IPR:/ blastx_SP:/
TGACTCTTGGACTTGAAATTCAACAAGAAACCAGTCCATAAGTCCACCATGATGTCTTAGGAATGTGACAGTTTCGTGAAATGAAATGGCAGTCCTCCCATGAAACTCAAACACAATGAATTCCTATAGGTTTATGAACTTTGATTTTCGGATGCTTAAAGATAGGAGTAAAAAGAACACTTCACTAAAAAAAAAGAATGATCCAAACAACAAATGTCTTTATCACAGACAACCACAAAGCAAAGATTCTGTTTCTTTCTTGACCATTTGGAGTCACGTGGCATCAAGTATTAGGGAGTCTGTCCCATGACTAAACACCTAAATGGAATTAAGTGAAGGCTCCCCGCAGGAATTTTCAGCTTTAAGAGTAGCTAATCATCTTTGTATATTGAGTACTATGTCCAAAAAATTAGTCTTAGGAAACCATAATCTGGCAAGTAGCAATGAGTCATAAAAAGAAAAAAAATTAGAAACGATTAGCTAAAAAAATATCTAAGAAAAATTAAAACGTTTAACTGGAATACAAAGCAATGGCTAGTGAATGCTA
>Bg-c30955 len=446 count=2 IPR:/ blastx_SP:/
TCGAGCCCCCTTCTAGGTAGCCAAGTTCAAGCGCACTTGGTCTCTCGCCCACGCTTCCCATGACTCTCTGACACAATGAGTTGTAAAAAACAAACAAACAGGTTGTATTTGTAATGTATTGTAAAATGTATCTATTATTGTATTTATACTTAAGAAACTTTTCATTCCTTATTTGAAAATAGACTAGTTACATTTGCTATGTAGACAAAGATATTTGTGGAGCCAACCTTTGCCTTGAAAACTTTATTACAATGAAGTTTGTTTAGGTTGGGCCTGAGTTAGCTTTTTGTATATAATTTCCAAGTTAAACCTGACCTCATTATAATATTCTTACATATGAACTAGTTTAAAAAGACATCAGTTTTTGTTCATTGTATTTTGTTACACCCTATCACACACCAACATTGACATTTGTTTTTTGACAAATAAAAATGTAATTACTTAAA
>Bg-c29744 len=678 count=2 IPR:/ blastx_SP:/
AAACATATTATATTATTAAACATCTAAATTTCCTCCTATTGCTATGTGTGGGGTTGTGCCATAATGGATAGTTTTCACATAGTCAATTCTTTTGGATATTACTTTTACTATATCACAATAAACAATACTATTTTGTAAGTGCAATGTGGGATGTTTCATCTATGCAATATATAAATGATAGAGTTAATTTCAATTTCCTTATAAGTTCAAATTTAATAAATTATTAAAGTCTAAATTTACCTCCAGCTGTGTGTGTTTGTTTTTATTATAAAATTGATATAATTTCCTTTAATATAAGTGCAAGAATGTAGCAGTTCAAATAATTTTGAGATGATAATATTATCTACTGCTTGCAAATTGATATCTTTAGACATTGGCAAGGCATTTTTTTGTTTATTCTTGTCATCTGTTGTGAGCTTTGTAGATACTTGCATCATCAGAGAAAAAAAAGTTCCTGTCTTGATACATTTGATTAACATCACTTGATCTTCTTTGTTAATCTCCCACTTCAATTGTTTTCTTTTTCAGGTTTTACCGTCTTTAGAATGCACATTTAACTTCATTGTATGTTGTATTAATTCCAAATGTCTGCAATAGTTTAGTGTTTTCCTGAAGTCAATTTTCTTTGTCTTTTTCAGTGATCTCGAAAGTAACAACAAATTTTTTATGAGTACATTC
>Bg-c10188 len=556 count=10 IPR:IPR007081:RNA polymerase Rpb1, domain 5 blastx_SP:sp|Q5ZL98|RPC1_CHICK/DNA-directed RNA polymerase III subunit RPC1 OS=Gallus gallus GN=POLR3A PE=2 SV=1/2e-54/28-555/1168-1345 // sp|O14802|RPC1_HUMAN/DNA-directed RNA polymerase III subunit RPC1 OS=Homo sapiens GN=POLR3A PE=1 SV=2/1e-53/28-555/1168-1345 // sp|A4IF62|RPC1_BOVIN/DNA-directed RNA polymerase III subunit RPC1 OS=Bos taurus GN=POLR3A PE=2 SV=1/1e-53/28-555/1168-1345 // sp|P04051|RPC1_YEAST/DNA-directed RNA polymerase III subunit RPC1 OS=Saccharomyces cerevisiae GN=RPO31 PE=1 SV=1/9e-41/46-555/1245-1413 // sp|Q86AQ5|RPC1_DICDI/DNA-directed RNA polymerase III subunit rpc1 OS=Dictyostelium discoideum GN=polr3a PE=3 SV=1/6e-40/76-555/1256-1415 // 
AAGTGAATTACCCTGATATTAAGATTTCACAGTGAGGCTGTGTTCTCTGTCTCCGCCAATGCCTCATCTAAAGTTTCAATGTACTACAGGCTGCAAGCACTTAAGAAATCGCTGCCAGATATCACAGTCAGGGGAATCCCCTCTGTGACAAGGGCAGTAATTCACAAGAGTGACAAGTCCGGGAAATACAAACTGCTTATTGAGGGGGATAACCTCATTGGGGTCATGGGAACACAGGGCGTTCTGGGGGCAAAGTGCAAGTCCAACAACACCCACGAAGTCTGGCAAACTCTAGGCATTGAGGCCGCTCGAAGCACCATCATGAAGGAGGTCACTGTCACCATGAAGAGCCACGGCATGAGCATCGACGCCAGACACACCATGCTCCTTGCTGATTTGATGACCTACCGTGGCGAGGTGCTAGGAATCACCCGCTACGGCCTGGCCAAGATGAAAGAGTCTGTCCTCATGCTGGCCTCCTTTGAGAAACAGCTGAGCATCTCTTCCAGGCAGCTTATTTCGGCCAGCGTAGACGCCATCTGTGGTGTCAGTGAGT
>Bg-c6734 len=520 count=17 IPR:/ blastx_SP:/
CTTTTGTCAGACTAGTATAAATATTACAGGCCCTGAGCCTAGCAAGTAGTCCCAGAGAAGAACCTGTTACAGACAGAATATTTTCTGACTGTGCCAAGCTTGGTGCCTGTGAGGTCAAGATGACCAGGATTGTCCTACTACTGCTCGTGTCTCAGCTTGTCTTGCAGTGCTTGTCCTCGGGTGGACATGATACTGGGCATGGGGGGCACGAAGCACCTCAGGCAGGTGGACACGGAGATTCTCACAGCACAGGACATGGAGATGCCCATGGTGGCGGACATGGCGACCGCCATGGTGGCGGACATGGCGACGCCCATGGCGGTGGACATGGCGACGCCCATGGCGGTGGACATGGTGGAGACATCCACGGTGGGGTACACGGTGACCCCCATGGAGGAGGAGGACATGGTGGAGGCCATCACGGACCACCTCATCCGGGATACTCNAGTGCCTCTGTTTTAGGATTGAGCTCAATTCTTCTAGCATCTGTANTTTTATTGAAACATTTGATTTAAACTGT
>Bg-c41544 len=224 count=2 IPR:/ blastx_SP:/
CAGTCAAAGCTGTAGCCGAGTCTAATATGGACGACGTCCAAGCTTACGAAGAGTCAGAAATTGCCGATGGCTACTCCAGCGAAGAAGAAGCATCCTCTCAAAGCACAACTTCAACCCCAACTCAAATCAGTGATGCGTCTAGTTTGCCTTCCGAGAACGATAGCTCGCCCACCATCTTGGAAGCCGTGACCCCCGGAAAANATGCATTCGACTCTTCAGTAGAC
>Bg-c35655 len=284 count=2 IPR:/ blastx_SP:/
AGTCCATTTCATCGTTATTGTTTTTTAAGACGTTGATTACGTCATGAATGGTCTAACTATTCATCCATCTCGCTCGTGCCTTTACTTGCTGGACAGCAGAGTATACATTTGAATTGACAGTTTTGTTTCGTCAAACTTTTAAATTCATGCTTTTACTTGTTTAGGCGGAAGTTTAAAATAACTTCCGCTTTTGTCTCCACTGAGATCGATGTTTCTAACTTCCTGTGTGTTCGCGCTTAAAGTTCACAAGAGTATTTAAGTTTGGAAATAAATAACTTATTTTA
>Bg-c27897 len=256 count=3 IPR:/ blastx_SP:/
CCGACTTGTGAAGAGAGTTGAAAGACCAGAAAGTGAGCATTTGGAGCTATTCTTGACTTTATTGCGGTTCAAAAATATTTGCCGGTGCGCATGCTTTGCATTACGTCTAAAATAAAATACACATAGGCTAGACCTTATTATATACACGCCATGACGGCCTTACAATTATCTCTGGTCTGTTGTTTGTGCCTGGCACTGACGTATGGATATCCCAATTACTCATCGTTTATACCCAATGGAAACAGAGTCTTTCATC
>Bg-c39001 len=253 count=2 IPR:/ blastx_SP:/
TTTGTGTGTGGAAAAGTGTTTCCAAATGTGTAGACAAATGTGTATGATGAGTATTCTAAGCTGAATTAGCTTTTAAATGCAACATATTTCTTCAGCTACAATTTTATTTTTCTAATGAAGGTGAGGATGAGAGAGAGGCCAGTTCAACAAGTCCACCACACTATTTTCAAAGTTTCTGGAGGTAACAGAATAGGAATATGAAGCAATTCCCCATGATTATAGTGATTTATATCTGATTTTACTATATTACTAG
>Bg-c10551 len=323 count=10 IPR:/ blastx_SP:/
TGGGCCCCGCACTTTCATAGGCCCCGCGCGATGCGAATTCTATGTGTAAATTATTAAATTAAACTATTTTAACATATTAAAGGGTTCCTGGAATTATCCTGAAATTTTAAAATATAAGAAAAAGTCATGAAAATCTCCTGAAATAAATAAAATCTTCTGAAAACTGATGCAAATCTCCTTAAAACAGACAAAATTGTCATTTCGGGGTGTCATTCTATATGGAAGACGCCAATCCTACTCGCGATTTAAAAAAAAAAAAACGAAACNGCTTTGTCAGCTTTTATTTAAATAAAAAATGTAATAATAAGCCGAATTTAACCAAA
>Bg-c28119 len=251 count=3 IPR:/ blastx_SP:sp|P36916|GNL1_MOUSE/Guanine nucleotide-binding protein-like 1 OS=Mus musculus GN=Gnl1 PE=1 SV=3/2e-05/172-249/355-380 // sp|Q6MG06|GNL1_RAT/Guanine nucleotide-binding protein-like 1 OS=Rattus norvegicus GN=Gnl1 PE=2 SV=1/3e-05/172-249/355-380 // sp|Q5RA07|GNL1_PONAB/Guanine nucleotide-binding protein-like 1 OS=Pongo abelii GN=GNL1 PE=2 SV=1/3e-05/172-249/355-380 // sp|Q7YR35|GNL1_PANTR/Guanine nucleotide-binding protein-like 1 OS=Pan troglodytes GN=GNL1 PE=3 SV=3/3e-05/172-249/355-380 // sp|Q5TM59|GNL1_MACMU/Guanine nucleotide-binding protein-like 1 OS=Macaca mulatta GN=GNL1 PE=3 SV=1/3e-05/172-249/355-380 // 
CGCATCCGACGCGGGGGGAAAAAAACAGTAGCTGAGGATGCTTCTTATATACCTGGAGATGATGATGAGGATGACGAGGATGAAAGTGATAAGTCTGATGAAGCAGCTAGCAACAATGATGCTGAGCAAGAAGAGTGGGATAGAGAACGGTTTGAAAATGCTGCACATGCCAAATACAAGAATGGAATAGTGACAATAGGATGTCTAGGTTATCCTAATGTTGGCAAATCTTCAGTGCTAAATGCTCTAAT
>Bg-c19108 len=814 count=4 IPR:/ blastx_SP:/
ATATTCCCGTGGAATTTCACCCTTAAAGGGAATCTGTTTTTGAAAAGTGTTGTCTTAATTATGGCTTTAATTATACACAAATCTTGGAGTTAGAAAACGATACAATTAGTTTAGCCAGTTTTGGCAATGAAATGCCGAAAAAGATGTTCATCAGAATTAGCATATGTGGCAAAGTCTCTAAAATATATAATTTGACAATACAATACGATTCGAAAACGAAAAACGCTACAAATAAGTCTGGAATACATAAAAACACATTTAATACATACAATGCAGCAGGTAACTTCAGTGGTGAAGACACACAAAGCACAGCAATGAACTTGCATTTCATAATTCCTGTGACTCTTGCTATTATCAGTATCATCATAATCATTTCTATTATATTCATACGAGGGAGAATAGTCAAGAAAAGCAAGAAAATCAAAGACAACGCAAATCAATATATTTGTGAAGAAAATGAAAGCTTGAAGACAAGTTCGCTTACCTAACTTAAAGTGTTATTTTTACCAGAAATCAAGTTTCCCTGACTTCATCGCTGGTTTCTTTGAGGAAGAATATACTATTGGCTGCGGATCCATTCGTTGCATATGGGGAGGATGTTATAAACTCACCATGTCGAGACCGTGTGCGTTTCTTCTATAAAACGGTTCTTCAACAGTTAGCACTTGTGGAATTGAGCAATGCTCATGAGAACAAACGCCAAAGTGTTTGTGATAACAAGATCTACCAGTAGATGGGGAAGGTTGATTGACATTTTTCTATTCTTAGAATCTCCTTACTATATTAAAATACAACTGAATTAATAAATAATTTA
>Bg-c20251 len=422 count=4 IPR:/ blastx_SP:/
CTGAAGGAATCACCGACATGAAACCAGTTTGTAAATAGTTGTTCACTGAAGAACAGTTTGACTTGAACATTTCTTGCTCTGTTAATACCTATGTGTCATCATAGCAGCACTGGGCGTGTCTGATTTGATATTTGTATGAGAACTTTCGACCAGCCTCATTCTCAAATTTAGTTTTAATTCAATTATTTGGATCTCTATTTCAAACCAAAATCTTAGCCCAAAGCTAAGGAAGACATGACAAGAGACCAAATTCTTAGCCCAAAGCTAAGGAAGACATGACAAGAGACCAAAGTCTTAGCCCAAAGCTAAGGAAGACATGACAAGAGACCAAAATCTAAGCCCAAAGCAAAGGAAGACATGACAAGAGACCAAAATCTAAGCCCAAAGCAAAGGAAGACATGACAAGAGACCAAAATCTTAGC
>Bg-c34658 len=298 count=2 IPR:/ blastx_SP:/
TACTCATCTTCATCTAGATCTTCTCGTTGACTCTCCCAAATATGTCTCAGTCTTTGATGGCTTTCAATCTCACGCTCTGTAACATCGTATGTAGACTTACGGTCAATGATCTCCTCTGCGCCATCCTCCCACTCACCGTCGTCTTCATCATCCTCATCATCAACATCAGCCTCATCAATGATAAAGCCTAAAGCTCCCTTTCTCTTTTTTTTCTCACTCGAGTCTCTTCTTCCTCATCTTCCTCTTCATCGTACTCATCTTCATCTAGATCTTCTCCTTCGGGTTCTTCCTCATCTCC
>Bg-c36192 len=278 count=2 IPR:/ blastx_SP:/
CCGCTACTTCCGACACCTATTAGCAGAAGATAAAAATAGTCACCTGCTGCCAATCTGCAATCGACTGCTTTCTCTCACACACGTCATTAGAACAATCTACGGAATTCGAGAAAAACGTGTAGCCACTTTTGTCAGCATCCGAAACTCCTTTCGTTCTCTTGTTAACGAACCAGGGAAAGTAGAAAACATTCAACATGTCTAGAGCTGTGACTATGGAGCATACGAGAAGACCAATACCGGCCACGTATTTGTGTTTGGGGTGTGTCCAGCCTTTGATA
>Bg-c23248 len=662 count=3 IPR:/ blastx_SP:sp|Q861W0|IKBL1_PANTR/NF-kappa-B inhibitor-like protein 1 OS=Pan troglodytes GN=NFKBIL1 PE=3 SV=1/5e-10/647-207/224-374 // sp|Q9UBC1|IKBL1_HUMAN/NF-kappa-B inhibitor-like protein 1 OS=Homo sapiens GN=NFKBIL1 PE=1 SV=1/5e-10/647-207/225-375 // sp|Q5TM19|IKBL1_MACMU/NF-kappa-B inhibitor-like protein 1 OS=Macaca mulatta GN=NFKBIL1 PE=3 SV=1/1e-09/647-207/225-375 // sp|Q8R2H1|IKBL1_RAT/NF-kappa-B inhibitor-like protein 1 OS=Rattus norvegicus GN=Nfkbil1 PE=2 SV=2/5e-09/653-207/212-375 // sp|Q9TSV7|IKBL1_PIG/NF-kappa-B inhibitor-like protein 1 OS=Sus scrofa GN=NFKBIL1 PE=3 SV=1/5e-09/446-207/291-374 // 
AAATAATTTATTATTTTCAAAGTTGACAAATAAACACCGTAAGGGAAACATTCCTGGCATAACGTAGGATGGCTCTATGTCTCTATGTCATGTAAATCTGAAGTTTGGGTTAGTTTAGAGGTTTAGAATAGCCTTATATCTCTGTAAATCATCCTAGAAACTGTAAAATGGCATTATGTCTCCTTGTCATGTAAATCTGAGCTTAGTTTGTTCAGAAACTGTGAAATGGCCTTCACCTTGCCCATGATCTTAGTCACATGATCAGGATGCATATAGTCCTTGAATTTCTGGGTAAATTTATCTGGATGCCAACGTACTTGCTGAGAGCGAAGATATTTACGATAGTCAGGGCCTTGTTTGTCTTCAAAGTCACTGAAGAGAATGTCACCAAGAAGAGTCAGGTCTAAACACGGCCAAGGTATACAGCCAAAAGTCAATGTTTTTCCCGCCAAATGGCCAAGAAGGTAGCTGAACTTTCTTTCATAATTCTCCTTCCGTGATTTATATCTGTTCAGCTTGACCTCAACACCGGGTTTGAAATTCTTCTTCATTTGTTCCCTGGCAGCCTTTAATTTCTCGTCCTCGCTCTCTTTGTGAGGCTTCTTTGTTTTTTCACTTGAGTCATCACTCTCCAGCTTTCTCTTTCTGTCTCTCCAAAATAT
>Bg-c27298 len=269 count=3 IPR:/ blastx_SP:/
TAATTATATTTTGTGATCACCATATCGATTATAAAGTAAGATTGGGGTTAGTATTCAATATTTCACTATTTCAATGTGATATGTAGGAAGCGTAAGTACGCATGAACTTGGCTTGTCTTGGCTACCTATGAAAGAGGCTCGGGCAGAGTTTTGTGTACTGAGCACCTAGATGCAGCACGGATTACATTCTCCCAGATACCCTCTTCCTTCCACTTGTTCACAAATGAGATTGGACATACTATAAGCACGAATGTAGCGCTATATAAAAG
>Bg-c4243 len=760 count=29 IPR:/ blastx_SP:/
TCCTGTTTCTTATTTAAGCTCACCAAAAAACAGAACGAATTCATCACTGACAGGTGTTGTTGATAAGTCATCAACTTATCGCAAGCTCAAAAGAGTTCCTAAAAGGCCAATGAAGTCATTATGTACACAGACAAGTCCACAAAAGTAGAGATAGTTTCATCACTTACTGCGTGTGTTTCATCACTTTTGAGTTAACATCTTGATAACAAAAACTTGTAAAGTATTATCATTTGAAAGGGAGGGAAAAAAAACTGCCATTTGTTTTTCTCTACTTTATAAATTTAATGGGCTTTTATAAATTTATTATTAAATGTATATACCATCATTTTAGTATTACATGATAATCATGTAATTTAAGCATTTTTATTTAAAACTTAAAATAAAAAGGACAAACAGTTTCTTATAGATCTTTACAGTGTCATCACCTTAATTCTGTAATGTTATTTTGTAATGATTCAAGCTTGAATTGTAGACCATCTGTAATGTTATGTTGTAATCATTCAAGCTTGAATTGTAGACCATCTGTAATATTATTTAGTGATCATTCAAGCTTCAATTGTAGACCATTTGTAATGTTTATATATATGTATAGTGAAAAATAAACTAATCTGCATAAAATGTCAATATACAATTAAAGTCACATTTCATAGATCTCCTATTATTGAATGCTATTATACAATTGGTTTGTTTGTTGTTGTTTTTTTGTGTTTTTGCCCTAAAAGGTTTGTTGATTGCAAAAAAACTGAGTATTAAAAAATCA
>Bg-c31400 len=414 count=2 IPR:/ blastx_SP:/
TTCATATCCCTGTCCCCGGTCATGTAAAACGCGATGTAGGCCTACAATAGGAACAAGAATACGAAAACCGGAAATTACATCGATTCGGAAACCACTTTACCGATCAGCCACTGCGCCTACATCAACATTAAAACGCATTATTATGTAACAGTTTATGAAAAAGTACTTCCACCTTCGCTCATCTTGCAGTCACTTGTTTCAATAGTATTTGACATTAGCCGTCATTAGATACACTGTAAATTTGTGCTTAACAAAACTCAAATGCAAATTCAAATTCAAATCCAGATGCAACTAGTTATAAATACAAAATACAAAAGTAAAAATTACGACAAATAATTAATTACCAAATAGTTTACAAACTACAATTTTAAAAAGACTAGGAAAATAGTTAAGTAGAAAATTTTTAAATTAATT
>Bg-c30370 len=546 count=2 IPR:/ blastx_SP:/
CTTCGAGTGAGCTCAAAATGTTGGATGTGTTTAGAGTTAAAGCCTTTTATTACCAATATTTATTGGCTGGAATGCTTGTTTTTAAACTACGGATCTTGTGAGATGGAGAGCCCTAGTCAGATATAAGACAGCACATTCCTTGACTGCATTATTTTCTATGGTTTATGAGATATCTCTGGAATGTAAAACTTCTTTCCAAAAAGAAGGCGAGATAAGTCCCCAAAGTTTTGTCAAAATTTCTAGTCACTTCAAACATTGCTATAAAAATATTGCGTGTAAATCAGTTGGGTCACGGTGTGTCATTCGTACAAATTGTAAAGGCAAGATTATTTTATCAGTATTTGCTTCTCATTGTGTTTAATCACCATGGCTGCCTGGTCGTATTGTATGCGCTTTGGACTGTCTTCTCGAAGGTCCCTGGCTCTAAATCGACATCTCTACCCTTCATTATCCAGTGGGAGGGTGAGACTAGGATGTAATCATCTTCAGTTCTGAAGGATTATACGAAACATGTTAAAACAAAAACAAATTTTGTTTAATAGGTTT
>Bg-c34504 len=301 count=2 IPR:/ blastx_SP:/
TTATTTTGTAATCAGCTTTTTATTATTATTTGATTTTGTTATCTCAGTATCATCCAAAACCTTATATATATATCTGTACATTAAGCTTAGTATTTAAGTTTCTTGTGTTGTTCAACCAGATAATTGAATTTACTTTATTTTAAAATCAAAATGTATGCAAACAACTGAAAATGCAAATTATTGTAAATCAAGTGATCCAAAAGCAGCAGTATTGTTCTGTTGTTGTTTTTTAAATTACTCCATAAGCTTTAATTAGTACAATTATTTTTTTTTCTTTAAATAAATTTTTTTAAAAATTTTT
>Bg-c30230 len=580 count=2 IPR:IPR001223:Glycoside hydrolase, family 18, catalytic domain; IPR013781:Glycoside hydrolase, subgroup, catalytic core blastx_SP:sp|Q13231|CHIT1_HUMAN/Chitotriosidase-1 OS=Homo sapiens GN=CHIT1 PE=1 SV=1/1e-06/44-280/315-384 // sp|Q9D7Q1|CHIT1_MOUSE/Chitotriosidase-1 OS=Mus musculus GN=Chit1 PE=1 SV=2/4e-05/56-280/297-382 // sp|Q15782|CH3L2_HUMAN/Chitinase-3-like protein 2 OS=Homo sapiens GN=CHI3L2 PE=1 SV=1/5e-04/8-277/307-385 // sp|Q91Z98|CH3L4_MOUSE/Chitinase-3-like protein 4 OS=Mus musculus GN=Chi3l4 PE=1 SV=2/0.001/8-280/306-386 // 
TTGGGTCTTATGCTCCAGACTTAAGAAGTATTCTCCCAAAGTACAGCGATATAGTGATCAAGCTCCTTTTGTCTATTACGAGATATGGAGAAATGATTGGCAACGTGTGGAAGAAAGCTTCTACAATTTCTACGAAGATGCTGTCAGTTTAAAGGCGAAGGTAAATTATATTAAGCAAAAAGGTCTTGGTGGATTTGTACTTTGGGAATTTTATAGTGATGATAATTCGAACGATTGCTTAGATGGTCGATATCCAATCTTAAGTATACTCAATCGAGAATGCACGTGAGGACTGTTGATGTCATACAAGTTTATTTACTCAACCGATGAAACTAGACATCACGTATTTAGGAGAGTCAAACAAAAAGATTCTTGGCTTTCAATATTTTTTTCTCTTTATGTTATAAATAAGTAACATTTAAACATTATTGGGATTATTGTTTTTTGTTTCTACTTTTTAAAAAATTTTTGAATACTTATTTCTAATACATAAATAGTCATCTCGTCCAAAATTTATTATTCAAATCTCTCAAAGTCATTCTAATATAGAAATAATAAAAGAAATATGGAAGAATATACG
>Bg-c2754 len=323 count=48 IPR:/ blastx_SP:/
TTATTACCTTGATATGTAGATCACTAGATAAACTTAATGGACAAAAATAAAAAAGTTAAAAGAAAACAAAGTAAGAAAAGAAAACCAAAGACCATCAGTGGAGAACCTAGGCATCAGTGGAGAACCAAGGCATCAGTGGAGAACCAAGGCATCAGTGGAGAACCAAGGCATCAGTGGAGAACCAAGCCATCAGTGGAGAATTAGTGCACAATGATTGAGCCACTAACCTGCAGATCAGTAACACATTGTTATTAAAAGAAAAAAAACTTGAATTTTGAAGCAAATTTACAAATTGAAAGAAAAATGTCAATAATCTAAAAAGA
>Bg-c34451 len=302 count=2 IPR:IPR020946:Flavin-containing monooxygenase-like blastx_SP:sp|P97872|FMO5_MOUSE/Dimethylaniline monooxygenase [N-oxide-forming] 5 OS=Mus musculus GN=Fmo5 PE=2 SV=3/1e-18/3-293/201-301 // sp|P49326|FMO5_HUMAN/Dimethylaniline monooxygenase [N-oxide-forming] 5 OS=Homo sapiens GN=FMO5 PE=2 SV=2/1e-18/3-293/201-301 // sp|Q8K4C0|FMO5_RAT/Dimethylaniline monooxygenase [N-oxide-forming] 5 OS=Rattus norvegicus GN=Fmo5 PE=1 SV=3/4e-18/3-293/201-301 // sp|P49109|FMO5_CAVPO/Dimethylaniline monooxygenase [N-oxide-forming] 5 OS=Cavia porcellus GN=FMO5 PE=2 SV=2/4e-18/3-293/201-301 // sp|Q04799|FMO5_RABIT/Dimethylaniline monooxygenase [N-oxide-forming] 5 OS=Oryctolagus cuniculus GN=FMO5 PE=1 SV=2/2e-16/3-293/201-301 // 
TAGCTTGTGATCTGGCGAAAACTTCAGAACAGGTTTACTTAAGTTCCAGACGAGGAGCCTGGATTGTACCAAAGACTGCTTTCTGGGGTCTTCCTGCCGACATGCTGGCCAACAGCCGAGTGGTCTTCACACTGCCCATGAAGCTACTGGACTGGTTTGTACAGAAGCAAGCCAACTTCAGGATTGACCACAATACCTACGGTCTTCGTCCTCATCACGGTGTCCTCAATTCTCACCCCACCATCAACGACGAACTTCCTATCCACTTGGTATCTGGTCGAGTTAAAACTAAACACAGATCG
>Bg-c6317 len=766 count=18 IPR:/ blastx_SP:/
CCAATGGCAGAAGGAGAAGGATTTTTATTGAAGTCAGTAGCTTAAATTTAATTGGTTGCTGGCTGAGTCTTTGATTTCCTTTAGTCTGTCTTGATTCAGTTCCTTCCCCTGAGGTAATTTGGTTGACAGCATACATACATGTTGCATTTTTTTTTTATGTGAACTGTTGTACAAATCATATTTAATCTTGATATTGTTGGTAGTTGGGAGAGGTTTTTCTTTTTTTTTTGACATTTTTTTTTTTCTTTGATTTATTTATTTAATATCACCATCATTCTTTTAAAGATTTATGTGAATTGTGTTCTAAAATGTTCCAAATCATATTTTAAATTTTGGTTTATTTTAAAATGTCCCTTAACAAAATTTGGTGGTAATTAACACTTCATGTGAATGTGAGCTTTCTTTTTTTTTGTAATTTTTGCATTTGAGATGTTAACACAAGCTGGATAAGGAATTGAGTGTTACAATTAGAGTTATTGTGCTCTTTATAAAAGAAAGAGCACCATATTTTTTATGTATTAAAAAACAACAACTTTAATTAAGATGCAGAATTGTTTGAAATTGTAATGGCTCTTGAACTATGTTGTTCTTTTAAGTTGTCTCGTCCTTTTGTAGATTGATTGATGTTATTGTTCACAAATGATTAAATGAAGAAACTCTGTCACAGTATGTTCAATGTCTAAACTGATTTTACATGTAATATAAGAATGTTGTGCTTCTTTATGTTGAACCTCAAGGCAAGAGAGAATAAACTGGACAGAACTCT
>Bg-c42918 len=126 count=2 IPR:/ blastx_SP:/
AAGGATACATTATGCGGGATTAATACTAAATTACATTTAATGAATAGCTATGCTCTCTTGGTGTCAAGGTTTTTTAAAGGATACATAATGTAAATATTAATGCTAGTAAGTCCTAACTACTAATAA
>Bg-c40389 len=241 count=2 IPR:/ blastx_SP:/
TGTAAGAAGTCTGAAATCTATCTGGCAATGTATGCTATTAAATAAATATCAGCCAAGCCTTGCTACATGGGGCTGAATGGTTCTACATGTTAACAATCTTTATATACAATCCTCCCTGCCCACTTATCCAGGAGGCATGCCACCAAAGTTGTTTCTATATAAAAGAAGAAGACTGTATCCATATGAATATCTTGGGAGATACACATTTTAAAGCAATGTTTGTAGAAAATAATGAGCTCTA
>Bg-c4549 len=510 count=27 IPR:/ blastx_SP:/
AAATAAGTCTACTATACGTCACTTTCTGAAAGAATTAAAACGTTCCATTGAAAGTCACCTCAGAGTCCCGCTATGAAAAGAAGTCGACTGATGAGGTTACCGTCTGCTGTACAGACGTGCTACTGATTTTGAAACAGAAGCAGTGCCCATTCCTCTAGCTGACCAGTGGACTGTAAGAGATGTTGGAAAGGGGAAGTTGAACACAAATGAACCTAAAATAAAAGTCTTGGTGGTCATTTTAAAATACGTAATGTATTGGAACTATGTGAGAAAAAAAAAGTACAACTATCAAGATGAATGAAAAGAAATAATCCAATGTTCTTGCACCCAAAAGAAGCTGTTCTGGCCTGCAGAAATTAGAAAATGCTATCTAAAAAACAATGCATAGAAATTTGGGGGATGTTATCTTTTAACTCTATTAAACCCGACTATTGCTGCTTCAAAACCATCATGACCCAATGTTCTATCCTTTCGTTTAGTAGGTTAAAATAGACAAAAATAATTAGTGTT
>Bg-c21199 len=319 count=4 IPR:/ blastx_SP:/
CAAAATGATACGAATATTAACTGATAAGTCTTAAGAGTCTAGTCTAGTAATAGAATTAAATTTAAACAGATATATCTAAATCTAGATATGGAATCTAGATCTACTAGTGATCGGTCTAGTCTATACAATAACAATAATAGAGTCTAGATCTATAAAGAATTTACCAGAGACTATAAAGTATAGTAAAGACCAAAATGGCTAGAGGTTACGATTTAACTTCAGATGCTGGTACAAAAGAGTACTTACGAGATGTTGAAATTGAATATCAATTACTAGACTAGACTCTTAAGACTTATCAGTTAATATTCGTATCATTTTG
>Bg-c3708 len=570 count=34 IPR:/ blastx_SP:/
AATGGCTGTTTAACACAGTCTGTGAACTTTAGATCTAGATTTATCTTTCTGTCTGACAGATTACATAAACCCTATCATTTAGGCTTGAAGTTTATATAGAGAGTTGTTGATGGACATCACTGCAAGTTCATTGACGGCTCTAGTCTGTAAATGGCAGAGTCCTCAGTTCCCAGATTGACATCTCAGCACGATCAGGGGATTTACAAATGGGAACATGCTGTTCTACAGGTCGTCAAGAATCGTTAACCGAAGAATGTCAAGGTCACCACAGCTTCTACATCCACCCCGAGGGTTCGTTCAACCAGAGTACCTACAACATTGTATACAGACCACCACAGTTGAATGAACGTCTAGCGCCTGGCGAGGCTAAGCCCAGATTTTGGCTTAACCCGAATAGCTTTGCCAGCGGCCCTGCAGTGGTCAGTATAGCGTGGTGCCAGTTAGGACAGACACATCCAGTGTGTCCCCCACCACGTCCTGTCAAATAAATAAAAGATAGGAAACAAAAGAAAAAAAACAAACAGAGTCGACTCCGGAGGGGAGGCGCGACGCTTGTCTAGTGATCGGAAG
>Bg-c18763 len=252 count=5 IPR:/ blastx_SP:sp|O08760|OGG1_MOUSE/N-glycosylase/DNA lyase OS=Mus musculus GN=Ogg1 PE=2 SV=2/1e-18/2-250/253-337 // sp|O70249|OGG1_RAT/N-glycosylase/DNA lyase OS=Rattus norvegicus GN=Ogg1 PE=2 SV=1/2e-18/2-250/253-337 // sp|O15527|OGG1_HUMAN/N-glycosylase/DNA lyase OS=Homo sapiens GN=OGG1 PE=1 SV=2/2e-18/2-250/253-337 // sp|Q9V3I8|OGG1_DROME/N-glycosylase/DNA lyase OS=Drosophila melanogaster GN=Ogg1 PE=2 SV=2/9e-15/2-208/254-322 // sp|P53397|OGG1_YEAST/N-glycosylase/DNA lyase OS=Saccharomyces cerevisiae GN=OGG1 PE=1 SV=1/5e-05/2-208/245-328 // 
CTGCGTTTGTCTGATGTCACTGGACAAGACTGAAGCTGTACCAGTAGACACACATGTGTGGCAAATCACAATGAAACATTATTTGACAAAACTACAAAGTGCCAAGTCTTTAACTGACAAACTGTACAATGAAATAGGTGATTTCTACAGAAGTTTATGGGGACCTTATGCTGGTTGGGCTCATTCTGTTCTCTTTACTGCTGACCTAAGACACAACAAAAGAAAAGGTGAGAATGAAACCACAAGAAAAAA
>Bg-c32136 len=376 count=2 IPR:/ blastx_SP:/
TTTTGTCATGAGGCAGTTCACCCTCTAGTTTGGTTGTAGCGGACGGTGTGTTGGCTCGTAAAACGTTATTTGTCTGTACCAGTTTCTCGGACTTATTATTCAGTTGGGTTTTTGGGACCCCTGTTTAGGGGTGCCAGTGTTGGAGTTGTGGGCGTCGCTTGCCGTCTCAAAAATACATATTGCAGTTTTTCATTGTCATTGTCAAATTGGCGATTATCTGAGGTCTAACCTTACTATTACCAAGGTTGGCAGTGTTGTGACGCCAGTCGGTCAGAGTCTGGTGGACCATAAAGCCAGGGTGACAAGTTAGTTGCAGTAGCAAAGAACAGGCCCGCAACGAGGACGTCTTTTACAAAGATGATGATGATCTTAGGAA
>Bg-c36784 len=272 count=2 IPR:/ blastx_SP:/
TCTTTAATAGCAGATGCAGTGGATAAATGTGTGGTAACAAGGCTAGGATATATTACAAACGATGGAATGTGCATTGAAAAAAATGTCAGTTGATACAACTATTACGGTGATGAAATACATAAAAGTTAACAGATAACTGGGTAAAACATAACTATTTACATTCATTCTTTATTAGCTCTGAGTAAAATAAAATGCTACTTAGAAAGTAAAGTAAACTTGTTACTTGTAAAAAACCAAATGTATAGAAATCGATGAAACAGAAATTAACACTA
>Bg-c20368 len=408 count=4 IPR:/ blastx_SP:/
CTTAATACCATATTAACCTTTAGTGCAATAATATGTTATTAAGCATAGATACTATTATTATGCTGTCTATATTACAAAATATGCTTAACTTTATTTTTTCCGTTATTATTATTAAATAAACTAATAATTATATCAAATATAATGGTAAATGAAGATATTGTATATAAAATTCCACATGTAATAAATTAATCAATCAATCATTCAATCAATCTCTCTCTCTGTCCCTCTGACTCTGCCTCTCTCTCTCATCTGCCTGTCTATCTGTCTCTCAGTTTGTGTGTCTTTCTTTTTATCTGGCTGTGTGTTTGCTATCAACTTCTGATCTTACCTTAACACTTGGCTGCTTAAGAATAACGCATGTAATGTTTAGTTAAATGTGGGTATTTATATATGTACAAGAATAAGTTG
>Bg-c5806 len=717 count=20 IPR:/ blastx_SP:/
GAGGATATTCTACGTGTTTTCTTTTAGTAGTCGTTTCAGTTATTGTTCATGTTCATGAACCAGATAATGAAATTTCAGGACAACTGGCATTTGAGCTTAAATGCGGGTGCATCTGAAGTTGAGATGGCAATACCAAAGAATCTTGAGTGGAAAAAATAGCTTGAAAGGATAGGCTGTGATTTAGTAGAATAAGCAAACTGTTTCATGTTTAGGAAAAAATAACATAGAAGAGAGAGAACAACTGTTGAAGCAGTTCCTTCCAGCCTATCATCTACCCCAGTCTAGGACAAGCCATGGATTATGAATTCATGATGCAACATTGCTCAGAAAGACAAAGGGGTGTGTTTGTGTATTGGCCTGAATAAGGAGTGGTATAATCTGTATGGCGCATAGAGTTGAACTCCCTGCTGTTTTGTTTATTGGGAGGTGCTGGATCAGTTTTGGCCACTGAACTTCAAAAAGGTTGTAGGCACTGGAGATTTAGGAAGGATAATATGTACGGAAAGAATGGTTGATTGAATTAGTATTTTTGTAACTCAAAGAAAACATCAAGGGAAATGATGAGCCTTTAAGCTCATGTCACGATATCTTCATACTTAATACCTCCAGAAAGACAGCGATCAGGTTTTGGGTGTCCTGCTGGGTGTGCCTTGCGTCTTTTTGTGAGCCTTTATTCTTCTGGCCCTTTGAAACTTCCCCAGTTTTTCTTTTTGCAGA
>Bg-c8096 len=261 count=14 IPR:/ blastx_SP:/
GATTTCTTGGTGACACCATGATTTTTTTGTTTATCTGGGGTCTTTAAGTCAGAGAAAATAGCTTGTGAGTTGGTTACAGAGCTTGCTCCTTTATCTTTAACTGGTGAGGAATGACTACGAGCAATGTCTTCCTCAAGTTCACATTTAACAGGTCTGTCTAGTGTATCCTCACTGACCTTGGTCTCTTCAGACTGTAGCAAATGTTTGTAGTAGGTTTTAAGTCTGAAGGAAGACAAAAATAAATAAATGTCGTAGACAAAA
>Bg-c16183 len=253 count=6 IPR:/ blastx_SP:/
AGAAATACAAATATAGAAGACATTCTAAAATTGTGTTGATTAACATTTGTCAACACTTAAGGAATTAATCAACAAGAGTGATAATACCTGCCTCCAAAACATGATTAATATAACATCTATGTACAAAAATACAGAGGTTTTCTCTGTGAGAAACATGTAAAGACCATCCTCTTTTCATTTGAAAATTCTGATAGCCTCTGGCTCACTGCTAGCATCCAGAAGAAAATTGTAAACTCCGGTTTTGAAGTAACAT
>Bg-c21529 len=293 count=4 IPR:IPR014044:SCP-like extracellular blastx_SP:sp|Q9ET66|PI16_MOUSE/Peptidase inhibitor 16 OS=Mus musculus GN=Pi16 PE=2 SV=1/4e-04/125-280/18-71 // 
GTTGTCGGACCTGCCAGTTTGGTCTTTGGTACTGATCGTCTTTATTAGTCGACTGGATGTCTCAGGCTCAATAGAGTTGAGGAGAGTATTGAATGTCACAAGTCATAGGTTAGTGAAAAGAGGAACTAACGGATTCAGTGAAGAGGAAAAGGTGGCTATGGTCCTTGCCCATAACGAGGCAAGAGCCAAAGAAGGTGCAGCCGATATGCTTGCTATGCACTGGGATGAGGCTCTAGAAAACTCTGCCCAGGCTCATACAGATAGATGCAATTTTGAACACGGCACCAAAATGG
>Bg-c10125 len=613 count=10 IPR:/ blastx_SP:/
CCAATTGATGTGTAAATTCATCATATTTCATTTTTGTACAATTCAGCTCATTATTCTGTGAAAGTCTATGCTAAATGTTGTACATTTGTTTTTTAAACAAATACATTAACTGATATAATCTAGTGTTCATTTGATTAAAAGGTCTTGTCAGAAATTGTTTAAAGGCATGAATATTTCAAACTTTTCATTATCAAATACTATAGAGATTTCAATGAACTCACTTTTTAAACATTTAAATTAAGGGAACACTGTGAATATAAGACTTGTCTTTGCCATCAGTTTAAAATTGCCAAACAAAATGGGAATCCATGAGTCTGTGAAAAGCTTTAATCAAGAAAAATAGGAAATGACAAGTCATATATTTTAGTAGATATTTGCTGTTAGTATTTTACTGAAAAATCATTTGATTTTTTTTCATTCTGTGAATTTAAAGAAATAAAATGTTTATTTCTATGCATTCATTTTATTTTATTAGGCTACATTTAAGTTTATTTTAATAACAGTTTTAAAAAAACAACATAACATTCATTGTCTCTTATGACATTTATTTTCTTTTATGTATCAAGAGTAGATAGAAAATAGATAAAAGAATTAATATATTTACACACTTTCT
>Bg-c7234 len=1800 count=15 IPR:IPR009038:GOLD; IPR000348:emp24/gp25L/p24 blastx_SP:sp|B4LYB8|TMEDE_DROVI/Transmembrane emp24 domain-containing protein eca OS=Drosophila virilis GN=eca PE=3 SV=1/4e-72/3-569/28-216 // sp|B4KB41|TMEDE_DROMO/Transmembrane emp24 domain-containing protein eca OS=Drosophila mojavensis GN=eca PE=3 SV=1/4e-72/3-569/28-216 // sp|B4JG34|TMEDE_DROGR/Transmembrane emp24 domain-containing protein eca OS=Drosophila grimshawi GN=eca PE=3 SV=1/4e-72/3-569/28-216 // sp|Q295B2|TMEDE_DROPS/Transmembrane emp24 domain-containing protein eca OS=Drosophila pseudoobscura pseudoobscura GN=eca PE=3 SV=1/5e-72/3-569/28-216 // sp|B4PVC6|TMEDE_DROYA/Transmembrane emp24 domain-containing protein eca OS=Drosophila yakuba GN=eca PE=3 SV=1/9e-72/3-569/28-216 // 
AAACCGAAAGAAAATGTTTCATCGAAGAAATACCAGATGAAACTATGGTTGTTGGCAACTATAAAGTCGAAATGTTTGACAAAAATATAAATGCCTTTGTCCCAACAATGTACGGTCTTGGTATGCACGTTGAGTGCAGAGATCCAGATGATCGTATAGTTTTATCAAGGACATATGCAGCAGAAGGCAGATTCACTTTCACATCTCATTCAGCTGGAGAGCACATTATTTGTCTCAACTCCAATTCATCTGCATGGTTTAATGCTGGGCAGCTGAGGGTTCACCTTGACATAAACGTTGGAGAGCATGCTGTCGACTACCAGCAAGTCCAGGCTAAAGATAAACTAACAGAGCTCCAACTGAGAGTCAGGCAACTGTTGGACCAGGTAGACCAGATCACAAAAGAACAGAATTATCAAAGATACCGTGAGGAGAGGTTCCGTCAAACCAGTGAGAGTACAAACCAGCGAGTCTTGTGGTGGTCCATAGCTCAGACTTTAATCCTCCTGATCACTGGTTTCTGGCAGATGCAGCATCTAAAGAGTTTCTTTGAGGCTAAGAAGCTGGTTTAATATTTGTAGATTATATTATTGAGTGATTGCTGTCAGGTCATGTCATGTTTTATTTATTTGTGTCCACTATAAAATTTCATCCATTTAAAACAACACTTCAGACATATGAGATTTAGGAAAAAAATTTTACATTCCTAAATTATAATTCACAAACTCCTAATAACTCCTAATTAATAATAAAATATTAAATGCAATGAATACATTTTATTTTATGTACAGTAGACTTAATATAATATGTCATTGCCATTTTAAAACAGATCCTAAAATGTCTACACATTTCATTGTTGGTATTGCTGTGGTTAAGTCTACATTTTAATATTGTAAGCATTAGTATAAATGTTTTATGCTTATGTAGTGCTCTTCTTTGAAAAAGTGTTGAGCTCAAGTGAATGTTGTATTTGCTTTGGCTGGGGGGTGTAAGAGATGGTCTAAATGTTGGCGAATGATGGAATTCTTTTCAAGAATTTGATGTAAAATATTTTATGCATTTTGTTATTTTCAATTTTTTTTTTTCAACCTTTCTATACCACTAATAACTGCCATAGTAACAGTTTGTTTAGTGAGAATTTTCTTGTGTGTGTTAGCCTTATGATTGTTTGGTTTTAAATATACTAGGATCTGTACACAGATGACATGTAGTACATTCCCTGGACTTAGGTGTATTGAAATAGGTTTAGAACTACTGCTATTGTAGGAGACTAACTGATTGGTGCTACTTGTTCTCATAACTTCTCACTTTTAGCATCCTATTGAAAGTTGTCTTTTATAGGGAGGCGGGGGTAACTAAAGGAAGCAATGGTCAATAGGGGAACATATTTATTATGAGTTACATAAATTGTGTGCCTTTTAAGTTACCGGTATTAATGATATTTTGTATGACATCTACATATAAACATGTCTTAATAACTAAAAGACTACAGCTAGTTTCAGGGAGCACACATTAGTTGAACAATTTAGAAGAGGACTTCCTGTAAAGTGAGAGATGAGTTTTATTTACACTCAAATGTACAGAGAGAGGATTCTGTGTTGTTTTTTTTTTTATCAAACTTACATTAAGTTAAGGTTTCATCAAGAAGTTGTACTGAAGTCAAAGTGTGGTGGGATGTTTGATCAAGGATAAGCTAACAAAATATCAGTCTTGTCTTGTTGTCTAATTTAATGATGTCTTTTCTCTAAAATCTCACCCTCTAACGTTGTTTAGAATAAAACAATCCTGTTATCAAAAAAA
>Bg-c18861 len=243 count=5 IPR:/ blastx_SP:/
AGGCATATATATAATAAAAGCAATCATAAACTTGTTTAACACTTATTTATATAAACACCTGCTTCATACATTTTAGTATGATTAATTTAAGCAGACTGTATACATAGCTTATAATAATTAGTTTTTTTTTTTTTTTTATTTCCACAGTTATGTGCTATTATTTTAAGTTTTAAGTTGATATATTAATAATGAGTTCTGGACACTCAAGTAGAACTTTATTGAAATCAAAAGTATCAAATGAAG
>Bg-c15297 len=408 count=6 IPR:/ blastx_SP:/
AACTAATCTATTTTTATTGTAAATGTTCAGATTACCAGGTACCAAAAATACGCAGAATACTGCAGATGTGTGTGATTATTGTCACGGGTCCAGTGGCGTAGCTAGAGTGGGGGGAGAACTGGAAAAGCCCTACTGGCCCCCACTTTAGTCGTGCCCAAATGAGTGTTTGAAATTGATTTTTACAATAAATATTAAATATTAAGCAAAATGCCGGGGCCCCAAAGAGGTCACACCCAAGGCCCCCAAATGCTGGCAAATTCCTAGCTACGCCATTGCACGGTCGTCCTCCGCCGAATTAGTACATTTTCCATAGAAAACAATTAATTAATTAAGACTTATTGATTAATTAGTTGTTTATTTATTTTCATTGATGCAGGTGTTGTCATCAACAATGAATAATTGTGCAAA
>Bg-c7543 len=389 count=15 IPR:/ blastx_SP:/
ATTTATGAGCAGCAAGTTAAAGCATGTGGAGTTATGACTTTGAAGGACCATAGGTTGTATATTGTTTTTTTTAATTGGAATCGTATTTTTAAAAAAATACGAAGTCCTGTCACTTGGTGATGTAACACCACTTTCCACAAGATGTTCTAAACTAAGCCAGATTGTATCTTTCATGTTTGAATTCAACTTAAAATGTAATAGTCTTTAGTTGGTGTTGTTTTAAGTGTTATAAATTAGAAAAGTCTTCACTTTTTTTTTTCCTGATTGAAATATTTGAATCTTCAGATGTTTTGTGTGTGTGTGTGCTACTAAATGTTATGTCAGTGCTACTAGATGTCATGTCATTTCAGAATTGTTGTACGCTCCTCAATAAAATGTATTCTTGTAAA
>Bg-c30077 len=613 count=2 IPR:/ blastx_SP:/
GAAATAGTTAGTGATGGTAGTAGAAAGTGTAACTGTTCAGAAAACAATTGGTCTTTTAAAAAAAAAAAGGGAAACAATAACCTGCCAATTATTTACAGATAGTGAACTATTGTCACTGAAATAACTCTTGTCTCAGTCTAGATATTTGTGATGATGACATTGAAGCCTTTTTCTTAATATTTTAAAAGTAGTAACTTAACAACCTTAACAATTGCAACTATCAGGTTACCACAAGTCAAGTAAACTGATGGCTAGATCATGAAATATTGGACTATCAATAGGACTACAAAAACAAAACCCAAAGTATTGAGAATATTTTTATATACATTCTTTAAAATTGTATATTGTTTCTAACTTATTTGTAACCAAAGATTTGAAATTGCATTAATTGGTTGCTATCAATACCATCATCCCATTGCAATTTAGTGAAAAGACTTCATCAATTGACATTGTATATGTATAGTATTTAGTGTTTAAATTGATTTCAAGACCTACAATGTTTACAATGAAGTATTTGTCATATAAGTGGGGAGGGTGGGGGTTTAGCTGTTGATGTGAATCCTTTTAAAGGTATATTTTAATGTTTTTTTTTACTGCAACCATCTTGATTGTT
>Bg-c38146 len=260 count=2 IPR:/ blastx_SP:/
TTGTATTTGTGGTCGCTGGTATGTTAATTAAGGTGTGGTGAGAAAGGACCAGTTGAATGGGAACAATTAATGAAATTGAAAGAGTGAAGGAGGGAAGCATTGAGCACGAATGTAACATAAACATGGCTAAGGGTACACTGGAAGAAATAAAGGACTATCATGGCAATGATGCAGTGGAGGAAATAAAAGACAATCAGGGCAAGCTTACAGTGGAGAATATAGAAGCCAATCAGGACAAGGGTACAGTGAAGGAAATACAA
>Bg-c28292 len=247 count=3 IPR:/ blastx_SP:/
ATCTGTCAGGACTTTGCACTTGTTTCTTTCGACAATATCTGGGATGACATTTTGTAGATGGAGCTGAATGCGTTGGCCCTGTCGACTGCTGGCTCGCCGTACTGTATGACTAATAGACATCGCCTCACACAGCTTGTCCATATGTACAAGCTCATCCGTTAGGTCATAGGTCTTTATTAGATCCACCTGTATATATTCTTTATCGATATTTGTCTGTCTCAGACTTATCAGCTTATCATTGCAAGAT
>Bg-c37111 len=269 count=2 IPR:/ blastx_SP:/
TTTTGCTAGGAACAACAGTCAGGCTGTGGAGCTTTACTTCCCCTTTTAGCAGAGAAAAAGTTATACCTATTTGAAAGTAACTGGCAGGAGGCTGGAAGATGATACTTAAGCATCCAATTAATCCTTTATCTATTAATATGACAAGAGTGAGGAAGATTAATATAATTTGCATTGTAACCTTTTTTTGTTTTGTTTTGCTTGTCTTTTGGTGTGTACATCCATAATGTAAGATGTAGCAACCAATAAAAAATAAAACAAAAAATGGAATA
>Bg-c24669 len=390 count=3 IPR:/ blastx_SP:sp|Q0VC80|YRDC_BOVIN/YrdC domain-containing protein, mitochondrial OS=Bos taurus GN=YRDC PE=2 SV=1/1e-05/364-200/215-271 // sp|Q86U90|YRDC_HUMAN/YrdC domain-containing protein, mitochondrial OS=Homo sapiens GN=YRDC PE=1 SV=1/8e-05/370-200/217-274 // sp|Q3U5F4|YRDC_MOUSE/YrdC domain-containing protein, mitochondrial OS=Mus musculus GN=Yrdc PE=1 SV=1/1e-04/340-200/228-275 // sp|Q499R4|YRDC_RAT/YrdC domain-containing protein, mitochondrial OS=Rattus norvegicus GN=Yrdc PE=2 SV=1/7e-04/364-200/218-275 // 
CTGGAAGCAAGATTGAAACATACACACCAGGATACTTATTACATTCATCTGTATTTACAAGTGTTTTTCTTACATTAACTTAAGGCTTGAGATCACATTAGTTTATTATACAAATACCAACGCCACTCATTGAATCATTTTCCATGGCATGAGGTCAGTTTTGTGGCAGTTTTTACAAATGTTATGATGATAGTTGGGTACTGTCCTTTAATCCAAATCTCTCTAAGCAGTCCACTGTCGACCTGTAAGCACTGCCAGGACGAATGATTCTGTAGACATTAGGGATAGACAAGTCCACAACAGTTGATCCTAATCTGGCTTCTTCTGCATCATTTAATCCTCCCTCCATTTACAACAATGTCAAGCTGAGGCCAAAGATTTTTACAAATT
>Bg-c32310 len=369 count=2 IPR:/ blastx_SP:/
AAATTATTAGAAGATCTACATTGAAAATGTGTAGCTAGAGACACAATATAGGAAAATAAAGGTCCCAGACACACGAAGGCCTACATTGGGTAGATTGGTTTTTTAGACATAACATTAAATAGACATTTTGTGATAATTCACTAACTTAGTGCAGGCTATATCTAGTTAAATTGTGTTATACTATTGATGACAAAGATCATGTGGCTTTATACTTTTATTTTAGTGATCTATAGAACTAGTAGTCATTCTGTTTTCTGTATTATTTCATGCCCAGCATCAGCTTACATTTTTTGTTTGATTTCTAAAATGCACTTAGAAGGCTGTAGTGGAAACATTGCCAGAAAGTAACTTGGGTAACAACACACTTTA
>Bg-c5339 len=804 count=22 IPR:/ blastx_SP:/
AACTTTTGTCTAGAAACTACCTAAAATTTTATTGTCAAACTTAAAAAAGAAAATAAAGCCCAAATCAGAATTGTATTGTGGAGATAGATAGGTGAAGAGATTGGAAACACAAGATTGATATCAACACTGCGGGCTACATACATCTCAGCAAGACAGAGAACTAGTTCACATTGGCTAACACATACAAATCTTCAGATTTACCAAGACACCCAGAGACAACAATGTTGGTTGAAAGAAAACTTTTACTTTTAATTTGGAATGATGAACAAAATAATTAATTTGTTTCAAATGCTTCATTTATCTCAGAATGCTATAGCGTCAAGGAGTTCGTCAATTCTTTGACATTGGCACAAAGATTATAAAAATTATAAAATGTGAAAGGAAAGTAGTTAAAGGTTTAAAAGTGTGCTGGGGATCATGGTTATAATTATTTCCATTAAAGGAGATTAAAAGTCAGGTGAGCAGGTATAAAATGGAACACTATAAGTCAGTTTATACAAATAAAAAGATGTTTATGGACATTATGTCTTTACTTTACTTTATGTCGTTTACTTGCAGAACAGACCACAGACAAGAATCATGTCAATGTTTGATGTAATCTTTATAAGGCTTGTCTTGAAGCCTGAAGATAGCAGCTACAACCATGCAATATTTAATGATTTGCAAAATGCTTAAAAACTAATATAAAATAAAAAGGAAATTAACTATTACAGGCCATGGAAAGTAGAAGTGTCTTCACTCTTTTTTTTTTGTTTGTACACTACTTGGTCGTCCTTACTAATTATTTAACTAAACTAACTCTAG
>Bg-c13113 len=684 count=7 IPR:/ blastx_SP:sp|P51942|MATN1_MOUSE/Cartilage matrix protein OS=Mus musculus GN=Matn1 PE=2 SV=1/3e-04/291-545/331-418 // 
CCAAAGCTGACAACTACGACAAGCTGAATAGTATCATCAAAAGTCTGTTCAAAAAAACATGTGACAAAATTGGACAAACTACTGTTCCACCAGATTATCCAGAAGAAGATCCAAACGTCTGTACACCGAAGAATCCGCTGGACATTTACTTTGTATTTAGTACTTCTGACATGGGCATTGGGAAAACCATTTGGACCAATGAATTTATTCTGGCGACTGTTTCTACTCAATATGGGAACTCACTCTTTAAGTATGGCTTCATAGCCGATCTATGTCTTGGGGACTCTGGCTTTAAACTAGAGAGCTACAATTCAGACAAAGATATAAAAGCTCGATTGAAACCCTATGAGACCAGAAAGCTTTACACTCTGCTACAGCAACTCATTGATTCTGGTTACACCGCCAATCAAGGAGCCAGACGTAACGCTAAAAAAGTTGTCGTCTTGGTTGTAGACGGTGCCAAGTCTGCAGAGTTAGTGTCAAAACAAGTGAAAACTTTGGTGGACAAGGGCATTTCGGTGTTCATTGCTGACCCAACCAACTCTGGAATTAAGATTAAAGGTACAACCACACTTGTGGGAGAGTCTCTGGCGCAGTCTACAGAACTCGTCCAGTACTTGTGTAAACCATGTCTCTCTAATAAATCAAACTAAAAGACTTTGGTGAACTTAGCTCTGGACTGCC
>Bg-c12639 len=327 count=8 IPR:/ blastx_SP:/
TCTTTTCAATGAAAAAAACTTTATTAACAACGAAACGAGTAGTGTTTCCTGGAGACGACAAAGCTCGACGTCTCACACCAATAACAAAGGGAACAATCGAAGATAACAATAAGATCTAATTTCATATAACATAATTGCCTAAGCATCAATATTCTACGAAAACCTAAGGCAGCGTGCAACCTAAAACTGCTTGATCCTCTGATATGATATCTGTAGAGATCAGAGAATGCGTTTTGATTGAACAATGTCCCTTTCAACTTCCTTGTTGGCTCTATTTTTTGAAGGGCAGCTTCGTCATTCTGTGACGTAGTTGTTAGTCGTTTTCCC
>Bg-c41764 len=217 count=2 IPR:/ blastx_SP:/
TTCACAGAACACCAGTTTCCTTCATTGTATACACCTTTGGATGCTGATGGCCAAGAATATTATTATAACTATGATGATAATGTCTTTAAAAATGATGAGGGTGTTGAAGATGATGTGAATGATATGGAAAATGAAAAACTGTTTCGTGAGATGATTCTTACTATATCCCTGGATGATGATGATACATCCTTCATGAAGCAAAACAAAAGTGGAGAAT
>Bg-c7383 len=652 count=15 IPR:/ blastx_SP:/
TTTAAAAAATACCAAATTTAAAAATTCAACAGGATTATATAATTTCAACATGTGGTCTAGTGATTCAAAAAATAAAATCACTTTAAAGCAGTCACAGTCAAATAATCATCTTCCATATTTCATAGAAGTCAATGGATCAAGCACTGTCCTTCACACCTCAATATGATGTAAGATATACAAGAACAATTTAAGTATAAATAGATATCTAATTCAAGAGTGAGCAAATTCTATCTCTGCAATACACGCTAAAAACAATGATCTCAACAACATAGTCCATGGCCTTATCTCTGCTTGTACAGTTACACAACAGGAAATAGTACAGAACATATTTAGAATCTAACTTATTAAGATACACTTTCTGAATCAAGGTTGGCAGAATCACTTTCATCAGCAATCAAAGGAGTCACTTCTTGGTTATCAGTAGCCCCAGAAGAAACCAGTACTGGTAATTCAGCTTCCATAGGCAGAACTATTGGTATATCAGACTTATTAGTTGGCAAGCTGCTGGGTGCGTCAATAGCATTGTTGACATCGATAGCTGGCTGCTGTACTTCAGAAGCAGCTGAGACATTTTGAATGCAACCATCTATTTCTGGAACAATACTTGGAGCTTCTGTCAAGGAAACAGATGCTGAGTGTTTTGAATTATCTT
>Bg-c13557 len=451 count=7 IPR:/ blastx_SP:/
TTTCTTCTTTTAATAAAACAACAATGTTTGGTACTCTACAGTTTTTATGTCAACTTATCTCCCTTGCATTAGTACATTTTCAAAGCATTTCTCTTAAGGTAAAACATCTTAATCCCAATGTCCAAACACACACACAGAGACCTCTCCTTAGTACATGTATAGAATCTCTTTTTACAGTCAGGTATCTAATAGTAGGCCACACAAAACATGTTAAAATAAAAGCATTGAAAGTTGAAAACCTTAACAGTGACTACAGTACATACATAGAGCTAGAACTAACAATTTGGTTAAACAACAATGAAGCAGAACCAGTTCAGAAGAAAAAACAAGAACCTAGGAGTCAGTTCCAACAAAAAAAAAACCCCTAGAAGTCAGTTCCAACAATGATGACAATGTCTATATGTGTGTACCAAGTTGAATGTTCCAGAAGTAAAATGACAAGCGAATGTAC
>Bg-c31934 len=385 count=2 IPR:/ blastx_SP:/
GATCTTCTGTAAACAGGGTAAAAAAGATGTATCAAGCTAAACTGCTAAATGTCCGTGCACTGCAAGAAACTTCACTATAAAGAGAGTGAAATCCCAGAGGAATTCATGACACAATATTGGGTCCTTTTGTTCATTCTGTTAAAAGGGGAGCCTGCCTTATAGCTGCTTTCCTTCTGAGCAGGTTTTAACTGTACATAAAACACGGCATCTGAGATGTGGAATTTATCTCAGTCTATCAGCTGTACCGCTGTGTACGAGCTGCTGCTTTGTAATCCACTTGTGACAAATGTATGATGTGTTATCATACGTTATGATACAAATATCTGGGTGGATTTCCCTCATTCAGCTGAGGAAGTCACACTGCTATAGGCCTTCTATTTAATAA
>Bg-c26240 len=300 count=3 IPR:/ blastx_SP:/
ATCTAATAAATCAGTAGATCATCTTATTTCTAGTTGTTTATTGTTTCTTCAAGATAATATTTCAATAAGAGGTGATGTTAATAATTCATCATTGCTGATGAATCAAAGCCAGCATCCTTGTCAGTGTGTAACTAATATTTTTAACTCCAAAAACTGTTCATTAATTTGATCAGTGGATTAAACATTGTAGTGAAGAAAAGAATTCTTTTGTTTTTTTTTTTTTTAAATTTAAGAGTAGTTAGTTTAGTTAAGTTTAAGTTTACCGTACTTTTAGGTTTTTGGTAGTAGTCCGAACTTAAG
>Bg-c21704 len=282 count=4 IPR:/ blastx_SP:/
TAGTCATTAAAAATACCAGATTTACTTCTAGAACAGATAAAAGAGTGTACACCCATATTAGAGTAATCTAAATTGAGGATCTCATATATCTAGTCTAGTTTTTTTTAGTTAGATGTAGATCTATATCTTAACTGAATCTAACCATATGAAAATGTTTCTTTAACGAATGGCACTTAAGCAGTGATTGGAATTTTCCGAAAATGTCTCTGATCCATTTTTCTAATATAAAAATCCAATTTTTCCCCCGTTATTTATTAGACAAAATTAAAAAAATATTTTTAA
>Bg-c42621 len=152 count=2 IPR:/ blastx_SP:/
TTTCTTATTGCTTTAACACAGCCATCTACACTTCGCAAGCAAACACGCTAAACACGCTCCTTTAACCTTTCGACTCTTTCGTCCTACCCAGCATCCCAAAATTCAAACTTAAAAGCATCTAATAGTAAGTTAAAGCAATCCCTCTACCAGGA
>Bg-c32783 len=349 count=2 IPR:/ blastx_SP:/
TTAAAAAAAAAAGTTAAAGATTTGAACCAAAGAAGTAAAAAAAAGAAAAAGAATCATTACCAAACTTTACTAAAGAAAAAATAAAAGAATAAGAAAGAATACTTTGACTTATTTTTAAATTATTCACAATACATTTCTTCTAGAACTATTCTTGCAAGAATATAAATACAATGTTTTATGTAAATATCAGATCTGTATATAATACCTGTAGTTTCACAAAAATTAGTGATACTGCCACATGTTTATAGGAAGGCTGTTATTTGGTTAGTTAAACATATTACATGAATTTTCTTGCAAGAATAGTTCTAGAAGAAATGTATTGTGAATATTTAAAAATAAGTCAAAGTAT
>Bg-c9490 len=562 count=11 IPR:/ blastx_SP:/
CAATCCTGGACTGGTCCCTCAGAGGAGGCTGAAGGGTGACAAGTTAAAATTCAGAGCAGGTAAATCTGAAACTCCCCCATCAGCAGGAGCACCGTGAAATAATCCCTAACACTAATGCAAAAAGAGGTGGAGGAACTGAGGTGTATCCGTTGTGCTACAGCAATAGGAAAGAGGGAAGAGAAATCTAAATTATTGTGTAATGATCTAAAGGAGAAATCTCTAAATTATTTCTTGGTTTATATTCAAGTTCCTGACAAGTACTGACTACTAACTGAAGGAAAAATACTGAGCACGTTGCTTGAAGCAATATTTCTAGTTTCTTACACGTATCTTCTTAGTTGCCTTTGCCTGTTTCCTTGAAGTGCATTGTGCAGGGCTGGGAGAGGCCAGGAAGGATGAGATATGAACAGAGCTGGCTTGTGTCCATTGAGTTGTTTTATACTTTTTCCTCTGTATAATAATATTCATTGCTTTTTTTATTGTATTTTAACTCAATTTAAGTTGCTGGCCAATAGAAAAATAAAGCTCTGTACTTATTCTGTGTAGCAGAACCAAAGGGAAA
>Bg-c6150 len=455 count=19 IPR:IPR000237:GRIP blastx_SP:/
TTCGCATCAACTTGATTATCGCCTCCGCGAAGTAGAACAAGCAGCGCTTGTCAAGGAACTCGAACACCACAAGAAGACTGAAGCAATGAGCGAAGAAATCACAAAGTTACAAAATAAACTCTCGCTTCTCTCCACTGGTGGTGAAATGGAGTATTTGCGGAATATTTTTGTCCAGTTTATACAGTCGAACAATTCCTCAGCCAAGAAGAACATCCTAAAAGCGATGGGAATGGCGTTGAAATTAAGCGCTAATGAGATGAAATCGATTGAAAGTAAATGATCGATATTCGCACTGTACTCTATTAGTTTGTATAGGTACATATATGTACAATTTTATCAGCATATTATGCGACGATACTCACGGTTCGTGTTGTGATTCCCAGAAAAAAAGTGTGATACGGTGCAAGCTCTAAAAGTTCACTTAATGCTCTGCTTGTTTTAGAAAATAAATGTGT
>Bg-c37208 len=268 count=2 IPR:/ blastx_SP:/
TGTCGCAGAGTAGTATAGGGCACTTAATCAGGTGGGCGCTGAAGCTTCGTTTCACTCTGTAATATTTTCACTTCTACTACGTGACCAATTCCCTCCTATTTCATAATTTGGGAAGATCCACTATTTTGTGATTCGCCTTTAAGCTGCATTTTTCAGCATAATTTTAAATGTTTTCTTCTAAATACATGTAAATTGACTAGTGTTCAAAACAAGTTCCACTCAGTACCTAGATGCGATATAATCTGCTTTTACGGTATTACATGTTTAA
>Bg-c25413 len=343 count=3 IPR:/ blastx_SP:/
TGTTTTTGGCCTTTAAGTTTTAGTTTTCCACCTTTTACCATTGTAATTAGGAGTTCTTGCCATTTTTATGACTGTTTTAGTGATTACTATTTGTATGAACTGGATTTAGATCTAGGTTACCTAGAGTCTAGTTAGTAGATTCTCAGAGTGTCTCAGTGTGACTCTCACTGTCACTAGATTGAAGATTCTCATTACTCATTCTGATTTGAAATTATTCTAGTATATCTACTACTAGACTAATTTAGATCTAGAATCTAGATCTAAATCTAGACCATCTAGACTAGGTCGTATCAAATGTAAATCTAAATCTTAAGATCTCTATCTGGTAGGATCTAATGGTTAA
>Bg-c27529 len=264 count=3 IPR:/ blastx_SP:/
CAGGCCCCAACAAGTACAATGTGCAGGGGAATGCCGTGTGGGGGAAATGCAAAAAATTTCCGATGGGAATCAAAACTTTTACTTCTGACGTCAAGCCGTCACCCGGACCAGCAGCTTATAACTTACAGAGCCAGAAGCCTACGGGTCCAAAGTATTCTATGGGCAGCAAGGATGAGAATATCATCGGCGGACTGATGAGTAAGCTGGTCTTCATCCCAGATCCACGAAACCCAGGACCAGACTTCTGCCCGTCGTCTGATGAGT
>Bg-c12942 len=1023 count=7 IPR:/ blastx_SP:/
CTTTGTGCAGCCAGTCCTGTTCATACATTATAAAAGGACCCCAATGTTGTGTCATATTAAACAGGTCAATCCAGAATTGGTCATCATCAGGTACAGCCAGTCCACTGCCCTGGTCAGATAGAAACAAATATTTTCCACCATTGTATTGGGCATACGATGTTTGGTTGTCCCAAAAACGGTTATGGCAGGAAAACGGTACCTTGGTCTTGTTAGCTAAGTAACCGAATCCGTCAGGTATGGCGTCTGCTGTAGGAGTCCAGGTCAACGCGCCACCTACGTGACCTTTGTCGTACCACCAGGAGTCGTACTGAGTATATTGAAAAGGAATGTTCTCACTGTTGACATAATTCATAACATCCAGTATTGTAGTCTCATAGTTCTTGCCTTTCTCCGGGTTATAATAATAATAGGCACCATTATCAGTCCAATAGCCCATGTAGTTTAATGTTGTATCAAACTGTCTACGGAATGAATTCTTCTGATGCAGTGACAGTAAAAATTTGCCCCAATTTGTCATTGCCTGGTTGATGCCCCTGTTGCTGTAGTACACAATGAAATCAACGCTGTAATTGGCTGGCACGCTGTCAACAAGGCCCATAATTCCGTAATTTAATTCTTGACGAACAAGTGAAAGTTGGTTGGAGGCGGCCATAAACTGGGACATAGGGGAAATAACCAAAGTGTTACTGGAATTGTCGAAAATAACGAGTGGCCCTCCTTGTTTTCCTGTAGCAAATGATGCACTAGACTGCCATCTTCCCATGCTTAATCCACTGTATCCAGAGTGATAGCCTCCATAGGCTAAATATCCAAGATCTGTTGGACTTCCTGGAAATGTTTTGAAGCTGGGAAAACTGCTGATCACGTGGTCACTGTCACTGCCTGCTGCTCGATTGGCTGAGCCTGTGTATGTCTGAGAAAAAATCACCAGAGGCAAGTCTGGCATATTGTACGTCTTGAAATAAAGAATAGACTGAGACCGGAAGTGATCCGGCTAGGTAGTTGAAGCTCGTGCTCTGCCAC
>Bg-c16268 len=239 count=6 IPR:/ blastx_SP:/
TTCGCTGCTGTTGAATTTGTTGGAGATGGTACTGCTGCTGTTGCTGAAACTGTTGCTGGTGAAATTTCATTGGTTGTTGCTGGGTAATGGGTGGCTGTAAGCTAGGTGGAAGCACAGGGTTAAATGTGCCAGCTGAGGACTGTGGCACCATGACTAGGGTCTCCATCAGCGGTGGTGGGGGAACTCGAGTGTTAGCAATCATCTGTAATCCTGAACTATTTTCAGCATAGACATAACCA
>Bg-c13078 len=708 count=7 IPR:IPR001087:Lipase, GDSL; IPR013831:Esterase, SGNH hydrolase-type, subgroup blastx_SP:sp|Q503L4|IAH1_DANRE/Isoamyl acetate-hydrolyzing esterase 1 homolog OS=Danio rerio GN=iah1 PE=2 SV=2/9e-32/225-707/9-166 // sp|Q711G3|IAH1_RAT/Isoamyl acetate-hydrolyzing esterase 1 homolog OS=Rattus norvegicus GN=Iah1 PE=2 SV=2/2e-31/183-707/2-174 // sp|Q9DB29|IAH1_MOUSE/Isoamyl acetate-hydrolyzing esterase 1 homolog OS=Mus musculus GN=Iah1 PE=2 SV=1/2e-31/183-707/2-174 // sp|Q2TAA2|IAH1_HUMAN/Isoamyl acetate-hydrolyzing esterase 1 homolog OS=Homo sapiens GN=IAH1 PE=1 SV=1/2e-30/183-707/2-174 // sp|Q3SZ16|IAH1_BOVIN/Isoamyl acetate-hydrolyzing esterase 1 homolog OS=Bos taurus GN=IAH1 PE=2 SV=1/3e-29/225-707/15-174 // 
ATCCTTAATATCTATAGATTAGATCTAGAATCTATATGGGGATCAAAATATAGATTCTAGATCTATACTATATTTTATAGATTCTAGATTATTTTTTCTAAATCTAGATTTAAGCCTGGTTTTATCAGTTTTATAGGCTATATCTTTAGTTCTTTAGGATTTAGATCTACCCTAGACTCTAGAGCTTAATCAAAATGTCTGGACCTGGAGGAGCATTTTGTTCTTGGCCAAAAGTACTTCTCTTTGGTGCATCAATCACTGAATACAGTTTTAGCAAGGACGGCTGCTGGGGCACACTGTTGGCTGATTATTTGCAAAGAAAGTGTGATGTCATTGTCAGGGGCTTTGCTGGTTACACCACGCGGAATGGCAAGCAGGTGCTCCCTCATCTGCTGGATGCAAAACTTGCCAAAGATGTGGTTGCCATGACAGTACTTTTAGGGTCAAATGATGCAAATGACAAAGGTCGTAATCCATGGGAACATGTTTCTTTACAAGAATACGAGAATAATCTCAAGGAAATTGTGTCCTACGCTGTGTCTCAAGGAATCTCTAGTGACAAGATTATTCTGATTACTCCTCCTGCATTCCACATTGACCAATGGGCAGAATTTCATGGCTATTTGGGCTATTCTGATGGGGGCAGGAAGAATGAGCTAACATCCCAGTATTCACAAGCTTGTCTGAGAGTGGCCACTGAAATGGGAA
>Bg-c39141 len=252 count=2 IPR:/ blastx_SP:/
CACGAATTAAGCTTGTTGCTTTCTCTAAAGTTCTACCAGCGCTTTAAACCGATCTGACACCATGTTCTGTTTTCATAGAGATTAAATATATAAATTATTGTAGACAATTAAACATAACGACTAATTAGCAGAGACTTTGTGCTGATATAAGTAGACATTTTAAGACTATAATTTTATTACGCCACATCCTTTCAAGTACTTTTCACAAAAACATCCACAACAAATAATGACCCAATATAAACATTTCTACTA
>Bg-c24587 len=396 count=3 IPR:/ blastx_SP:/
GGAATAAAACCATGATGGCGGTAAGCTCCCAGCATGCTTCCTTCCACCAGGAAGTAAGACAGATTGAACGCAGACATGGCCGTGTGAAACACTTTGAAAGTTAGTAGCATCTGGTGCTGCCAGAAGCCCGTCATGGAATTTCTAAACCTAGAAAGACTGGAGTCTAGGTTGGACAGATCTTGTAAAGATGTCCCGTACACGTCCATGTGGGACTTCAAAGTCTGGAAGTAGGTATCGTCATAGACCTTCGTCTGGAAACTTTCAGTGGTCAGCTGGTCAAGATTAAGGCCATTGATAATTCTCTCCACAGAGTGGCATTGAACGGGAGTCTCCTCAACTGGGTAACTAGGATCTATCACGTACATTCTCAGTATCTGGTAGGGTGCTGAGTGGTCT
>Bg-c10213 len=539 count=10 IPR:/ blastx_SP:/
CAGTGGTAACAACGCATCCGACGCGGGGCCGAACACTTTTAATCATTCAACACTGGACGCCATCTTAGGCAGGAAGGACATGTTCACAACAAAGACCCATAACTCACAATGACTAAGGGCTATAACTTACATATCAGATATTAACATCCTTCCCTTTCTATTGACGAAACATATTCACTACAACATTCCAAAACGAACACATGGCATATTATCGTCCCTCTTTCACCACGAACAGAGGTACATTGTAGACAATAACCTTTTCACATACAGTGACACGATGATACTACGATACACCGATAACATTGTCAACAGACAACATTGGTATGTACACAACATTATAAACATGAGTATAACATATAATATACATAGAACATAATATACATCAATATCGAAGGCTATACAGATCTAGCAAAAAATGTAGCAAAAAAAAACAACAACAAAAAAACAAAACTTCAGGGGTTGTCACCACTTGCCCATGTCATAGTCCTTGTGCCACGTAGGTGTGGCTCTCGTTCGCCCACTCCTTCTCACGCTAGGCT
>Bg-c27043 len=275 count=3 IPR:/ blastx_SP:/
ATAACCATAATGTCAACATTAAGATTTTGTATTAGAAGATACATTCGTCTACAATTTCATTCAGTGAAAGCTGACAATGCCGGTTTTTTTTTATCGCACAAAGGATTGGCAACACAAAATGACAATTTGGTCTATTTTTCAGAAGATTTTTATGACTTTTCAGGATATTTTTTATTTCTGAATATCTCCAGAACATTTTGGTATATTTTGCAATTACATGAGATTTCCACTAGCCCCTAGAAAATCAGGAAGATGACGGAAAAAAACCTGTTCTA
>Bg-c6628 len=751 count=17 IPR:IPR014898:Zinc finger, C2H2, LYAR-type blastx_SP:sp|Q08288|LYAR_MOUSE/Cell growth-regulating nucleolar protein OS=Mus musculus GN=Lyar PE=1 SV=2/3e-45/121-648/1-157 // sp|Q6AYK5|LYAR_RAT/Cell growth-regulating nucleolar protein OS=Rattus norvegicus GN=Lyar PE=2 SV=1/3e-45/121-642/1-174 // sp|Q9NX58|LYAR_HUMAN/Cell growth-regulating nucleolar protein OS=Homo sapiens GN=LYAR PE=1 SV=2/3e-45/121-558/1-146 // sp|Q09464|YQ58_CAEEL/Uncharacterized protein C16C10.8 OS=Caenorhabditis elegans GN=C16C10.8 PE=2 SV=1/5e-33/121-543/1-139 // sp|P37263|YC16_YEAST/UPF0743 protein YCR087C-A OS=Saccharomyces cerevisiae GN=YCR087C-A PE=1 SV=1/9e-09/121-495/1-115 // 
GTGTCTACGTCAAAATCCATGCTTATAAATTAATGTCCACTTATTGATAAATTAGATCTAGAATAAGAATATTAGGACAATTAGGATCTCCAAACTATAACTATAAGACGAGAACAAACAATGGTATTTTTCAACTGCAATGCATGCGCCGAAGCGTTAAAGAAAAATCAAGTTGAAAAACACTTGCTGAGGTGTCGACAATGTAAAGTTTTATCATGTGTAGACTGTGGAAAAGATTTTTGGGGCAATGATTATCAGCAACACACAAAGTGCATGACAGAAAATGAAAAATACTGTGGTAAAGGATATGTGCCGAAAGTAAACAAGGGGGAAGTCAAACAAGAGCAGTGGATTGAGAAAGTACAAAAAGCTATAGAAGTTTCAGCTTCAAATACTAAATTGAAAGATATCTTAGAGAAACTGAAGGAATATCCTAACATTCCACGCAAGAAGCAGAAATTTGAAAATTTTCTCAAAAACAGTTTAAGATTTCATAACCAGGCATTAATCAATCAATTATGGGACGTGTTGATGTCTGCTGCTGTGGCCAACACTGTACAGAATGGTAAAAACACAACATGTGAGACAAAGACTGAAATCTCCCAAAATACAGAAACACTGAATCCTCCATCACATACAGAGGCTCCACCAAGAAAATAGATAAATACTAAACTGAGACAAACGAGAAGAAAAAGAAGAAAGGAAGGAAACTAAGCCAAACAAAAAAGGAAAAAAGGAAAAGAACAAAACG
>Bg-c1959 len=546 count=69 IPR:IPR000235:Ribosomal protein S7 blastx_SP:sp|P46782|RS5_HUMAN/40S ribosomal protein S5 OS=Homo sapiens GN=RPS5 PE=1 SV=4/3e-65/485-153/64-174 // sp|P46782|RS5_HUMAN/40S ribosomal protein S5 OS=Homo sapiens GN=RPS5 PE=1 SV=4/3e-65/178-62/166-204 // sp|Q5E988|RS5_BOVIN/40S ribosomal protein S5 OS=Bos taurus GN=RPS5 PE=2 SV=3/3e-65/485-153/64-174 // sp|Q5E988|RS5_BOVIN/40S ribosomal protein S5 OS=Bos taurus GN=RPS5 PE=2 SV=3/3e-65/178-62/166-204 // sp|P49041|RS5_CAEEL/40S ribosomal protein S5 OS=Caenorhabditis elegans GN=rps-5 PE=1 SV=1/4e-65/485-153/70-180 // 
ATAACACCTTTTATCTTATCAACAGTAACAGGATAAAAGGAGGGATAACAATATATACTTAGCGGTTGGACTTGGCGACACGTTCGAGTTCGTCTTTCTTTTTGATGGCATAGCTGTTAGATGATCCCTTAGCAGCATTAATAAGCTCGTCAAGAAAGACATTCCGCAATCGTCTTAATGTTGCGGAAGGCAGCCTCTCGAGCTCCATTACATAAAAGCCACATTGCCTGGTTAACTCTTCTAAGTGGAGCTACATCAACGGCCTGACGACGGACTGTTCCGGCGCGTCCAATACGAGTAGAGTCCTCACGGGGTCCACTGTTAATCACGGCATTAACAAGAACTTGCACAGGATTTTCACCAGTAAGTAAGTGGATGATCTCGAACGCATGCCTCACAATGCGCACAGTCATCAACTTCTTTCCATTGTTGCGACCATGCATCATCATTGAGTTGCACAGACGTTCAACAATTGGACATCCGGCCGTTTACGGTAAACCGGTACCTAACCTACGGTAACGGTACCGGCCAGAAATAGAGGAAAGG
>Bg-c26832 len=281 count=3 IPR:/ blastx_SP:/
AAAAAAAAGGGGGTAAATGTTTTGGACTTTTAAGTTACAGTTGTGATGGAATCGCAGTTTACAGTTAGTGAGTAATATTTCTCTGGGTGTGTGTGTGTTATAAAATAGATAAAAAGTTCTGAAGCAAAAGTTGAGGCTCTGTTGAGGTTGACAGAACTTGGGAAAGTCAATAGCATTGTTGCTGAAAAGATTTTATACAAACAGCCATGTCAGCTCTCCAGAGACTTAAAAGTTGAAATTGTTCAAGAGACAAAGAGTACTAGTTTTTACAGATGAAGTAA
>Bg-c39980 len=245 count=2 IPR:/ blastx_SP:/
ATATCTGTAATTTGTGTGTTACAGAAGTCAGCATTCTTTTATATTTTTACATACAAGTCACTTTTATTGAATCTAAGCTGCAATTTCCAATTATATATTATAAACGTATATTTTTAAAAATCATAATGGAACAACTAATGCATCCCGTGTATATTTATATTCAATGTTTGTTTATTGTAAAATCTGGTATCCTTAACAAAAAAAGAAAGATTATGTATGTTTAAAACATTAACTAATAAAACATG
>Bg-c25271 len=351 count=3 IPR:/ blastx_SP:/
TTTTATTTTTGAAACAAAAAAGTAGTGCTACCAAAAGTTAATTAAGCTACCTACAGTGCTAGTTTTGATGCTAACATCCTCCTAGAATTCAGCTTGAGAGTTTCATTCCTTTTTAACAAAAAACTAGCAGATTTAAATAGATATCAACAATCTCTTTGTTCTTTATTAGTTTGTGAGGTTTCATTTGGGCATCAGAGGACATGATCCTTGTTTTCAATGCTATTTTTTTTCTTTCTTAGTCAGCTAGCTCTTATTTCCCAATGTTTACAAATTATGTTTTTCTTTCTTAGTCAGCTAGCTCTTATTACCCAATGTAAACAAATTATGTTTTTCTTTCTTAGTCAATTTTAG
>Bg-c33386 len=328 count=2 IPR:/ blastx_SP:/
CGACAGNGCCCCCTCGTGAGCAAAAGAAGGCTGGCGGTAAGAAGCATATGTTTTTACCGGTAAAGAGATCGCAGCTCGGCGAAGGCAACAAACACAGTGAAGCAGGCAAGCAGAGAGAAGAGAGAGATAGGCAAGAGAACAGCCACTACGATATTTTCAGCGCCGCTCCGTTTGCTCGTGTCTCTGTTTAGTTTGAGAAGGAAATCTTCCAGGCAAATGCTGACATTGCCGTCCAAGTCCATGGTATACCTGGACCGGTAAACTACTGAGTCGGGATCACCCCAGTGAAGGCCAATGCTGCCGCCTGGTCCTGGTACCGTGTACTCGG
>Bg-c13040 len=742 count=7 IPR:/ blastx_SP:/
CTAGCAGCGGGGTGGGACCCTGAGAGTGTCATGTCAGGTTCATGAACATTTGCAAGATGGGATGTCGGTGACGAAAGATTTCCGTGTGTATGTGTGTGTTCCTATGGCGACGGTGGGAAGAGGTTAGCGATTGGTTGTGAATGGTGCAACATAGGTGGCATTGTCGTCATTTGGTCTTAGTAGGTGAGACGACTTCAGAACGTGAGACTGATAAGTTGTGTTATTGTTTTTGTTGTTTGGTCGCTTCTTTACATCAGAATAAATCCTAGAAATATCTCCCTTGTCGAGTTGCACTGCTCCTCTCCTGTATTTTCCTGGCATAACCTGTAGTGTTGATTCGATGTGTGGTTTTGGTTTTGACTGTTTATGGGCTATAACAGGTACTCTGGTAAATTTTGGTAATCCATCATGTTTTCTCTGTGAAAATCTTAACAGCCTCTGCTGATTCTATATTGGTTCTTTCAAGTTGTCTTCTTTTTAGTTCTGGGTATGGTATAGATACCTTCTTTGGCTTGATGCCTAATGATTGCCTCCACCTGTGTTTTGCAGCAGCAGCAGCCTCCCCCTCAAGCCCCTTTTCACCAAAACTAATAACATCACGGAAAAATATTTTGTCTTTGCTTTTGTGAGTCTGTCATCTCAAACTTTTTGAATCGCTTAGCATCAATTTTAGTTTGAGCACCTAATCCCCCACTNTTTATCAATTGATTTATTGAGNGCCTCCTTTCTGCTATTGTCACCG
>Bg-c6467 len=387 count=18 IPR:/ blastx_SP:sp|Q28DT7|EED_XENTR/Polycomb protein eed OS=Xenopus tropicalis GN=eed PE=2 SV=1/2e-43/1-384/272-399 // sp|Q6AZS2|EEDB_XENLA/Polycomb protein eed-B OS=Xenopus laevis GN=eed-b PE=1 SV=2/2e-43/1-384/272-399 // sp|Q8UUP2|EEDA_XENLA/Polycomb protein eed-A OS=Xenopus laevis GN=eed-a PE=1 SV=1/2e-43/1-384/272-399 // sp|Q921E6|EED_MOUSE/Polycomb protein EED OS=Mus musculus GN=Eed PE=1 SV=1/3e-43/1-384/275-402 // sp|O75530|EED_HUMAN/Polycomb protein EED OS=Homo sapiens GN=EED PE=1 SV=2/3e-43/1-384/275-402 // 
CAAGAGTCTCATTATTATACCACATCAAAACAAGATGAGCCTTTCCCAACTGAATACTGTCACTTCCCAGAATTCTCCACTAGAGATATTCATAGAAACTATGTTGATTGTGTCAGATGGTTGGGAAATCTTGTTTTGTCAAAGTCATGTGAGAACTGTATAGTGTGTTGGAGACCAGGCAGTTCTCAGGATGCAGTCAAGATCAGAACAGATGAGAAAGAAAGTGTTTGGATATTACATAGATTTGAATATAAAGAATGTGAGATCTGGTACATGAGATTTTCACTGGACTACAATTTACAGATGATGGCTCTAGGGAATCAAGTTGGCCACATATTTGTTTGGGACCTAGAAGTAGATGATCCAACTCAGACAAAATTCACCAAA
>Bg-c32464 len=362 count=2 IPR:/ blastx_SP:/
TTTGAAAAAAAAAAAATTTTACTAGAGAGTGTATTTAATTCTTACCCAAGACAAGAGAGTCAACTTACCCATATGTGACATCTACACAGAAAGTAGATTTGAACACTAGCATGCCCACTGTCATTACTAAAATGCCAATGACAGGTCATTAAACAAATATATGTAATGGGAATGAACTGTAAGTAGTGTCAAATATAAATTTATTTTTTTTAAATCACCATCACCACCTCATTGTAAATTTTCTTTATGTTACATCAGGCTATGCTAACAATTAATTTCAATAATAAAAAATAGTGTNTTTAGTACAACAAATTAAATAGCAGAAGAAAACAAGTAAAATAATTGATTCTTAACTTAAGAAA
>Bg-c18978 len=207 count=5 IPR:/ blastx_SP:/
AAACATATACACGCATGAGAAAAGGAAAATAATTTTTCATGACTATTATTTTCTTTTTTGTAAAGTAATTATGTATATGTAAATTATGTTTAAAATTTTCTCTTTAATTCTGTATGATTTAATATTAGTGAAATGCGAAACTTTACAAATAAAATTGAAAAAAATATACTAATAAATAAATAAATAAAAAGCTCCCCCCCCAAAAGA
>Bg-c23169 len=688 count=3 IPR:IPR011990:Tetratricopeptide-like helical blastx_SP:sp|Q8N0Z6|TTC5_HUMAN/Tetratricopeptide repeat protein 5 OS=Homo sapiens GN=TTC5 PE=1 SV=2/4e-48/28-375/7-122 // sp|Q8N0Z6|TTC5_HUMAN/Tetratricopeptide repeat protein 5 OS=Homo sapiens GN=TTC5 PE=1 SV=2/4e-48/438-686/142-226 // sp|Q0P5H9|TTC5_BOVIN/Tetratricopeptide repeat protein 5 OS=Bos taurus GN=TTC5 PE=2 SV=1/5e-48/28-375/7-122 // sp|Q0P5H9|TTC5_BOVIN/Tetratricopeptide repeat protein 5 OS=Bos taurus GN=TTC5 PE=2 SV=1/5e-48/438-686/142-226 // sp|Q5BK48|TTC5_RAT/Tetratricopeptide repeat protein 5 OS=Rattus norvegicus GN=Ttc5 PE=2 SV=1/1e-47/28-375/7-122 // 
GACATTGTTTAGNTAATGGCTGCCGGCGAAAGAGTTCCAAGCCCAACAGACAAAGCTCTTACCATCGTTGAAGAGTTGTACAATTTTCGAGACCATTACATTGAAAGTCATGGGATGGAAAATGCTGGGAATAAAGAGACAGATGTTCAAAATAAAATGAATGAAAGTTTGGCACAATTAGAGGAAGTCAAAGGAGACATAAAAAACAAAGCACAGTATCATTTACTCCGAGGGAAGATCTTAAATATCACATCCAAATATAATCAGGAAGCTGAGGAGTCGTTGAGCAAAGCTGTGAAATTAGATCCTAGACTCGTTGAAGCTTGGAATCACTTGGGGGAGTGCTACTGGAAGAAAGAAGATATTTCTGCTGCCAAAAAATTGGTTTTACTGGTGCCTTTAAACCATTTAAAGGAATAAAGTCTCATTAAGGAATCCTTTCAATGGTGCTAAGACAATTGAGTGGTTCGCCAAGAGAAAAAATCAAATTGATTGAAGAAAGTGTTGAGAGAGCCAAGGAGGCTGTGCATCTTGATATTACTGATGGGACTTCTTGGTTAATTTTAGGCAATGCTTACTTGTGTCAGTTCTTTACTGCTGGTCAGAATCCCAAAGTCTTGAAACAATGTATGCAAGCTTATTTACAAGCAGAAAAAGATAGTGTGACAAGAGGCAACCCAGACCTACA
>Bg-c11459 len=338 count=9 IPR:/ blastx_SP:/
ACCCAGCACTCATCGTTTACAGCATGGACTACCAGGGTATCTAATCCTGTTTGCTCCCCATGCTTTCGCGTCTCAGCGTCAGTATCTGCCCAGAGAGCCGCCTTCGCCACTGGTGTTCCTCCCGATATCTACGAATTTCACCTCTACACCGAGAATTCCGCTCTCCTCTTCAGTACTCAAGCTTCCCAGTATCATGTGCACTTCCGAGGTTGAGCCCCGGGCTTTCACACCTGACTTAGGAAACCGCCTACACGCGCTTTACGCCCAGTAATTCCGAGCAACGTTTGCACCCTCTGTCTTACCGCGGCTGCTGGCACAGAGTTAGCCGGTGCTTGCTA
>Bg-c42842 len=132 count=2 IPR:/ blastx_SP:/
ATTTCTCTTTATTGTTGTGTTGGACTAAACTCACACAAGTCACCTCAGGAAGTAATAAACTAGTCAGTATGATTTTGTTAATCTTAAACCATTTATGTATAATTTTATTTCTCTTTTTGAGTTCATGCTGGC
>Bg-c27576 len=263 count=3 IPR:/ blastx_SP:/
AACACAACTACAAAAACATAAAGAGAAAGTTTTCTTTTTAAAAATTACTTCATGTAGTTCTGACATGGCTATCACTCAAACTATTTATCAATTTATTAATCTGGAGCTGTGGATGATGTGAGCACAAAGCTAAATGTTTCCCATCTTTAATCCATATCTGTATTTTGATAGATGGTTTTTTTTTCTCTCTAGATTTAGTTTTTACAAACTGTTTGAATTCAATTGCTGTATACAAAAGTATATGTGATGAACGTAAGGACTCC
>Bg-c31401 len=414 count=2 IPR:/ blastx_SP:/
GGATGCGTGGGATGTGTAACTTTTGGTGTTCGTGTTTTGTTGGATGGGTGGGATGTGAACTTTCTGTTCTCGTGTTTTGTTGGATGGTGGGATGTGAACTTTATGTTCTCGTGTTTTGTTGGAGGAGGAAGCTGGAACGAATGCAATAATACTGGCGTGTGAGTGTGAGTGAGTGAGTGAGTGAGTGGTAGATGTTAAAATAGAGAGAGAAAAATTGTGACAAAGTGAGAGATATTGACACATAACCCTAACGACCTAGAGCTATAAATGTAAAGTTTTCATCGTATTTTATCTATTGAAATATTTTGTCATTTAACAAATAAAAAAAAAATTAAAAAAGTAAAAAAAGTAAAGGAAAATAACTAATTTTAAATTTAACTAATATAGCACGACTAGTTTTTTCTTTTAACTTAA
>Bg-c9861 len=261 count=11 IPR:/ blastx_SP:/
TATAAAAATATATGTAATCAAGTCTTTGTTGTTGTTTTTCTGGTATGTTTATTTTGCATAATCTATTTCGTCCCTTTTTAAACGTATAATTAATCCATTTCAGTCAATAAGTAATTGAATGTCATTTGATATCCTATTGTGAATGCTGTTTTCTTATAGTCATTGAGATATCTTGAAGATGTCATGCAGATACGGCCAGCGGGCCAGATCCGACCCGCGACGTGCTTCCCTCCGGTCCGCAGAAACGTCGGCACAAGAGAG
>Bg-c26145 len=304 count=3 IPR:/ blastx_SP:/
AAAACATACCTGCATTCATCCTTTTGACAAGACATATTAATTATTATATAGTTTGTCCATCATGGTTCGATGATGACCACTTTTGTCATCCAGGGGGCTGAGGGCTTTGTGCTTCCTCGTGGCTGGTGAAACCTATGTGAGCCTGGAATTTTTGGCTGCACACTGGGCAGGTTATTCAGTTATTGTCATTGGCCTTGCTTTTTTTTCCTGCTAACACAGACATAATGTGTAACTAAATTAGGTACTTTTTTGGTTTTTATCCCCATTGTGTTGTCTTTTGCTGAGGTTTCTCTGCTGTACATAA
>Bg-c28169 len=250 count=3 IPR:/ blastx_SP:/
TTATTGTCTTGCAACTCTTGAAATATTAATAAACAAACTATATTTTGAAAATTATTGCAAGAGCTACTAAGGGAGAGAGTTGGTAACAAATATCAGGCTTTCAGTGACATATAGATATTGTCAAGAATTGGCTGGCTGGTCGTGCAGTATGTGTGCTGAACATTCGTTCAGACGTTGATTGTTAGCGGGTTCATACACTGCCTGCTGCCATCCCCTGTCATGTTGGCGGGAGGTTTGGACAAGGAAGTAG
>Bg-c25590 len=332 count=3 IPR:/ blastx_SP:/
TATGAGCTACCAAAAAGACCCTCATTTTAACAATGCCCCCTAAAATACTTTTTAGCCCATCAATTCTAGTATGTTACATAGACTTTTAACAGCTTAATCTCTCTTTTCTTATCTCTCCTTCTGTTTGTCCTTCCCCCCCCCCCTCTCTCTCTTTGAACTCCCTTTTTATTTTTTATTCACTGCACATTGTAATGCTCTCCGACAGACTTTTGACAAGCATTTTAATAACCAGCTTTGTAAACCCACCCNCCAAAGCCACCACTCCCCGGCCTCTTCCAAATCCTCCTCTAAATTCAAACTTTCTGAGCCAGCACCAACTTCAGATTTCTTGT
>Bg-c23231 len=667 count=3 IPR:/ blastx_SP:/
AAAGACATTTGTAAAAATAAAGTGTATTATGCTAAAAAAAAATAAAAAAATTAGTACATATGGCACTCTGTCACACTGCCACATCATAGGGGCCCTAAATGTTGTTATGCAGATGATGAAAAAAAAAAGCCACATCATCTTCAACCTATATGGTTACAATGAGTTTCCCTAAAGACTGCAATCTTACTACATACTCTGCAGTGAAGGCAATGCATTAATTTTTTTAGTTTTTGTTTTGTGAGATGTTCCTGTCAAAGTTTTTATATCAACTGCAGACAAGCACTAATTTGGTGACAAAAACAAAAGATTCAGAAAAAATTCCAGAACCGATGCAATCAAAAAGTGAACACAAAATATGAACACAGGAAACATGACACCAGTGTTCAACACATCGGGTAACATCATACAATACATTATAGAATACATCTGTATTACAATACACTGGCAATACAATTTATTATCATACAATACACAATTAACACAAGTGAAATACTAGTTTTTATAAATGGTAGACTCACAGAATATTGTATAAAATAAATAGATGAGCAAAAAAGATGTATATAAATATTTTTAAATGACATTTTTAAGAGCCTTGAGATTGTGTGACCAGAACTGACCACCCCTTAAAGGGCCAAATGAGTCTCCATACATTGAACCTGAGATGAAC
>Bg-c12363 len=423 count=8 IPR:IPR017868:Filamin/ABP280 repeat-like; IPR001298:Filamin/ABP280 repeat; IPR013783:Immunoglobulin-like fold blastx_SP:sp|P21333|FLNA_HUMAN/Filamin-A OS=Homo sapiens GN=FLNA PE=1 SV=4/1e-17/2-418/2377-2517 // sp|P21333|FLNA_HUMAN/Filamin-A OS=Homo sapiens GN=FLNA PE=1 SV=4/5e-15/20-418/2002-2135 // sp|P21333|FLNA_HUMAN/Filamin-A OS=Homo sapiens GN=FLNA PE=1 SV=4/2e-14/23-415/1313-1442 // sp|P21333|FLNA_HUMAN/Filamin-A OS=Homo sapiens GN=FLNA PE=1 SV=4/9e-14/2-403/527-659 // sp|P21333|FLNA_HUMAN/Filamin-A OS=Homo sapiens GN=FLNA PE=1 SV=4/9e-14/29-415/1408-1539 // 
TCAGGTTTTTAAAACTCCTTTGGGTGGAGGAAAGTACAGATGTACTTACATACCTGTTATCCCCGGTGCTTATCTACTTAATATCTCCTGGAATGGTCGACAGCTACGAGGAGCCCCATACAAAGTGAATGTCATTGGTGCCTCTTATCCAAATCGTGTCATAGTAAATGGTGAAGGCCTCAAAGGTGGATTGATGGGTAACAGTTTAGATTTCAGAATAGATACCAGGAAAGCTGGACCAGGTGAATTGACGGCTTACTGTATGGGGCCAAACAAAGTAGCTTACTGTGAGCTCAGTGATCATCATGATGGCACCTACAGGCTGGTAGTTAGGCCACAGGAAACTGGTAAACATGTGTTACAGATCAAATATGGTGGAGAACATGTGCAAGGAAGCCCATATGCTTTCAAAGTAACAGCACA
>Bg-c18125 len=306 count=5 IPR:/ blastx_SP:/
AACAGTGAGCTCATCTGACTGTTTTAGGATGTACAGCCTAGTGTTAACATTCTTGGTATCTTTTTGTGCCTTATCTAAAACATTTCTCGATCACTGGTTTCAGATGTTCATTTATTATATTCTTTTCAAAGATTCAGAGATTGGCTTTTGTCATCCACTATTGTATGTTTTGTTTCATTGACCTCTAGTGATACCTTGATCTTTCCCACTTTCTGTTTTCGGATTGGAGAAGATGTTACCTTTTTTGTCAACTCTTACTTTTATCAGATGATCAACTCACGTATTTGGATCTTTATTTGTTTTCTC
>Bg-c20708 len=368 count=4 IPR:IPR012348:Ribonucleotide reductase-related; IPR000358:Ribonucleotide reductase blastx_SP:sp|Q4KLN6|RIR2_RAT/Ribonucleoside-diphosphate reductase subunit M2 OS=Rattus norvegicus GN=Rrm2 PE=2 SV=1/3e-21/268-366/96-128 // sp|Q4KLN6|RIR2_RAT/Ribonucleoside-diphosphate reductase subunit M2 OS=Rattus norvegicus GN=Rrm2 PE=2 SV=1/3e-21/143-274/55-98 // sp|P79733|RIR2_DANRE/Ribonucleoside-diphosphate reductase subunit M2 OS=Danio rerio GN=rrm2 PE=1 SV=1/9e-21/268-366/92-124 // sp|P79733|RIR2_DANRE/Ribonucleoside-diphosphate reductase subunit M2 OS=Danio rerio GN=rrm2 PE=1 SV=1/9e-21/158-274/56-94 // sp|Q4R7Q7|RIR2_MACFA/Ribonucleoside-diphosphate reductase subunit M2 OS=Macaca fascicularis GN=RRM2 PE=2 SV=1/2e-20/268-366/95-127 // 
GTTATGAAAGATTTAGTCGCATGTTCTGCGAATGATTTATATTTGTTACATTATGCTGGAGTATTTTCACAAATATTTGAAAAACTTTTTAGTATTGTGTCCTCCATTTTTATTGAAATTTGGTTATAACTTCTGCCCACAGTCTTCCCAGCTGACAAAAACAGAAATTCGCAAAAGAGAGGCCCAGGAGGAGCCTTTGTTAAAAGAAAACCCCGGGCGATTTGTTTTATTTCCAATTCAGTACCATGACATTTGGCAGATGTAACAAAAAAGCTAGAAGCATCATTCTGGACAGCAGAAGAGGTGGACTTGTCTAAAGATCTTGATCACTGGGAGAAGCTGAAACCAGAAGAAAAGCATTTTATCTC
>Bg-c217 len=1237 count=314 IPR:IPR001299:Ependymin blastx_SP:sp|Q5XII0|EPDR1_RAT/Mammalian ependymin-related protein 1 OS=Rattus norvegicus GN=Epdr1 PE=2 SV=1/6e-18/140-667/39-214 // sp|Q99M71|EPDR1_MOUSE/Mammalian ependymin-related protein 1 OS=Mus musculus GN=Epdr1 PE=2 SV=1/1e-17/140-688/39-220 // sp|Q9UM22|EPDR1_HUMAN/Mammalian ependymin-related protein 1 OS=Homo sapiens GN=EPDR1 PE=1 SV=2/2e-17/140-667/39-214 // sp|A6QLI0|EPDR1_BOVIN/Mammalian ependymin-related protein 1 OS=Bos taurus GN=EPDR1 PE=2 SV=1/2e-17/149-691/54-233 // sp|Q9N0C7|EPDR1_MACFA/Mammalian ependymin-related protein 1 OS=Macaca fascicularis GN=EPDR1 PE=2 SV=3/2e-17/140-667/39-214 // 
TTATATATGATCAGGGTGTCTGAGGCCTGGGCGACTCACTTCTCAGGTTTAGGTTTCACGAGCGGGTCTTGTCGATTCAAGCATGTACAGTGTATTAGCACTCACCCTTCTAGGGGCTGCTGTGGTCTACGGCCAAGTCCCCCACCACTGTTTCTCACCCCCACAGTTGACGTTCCGTGCCACTCAATACAACCATGAGTTTACAACGTTCAGACAGTTCGATGCTGAGTACGATGCCATGGGAGAGAAGATTGCCTTCGATGAAGTAGAACAATCTGGACCTGCCCCAGGAAGACAATATTACCGTATCATCATCCTGCACAGAGAGAACATTGCCTACGAGTACAACCGTCAGACCAAGACCTGCAAGAAAGGACAAGCCGGACCATTCTTCCCATTCGCTGTTCCTGAGAATGCTACCTTCGAGGCCGAGTTCTACGTTGGTGGACCAGGGGAGGAGGTAGAGGCTGTTGAATGGTCAGACCGCAGTGCAACTGCAAGGGAAGCCTGGGTTGGAGTCTTCAGTAGATTCAACTGCTACCCTCTTCGTTCATTCTTCCTCAACGGACGCAACAATGAAACCTTGACCACTCAGTACTTTGACTTGGTCCAGGGCATCGTTGATCCTCAGTTGTTTATCCCCCCACAGGAGTGTCTCCAGGCTGAGAAAACAGAAGGCATCTCTGACCAGGTTGCAAGACTGACCTCAATGTACACTCGCAGATTCCAATAAACTACTCACAGACGGTGTGATTGACTAAATGTTGACTGCATTTAACTCTTTATATTAGTATGTTGTGTGTCACCTTGTAAGTTGGAGATTGTTCAAGTAGTATATCAATATCTTTTATATCAACTGTAGGCTTCAACATGAACATCTTGTTAAAGTTGACAGTTTTATTTTGTTTTCTTAAAATAATGAATGTGTGTTTTGAATAAACTTTGATTCATACAAAATAAAAATGTGAAACACAAATCTATATCTATCAGCATCTATCTAAATATAATAAAGACATCATCTTCAATTCTGAAGATTAAGGATGAGTGTAATCTTTCCTGAGGCCACACTACTATGTTGCATAAACTAAATGATGCAAAGAAATAAAAACAATTGTCCTCCAGGATCTAAAAAAAGAGACAATAACAACACAGTCACCACTTGTGTTATATTAAAACAAATTGTATTCAGAAGAAAAACAAACTAAAATGTAAATAAAAGATATGTTTAAAATAAAAA
>Bg-c34719 len=297 count=2 IPR:/ blastx_SP:/
TGTCAGTTAGTTAGTCTTAGACTTACCTTAATGAGATCAAAGTAACTGTTTTACTGTGTCTGAAAGTGTTATTTTAATATATAGTGTATCAAGGCGTATCTGTATGTCATTATGATGTTCAATGTTTTTGTGTCATGCTAGTTAATTTTTATCTTTGTCAGTGTTTCTTTGTGTCCCACAATGTATTATTGACTCAAATACACTTTGCAATAATCTAAACTTTATTCCAGTATTGCATTCCTTACGACTTAGATTTTGAGAAATAAAAGAAATAAAATTCATCAATTTTGATTTGTA
>Bg-c5671 len=438 count=21 IPR:/ blastx_SP:/
GCATGGTAACAACGCATCCGACGCGGGGTCCTAGTGTAGCAATGTTCAAGTCAGTCTGTCCTAGTGTAGCAATGTTCAAGTCACTCTGTCCTAGTGTAGCAATGCAAAATATTTTTGTAGAATCCTAAAATTGTTTTTAATGACCTGAAGTGACATTCATGACCATGAAAACCAGAGCTTCTTGCCCAGAGTTGGATTATAAAATATACGTCTTTGCAGCTCAGGTAGGTCCTGGACTTAGACAATAAGTTTTTCATGTTCATCACTGAATGTCTCCTTGGAAACTATACACACATTTTGACACACATTTTGTCAATATCAGATAACTTTGAAGGGACCTATGTGTTTTCTATTGTTAAAGTTACTTATTGTTTTTATTTCAGAATGTTTAATTAGTATTAGTAATACTAAATTAGGTAAATAAAAGTTTAAATTAGT
>Bg-c17456 len=402 count=5 IPR:/ blastx_SP:/
ACTATTTTGGTGAAACCACAGAACCCGAAGACACTGCGAAGACCTTGGATGAAGCCACAGAGAAAACAGAAGTAAGTAGACAATGAGATAGAGTTTTGGGTGCAAAATGTTTATTAGGGATCAACCCCCAGGAGAGGAAAAGGGTGGAAGGAGAACTGAGCAGAGGGAGAAATCACACTGTGATGTGGACTGACAAGGCCTCAGCCAACCCAGCAGGGAGCTCAGAGGAAAGAGTAACCTTTCAGAGTGTCCTGCACTGGGCTGAAACGGGTGGCCAGGTCTGTCTATCCCTGCCACGCTCAGTTGCAGAAGGCAGGTATTCCATAGAACATGAGTTCAGGGGAAGCAGTTCTCTGCAGCTGAGACAGATCTAGAAAAGAACTGACAGGTAGAGGCTGTGTG
>Bg-c36084 len=279 count=2 IPR:/ blastx_SP:sp|Q5XI03|CCD45_RAT/Coiled-coil domain-containing protein 45 OS=Rattus norvegicus GN=Ccdc45 PE=2 SV=1/6e-16/9-227/653-723 // sp|Q8BVV7|CCD45_MOUSE/Coiled-coil domain-containing protein 45 OS=Mus musculus GN=Ccdc45 PE=2 SV=1/3e-15/9-227/659-729 // sp|Q96GE4|CCD45_HUMAN/Coiled-coil domain-containing protein 45 OS=Homo sapiens GN=CCDC45 PE=1 SV=1/7e-14/9-218/658-727 // 
AAATGCATGAAAAGAGGATGCAGTCTGCACGTGCCAGACGCTACTACAATGAATATCAAGTCCGTGCAAGGTCAAAACAATTAAAAAAGAGAACAAAAGAAGAAATGGTTTTCCGTGATTTGTTCAAAACTGCATTAAATATCCAGAAAGAAAGACTGAAAGATATTCGTCATTATGCCAATGATTGTCGAAAACGTCAGGAAATTCAGCGCCAAAATAGAAATAAGAATCATTAAGTAAAATTTTTAACCTAAACGAACCCAAACTTTACCCTAGGAT
>Bg-c25766 len=323 count=3 IPR:/ blastx_SP:/
ACTTGGGGACCTGATTGAAAACCCACATCACAGGAAAGGTGTGAACCTTACATTGGAAAACAAATTTTCAGCCTTGCAAGATTGACGCTTGTACAATTAGAAGAACTAATTGATTTCTGTGTTACGACCGACTGAGCTTTCATGCTGACTAGTGCTCTGCCTTGAAATCAACGCGACGTCTGCTCCATGGGCTAGTTGTCATCTGCCATGTGGTCTGGCTAATCTCTTTTATCTATTTGTAAACTGTCTGTTTATGTTTGAAATTGGTAAATTTTTTTTAAACATTCATTAGAATGCTATTGTTGGGAAAACTGACTTCCGAG
>Bg-c3964 len=1401 count=31 IPR:IPR001464:Annexin; IPR018502:Annexin repeat blastx_SP:sp|P22464|ANXB9_DROME/Annexin-B9 OS=Drosophila melanogaster GN=AnnIX PE=1 SV=2/3e-65/1258-830/36-178 // sp|P22464|ANXB9_DROME/Annexin-B9 OS=Drosophila melanogaster GN=AnnIX PE=1 SV=2/3e-65/824-402/181-321 // sp|P22464|ANXB9_DROME/Annexin-B9 OS=Drosophila melanogaster GN=AnnIX PE=1 SV=2/1e-09/788-396/37-164 // sp|P22464|ANXB9_DROME/Annexin-B9 OS=Drosophila melanogaster GN=AnnIX PE=1 SV=2/1e-09/821-396/99-248 // sp|P22464|ANXB9_DROME/Annexin-B9 OS=Drosophila melanogaster GN=AnnIX PE=1 SV=2/1e-09/1072-875/26-91 // 
CTATTGCTTTTGAATGTAAAGATTATTCTTTAACAAAGGAAAATATAATACAGGATTACATCAGGTTCCAAACTTGTACTACAAAAATAAAAAAAAAAGTTACTGTGAAGCAAACATGGTATATACAATCTAAAATTGTTTTGTTATAAAGGAACAAAATCCTTTTAGAATCATGTGTACAATAAAAGTTGTAGACATGACTCTATTGGAAAATTATACTTAATGAATTTAAAAAATTAAATGCAAAATAATAAACAAAATTGGAATATAAAAATAAAACAGAACGCTGAGCTTGAAATACTTGTTTAAAAAAAAACCCAGACATAATCCAATGTCAAGTTCAGACAAGCTGGTTTAGTGAGAAGATTGTCATGTGATGCTGCTCAGTCTTGCATCTTGACAAGTGCAATGAGCATCTTCTTGTAGTCTCCTCCACACTCACTTTCTATGGCTTCAATCAGACTGACACCATACATAGCTTTATAGGCTCTTTCTATTTCCTCCATATCAATCTCACATCTGTTAACAATTATTCTAATGAGATCCCTGTCATTAGTTCCTAGACCCTTAAAACATTCATTGAGTCTTCTGGCAAAATAGGCTGGTGTGTTCATTACGCTGTCAACAATGGCCAAGTAGCCTTCTTGTAGACTGCCACTGCATTCAGATTTAATTGAGTCCTCAAAGCTTTTACCAGCAATTTTAGCAAACTCTTTGAAGGTTTCTCTCAGCTGGGAATGGCTTCGTAGACACAGAATAGCATTGAGCTCAGCTTCATCAGTGCCCCAACGAGCCTCACCAGCATCAAATAGTTGTTGTGCATCTTTTTAGCTCGACCACTGTCCACAGCAGCCTCTGGCTCACGCCCTGCAGCCATGAGTGAGATCATCAGACGTCGGAAATATCCTGACGTATCTCCACACAAATCATCCTCAAGGTTAGAGCCATATTCTGCTTTATATGCTGCCTTAATTTCTTCTATCTCTTTATTGGTTTTTGTCACTAAAATTTCAACGATGCTTGTTTCATCAGTGCCAGCTCCCGAAACAGCCTTATGAAGCTGCTGGGCATCCAGCTTTCTAGGAGGAGTCAACAGAGCAAGACACACTTCCTCAAAGTCGCCTGTCAGTTCTGACTTAATGTCACTAAGTAAATCCTTTCCATAGGCCGTTTTATATGCCTGGGCGATTTCAATTCTTTGTTGACAGTTATGGTTTCCAAGAATGTGAATAATAGCAGCTTCATCAGTGCCGAATCCTGCCATAGCACTCCTGAGCTTTGCAGCAGCATCTGCAGCCTTGAAGTCTGGACGATGCTTGACTGAGCCGGGCATTCTTGATTTGTTACAGGGGAGATAAACCAAAACGAGTGCAGTGCAGTCTGACTAGATTGTACGATCGC
>Bg-c15769 len=310 count=6 IPR:/ blastx_SP:/
GTTCTGGGTTTTTTTCTTGTACATTGGAATTGTTGAGTATTTTGATGCTTTAGACTTTTTTATTTTTATACTTTTTCATAGTTTAAATGAACAACAAAATTTATTAGCCAGTTTTTTATTCGTTGATTTGTCTGTCTGTCCGATTTTCTTCTTTCTCTACATTTTTGTCTTTCTTTACATTGGTTTATTCTTATTCGTTCTTCAATGTTTGAACGTTTAAAATATTTGTACGACTTTGTATACAACAACCGAAAGAATTCCAGTCTTAAACCAGCACGCCAGTCCGAGTTGTTCACTGTCATATTCAAAA
>Bg-c38268 len=259 count=2 IPR:IPR004273:Dynein heavy chain blastx_SP:sp|P37276|DYHC_DROME/Dynein heavy chain, cytoplasmic OS=Drosophila melanogaster GN=Dhc64C PE=2 SV=2/3e-36/256-11/4182-4263 // sp|P38650|DYHC1_RAT/Cytoplasmic dynein 1 heavy chain 1 OS=Rattus norvegicus GN=Dync1h1 PE=1 SV=1/8e-29/256-11/4196-4277 // sp|Q9JHU4|DYHC1_MOUSE/Cytoplasmic dynein 1 heavy chain 1 OS=Mus musculus GN=Dync1h1 PE=1 SV=1/8e-29/256-11/4196-4277 // sp|Q14204|DYHC1_HUMAN/Cytoplasmic dynein 1 heavy chain 1 OS=Homo sapiens GN=DYNC1H1 PE=1 SV=5/8e-29/256-11/4198-4279 // sp|Q19020|DYHC_CAEEL/Dynein heavy chain, cytoplasmic OS=Caenorhabditis elegans GN=dhc-1 PE=2 SV=1/3e-27/259-8/4145-4228 // 
AATTCTTCCTTTCAAAACTCTTGGGGGTGAAGAGTTTGTTGACAAATGTAGTTAGCAGACGCTGATCAAAGTCATTGTCTATCTTGCCTCCATAAATACACTGGGACAGTAGGGTTCTGATAGCATCCCAAGGGACTTTCTCTGGTGGTAGATTGGTGCGACCCATAGCCACAGAGTCAATCCAAACATCCAGCATGTCACAAGCCACACGCAAATCAGACTCTGTAAACTCATATTTCTTGGCCCAGCCTAGTGGGGT
>Bg-c30276 len=567 count=2 IPR:/ blastx_SP:/
TGAGTTTGAGCCTTTTCAAACAATTAACTTATTTAGTTCGAACCGAGGGTCCCGGGTTCGAATCCTGGTGAAGACTGGGATTTTCAACTTTGGGCGCCTCTGAGTCCACCCAGCTCTAATGGGTACCTGACATTAGTTGGGGAAAAGTACAGGCGGTTGGTCGTTGTGCTGGCTAGCTGACACCCTCGTTAACCATAGGCCACAAAAACAGACGAACTTTACATCATCTGCCCTATAGACCCCAAGGTCTGAAAGGGGAACTAGTTAGGCCAGGTTCACATCTAACTTTACATTCACTTTCACCCATCCTTTGTACTTATTGGTTGTGATGTGCATTAATTGAACATTGTGATTGTTTAGTTCAGTGATGCCCAACCTAATTTGACCAGAAAGGCATTATAATTTCCAACACTCATCTCGCGGGCCACATGAACAAAAAGTTATAAAATGAAATGAAATTGTTCTGAAATTATTTTGGTAGAAACCAATGGATTTGATACTTAATTAATAGGTTATTTGACAATTATATATTTTTTTTCGAGTAAAATAATACCTTCATTTCTCTAC
>Bg-c27170 len=272 count=3 IPR:/ blastx_SP:/
ACAATTGAATGTTTATTACTTCATTTAGTACAGCAGTACTCATGACCCATCACCAAAAGAACAGAAATCTGACCACCAAATCAACACATACACTTAACACATTTGAAAACAATACAATAGATGCTTTGATAACTTTCGTACAACTAAATAACTTATTTTGTTTTTGTATTAGTTTTAAGTAGTAGATACTTTTGTATTGACAGTAGAAATTTAGTATAATTGCTAAGAGAAGAATATCAATGGAATTTATTCATATGGGGCGAAACAAATTA
>Bg-c26612 len=287 count=3 IPR:/ blastx_SP:/
GTTTTTTTTTCTTATTGTATTTATGTCACCAAAACAACACAGGACATAGACAATTATAGACAGTTATGTAAGCCTGTAACCATTCCAAGCTTTCTCTATGATAAAAAGTTGAAACAATGATTTTTTAATAAAAAAAAAAAATAGAATGTAAATAATGAATGATGATGGTGATGATGCAGTGATGGTGGTGATGACAAAAACAAATTTAAAAGCAAGAAAATGTCATTTATCGAATAGTAGAAAACAAAAAAAATTGCCTGTCCAACCAATGTCCTTGGAGGGCTTAG
>Bg-c29817 len=664 count=2 IPR:/ blastx_SP:/
GTTAGAATTACAATCAGAGTTACATAGTTACATGTATGTGGACCATTATATTCATATGACATCTTATTATAATGCAAATGTTTTGAAACTTTATTCCAGACAGTATTTTTGAATAACTATATATATATATATATATATATATATATATATATATTTAGATAGAGATTTCAACTTATGTGCATCAATTGATGACTTTAAAAGTCACGTCAAAACAGTGAGTAAAGATTTGGTTCTTAAAAAAAAAAACGTGCTTAGATAAACTAAAGTTTTATTCAGGAATGTCCCTATTATCTCAATTTTACATGCAATGTTTTAGTATGTGATTCTTCAACGTTCTTTGTATTTAATCTAAGTCCACATTTAATTTCAACTATGTGCAACATTTACACTACATGGGTGTGTATTTATAGGTGAAAACTCTTGATTGTTTTTATGTTTTACATCTGAGAACGTTCCGACCATTTCCAGTAATTTAAATTATATTTGAGAATGTTCCAACATTTGTAGCATTCCATCCAATGTTATACTTTGAAAATGTTTGATTTTTTAGCACTCTTCCATCTAATGTCATCAGTTAGTCATCACGGTGTGCATCTTACTTCCTACATGACTGAATGCATTAAATGTTCATCTTGATTAATGCTAATAAACAAATTACACAAAC
>Bg-c33637 len=321 count=2 IPR:/ blastx_SP:/
ATACCGGTAATGCTTTTATCGCCATAGACTTATAGTCGGCTATTACAGAAGTGCTCTTGAAGCCTTAGGCTAAGCCACTGCAATTTTTTTTTCTAAGCAGAGTCCTTCCTGTGCGATAGAATTCTCCTTAAGCTGGTGATTTCCTGGTGTTGGTAGTCTGAAAGATAGTTGTCGTTCGTCTCGTGACTGAGTAATCCATCACAGTAAAGGTTTTTTGTCCAAAGTTTCCTGTCAATATTGCGAGGTTAAATTGCTTCGTCTTTGTACAGTTCTTTCCTTGTATAAATTTCCTTGTGAAGTTCTTTCTTAATGTTTTGAGGT
>Bg-c11601 len=288 count=9 IPR:/ blastx_SP:/
GTGTTTAGGTTACAACAGTATTCATTACACAGCCAGTAAACAAGCATTGCTGGGTCCAGACGCTGTTCTCTCTTAAAGTTTTCACTTGTTTGTGCATCTAAAATTACAGTTCATCGATCACGTGGAGTCTCTGGAAACATGTTGATCTACGCAGCTCTTCTAGTTTTGATGACCCCTAGCTGGGGCGCGCTGACCCCCAGGGAACTGACCGATGCACAGAAAGAAAAGTAATACTACTAACAACCTGGGGCTTCTTGAATATGTGCCCCGAGGGCAGCGGTGCTAAGG
>Bg-c17063 len=475 count=5 IPR:/ blastx_SP:/
AGGCCTCGTCAACGGAAACACAAATGGTATCGAAGGAAAGAATCTTGATTCCCCACTACCACGTGATCACGAGAAAGGTGACGAACATCAAGAAACAAATACTGGACCAGCTATTTCAAGCTTTATGATAAGGCTTTCAGCTGCAGCCACCAATGAGTCTATAGGAGACCCGCAAGTGACAGTTTCCAATGAAGCTCCTAAACCCCCCAAAGGCTATGTGGCTATTCCTATTGCTCCAGAATTAGATATGTCTGAACCAAAGGATACGGTTGAAGTTTGAGTTGTATTATCCAGGACCCAAAGACCTTCCATTCATAAGTAAATGAACAGCTAAAAACTTAATCATTTGTGACCTCTTTCAATTGATAAAATTCTGAACTCGTCAAATTATCGAATACATTCTCTCAATGACATCTCTAGTTAATAAGTACTAAAAACTTTTTTTCTAGCCAGTAATCGCACAAACTCTTACATT
>Bg-c9888 len=243 count=11 IPR:/ blastx_SP:/
ATCGAGTTGGAAAACGCTGGATCGTCCTCTACGAAACGCATGAGAGTTTGGTTCATGTCATCAATACTGATCAGCTCAAAATTCTTCATTCTCATCAGAAATTGTGGTTCTGGTCTGTTGATTATCAAAAAGTAGTCGATCAGTACGGGAACTTGAAAATAGACTTGATAGAAAGAATATTCTTGCGACATATTGGCATGAAAACACATCGAGTTTTCAAAAATTCCGTTTCTAGGATACTCG
>Bg-c23933 len=462 count=3 IPR:/ blastx_SP:/
TCAACTACATTGAATGGGCAGTGGGGGACACCGAAAGGACTAACTAACTAAGAATTTACCAAAAGACATCATAAAACAGAGCTACATAATTGCACAATCCATTGATTTCGTCTCTGGCTGAGCGGTGTCAGATTCTTGGTCAGTTGCTAACTGGACAGATTGAGCAGCTTGTTTCTCCCCGTTCTTTGGTGAAACAAACACCATCTCATCCACAAGGACAGAAGTTGAGGTTTGTTTCGTTTCTTCTGGACTGACCACTTTCTTGGAGTTTAGTTTTGGAAATATTGCTGACTTTCTGACTAGATCTAGAACGTTTGGACGGGGTCTTGGCAGATGCTACACAAAACTTACTGGCACCTACTTGCAAGCTACAAGTTTGTATTTAGTTGACCATCACAGCCATTTTTTCACTATGTCGTTGACTTGATTTGGTAACCAGGTGACAGCGGACCAAACGGATGG
>Bg-c19618 len=551 count=4 IPR:IPR013102:Pyrimidine nucleoside phosphorylase, C-terminal blastx_SP:sp|P19971|TYPH_HUMAN/Thymidine phosphorylase OS=Homo sapiens GN=TYMP PE=1 SV=2/4e-16/545-135/308-445 // sp|Q5FVR2|TYPH_RAT/Thymidine phosphorylase OS=Rattus norvegicus GN=Tymp PE=2 SV=1/5e-15/548-135/300-438 // sp|Q99N42|TYPH_MOUSE/Thymidine phosphorylase OS=Mus musculus GN=Tymp PE=2 SV=1/9e-14/548-135/293-431 // sp|P39142|PDP_BACSU/Pyrimidine-nucleoside phosphorylase OS=Bacillus subtilis GN=pdp PE=3 SV=2/3e-13/548-147/273-398 // sp|P77836|PDP_BACST/Pyrimidine-nucleoside phosphorylase OS=Bacillus stearothermophilus GN=pdp PE=1 SV=1/4e-13/542-72/275-423 // 
TATAATAACACGCACAAAATAGAATTCGTTATGGTTTAGGTAAAGACGCGCAGCACTCTTGTTTTTTTAGGCTGCACAGGAGTCTGGGAAATTTCCAAAGACTTTTTGATCTTGATCTCAATCTTATCCGATAATGGCTGATCATGATAAACTTTGATCCAGTTTTGACCTTTCAAGACTTGGTCTCCCGGCTTGTGTAACAGTTCTATACCCACAGCCCAGTTGATGGGTTCCCCTGGTAGATTACGTCCAGCTCCCAACATGTGGACAGCTACAGCAATATCCATGGCGTCGATACGTTGAATATAGCCATTTTGATGACACTGAAAACAGGTTGTACTCTTGGCTTGTGGTAGTAATGCAGTGGGGTCTAGGTCAGGGTCACAAAGATTATCTGCTAAGGTCGCGTCGACACCTTGGCACTTCAGCATGGCACAGAATTTGGACAAGGCTTCTTTATTTTCTATACTCTGACGCAATTTCTTGGACGCCTCTTCTACACTGTCAGAAAAGCCTCCAGCATGTAATAATTGACCACCAAGACTGATTCG
>Bg-c12721 len=298 count=8 IPR:/ blastx_SP:/
ATGGAAGTTCTTAAAAGAGTCAAGTTATTTTTAAACTGGTTGATATAGTCATAAGCAAAGTTGGCAACTTTTCAGCAAATGTCTGCAGCAACTCAACGGTGATGTAAAGATAATCTTATTAACAGACAACATTTTGTATAAAAAAAACACTAAAGCATATGATTTAGATTTTTTTTTTAAATGTCAACACTGTTTCATATTTCAATCCCTGATGTTGTATTTCTGGAATGAGTGAGAGATGTGTTTTGTGTTGAAATTTTGACATTGGAATACTTGTGAGAAGAATAAACTTGAAAAA
>Bg-c39981 len=245 count=2 IPR:/ blastx_SP:/
CTATATCTCAGGGATTTGATGCCAGCTCAGCCTCCCAAAAGCAGCAAGACCTCTCACACAGCAGCAGCGGTATGCAACACCAACCAACACCAGACTACAGTTCTCAAAGTTATGCCCAGAATGACAACATATATTCAACATCAGGAGTAGATGAATCGGATCAAATCTCATCTCATTCAAACATGATGGGTCATATTGGAGAAGACGGAGTTACTTATGAGACTTCCCCCCTACCATTCCCATCG
>Bg-c491 len=828 count=200 IPR:/ blastx_SP:/
TTTTGATTCTAAACACTGAATCAAGCAGTGGTAACAACGCATCCGACGGCCGGGGTGGAAAGAAATAATAGTCATACCAACACTTTGTATTGTCAGGTTTTTCAGGTTGAGGAAACAAATTAAACAATGTCTAAGCAAGTTCTTTTGGTCGCTTTGCTGGTGACTATTATAGGGTTGACTTCCTGCCAGTCTTCTTCATCTGCAGTTCGAACATCAACAGGACAGACGTCTGAGGATAGAATTCATGAGCTTATTTTGCTTGGTCAAAACAAATGCCCATACTGCCAAAGCGAAGCAAATAAGGTTCAGATTAGACCTGACAAGTCGGACATGTGCGAAGCTGTACGTGAATACTCAGGATGTGCATTAATTCGTTGCAATGGTGGTGAAAGTGTTAGAGTATATTACGTTCAGGCTCAAGATATCTGTGACAACGCTAACATTGCTCAAGTGTCCTTATTGATACTTCTCATAACTGCAGTGGCCAGATTTCTGGTTTAGACATCTTTCCTCTTGGAGCCAATTTTAAAAAATAGAAATTATTAAATTATTTGAAATAAAAGAAAAGGCAAAAAATGTTTTTCAAATAACTAACAAAGAAAGGCATAATACAATAAAGTAAAAGAGTTACATACTAATAAAGAAATATGAACAGAAAAAAAAAAAAAACAAATAATTTGTGTGATAAACCTTGATAGATCAAAAATGTTTCTACTAAACAACTGCCTGGTTCATGTTTAACTTCATTCCTTGCAGACAACTTTCTCTGAACCAAAATTATGTTCTAGAATTTTTTGTTTACAACGAGAGACCCAAGACAGGTTTGAT
>Bg-c27214 len=271 count=3 IPR:/ blastx_SP:/
AATGGCTTTAATTGTCATTTTCAGTACGTGGTGGGGAAAGTGTGCTTTAATTAAATACACATCATACCAGTGATGCTGGAAAGGTTGAAAGTTATTCCTAATGTTCATAGCTATAAAAACTAGTTTGTACATGACAAGACAATGAGGAAAAAGCAATCCCTTTAAACAAAACTTTAAAATAAAAAATGTTCACAGTGGTTTTTATCAACAGTATGCATTTTATGACAATAACATGTACAGATTCAGAGCACCCTAGGGCTCTCTTTATATA
>Bg-c3102 len=2185 count=41 IPR:IPR002452:Alpha tubulin; IPR018316:Tubulin/FtsZ, 2-layer sandwich domain blastx_SP:sp|P02553|TBA_LYTPI/Tubulin alpha chain (Fragment) OS=Lytechinus pictus PE=3 SV=1/4e-33/2183-1959/68-142 // sp|Q8WQ47|TBA_LEPDS/Tubulin alpha chain OS=Lepidoglyphus destructor PE=1 SV=2/4e-33/2183-1959/358-432 // sp|P52273|TBA_BOMMO/Tubulin alpha chain OS=Bombyx mori PE=2 SV=1/4e-33/2183-1959/358-432 // sp|P18288|TBAT_ONCMY/Tubulin alpha chain, testis-specific OS=Oncorhynchus mykiss PE=2 SV=1/4e-33/2183-1959/358-432 // sp|Q6AY56|TBA8_RAT/Tubulin alpha-8 chain OS=Rattus norvegicus GN=Tuba8 PE=2 SV=1/4e-33/2183-1959/358-432 // 
AGGATCAATTGTTTATTTTGTCATAGCTTTCATTCAAATATACACAGAAAGTGTTGCTCTCATAATTACATCACAAATTTACAAACACATTAGTCACCATGCCTATGTGTGTAAGAAAGGCGTCAGCATTAAAGTTGTCTCTAAAATATAGTTGTATTTAGACAGACCTTTCTATGGACACCTGCCAGGGCTGACACAAGGGGACATTAACAACTACAAATTCACACTATTAAGTTTGCATGATGTCAACTAAAGCACAATGATACAAAGGTGAATATGAACAGAATGAATTTGTTGGATGCATGCAGTACCATCCATTGAGTTATTAGGTGGACTGGCAATGACTGAGCAATGGACTGGGTCCAAAGGATGGTCCCAACAAAACTTGACTGTATACTAAAGTAGCCTGTGTAACAAAAGTCCAAGCAATAACCTTATTGGTCCAATAATTGAACAAGTGTAGTGTAAAAAAAAAAACAATACCAGAACAAGGTATTATATTTTAAGCCAATCTTTGAAAAAAACAAACAAAGTACTATATTGTTGAAGCCAATCTATTTCACTCCTGTTTAGCTTTGTGGATAGCCTTTTCATAGAACAGGAGCAGAACATCCCAATTAACCTGACAAATAAGATGTATACATATACCATATTATATTTTCTTTTGCAATGTATGGCTTCTGTGTGTATCATAAAATGGCAAGTAAAAATGAAGGGGGCAACAATATTGTCACATCAAACACTGTCATAAGGAAAAAAAAAAAGGTTGCATTATTGCATAGTTAGTAACTGACTTGACACTTGGACTACAAGGAGGAGTGAAAACCAAATCAATGGTTATATACAACTACAAAGATTGTATGTAGACATCATTACTTCATTCAAAACCAATTATTGCTAGAGTTATCAATTGTCAACAGTAAACCCACTCAATCACTGACATCATCCACTGACATCATCGGTGGTATAATTTTCACTTCCTAGGATTTGACTCAAATGGTTTACAGTCTATTCTGTCTCATTCACACCACTACTTTTGCAATGTTTATTTTTTCCATTTTAATTTCATAGCTACAAACCCGTAAGAACAATTTATAAATGAAATACAAGAACTGTCTATTATTATGTATACAAACAAATGACAAAACAACTAAGCCATAGTGCACAAGGAAGTTCACATCAAGGGATGATCCTAGCCAGCCCATATTGTCAAGAAAGGCCAGCAACATATCTCCTTAGCTCATTTAACCATCTGACAATGTTCTAGTGTTTGGTATATATCAGAGGCAGTCCATGTTTAGTCCCAGAGTATCCCAAACATAAATTATTTCCCTTGACAACCTGCCATAATGAAAGGATTATTTCTTTGTAGAAGCCTTACAATATTTGAAAACTGTCTATTTTAAAAGTCCACCACTTTAACTCATACAATGCAATGCGTTATATCATATTTGTACAATCTTAGGCCACATTAGAAATATAACTATAGTACACAAGCAGTAATGTAATAATTTACTTCACAAAATATAAAAGAATGAGAAAAACAAAAAGGTCGAAAATAAGGACAAAATGGCAACAAGAACAAGCTCATTATGAACAGTGTTTGTATACTGACCAAGTCATAGATGTAGTTCCAGGCTGTTCAACAGGAGACAAGACTACTAGGAAACTATGTACAACTATAGTAACATTAAGTCTCCACATCTTGCCCTAGAAATAGATACAATAGACTTATATATATATACATATATAATACATAAATCTTAGGCAGCCACAAAAATAAAAGTTCTCTATTATAAGTATGCATGATAAAAATACTCCAACAACATACATAGGACTGACAGATGTATGCTGTTCAGATGTTGGAATGTTCATGTGTTAAGTATTCAATTTAGTTTTTAGTACTCCTCTCCTTCCTCCTCCCCTTCACCCTGCACAGAGTCCACACCAACCTCCTCGTAGTCTTTCTCCAGGGCAGCCAAGTCCTCACGAGCCTCCGAGAACTCACCTTCCTCCATACCTTCACCCACATACCAGTGGACGAAAGTACGTTTTGCATACATCAGGTCAAACTTATGGTCAAGTCTTGCCCAGGCCTCCGCTATAAGGGCGGTGTTGCTCAACATGCAGACTGCGCGCTCCCCTAAGGGCAGGTCACCTCCTGGTACAACAGTTGGGGGTTGGT
>Bg-c260 len=1294 count=291 IPR:IPR013781:Glycoside hydrolase, subgroup, catalytic core blastx_SP:sp|Q8WPJ2|MANA_MYTED/Mannan endo-1,4-beta-mannosidase OS=Mytilus edulis PE=1 SV=1/2e-106/1177-137/17-363 // 
CAGCAGATAATTCTTCTTTATTCATAGCATAAGTGAGAAACATTAAAAAAAATATTTAAACAATATCAAGAAATATAACTCAGAAATAAATGAAGTAAAAAAATAGAATGTCTTTAAATCTTTACACATTGATGGGTATTACACCATTAGAAGTTAAGCCCTTAATGTGACTGATTCCACCTCGTTGGTTGGCTCCGTGAGCCTGAAGGTCCCAGCTCCAGGCTCCAGCGTAGCCGTGATTGTAGACGTATTCAAATAGCTGGTTAATGTTCATGCCTCCACCATCTTGTTCCCAGAATTCACCGACTACAATTGGCTTTCCGGTTCCGAAATCTCCAGCAGAATGCTTAAAAGGGGAGACGTTGTCAAAGTTCCCTTGGTAAGAGTAAGAATGGACTTGGTAGAAATCTAGTTTACCGTTTGGTTTACCTCCCTTGAGCAGACAGGCATCTGAGTAATGGTCGACAAAGCCGAAACGGTCAGTGTTGGATTTAGGGTTCCAGGCGCCAACAGTGACTAGAAATCCGGGGTCGACGTGCTTAATAGCTGCAGCCTGCCAGTTGACGAATCTGATGATGTCATGGTAACCATATTTCTTGGCAGCCCAGCCAGCTCCACTGTTTTTCAGAGCAGTCGCGTCATAACAGCGATCAGAGTTACCGATATCAGGGTTGATCATGCCTTCTGGCTCATTCATGATGTCCCAGGCGCCCAGAGCAGGGTGACCTTTGACAGCAGTAGCCAGGGGCGTCAACACTTTATCAATGTAGCTCTGCAGCTTTCTTTCATCGACGATTAGACCGTCGAGTCTGTGGCTGTTATCTTGGTTCACAGCGGCGTTCCATAAGGTGGGGAACACAAGGATGTCATGGCTCTGTGCTAAGTTCAACATGTCTTTAAAGTCATTAATAAGTGTTCCTTGATGGTCAGGCGCTGTGACGTAACCGTTGCCGTCAAAGGCTGGGGTCGTTTCTCCTTGAATGTGAATCCATAGTCTCAGAGAGTTACCTCCCGCTGCCTTCAGTGCTGCTAATTGACTGTTGACCGCACTTTTGCGACTTGCCCACTGATGGTCACCAAAGTCATGGGCATACTGGATCCAGGGCAAGTTGCCACCGGAAAGAAACACCCTTTGACCATTGAAGGTGAAATGGTTGCCTGATACTGCCAGTCGGGCGCTAAGTGCTAATTTGAGCACACCAAACACAAGAAGCAAGCACGTTATTGTTTTATCCATATTGTTTGGTTTCACTGGCTATGATCTTAATGAAGCGATTAGCCTCCCCCGCGTCGG
>Bg-c22497 len=243 count=4 IPR:/ blastx_SP:/
TTTTATCGTAGATCGCGATTCTTGTTTAATATAGATTCTAGATCTAGACTCTAAATCTAGACTCTATACACTAACTATAAGTGACTATTCACTATATAGCCTCTAGACGTCTAGACTAAATATATCTATATTATAGTATACAGTGATTAGTGATACACTCATACTAGAATCTAGACTTAAGACTAGTCTAGATATCTACTACACTACTAATCTAGTCTGACTAGTCAAGCTGTTGCTTTTACG
>Bg-c19792 len=498 count=4 IPR:/ blastx_SP:/
TTTTAGATGATATATTTTTTGATAACCTAACCACCATTCTATTTTAAATAAACTTTTTTTCCCTATTCAAACTACAAGTATTTTAGAATGCTTCTTGTATACTATTGTCCACAAATAAGAACAAAAATTTCAACGTCAATGTCTAATAAAAGATATCACATTTAATTTAAAGTCAACATTATAGAAACATTTTAAAAGTGAAAACATAAAGCAAAGAATACAAAATTGATGAAGAGTTATGTTTCACACAGCCAGCTAGCACAAAGAGTGCTTACAAATAAGTCACAAACTCACCCTACATTGCATTCACAACAGTGGCTATAAAGTAACCATTGTATCACAAACAGACTTCAAGGGGATGTTTTTAAAAGTAACTTAAGAAGCTATTGCATACATTTCAAGTCGTTTTAATTGAAAGAACTTAAGTAGCTATTGCATACATTTCTAAGTCGTTTTTAAAATTACAATTAGTTAAAAATTTAAAAATTTAGGAATAAA
>Bg-c41227 len=230 count=2 IPR:/ blastx_SP:/
TCCAGATGTCTTTGCCCTGGTGGTTATGGTGGAACTGATTGCACAGAAAAAACCTTTTGATGTATGCGGTCGAGAGCTAGCGGCAAATAGAACTTGGCAAGAGTTGGTGATTAGGATTCAAAATAATAACGCATATGATTATCTAGATGGATACAAAAAGTAGTCACTACTGGATCAAGTCTCCGGAAGGCACCAAAATACAAGATTATACTCGAGAAACTTACTATTTG
>Bg-c16081 len=265 count=6 IPR:/ blastx_SP:/
TTTTTATTCTTTGCTCTAATATCTAAACCACAATCTTATAAGGACTTCCCATTCATAGCTGATACTTCCAAATGTGTACCTCTTACACTTCAACGTCTGTCCACCGAAACATAGAGATGAGAATCCTCTCTATATATATTGTATATCAAAAGTAAACTTTATAGTTAGCTCCAATGTTTATCTTTTATATTTTAGTAGTAAAATCCAATGGTACACATCTTTGTTTAAACTAATGGTACAGTAGAATGTTATTCGAATGTATGCT
>Bg-c16120 len=261 count=6 IPR:/ blastx_SP:/
CTTTCTGTATCTCCATTTCGCTAAAAAGAAACAGATATACTCCAATGACTTCTGAGAATCTTCGAACTATGCCATCGTCTTACCCTATGTGGCTCTTTTGCTTTAGTTTTTTTTGTTTTGTTGTCGTTTCTTTATCATCGAGTTGCCCGAAAGCTTTGTTCTGCATCATCTTAGCGCTTGAAACCTTTGTTCTCTAGCATCCTAGTGCTCGACACATCTTTGTTTCTGCACCAAGCTAGTAAAGTGCCTCAATAGTAACGG
>Bg-c11539 len=305 count=9 IPR:/ blastx_SP:/
ACCGGCCAGAGTCTGTTTAGATTTTCCTTACATCTTCTGCGCTCTTCCGCTTTCTAGCAATATAAGAAACAAAATTCAGATAATGATTAAATCTCTCTTAGCCGGCTAATGATTTTACTTTTAATTCAACACATATGTTATAATCGTCATTATTGCCAGTATTATCATATTTATTGGTACCATTATATTTGTTAGTAACTTTATATGTTAGTAACTTTATATTTGATAGAACCATCATATTTGTTTGTAACTTTCTATATGTTAGTAACTTTATATTTGATAAAAACATCATATTTGTTAGTAAA
>Bg-c27759 len=259 count=3 IPR:/ blastx_SP:/
TTGAGGGTCTTACGTTTGTTTTAGGGCTACAATACATTCACTAAGGTCTAAGTTGGTACCCAAGGAACATTCCTGCCTAGTTTTATCAAGATTGGTCAAGCGGTTTTGATGTCTATAAGTAACATACATACATACATACATACGTCACACATTCTACTTTATAATATAGATATAATTCCAGTAAAATATAGACCTACATGTTAATAAAAATACAAATTATTACAAAGTTTTTCTAGTGGACATAGTTAATGGATTACAA
>Bg-c33290 len=331 count=2 IPR:/ blastx_SP:/
ATTTTTCGAGAAGTCCCCTCTCAGATCTGGAACTATCTTAAGTTTCCATACTTAGATACATAAGGCAGGTGCTGAGACCACTGTGCCCAAGAGATTTAAGATAGAAGAAAAGTTAGTTTCGCAAAAAGCACCCCCCCCCCCAAAAAAAAAACGTAGCTTCAAACTTATAAAAAGTTTCTCCTCCGTCGTGTGCTTATAAGGTCTCCTGCGCGCACTCTTTGGGTACTTCAGAAACAGTTTTAGTACACCTTACAGAGTTAACTACAGCACTTCCTGTACAGCTCTATAGAATATACGTGTTCCTCTTGTGAATTCTCAAAGGTCTAATAAC
>Bg-c30363 len=548 count=2 IPR:/ blastx_SP:/
GAAAAAAAAAAACCCTTTTTGGGTTGAAACATAAACATACATTGTACTACGAATCCTTATACATCCTATTTATAGGCAATGCCTTTTGTAGAATATCATGGCAAAGTATATAAATGACTGAATGAAATGACATAATTGTTTTATTTTGTAGACGAAAACAATTGTATGCAAACTTTGTACCTTGACTTCATAAGAACATTTCAATGAACAAAAAATACAATTGTAGACTTTTACAAGATTACAAAAATTATCAGATAAAAACTTTTCAAATGAAAAATAAAAAGACTAGCAGCAGACTTGAATACAAACACTTTTGGAATGTCCAGAACAAACATTTGGTCAAATAACAGTCAACAGTATTTTTTGTTTCCCAAAGCTATGACCTTGATGGTCTCCCTTGAATAGCTTCCCTCTTATTGAACTTAGACATCATCAGTCAAAACAAAATTACGACACACTTTCAGTTGAATCACTTGCATAGATAATTGTATCACTCACTGAAATATTTCACTTTGCCTGCCAGGTAAAACTTTCACAAGTTTGATTTT
>Bg-c4618 len=1196 count=26 IPR:/ blastx_SP:/
TGGGATCATTCGATTCGCTTCTGATGGTTCTTTAATTCGTCTTTATGACCCTTTCAATAAGAAAGAGTGGGCGTCTTCGACCTCGAGAATTGGCCAGATCCTTTACGACACGTACGTGGAACAAGATTTTATTGACATGGCCAAATTGTACAACTATGTTTCTGGTGTTGGCTATGACAAGCCAAACATGACAGACAACGCTCATCCAGAAAAGAAAAGATGGGAAGTGACAATGCTAAACCTGTACAAATATATCGGATTAAATTTTACTACGTGTGTCTTTTGGGTCAAGGCCGTTACTAAGGATGATCAGACGAGAACTAAATATGGCGCCCCGAAAGTATTTTATATACAGTATAGAATGGAGTCTTCCATTATGCCTACCGTAAATGTCACATTCTTGATGCTGGGTAAGACTACTACCAGACTGCCTGAGTCCATCTCCTTTGGCTTCCAGCCTGTCAATCAAGGTTACCAGTGGACACTGGACAAGATGTCACAGTACATTGACCCCTGTGATGTAGTGTTAAACGGGAGTCAGTATGTCCACGGTGTCAAGAATGAAGTGAACTTGTTAAATGAAGACAACAAGGGCATACAGTTCTTCACCAGAGACGTGCCGATAGTCAATCTAGGGACAGACAATCGCATTGACTCCCCTTTCCCTGTCCCACTTGAGCCATTTGAGTGCTCTACCCTGAGAAATTTCTCCTTCAATATCTACAACAACATCTGGAACACCAATTACCCCATGTGGTATCCATTTAACCAACAGGATGAGAACTTTAAAGCAGAGTTTACCATTTCGTTCATTAGTTAGAATTGGGAGCATTAATTGGTACAAGTTTAAGTCTTAGTGGTGGAGGGAGCATTTATTAATACACATTCTAGACTTTAGCCACTTAAGGAGCATTTATTAATTCAAGTTTTAGACTTAGGGAGCATTTATTAATACAAATACACATTGTAGAATAAAGGAAAGTTTAATACTATCATTGCCCAACTTTATGCTTCACTCTGTATAATATAATTAAACTTTTAAAACTTGTATGCGATAAATATGCAGTAGAACCAATACAAGTGTTGGCAAAATAAACTAAAAGCCTAATATTTTAATGAACTCAATGAATTATTATAATTATATCATTGATTAGATCTAGATCTATGTAAGTCTATTTGGTCAGCATTATTACTTA
>Bg-c29078 len=163 count=3 IPR:/ blastx_SP:/
ATGAGAAATGTATGTCTAAGACATGGAGCGGATTTTCAGGAGAACAACTTAATGCATGTAGATACGCATTTGAACAAAAAAGTAAACTTGAATCCAGATCAAAGTTGCATCTTGGCGGACTGCATATGCGAATTGTTTCGAACAGCAGTTCCGAAGTGAAGAA
>Bg-c7014 len=643 count=16 IPR:/ blastx_SP:/
ATTAAAAGTTTATTTTTGTTAAGTTACTTCTAGTGTTTATTGAGTCGTTGTTATTCATTTTTACTAAGGGAGAGTATTCCAAATATTCATCAAAGATGTGTTGTTGCTTCCCTTCTGGAACATGTCCAAAGATCTATTGACCATCGTCGCCCTAAAACTAACGGTTTGCTTCCTGCAGCTATCATCAAGGCTGATCCGCTTAGACTAAAAAAGAAGCCCCACGATGCACGATAGTAGGTTGTCTCTTTATCATATGGAACCAGGAACTCGACCCCCATTTTGAAGAAATACACCAGAGCACCCAGTAAACTCGTGGTTCCACTTAAGACAAATGCTATGTAACTCAAATAGTAGTTTTTCTTCTTTTTGGACACAGTCAAACAGACGGCTGTCAACATCCCATTACAAAACATGATTCCACTGGTCATAAACAAAGCCCCACCTACAGCCAACCACTCGGAGGATTCAATGTCTTCTACAAACTTTGAGCAGTAAGGGTCACTGTAGGTCTGCAGCTCTGGTATGGACCCCACCACATACATATAGCAGGCCTGCCACAAACCAGCGTGGGCCACTTCTTGCATCTCTACATTCCGATTGAGGCAGTCCATCCATTTGTAGCAGAGAGGCCTCCGGAGTTCAA
>Bg-c27215 len=271 count=3 IPR:/ blastx_SP:/
TGATTACATTTGTCAATTAAATAATCAAAATTTCAATATATACATACAAAGTAAAGAAGTCAAGTCCCCTTTTCAGACCTTGCGTTCTATAGGGAAGATAATGTAAAGGTTTCTGTGACCATGGTTCATGAGGGTGTCATATGGCTAGCACAATGACCAACCGGTTTTACTTTTCAAACAGCTAATGTCATGTACCCATTAGAGCTGGGTGGACTCAGCTGGGCCCTTAAGATCTCAAAATTAAAAATCCCAGTCGTCACCAGAATTTGAA
>Bg-c26833 len=281 count=3 IPR:/ blastx_SP:/
GAAATTTCAAGTAATTGATAATTGATTCTAAATATTTAATAACATACTGATGTATATTTTGTACAAATAAGCCAATATGCTTAATTAACTTCCTTATATTTTTAAAAATATTTTTTATCTCTCAAAAACTACAAATTCGTCTTTTACTTAGTGGTCTTAACAGATTTCAGATTATTTAGTCATATTAGCTGCAGTCTTATCAAGTTATTTTAAACATTGATATCAGTCTAAATTATATTTTATCAATTGGTTTTATTATTTTAGATGTTTTTACACAATAA
>Bg-c13897 len=343 count=7 IPR:/ blastx_SP:/
TTATTAGTGCTATATCATGCTTTTAGCTTTCTCAATATGCTATGATCCTATCACTTATCTGTACCAGTTGGAAATACTGGAAAGGGGGATGGGGGAGAGTTCTGGGTGAATTTTACTGCAATCACTTTTTAAAAACATTTAAAGGAGTGGGAACGACCAGAATTCGAAATCATGGCTCACACCTACTCAAGCAGACATACTAACCACTGCTAGTGAGGTGAAAATGAAAACAGAAGATTATACTGTATAGTTATCTATTGTTTGTTTCAATCTTACTTCATAAACTAATTGATTTAGTTTTTTTAATTGAATATTGTATTGTCAGGTAAAATAAAAATAAAAT
>Bg-c11355 len=378 count=9 IPR:/ blastx_SP:/
GACAGATGTCTTCCATGTCTCACATGCATATGTAGCAGTTGGAATGATGATTGTGTTGAGAAAGTGTATTTTTGTCTCGAGTCCAATGGCTTGGCGAGTCCAATGGCACCCTAAAGATCCTAAAATCAAAATACTTGTCTTCACCAGGATTTGAACCCATAGCCTCTGGTTTGGAAGCCAAGTGCTTTACCACTCGGCCATATGCCTCCATTAGCTTTGAAAGACTTTTCTGAATTGTTTAAAGGCTAGATTTTGTGGCAATTATATTTAGGAGAGAAAATGATGTACCGGTATGCATAAAGTTGTACCATTTTTTTAAATTTATCAGTATTTAACTACTGACTTCTTAATCTCTGGATAGACAATATAAGTTTGTAA
>Bg-c37557 len=265 count=2 IPR:/ blastx_SP:/
GTCACGGTGGTCGTTATAGTGGAGGATGGAGCGGATACGGTGGTTGTCGTCGCCGACGCAGTTTCACGAAGAGCCCAATTAGGGATTCGCGGAGATCTAGACGTTCTCGTACACGCGAAAGATCTCATTCGCGATCCCGGTCTCGAAGCAGCTCACATTCGTCTCACTCTACACGCTCGCGCTCTCGTTAAGACAGTAAATCACCCTCGAGGACTCTAGTCGTTCTTTGTTATGACCGTAGTTAATTTTTAATAACTTCTGTTAA
>Bg-c42988 len=120 count=2 IPR:/ blastx_SP:/
CACATCAAGAACATTAAGAATTCACCAAAAGTATGATGATAGCGTGCCCTCTGATCTAGTAGGAATCTGAATATATTCAATATTTAAACTTTGTATGAAGTTATTCATCTATTACAGTTT
>Bg-c39644 len=248 count=2 IPR:/ blastx_SP:/
AAAACACAGATAGCCATCAAACTTTGATCTATAAGTTAGTACGAACAAATGTGTGAACAGCCAACAGTAGACAAGTTTGAAATGATATCATTTGATTGCTGAGGAAAGTCTTCATGGCGGGAGCCAAGAAACTGAAACTTCCTCAATACTTTGATTTGGACTCGTCTTCAGATTTCAATGCGCCGATTGAGGCACCTGTTACAGGTCGTTTACCTGGCTGGTTGTGCGGGTCGCTCTATCGCAACGGA
>Bg-c34550 len=300 count=2 IPR:/ blastx_SP:/
TTGTAGATGTAACAAAAGAAGACTGGATTCAATGAAAATAAAGAAGGAAAAAGGAAAAATATCGGAAACAATGTCTAAGAACAGGGTGGATGAATGTGACTTATCAAAAACAAAAGTTTTAATTATAACCGATTCAGAGGCGGAACAACAAAAATCTCCAAAAACAGAAGAACAAGGGGATCGCATTTACTTCCAACTTGAAAAGGACCCAGACAATTGTCAGTTTATTTTAGAAGACAACCTAAAGAAAAGGATGTTGCTTCAAGAGTGTTCTGGTCTGAATTTCAATGTTTGGTACAG
>Bg-c3017 len=660 count=43 IPR:/ blastx_SP:/
GTCCCAGGTTGTGTGCAAGTTTATTTATAGTTCACTTTCAGTGCAATAAAAAGACGAATCACTAATTGTATCGCTTAAGAAAGAAAAGGAAACTATTTCATTTCTTTTGTTTTGGACTAAAGATGTTTTCTGTTCGATTAGCATTCCTTCTGACCATCCTGTCAGTGAGTGTTCCAGTTATTTCTACGCAAGAAAATCCCCAGAACCCGATTGTTAGCCTCTACGCCAGCATAATGAACACAGTAATAGACGCGTATAAAAGTCATTTTGGCTCGCCGCCACCAAAGTGCTTTATCACCAATAGTGCTATAGATTGCTTCCTAAATGTGAGAAAGAACACTCTTAGCAAGGCAGAGTCGCTGACGAAGTCTCCTCCGGAGTTTCAACAGGTGTTTTGTCAGGCGAAGCGGGACTTAAAGAACTGTTCCAGGCCGTATATCAAAGAGTGTTCACGTTTGAACAGAATTCTGCGTTACATCATGCACCAGGCTTTCCAAGTCACGTGCTCATCACATGGTTATCATAGGTACATGTGCTTAGAGGTGGTTGCAGTGCCCGCAAGAGAGTGTGCAAAAACTGTGGCTACACATACACTGAGCTCCAACTTCGGCGTAGCGCTTGATCAATACGTGGATTGCTTGCTGCTAGCTCGACCAACAC
>Bg-c29725 len=683 count=2 IPR:/ blastx_SP:/
CATCTGTGCAGCAAAATGGCCGACAAGTTCAACCTGTTTCTGTCCGGTTTAAGAGTGCCTGTATTGACCTCGTTCAAAGTCTCTGATCTTCAAGTGGACAGCACCAGTAGGATGGTGATAAAAAAAATTAATAAGACATTGACGGAAATGTTCGCCATGGACTGTCATCTGAAGGTCTTTGAGATGCCTGCCGCCCACTGGCCGAATAGATTTGGTCGCGAGCTCCTGAACACCGTGCTTCAAAAATCAAGAAACACTTTAGAAATACTAAATATTAAGGACTTCTTCATTGGTAAAAACGGGTCAGTTGTTAAAGGCTCGAATTGGTTTTTAACAGTGCTGACGTCATTGGCAAACTTAACAAGACTTTCAATCACTATATACTATCTCACCGATGAATTGGTTTTGTCTCTTGCTCGAGCCAGAAGAGGTCAACTGACGCACCTCTCTCTGTTGACGAACTTTATATTTACAAACACTGTACAACGAGATAGCTGGGACTACCTGTGGAATGCATGCCCCAATGTTGCAATCGAACTAACGATTGACGGAGATATTGACGAACCCCAAGAGGATCTTCCATATTTCTTGGAGCCGCTAGAAATTCCCATCAAGAATATAAAGATGATAGTCAATAGGAATTTTCATTCTTCTCCATCGTCTGTATTTGGGGATGAAAGTCG
>Bg-c19522 len=588 count=4 IPR:/ blastx_SP:/
TTCATTTGAATGCAATGATATTATTAGAACCAAAGTTGCTTTTCCTTTTCACATATCCAGCAGTATTTTGTCTTTTTGTTCACCTACTAAAAGAGCTTCTTCTTATAAACAGATAACTTTTGTATATTCCTGCAGGGAATGTTGGTAGCTAGGAATGGAACACAGATAATCTCATTGACATTTTGAAAGACATAAGATATGACTAGCCCCTGTAGTATCCTATACTAAAGTCTAAAAAATAAACATTGTATTTTTTTTTTAGAGAGAATATAAAAAAAAAAAAGTAAACAATCCCTTACAGACCTTGCAATCTATGGAAAAGAGGATGTCAGGTACCCATTAGAGTTGTTTGGACTTGTGGGTACCCTGGGAAAAAAATAATAATTCAAAATCCCAGTCAATATTCAAACCTGATAAACCTTGGTTCAGATACCAAGCAATTCACTCCCCTTTTATAGAAAAGCAATTATATTTACTACACTGTCATAACAAACATTTGATGATTTAATTTGTATTTACAATGTATAGAGTATGTCATCTCGTTTTCATTTTTTTTCTCAAATCCAACATCATTTCATCATTCACTAA
>Bg-c5275 len=314 count=23 IPR:/ blastx_SP:/
AAACACACAAACACCACAGTTACATTTGCAACAAAAAAACAAAGGGGAAAAAACAAACACATTATGTAGATAGTGGAATGTAGATAACATATAAATAAGCAGTCAACCCAAAGGAAAAGAACAGATAGAGAAAGATACTCGCAAAAACCACAGTTACATGAGCAACCAAAACAAGAAAAAAAAACAATACACATTAGGTAGATAGTCACACATAAATAAGCAGCAAACCCAAAGGTACAGAACAGAGAAACAAACACACAAACATCTCATTTACATAGAACAGATAAAAAACACAGACAGAGTACAAAAGAACA
>Bg-c43025 len=117 count=2 IPR:/ blastx_SP:/
CTTTTACTGACAACCAGGGAAATCTTGAACATTTTACTGACATCCAGGGACATCTTGAACATTTTACTGACATCCAGGGACATCTTGAACATTTTAACTTCCTCCTGTGTATACCTT
>Bg-c40574 len=239 count=2 IPR:/ blastx_SP:/
AATGCCAGTTGTATTAATTCAAATTCTTAAATGTATTTGGCTAGAATTTTTTTGTTCAATGTATACATTTATTTTTGTGTGCAATACTCTGACAGGGTATAAGTTTAAAAAACTAAGACTGTGATAAGAATGAACTTTTTAAAAGTATTCCAAGTTTTTACATTTGATCATTAATTTCATATGAAATGTCATCTTATATTTATTCTTTTTAGTTTTTATGTTTCTTGCTCGTCAAGATA
>Bg-c19488 len=601 count=4 IPR:/ blastx_SP:/
ATCTACAAAACGGCTCTATGTATTTTCCTAAGTAAATTTAACACTTGAAGTATATCACTGAGGAAAAGAATTACTAGCCACACGAAGGGAAAACTCTAGATTGACCGTTATTAACTTTTTACAAAATAAAAAATAAATGTAAATTTGGCTAGGTCTAGATCTAACATCGGTTTTGAAAGGCCTCTCGAGTTTGGGCATTTCCGATTGTAAGGCATTATTGATTCTAGAGTTTAATAAATTAGTGTTCAGGAAAATTTTGCTCATAGTCTTATAAGTCTAGATTTAAAGGCCTATCATTGAAATTATTCAAGAAACTTAAAAAAACATGGCTTATTAAATGTAAAGTTGCTTCAAGTTTTTAAAAAGAATATCTAAGCTTTACTAATATTTTGTACTAGAAGTAGACCTAGACTTGTTCTTAAGTACTAAATCAGAAGTCTAGGTCTAGAGCTAGATATCAATATAATGATAATATCAATAAATAGTCGCTAAACTATGCATTTCCATGGCCAATTTGTTAACGACATACTTTCAAGAAGTTGATGGTCTCAAAATCATGGTTTGAACTAAATATTTTTAAATTAATTACAAAAATATTCTA
>Bg-c23406 len=595 count=3 IPR:/ blastx_SP:/
CTTAAACTGAAAGGACTGCAAATTAAGGCTCATAAGGCTACACAATGTGGTCACTTCTTCTCTGTCTGCCACTGCTTCCCTCCATTGCACTTTCTCTGGACCTTCAACCCCACAACGAGACGGCCGCTGCAGCGTTTACAGCCACAGACCTGAACCATGATGGTCATATCGATAAACCAGAAATAGAGAACCTTTTCAAGTTGTTTGATACGAACCACGACGGCCGGGTCAGCGGGGATGAGTTCATGGCGACTGTGCGTAGCCACCAGCACGACGCGCGGATCAACTTCATCTTCTGGAGCCTGTTTAACATTTATGACATCACCAACAACAACGTCGTGGACCACATTGACATTGACCGCCTGTTTGCGCTGATTGACAGGAACGGGGACAATGTCGTCAGCCGACAAGAGTATACCCAGTACTTCGCTCATCAATTTGAACTAATGGATAAAGAAATTTAAGTGTGGAAAAAACATTGTGCACAATCAGTCCAGCACTTTCTTCACAATTCCAGATCATAGTTACAGCAGCTTTGTATTTCTCTATATGTATGATGTCAAATAAGAAGCCAATACAATAAAATTTAACATTC
>Bg-c23245 len=663 count=3 IPR:IPR016186:C-type lectin-like; IPR001304:C-type lectin blastx_SP:sp|P70194|CLC4F_MOUSE/C-type lectin domain family 4 member F OS=Mus musculus GN=Clec4f PE=1 SV=1/2e-07/394-101/436-538 // sp|Q67EQ1|CLC4E_RAT/C-type lectin domain family 4 member E OS=Rattus norvegicus GN=Clec4e PE=2 SV=1/3e-07/433-104/92-207 // sp|Q9ULY5|CLC4E_HUMAN/C-type lectin domain family 4 member E OS=Homo sapiens GN=CLEC4E PE=2 SV=1/4e-07/433-104/91-206 // sp|P26305|LPSBP_PERAM/Hemolymph lipopolysaccharide-binding protein OS=Periplaneta americana PE=1 SV=1/1e-06/412-95/138-256 // sp|P10716|CLC4F_RAT/C-type lectin domain family 4 member F OS=Rattus norvegicus GN=Clec4f PE=1 SV=1/1e-06/394-101/436-538 // 
GGTAATTAACTATTTATTTTGTTCGCACGCCAGAAACACCAAAAAGCTTGCTATTTATAGCTTTTCGTTATACATTGAAATCTCCCGTTTTTTACAATTCTTTCTCACACACGAAGAGGTATGACATAGTGCACGGCACATCGTTGAGTAGGCTCCCTTGCAGGATGACGCAGTCTTCAACGTGTAGACTATTACTTGGCTCATTTAAATTAAAGAAACCACTGGAGCGTGGATTTTCAAGCTGTTCGCCGTTATGCCATATGAACTTGCCTTCAGTCTGTAGGTCATCTAAACCGATAAAGGTCACGTTGTTTTGTCTCTGCAAAATTTTCATTTTATCCCATGTATTGAAAACGCCCAACCTGGTTCCACGAATCAGACAATACTTTTTAGCTCCAGCGAAAGTATTAGGCCAGGAAGACCATCTAAGACACATTACAACATCTCCTTCCTCCTCTATCTTAAAGTCTGAAAAGAATCCTGTTCCACACTGATCATGTGCTGGGTCAAATGTGTCGATGGGAGGTGCTTGCCCACCTATAACTGTGGCGTAGAGAATCAAAACCAATATTGACGAAAACATTTTTGATAAGCCAACACAAAGACCAAGAGACTTTGTTGATGCCGTTCAGATATCTTATCAAGCTGTTTCTTCCCTAAGAA
>Bg-c7120 len=457 count=16 IPR:/ blastx_SP:/
ATATGTGCATATCTGTTAAATTGTTTCGGATTGTGTTTCGGATGAGGTCTTGTGACGTCATTCGCCTGAAATGGTCGTAATTACGGACCTCTATCGAACATGACATCATCAAAGCGTTCACTAGGAGAATCGTGGTAGATTCGGCACTTCCTTTCGGCTGCCTTCATGCTCGTTCCATGCAGTTTTCCGTCTTTTCTTTCTCATTTCATTCTTTCCTCTTATTCATCCGATCATTCTCTTGTCGCGTGTCAATCAGGGCTTTGTAAGTCGTCCTACTTCAGACGGCATGTATACGAATTATTGATTTTATTGATTTCTGTAGGAATTCTATGGTGCAATAACTGTTATATGTGACGAAGTGTGTTGCGTTTCTCGACTGGTATACCACCAGCTCTTGACGCTGTCTAGTCGTTGGTGATGGCGTAACAGTCGTTTTATGAGAGTAAAATCTATTGAT
>Bg-c42853 len=131 count=2 IPR:/ blastx_SP:/
GTAAGGCAGACATCGTAAGGCAGACATTCTAACATTTACAATTAAACTTCAGTATCTCAACAACAAAATGGAAATAACAAAAAGAACAACCCGCAGACTTTCAACAGAGAAAATAATTCCTCCCCCACGTT
>Bg-c34776 len=296 count=2 IPR:IPR013781:Glycoside hydrolase, subgroup, catalytic core; IPR001223:Glycoside hydrolase, family 18, catalytic domain blastx_SP:sp|Q15782|CH3L2_HUMAN/Chitinase-3-like protein 2 OS=Homo sapiens GN=CHI3L2 PE=1 SV=1/2e-12/160-291/23-66 // sp|Q95M17|CHIA_BOVIN/Acidic mammalian chitinase OS=Bos taurus GN=CHIA PE=1 SV=1/1e-10/106-294/1-62 // sp|Q9BZP6|CHIA_HUMAN/Acidic mammalian chitinase OS=Homo sapiens GN=CHIA PE=1 SV=1/2e-10/166-285/20-59 // sp|Q91XA9|CHIA_MOUSE/Acidic mammalian chitinase OS=Mus musculus GN=Chia PE=1 SV=2/4e-10/106-285/1-59 // sp|P29030|CHIT_BRUMA/Endochitinase OS=Brugia malayi PE=1 SV=1/6e-10/124-291/7-62 // 
AAACCGTGTCTAAAACAAACTAATTGTATACAAGACAAAATTAATTCATATAGGATATTTCACATATCGATTTGCTATCAAATAAATTTTATATCATATTTAATTATGTTTAAATTTTTGGCAGTGATTTTCTTTGCCTGTATGGCAAATGGCGTTTTTGGTGCCTCGTGTCGACGTCGTGTCTGCTACCACACCAACTGGTCCCAATACAGACCAGAGCCTGGAAAGTTCATGCCAGAAAATATTGACCCTCACCTGTGTACTCACATCATCTACTCCTTCGCCAAACTTAGCAG
>Bg-c23328 len=629 count=3 IPR:/ blastx_SP:/
GTTTCGTATGATTTATTGATTTAGTTTTTGCAAGTTATGTCGTGTCAAGGTTATTGCAACATCAAATATGAAAAGAACAAATTTTGTACAAGTTATCTCCAAATAAAGAGAGAAATGAAGGCAATGACATAGTCATGTGTGGGCCCATATCAGGCCATGCAGGGGAAATCACCCAGAGACAAGACAGGTTTAGTTAGTACTAGTGTTATATTGACAAGAGTTACTTGCCCTGCCGTCTAACTACATTTTAAAAAAGTAAAGAATGGCTGCCAAGGTGGTCGTACATGTGTGTGTGTGTGTGTGTGTGTACAAATGACTGGAAAAAAAATGAAGTAAAAACAAAACAAGTTAAAAACATGTTTACATAATCGAATTAGATCATTCTAAAAGAAAGGGGGGGGGGGGCAGTACACGACCAATTGAATGTGAGATTGGGAGGTACTGTACAATCTGAAGCAACTCTTCTGTTAGCATTAGCTTTTTTGGGGGGCGAATAGAGTTTGCCTTTCAATTGAGAGTCTGAGACAATCTAAACAATTGATGATGGATTGTCAATGTGGTCTTTGAAATAATGTCCAAAACAAGAAGAAATGTTTAGCAAGCTTTAGATTAAACAATTTTGTCACATG
>Bg-c3741 len=1876 count=33 IPR:IPR002861:Reeler domain blastx_SP:sp|Q8MSU3|FRRS1_DROME/Putative ferric-chelate reductase 1 homolog OS=Drosophila melanogaster GN=CG8399 PE=2 SV=1/4e-09/147-569/30-177 // sp|Q86RS3|DFP_MANSE/Putative defense protein Hdd11-like OS=Manduca sexta PE=2 SV=1/7e-08/96-569/3-161 // sp|Q008X1|DFP_BOMMO/Putative defense protein OS=Bombyx mori PE=2 SV=1/7e-08/102-569/7-167 // sp|O96382|DFP11_HYPCU/Putative defense protein Hdd11 OS=Hyphantria cunea PE=2 SV=1/2e-07/144-569/16-161 // sp|Q9V3Y3|DFP_DROME/Putative defense protein CG7532 OS=Drosophila melanogaster GN=CG7532 PE=2 SV=1/5e-07/93-548/1-148 // 
TAATAGTGCTGAATTGTTGGATTTTCAAAACTGTCAAGGATTTTTGTCTGCTATTTCGAGGTATACAATTCAGAATGGAAAAATTGCATTGTATATTTAGCGTCTGTGTTTTGTGCGTAGTGCTGGCCGGTGTTCAAAGAACTTCTGCATTTCCTACTGGATCTCCTGTTTCTGCCTGCAGTGACCTCATGCCTGTCCACATAGACGGGGAGCTACAGAAGGGTGCCTCTCCCTACATGATTATCCTCAACGACACAGTGTATGGATCAGCGGCCATTCGAGTCTCTATTACTGCTGTTGGGCCAGCGACCAAAGAGTTTATGGCCTTTATGCTTCAGGCCAGAAACGAAGCCGGATCGCCACAGGGCAACTTTGGAGACGTCCCTGTGGCTTCCAAGGCCATGACGTGCTACAAAGATGGGGACACACTGACCCACACGGCTGCCTTCATCAGACAGTCCATGGAAGTGACCTGGTTCCCGCCTGCCAATAACGTTGGGAAAATAACAATTACAGGTTCTATAGCACTATCCAAGGTAAAATACTGGGCAGTGGAGTCGGCACCAGTCTCGGGCCGGGGAGCCCAGGATGCAAGCACGATTGTTAGAAATTCCTCAGGAAGTGGAACTGTGTCGCATCTGAGCAGCGCCTGTCTCATCCTTCTGACAGTGTTCGCCAAGCTGGCCAATCGTTTTTAACTTTCCTCATAGTGGGGCTGAGGGAGGGGTGGGTTTGGAAGTGTTTTGCCATGTGATACATGTGTGTTAGTTTTATGTACAGATATGACACGGTCTACTTTAAGTATAAAATGTAGTGGAAAGATTATGTTCCTTTTTACTTCGGAACGTAGATGTTAAATACTTTTGTTTCATATTTAATACTTTTGGATTTAAATACTATTAGAAACATTAAACAATAACTTTTTAAAGTGTAATTTTATCAACTTCTTTTTATTCAGTAGAAGATGACATTGCCAAAAAACACACAAGACAAAACCTTAGGTTTCATGTTTTTTACAACAATTACTGTTAACGTCGTCCAGAAATTATTCAAACTTAATTATTAATAGGTTGTCTGTGATTCAGTCTTTAGTGAATTTTTTTTTAAAGGAAACCTCAGATTTTGTACTATGCAATGTTAGCTATGTTATTAAAGAGAGTGTAAATTAGTCTGCTACAACTTTATCACAAAATGCATATTTGGGAAATGTAAATACATTCAAATCTTATTGTTTTTAGAGTTTAAAGTACAGTGCATTTTGCTTAAGGTCCATTGATATCAATGGTCTGCATATTATCAGTAAACACAAAATCTTTTATTTAACGGTAAACTAATACAGTAGCTAATTGATCTTTAAAAAAGGGGCTAAAATAGGAAGAACAACAACTATTTTAGACTGGTTTATCATTTATATGTCTATTCCATTTTTTTATTACTCCCCTTGTTAAAAAACTTTCTATTCAGCCTTAGTTTTATTTTGTAACTTGTTTTTTTTTACCCTTTCATTATTGATTGAAACAAAAGAATTCTTTAAACTAGTTGTGGTTACTGCCTTACGATGTCTAAAGGCCCAGGCTTTTTTTTTGTTCCCCATAAGATGCTAATTATATTCTTCCATCTTAAGAAGCTAAATTTTGTTTCCACTTTTAATGCTTAATGTGAGTAACACCGTCCAAAACAACAAAATAATGACGCGTGTACCAGCGCCGATATAAGCTCAAAGTCTATGCCGAGACAGTGAACTGTCATTTGATTAATGACTGGCTATTAAAACTGTTTTGAAATAAAAAGAACATTGACAAATTTGAGTTTATTTTTTCTGATGTTGTTGGTGTGACAGTTTGAAAATATTGATTCAATAAAATATCGTTACTCG
>Bg-c39982 len=245 count=2 IPR:/ blastx_SP:/
CCTGTTGACGTCTTTTCTTAAGTTTTGACTATTCCATCTCCACTTTCTAAGACCTGTCTAGCTCTGATGTTGGCATCTTTTCTTAGAGTATTTCAATCCATCTCCACTTTCTAAGACCTGCCCCTCCTATAGTTCTCTTCACACATCTCACAGAGCTGGAGCTGCGGCTCTCATGCCAGTCTAGTTAAGATTCTGTGTGCACGGACCTAAAAAACATAAAGTTTCGGGTCCAGAGGAAATCCTTA
>Bg-c6260 len=1107 count=18 IPR:/ blastx_SP:/
CCAATGCAGAATGTGGGGCCGCATTTCAGAAAGCGCAGGCAGGAAGCCAGGCAGGAACAGACAGTAACGGCGCTTCCTTGTATCGTATACCAAATCAATTAATCGGTGACCAAAATGAAATCTATACACCGGAAATCTTGGCCGAGTCTAATAGTACAGAAGTGTTTATGCAGCAGTCAGTTAGAGGGGCCCTTGACATGCTTGTTGGGTATACGCCTTTGTTTCTGTTTGTGTGCGCCGCTATTCTTAAATGCGTCCATGTGCTGTATCTGCTGTACGTGAGCTGGTACGGCGTGAACGCGAGTAACCTCATCCTGTCCTGGAAGAGCGTAAGTCTAATTTCTGAACAAAATGAGCTTGCGAGGAAAGAGTTTGTCCAGGAGCTAGCCTCAAGGTTCCAGTCTGCAAGGCAATTGGCCAAGGGGTACATCGTCTATAAGTTTCTGATCTTCACTGTCTGCTTTGTCATCGCGATGCTTATCTATATATTCCCCGTACCAGAATCCCCTGAAGCAGTCGCGCAATCCGTAAATGGGCAATCTTCTGGCTTCAAAAGCGTGATGCTTATTTGTAGTTTGACTGTTCGCGCCCTCCAGAGAAACAGTGTCTACATGACGCAGTGCACGTTCATAGAGTCTCCGAGTGCTTTTGATGACCAGTCGACGCAGTTCAGAGAAGAGCAACTACTAAGCCTCTACAGGTCATTCTTCTCCGCCCTTCTCTTCGCGTTCGTTCTTATTACCACCGTCAACGCTGTCAACCTGTTCCTGTGGATCGTTAAGCTGTACCGCGGGAAACCTGCAGGACGCTCAAATAAAAGACTGTCTCCGGATTCCAGACTTTTGGTTTGCCTTACAGAAGAGCATCTGGACTTCACAGCCTCCAACAAACTGGAAAAACTCCTGGTTCAAGACCAGAAAGACTCTGCTCCCGACGAAGAACTAACCAAGACATCAGCAGACATAGTTTAGTGGCTTGAGTATTGGTTAATTCGAATACAAGCAAAGAAGTCTCATTGTATACCAATTAAAATTTTACTGACATGCTTGTTACTAGATGTGTATCTTTTGAAGTTTGTATCCAAAAAAATATAGTATCGAAGTCAAG
>Bg-c38533 len=257 count=2 IPR:/ blastx_SP:/
AGAAGTTATTGTTCCAAATTTCCTATAACATGTACAGGGAAATAACACAGGGTGACATTGTTTATGTAAATTAAAGTGTATACTTGTTTATATCATAACTATATAGTCTGTCCCCACTACATCATTCTATATATATATCACTTTCCAAGAAGATGACAGTAATATTTTAATATCTATGTAGAACTTTAAATTCAGATTTCTGCTGTTCAGCTTGCTGGACTGTCTGAATTGGTTAAATCAAGAGATAAATGTACTTA
>Bg-c42712 len=145 count=2 IPR:/ blastx_SP:/
AAAATTATCACCAAAAATTTGTGGGAGTTGCTTTATTGAAACTTAATGCTATTGAATTTTATATTTAAAAAATTGACTTTGTTAATAACCAAGGAAATATTTATGACATTAACCATTTTTTAAAGTAAATTAGGCTACTCTAAGG
>Bg-c41488 len=225 count=2 IPR:/ blastx_SP:/
TTACTGTACTTTGAATTACATGACACTGGCAATATATTTTCATGTACTCTTGAACCTGTTATCCTCATGTTCATAAGTCTAAAAAATGTGAGAAACTGTTGCCACATTTGTAAAAATGTTTACTCTTGCACATTCCATGTACAGCACCTCATATGTACTAGCTTTACTGACTGATATACCAACACAAAATGGCAATGTCATAGTTTGTAAATAATTTAATCTTCA
>Bg-c4311 len=1556 count=28 IPR:/ blastx_SP:/
CGCCCATATCACACCACTAGAAAATAACTTCAGAGCTATTTTAATTAAACCTNCCTAGTGAGGAAAAACAAACTAAATTTTATATGTATAAATATATACAATGATTTAAGCAGAATATACTTCATGTATTGTACATACGAAATAAATAGTACCTTCATAAACTTGTCATAAATACACAGTAAAAACTAGAGCCAATGTGGCAATCTTTTTTAAACAGCAGTGATCCAATAAGACTTTCATTTGATCAATGATATTAATGTTAGGGGGACGGGGACATTGATTGCTACTTTAGCATTGGTAGTATGTGGGTGGGGGGGGGGGGTAAAGGATCACCAATTAATCCATTGTGCCCATATTTGTAAGAGAAATCAATAGGACTTGACATGGCCTACCAGAGGTCAAAGTCATTAAAACTAAGGAATTCGTCAGTTTTGAGCAATCATTGATTAGGTGGGTGTACATTCATTTAGAACTACAAATACAACTCAGTAAAGACACAGAGTTTCGGTAAAGAAACAGCGTTATAGTAAAGTAAGTTATCACTATAACTTGCTGGGTGCCAGATGTCCTCTTAGGCTGTATTCATTTGTTAATAGTACATTTGCAGATTGTTTTTTGCTGCTTCTCATAAACAATCTTTCTAAACTGATTCTTCAAATCAACTATCTCTCTAAACATTTGGTGCATAATTCTATAAAGATGCAGAAGGTCAGAACAAAATGAATGACATTAGCTAGAATTTAAAAACAAAAAAAAAATGAAACTGACAAAACAAACTCTACAGCAGTACAGACTGTCTGGGTCATAAGCTTGGTTTTGTGACTACAACTACTATGCTACCCAACAACCTAATGATATTTCACCCACAGAGAGAACAAAACCAAAGAATGAACTAGTTCTAACCAAAGTGTCAGTGATGCTTTCAATGGGCTCTGGCTACAAAGTGTCTAGCAGACCCATCTACTGCACTACCTTGTCCTTACACTAACACATTTTGTTTTCCCATTAGCAACCAAGTTAACCAAGAGTTTTAATTTGTAACAAGAAGACAAAGAAAACAAAACAGCTACCATGTAGATAGTAGATAAGTTTTGGCATAGCTGCTGAATACATGAACATATAAAATGTAAATTAAAAGTTTTGTTTTGGGTGGGTGGGGGGATGTCTTACATGTTCACAAGTCAACATAATAATGGATAAAATGGTATCAGAAAGTAATACTAGAGTCTCCATAGTTATCAATACCAACATAATAACACAACCAAGAAGTAATTACTCTCTCTCTAATTACTTCAATCTGAATAGTAGAAATAATGAATTTGTAGCTCGTTTTTCTTTCAAAGATATATGAATGTCTACGTTCTTTCAGTTTTTCACATTCCCAAATGCACCGAAGGCTGTGTCTGTCCTCTTCTGAAAAGATGCATTGCCGGACATGCTGTTGGTAGACATCATCTGAATTCCAATGTTGCCCTGTGGCATCGTACTCATCATGCTGGGTTGACCCATTCCCATCATCCCCAAAAGGGTCATGACTGGCCTTTGGTTGGCTGGTA
>Bg-c2939 len=909 count=44 IPR:/ blastx_SP:/
ATTATGTGCAAAAAAAGGTATCGCATTTTTTTTAGTAAAAAAAAAATAAATACATTTGTTGTGTTCTATAAAATGTACATGCCAGTAACTCTACTTGTAGCAGCTTTTGCTTTTTGTGACGCAGCGAATGTAGCCAAGAGAGATGAGCAATGCACCGGGTTGTTACAGTGCGCTAATATAGTTTCGCAACTGGAACCATCTAGCCTTGGTCCAATGCAGGAAGAAGAACGATACGAACACCTATGCCAGATAAGCAATGACCTACGAACGTGTTTTCACAACATCGAAGATGACTGCCAAGATTTAACTTTGTTAAACACGGTCTTAGTAGGAGTTCAGACGTTAGGCTTCCTTTGCTCGGCACAGGGCAAAGCAGAGATTGATCTAGATTCTGTCAAAGACTCTCCATGTCTCAACGATCAAACCACTGCCTTGTCAGCCAAAGAAGGAATGAGCCAGTGCGTGCAGAGTTTCCAGAATGAGTTACAGTTGGCCTCTTTCTCTGTCATGTCTACTGTTGAAGAACTGGAGTCCATCAATGTCTGTTTCTACTTAGATCAACTGAAAAGTTGCATTCTGGGATCAGTGGAAAATGATTGTGGCCAAAACATTAGCATCGTTGCTCGAAAACTTGTGGACATTGGCTACCAGCCTTTTGCCTCGGAGTTGCGCTGTGGTCAACAAGATCGTCACATCCGTTCTCTGAAATCGAGACTTGTTCCCCTGAGTATCTCTGGTGTCAGAAGAAGAAGTTAAATGTCGACCAGCTTCACGGTTCAAATCTTTTGATGTCCAAGATTTAAAAAAAATGAATTTGTCAAGATATTATTTTTTTTAAAAACTATCTTCAATGTATTTCAAAAAAATTCTTTTATATATTATAATAAAGGAATAAAATTTCGATGGTTC
>Bg-c4469 len=1223 count=27 IPR:IPR009068:S15/NS1, RNA-binding; IPR000589:Ribosomal protein S15 blastx_SP:/
CTGCGCCCTTGACAGCTGAATTATTTTCTGTAGCTTTCAAAAGGCTAATATTTAAAAATACGTACCATCGGCGTTACCATGTGTGAACGGAATACAGTTGAATCACGAAGCTACATTCAGTTCATTTGTTGGGTCTAAAGATAACAATGCATCATTTAAATGGCTAAATACCGATCTACAAACACCATTTTTAGCAGCTCAGTTGTATATGTACAACTCTTCAAGAGAGTATGGACACAAACGCCCTCAGAAGCCTCCAAAGGTTACATTCTTTCAGTATTCTGGTGACCTGAGGAAAATTCCAGAAATAGATCCCACTAAACTAGTGTACACATATCAAGGCTTGGAAAAAGAAATTGGCCAAAATGAAGCCATCCAGAAATTGTCAAGTCTAGAATATGCTACAGGTGACGAGAAAGCCAATCATAAAAGAGAGTTGGTTATTGACAGAATTGTTCAACTTTTTGGACCCAACTCTGAAATTGAACAGGAAATTGCGCTGCTGACCTTGGGCATCAGACAAATGATTCCTTACTGTATATCACAAAGACAGGACAAAGGGAATAAGATTTTTCTGCTCAAGCGCATCTTTCGGAGGAGGCGACTAATAACTCGGCTAAGAGAGTTAGACCACGAGAGATTTGAGTGGCTACTGAGAGAATTGAAGATACGGTATGTGCTCCCAAGAGATCGTGAAGAGTACAAAGGGTGGAAATACAACTTAAGAATGAGCACACAGAACGAGGCCGTAGCTAAACAAAGAGAAAAACTAGAGAAATTAAAAGAAAAATTTGAAGCTGAGAAAAATAAATTTTTTGAGAAGAAAGCACAAGTGTTGTCTGAAATTCAGAACGACCTGGAGAAGTTTGGTCTCAGCAGGGACTTCCTGGACCAGCTTCAAGTTGTGGAAAAACAGGAACCAGTGAAAGAAGTGAAAATGAAAGTTGAGAAGGAGGAGCCTGTCAAAGCCTCCAAAAAGAAAAGATAAATCTTGTTTACATTTTGAATAGAGGAACATTTGGTTGGGTGAAAATATGTCAAGTCTAATTCATAAAATGGTTACTCATAACATACATTCATGTTTTCTTTTTTTTTGAATGCATTTGTATGTTCGGTGAATATTCTGAGTGACCAATATAATGAATGGGACACTTAAGTTTTAGAGATGAACAGTAAGAAAGTAAAACAATAAGCAATCAATAAAAGAAGAGGAAAGATAGAAA
>Bg-c40575 len=239 count=2 IPR:/ blastx_SP:/
TCACAAGGCTGGGTGGACGCAAGAACTGATCAAATTTTTTTTTAGGTCTCTAAATTTTGCTTACAATAGTTGAAAGTTTTCGCAGTGTGCCCAAGTATGTCTATATTATTGTTACATTATCTTTGCTGTATGTACCAACCAATATAATTGTGTTAAATAATGATTCTGTTTCAATGACTCTTTTTGCCTTTGTTCATAATTCTTTGTTGAAACTTGATTACATTTGCTTAACAAATACG
>Bg-c24893 len=375 count=3 IPR:/ blastx_SP:/
GGGGGGTGGCTTTTTCCCTTACGTAGGCTGCTCAAACAGCCTATTTTTCAATGGCTAACTATGAGGTTGTCATCTCTCTTGTCTACAATGACGTGTAGGCCTGGCCTGTAACAGCACGCACAATGTAGAGTACCAGGACCATCTAACACAGTGACGCAATATTCATACAAAGTACGACGAGATGGTTCATAAATCACCTTGCTGACCTTCCACCGTCCCTCCGCCAGATAATAAACCTTAAGAGTCACTACTGGCTCTGTTCCCAGAGGTTCTCATTTCGTAAAGCTGTGGCGGGCTCATTACATCATTCCATCATTTTATCAATTAAAAATTATGTTTAACTTTTTTTTTTGTTCCAATCCTATTCGTTCTTTT
>Bg-c40672 len=238 count=2 IPR:IPR001506:Peptidase M12A, astacin blastx_SP:sp|P98068|SPAN_STRPU/Protein SpAN OS=Strongylocentrotus purpuratus GN=SPAN PE=2 SV=1/9e-20/2-238/202-280 // sp|P42674|BP10_PARLI/Blastula protease 10 OS=Paracentrotus lividus GN=BP10 PE=2 SV=1/8e-19/2-238/202-280 // sp|P55115|NAS15_CAEEL/Zinc metalloproteinase nas-15 OS=Caenorhabditis elegans GN=nas-15 PE=2 SV=2/4e-14/2-238/217-293 // sp|Q20191|NAS13_CAEEL/Zinc metalloproteinase nas-13 OS=Caenorhabditis elegans GN=nas-13 PE=2 SV=5/7e-12/2-238/213-289 // sp|P55112|NAS4_CAEEL/Zinc metalloproteinase nas-4 OS=Caenorhabditis elegans GN=nas-4 PE=2 SV=4/4e-11/2-238/199-276 // 
ACAATCACGTCCAGATCGCGATACATATATCACTGTCAATTATGCTAACATTCAAACAGGCAAAGAACATAACTTCAATAAATATGCTTGGGGTAGTACTGTGCTCAATCAAAATACACCTTATGATATGGGAAGTATTATGCATTATGCTGCCAATTCATTCAGTAGCAATGGACAACCAACAATTACAGCGAAACAAGCTACTGGTGTTACAATGGGTCAACGTACAAAGCTTAGT
>Bg-c3550 len=329 count=36 IPR:/ blastx_SP:/
TTTTTCTAGCGGCTAGCTAGATCTATATCTGTTGCGAGAACTAGATCTACTGTTGTAGTGCATTCTTATTACTAGACTTCTTGTCTAAGGACACTCGAGGTGTTGCATGGTCGAACACGTTGAGTTAGAGATCGTCAACAAGATTCACGTTTCACTTGTGTGGTGACGAAATAGTTGAACCCTAGGCCTAGCTCTGCACCTGTTTGTGGCAACTGATTGCCATTATCATGGCAGTGCCTGACAACAACAATTCCAAACTTACAATCGAACATCCTCAATCAATGTCAGATGTACTCACGAATTCATCCCTGCGTCGGATGCGTTGTTAC
>Bg-c14516 len=734 count=6 IPR:/ blastx_SP:/
TACAGAAATATTATATCTGTCCTTTTATCTTTATCCTTATTTGTAGGGAAATTGAAAGACAAATGTATTCTTATTTTCTTTTGAATGTTACAGTGAATGTGCCATACATATTAAAGACGATGCTTAAGAGTACAGTTCGGTTTACTATAAAGTTGCTAAATTTTCTATATATATTAATACTTTTTGTACATTTATCATCGTTTATTTTGTTTTGTTTGGATTGGTGTTACAAATAACTTTTATTTATGCTCTGTTTTTTGTTTATGAACACACAGACTTGTATAGTGTTAGATATTGTCCAATGATGTGATAAACCAGAAACACTAAGGTTCAGTGTTCTACTTTTACAGATGTTAATGAATATTTGTTTACAATATAAATTTGGTCTTGATGTAGCTCTCTCTCTCTCTCTCTCTCTCTCTCTCTGTTGATCAAATTGATCAAATGAACTAAGTTAAAGTAAGGATTTTTTTTTTTTTAATTTGTTGATTCTACACCCCCACTACCTCCCCTCTACCTTTTCACTCCTTGACCCAATTGCCAGTTAGGATTCAGTTGTAGATTCTCTCTTTGTTGTAAGCATTGAATTTTCAGTGGCATTGTTCAATTTCATATTGACAGTACTTCTGCTAGCCAGCCTTTTTTTTTTAAATTGCTGAGCAAAACTAAATATGTACATGTGGTTTTTAATTTGTTTAAATCTAAATTGTATTATCAATATAGGACCTAAGTGT
>Bg-c15043 len=473 count=6 IPR:/ blastx_SP:/
TAAATTTTGATAAAAAAATAAAAGCTTCGTCCAGTAGCCCAGTGTCTTTCACATGTACAAGACAGTTGTGAAGTAGTAGCTTATGTTAGTGAAGTGTTTGGGATTGTATGAGTGATACTCTGAATGCTAAACATAGAATGAACCATTATAACAGTGGAAACCGCCTTAATGTATGATTATTATGCAACCTTGTGAATATGTGGAACGATCGTTTTAATAGAGTAGAAGGTACTTTTGTGTTGGCTTTTATTTGATATACGATTTTAATAGCATTAACGAGAAATACAATGGACTGGATTCCACTGTAGATGTATAGTAGATCTATAATACATTTTTTATATGTGTACAAATTACGGATGTTTTAAAACTACATATCGCCTATACATTTATAATAAATCAGGGATGTGTCTTTTAAGTATGAATTTAAAAAAGCATTGTGATTTGTAATTTAAATAAAGAGGAATGTGTCACTA
>Bg-c11371 len=374 count=9 IPR:/ blastx_SP:/
AGTAAAAAAGCTGTTCATTAAAAGACATTTGATTGTATGAAATTTAATATCTGCCACACCTGGCTTGAACAAAATAATTTACAAGTCTTAATAATTCATGAAGTAAGTGAATGATCGAACTAAGTGGATCATACATTTGAAATGTAAAATAAAGTCTACAAATTGTGACTTCTTAGACTACAGGTTGGGATACAGACATAAGTTTAAAATAGTATCAGTCCATCAGACAATAGGTTGATACAGGAACCAGCTAATATGGCAGGTTTTTACAGACCATCGTCATAATCAAATTAACCCGTATTAATTAGCCTTTAGTTTATGACTAATTTTTTCTTTTTTTCAAATATTTGTGCGTGTTTGTTTCAAAAACCAAA
>Bg-c2315 len=1437 count=57 IPR:/ blastx_SP:/
[truncated: 1,200,923 more chars]
