# Supplementary material for: Specific versus Non-Specific Immune Responses in an Invertebrate Species Evidenced by a Comparative de novo Sequencing Study
Source: PLoS One. 2012 Mar 12;7(3):e32512. doi: 10.1371/journal.pone.0032512 (PMC3299671; doi:10.1371/journal.pone.0032512)
Supplement: Text S2 — List of the candidate unisequences belonging to the expression clusters (codes as deposited at http://www.snaildb.org/). (RTF) [file pone.0032512.s006.rtf]

Cluster 1:

Bg-c1116
Bg-c2844
Bg-SRR000076.102245-1
Bg-CX727676-1
Bg-SRR005375.179469-1
Bg-c434
Bg-c1420
Bg-c6005
Bg-c915
Bg-c496
Bg-c453
Bg-SRR000072.8066-1
Bg-SRR005372.33323-1
Bg-c9303
Bg-c5360
Bg-c28889
Bg-c24926
Bg-c288
Bg-c20601
Bg-SRR000072.107593-1
Bg-c4159
Bg-AA547689-1
Bg-c649
Bg-SRR000072.12876-1
Bg-SRR005372.29491-1
Bg-c3233
Bg-SRR005375.200549-1
Bg-SRR000076.164736-1
Bg-c31812
Bg-SRR000076.100634-1
Bg-c687
Bg-c25704
Bg-SRR000076.120473-1
Bg-AW739711-1
Bg-c10721
Bg-c13614
Bg-c157
Bg-SRR005372.15795-1
Bg-c29445
Bg-SRR005372.83689-1
Bg-SRR000076.185129-1
Bg-c43
Bg-SRR005372.54308-1
Bg-c30647
Bg-c11261
Bg-c5729
Bg-c37378
Bg-c23656
Bg-SRR000076.20084-1
Bg-SRR000072.33169-1
Bg-SRR005372.20202-1
Bg-c853
Bg-c15911
Bg-c25994
Bg-c5983
Bg-SRR000072.139258-1
Bg-SRR005375.127416-1
Bg-c12533
Bg-c3142
Bg-ci9
Bg-SRR000076.12386-1
Bg-c3103
Bg-c9138
Bg-c14944
Bg-DW474243-1
Bg-SRR000076.164202-1
Bg-SRR000072.157748-1
Bg-c12802
Bg-c876
Bg-c4401
Bg-c30592
Bg-c15676
Bg-c12635
Bg-c37996
Bg-c16975
Bg-SRR000072.143556-1
Bg-c827
Bg-c307
Bg-c1994
Bg-c32566
Bg-SRR005375.37581-1
Bg-c11963
Bg-c23934
Bg-c10876
Bg-c179
Bg-c38923
Bg-c36442
Bg-c37110
Bg-ES484903-1
Bg-SRR000076.7130-1
Bg-c39
Bg-c928
Bg-ES489049-1
Bg-SRR000076.150640-1
Bg-c31265
Bg-c2688
Bg-c7031
Bg-SRR000072.29011-1
Bg-SRR000076.102544-1
Bg-c12108
Bg-EW999478-1
Bg-c14470
Bg-c447
Bg-c22510
Bg-c2187
Bg-c361
Bg-SRR005372.64691-1
Bg-c19957
Bg-c1654
Bg-c42763
Bg-c42913
Bg-c39537
Bg-c18036
Bg-ES485057-1
Bg-c1819
Bg-CK989855-1
Bg-c16341
Bg-CK656804-1
Bg-SRR000076.195161-1
Bg-SRR005372.17457-1
Bg-c9256
Bg-SRR000076.52914-1
Bg-c26105
Bg-SRR005372.58040-1
Bg-SRR000072.123807-1
Bg-SRR000076.141011-1
Bg-c7912
Bg-c13256
Bg-c22116
Bg-c6315
Bg-SRR000076.27783-1
Bg-EV815215-1
Bg-c1759
Bg-ES750489-1
Bg-c2326
Bg-c7606
Bg-c8436
Bg-c25903
Bg-c32849
Bg-SRR000076.81722-1
Bg-SRR005372.7973-1
Bg-c23381
Bg-c391
Bg-c40441
Bg-SRR005375.127550-1
Bg-c14431
Bg-ES491678-1
Bg-c25986
Bg-c30211
Bg-SRR000076.125862-1
Bg-EX002591-1
Bg-EE049672-1
Bg-c1093
Bg-c4557
Bg-c6571
Bg-SRR000072.103748-1
Bg-c19617
Bg-c34465
Bg-CK989013-1
Bg-c744
Bg-c8434
Bg-SRR000074.997-1
Bg-c11901
Bg-SRR000072.88217-1
Bg-SRR000073.855-1
Bg-c6576
Bg-c337
Bg-c30295
Bg-c5841
Bg-SRR005375.158889-1
Bg-c15299
Bg-c114
Bg-SRR000073.986-1
Bg-SRR000072.103577-1
Bg-c24393
Bg-c1325
Bg-c41374
Bg-c14501
Bg-c6588
Bg-c1805
Bg-CK640653-1
Bg-c8157
Bg-c32979
Bg-SRR000076.91931-1
Bg-EX000326-1
Bg-c35455
Bg-c20532
Bg-ES488102-1
Bg-EX001061-1
Bg-ES485142-1
Bg-SRR000076.10462-1
Bg-CK988681-1
Bg-EE722667-1
Bg-c2347
Bg-SRR000072.8531-1
Bg-c24068
Bg-c737
Bg-EV820365-1
Bg-ci65
Bg-c206
Bg-c26820
Bg-EX003900-1
Bg-ES490320-1
Bg-EV819272-1
Bg-c2730
Bg-ES488779-1
Bg-EV813834-1
Bg-ES482876-1
Bg-c26020
Bg-c14698
Bg-SRR005372.27209-1
Bg-SRR005372.40298-1
Bg-c92
Bg-SRR000072.68642-1
Bg-SRR000072.118164-1
Bg-c214
Bg-c4831
Bg-SRR005372.71830-1
Bg-SRR000076.196181-1
Bg-SRR005372.61126-1
Bg-c15822
Bg-c4532
Bg-c37752
Bg-c18205
Bg-ES488243-1
Bg-c1333
Bg-SRR000076.112101-1
Bg-SRR000076.109470-1
Bg-SRR000076.144192-1
Bg-c28807
Bg-c196
Bg-c598
Bg-c16177
Bg-SRR000076.90397-1
Bg-c14414
Bg-c30243
Bg-SRR000072.1246-1
Bg-c23257
Bg-SRR000072.190-1
Bg-SRR000072.47680-1
Bg-SRR005372.14444-1
Bg-SRR005375.17334-1
Bg-ES486630-1
Bg-AW739888-1
Bg-c1658
Bg-c30049
Bg-c5570
Bg-ES486480-1
Bg-c41475
Bg-c28252
Bg-c12061
Bg-c6611
Bg-EW998868-1
Bg-ES490516-1
Bg-ES484888-1
Bg-c41205
Bg-SRR000076.48535-1
Bg-c4643
Bg-SRR000072.121642-1
Bg-SRR000074.1738-1
Bg-SRR005375.153084-1
Bg-AW739693-1
Bg-c1235
Bg-c19780
Bg-c25900
Bg-SRR000072.130916-1
Bg-SRR000076.34345-1
Bg-ES746847-1
Bg-c1523
Bg-c2594
Bg-c1537
Bg-c6342
Bg-SRR000076.16664-1
Bg-c336
Bg-SRR000076.15081-1
Bg-SRR005375.92671-1
Bg-c20273
Bg-SRR000076.52269-1
Bg-c11803
Bg-c27572
Bg-c31016
Bg-c2715
Bg-c7351
Bg-c24887
Bg-c1142
Bg-c4822
Bg-c8711
Bg-c30118
Bg-c39311
Bg-c3729
Bg-EV822743-1
Bg-c23661
Bg-SRR000072.66068-1
Bg-SRR005372.84312-1
Bg-c473
Bg-c2349
Bg-c25616
Bg-SRR000076.131766-1
Bg-c1239
Bg-c1724
Bg-c807
Bg-c26108
Bg-c1087
Bg-c6308
Bg-c10091
Bg-c4327
Bg-c25368
Bg-c160
Bg-c30387
Bg-SRR005375.42113-1
Bg-SRR000076.128528-1
Bg-c6161
Bg-EE049549-1
Bg-c29589
Bg-c41259
Bg-ES488029-1
Bg-c31505
Bg-c9764
Bg-EV821557-1
Bg-EV813636-1
Bg-ES272255-1
Bg-ES486728-1
Bg-c23566
Bg-GH716940-1
Bg-SRR005372.47568-1
Bg-c1392
Bg-EV818889-1
Bg-c30241
Bg-c12978
Bg-c3700
Bg-c639
Bg-c1683
Bg-SRR005372.28622-1
Bg-SRR005372.67767-1
Bg-EV819956-1
Bg-EX005311-1
Bg-CN655053-1
Bg-c1496
Bg-c43000
Bg-c30657
Bg-ES484966-1
Bg-c558
Bg-ES488849-1
Bg-c26078
Bg-c11145
Bg-ES272342-1
Bg-c516
Bg-ES491828-1
Bg-SRR000076.108584-1
Bg-c29407
Bg-ES486512-1
Bg-ES488819-1
Bg-c16660
Bg-c1303
Bg-c10916
Bg-c320
Bg-c6702

Cluster 2: 

Bg-c6702
Bg-c2084
Bg-c27802
Bg-c9226
Bg-SRR000076.184156-1
Bg-c2834
Bg-EV817610-1
Bg-c19113
Bg-SRR005372.84866-1
Bg-SRR005375.209961-1
Bg-SRR005375.216463-1
Bg-SRR005375.235629-1
Bg-SRR005375.235896-1
Bg-c27319
Bg-c9824
Bg-c28184
Bg-c5050
Bg-SRR005372.26623-1
Bg-EV817413-1
Bg-SRR005372.24553-1
Bg-c27463
Bg-SRR000076.106312-1
Bg-SRR000076.132082-1
Bg-c2198
Bg-c10175
Bg-CK149392-1
Bg-c319
Bg-CK988984-1
Bg-c41278
Bg-c1378
Bg-CK989898-1
Bg-c7684
Bg-c2720
Bg-c31102
Bg-c34418
Bg-c15752
Bg-c23055
Bg-SRR000073.1599-1
Bg-SRR000072.140609-1
Bg-c1290
Bg-c33425
Bg-c23340
Bg-c23794
Bg-c5178
Bg-c1954
Bg-c40833
Bg-ES491715-1
Bg-SRR000072.117004-1
Bg-c999
Bg-SRR000072.143874-1
Bg-c736
Bg-c8804
Bg-EX001309-1
Bg-EV814317-1
Bg-c4472
Bg-c4403
Bg-c10809
Bg-SRR000076.63429-1
Bg-SRR000072.132471-1
Bg-c4791
Bg-c260
Bg-c29569
Bg-c2282
Bg-c1981
Bg-SRR005375.216413-1
Bg-c4029
Bg-c678
Bg-c14192
Bg-SRR000076.194503-1
Bg-c16053
Bg-c23706
Bg-c1267
Bg-c4280
Bg-SRR005375.62867-1
Bg-EW999603-1
Bg-c2982
Bg-SRR005372.79091-1
Bg-c1865
Bg-SRR000072.3546-1
Bg-c9304
Bg-c3107
Bg-ES488248-1
Bg-SRR005372.23109-1
Bg-SRR000072.45339-1
Bg-c3119
Bg-c11805
Bg-c19594
Bg-c1945
Bg-SRR005372.34844-1
Bg-c36570
Bg-SRR005372.21433-1
Bg-SRR005372.50015-1
Bg-SRR005372.73318-1
Bg-SRR000072.20906-1
Bg-AW740418-1
Bg-c31177
Bg-c33920
Bg-c10700
Bg-SRR000076.6223-1
Bg-c14003
Bg-c13351
Bg-c7320
Bg-SRR000076.198907-1
Bg-c962
Bg-c11664
Bg-c5169
Bg-c12211
Bg-ci87
Bg-CK990110-1
Bg-c305
Bg-c23287
Bg-c1159
Bg-c145
Bg-c29014
Bg-SRR000076.89291-1
Bg-AW739716-1
Bg-SRR000072.143268-1
Bg-CV548318-1
Bg-c11398
Bg-SRR005375.182889-1
Bg-c29757
Bg-c30100
Bg-GH716259-1
Bg-c35319
Bg-c5464
Bg-c636
Bg-SRR000072.125275-1
Bg-c81
Bg-c14923
Bg-SRR000076.34773-1
Bg-SRR005372.26810-1
Bg-c1866
Bg-SRR005375.84107-1
Bg-c23744
Bg-c18059
Bg-c41858
Bg-c3062
Bg-SRR000072.66442-1
Bg-c2501
Bg-c22454
Bg-c31175
Bg-c470
Bg-c18086
Bg-SRR000072.30516-1
Bg-c5619
Bg-SRR005372.82220-1
Bg-c31132
Bg-c728
Bg-SRR005375.75263-1
Bg-c465
Bg-c6602
Bg-c1935
Bg-c9630
Bg-c38313
Bg-c4514
Bg-c4561
Bg-SRR000076.10114-1
Bg-SRR005375.120957-1
Bg-c17802
Bg-c633
Bg-ES491556-1
Bg-c25280
Bg-c8915
Bg-c2244
Bg-c1184
Bg-c11808
Bg-c987
Bg-c3212
Bg-SRR000076.60662-1
Bg-c31456
Bg-FC855402-1
Bg-c25689
Bg-c14427
Bg-c2300
Bg-SRR000076.104656-1
Bg-c8653
Bg-SRR000074.3958-1
Bg-c36
Bg-c607
Bg-c8916
Bg-SRR000072.85140-1
Bg-c38995
Bg-SRR005372.17605-1
Bg-SRR005375.48109-1
Bg-SRR005375.60900-1
Bg-SRR005375.112779-1
Bg-c15853
Bg-c25815
Bg-c6415
Bg-c19215
Bg-SRR000072.151878-1
Bg-SRR005375.235317-1
Bg-EX002155-1
Bg-c289
Bg-EX003959-1
Bg-c6918
Bg-c432
Bg-c31937
Bg-SRR000076.168864-1
Bg-c5661
Bg-c10029
Bg-c1345
Bg-c120
Bg-c1648
Bg-SRR000072.18772-1
Bg-c8717
Bg-c8169
Bg-c563
Bg-c4483
Bg-c19124
Bg-c6597
Bg-c794
Bg-c1590
Bg-c1012
Bg-c3927
Bg-SRR000076.68029-1
Bg-c13231
Bg-c4949
Bg-SRR000072.134508-1
Bg-c924
Bg-c955
Bg-c19045
Bg-c1250
Bg-c6103
Bg-c13530
Bg-SRR000076.153543-1
Bg-EE049573-1
Bg-c1547
Bg-c785
Bg-c6908
Bg-c3314
Bg-SRR000072.107810-1
Bg-c1743
Bg-SRR000072.163113-1
Bg-SRR000076.15174-1
Bg-SRR005372.67159-1
Bg-c11650
Bg-SRR000072.106717-1
Bg-c5912
Bg-EW996690-1
Bg-c3508
Bg-SRR000072.10802-1
Bg-c25005
Bg-c16482
Bg-c1522
Bg-SRR000075.2448-1
Bg-c13238
Bg-c923
Bg-c26155
Bg-c1685
Bg-c9789
Bg-SRR000072.25971-1
Bg-SRR000072.105402-1
Bg-SRR000076.20754-1
Bg-c3679
Bg-c76
Bg-c4442
Bg-c7964
Bg-c1572
Bg-c17938
Bg-SRR000072.108544-1
Bg-c3114
Bg-SRR000076.51699-1
Bg-FC856097-1
Bg-SRR000072.151031-1
Bg-SRR000072.161926-1
Bg-c37884
Bg-c40030
Bg-SRR005375.71577-1
Bg-c1592
Bg-c34185
Bg-SRR005375.199679-1
Bg-c23944
Bg-c509
Bg-c954
Bg-ci34
Bg-c1341
Bg-c2797
Bg-SRR005375.126752-1
Bg-c3087
Bg-c2546
Bg-c9567
Bg-SRR000076.177855-1
Bg-c23516
Bg-c36615
Bg-c2829
Bg-c2862
Bg-c1264
Bg-c138
Bg-ES751507-1
Bg-c201
Bg-SRR005372.63526-1
Bg-c16028
Bg-SRR005372.88067-1
Bg-CK989051-1
Bg-c3236
Bg-c8805
Bg-c29760
Bg-c42355
Bg-SRR000072.61369-1
Bg-SRR000072.107693-1
Bg-SRR000076.183614-1
Bg-SRR000076.194449-1
Bg-SRR000076.192019-1
Bg-c1551
Bg-c228
Bg-c488
Bg-c1600
Bg-c185
Bg-ci3
Bg-c11071
Bg-c2313
Bg-SRR000072.84913-1
Bg-SRR000072.56958-1
Bg-c9843
Bg-SRR000072.5653-1
Bg-SRR000072.52515-1
Bg-SRR000072.90823-1
Bg-SRR000075.913-1
Bg-SRR000076.98067-1
Bg-SRR005372.2588-1
Bg-c62
Bg-SRR000076.186282-1
Bg-c169
Bg-c40675
Bg-c852
Bg-SRR000072.128996-1
Bg-SRR000076.113779-1
Bg-c28
Bg-SRR000076.122928-1
Bg-c3896
Bg-c7544
Bg-c664
Bg-c11199
Bg-c2877
Bg-c466
Bg-EX005854-1
Bg-SRR005375.128167-1
Bg-SRR005375.143705-1
Bg-c2853
Bg-c6601
Bg-SRR000076.165846-1
Bg-c1437
Bg-EW996741-1
Bg-c368
Bg-c25571
Bg-c39837
Bg-c1255
Bg-CO870301-1
Bg-c19084
Bg-c121
Bg-SRR000076.41536-1
Bg-c19340
Bg-c17291
Bg-CK800799-1
Bg-EV823172-1
Bg-c2436
Bg-c23912
Bg-FC857931-1
Bg-c14709
Bg-c6427
Bg-c35837
Bg-SRR000072.149961-1
Bg-c1643
Bg-c906
Bg-c3413
Bg-c1505
Bg-SRR000076.198273-1
Bg-FC856704-1
Bg-SRR005375.33591-1
Bg-c2004
Bg-c4956
Bg-SRR000076.32563-1
Bg-EV817148-1
Bg-EV822754-1
Bg-ES486521-1
Bg-c8256
Bg-c21763
Bg-c5519
Bg-ci80
Bg-c2012
Bg-c382
Bg-c1566
Bg-c1720
Bg-SRR000076.162723-1
Bg-c448
Bg-c11316
Bg-c13587
Bg-c6022
Bg-SRR000076.94352-1
Bg-c38554
Bg-c19521
Bg-c193
Bg-c23805
Bg-c227
Bg-SRR005372.15566-1
Bg-CK990128-1
Bg-c9593
Bg-SRR000072.18617-1
Bg-c267
Bg-c18264
Bg-ES748451-1
Bg-c24291
Bg-SRR000072.149137-1
Bg-SRR005375.189700-1
Bg-c20997
Bg-c1340
Bg-c19192
Bg-SRR000072.28776-1
Bg-c8084
Bg-SRR000072.146299-1
Bg-c32055
Bg-FC859428-1
Bg-c33311
Bg-c43057
Bg-GH716564-1
Bg-ES491699-1
Bg-c4113
Bg-c10407
Bg-SRR000072.62030-1
Bg-c184
Bg-c20780
Bg-DT724782-1
Bg-DT725015-1
Bg-ES491892-1
Bg-c29064
Bg-c30187
Bg-c33135
Bg-c3196
Bg-c976
Bg-c26
Bg-c23249
Bg-SRR000076.80195-1
Bg-c415
Bg-c10090
Bg-c132
Bg-c18037
Bg-c22893
Bg-FC856323-1

Cluster 3

Bg-c19295
Bg-SRR000076.167204-1
Bg-c69
Bg-SRR005375.2588-1
Bg-c783
Bg-SRR005375.134069-1
Bg-c632
Bg-c39242
Bg-c16535
Bg-c30220
Bg-c762
Bg-CK327224-1
Bg-SRR000072.102814-1
Bg-c2311
Bg-SRR005372.9631-1
Bg-c21559
Bg-c15542
Bg-c1930
Bg-c8543
Bg-ES745865-1
Bg-SRR005372.73244-1
Bg-CK989481-1
Bg-c1836
Bg-SRR000072.107318-1
Bg-c10948
Bg-c1288
Bg-EW998499-1
Bg-SRR000076.97891-1
Bg-c13432
Bg-c16099
Bg-SRR005372.80384-1
Bg-c6497
Bg-c2482
Bg-SRR000076.27998-1
Bg-SRR005372.38449-1
Bg-FC856310-1
Bg-DW474825-1
Bg-c9241
Bg-EW998653-1
Bg-c16030
Bg-c40711
Bg-c525
Bg-c41228
Bg-SRR000076.153706-1
Bg-SRR000072.152619-1
Bg-c30249
Bg-c377
Bg-SRR005372.35572-1
Bg-c139
Bg-c28097
Bg-c12928
Bg-c31804
Bg-c30073
Bg-c3909
Bg-SRR000072.123120-1
Bg-c867
Bg-SRR000076.67728-1
Bg-FC855664-1
Bg-c19841
Bg-SRR000076.12295-1
Bg-c5137
Bg-SRR000072.160669-1
Bg-c1962
Bg-c5176
Bg-c1038
Bg-c7819
Bg-SRR000076.57866-1
Bg-c1567
Bg-SRR000076.34297-1
Bg-c806
Bg-c95
Bg-SRR005375.135834-1
Bg-c11015
Bg-c9246
Bg-SRR000076.43607-1
Bg-c29383
Bg-c36524
Bg-SRR000072.40831-1
Bg-c3239
Bg-SRR005372.82498-1
Bg-SRR005375.134939-1
Bg-c686
Bg-EV815167-1
Bg-BG360442-1
Bg-SRR000072.149582-1
Bg-SRR000072.160163-1
Bg-ci58
Bg-c24676
Bg-c17388
Bg-c908
Bg-c144
Bg-c680
Bg-c35940
Bg-SRR005372.40954-1
Bg-EV814114-1
Bg-c386
Bg-c2503
Bg-c9545
Bg-SRR000076.70253-1
Bg-c33608
Bg-c42056
Bg-c2760
Bg-c670
Bg-c1497
Bg-c17428
Bg-DN966885-1
Bg-c4652
Bg-c6299
Bg-SRR000076.187329-1
Bg-c559
Bg-c23378
Bg-c773
Bg-c837
Bg-c3964
Bg-c7271
Bg-SRR005372.80123-1
Bg-CK989735-1
Bg-EV822286-1
Bg-c791
Bg-c12932
Bg-SRR000076.5282-1
Bg-c2717
Bg-c1127
Bg-SRR000076.196588-1
Bg-SRR000072.58231-1
Bg-c4316
Bg-c3861
Bg-c16776
Bg-SRR000076.152612-1
Bg-c849
Bg-c38271
Bg-c32
Bg-c468
Bg-SRR000072.155091-1
Bg-SRR005372.8199-1
Bg-SRR005375.93361-1
Bg-c42385
Bg-c16585
Bg-c4789
Bg-c20
Bg-c302
Bg-SRR000072.152924-1
Bg-c456
Bg-c898
Bg-c24
Bg-c13144
Bg-c191
Bg-SRR000076.113536-1
Bg-CK988916-1
Bg-AW740522-1
Bg-c21464
Bg-c32781
Bg-c29244
Bg-c27128
Bg-c2070
Bg-c16133
Bg-c23893
Bg-SRR000074.1316-1
Bg-c403
Bg-c30374
Bg-SRR000076.139954-1
Bg-c31539
Bg-c31535
Bg-c30219
Bg-SRR000076.185795-1
Bg-c5295
Bg-c106
Bg-c2860
Bg-c472
Bg-c370
Bg-c383
Bg-c1048
Bg-EV814918-1
Bg-c11053
Bg-c13440
Bg-c22400
Bg-c7177
Bg-SRR000072.105721-1
Bg-SRR000074.2701-1
Bg-SRR000076.88952-1
Bg-SRR000076.129059-1
Bg-SRR000076.159861-1
Bg-SRR005372.32252-1
Bg-SRR005372.33822-1
Bg-SRR005372.49458-1
Bg-SRR005375.49191-1
Bg-SRR005375.92183-1
Bg-SRR005375.200635-1
Bg-EX002253-1
Bg-AW739635-1
Bg-c34651
Bg-c35706
Bg-c36851
Bg-c3028
Bg-SRR000072.29484-1
Bg-SRR000072.146485-1
Bg-SRR005375.143723-1
Bg-c36628
Bg-c35686
Bg-c41466
Bg-CK988833-1
Bg-c30120
Bg-c140
Bg-c23522
Bg-SRR000072.12689-1
Bg-c16203
Bg-c19659
Bg-SRR000073.2936-1
Bg-c1539
Bg-c2101
Bg-c4500
Bg-c32122
Bg-c37253
Bg-c23157
Bg-c534
Bg-c2531
Bg-c6132
Bg-c8658
Bg-c23411
Bg-c23671
Bg-CV548559-1
Bg-c37403
Bg-c515
Bg-SRR005375.131070-1
Bg-c19839
Bg-SRR005372.39988-1
Bg-SRR005372.42755-1
Bg-c37371
Bg-c36286
Bg-c39203
Bg-c24586
Bg-SRR005372.80750-1
Bg-c1789
Bg-c5850
Bg-c13251
Bg-ES484251-1
Bg-c16
Bg-SRR000072.13066-1
Bg-c5927
Bg-c2517
Bg-SRR000076.172600-1
Bg-c354
Bg-c6124
Bg-c3043
Bg-SRR000074.3879-1
Bg-BI936365-1
Bg-SRR000072.13403-1
Bg-c1276
Bg-c4917
Bg-c16165
Bg-SRR000072.107780-1
Bg-c11016
Bg-c467
Bg-c931
Bg-c29988
Bg-c314
Bg-c19122
Bg-SRR000072.105376-1
Bg-SRR005375.157272-1
Bg-EE722859-1
Bg-c39297
Bg-c25508
Bg-c14385
Bg-c19549
Bg-EE723557-1
Bg-SRR000072.69012-1
Bg-c22094
Bg-SRR000076.170085-1
Bg-SRR005375.35287-1
Bg-c29276
Bg-c3919
Bg-SRR005372.76075-1
Bg-EV823131-1
Bg-c5699
Bg-c36266
Bg-c172
Bg-c7338
Bg-c230
Bg-c809
Bg-ci85
Bg-c21
Bg-SRR000072.6700-1
Bg-SRR000076.186058-1
Bg-c675
Bg-c3171
Bg-c26490
Bg-c33369
Bg-SRR000076.112269-1
Bg-SRR000076.147067-1
Bg-AW740094-1
Bg-CB381769-1
Bg-c36223
Bg-c3147
Bg-c10146
Bg-c32567
Bg-SRR000075.1115-1
Bg-SRR000072.55333-1
Bg-c20342

Cluster 4:

Bg-c27937
Bg-c3050
Bg-c2215
Bg-SRR000072.162358-1
Bg-c692
Bg-c581
Bg-SRR000076.159179-1
Bg-c74
Bg-SRR005372.68763-1
Bg-c6583
Bg-c355
Bg-c15337
Bg-c8408
Bg-SRR000072.53573-1
Bg-SRR000072.92559-1
Bg-SRR005375.153450-1
Bg-c697
Bg-c30464
Bg-EV823036-1
Bg-EV822925-1
Bg-c2561
Bg-SRR000072.23379-1
Bg-SRR000076.189108-1
Bg-c23
Bg-c31903
Bg-c11067
Bg-c1094
Bg-c22855
Bg-c8875
Bg-c3081
Bg-SRR000076.169263-1
Bg-c23505
Bg-c19339
Bg-c24304
Bg-c96
Bg-GH716946-1
Bg-c7453
Bg-c297
Bg-SRR000076.108357-1
Bg-c803
Bg-SRR000076.36170-1
Bg-c32422
Bg-c30105
Bg-CK989728-1
Bg-SRR000076.182263-1
Bg-SRR000072.136885-1
Bg-c31489
Bg-c29328
Bg-c41741
Bg-c20730
Bg-c32478
Bg-EW996926-1
Bg-c12951
Bg-DW474711-1
Bg-c3746
Bg-c5309
Bg-EW999996-1
Bg-c10033
Bg-c1611
Bg-c983
Bg-c1419
Bg-c12083
Bg-c85
Bg-c2099
Bg-c4610
Bg-c13545
Bg-c1225
Bg-SRR000072.48302-1
Bg-c255
Bg-c1197
Bg-c242
Bg-CK656593-1
Bg-c476
Bg-c4126
Bg-ES272514-1
Bg-c2798
Bg-SRR000076.144399-1
Bg-c1133
Bg-c12463
Bg-c8456
Bg-SRR000076.181360-1
Bg-c22141
Bg-c27248
Bg-c97
Bg-c1937
Bg-c25506
Bg-SRR005372.85903-1
Bg-EW997042-1
Bg-c37550
Bg-CK989230-1
Bg-c1700
Bg-c23267
Bg-SRR005372.44787-1
Bg-c13
Bg-c13136
Bg-c28764
Bg-SRR005372.38832-1
Bg-c37409
Bg-c23283
Bg-SRR005375.85819-1
Bg-SRR000076.40829-1
Bg-SRR000076.164813-1
Bg-c33615
Bg-c31925
Bg-c2446
Bg-SRR000076.182636-1
Bg-c21233
Bg-c1516
Bg-c246
Bg-c2209
Bg-SRR005372.87015-1
Bg-SRR005375.226924-1
Bg-c37581
Bg-c20651
Bg-c10228
Bg-c13675
Bg-SRR000076.192805-1
Bg-SRR000076.59050-1
Bg-c36078
Bg-SRR000076.182571-1
Bg-c183
Bg-c2625
Bg-c3115
Bg-c8631
Bg-c8816
Bg-SRR000076.118912-1

Cluster 5: 

Bg-c712
Bg-c11763
Bg-c90
Bg-CK085009-1
Bg-c41779
Bg-c19233
Bg-c25152
Bg-c19712
Bg-SRR000072.101258-1
Bg-SRR000073.1074-1
Bg-SRR000076.27726-1
Bg-SRR005372.17669-1
Bg-SRR005372.43293-1
Bg-SRR005375.121781-1
Bg-EV823508-1
Bg-c3105
Bg-c14600
Bg-c13095
Bg-c20079
Bg-c23390
Bg-SRR000072.35905-1
Bg-SRR000076.136591-1
Bg-SRR000076.173661-1
Bg-c259
Bg-SRR000072.21928-1
Bg-c35
Bg-c245
Bg-c161
Bg-c7896
Bg-SRR000072.117215-1
Bg-SRR000072.151755-1
Bg-c433
Bg-SRR005375.55337-1
Bg-SRR005375.156773-1
Bg-AW739872-1
Bg-SRR000072.155714-1
Bg-c31305
Bg-c4895
Bg-SRR000072.130410-1
Bg-c17106
Bg-c1109
Bg-SRR000072.20812-1
Bg-c3982
Bg-c10042
Bg-c1963
Bg-c3662
Bg-c40959
Bg-FC856302-1
Bg-SRR000076.6785-1
Bg-SRR005375.49618-1
Bg-c19953
Bg-SRR000072.124504-1
Bg-c3326
Bg-c9736
Bg-SRR005372.48275-1
Bg-SRR005372.86271-1
Bg-c5633
Bg-c338
Bg-c519
Bg-CK988991-1
Bg-SRR000076.176316-1
Bg-SRR005372.58412-1
Bg-c276

Cluster 6

Bg-SRR000076.99547-1
Bg-SRR000076.195661-1
Bg-c22928
Bg-SRR000076.34480-1
Bg-c1879
Bg-c24506
Bg-SRR000076.83659-1
Bg-c858
Bg-c3946
Bg-c31360
Bg-c35643
Bg-c9438
Bg-c7410
Bg-c4318
Bg-c28922
Bg-c18038
Bg-c5647
Bg-SRR000072.57211-1
Bg-DW474464-1
Bg-SRR000072.4749-1
Bg-SRR000072.34190-1
Bg-SRR000076.83114-1
Bg-c151
Bg-c24253
Bg-c1665
Bg-SRR000076.4897-1
Bg-SRR000076.120670-1
Bg-c27964
Bg-c39312
Bg-SRR000072.159421-1
Bg-SRR000072.159400-1
Bg-c33545
Bg-c42057
Bg-c25341
Bg-c666
Bg-SRR000076.121178-1
Bg-c12995
Bg-c3969
Bg-SRR000076.163561-1
Bg-SRR005372.54927-1
Bg-c2551
Bg-SRR000076.147972-1
Bg-c26354
Bg-c235
Bg-c153
Bg-c103
Bg-c838
Bg-EX002685-1
Bg-c40901
Bg-SRR005372.44463-1
Bg-c1515
Bg-SRR000076.133012-1
Bg-CO654053-1
Bg-SRR005372.74149-1
Bg-c1372
Bg-c1040
Bg-c87
Bg-c13477
Bg-ES485333-1
Bg-c854
Bg-c4912
Bg-c2756
Bg-c7222
Bg-c764
Bg-c275
Bg-c20498
Bg-SRR000072.33087-1
Bg-SRR005375.141856-1
Bg-c30949
Bg-c36516
Bg-c17911
Bg-c158
Bg-FC856049-

Cluster 7:

Bg-c9353
Bg-c31063
Bg-CX727727-1
Bg-c9601
Bg-c444
Bg-SRR005375.19157-1
Bg-c10789
Bg-c7880
Bg-c2974
Bg-c113
Bg-AW740476-1
Bg-c9252
Bg-CK990121-1
Bg-c32561
Bg-c15813
Bg-c171
Bg-SRR000072.160241-1
Bg-c207
Bg-c11066
Bg-c31334
Bg-SRR000076.189229-1
Bg-AW740527-1
Bg-c36763
Bg-c37539
Bg-c10244
Bg-c178
Bg-c12140
Bg-SRR005375.62787-1
Bg-SRR005375.103708-1
Bg-c1320
Bg-GH716129-1
Bg-SRR000072.42690-1
Bg-SRR000076.191511-1
Bg-c102
Bg-SRR000072.94258-1
Bg-c215
Bg-SRR000076.44261-1
Bg-SRR000072.10475-1
Bg-SRR000076.53646-1
Bg-SRR000076.2412-1
Bg-c15604
Bg-CO870292-1
Bg-c1410
Bg-c1929
Bg-c6345
Bg-EE722442-1
Bg-AW740286-1
Bg-c3387
Bg-SRR000076.148933-1
Bg-c831
Bg-SRR005375.195258-1
Bg-c9356
Bg-c601
Bg-SRR005372.58379-1
Bg-SRR000076.106568-1
Bg-SRR000076.112129-1
Bg-SRR000072.160747-1
Bg-c38758
Bg-c742
Bg-c384
Bg-c3368
Bg-c1424
Bg-CK989218-1
Bg-c244
Bg-SRR000072.63375-1
Bg-c110
Bg-c499
Bg-c12048
Bg-c10036
Bg-GH717225-1
Bg-c4377
Bg-SRR005372.44544-1
Bg-c2839
Bg-c1580
Bg-c7382
Bg-c5433
Bg-c42903
Bg-c9254
Bg-c3885
Bg-c535
Bg-SRR000072.113533-1
Bg-c1837
Bg-SRR005372.16440-1
Bg-ci38
Bg-c579
Bg-CK988946-1
Bg-c11935
Bg-GH716274-1
Bg-c26290
Bg-EV816166-1
Bg-c452
Bg-c4456
Bg-c30958
Bg-c953
Bg-c872
Bg-c5043
Bg-c14451
Bg-c1361
Bg-c17640
Bg-SRR000076.730-1
Bg-c23408
Bg-c1783
Bg-SRR000072.123361-1
Bg-c2041
Bg-SRR005375.14439-1
Bg-c552
Bg-c173
Bg-c3891
Bg-c1737
Bg-FC858576-1
Bg-c22848
Bg-c12454
Bg-c7702
Bg-c350
Bg-c8291
Bg-SRR000072.93280-1
Bg-c273
Bg-c344
Bg-c208
Bg-c37269
Bg-c1751
Bg-c1482
Bg-c12863
Bg-SRR000076.99488-1
Bg-SRR005372.35048-1
Bg-c52
Bg-c1655
Bg-SRR000072.40117-1
Bg-SRR005372.59423-1
Bg-c42745
Bg-c14760
Bg-c9973
Bg-c481
Bg-c107
Bg-c1742
Bg-c1291
Bg-c4742
Bg-c15414
Bg-c13863
Bg-SRR000076.162365-1
Bg-SRR000076.194407-1
Bg-c2830
Bg-c1752
Bg-SRR005372.87427-1
Bg-c3624
Bg-SRR000076.88103-1
Bg-c13586
Bg-SRR005372.43004-1
Bg-c4855

Cluster 8:

Bg-SRR000072.160090-1
Bg-SRR000075.1160-1
Bg-c464
Bg-c73
Bg-c40073
Bg-SRR005372.63134-1
Bg-EW999773-1
Bg-SRR000072.49350-1
Bg-SRR000076.143533-1
Bg-c8223
Bg-c29111
Bg-c34694
Bg-SRR000074.937-1
Bg-c970
Bg-c42764
Bg-CV548414-1
Bg-c17959
Bg-c4523
Bg-c25834
Bg-c19050
Bg-c17232
Bg-c19916
Bg-c1908
Bg-c83
Bg-c316
Bg-SRR000072.35985-1
Bg-c6900
Bg-AW740308-1
Bg-c22592
Bg-c4343
Bg-EV816067-1
Bg-c1591
Bg-c240
Bg-ES750368-1
Bg-SRR000076.192921-1
Bg-c5180
Bg-c6955
Bg-c1810
Bg-c16424
Bg-c25488
Bg-c30192
Bg-c2160
Bg-c556
Bg-c16870
Bg-c1518
Bg-c3633
Bg-SRR000072.30599-1
Bg-SRR005375.126748-1
Bg-SRR000076.23138-1
Bg-c25
Bg-c23088
Bg-c35540
Bg-c11351
Bg-EV820394-1
Bg-c4207
Bg-c31162
Bg-c19098
Bg-EV818742-1
Bg-c6251
Bg-c5294
Bg-c1019
Bg-SRR005372.5775-1
Bg-c627
Bg-c437
Bg-c3574
Bg-c1764
Bg-c4273
Bg-ci30
Bg-c406

Cluster 9: 

Bg-c3249
Bg-SRR000073.910-1
Bg-FC856406-1
Bg-c10745
Bg-c284
Bg-c784
Bg-SRR000076.75413-1
Bg-c19679
Bg-c30253
Bg-SRR000072.70370-1
Bg-SRR000072.157703-1
Bg-c1979
Bg-c26031
Bg-c3092
Bg-SRR000076.104679-1
Bg-EV820051-1
Bg-c2621
Bg-c23401
Bg-c7559
Bg-c848
Bg-c1132
Bg-c29699
Bg-SRR000076.92504-1
Bg-c38249
Bg-c638
Bg-c25380
Bg-SRR000076.42521-1
Bg-c4282
Bg-c1242
Bg-CK988702-1
Bg-c23740
Bg-c8319
Bg-SRR000072.9609-1
Bg-c190
Bg-c2139
Bg-SRR000076.60015-1
Bg-c458
Bg-SRR000076.110543-1
Bg-c3457
Bg-c82
Bg-c25047
Bg-c16737
Bg-c1006
Bg-c16517
Bg-c5621
Bg-c4602
Bg-c727

Cluster 10:

Bg-c513
Bg-c119
Bg-c16201
Bg-c26041
Bg-c31906
Bg-c281
Bg-c9245
Bg-c743
Bg-c31641
Bg-CV548783-1
Bg-SRR000076.36777-1
Bg-CK989642-1
Bg-SRR005372.62451-1
Bg-c3764
Bg-c16518
Bg-c9043
Bg-c1875
Bg-SRR000072.124946-1
Bg-EV822799-1
Bg-c21825
Bg-c4520
Bg-c32221
Bg-c19389
Bg-c31302
Bg-SRR005375.185136-1
Bg-c202
Bg-c8189
Bg-c2677
Bg-FC855692-1
Bg-c8267
Bg-c7087
Bg-c5505
Bg-SRR000072.158940-1
Bg-c22938
Bg-c11933
Bg-ci68
Bg-c19704
Bg-c2204
Bg-SRR000072.162620-1
Bg-c10140
Bg-c501
Bg-c4716
Bg-c3513
Bg-c210
Bg-c8246
Bg-c108
Bg-c37953
Bg-c768
Bg-c2145
Bg-c290
Bg-c690
Bg-SRR005372.22891-1
Bg-c7550
Bg-c126

Cluster 11:

Bg-c25645
Bg-c19157
Bg-c1102
Bg-c517
Bg-SRR005375.215058-1
Bg-c7568
Bg-SRR000076.163790-1
Bg-c1062
Bg-FC856770-1
Bg-c30201
